# Deletion of the p16^INK4a^ tumor suppressor and expression of the androgen receptor induce sarcomatoid carcinomas with signet ring cells in the mouse prostate

**DOI:** 10.1371/journal.pone.0211153

**Published:** 2019-01-24

**Authors:** Dong-Hong Lee, Eun-Jeong Yu, Joseph Aldahl, Julie Yang, Yongfeng He, Erika Hooker, Vien Le, Jiaqi Mi, Adam Olson, Huiqing Wu, Joseph Geradts, Guang Q. Xiao, Mark L. Gonzalgo, Robert D. Cardiff, Zijie Sun

**Affiliations:** 1 Department of Cancer Biology, Beckman Research Institute, City of Hope, Duarte, California, United States of America; 2 Department of Pathology, Beckman Research Institute, City of Hope, Duarte, California, United States of America; 3 Department of Population Sciences, Beckman Research Institute, City of Hope, Duarte, California, United States of America; 4 Department of Pathology, Keck Medical School, University of South California, Los Angeles, California, United States of America; 5 Department of Urology, Sylvester Comprehensive Cancer Center, University of Miami Miller School of Medicine, Miami, Florida, United States of America; 6 Comparative Medicine, University of California at Davis, Davis, California, United States of America; University of Minnesota Twin Cities, UNITED STATES

## Abstract

The tumor suppressor p16^Ink4a^, encoded by the INK4a gene, is an inhibitor of cyclin D-dependent kinases 4 and 6, CDK4 and CDK6. This inhibition prevents the phosphorylation of the retinoblastoma protein (pRb), resulting in cellular senescence through inhibition of E2F-mediated transcription of S phase genes required for cell proliferation. The *p16*^*Ink4a*^ plays an important role in tumor suppression, whereby its deletion, mutation, or epigenetic silencing is a frequently observed genetic alteration in prostate cancer. To assess its roles and related molecular mechanisms in prostate cancer initiation and progression, we generated a mouse model with conditional deletion of *p16*^*Ink4a*^ in prostatic luminal epithelium. The mice underwent oncogenic transformation and developed prostatic intraepithelial neoplasia (PIN) from eight months of age, but failed to develop prostatic tumors. Given the prevalence of aberrant androgen signaling pathways in prostate cancer initiation and progression, we then generated *R26hAR*^*L/wt*^:*p16*^*L/L*^: *PB-Cre4* compound mice, in which conditional expression of the human *AR* transgene and deletion of *p16*^*Ink4a*^ co-occur in prostatic luminal epithelial cells. While *R26hAR*^*L/wt*^:*PB-Cre4* mice showed no visible pathological changes, *R26hAR*^*L/wt*^:*p16*^*L/L*^: *PB-Cre4* compound mice displayed an early onset of high-grade PIN (HGPIN), prostatic carcinoma, and metastatic lesions. Strikingly, we observed tumors resembling human sarcomatoid carcinoma with intermixed focal regions of signet ring cell carcinoma (SRCC) in the prostates of the compound mice. Further characterization of these tumors showed they were of luminal epithelial cell origin, and featured characteristics of epithelial to mesenchymal transition (EMT) with enhanced proliferative and invasive capabilities. Our results not only implicate a biological role for AR expression and p16^Ink4a^ deletion in the pathogenesis of prostatic SRCC, but also provide a new and unique genetically engineered mouse (GEM) model for investigating the molecular mechanisms for SRCC development.

## Introduction

Mounting evidence has shown aging to be one of the most important risk factors for human prostate cancer (reviewed in [1]). Aging leads to decreased regenerative capability and an increased risk of malignant transformation (reviewed in [2]). The tumor suppressor p16^INK4a^ has been shown to play a critical role in cellular aging and proliferation [3, 4]. An inhibitor of cyclin D-dependent kinases, 4 and 6, p16^INK4a^ prevents phosphorylation of the retinoblastoma protein (pRb), which inhibits the transcription of E2F-regulated genes required for cell cycle entry at the G1/S checkpoint [3, 5]. The suppressive role of p16^Ink4a^ on cell cycle progression is frequently disrupted in tumor cells, either by deletions or inactivating mutations of p16^Ink4a^ [6] or pRb [7]. The expression of p16^Ink4a^ is generally low in normal tissue [8]. The tumor suppressor role of p16^Ink4a^ can be initiated by a number of distinct stimuli, including oncogenic stress, DNA-damage, and aging [9]. It has been shown that deletion of *p16*^*Ink4a*^ occurs in human prostate cancer [10, 11]. Conversely, a suppressive growth effect of the induced expression of p16^Ink4a^ has been observed in a prostate cancer xenograft model [12]. These lines of evidence suggest a pivotal role of p16^Ink4a^ in prostate tumorigenesis.

Androgen signaling, mediated through the AR and its ligands, testosterone and 5α-dihydrotestosterone (DHT), plays a critical role in prostate tumorigenesis [13–17]. The AR is a member of the nuclear hormone receptor superfamily [18, 19]. The unbound AR forms a complex with heat-shock proteins (HSPs) [20, 21]. Upon binding to a ligand, the AR dissociates from the HSPs and translocates into the nucleus, where it binds to the androgen responsive element (ARE), recruits cofactors [22], and activates its targeted gene expression, which in turn controls prostate development and tumorigenesis. Aberrant activation of the AR directly promotes prostate cancer cell growth and survival, and androgen deprivation therapy (ADT) has been a standard treatment for prostate cancer for many decades [23]. However, many patients eventually relapse after therapy and develop castration resistant prostate cancer (CRPC) [13, 15, 24]. An important re-occurring element in prostate cancer is hormone refractoriness, the reemergence of androgen signaling through a variety of molecular mechanisms [13, 15]. Global gene expression profiling has shown the *AR* to be the only gene to be consistently up-regulated in CRPC [24]. Alterations of AR activity through mutation [25], amplification [26], overexpression [24], or cross-talk between AR and other growth factor pathways [27] directly contribute to tumor progression and CRPC development [28].

While abnormal expression and deletion of *p16*^*Ink4a*^ have been observed in human prostate cancers [10, 11], its roles and related molecular mechanisms in prostate cancer initiation and progression are poorly understood. In addition, there is no mouse model that can directly assess the role of p16^Ink4a^ and the collaborative effect of p16^Ink4a^ suppression and AR activation in prostate tumorigenesis. In this study, we first developed a conditional *p16*^*Ink4a*^ deletion mouse strain, in which the mouse *p16*^*Ink4a*^ gene was selectively deleted in prostatic luminal epithelium. Intriguingly, deletion of *p16*^*Ink4a*^ alone was sufficient to induce oncogenic transformation and mouse prostatic intraepithelial neoplasia (PIN) from eight months of age. However, the PIN lesions failed to progress to prostatic tumors in the mice, suggesting that additional hits are required for tumor formation. Given the prevalent role of androgen signaling in prostate cancer initiation and progression, we generated *R26hAR*^*L/wt*^:*p16*^*L/L*^: *PB-Cre4* compound mice, where conditional expression of the human *AR* transgene and deletion of *p16*^*Ink4a*^ co-occur in prostatic luminal epithelial cells. The *R26hAR*^*L/wt*^:*p16*^*L/L*^: *PB-Cre4* compound mice revealed an earlier onset of PIN as well as prostatic carcinomas. Moreover, we identified a rare form of highly malignant tumor, sarcomatoid carcinoma with signet ring cell carcinoma (SRCC) in *R26hAR*^*L/wt*^:*p16*^*L/L*^: *PB-Cre4* compound mice [29–31]. These results not only implicate a biological role for transgenic *AR* expression and *p16*^*Ink4a*^ deletion in the pathogenesis of prostatic SRCC but also provide a new and unique GEM model for investigating the molecular mechanisms for SRCC development.

## Results

### Conditional deletion of *p16*^*Ink4a*^ in mouse prostate epithelium induces oncogenic transformation and prostatic intraepithelial neoplasia formation

**Fig 1:**
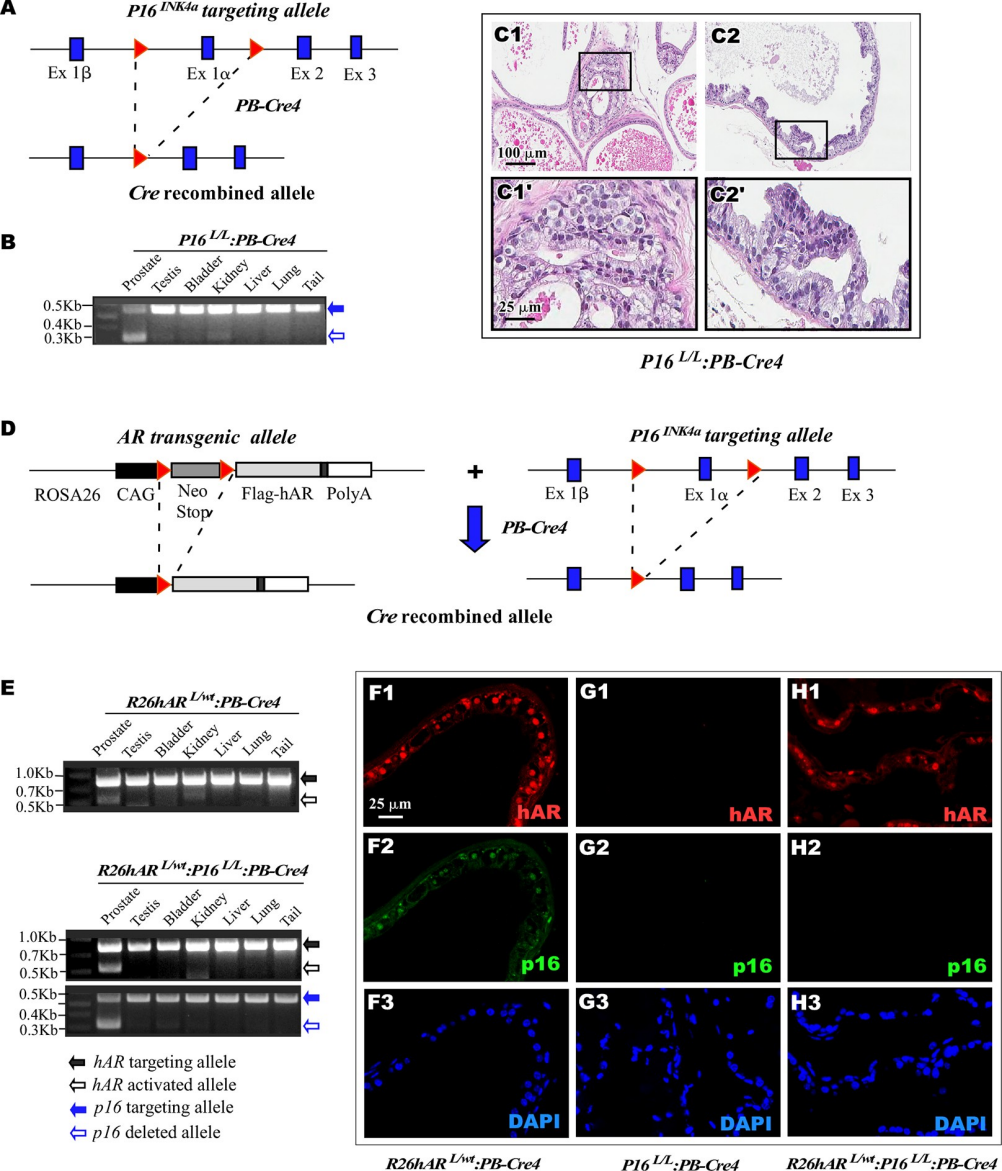
Generation of *p16*^*Ink4a*^ conditional deletion and *AR* conditional transgenic mice. (A) A scheme was shown of the conditional *p16*^*Ink4a*^ deletion target construct. (B) Genomic PCR was used to confirm the *p16*^*Ink4a*^ targeting allele (blue solid arrow) and deleted allele (blue empty arrow) of different mouse tissues. (C) Representative H&E of the prostate tissue was shown for dorsal/lateral prostate (DLP), C1, and ventral prostate (VP), C2, for *p16*^*L/L*^:*PB-Cre4* mice. (D) A scheme was shown of the conditional *AR* transgene target construct and the *p16*^*Ink4a*^ deletion target construct. For the *AR* transgene construct, a PGK-neomycin cassette was flanked with *loxP* sites (LSL), red triangles, inserted between the CAG promoter and a FLAG-tagged *AR* coding sequence. For the *p16*^*nkK4a*^ deletion, two *LoxP* sites, red triangles, were inserted to flank exon 1 alpha. Targeting constructs were shown, upper and Cre-driven recombined alleles for both constructs are shown below. (E) Genomic PCR was used to confirm the *AR* (black solid arrow) or *p16*^*Ink4a*^ (blue solid arrow) targeting allele and *AR* recombined (black empty arrow) or *p16*^*Ink4a*^ deleted (blue empty arrow) allele from different tissues of *R26hAR*^*L/wt*^:*PB-Cre4* and *R26hAR*^*L/wt*^:*p16*^*L/L*^:*PB-Cre4* mice. (F-H) Immunofluorescence (IF) assays. Prostate tissues of *R26hAR*^*L/wt*^:*PB-Cre4*, *p16*^*L/L*^:*PB-Cre4*, *and R26hAR*^*L/wt*^:*p16*^*L/L*^:*PB-Cre4* mice were used for IF analyses and tissues sections were probed for AR (red, F1, G1, H1) or p16^INK4a^ (green, F2, G2, H2) and DAPI (blue, F3, G3, H3), respectively.

**Fig 2:**
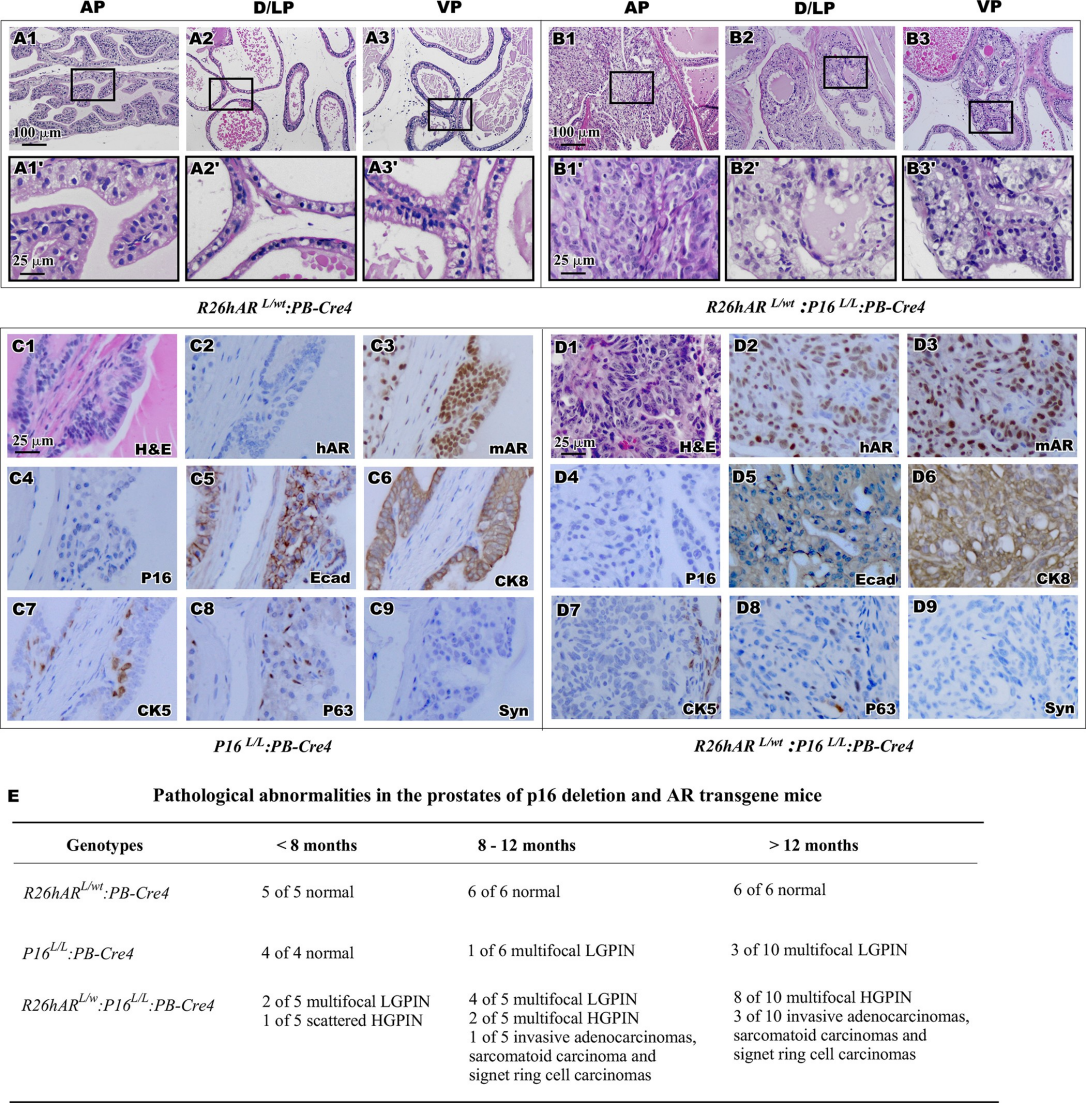
Histological and immunohistochemistry analyses of the prostate from *R26hAR*^*L/wt*^:*PB-Cre4*, *p16*^*L/L*^:*PB-Cre4* and *R26hAR*^*L/wt*^:*p16*^*L/L*^:*PB-Cre4*. (A-B) Representative H&E images of the prostate tissue was shown for AP, DLP, and VP for *R26hAR*^*L/wt*^:*PB-Cre4* (A1-A3, magnification A1’-A3’) and *R26hAR*^*L/wt*^:*p16*^*L/L*^:*PB-Cre4* mice (B1-B3, magnification B1’-B3’). Scale bar used was 100 μm or 25 μm. (C). Representative H&E and IHC images of adjacent prostate tissue sections of AP from the *p16*^*L/L*^:*PB-Cre4* mice were shown for staining with different antibodies as labeled in right bottom corner. (D). Representative H&E and IHC images of adjacent prostate tissue sections from the *p16*^*L/L*^: *R26hAR*^*L/wt*^:*PB-Cre4* mice were shown for staining with different antibodies (please see S1B and S1B’ Fig for positive staining of synaptophysin). Scale bar used was at 25 μm for all panels (C1-D9). (E) Pathological abnormalities of *R26hAR*^*L/wt*^:*PB-Cre4*, *p16*^*L/L*^:*PB-Cre4*, and *R26hAR*^*L/wt*^:*p16*^*L/L*^: *PB-Cre4* mice reported for different time points.

Aberrant expression of p16^Ink4a^ has been detected in human prostate cancer samples [10, 11, 32]. To directly investigate the biological role of p16^Ink4a^ in prostate tumorigenesis, we generated a mouse line, *p16*^*L/L*^:*PB-Cre4*, in which *p16*^*Ink4a*^ was conditionally deleted in prostatic luminal epithelium using a modified probasin promoter, ARR2PB, (Fig 1A) [33]. Using genomic PCR approaches, we confirmed the *Cre/LoxP*-mediated recombination of *p16*^*Ink4a*^ in the mouse prostate (Fig 1B). The *p16*^*L/L*^:*PB-Cre4* offspring were born at the expected Mendelian ratios and appeared normal with no obvious differences from their wild-type littermates at birth. Per the recommendations of the Mouse Models of Human Cancers Consortium Prostate Pathology Committee [34], we assessed *p16*^*L/L*^:*PB-Cre4* mice from birth and identified pathological lesions resembling low-grade prostatic intraepithelial neoplasia (LGPIN) in eight month old *p16*^*L/L*:^*PB-Cre4* mice (Fig 1C). These lesions originated predominantly in the dorsal/lateral prostate (D/LP) and ventral prostate (VP) lobes (Fig 1C1 and Fig 1C2, respectively), consisting of atypical proliferative patterns with occasional stratification of cells and papillary structures. These atypical lesions contained epithelial cells that appeared larger than adjacent normal cells and lacked normal polarity (Fig 1C1’-2’). Development of these lesions in *p16*^*L/L*^:*PB-Cre4* mice suggests a tumor suppressive role of p16^Ink4a^ in prostatic epithelium. However, the animals with these lesions failed to develop prostate tumors after twenty-four months of age (Fig 2E). This implies that other genetic and/or epigenetic alterations are required in combination with *p16*^*Ink4a*^ deletion for the formation of a prostate cancer.

### Simultaneous expression of AR and deletion of p16^Ink4a^ in the prostate accelerates oncogenic transformation in the mouse prostate

Genetic alterations of AR frequently occur during the course of prostate cancer initiation and progression in the presence of other genetic and epigenetic changes [24, 35, 36]. Given the prevalence of alterations in androgen signaling in human prostate cancers, we generated *R26hAR*^*L/wt*^:*p16*^*L/L*^:*PB-Cre4* compound mice, in which conditional *AR* expression and *p16*^*Ink4a*^ deletion co-occur in prostate epithelium (Fig 1D). While the *AR* transgene and/or *p16*^*Ink4a*^ target alleles were detected in various tissues isolated from *R26hAR*^*L/wt*^:*PB-Cre4* and *R26hAR*^*L/wt*^:*p16*^*L/L*^:*PB-Cre4* mice (black or blue solid arrows, Fig 1E), the activated *AR* transgene or *p16*^*Ink4a*^ deleted alleles were detected exclusively in prostate tissues.(Black or blue empty arrows, Fig 1E). Using co-immunofluorescence (co-IF), we assessed the expression of the *AR* transgene and *p16*^*Ink4a*^ deletion in the prostate tissues isolated from mice of each genotype. The expression of transgenic human AR protein was only detected in prostatic epithelial cells of *R26hAR*^*L/wt*^:*PB-Cre4* and *R26hAR*^*L/wt*^:*p16*^*L/L*^:*PB-Cre4* mice (Fig 1F1 and Fig 1H1). Similarly, p16^Ink4a^ expression presented in prostatic luminal epithelium of *R26hAR*^*L/wt*^:*PB-Cre4* mice (Fig 1F2) but was absent in the samples of *p16*^*L/L*^:*PB-Cre4* and *R26hAR*^*L/wt*^:*p16*^*L/L*^:*PB-Cre4* mice (Fig 1G2 and Fig 1H2). These data confirmed conditional expression of the *AR* transgene and deletion of *p16*^*Ink4a*^ co-occur in the prostate of the *R26hAR*^*L/wt*^:*p16*^*L/L*^:*PB-Cre4* compound mice.

The *R26hAR*^*L/wt*^:*p16*^*L/L*^: *PB-Cre4* mice and their littermates, *R26hAR*^*L/wt*^:*PB-Cre4*, were born at the expected Mendelian ratios. They appeared normal with no obvious physical differences from their wild-type littermates at birth. Gross and pathological analyses showed no obvious abnormalities in the prostate of *R26hAR*^*L/+*^:*PB-Cre4* mice (Fig 2E), which is consistent with previous reports [37, 38]. All prostatic lobes displayed normal prostatic epithelium consisting of a single uniform layer of both luminal and basal cells (Fig 2A1-3’). Intriguingly, we observed that *R26hAR*^*L/wt*^:*p16*^*L/L*^:*PB-Cre4* compound mice developed PIN in all prostatic lobes (Fig 2B1-3 versus Fig 1C1-2), suggesting that the atypical lesions observed in the p16 knockouts were indeed precursor lesions. Specifically, pathological changes resembling high grade PIN (HGPIN) occurred within the anterior prostate (AP), containing atypical cells filling the lumen of the ducts, large pleomorphic nuclei with prominent nucleoli, and nuclear hyperchromasia (Fig 2B1-3’). Using immunohistochemical (IHC) approaches, the cellular properties of atypical cells within PIN lesions of *p16*^*L/L*^:*PB-Cre4* mice and *R26hAR*^*L/wt*^:*p16*^*L/L*^: *PB-Cre4* compound mice were assessed. Positive nuclear staining of human AR protein only appeared in atypical cells of the prostate tissues of *R26hAR*^*L/wt*^:*p16*^*L/L*^: *PB-Cre4* compound mice (Fig 2C2 and Fig 2D2). However, positive staining of mouse AR revealed in the nucleus of luminal cells of both *p16*^*L/L*^:*PB-Cre4* (Fig 2C3) and *R26hAR*^*L/wt*^:*p16*^*L/L*^: *PB-Cre4* compound mice (Fig 2D3). Loss of p16^Ink4a^ staining was observed in prostate tissues of both *p16*^*L/L*^:*PB-Cre4* (Fig 2C4) and *R26hAR*^*L/wt*^:*p16*^*L/L*^:*PB-Cre4* compound mice (Fig 2D4), whereas the p16^Ink4a^ expression was detected in *R26hAR*^*L/wt*^:*PB-Cre4* mice (S1A and S1A’ Fig). Most of the atypical cells within PIN lesions from both *p16*^*L/L*^:*PB-Cre4* and *R26hAR*^*L/wt*^:*p16*^*L/L*^: *PB-Cre4* mice showed positive staining for E-cadherin (Fig 2C5 and Fig 2D5) and cytokeratin-8, CK8, (Fig 2C6 and Fig 2D6), which was consistent with their luminal epithelial origin. A scattered subset of cells were immunoreactive to the basal cell markers cytokeratin-5, CK5, (Fig 2C7 and Fig 2D7) and p63 (Fig 2C8 and Fig 2D8), but no staining for the neuroendocrine cell marker synaptophysin (Fig 2C9 and Fig 2D9) was seen in the prostate tissues from mice of either genotype. Taken together, the above results demonstrate that simultaneous expression of *AR* and deletion of *p16*^*Ink4a*^ accelerates, and is necessary for oncogenic transformation in the mouse prostate, and that the atypical cells within the PIN lesions of the both *p16*^*L/L*^:*PB-Cre4* and *R26hAR*^*L/wt*^:*p16*^*L/L*^: *PB-Cre4* mice are prostatic luminal cell in origin.

### Development of prostate tumors possessing simultaneous expression of AR transgene and loss of p16^Ink4a^ expression in prostate epithelium

**Fig 3:**
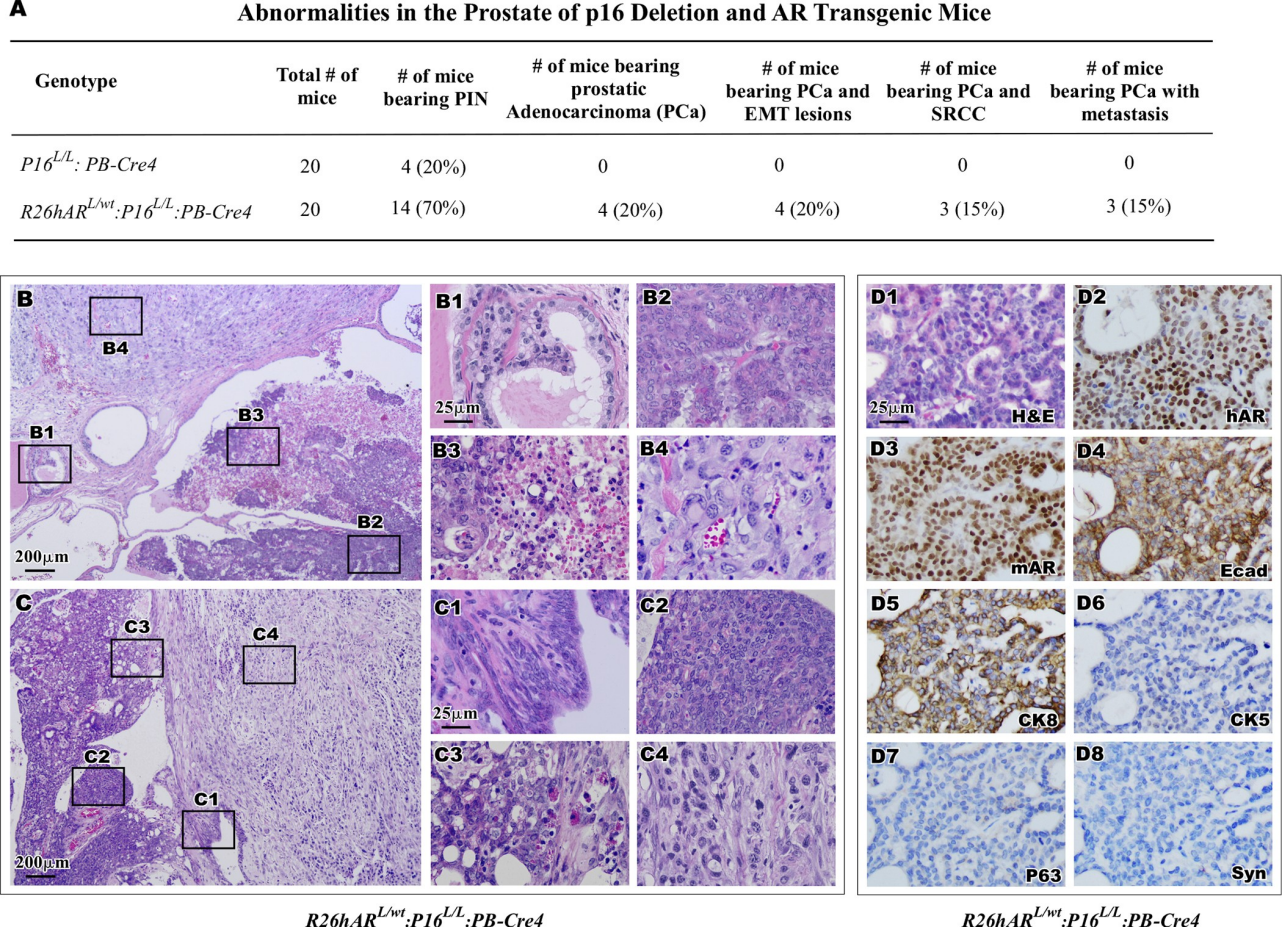
Synergistic effect of conditional *AR* expression and *p16*^*Ink4a*^ deletion accelerates formation of adenocarcinoma. **(**A) Pathological abnormalities of the prostate were listed for both *p16*^*L/L*^:*PB-Cre4* and *R26hAR*^*L/wt*^:*p16*^*L/L*^:*PB-Cre4* mice. (B-C) Representative image of prostatic tumors from *R26hAR*^*L/wt*^:*p16*^*L/L*^:*PB-Cre4* mice. Scale bar used was at 200 μm. B1-C4. Pathological dissemination of typical morphological characteristics of HGPIN (B1, C1) adenocarcinoma (B2, C2 and C3) and regions of necrosis (B3) and sarcomatoid carcinoma (B4, C4). D1-D8. Representative H&E and IHC images of adjacent prostate tissue sections of adenocarcinoma lesions from the *R26hAR*^*L/wt*^:*p16*^*L/L*^:*PB-Cre4* mice were shown for staining with different antibodies (right bottom corner). Scale bar used was at 25 μm for all panels (B1-D8).

Based on the reported observations that HGPIN lesions can develop into prostate adenocarcinoma [39, 40], we continued examining *p16*^*L/L*^:*PB-Cre4* and *R26hAR*^*L/wt*^:*p16*^*L/L*^:*PB-Cre4* mice at advanced age. There was no observable progression of the focal atypical lesions in *p16*^*L/L*^:*PB-Cre4* mice after twenty four months (Fig 2E and Fig 3A), suggesting the deletion of *p16*^*Ink4a*^ alone in the prostate is not sufficient for formation of malignancy in mice. In contrast, prostate carcinoma developed in *R26hAR*^*L/wt*^:*p16*^*L/L*^:*PB-Cre4* compound mice (Fig 3A). The invasive prostatic carcinomas were observed at eight months of age and increased incidence was observed in *R26hAR*^*L/wt*^:*p16*^*L/L*^:*PB-Cre4* mice over twelve months of age (Fig 2E).

Intriguingly, intracystic adenocarcinomas, invasive adenocarcinomas, and sarcomatoid carcinomas were observed (Fig 3B and Fig 3C). The co-existence of these tumor types in the *R26hAR*^*L/wt*^:*p16*^*L/L*^:*PB-Cre4* mice could be evidence of disease progression. These neoplastic lesions included characteristic regions of HGPIN (Fig 3B1 and Fig 3C1), multi-focal, poorly differentiated adenocarcinomas (Fig 3B2 and Fig 3C2), areas of increased proliferative activity and necrosis (Fig 3B3), as well as moderately differentiated adenocarcinoma (Fig 3C3). Adjacent to lesions of intracystic adenocarcinomas were regions classified as invasive sarcomatoid carcinoma, which was the dominant pathologic feature in all cases (Fig 3B4 and Fig 3C4).

The cellular properties of prostatic adenocarcinoma were assessed using IHC in tumor tissues of *R26hAR*^*L/wt*^:*p16*^*L/L*^:*PB-Cre4* mice. A typical nuclear staining for the transgenic AR was observed in most tumor cells, providing a direct link between the expression of the human *AR* transgene and tumor development (Fig 3D2). The tumor cells also showed positive staining for endogenous mouse AR, E-cadherin, and CK8 (Fig 3D3-5), the hallmarks of prostatic luminal epithelium. These tumor cells were negative for the basal epithelial cell markers p63 and CK5 (Fig 3D6-7), as well as for the neuroendocrine cell marker synaptophysin (Fig 3D8). These results demonstrate that the prostatic adenocarcinomas possess the same luminal prostatic cell properties as observed in PIN lesions of *R26hAR*^*L/wt*^:*p16*^*L/L*^:*PB-Cre4* mice, indicating a luminal epithelial origin.

**Fig 4:**
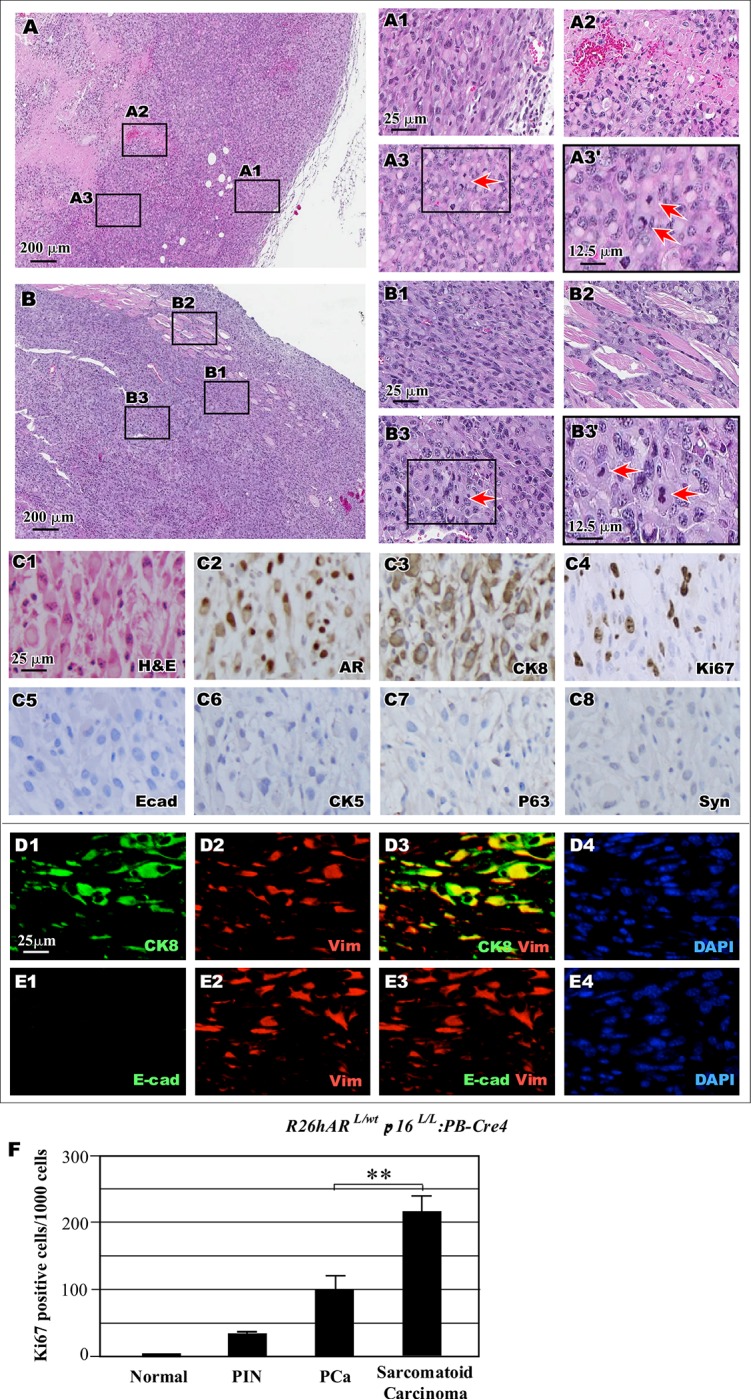
Simultaneous conditional expression of *AR* transgene and loss of *p16*^*Ink4a*^ expression induce sarcomatoid carcinoma development. (A-B) Representative images of two tumor samples display the region of sarcomatoid carcinoma. Scale bar = 200 μm. Representative images of different lesions are shown, including sarcomatoid carcinoma (A1, B1), area of necrosis (A2), invasion into muscle (B2), and images of mitotic figures (A3, B3, see the arrows). (C) Representative H&E and IHC images from the *R26hAR*^*L/wt*^:*p16*^*L/L*^:*PB-Cre4* mice were shown for staining with different antibodies as labeled in the right bottom corner. (D-E) Co-IF from the *R26hAR*^*L/wt*^:*p16*^*L/L*^:*PB-Cre4* mice of secretory epithelial cell marker CK8 (green), mesenchymal cell marker vimentin (red), and DAPI (blue), upper panels; Co-IF of secretory epithelial cell marker E-cadherin (green) and mesenchymal cell marker vimentin (red) and DAPI (blue), lower panels. F. *R26hAR*^*L/wt*^:*p16*^*L/L*^: *PB-Cre4* mouse tissues were stained and quantified for Ki67 for the above tissues were measured in total 1,000 epithelial cells. Representative data shows mean and standard deviation. ** p < 0.005 Scale bar = 25 μm and magnifications were at 12.5 μm (A1-E4).

In this study, diffuse, poorly differentiated lesions with biphasic epithelial and mesenchymal properties were observed in the prostatic stroma of *R26hAR*^*L/wt*^:*p16*^*L/L*^:*PB-Cre4* mice over twelve months of age (Fig 2E). These are classified as sarcomatoid carcinomas and are biphasic malignant neoplasms with features of epithelial-mesenchymal transition (EMT) (reviewed in [34] [41]). As shown in Fig 4A and Fig 4B, these lesions have tumor cells with a spindle-cell morphology (Fig 4A1 and Fig 4B1), focal regions of necrosis (Fig 4A2), and increased mitotic figures (Fig 4A3-3’ and 4B3-3’), indicative of a highly proliferative cell population in these sarcomatoid carcinomas. Invasion of the spindle-like tumor cells into surrounding muscle layers also appeared within the tumor samples (Fig 4B2). Development of this type of invasive prostatic carcinoma in this model suggests a collaborative role of AR activation and p16^Ink4a^ deletion in prostate cancer initiation and progression.

Our observation of sarcomatoid carcinomas adjacent to areas of PIN and invasive adenocarcinoma in the prostate tissues of *R26hAR*^*L/wt*^:*p16*^*L/L*^:*PB-Cre4* mice suggests that the sarcomatoid carcinomas may have developed from PIN and prostatic adenocarcinomas. To test this hypothesis, we assessed the properties of sarcomatoid carcinoma (Fig 4C1). IHC revealed positive nuclear staining for mouse AR in spindle-like tumor cells, suggesting an origin of the AR-positive luminal epithelial cells (Fig 4C2). The tumor cells also showed positive staining for CK8 (Fig 4C3) but not E-cadherin, CK5, p63, or synaptophysin (Fig 4C5-8). Using co-IF, co-expression of CK8 and the mesenchymal cell marker vimentin in tumor cells was observed (Fig 4D1-4). Loss of E-cadherin, an epithelial marker, expression was confirmed in these CK8- and vimentin-positive cells (Fig 4E1-4). The expression of AR and CK8 but loss of E-cadherin suggests that the sarcomatoid carcinomas retain luminal epithelial cell properties and undergo EMT, providing a clear evidence of disease progression.

We examined the proliferative activity of the cells in tumor lesions from *R26hAR*^*L/wt*^:*p16*^*L/L*^: *PB-Cre4* mouse prostates. Using IHC, we measured the expression of the proliferation marker Ki67 in normal and aberrant pathological lesions in each sample. An increase in Ki67 staining in areas of PIN, prostatic adenocarcinoma, and sarcomatoid carcinoma was observed when compared to normal prostatic tissues (Fig 4F). Notably, there were approximately 200 Ki67- positive cells per 1000 cells within the sarcomatoid carcinoma lesions (Fig 4F), which was significantly higher than those in PIN or prostatic adenocarcinoma lesions. These observations are consistent with the frequent appearance of mitotic figures presented in Fig 4A3’ and Fig 4B3’, and demonstrate the highly proliferative nature of the sarcomatoid carcinoma cells.

### Development of signet ring cell carcinoma in the prostate and metastatic sites of *R26hAR*^*L/wt*^:*p16*^*L/L*^:*PB-Cre4* mice

**Fig 5:**
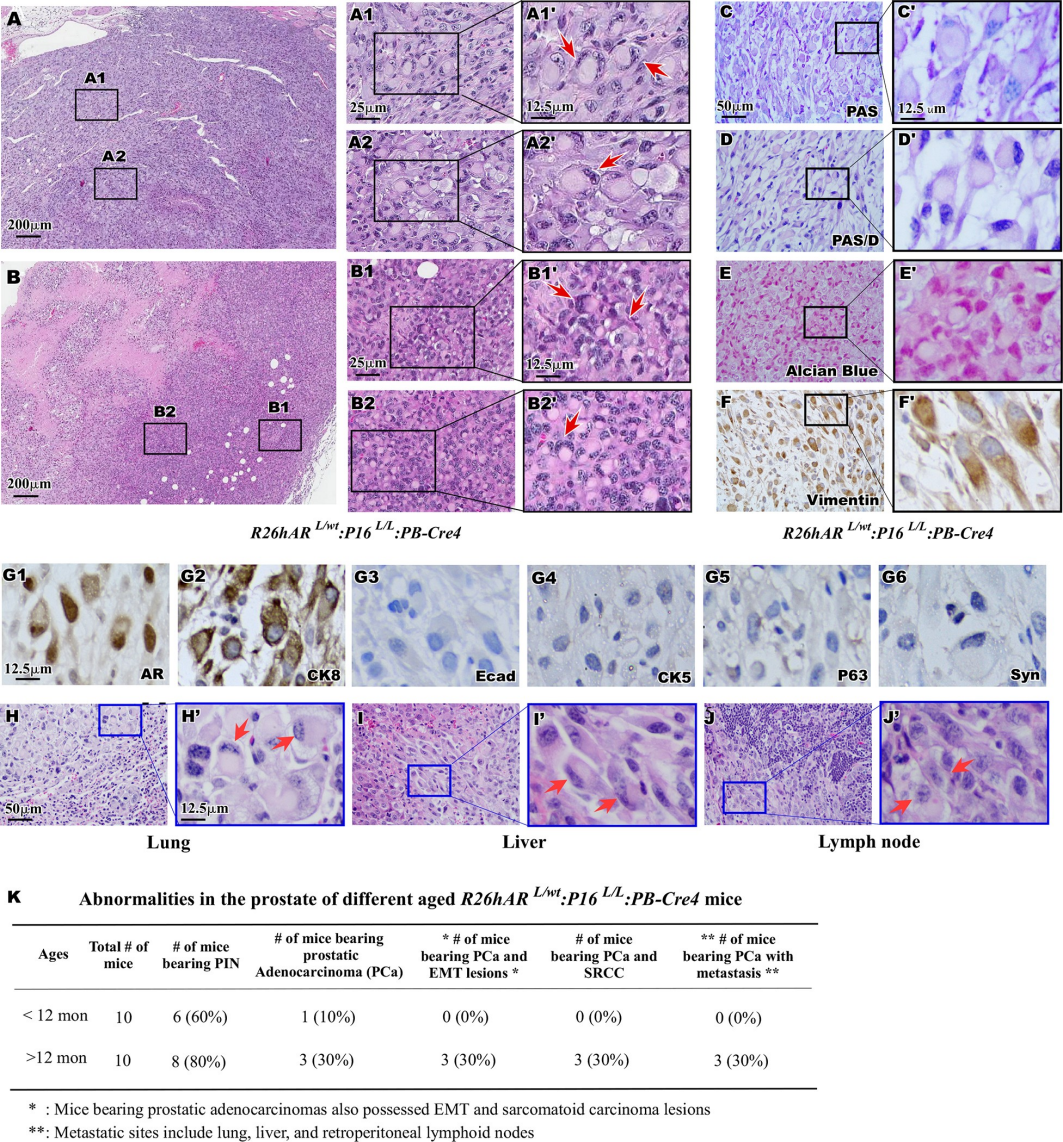
Histological and Immunochemical identification of signet ring cell carcinoma. (A-B) Representative image of the prostate signet ring cell carcinomas, SRCCs, in *R26hAR*^*L/wt*:^*p16*^*L/L*^:*PB-Cre4* mice. Scale bar = 200 μm. A1-B2. Pathological characterization of typical morphological characteristics of SRCC was highlighted in two different tumor samples with magnified views (A1’- B2’). (C-F) Representative view is shown for PAS/PAS (C-D, magnified view C’-D’) staining, Alcian Blue staining (E, magnified view E’), and vimentin immunoreactivity (F, magnified view F’). Scale bar used was at 50 μm and magnified views scale bar used was at 12.5 μm. (G). Representative view of IHC images of adjacent prostate tissue sections of signet ring cell carcinoma lesion from the *R26hAR*^*L/wt*^:*p16*^*L/L*^:*PB-Cre4* mice are shown for staining with different antibodies (right bottom corner). Scale bar used was at 12.5 μm. (H-J) Representative images of H&E of metastatic lesions in the lung (H), liver (I), and Lymph node (J) with magnified views (H’-J’). (K) Pathological abnormalities of the different aged mice were listed for *R26hAR*^*L/wt*^:*p16*^*L/L*^: *PB-Cre4* mice. Scale = 50 μm, or 12.5 μm (C-F, H-J).

Intermixed with regions of both the prostate adenocarcinoma and sarcomatoid carcinoma lesions of *R26hAR*^*L/wt*^:*p16*^*L/L*^:*PB-Cre4* compound mice was a tumor subtype with a particularly unique phenotype. Upon pathological examination of the tumor samples a distinct population of cells resembling signet ring cells (SRC) was observed, featuring large eosinophilic vacuoles displacing the nucleus to the cell periphery [42]. These prostatic SRCs appeared in various proportions in sarcomatoid carcinoma including up to 100% of some regions (Fig 5A1-2 and Fig 5B1-2). High magnification visualization of these tumor lesions showed typical SRC morphology (Arrows, Fig 5A1’-2’ and Fig 5B1’-2’). It has been shown that intracytoplasmic mucins within the large eosinophilic vacuoles were frequently detected in SRCs [43]. To evaluate the status of neutral mucinous components within the SRCs in the *R26hAR*^*L/wt*^: *p16*^*L/L*^:*PB-Cre4* mouse tumors, we performed Periodic Acid Schiff (PAS) and PAS-Diastase (PAS-D) staining. As shown in S1C to S1D’ Fig, positive staining of both PAS and PAS-Diastase were revealed in liver tissues of wild type mice. However, there was no positive staining with PAS and PAS-D in prostate tissues with SRC lesions (Fig 5C’ and Fig 5D’). Staining with Alcian Blue was also negative in the above prostate tissues containing SRCC (Fig 5E and Fig 5E’), in the contrast to the positive control samples (S1E and S1E’ Fig). These results suggest the absence of mucinous components and proteoglycans in SRCs of *R26hAR*^*L/wt*^:*p16*^*L/L*^: *PB-Cre4* mice. Identification of prostatic SRCC in the *R26hAR*^*L/wt*^:*p16*^*L/L*^:*PB-Cre4* mouse model is novel and provides a useful tool for investigating signet ring cell carcinomas in the future. Using IHC approaches, we further assessed the cellular properties of the prostatic SRCs. Notably, the SRCs were immunoreactive with antibodies against vimentin (Fig 5F and Fig 5F’), AR (Fig 5G1), and CK8 (Fig 5G2), but not for E-cadherin (Fig 5G3), CK5 (Fig 5G4), p63 (Fig 5G5), or synapotphysin (Fig 5G6) respectively. These results demonstrate a prostatic SRC expression pattern similar to the spindle-like sarcomatoid carcinoma cells, suggesting that these tumor lesions may be derived from the same cell origin.

During the course of our investigation, we closely monitored the mice for metastases in distant organs. Interestingly, we identified metastatic lesions in three *R26hAR*^*L/wt*^:*p16*^*L/L*^:*PB-Cre4* mice over twelve months of age (Fig 5K). Histological analyses showed metastatic tumor lesions, in the lung (Fig 5H and Fig 5H’), liver (Fig 5I and Fig 5I’), and lymph nodes (Fig 5J and Fig 5J’). Each of these metastatic lesions was composed primarily of spindle-like cells with focal regions of SRCs. IHC analyses of the SRCs in metastatic lesions revealed similar cellular properties to those in the primary tumors of *R26hAR*^*L/wt*^:*p16*^*L/L*^:*PB-Cre4* mice. SRCs in metastatic tissues from the lung were positive for AR and CK8, but negative for E-cadherin and CK5 (S2 Fig). These data confirm the prostatic cell origin of the metastatic disease and provide an additional line of evidence demonstrating the highly aggressive features of prostatic SRCC in *R26hAR*^*L/wt*^:*p16*^*L/L*^: *PB-Cre4* compound mice.

### Identification of downstream targets that promote prostate tumor initiation and progression

**Fig 6:**
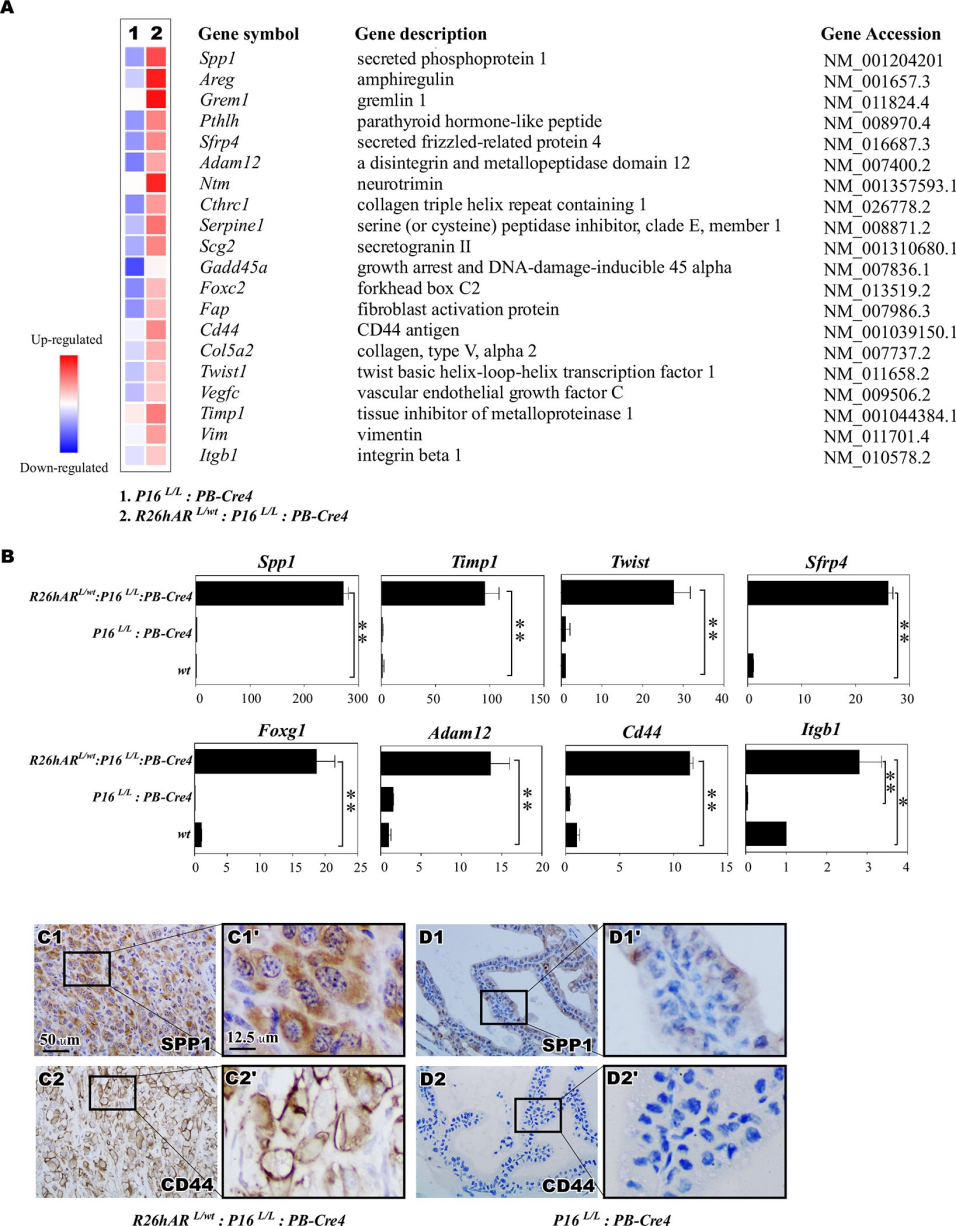
Co-expression of *AR* transgene and loss of *p16*^*Ink4a*^ expression induces an EMT signature. **(**A) Heatmap showing a subset of differentially expressed genes (DEGs) that corresponded to an overlap of hallmark EMT genes comparing *p16*^*L/L*^: *PB-Cre4* and *R26hAR*^*L/wt*^:*p16*^*L/L*^: *PB-Cre4* mice. (B) qRT-PCR confirmation of the gene alterations in prostate tissues of *p16*^*L/L*^: *PB-Cre4* and *R26hAR*^*L/wt*^:*p16*^*L/L*^: *PB-Cre4*. The data is presented as the mean ± (SD) (n = 3) ** p < 0.005 and * p < 0.01 by student’s T test. (C-D) Representative view of IHC images of adjacent prostate tissue sections of sarcomatoid carcinoma lesion from the *R26hAR*^*L/wt*^:*p16*^*L/L*^: *PB-Cre4* mice (C1-C2) and *p16*^*L/L*^: *PB-Cre4* mice (D1-D2) and their magnified views (C1’-D2’). Scale bar = 50 μm or 12.5 μm.

In search for the molecular basis by which transgenic *AR* expression and *p16*^*INK4a*^ deletion regulate prostate tumorigenesis, we performed RNA-sequencing analysis to examine the global transcriptome profiles in the mouse prostate tumor samples. Given that HGPIN and prostate tumor development respectively occurred in *p16*^*L/L*^:*PB-Cre4* and *R26hAR*^*L/wt*^:*p16*^*L/L*^:*PB-Cre4* mice, we focused specifically on those mice. RNA transcriptome profiling yielded differentially expressed genes (DEGs) of 960 genes that were upregulated with a fold change ≥ 5 and 537 genes that were downregulated at a fold change ≥ 5 from samples of the *p16*^*L/L*^:*PB-Cre4* and *R26hAR*^*L/wt*^:*p16*^*L/L*^: *PB-Cre4* mice in comparison to those from age and sex-matched wild type littermates (S1 Table). The change in expression of these genes reflects the activity of either *p16*^*Ink4a*^ deletion alone or in combination with transgenic *AR* expression in the mouse prostate. According to gene set enrichment analysis (GSEA), the hallmark EMT signature was the most significantly enriched in the *R26hAR*^*L/wt*^:*p16*^*L/L*^:*PB-Cre4* compound mice (S3B Fig) [44]. Forty-nine DEGs within our data set overlapped with the hallmark EMT signature gene set (200 genes) (S3A Fig). Using real-time quantitative RT-PCR, qRT-PCR, alteration of a subset of these genes was validated (Fig 6A). We confirmed the statically significant upregulation of *Spp1*, *Timp1*, *Twist*, *Sfrp4*, *Foxg1*, *Adam12*, *Cd44*, *and Itgb1* in the *R26hAR*^*L/wt*^:*p16*^*L/L*^:*PB-Cre4* mice in comparison to *p16*^*L/L*^: *PB-Cre4* and wild type mice (Fig 6B). IHC analyses showed increased expression of Spp1 and CD44 in the prostates of the *R26hAR*^*L/wt*^:*p16*^*L/L*^:*PB-Cre4* mice relative to the *p16*^*L/L*^: *PB-Cre4* mice (Fig 6C1-2’ versus Fig 6D1-2’). The above data provide a molecular basis for the effect of transgenic *AR* expression and *p16*^*Ink4a*^ deletion in prostate cancer progression.

## Discussion

Loss of the CDK4/6 inhibitor, p16^INK4a^, due to its deletion or reduced expression through promoter hyper-methylation, has been observed in human prostate cancer samples [32, 45, 46]. Although the oncogenic role of p16^Ink4a^ loss has been investigated in several tumor types [47–49], the loss of the tumor suppressor in prostate tumorigenesis has not been evaluated in a relevant mouse model. In this study, we developed a novel mouse model, *p16*^*L/L*^:*PB-Cre4* mice, where the *p16*^*Ink4a*^ gene was selectively deleted in prostatic luminal epithelial cells through *Cre-LoxP*-mediated recombination. We observed the development of mouse prostatic intraepithelial neoplasia in *p16*^*L/L*^:*PB-Cre4* mice at eight months of age, providing the first line of evidence demonstrating that *p16*^*Ink4a*^ deletion can initiate oncogenic transformation and PIN formation in the mouse prostate. In this study, we did not observe further severe pathologic changes in *p16*^*L/L*^:*PB-Cre4* mice, suggesting that deletion of *p16*^*Ink4a*^ alone is not sufficient to induce progression to prostate cancer. The precise mechanisms for this observation are currently unclear, potentially requiring additional genetic alterations for p16^Ink4a^-mediated tumor development in the prostate. The current mouse model, *p16*^*L/L*^:*PB-Cre4* mice, is a biologically relevant experimental tool for identification of such alterations in the initiation of malignant transformation.

Aberrations of androgen signaling have been frequently identified in prostate cancer. Specifically, *AR* gene amplification or upregulation of the *AR* gene expression appears along with other genetic and epigenetic alterations in human prostate tumor tissues [35, 36]. Given the significance of both AR and p16^Ink4a^ alterations in human prostate cancers, we developed *R26hAR*^*L/wt*^:*p16*^*L/L*^:*PB-Cre4* compound mice in which the deletion of the *p16*^*Ink4a*^ gene and conditional expression of the human *AR* transgene co-occur in the prostatic luminal epithelium. We observed accelerated PIN development in *R26hAR*^*L/wt*^:*p16*^*L/L*^:*PB-Cre4* compound mice. We did not find any significant abnormality in the *R26hAR*^*L/wt*^:*PB-Cre4* mice. In contrast to 20% of the *p16*^*L/L*^:*PB-Cre4* mice developing PIN lesions, the *R26hAR*^*L/wt*^:*p16*^*L/L*^:*PB-Cre4* compound mice showed PIN penetrance of 70%. This evidence demonstrates that the combined upregulation of AR and deletion of p16^INK4a^ enhances oncogenic transformation in mouse prostate epithelium. Intriguingly, about 20% of the compound mice developed prostatic invasive carcinomas, sarcomatoid carcinomas, SRCCs, and metastatic tumors. Despite the aggressiveness of the above tumor phenotypes, the level of penetrance suggests that other genetic and epigenetic factors are required for tumor formation, progression, and metastasis.

In this study, we also observed aged mice showing increased tumor incidence and more aggressive tumor phenotypes, indicating a clear inverse correlation between aging and prognosis in the compound mice. All mice over 12 months of age bearing adenocarcinomas also developed invasive sarcomatoid carcinoma with SRCC, and metastatic disease. Not only do these data demonstrate a collaborative effect of *AR* expression and deletion of *p16*^*INK4a*^ in promoting prostate cancer initiation and progression, but also provide a new and biological relevant mouse model that mimics the age-dependent human prostate cancer phenotypes.

As described in this study, we observed a variety of pathologic changes in the prostate of *R26hAR*^*L/wt*^:*p16*^*L/L*^:*PB-Cre4* compound mice. In addition to HGPIN and prostatic adenocarcinomas, we also identified sarcomatoid carcinoma that was the dominant pathologic feature and signet ring cell carcinoma lesions in the compound mice. Prostatic sarcomatoid carcinomas included spindle-like cells with highly proliferative and aggressive growth features, and invasion into the neighboring stroma. IHC analyses showed positive staining for AR in tumor cells within sarcomatoid carcinoma lesions, indicating that they originated from prostatic epithelium and, perhaps, PIN and/or prostatic adenocarcinomas. In agreement with these observations, earlier clinical studies have shown that prostate cancer patients with sarcomatoid carcinomas also had prior history and diagnosis of prostatic adenocarcinoma [50, 51]. A study of 42 patients showed approximately 66% of prostatic sarcomatoid cancer patients had prior pathologic diagnoses of prostate adenocarcinomas [52]. Deletion of prostate-specific *erythroblast transformation-specific-related* gene (*ERG*) has been detected in both the sarcomatoid and adjacent prostate adenocarcinoma, indicating that these two tumor types may be derived from the same cell origin [53]. We also detected positive staining for vimentin, a mesenchymal marker, in prostatic sarcomatoid tumor cells in the compound mice, suggesting the that these sarcomatoid carcinomas are undergoing the EMT process.

Most notably from this study, we identified an tumor type pathologically resembling signet ring cell carcinoma (SRCC), found as a part of the sarcomatoid carcinoma lesions in the *R26hAR*^*L/wt*^:*p16*^*L/L*^:*PB-Cre4* compound mice. SRCC is a rare type of highly malignant adenocarcinoma in humans but has not been described in mice. It is of epithelial origin and is characterized by cells featuring a crescent-shaped nucleus compressed against the cell membrane due to a large cytoplasmic vacuole [54]. Primary SRCC tumors are most often observed in the glandular cells of the stomach but also can be found in other tumor types, including prostate cancer [43, 55]. SRCC possesses invasive characteristics and is capable of colonizing at distant sites [29, 31, 56]. Previous studies have shown prostatic SRCCs to be highly aggressive with poor survival rates [29–31, 55].

As shown in this study, we observed tumor lesions containing the morphology of SRC in the prostate of *R26hAR*^*L/wt*^:*p16*^*L/L*^:*PB-Cre4* compound mice. IHC analyses further showed the prostatic SRCs to be AR and CK8-positive, suggesting they are derived from prostatic luminal cells. The positive staining with vimentin in the SRCs further indicates the EMT features of SRCs. We also identified SRCs in metastatic tumor sites, including the lung, liver, and lymph nodes. These multiple lines of experimental evidence indicate that prostatic SRCC in *R26hAR*^*L/wt*^:*p16*^*L/L*^:*PB-Cre4* compound mice share many pathological and biological features with human SRCCs. In this study, we also assessed the alterations of AR and p16^Ink4a^ in human prostatic SRCC samples. Interestingly, from a human tissue microarray, we found 7 of 7 samples displayed positive staining for AR, and 5 out of 7 showed absence or reduced staining for p16^Ink4a^ (S2 Table). These data imply a possible role of AR and p16^Ink4a^ in SRCC pathogenesis, and warrant further investigation of patient samples.

Observation of sarcomatoid carcinoma and SRCC lesions with invasive and metastatic features in *R26hAR*^*L/wt*^:*p16*^*L/L*^:*PB-Cre4* compound mice supports the pattern of tumor progression and EMT development. This suggests a collaborative role of transgenic *AR and p16*^*Ink4a*^ in enhancing tumor cell transdifferentiation and EMT promotion. We assessed this collaborative program by RNA sequencing and quantified real-time PCR approaches to analyze the changes of the transcriptional profiles from the prostate tumor samples isolated from *p16*^*L/L*^:*PB-Cre4 and R26hAR*^*L/wt*^:*p16*^*L/L*^:*PB-Cre4 mice*. We observed an increase in the expression of a cluster of genes related to EMT and tumor progression in tumor samples isolated from *R26hAR*^*L/wt*^:*p16*^*L/L*^:*PB-Cre4* compound mice in comparison to those from *p16*^*L/L*^:*PB-Cre4* mice. All together these data provide the molecular basis for future investigations to identify the specific regulators and signaling pathways that directly contribute to prostate cancer initiation and progression in *R26hAR*^*L/wt*^:*p16*^*L/L*^:*PB-Cre4* compound mice.

## Materials and methods

### Ethics statement

All animal experiments performed in this study were approved by the ethics committee of the Administrative Panel on Institutional Animal Care and Use Committee at Beckman Research Institute/City of Hope.

### Mouse mating and genotyping

All mice that were used in this study were from a C57BL/6 background. Mice containing the conditional *p16*^*Ink4a*^ allele (*p16*^*LoxP/LoxP*^), also named *p16*^*L/L*^, were kindly provided by Dr. Sharpless at University of North Carolina [57]. The *AR* transgenic mouse strain, *R26hAR*^*LoxP/wt*^ (*R26hAR*^*L/wt*^) was generated previously [38]. Prostate specific conditional knockout mice were generated using *PB-Cre4* mouse strain [33]. We generated *p16*^*L/wt*^:*R26hAR*^*L/wt*^ mice by intercrossing *p16*^*L/L*^ with *R26hAR*^*L/wt*^ mice. The experimental mice were produced from intercrossing of *p16*^*L/wt*^: *R26hARL*^*/wt*^ and *p16*^*L/wt*^: *PB-Cre4* mice to generate three goal mice with genotypes: *R26hAR*^*L/wt*^: *PB-Cre4*, *p16*^*L/L*^: *PB-Cre4*, and *p16*^*L/L*^:*R26hAR*^*L/wt*^: *PB-Cre4*. Mice were genotyped by PCR as described previously [38, 57]. Detection of the *AR* targeting allele used the forward primer, 5’-TTCGGCTTCTGGCGTGTGAC-3’ with the reverse primer, 5’-CTCTGGAACAGATTCTGGAAA-3’ and 5’-AGCGCATCGCCTTCTATCGCCTTC-3’. The *p16*^*Ink4a*^ conditional allele and deleted allele were detected with the forward, 5’-TACCACAGTTTGAACAGCGTGA-3’ with the reverse primer, 5’-AACCAACTTCCTCCTTCCCC-3’, and 5’-CCAAAACCACAACAGCTAGGA-3’. Genomic PCR conditions for *AR* recombined allele were 95°C for 3 min, then 95°C for 30 sec, 64°C for 30 sec, and 72°C for 60 sec for 35 cycles, then 72°C for 5 min. Genomic PCR conditions for *p16*^*Ink4a*^ amplified at 95°C for 3 min, then 95°C for 45 sec, 58°C for 40 sec, and 72°C for 60 sec for 35 cycles, then 72°C for 5 min.

### Histology and Immunohistochemistry, Immunofluorescence, PAS and PAS/D, Alcian Blue staining and histological analysis

The new guidelines recommended by The Mouse Models of Human Cancers Consortium Prostate Pathology Committee in 2013 were used for our pathological analyses [34]. Mouse tissues were fixed in 10% neutral-buffered formalin and processed into paraffin as previously described [38]. Mouse tissues were cut using 4 μm serial sections and rehydrated through a decreasing ethanol gradient. Histology was visualized by hematoxylin-eosin (H&E) staining performed as reported earlier [38]. Immunohistochemistry (IHC) included boiling the tissue in 0.01 M citrate buffer (pH 6.0) for antigen retrieval, and then incubated in 0.3% H_2_O_2_ for 15 min. Samples were blocked in 5% normal goat serum for 30 min and then incubated with primary antibodies diluted in 1% normal goat serum at 4°C overnight. Slides were incubated with biotinylated secondary antibodies for 1 hour, and then incubated with streptavidin ligated to horse radish peroxidase (HRP) (SA-5004, Vector Laboratories) for 30 min. Detection of HRP was with the 3,3'-diaminobenzidine (DAB) kit (SK-4100, Vector Laboratories). Nuclei were counterstained with 5% (w/v) Harris Hematoxylin and then mounted with Permount Medium (SP15-500, Fisher Scientific). Immunofluorescence (IF) of tissue samples were processed the same for IHC, except that the samples were incubated with fluorescent-conjugated secondary antibodies for 1 hour, and then mounted with Vectashield Mounting Medium with DAPI (H-1200, Vector Laboratories).

PAS staining was performed with the addition of 0.5% periodic acid (19840–0050, Acros) for 5 min at room temperature and then washed with distilled water. The tissue glycogen was digested with 0.5% Diastase (PAS/D) (AHDIA50, American MasterTech). Tissues were then incubated in Schiff ‘s reagent (26312-1A, Electron Microscopy Sciences) for 15 min at room temperature and then washed with tap water. Nuclei were counterstained with 5% (w/v) Harris Hematoxylin and mounted with Permount Medium (SP15-500, Fisher Scientific). Tissue samples for Alcian Blue staining were incubated in 1% Alcian Blue and 3% Acetic Acid at pH 2.5 for 30 min and then washed in distilled water. Counterstaining with nuclear-fast red solution was performed. After dehydration and clearing, the slides were mounted with Permount Medium (SP15-500, Fisher Scientific).

### Antibodies

The primary antibodies used and their subsequent dilutions were: anti-P16 (rabbit, 1:1000, SC-1207, Santa Cruz), anti-human AR (mouse, 1:250, sc-7305, Santa Cruz Biotechnology), anti-CK5 (rabbit, 2400, PRB-160P, Covance), anti-CK8 (mouse, 1:2000, MMS-162P, Covance), anti-p63 (mouse, 1:2000, sc-8431, Santa Cruz), anti Ki67 (mouse, 1:1000, NCL-ki67, Novacastra), anti-E-cadherin (mouse, 1:200, Cat. No. c20820, BD Transduction Laboratoriesc, Sparks, MD, United States), anti-mouse/ human androgen receptor (rabbit, 1:250, sc-816, Santa Cruz), anti-synaptophysin (rabbit, 1:100, Cat. No. 18–0130, Invitrogen), anti-SPP1 (rabbit, 1:200, Cat. No. 91655, Abcam, Cambridge, MA, USA), CD44 (Rat, 1:50, sc-18849, Santa Cruz) and anti-Vimentin (Chicken, 1:2000, Cat. No. 919101, Biolegend). The biotinylated anti-rabbit or anti-mouse secondary antibody (BA-1000 or BA-9200, Vector Laboratories), or anti-rabbit or anti-mouse conjugated to AlexaFluor488 or to AlexaFluor594 (Molecular Probes) secondary antibody that were used for IHC or IF staining, respectively.

### Microscope image acquisition

Images of H&E and IHC were acquired on an Axio Lab. A1 microscope using 10x and 40x Zeiss A-Plan objectives with a Canon EOS 1000D camera and using Axiovision software (Carl Zeiss). Images of immunofluorescence staining were acquired on a Nikon ECLIPSE E800 epi-fluorescence microscope using 20x and 40x Nikon Plan Fluor objectives with an QImaging RETIGA EXi camera and using QCapture software (QImaging).

### RNA isolation and qRT-PCR assay

Mouse prostate tissues were homogenized in RNA-Bee (TEL-TEST, Inc., Friendswood, TX, USA), and total RNA was isolated as recommended by the manufacturer. Reverse transcription was carried out following our previous report [58]. For quantitative PCR, cDNA samples were mixed with SYBR greenER qPCR Super Mix Universal (11762, Invitrogen) and performed quantitative RT PCR according to the manufacture’s protocol. Relative mRNA levels were calculated by Delta Delta C(T) methods (KJ Livak, 2001, Method). Reactions were done in triplicate, and the values were normalized by PPIA (peptidylprolyl isomerase A) expression levels. Primers for SPP1 (5’- ATCTCACCATTCGGATGAGTCT- 3’; 5’- TGTAGGGACGATTGGAGTGAAA-3’), Timp1 (5’- TCTGGCATCCTCTTGTTGCT-3’; 5’- CATGAATTTAGCCCTTATGACCAG-3’), Twist (5’- GCTACGCCTTCTCCGTCTG -3’; 5’- GAAACAATGACATCTAGGTCTCCG-3’), Sfrp4 (5’- GTCACTGACCTTCCAGAAGATGT-3’; 5’- CCTTTTTGCACTTGCACCGA-3’), Adam12 (5’- TGGGGATGTGCCTCTTCAAC-3’; 5’- ATTCGTGCATTCCTCCGGTT-3’), Cd44 (5’- TCGATTTGAATGTAACCTGCCG-3’; 5’- CAGTCCGGGAGATACTGTAGC-3′), Itgb1 (5’- GGACGCTGCGAAAAGATGAA; 5’- CATTCTCCGCAAGATTTGGCA-3’), Foxc2 (5’- GCAACCCAACAGCAAACTTTC; 5’- GACGGCGTAGCTCGATAGG-3’) were synthesized and used in the qPCR reaction, respectively.

### RNA sequencing

RNA sequencing was performed by the City of Hope Integrative Genomics core facility. cDNA synthesis and library preparation was performed using Kapa RNA mRNA HyperPrep kit (Kapa Biosystems, Cat KR1352) in accordance with the manufacturer supplied protocols. For gene set enrichment analysis (GSEA), genes were pre-ranked by the signed log 2 fold change. The preranked data were uploaded to GSEA and enrichment of hallmark gene sets were interrogated with 1,000 random permutations to obtain the P value, q value and normalized enrichment score (NES).

### Statistical analyses

Cell numbers were counted manually using 40x photomicrographs as described in Fig 4F. Statistical analyses were performed using 2-tailed Student’s t test. The date was presented as the means ± S.D and cell numbers were made comparisons between groups, using a Student’s *t* test. *p* < 0.05 were considered significant.

## Supporting information

S1 Fig Immunohistochemical staining of P16Ink4a expression and neuroendocrine cell staining, and mucin staining. (A) Representative P16^Ink4a^ IHC images of prostate sample from the *R26hAR*^*L/wt*^: *PB-Cre4* mice. Scale bar = 100 μm or 25 μm. (B) IHC analyses of synaptophysin staining using mouse tissues as positive controls. Representative IHC images of positive synaptophysin staining showed in the cytoplasm of scattered neuron cells in the urethral urothelial and submucosal glands in mouse tissues samples as reported early (Toxicologic Pathology, 2005, 33:386–397). Scale bar = 100 μm or 25 μm. (C-D) Representative Periodic Acid Schiff (C-C’) and PAS-Diastase (D-D’) staining images from liver tissues of wild type mice were shown as positive controls in this study. Scale bar = 100 μm or 25 μm. (E) Representative Alcian Blue staining images from intestine tissues of wild type mice were shown as positive controls in this study. Scale bar = 100 μm or 25 μm.
(PDF)

S2 Fig Immunohistochemical staining of lung metastasis from the *AR* transgene and loss of *p16^Ink4a^* compound mice. **(**A-F) Representative H&E and IHC images of lung metastasis sample from the *R26hAR*^*L/wt*^:*p16*^*L/L*^: *PB-Cre4* mice were shown for staining with different antibodies (right bottom corner). Scale bar = 50 μm.
(PDF)

S3 Fig Heatmap of the Hallmark EMT genes enriched in the *AR* transgene and loss of *p16^Ink4a^* compound mice. **(**A) A heatmap of 49 DEGs ≥ 5 fold in the *R26hAR*^*L/wt*^:*p16*^*L/L*^: *PB-Cre4* mice that overlapped with the list of hallmark EMT genes are listed with the accession numbers of each gene. This gene list was generated through GSEA pre-ranked analysis [44] of the DEGs that were altered comparing *p16*^*L/L*^:*PB-Cre4* and *R26hAR*^*L/wt*^:*p16*^*L/L*^: *PB-Cre4* mice. (B) Gene set Enrichment analysis (GSEA) plot of Hallmark EMT gene set, NES = 2.85 FDR (q-value) <0.000001.
(PDF)

S1 Table RNA-seq data with Log2 fold change for comparison of *p16^L/L^:PB-Cre4* and *R26hAR^L/wt^:p16^L/L^: PB-Cre4*. Expression values were normalized to wild type mice and log2 transformed for the comparison between the RNA transcriptome alteration between *p16*^*L/L*^:*PB-Cre4* and *R26hAR*^*L/wt*^:*p16*^*L/L*^: *PB-Cre4* mice.
(PDF)

S2 Table Cellular properties of human SRCC from USC Keck School of Medicine cohort of prostate cancer patients displaying SRCC. Seven clinical prostatectomy specimens with signet ring prostatic carcinoma component were mounted on one TMA (tissue microarray) and analyzed for AR, p16, CK8, CK5 and SPP1. “+” indicates pathologist determined classification of presence of staining, while “–”indicates pathologist determined absence of staining.
(PDF)
