# Supplementary figures and images for: Deletion of the p16INK4a tumor suppressor and expression of the androgen receptor induce sarcomatoid carcinomas with signet ring cells in the mouse prostate
Source: PLoS One. 2019 Jan 24;14(1):e0211153. doi: 10.1371/journal.pone.0211153 (PMC6345450; doi:10.1371/journal.pone.0211153)

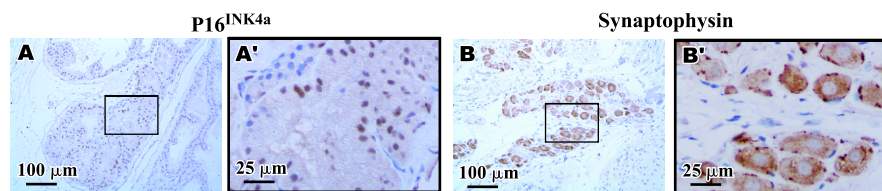

*R26hAR<sup>L/wt:PB-Cre4</sup>*

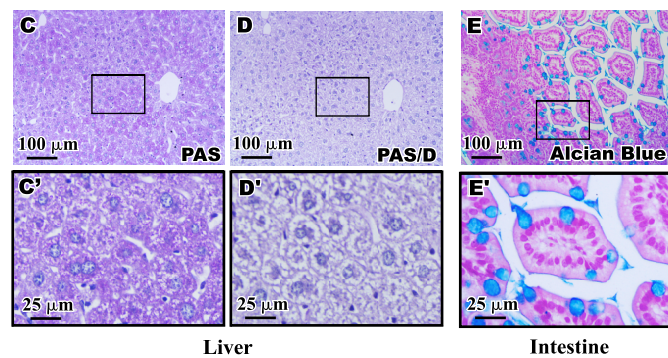

Supplement: S1 Fig — (A) Representative P16Ink4a IHC images of prostate sample from the R26hARL/wt: PB-Cre4 mice. Scale bar = 100 μm or 25 μm. (B) IHC analyses of synaptophysin staining using mouse tissues as positive controls. Representative IHC images of positive synaptophysin staining showed in the cytoplasm of scattered neuron cells in the urethral urothelial and submucosal glands in mouse tissues samples as reported early (Toxicologic Pathology, 2005, 33:386–397). Scale bar = 100 μm or 25 μm. (C-D) Representative Periodic Acid Schiff (C-C’) and PAS-Diastase (D-D’) staining images from liver tissues of wild type mice were shown as positive controls in this study. Scale bar = 100 μm or 25 μm. (E) Representative Alcian Blue staining images from intestine tissues of wild type mice were shown as positive controls in this study. Scale bar = 100 μm or 25 μm. (PDF) [file pone.0211153.s001.pdf]

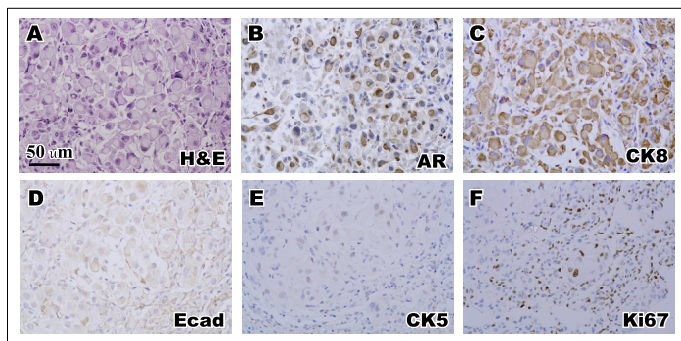

Supplement: S2 Fig — (A-F) Representative H&E and IHC images of lung metastasis sample from the R26hARL/wt:p16L/L: PB-Cre4 mice were shown for staining with different antibodies (right bottom corner). Scale bar = 50 μm. (PDF) [file pone.0211153.s002.pdf]

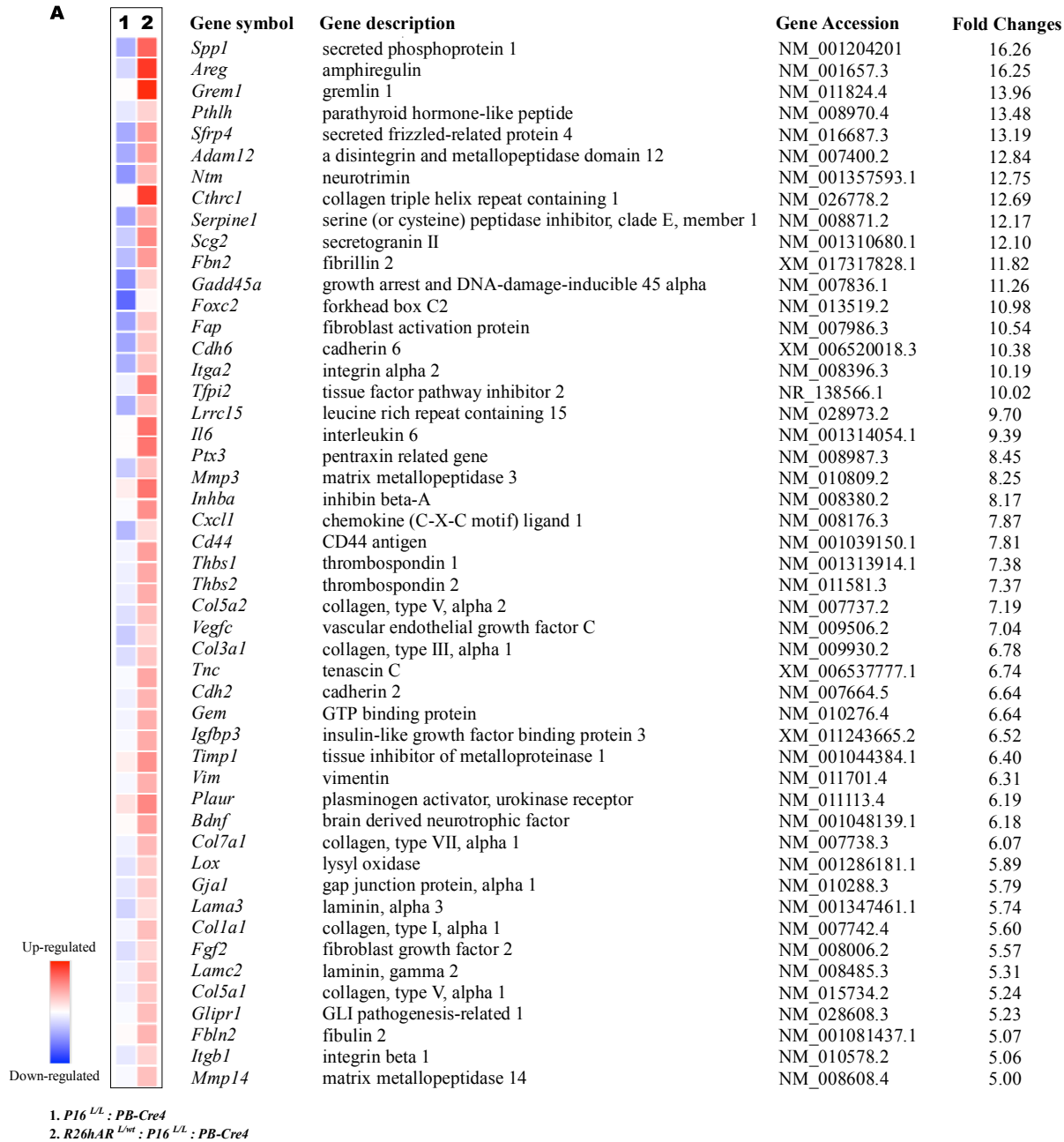

**B**

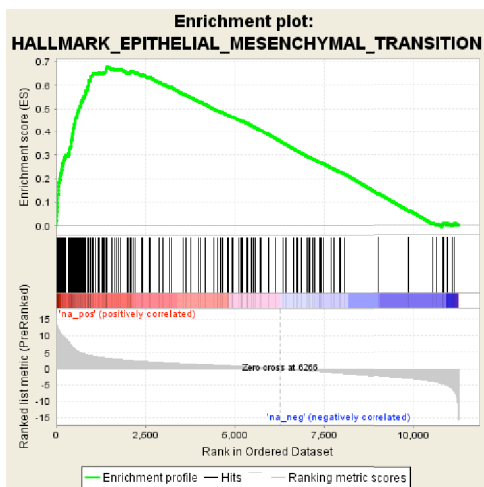

Supplement: S3 Fig — (A) A heatmap of 49 DEGs ≥ 5 fold in the R26hARL/wt:p16L/L: PB-Cre4 mice that overlapped with the list of hallmark EMT genes are listed with the accession numbers of each gene. This gene list was generated through GSEA pre-ranked analysis [44] of the DEGs that were altered comparing p16L/L:PB-Cre4 and R26hARL/wt:p16L/L: PB-Cre4 mice. (B) Gene set Enrichment analysis (GSEA) plot of Hallmark EMT gene set, NES = 2.85 FDR (q-value) <0.000001. (PDF) [file pone.0211153.s003.pdf]
