# Supplementary material for: Deletion of the p16INK4a tumor suppressor and expression of the androgen receptor induce sarcomatoid carcinomas with signet ring cells in the mouse prostate
Source: PLoS One. 2019 Jan 24;14(1):e0211153. doi: 10.1371/journal.pone.0211153 (PMC6345450; doi:10.1371/journal.pone.0211153)
Supplement: S1 Table — Expression values were normalized to wild type mice and log2 transformed for the comparison between the RNA transcriptome alteration between p16L/L:PB-Cre4 and R26hARL/wt:p16L/L: PB-Cre4 mice. (PDF) [file pone.0211153.s004.pdf]

Symbol	ENSEMBL ID	Chr	Start	End	Strand	Length	logFC	(UP:1;DOWN:-1; NO CHANGE:0)
Nptx1	ENSMUSG00000025582	chr11	119400033	119409067	-	5217	16.71	1
Spp1	ENSMUSG00000029304	chr5	104864137	104870069	+	1648	16.26	1
Areg	ENSMUSG00000029378	chr5	91568641	91577458	+	1199	16.25	1
Acan	ENSMUSG00000030607	chr7	86198369	86259985	+	7355	15.86	1
Dhrs9	ENSMUSG00000027068	chr2	69218502	69242590	+	4281	15.62	1
Ltf	ENSMUSG00000032496	chr9	110921796	110945270	+	2742	15.06	1
Add2	ENSMUSG00000030000	chr6	86028078	86069549	+	3119	15.03	1
Gad2	ENSMUSG00000026787	chr2	22477725	22549394	+	5876	14.99	1
Aqp3	ENSMUSG00000028435	chr4	41039755	41045216	-	1829	14.95	1
Abcb1a	ENSMUSG00000040584	chr5	8660077	8748575	+	5480	14.95	1
Ereg	ENSMUSG00000029377	chr5	91503643	91522675	+	4136	14.73	1
Sema3a	ENSMUSG00000028883	chr5	13125593	13602565	+	10104	14.54	1
Tmprss11bnl	ENSMUSG00000035861	chr5	87086656	87105387	-	4171	14.5	1
Klhl1	ENSMUSG00000022076	chr14	96504478	96918321	-	4122	14.38	1
Nipal1	ENSMUSG00000067219	chr5	73039035	73062317	+	4205	14.09	1
Scn9a	ENSMUSG00000075316	chr2	66318137	66473009	-	10723	13.99	1
Il1rn	ENSMUSG00000026981	chr2	24192373	24207014	+	3207	13.98	1
Grem1	ENSMUSG00000074934	chr2	113586321	113598803	-	4148	13.96	1
Tmprss11e	ENSMUSG00000054537	chr5	87134211	87174840	-	3416	13.94	1
Slitrk6	ENSMUSG00000045871	chr14	111147800	111154371	-	4242	13.93	1
Prl2c3	ENSMUSG00000056457	chr13	12883089	12892345	-	850	13.88	1
Pgap1	ENSMUSG00000073678	chr1	54529844	54532810	-	2967	13.82	1
Padi4	ENSMUSG00000025330	chr4	140301780	140330151	-	4835	13.75	1
AA467197	ENSMUSG00000033213	chr2	122462722	122466927	+	1203	13.71	1
Qrfp	ENSMUSG00000043102	chr2	31661686	31666100	-	2927	13.63	1
Sacs	ENSMUSG00000048279	chr14	61757294	61859532	+	20434	13.54	1
Pthlh	ENSMUSG00000048776	chr6	147200623	147212689	-	1603	13.48	1
A330021E22Rik	ENSMUSG00000040473	chr5	5579278	5664239	-	5497	13.45	1
Foxg1	ENSMUSG00000020950	chr12	50483680	50487843	+	4164	13.42	1
Siglec15	ENSMUSG00000091055	chr18	78240353	78254134	-	1156	13.41	1
1700012B09Rik	ENSMUSG00000031927	chr9	14562644	14575474	-	679	13.4	1
Tnfrsf26	ENSMUSG00000045362	chr7	150793568	150813858	-	3107	13.37	1
Fer1l6	ENSMUSG00000037106	chr15	58341603	58595425	+	8342	13.35	1
Uba6	ENSMUSG00000035898	chr5	86539745	86601828	-	6726	13.22	1
Sfrp4	ENSMUSG00000021319	chr13	19714973	19724863	+	4049	13.19	1
Adamts4	ENSMUSG00000006403	chr1	173180552	173190768	+	4810	13.16	1

Cldn6	ENSMUSG00000023906	chr17	23816332	23819413	+	1506	13.14	1
Mmp10	ENSMUSG00000047562	chr9	7502352	7510238	+	1720	13.13	1
Samd5	ENSMUSG00000060487	chr10	9347057	9395006	-	2495	13.02	1
Hmga2	ENSMUSG00000056758	chr10	119798331	119913525	-	6772	13.01	1
Egr3	ENSMUSG00000033730	chr14	70477252	70479964	+	1469	12.99	1
Ndst3	ENSMUSG00000027977	chr3	123229084	123393770	-	6179	12.91	1
Ptgs2	ENSMUSG00000032487	chr1	151947254	151955140	+	4143	12.88	1
Has2	ENSMUSG00000022367	chr15	56497182	56526094	-	4237	12.87	1
Prrg4	ENSMUSG00000027171	chr2	104670898	104690033	-	3165	12.87	1
2310002 L13Rik	ENSMUSG00000024512	chr18	70400083	70404052	-	1041	12.86	1
Adam12	ENSMUSG00000054555	chr7	141074882	141423829	-	8273	12.84	1
E2f8	ENSMUSG00000046179	chr7	56121799	56136966	-	5447	12.79	1
Nav3	ENSMUSG00000020181	chr10	109119716	109437275	-	9304	12.78	1
Itpripl2	ENSMUSG00000073858	chr7	125633241	125634848	-	1608	12.76	1
Ntm	ENSMUSG00000059974	chr9	28802335	29770726	-	8079	12.75	1
Cthrc1	ENSMUSG00000054196	chr15	38908478	38918667	+	1480	12.69	1
Klra4	ENSMUSG00000079852	chr6	129993749	130017289	-	1264	12.66	1
Hmgcll1	ENSMUSG00000007908	chr9	75862662	75984157	+	6517	12.64	1
Mmp13	ENSMUSG00000050578	chr9	7272514	7283333	+	2675	12.64	1
4930503 L19Rik	ENSMUSG00000044906	chr18	70612794	70632134	-	2291	12.56	1
Ano4	ENSMUSG00000035189	chr10	88411740	88720535	-	5142	12.53	1
Otop1	ENSMUSG00000051596	chr5	38667211	38695448	+	3239	12.52	1
Adamts 12	ENSMUSG00000047497	chr15	10994545	11276622	+	6252	12.5	1
Hao1	ENSMUSG00000027261	chr2	134323097	134380104	-	2284	12.49	1
Rbpsuh-rs3	ENSMUSG00000079575	chr6	46479350	46480921	-	1464	12.43	1
Bnc1	ENSMUSG00000025105	chr7	89111558	89137185	-	4653	12.43	1
Krt27	ENSMUSG00000017588	chr11	99206879	99212418	-	1550	12.43	1
Atp2c2	ENSMUSG00000034112	chr8	122223909	122281618	+	3223	12.41	1
Dock11	ENSMUSG00000031093	chrX	33428827	33616557	+	9240	12.4	1
Ctse	ENSMUSG00000004552	chr1	133534883	133572080	+	3168	12.37	1
Prokr2	ENSMUSG00000050558	chr2	132163469	132211183	-	7919	12.33	1
Gpr137b	ENSMUSG00000021306	chr13	13449890	13485891	-	3223	12.29	1
Kif26b	ENSMUSG00000026494	chr1	180459256	180862986	+	7820	12.28	1
Tnfrsf9	ENSMUSG00000028965	chr4	150288671	150320211	+	2722	12.27	1
Nrk	ENSMUSG00000052854	chrX	135448969	135545071	+	9210	12.22	1
Gabre	ENSMUSG00000031340	chrX	69501338	69520142	-	4736	12.21	1
Gm1413 7	ENSMUSG00000055926	chr2	119000245	119003311	+	3067	12.18	1
Serpine 1	ENSMUSG00000037411	chr5	137537374	137548142	-	3024	12.17	1

4930503B20Rik	ENSMUSG00000090202	chr3	146308157	146314281	-	2404	12.16	1
Rgs17	ENSMUSG00000019775	chr10	4424141	4520878	+	8299	12.12	1
Alb	ENSMUSG00000029368	chr5	90889915	90905629	+	2043	12.11	1
Scg2	ENSMUSG00000050711	chr1	79431244	79436665	-	2485	12.1	1
Styk1	ENSMUSG00000032899	chr6	131249162	131303615	-	4355	12.08	1
Rad18	ENSMUSG00000030254	chr6	112569844	112646680	-	5796	12.03	1
Ak4	ENSMUSG00000028527	chr4	101091882	101140376	+	5425	11.97	1
Cenpf	ENSMUSG00000026605	chr1	191464478	191511965	-	11368	11.95	1
Scin	ENSMUSG00000002565	chr12	40786356	40860815	-	3011	11.94	1
Lrp8	ENSMUSG00000028613	chr4	107474866	107549445	+	10514	11.87	1
Bmper	ENSMUSG00000031963	chr9	23027520	23289646	+	3793	11.86	1
Gm11019	ENSMUSG00000078952	chr13	98204925	98266960	+	2724	11.85	1
Adamdec1	ENSMUSG00000022057	chr14	69181445	69200143	-	2425	11.83	1
Fbn2	ENSMUSG00000024598	chr18	58168277	58369580	-	10480	11.82	1
Zfp57	ENSMUSG00000036036	chr17	37138108	37147580	+	3786	11.77	1
Pde7a	ENSMUSG00000069094	chr3	19123108	19211322	-	7748	11.76	1
Cenpc1	ENSMUSG00000029253	chr5	86441068	86494608	-	3173	11.75	1
Kcnq5	ENSMUSG00000028033	chr1	21388484	21952023	-	7716	11.73	1
Fga	ENSMUSG00000028001	chr3	82830075	82838036	+	2933	11.72	1
Pak3	ENSMUSG00000031284	chrX	139953134	140232335	+	9547	11.7	1
Gm13387	ENSMUSG00000087028	chr2	24978123	25000916	-	2881	11.69	1
Tnfsf13b	ENSMUSG00000031497	chr8	10006843	10035441	+	977	11.68	1
Ccna2	ENSMUSG00000027715	chr3	36463788	36471072	-	3510	11.67	1
Prl3d2	ENSMUSG00000062737	chr13	27213548	27219415	+	866	11.66	1
Aspa	ENSMUSG00000020774	chr11	73118494	73140309	-	5621	11.6	1
Trem1	ENSMUSG00000042265	chr17	48371903	48386091	+	3024	11.6	1
Ripk3	ENSMUSG00000022221	chr14	56403833	56407694	-	1964	11.59	1
Pbp2	ENSMUSG00000047104	chr6	135259802	135260365	-	564	11.58	1
Sele	ENSMUSG00000026582	chr1	165978365	165987808	+	2912	11.57	1
Calcb	ENSMUSG00000030666	chr7	121862157	121866879	+	962	11.49	1
Clec7a	ENSMUSG00000079293	chr6	129411611	129422790	-	2270	11.47	1
Tll1	ENSMUSG00000053626	chr8	66493567	66684790	-	4928	11.4	1
Nppb	ENSMUSG00000029019	chr4	147359897	147361314	+	781	11.38	1
Unc13a	ENSMUSG00000034799	chr8	74148316	74195656	-	13135	11.38	1
3110039M20Rik	ENSMUSG00000089922	chr12	50486161	50508333	+	4715	11.28	1
Trpa1	ENSMUSG00000032769	chr1	14862729	14908943	-	4263	11.27	1
Gadd45a	ENSMUSG00000036390	chr6	66985090	66987451	-	1764	11.26	1
Kif11	ENSMUSG00000012443	chr19	37450893	37496349	+	4819	11.26	1
Hdac9	ENSMUSG00000004698	chr12	34732451	35213754	-	9609	11.23	1
Slitrk1	ENSMUSG00000075478	chr14	109309208	109313456	-	4249	11.23	1

Serpina 3k	ENSMUSG00000058207	chr12	105576696	105583951	+	1654	11.22	1
Gpr149	ENSMUSG00000043441	chr3	62332999	62409062	-	4288	11.22	1
Gm1260 6	ENSMUSG00000087659	chr4	88881603	88919307	-	1331	11.19	1
Frzb	ENSMUSG00000027004	chr2	80252127	80287782	-	3237	11.17	1
Pik3cg	ENSMUSG00000020573	chr12	32858338	32893524	-	6816	11.17	1
Nrcam	ENSMUSG00000020598	chr12	45429872	45702951	+	8295	11.16	1
Stx11	ENSMUSG00000039232	chr10	12659787	12684065	-	2396	11.16	1
Bub1	ENSMUSG00000027379	chr2	127626858	127657601	-	4354	11.16	1
Tnfrsf23	ENSMUSG00000037613	chr7	150851714	150871777	-	3353	11.16	1
Gpr171	ENSMUSG00000050075	chr3	58900370	58905743	-	2225	11.14	1
Gas2l3	ENSMUSG00000074802	chr10	88871568	88906712	-	6748	11.13	1
Fgb	ENSMUSG00000033831	chr3	82846169	82853725	-	1644	11.13	1
Gm9833	ENSMUSG00000049230	chr3	10088173	10092564	+	1766	11.13	1
Nlrp3	ENSMUSG00000032691	chr11	59355070	59380458	+	4057	11.12	1
Srpx2	ENSMUSG00000031253	chrX	130442965	130466985	+	2922	11.12	1
Sfmbt2	ENSMUSG00000061186	chr2	10292137	10516880	+	9128	11.1	1
Caprin2	ENSMUSG00000030309	chr6	148791014	148844759	-	7656	11.1	1
Gpr35	ENSMUSG00000026271	chr1	94869696	94882968	+	4498	11.09	1
Nav1	ENSMUSG00000090399	chr1	137503024	137584682	-	1645	11.09	1
Hhipl1	ENSMUSG00000021260	chr12	109544480	109566510	+	2795	11.08	1
Igsf11	ENSMUSG00000022790	chr16	38901584	39027272	+	3620	11.08	1
Dmbt1	ENSMUSG00000047517	chr7	138175590	138265140	+	6274	11.07	1
Tnnt2	ENSMUSG00000026414	chr1	137737452	137748837	+	1106	11.06	1
Rims2	ENSMUSG00000037386	chr15	39029878	39513486	+	5402	11.06	1
Lgr6	ENSMUSG00000042793	chr1	136882925	137001853	-	3804	11.05	1
C33002 7C09Rik	ENSMUSG00000033031	chr16	48994298	49019822	+	5688	11.04	1
Apold1	ENSMUSG00000090698	chr6	134932019	134936854	+	3284	11.03	1
Npl	ENSMUSG00000042684	chr1	155350146	155396844	-	1403	11.02	1
March11	ENSMUSG00000022269	chr15	26238803	26339331	+	2848	10.99	1
Foxc2	ENSMUSG00000046714	chr8	123640071	123642792	+	2722	10.98	1
Nrg1	ENSMUSG00000062991	chr8	32928500	33119581	-	3220	10.97	1
Mex3b	ENSMUSG00000057706	chr7	90015843	90020073	+	3403	10.94	1
Glp1r	ENSMUSG00000024027	chr17	31038785	31073455	+	1507	10.94	1
Gm22	ENSMUSG00000043903	chr8	124793469	124796546	+	3078	10.92	1
Nr1h5	ENSMUSG00000048938	chr3	102743581	102768056	-	2835	10.91	1
Prl2c2	ENSMUSG00000079092	chr13	13088393	13097659	-	850	10.91	1
Cd80	ENSMUSG00000075122	chr16	38459013	38495821	+	2921	10.9	1
Aspm	ENSMUSG00000033952	chr1	141351350	141390667	+	11491	10.89	1
Sh2d1b 1	ENSMUSG00000026673	chr1	172207507	172216900	+	2488	10.88	1
Hectd2	ENSMUSG00000041180	chr19	36629129	36695625	+	9066	10.86	1

5830416P10Rik	ENSMUSG00000074788	chr19	53515702	53539286	-	8664	10.85	1
Tbx18	ENSMUSG00000032419	chr9	87597635	87626095	-	4679	10.85	1
Abcb11	ENSMUSG00000027048	chr2	69076339	69180671	-	5046	10.84	1
Megf10	ENSMUSG00000024593	chr18	57292744	57457121	+	7718	10.84	1
Fgg	ENSMUSG00000033860	chr3	82811781	82818975	+	1964	10.84	1
Krt20	ENSMUSG00000035775	chr11	99289717	99299464	-	1927	10.83	1
Cck	ENSMUSG00000032532	chr9	121398943	121404807	-	848	10.83	1
Mmp1a	ENSMUSG00000043089	chr9	7464141	7476869	+	2022	10.81	1
Trim66	ENSMUSG00000031026	chr7	116592520	116651648	-	10125	10.81	1
4930486L24Rik	ENSMUSG00000050345	chr13	60943982	60965777	-	1364	10.8	1
Gm12603	ENSMUSG00000085183	chr4	88696212	88730518	+	761	10.8	1
4732471D19Rik	ENSMUSG00000043183	chr13	54605140	54652651	+	5690	10.79	1
Htr2a	ENSMUSG00000034997	chr14	75040647	75106666	+	2971	10.79	1
Tlr7	ENSMUSG00000044583	chrX	163742861	163768490	-	4082	10.77	1
Ocstamp	ENSMUSG00000027670	chr2	165219260	165225905	-	3657	10.75	1
Sgol2	ENSMUSG00000026039	chr1	58052815	58082743	+	4939	10.74	1
Csgalnact1	ENSMUSG00000036356	chr8	70880680	71259045	-	4774	10.73	1
Serpinb2	ENSMUSG00000062345	chr1	109408000	109432055	+	2349	10.73	1
Gp49a	ENSMUSG00000089672	chr10	51200438	51206122	+	2107	10.72	1
Clec4d	ENSMUSG00000030144	chr6	123212125	123225286	+	1336	10.7	1
Atp6v0d2	ENSMUSG00000028238	chr4	19803988	19849752	-	2716	10.7	1
Casc5	ENSMUSG00000027326	chr2	118872855	118931237	+	8542	10.7	1
2610109H07Rik	ENSMUSG00000029005	chr4	147472546	147504807	-	5200	10.68	1
Dlgap5	ENSMUSG00000037544	chr14	48007454	48038082	-	4714	10.66	1
Itih3	ENSMUSG00000006522	chr14	31721759	31736946	-	3637	10.65	1
Gdnf	ENSMUSG00000022144	chr15	7761011	7787575	+	3509	10.58	1
Ap3s1	ENSMUSG00000024480	chr18	46901571	46950480	+	1479	10.57	1
Kif20b	ENSMUSG00000024795	chr19	34996848	35050221	+	5567	10.57	1
Ngp	ENSMUSG00000032484	chr9	110322254	110325514	+	1217	10.57	1
Erc2	ENSMUSG00000040640	chr14	28435614	29291721	+	6419	10.56	1
Kntc1	ENSMUSG00000029414	chr5	124199735	124271602	+	6961	10.56	1
Tmem171	ENSMUSG00000052485	chr13	99456190	99464789	-	1583	10.56	1
Col28a1	ENSMUSG00000068794	chr6	7947808	8142617	-	4896	10.54	1
Fap	ENSMUSG00000000392	chr2	62339000	62412132	-	5662	10.54	1

4833407H14Rik	ENSMUSG00000090800	chr19	53535121	53537213	+	1485	10.53	1
Rbm41	ENSMUSG00000031433	chrX	136424050	136533134	-	6638	10.53	1
Nuf2	ENSMUSG00000026683	chr1	171428065	171461595	-	2562	10.51	1
Psg17	ENSMUSG00000004540	chr7	19399286	19406956	-	2100	10.5	1
A830080D01Rik	ENSMUSG00000044150	chrX	155964620	156031013	+	4869	10.49	1
BC055004	ENSMUSG00000047592	chr5	138667126	138694591	+	4482	10.48	1
A2m	ENSMUSG00000030111	chr6	121586191	121629255	+	4670	10.48	1
Mthfd2l	ENSMUSG00000029376	chr5	91360222	91450386	+	2235	10.45	1
Cep55	ENSMUSG00000024989	chr19	38129515	38148915	+	2778	10.44	1
Csf2	ENSMUSG00000018916	chr11	54060773	54063169	-	800	10.44	1
Lipo1	ENSMUSG00000024766	chr19	33592230	33836441	-	3515	10.43	1
Kcnk12	ENSMUSG00000050138	chr17	88145177	88197334	-	1936	10.41	1
Tifa	ENSMUSG00000046688	chr3	127492790	127535053	+	2527	10.41	1
Prelid2	ENSMUSG00000056671	chr18	42035350	42110848	-	724	10.39	1
Evi2b	ENSMUSG00000070354	chr11	79326887	79344061	-	3941	10.38	1
9330188P03Rik	ENSMUSG00000087212	chr14	105988844	105993124	+	982	10.38	1
Cdh6	ENSMUSG00000039385	chr15	12963955	13103394	-	2632	10.38	1
Snap25	ENSMUSG00000027273	chr2	136539189	136608164	+	6791	10.33	1
Kcnc2	ENSMUSG00000035681	chr10	111708179	111903360	+	6196	10.3	1
Vmn2r79	ENSMUSG00000090362	chr7	94144975	94186478	+	2556	10.29	1
Apoa4	ENSMUSG00000032080	chr9	46048779	46051542	+	1815	10.28	1
E2f7	ENSMUSG00000020185	chr10	110182495	110224440	+	9009	10.28	1
Orm1	ENSMUSG00000039196	chr4	63005594	63009197	+	775	10.28	1
Ptpn4	ENSMUSG00000026384	chr1	121554621	121734190	-	5744	10.26	1
Krt6a	ENSMUSG00000058354	chr15	101520363	101524738	-	2256	10.25	1
Abcg3	ENSMUSG00000029299	chr5	105364076	105411737	-	3550	10.24	1
Ankrd6	ENSMUSG00000040183	chr4	32891010	33037816	-	5477	10.24	1
Clec5a	ENSMUSG00000029915	chr6	40524893	40535820	-	3840	10.24	1
Tmem179	ENSMUSG00000054013	chr12	113738395	113749371	-	2373	10.23	1
Apoc2	ENSMUSG00000002992	chr7	20256928	20266772	-	932	10.22	1
Bard1	ENSMUSG00000026196	chr1	71074072	71149720	-	5659	10.21	1
Uox	ENSMUSG00000028186	chr3	146260041	146295269	+	3072	10.21	1
Spin4	ENSMUSG00000071722	chrX	92217846	92222021	-	4176	10.19	1
Itga2	ENSMUSG00000015533	chr13	115626124	115722249	-	4214	10.19	1
C6	ENSMUSG00000022181	chr15	4677175	4765456	+	4036	10.19	1
Trim15	ENSMUSG00000050747	chr17	36997638	37004155	-	1959	10.19	1
Cxcl13	ENSMUSG00000023078	chr5	96385945	96390086	+	1174	10.17	1
Alg13	ENSMUSG00000041718	chrX	140752355	140808993	+	5964	10.16	1
Lcn13	ENSMUSG00000062061	chr2	25555563	25558846	+	744	10.14	1
Rnf183	ENSMUSG00000063851	chr4	62088574	62096286	-	1525	10.12	1

Wdhd1	ENSMUSG00000037572	chr14	47860619	47896532	-	7192	10.12	1
A530054K11Rik	ENSMUSG00000021510	chr13	67717938	67738690	-	5179	10.09	1
Cd244	ENSMUSG00000004709	chr1	173489324	173515449	+	3859	10.09	1
9330182L06Rik	ENSMUSG00000056004	chr5	9266118	9481825	+	7918	10.08	1
Fam13a	ENSMUSG00000037709	chr6	58882084	58974543	-	6328	10.08	1
Glrp1	ENSMUSG00000062310	chr1	90397698	90406461	-	525	10.08	1
Cntnap4	ENSMUSG00000031772	chr8	115093943	115406617	+	6281	10.06	1
D6Mm5e	ENSMUSG00000030041	chr6	82896896	82980177	+	2086	10.06	1
Ercc6l	ENSMUSG00000051220	chrX	99337055	99352430	-	5302	10.05	1
4930547N16Rik	ENSMUSG00000035365	chr10	87554177	87609686	-	3594	10.04	1
AI606473	ENSMUSG00000093738	chr3	153993785	153997218	+	1731	10.04	1
Fam5c	ENSMUSG00000035131	chr1	148342759	148749602	+	6134	10.04	1
2810417H13Rik	ENSMUSG00000040204	chr9	65738044	65751073	+	4813	10.03	1
Grem2	ENSMUSG00000050069	chr1	176763916	176851950	-	3745	10.03	1
Rnf219	ENSMUSG00000022120	chr14	104876751	104921883	-	3456	10.03	1
Tmem74	ENSMUSG00000054409	chr15	43698241	43701575	-	1472	10.03	1
Gm14286	ENSMUSG00000087445	chr2	157370002	157371205	-	464	10.03	1
Kcnj15	ENSMUSG00000062609	chr16	95479165	95521867	+	6714	10.02	1
Klk6	ENSMUSG00000050063	chr7	51079869	51087397	+	1455	10.02	1
Tfpi2	ENSMUSG00000029664	chr6	3912594	3918375	-	1530	10.02	1
2610021A01Rik	ENSMUSG00000091474	chr7	48854631	48883903	+	4004	10.01	1
Alpk1	ENSMUSG00000028028	chr3	127373317	127483445	-	6709	9.996	1
Nos2	ENSMUSG00000020826	chr11	78734289	78773756	+	4192	9.996	1
Ccl7	ENSMUSG00000035373	chr11	81859214	81861027	+	801	9.987	1
Gm15726	ENSMUSG00000087263	chrX	166416979	166423164	-	509	9.987	1
Dlx1	ENSMUSG00000041911	chr2	71366170	71372038	+	5396	9.917	1
Cysltr1	ENSMUSG00000052821	chrX	103769685	103799018	-	5142	9.907	1
Rgs20	ENSMUSG00000002459	chr1	4899657	5060366	-	4239	9.896	1
Kng1	ENSMUSG00000022875	chr16	23057938	23082141	+	3159	9.886	1
Tes3-ps	ENSMUSG00000074910	chr13	49588687	49591945	+	3259	9.886	1
Zscan12	ENSMUSG00000036721	chr13	21454696	21461693	+	2228	9.875	1
Sprr2f	ENSMUSG00000050635	chr3	92169109	92170364	+	609	9.865	1

Scgb3a1	ENSMUSG00000064057	chr11	49477112	49478620	+	598	9.854	1
Fignl1	ENSMUSG00000035455	chr11	11687434	11708965	-	4839	9.843	1
Slc14a1	ENSMUSG00000059336	chr18	78296830	78338858	-	3995	9.842	1
Slfn8	ENSMUSG00000035208	chr11	82815660	82834312	-	4091	9.833	1
Tmem200a	ENSMUSG00000049420	chr10	25712700	25714175	-	1476	9.833	1
Tlr1	ENSMUSG00000044827	chr5	65316050	65324000	-	2652	9.822	1
Ibsp	ENSMUSG00000029306	chr5	104728190	104740488	+	2068	9.811	1
Tnfrsf22	ENSMUSG00000010751	chr7	150820713	150835566	-	4287	9.811	1
Mgat5	ENSMUSG00000036155	chr1	129101563	129380281	+	3836	9.808	1
4930467D21Rik	ENSMUSG00000085273	chr5	97821459	98017144	+	2971	9.789	1
Klra2	ENSMUSG00000030187	chr6	131169241	131197380	-	1898	9.789	1
Skp2	ENSMUSG00000054115	chr15	9041742	9070207	-	6196	9.789	1
Casp12	ENSMUSG00000025887	chr9	5345430	5373032	+	3840	9.777	1
Gpr50	ENSMUSG00000056380	chrX	68916842	68922432	+	3069	9.777	1
Nanos1	ENSMUSG00000072437	chr19	60831890	60835816	+	3927	9.777	1
Stil	ENSMUSG00000028718	chr4	114672723	114715801	+	5836	9.777	1
Htr1b	ENSMUSG00000049511	chr9	81524950	81526222	-	1273	9.766	1
Uchl4	ENSMUSG00000035337	chr9	64083008	64084169	+	1162	9.755	1
BC055324	ENSMUSG00000041406	chr1	165876124	165924927	-	4156	9.743	1
Khdrbs2	ENSMUSG00000026058	chr1	32229651	32714583	+	1359	9.743	1
Tnfsf11	ENSMUSG00000022015	chr14	78677252	78707850	-	2236	9.743	1
Ms4a4d	ENSMUSG00000024678	chr19	11611291	11632957	+	1513	9.72	1
Ccdc14	ENSMUSG00000022833	chr16	34690702	34725280	+	4107	9.708	1
Gm6634	ENSMUSG00000086538	chr3	70576301	70611213	-	1804	9.708	1
Jag1	ENSMUSG00000027276	chr2	136907192	136942380	-	5827	9.699	1
Esm1	ENSMUSG00000042379	chr13	113999867	114008306	+	2097	9.696	1
Lrrc15	ENSMUSG00000052316	chr16	30269388	30283342	-	5310	9.696	1
Ccl22	ENSMUSG00000031779	chr8	97269490	97275599	+	2325	9.684	1
Cd177	ENSMUSG00000052212	chr7	25529004	25545330	-	2712	9.684	1
Foxa2	ENSMUSG00000037025	chr2	147868613	147872705	-	2370	9.66	1
Pyhin1	ENSMUSG00000043263	chr1	175560990	175578059	+	3497	9.66	1
Vash2	ENSMUSG00000037568	chr1	192771527	192803175	-	4188	9.66	1
Cfi	ENSMUSG00000058952	chr3	129539655	129578246	+	2075	9.648	1
Gm11714	ENSMUSG00000087113	chr11	107289590	107297000	+	876	9.648	1
Tlr6	ENSMUSG00000051498	chr5	65344345	65351287	-	2603	9.648	1
Rassf6	ENSMUSG00000029370	chr5	91032102	91069513	-	1958	9.636	1
Ttk	ENSMUSG00000038379	chr9	83728296	83765997	+	2904	9.636	1
Selp	ENSMUSG00000026580	chr1	166045395	166080157	+	4413	9.624	1
Ndc80	ENSMUSG00000024056	chr17	71845440	71876197	-	2201	9.623	1
Ccl17	ENSMUSG00000031780	chr8	97334353	97335935	+	513	9.611	1

Cxcr2	ENSMUSG00000026180	chr1	74200563	74207812	+	3040	9.611	1
Dlx2	ENSMUSG00000023391	chr2	71381466	71384811	-	2854	9.598	1
Lrrc55	ENSMUSG00000075224	chr2	85002491	85036856	-	4080	9.585	1
Mnda	ENSMUSG00000026536	chr1	175826486	175843053	-	1651	9.585	1
2010300F17Rik	ENSMUSG00000091444	chr11	96574296	96608750	-	2832	9.572	1
Ccdc99	ENSMUSG00000069910	chr11	34622692	34647143	-	3460	9.572	1
Clec4a3	ENSMUSG00000043832	chr6	122902533	122919893	+	1327	9.572	1
Vsig4	ENSMUSG00000044206	chrX	93442542	93488777	-	1484	9.572	1
Sox2	ENSMUSG00000074637	chr3	34548927	34551383	+	2457	9.558	1
1810011H11Rik	ENSMUSG00000041707	chr14	33599149	33631154	+	1473	9.546	1
Neto2	ENSMUSG00000036902	chr8	88161136	88224653	-	5096	9.546	1
Rel	ENSMUSG00000020275	chr11	23641514	23670970	-	2799	9.546	1
B230216G23Rik	ENSMUSG00000071112	chr6	142361961	142368250	+	1767	9.519	1
Dtl	ENSMUSG00000037474	chr1	193361244	193399413	-	4366	9.519	1
Foxf1a	ENSMUSG00000042812	chr8	123608286	123610851	+	1222	9.519	1
Gbp3	ENSMUSG00000028268	chr3	142223016	142236173	+	3239	9.519	1
Mia2	ENSMUSG00000035349	chr12	60196786	60210117	+	1629	9.519	1
Sell	ENSMUSG00000026581	chr1	165992207	166010916	+	2376	9.519	1
Has3	ENSMUSG00000031910	chr8	109394142	109408466	+	6380	9.518	1
Kng2	ENSMUSG00000060459	chr16	22985927	23029174	-	2509	9.506	1
Syt6	ENSMUSG00000027849	chr3	103379154	103449490	+	5765	9.506	1
Fbxo5	ENSMUSG00000019773	chr10	4540941	4547381	+	2003	9.492	1
Naip5	ENSMUSG00000071203	chr13	100981694	101016278	-	5204	9.492	1
Caln1	ENSMUSG00000060371	chr5	130845346	131316515	+	3565	9.478	1
Cenpi	ENSMUSG00000031262	chrX	130842623	130897178	+	5681	9.478	1
Prkg2	ENSMUSG00000029334	chr5	99358792	99466370	-	5369	9.476	1
Rnf125	ENSMUSG00000033107	chr18	21103126	21142349	+	1498	9.45	1
Tmem132b	ENSMUSG00000029483	chr5	126259004	126268438	+	1809	9.45	1
Ifi205	ENSMUSG00000054203	chr1	175942129	175961886	-	1613	9.436	1
Ckap2	ENSMUSG00000037725	chr8	23278624	23296291	-	2601	9.422	1
Hhex	ENSMUSG00000024986	chr19	37507377	37515218	+	3838	9.422	1
4930583H14Rik	ENSMUSG00000037161	chr3	51192337	51200660	-	2064	9.407	1
Chrna1	ENSMUSG00000027107	chr2	73401272	73418395	-	4386	9.407	1
Prl2c5	ENSMUSG00000055360	chr13	13274982	13284190	+	1177	9.407	1
Samsn1	ENSMUSG00000022876	chr16	75859038	76022515	-	4618	9.407	1
Sytl5	ENSMUSG00000054453	chrX	9462748	9571669	+	2301	9.407	1
Il6	ENSMUSG00000025746	chr5	30339701	30346508	+	1087	9.393	1
Pilrb2	ENSMUSG00000066682	chr5	138307057	138312986	-	1830	9.378	1

Csn3	ENSMUSG00000001622	chr5	88354604	88361690	+	4152	9.333	1
Ambp	ENSMUSG00000028356	chr4	62804309	62815833	-	1409	9.317	1
Clec4a2	ENSMUSG00000030148	chr6	123056446	123094017	+	2969	9.317	1
Shcbp1	ENSMUSG00000022322	chr8	4735980	4779534	-	2132	9.302	1
4930452 B06Rik	ENSMUSG00000021747	chr14	9263706	9498754	-	3279	9.286	1
1110002 L01Rik	ENSMUSG00000071456	chr12	3405308	3426644	-	1829	9.27	1
Mrap	ENSMUSG00000039956	chr16	90738569	90750030	+	1464	9.254	1
Srp54c	ENSMUSG00000073079	chr12	56331155	56377912	+	2906	9.254	1
Gc	ENSMUSG00000035540	chr5	89846549	89886790	-	1666	9.221	1
H2-M9	ENSMUSG00000067201	chr17	36776230	36779611	-	1443	9.221	1
Saa1	ENSMUSG00000074115	chr7	53995871	53998350	-	605	9.221	1
Akr1c18	ENSMUSG00000021214	chr13	4131861	4149900	-	1207	9.205	1
Rbm12b	ENSMUSG00000046667	chr4	12067411	12073878	+	3122	9.205	1
Zfp189	ENSMUSG00000039634	chr4	49534048	49544389	+	3006	9.205	1
Galnt12	ENSMUSG00000039774	chr4	47104781	47135942	+	2288	9.188	1
Glmn	ENSMUSG00000029276	chr5	107977986	108026907	-	2688	9.188	1
Tceanc	ENSMUSG00000051224	chrX	162937744	162956499	-	4207	9.188	1
Troap	ENSMUSG00000032783	chr15	98905006	98913783	+	2582	9.188	1
Gabra3	ENSMUSG00000031343	chrX	69678020	69902187	-	3924	9.171	1
Pdzrn4	ENSMUSG00000036218	chr15	92227346	92602250	+	4191	9.171	1
Slc16a1 4	ENSMUSG00000026220	chr1	84902473	84931700	-	3198	9.171	1
Vmn2r7 8	ENSMUSG00000091962	chr7	94063810	94103687	+	2611	9.171	1
Bbs5	ENSMUSG00000063145	chr2	69485228	69505628	+	2445	9.153	1
Fpr2	ENSMUSG00000052270	chr17	18024788	18030916	+	1296	9.153	1
Tarm1	ENSMUSG00000053338	chr7	3489960	3503346	-	867	9.153	1
Trbc1	ENSMUSG00000076490	chr6	41488217	41489346	+	512	9.153	1
Accn3	ENSMUSG00000038276	chr5	23919269	23923652	+	1942	9.136	1
Gzmf	ENSMUSG00000015441	chr14	56824103	56830244	-	906	9.136	1
Pck1	ENSMUSG00000027513	chr2	172978549	172984774	+	3429	9.136	1
Slfn3	ENSMUSG00000018986	chr11	83004832	83028656	+	1816	9.136	1
Ctla2b	ENSMUSG00000074874	chr13	60996712	60998808	-	1147	9.123	1
Gbp5	ENSMUSG00000040264	chr3	142159864	142185308	+	4028	9.118	1
Ttr	ENSMUSG00000061808	chr18	20823751	20832825	+	1219	9.118	1
Tigit	ENSMUSG00000071552	chr16	43648974	43664297	-	963	9.1	1
Cxcl3	ENSMUSG00000029379	chr5	91215129	91217112	+	1009	9.082	1
Gab3	ENSMUSG00000032750	chrX	72212182	72330797	-	4005	9.082	1
Gpr65	ENSMUSG00000021886	chr12	99506845	99514834	+	2049	9.082	1
Ivl	ENSMUSG00000049128	chr3	92374834	92377637	-	1892	9.082	1
Tnfaip6	ENSMUSG00000053475	chr2	51893529	51912206	+	1710	9.082	1
Syt17	ENSMUSG00000058420	chr7	125525370	125587066	-	1617	9.064	1

Snx16	ENSMUSG00000027534	chr3	10417819	10440124	-	2652	9.027	1
Bpifb1	ENSMUSG00000027485	chr2	154016554	154046105	+	3617	9.007	1
Casp1	ENSMUSG00000025888	chr9	5298517	5307265	+	1497	9.007	1
Clca5	ENSMUSG00000036960	chr3	144733229	144762068	-	3984	9.007	1
Cwh43	ENSMUSG00000029154	chr5	73797315	73844674	+	2994	9.007	1
BC100451	ENSMUSG00000076433	chr11	118193674	118203814	-	1944	8.988	1
Clca1	ENSMUSG00000056025	chr3	144392641	144423941	-	3899	8.988	1
Has1	ENSMUSG00000003665	chr17	17980292	17992152	-	2090	8.988	1
Prl6a1	ENSMUSG00000069259	chr13	27404496	27411121	+	1159	8.988	1
Zfp955b	ENSMUSG00000061532	chr17	33426594	33440141	+	1638	8.988	1
Gm12602	ENSMUSG00000085569	chr4	88746568	88774130	-	1118	8.969	1
Sprr2h	ENSMUSG00000046259	chr3	92189607	92191246	+	752	8.969	1
Fabp4	ENSMUSG00000062515	chr3	10204347	10208576	-	637	8.952	1
Gm1966	ENSMUSG00000073902	chr7	113744757	113747549	-	2793	8.949	1
Gm2788	ENSMUSG00000085995	chr7	56133328	56141978	+	1662	8.949	1
Pdcd1lg2	ENSMUSG00000016498	chr19	29485409	29545647	+	1645	8.949	1
Gzmd	ENSMUSG00000059256	chr14	56748393	56751445	-	964	8.929	1
Trpc1	ENSMUSG00000032839	chr9	95607046	95650777	-	3014	8.908	1
Prss22	ENSMUSG00000045027	chr17	24130502	24135067	-	1323	8.901	1
Gm13704	ENSMUSG00000086544	chr2	73434583	73454069	+	3049	8.887	1
Gm5152	ENSMUSG00000069355	chr5	10242041	10248928	-	929	8.887	1
Stxbp3b	ENSMUSG00000071640	chr19	9632096	9633738	-	1007	8.887	1
Zfp449	ENSMUSG00000073176	chrX	53599577	53618851	+	3738	8.887	1
Creb3l3	ENSMUSG00000035041	chr10	80547069	80561619	-	3016	8.866	1
F630028O10Rik	ENSMUSG00000078122	chrX	93435281	93438975	+	3279	8.866	1
Gm17419	ENSMUSG00000091520	chr13	29034917	29043540	+	2649	8.866	1
Mcpt1	ENSMUSG00000022227	chr14	56636801	56639228	+	1028	8.866	1
Gm12648	ENSMUSG00000085931	chr4	93756267	94092279	-	656	8.845	1
Ifitm6	ENSMUSG00000059108	chr7	148201711	148202791	-	541	8.845	1
Jph1	ENSMUSG00000042686	chr1	16987545	17087879	-	2192	8.845	1
Gm16701	ENSMUSG00000086622	chr1	168904155	168930972	-	2286	8.823	1
C430049B03Rik	ENSMUSG00000087365	chrX	50408223	50410367	-	1610	8.801	1
Gm14002	ENSMUSG00000086416	chr2	125033265	125043248	-	2839	8.801	1
Scel	ENSMUSG00000022123	chr14	103912558	104012563	+	3113	8.779	1

4930502E18Rik	ENSMUSG00000055555	chrX	51028003	51041618	+	1427	8.756	1
5730507C01Rik	ENSMUSG00000073197	chr12	18521544	18540997	+	1179	8.756	1
Lce1g	ENSMUSG00000027919	chr3	92554070	92556260	-	925	8.756	1
Prss35	ENSMUSG00000033491	chr9	86636434	86650680	+	2525	8.756	1
Serp2	ENSMUSG00000052584	chr14	76932621	76956665	-	1006	8.756	1
Sirpb1c	ENSMUSG00000074677	chr3	15695145	15748528	-	2847	8.733	1
Zfp780b	ENSMUSG00000063047	chr7	28747727	28759804	-	2421	8.733	1
Ncr1	ENSMUSG00000062524	chr7	4289326	4296766	+	1540	8.71	1
Zfp105	ENSMUSG00000057895	chr9	122832190	122840146	+	2130	8.71	1
1100001G20Rik	ENSMUSG00000051748	chr11	83560442	83566144	+	434	8.686	1
Ak5	ENSMUSG00000039058	chr3	152125779	152331104	-	3103	8.686	1
Expi	ENSMUSG00000000983	chr11	83522517	83524850	+	1220	8.686	1
Fam54a	ENSMUSG00000019992	chr10	20067625	20081475	+	2456	8.686	1
Gzmg	ENSMUSG00000040284	chr14	56775419	56778416	-	909	8.686	1
Zfp125	ENSMUSG00000069755	chr12	20905683	20906728	-	543	8.686	1
Ndp	ENSMUSG00000040138	chrX	16462647	16488900	-	1939	8.662	1
Plg	ENSMUSG00000059481	chr17	12571475	12612250	+	2755	8.662	1
Cts8	ENSMUSG00000057446	chr13	61348108	61356709	-	2493	8.637	1
Agk	ENSMUSG00000029916	chr6	40275477	40346761	+	2225	8.612	1
Gimap5	ENSMUSG00000043505	chr6	48696196	48704209	+	2353	8.612	1
Sh2d5	ENSMUSG00000045349	chr4	137806318	137817247	+	3740	8.598	1
Fam159a	ENSMUSG00000059816	chr4	108040382	108055954	-	593	8.587	1
Kpna2	ENSMUSG00000018362	chr11	106849943	106860855	-	2873	8.587	1
Gbp8	ENSMUSG00000034438	chr5	105443172	105482580	-	2603	8.561	1
Hgf	ENSMUSG00000028864	chr5	16059368	16125257	+	2755	8.561	1
Ank	ENSMUSG00000022265	chr15	27396432	27524664	+	3608	8.545	1
Cep57l1	ENSMUSG00000019813	chr10	41438652	41529676	-	2296	8.535	1
D730048I06Rik	ENSMUSG00000032108	chr9	35585618	35597697	-	1411	8.535	1
Gm2115	ENSMUSG00000085128	chr7	91677485	91726847	+	1840	8.535	1
Gm807	ENSMUSG00000074744	chr13	99870661	99877535	+	1723	8.535	1
Htr2b	ENSMUSG00000026228	chr1	87995601	88008545	-	2131	8.535	1
Epha4	ENSMUSG00000026235	chr1	77363760	77511663	-	6328	8.523	1
Gm6377	ENSMUSG00000048621	chrX	106392095	106395778	+	2795	8.508	1
Zfp382	ENSMUSG00000074220	chr7	30906961	30919684	+	2013	8.508	1
Nmnat2	ENSMUSG00000042751	chr1	154802231	154966390	+	4522	8.486	1
Fam89a	ENSMUSG00000043068	chr8	127264157	127275709	-	1315	8.48	1
H2-M1	ENSMUSG00000037334	chr17	36806953	36809164	-	1057	8.48	1

Mal2	ENSMUSG00000024479	chr15	54402921	54434401	+	2775	8.48	1
Havcr2	ENSMUSG00000020399	chr11	46268437	46294757	+	2721	8.465	1
Ptpn22	ENSMUSG00000027843	chr3	103663718	103716170	+	3064	8.461	1
Pgap1	ENSMUSG00000070889	chr1	54537550	54614526	-	2871	8.457	1
Adipoq	ENSMUSG00000022878	chr16	23146609	23158101	+	1381	8.452	1
Foxl2	ENSMUSG00000050397	chr9	98856026	98858545	+	2520	8.452	1
Ptx3	ENSMUSG00000027832	chr3	66023809	66029728	+	1922	8.452	1
Rgs5	ENSMUSG00000026678	chr1	171585632	171625944	+	4549	8.424	1
Ifit1	ENSMUSG00000034459	chr19	34715361	34724499	+	2656	8.424	1
Efcab7	ENSMUSG00000073791	chr4	99495889	99585393	+	2134	8.395	1
Gm5434	ENSMUSG00000059301	chr12	36816966	36818416	+	1451	8.395	1
Hoxc10	ENSMUSG00000022484	chr15	102797227	102802324	+	1909	8.395	1
Hoxc6	ENSMUSG00000001661	chr15	102828688	102842312	+	2502	8.395	1
Mtcp1	ENSMUSG00000090110	chrX	72650185	72661923	-	1310	8.395	1
Tat	ENSMUSG00000001670	chr8	112514337	112523703	+	2405	8.395	1
Bcl2a1a	ENSMUSG00000074147	chr9	88851738	88857254	+	816	8.335	1
Gm15247	ENSMUSG00000086695	chrX	166423733	166424871	-	403	8.335	1
Il1a	ENSMUSG00000027399	chr2	129125346	129135708	-	1974	8.335	1
Klra6	ENSMUSG00000061769	chr6	129963051	129976972	-	1116	8.335	1
Serpina1b	ENSMUSG00000071178	chr12	104966366	104976368	-	1394	8.335	1
Padi2	ENSMUSG00000028927	chr4	140462259	140508501	+	5013	8.33	1
Ccdc138	ENSMUSG00000038010	chr10	57960718	58038990	+	2431	8.304	1
Fxyd4	ENSMUSG00000004988	chr6	117883747	117887353	-	1199	8.304	1
Hrg	ENSMUSG00000022877	chr16	22951145	22961729	+	1734	8.304	1
Igfbp1	ENSMUSG00000020429	chr11	7097785	7102549	+	1525	8.304	1
Serpinc1	ENSMUSG00000026715	chr1	162908737	162933141	+	2157	8.304	1
Enc1	ENSMUSG00000041773	chr13	98011060	98022994	+	4742	8.276	1
Scn5a	ENSMUSG00000032511	chr9	119392528	119488134	-	8762	8.275	1
Ap3s1-ps2	ENSMUSG00000074154	chr8	96929008	96930286	+	1279	8.272	1
Gm5150	ENSMUSG00000078780	chr3	15848070	15906332	-	913	8.272	1
Rpp25	ENSMUSG00000062309	chr9	57351909	57353254	+	1346	8.272	1
Acsbg1	ENSMUSG00000032281	chr9	54452684	54509677	-	3604	8.266	1
Mmp3	ENSMUSG00000043613	chr9	7445822	7455972	+	1798	8.254	1
Prl8a9	ENSMUSG00000006490	chr13	27649878	27656473	-	926	8.24	1
Tmem132b	ENSMUSG00000070498	chr5	126102858	126179143	+	1222	8.24	1
Anxa10	ENSMUSG00000031635	chr8	64535839	64601915	-	1785	8.207	1
Calca	ENSMUSG00000030669	chr7	121774994	121779871	-	1480	8.207	1
Gm5127	ENSMUSG00000073010	chrX	103778525	103905896	+	2150	8.207	1

Gm19357	ENSMUSG00000093629	chr12	86427324	86429178	+		1133	8.173		1
Noxa1	ENSMUSG00000036805	chr2	24941187	24950669	-		1617	8.173		1
Zfp607	ENSMUSG00000020420	chr7	28647576	28664586	+		2061	8.173		1
Inhba	ENSMUSG00000041324	chr13	16103684	16119044	+		1980	8.166		1
Ear11	ENSMUSG00000047222	chr14	51874941	51875787	-		686	8.139		1
Npy	ENSMUSG00000029819	chr6	49772709	49779506	+		566	8.134		1
Has2as	ENSMUSG00000086541	chr15	56521499	56609954	+		1628	8.103		1
Hoxa4	ENSMUSG00000000942	chr6	52139670	52141752	-		1594	8.103		1
Prtn3	ENSMUSG00000057729	chr10	79337221	79345919	+		1732	8.103		1
Tmem202	ENSMUSG00000049526	chr9	59366493	59373308	-		1255	8.067		1
Apol9b	ENSMUSG00000057346	chr15	77559502	77566811	+		1594	8.033		1
Gzme	ENSMUSG00000022156	chr14	56736456	56739468	-		944	8.029		1
Orm2	ENSMUSG00000061540	chr4	63023483	63026912	+		775	8.029		1
Atg9b	ENSMUSG00000038295	chr5	23889999	23897961	-		3946	8.006		1
Zfp950	ENSMUSG00000074732	chr19	61192870	61195084	-		1390	7.991		1
Cdh23	ENSMUSG00000012819	chr10	59765496	60159238	-		16939	7.975		1
Cd209e	ENSMUSG00000040197	chr8	3847965	3854309	-		1802	7.952		1
Prl3d3	ENSMUSG00000062201	chr13	27248659	27254459	+		853	7.952		1
Btbd11	ENSMUSG00000020042	chr10	84849559	85123037	+		10033	7.933		1
Cdh17	ENSMUSG00000028217	chr4	11685294	11745042	+		4474	7.921		1
Gm527	ENSMUSG00000047227	chr12	66018898	66025578	+		1463	7.911		1
Gm6984	ENSMUSG00000057886	chr14	74353729	74355393	-		1665	7.911		1
Igkv12-46	ENSMUSG00000076564	chr6	69714517	69714986	-		347	7.911		1
Akr1c12	ENSMUSG00000021211	chr13	4267418	4278679	-		1265	7.885		1
Tlr13	ENSMUSG00000033777	chrX	103338543	103355832	+		4086	7.878		1
8430423G03Rik	ENSMUSG00000079069	chr5	149761826	149763234	+		639	7.869		1
Cxcl1	ENSMUSG00000029380	chr5	91320267	91322141	+		953	7.869		1
Dus4l	ENSMUSG00000020648	chr12	32324920	32339691	-		1768	7.869		1
Gm17024	ENSMUSG00000090582	chr12	40936074	40937660	-		634	7.869		1
2610203C20Rik	ENSMUSG00000074415	chr9	41388824	41400910	+		3268	7.863		1
Ggct	ENSMUSG00000002797	chr6	54932574	54942944	-		4437	7.856		1
B430203G13Rik	ENSMUSG00000067356	chr12	17931100	17932474	+		1375	7.827		1
Ect2	ENSMUSG00000027699	chr3	26996144	27052800	-		5202	7.819		1
Cd44	ENSMUSG00000005087	chr2	102651298	102741822	-		6851	7.814		1
4632433K11Rik	ENSMUSG00000063757	chr7	4985720	4986848	+		1129	7.782		1
Csf3	ENSMUSG00000038067	chr11	98562577	98564943	+		1413	7.782		1

Cxcl17	ENSMUSG00000060188	chr7	26185072	26197905	-	761	7.782	1
Gm6104	ENSMUSG00000062588	chr1	4869283	4870750	-	1468	7.782	1
Igkv10-96	ENSMUSG00000076527	chr6	68581957	68582424	-	347	7.782	1
Adam8	ENSMUSG00000025473	chr7	147164831	147178461	-	4638	7.776	1
Cd53	ENSMUSG00000040747	chr3	106561779	106593067	-	2802	7.758	1
Calcrl	ENSMUSG00000059588	chr2	84170783	84265568	-	4882	7.742	1
Apoh	ENSMUSG00000000049	chr11	108204668	108275710	+	1594	7.737	1
Ccl3	ENSMUSG00000000982	chr11	83461346	83462857	-	766	7.737	1
Serpina1e	ENSMUSG00000072849	chr12	105185141	105197185	-	1617	7.737	1
Kcnj12	ENSMUSG00000042529	chr11	60836066	60884633	+	2750	7.733	1
Crybb1	ENSMUSG00000029343	chr5	112684835	112698605	+	997	7.69	1
Gm10354	ENSMUSG00000072188	chr5	14974113	14978935	-	1215	7.69	1
Cd300lf	ENSMUSG00000047798	chr11	114977528	114995306	-	5149	7.682	1
Pde3b	ENSMUSG00000030671	chr7	121558795	121682765	+	6899	7.659	1
Ccbe1	ENSMUSG00000046318	chr18	66204956	66462393	-	9511	7.636	1
Ptprn	ENSMUSG00000026204	chr1	75243616	75260783	-	3535	7.617	1
Dusp4	ENSMUSG00000031530	chr8	35870351	35882948	+	2740	7.606	1
Prss37	ENSMUSG00000029909	chr6	40464823	40469507	-	1123	7.591	1
Btc	ENSMUSG00000082361	chr5	91786287	91831939	-	2795	7.585	1
Pqlc3	ENSMUSG00000045679	chr12	16995454	17007214	-	5408	7.558	1
Nrn1	ENSMUSG00000039114	chr13	36817494	36826346	-	1766	7.551	1
Fam78b	ENSMUSG00000060568	chr1	168931548	169021433	+	4986	7.543	1
Gm13816	ENSMUSG00000085976	chr2	92582387	92599365	+	715	7.538	1
Ap1s2	ENSMUSG00000031367	chrX	160346949	160371598	+	4965	7.527	1
Hs3st1	ENSMUSG00000051022	chr5	40005174	40146714	-	2230	7.494	1
Gm16194	ENSMUSG00000084956	chr17	29251090	29270756	+	733	7.484	1
Ighg1	ENSMUSG00000076614	chr12	114567187	114568734	-	974	7.484	1
Fosl1	ENSMUSG00000024912	chr19	5447703	5455945	+	1707	7.48	1
Odf2l	ENSMUSG00000028256	chr3	144781553	144816879	+	3237	7.451	1
Acsl6	ENSMUSG00000020333	chr11	54117300	54178258	+	9914	7.434	1
Ednrb	ENSMUSG00000022122	chr14	104213842	104243390	-	4037	7.433	1
2610002D18Rik	ENSMUSG00000078521	chr4	134066914	134079842	+	1275	7.428	1
Gm14221	ENSMUSG00000078956	chr2	160394115	160445707	-	340	7.428	1
Rbp2	ENSMUSG00000032454	chr9	98390956	98410190	+	672	7.428	1
Runx2	ENSMUSG00000039153	chr17	44632936	44951746	-	8837	7.422	1
Igf2bp2	ENSMUSG00000033581	chr16	22059082	22163372	-	7131	7.418	1
Dusp9	ENSMUSG00000031383	chrX	70884758	70888853	+	2722	7.398	1
Antxr2	ENSMUSG00000029338	chr5	98313707	98459981	-	3751	7.388	1
Inpp4b	ENSMUSG00000037940	chr8	83866455	84648935	+	10176	7.381	1

Thbs1	ENSMUSG00000040152	chr2	117937612	117952869	+	6073	7.377	1
Thbs2	ENSMUSG00000023885	chr17	14802507	14831269	-	5927	7.374	1
Gm17549	ENSMUSG00000091543	chr17	32421732	32435634	+	1209	7.37	1
Igkv1-135	ENSMUSG00000076502	chr6	67559739	67560502	+	362	7.37	1
Trem3	ENSMUSG00000041754	chr17	48386944	48398207	+	994	7.37	1
Aim2	ENSMUSG00000037860	chr1	175281010	175396171	+	3505	7.357	1
Cyp2e1	ENSMUSG00000025479	chr7	147949638	147960874	+	1827	7.351	1
Igf2bp1	ENSMUSG00000013415	chr11	95818477	95867254	-	9682	7.33	1
4930435H24Rik	ENSMUSG00000029092	chr5	45546250	45560803	-	408	7.309	1
Gm5483	ENSMUSG00000079597	chr16	36184298	36188197	+	414	7.309	1
Sema6d	ENSMUSG00000027200	chr2	123915705	124493506	+	10559	7.307	1
Dcbld2	ENSMUSG00000035107	chr16	58408556	58469840	+	8401	7.224	1
Kctd12b	ENSMUSG00000041633	chrX	150119697	150130934	-	4906	7.214	1
Chst11	ENSMUSG00000034612	chr10	82448243	82658645	+	5527	7.2	1
Col5a2	ENSMUSG00000026042	chr1	45431166	45560127	-	9591	7.192	1
Clcf1	ENSMUSG00000040663	chr19	4214238	4223490	+	3713	7.179	1
Ang5	ENSMUSG00000053961	chr14	44534734	44540745	+	708	7.178	1
Gm10033	ENSMUSG00000057924	chr8	71896044	71897282	-	654	7.178	1
Gm15050	ENSMUSG00000087626	chr6	52122880	52124058	+	552	7.178	1
Gm5551	ENSMUSG00000091052	chr5	28186989	28187606	+	618	7.178	1
Gm6712	ENSMUSG00000072761	chr17	17452246	17456475	+	474	7.178	1
Nppc	ENSMUSG00000026241	chr1	88562866	88567147	-	1043	7.178	1
Myof	ENSMUSG00000048612	chr19	37973526	38118067	-	7146	7.158	1
H19	ENSMUSG00000000031	chr7	149761434	149764048	-	2372	7.156	1
Gcnt4	ENSMUSG00000091387	chr13	97694644	97720867	+	5093	7.13	1
Brca2	ENSMUSG00000041147	chr5	151325205	151372321	+	10724	7.123	1
Twist1	ENSMUSG00000035799	chr12	34642536	34644682	+	1616	7.113	1
Gm6685	ENSMUSG00000032889	chr11	28239128	28239814	-	687	7.108	1
Disp2	ENSMUSG00000040035	chr2	118605455	118637029	+	9556	7.1	1
Chi3l3	ENSMUSG00000040809	chr3	105950472	106022425	-	1700	7.098	1
Prss12	ENSMUSG00000027978	chr3	123149831	123209520	+	2607	7.077	1
Egr2	ENSMUSG00000037868	chr10	66998223	67004936	+	3398	7.076	1
Mmp12	ENSMUSG00000049723	chr9	7344381	7369499	+	7237	7.075	1
Rai14	ENSMUSG00000022246	chr15	10498734	10644386	-	5008	7.067	1
Depdc7	ENSMUSG00000027173	chr2	104561941	104583035	-	2096	7.052	1
Gcnt1	ENSMUSG00000038843	chr19	17400631	17431157	-	5583	7.052	1
Slc25a24	ENSMUSG00000040322	chr3	108926067	108971375	+	3431	7.052	1

Vegfc	ENSMUSG00000031520	chr8	55162886	55271808	+	1896	7.044	1
Gm10053	ENSMUSG00000058927	chr19	24950227	24950702	+	476	7.035	1
Gm7665	ENSMUSG00000063628	chr18	16433157	16433453	-	297	7.035	1
Igkv14-111	ENSMUSG00000076519	chr6	68206398	68206863	+	353	7.035	1
Igkv6-15	ENSMUSG00000076592	chr6	70356463	70356986	-	347	7.035	1
Wfdc15b	ENSMUSG00000018211	chr2	164040190	164047396	-	571	7.035	1
Satb2	ENSMUSG00000038331	chr1	56850830	57035494	-	6783	7.004	1
Sestd1	ENSMUSG00000042272	chr2	77018397	77129163	-	9885	6.992	1
Tmem163	ENSMUSG00000026347	chr1	129383123	129574673	-	2840	6.972	1
Asap2	ENSMUSG00000052632	chr12	20996320	21276032	+	6971	6.967	1
Ucn2	ENSMUSG00000049699	chr9	108888208	108889209	+	1002	6.961	1
Bcl2a1d	ENSMUSG00000074148	chr9	88618126	88626708	-	816	6.957	1
Gm15230	ENSMUSG00000084920	chrX	163295601	163302381	-	510	6.957	1
Prl3d1	ENSMUSG00000057170	chr13	27186081	27192124	+	850	6.957	1
Spink3	ENSMUSG00000024503	chr18	43887723	43896891	-	422	6.957	1
Apbb1ip	ENSMUSG00000026786	chr2	22629614	22731173	+	7414	6.93	1
Gpr97	ENSMUSG00000060470	chr8	97541592	97569147	+	2436	6.899	1
Tmem156	ENSMUSG00000037913	chr5	65456368	65482794	-	888	6.876	1
AI607873	ENSMUSG00000073490	chr1	175653814	175671878	-	3304	6.849	1
Ctla2a	ENSMUSG00000044258	chr13	61035516	61037986	-	1373	6.794	1
Apln	ENSMUSG00000037010	chrX	45378323	45388030	-	3149	6.79	1
Spry4	ENSMUSG00000024427	chr18	38745922	38760922	-	4587	6.789	1
6030455H03Rik	ENSMUSG00000086591	chr11	69262050	69267178	+	859	6.789	1
Gm5611	ENSMUSG00000090602	chr9	16834489	16835343	+	855	6.789	1
Gm8258	ENSMUSG00000048334	chr5	105204930	105205843	+	624	6.789	1
Mrc1	ENSMUSG00000026712	chr2	14151041	14253684	+	5947	6.783	1
Stim2	ENSMUSG00000039156	chr5	54389656	54512296	+	5215	6.782	1
Col3a1	ENSMUSG00000026043	chr1	45368383	45406551	+	8393	6.781	1
Bcl2l15	ENSMUSG00000044165	chr3	103636524	103658541	+	2188	6.781	1
Tmem117	ENSMUSG00000063296	chr15	94459616	94926528	+	2781	6.753	1
Tnc	ENSMUSG00000028364	chr4	63620819	63681760	-	7509	6.738	1
Cd163	ENSMUSG00000008845	chr6	124254669	124280545	+	4492	6.698	1
Hist1h2al	ENSMUSG00000091383	chr13	51298057	51298434	-	378	6.697	1
Ifne	ENSMUSG00000045364	chr4	88525442	88526105	-	664	6.697	1

Ighv8-8	ENSMUSG00000076719	chr12	116532289	116532709	-	336	6.697		1
Stfa2l1	ENSMUSG00000059657	chr16	36156897	36162034	+	414	6.697		1
Plat	ENSMUSG00000031538	chr8	23868216	23893319	+	2531	6.695		1
Msr1	ENSMUSG00000025044	chr8	40667054	40728032	-	4015	6.678		1
Ubash3b	ENSMUSG00000032020	chr9	40819181	40969780	-	7326	6.676		1
Pcyt1b	ENSMUSG00000035246	chrX	90900202	90995290	+	5357	6.671		1
Obfc2a	ENSMUSG00000026107	chr1	51526342	51535243	-	2828	6.668		1
Soat1	ENSMUSG00000026600	chr1	158358239	158404459	-	4785	6.651		1
Cdh2	ENSMUSG00000024304	chr18	16747386	16967755	-	8066	6.643		1
Gem	ENSMUSG00000028214	chr4	11631604	11641899	+	2104	6.636		1
Evi2a	ENSMUSG00000078771	chr11	79340062	79344111	-	2297	6.612		1
Anxa1	ENSMUSG00000024659	chr19	20447918	20465434	-	2608	6.604		1
Igkv5-48	ENSMUSG00000076563	chr6	69676567	69677119	-	347	6.598		1
S100a7a	ENSMUSG00000063767	chr3	90458224	90462052	+	741	6.598		1
Dusp6	ENSMUSG00000019960	chr10	98725865	98730122	+	2796	6.583		1
Ugt1a7c	ENSMUSG00000090124	chr1	89991637	90116577	+	3233	6.553		1
Slc20a1	ENSMUSG00000027397	chr2	129024500	129037352	+	5580	6.547		1
Bmp2	ENSMUSG00000027358	chr2	133377895	133388621	+	3555	6.546		1
Kcnn4	ENSMUSG00000054342	chr7	25155282	25170228	+	2527	6.531		1
Arg1	ENSMUSG00000019987	chr10	24635014	24647276	-	1476	6.524		1
St6gal1	ENSMUSG00000022885	chr16	23224813	23360421	+	4914	6.522		1
Cep170	ENSMUSG00000057335	chr1	178663784	178744198	-	9039	6.52		1
Igfbp3	ENSMUSG00000020427	chr11	7106089	7113926	-	3130	6.515		1
Pcdh7	ENSMUSG00000029108	chr5	58109260	58520590	+	6204	6.511		1
Arntl2	ENSMUSG00000040187	chr6	146744577	146782051	+	3032	6.506		1
Cks2	ENSMUSG00000062248	chr13	51740601	51746029	+	692	6.492		1
Gm13014	ENSMUSG00000084943	chr4	116796582	116797771	-	345	6.492		1
Gm14376	ENSMUSG00000085228	chr7	151655438	151655898	-	297	6.492		1
Ighv1-9	ENSMUSG00000076689	chr12	115821780	115822213	-	351	6.492		1
Igkv8-24	ENSMUSG00000076583	chr6	70166852	70167415	-	365	6.492		1
Trbj1-6	ENSMUSG00000076488	chr6	41485553	41485605	+	53	6.492		1
Lilrb3	ENSMUSG00000058818	chr7	3663011	3671993	-	4471	6.485		1
Anxa3	ENSMUSG00000029484	chr5	97222404	97274987	+	1597	6.478		1
Sema7a	ENSMUSG00000038264	chr9	57787920	57810671	+	3296	6.477		1
Shc4	ENSMUSG00000035109	chr2	125453183	125549884	-	8263	6.476		1
Mex3c	ENSMUSG00000037253	chr18	73732698	73752229	+	3738	6.472		1
Flrt3	ENSMUSG00000051379	chr2	140476650	140497205	-	4865	6.462		1
Akna	ENSMUSG00000039158	chr4	63028159	63064479	-	5918	6.45		1
Col8a1	ENSMUSG00000068196	chr16	57624371	57754850	-	5108	6.448		1
Mapk6	ENSMUSG00000042688	chr9	75216869	75257812	-	6675	6.435		1

Gata6	ENSMUSG00000005836	chr18	11052508	11085633	+	3194	6.425	1
Pls1	ENSMUSG00000049493	chr9	95653061	95745730	-	3890	6.407	1
Timp1	ENSMUSG00000001131	chrX	20447292	20451861	+	888	6.397	1
Epha2	ENSMUSG00000006445	chr4	140857155	140885299	+	4674	6.393	1
Ptpre	ENSMUSG00000041836	chr7	142729507	142877977	+	6571	6.384	1
2610044O15Rik	ENSMUSG00000066057	chr17	95160590	95164201	-	390	6.378	1
Gm13264	ENSMUSG00000085257	chr2	10958501	10970693	+	302	6.378	1
Gm17635	ENSMUSG00000090756	chr18	90761007	90761174	+	168	6.378	1
Gm9804	ENSMUSG00000045946	chr12	50502264	50502824	+	561	6.378	1
Hamp	ENSMUSG00000050440	chr7	31727390	31729036	-	392	6.378	1
Ighv6-6	ENSMUSG00000076680	chr12	115672999	115673455	-	357	6.378	1
Igkv3-5	ENSMUSG00000076599	chr6	70613292	70613889	+	359	6.378	1
Prim2	ENSMUSG00000026134	chr1	33510656	33726631	-	2169	6.369	1
P2ry12	ENSMUSG00000036353	chr3	59020193	59066753	-	2379	6.358	1
Slc30a4	ENSMUSG00000005802	chr2	122506969	122528399	-	5482	6.351	1
Kcnh1	ENSMUSG00000058248	chr1	194014666	194334041	+	7321	6.344	1
Jam2	ENSMUSG00000053062	chr16	84774368	84826173	+	5872	6.319	1
Vim	ENSMUSG00000026728	chr2	13495554	13504453	+	2335	6.311	1
Sytl2	ENSMUSG00000030616	chr7	97497209	97558948	+	6374	6.291	1
Phldb2	ENSMUSG00000033149	chr16	45746346	45953711	-	7587	6.279	1
Rin2	ENSMUSG00000001768	chr2	145610470	145713352	+	5399	6.273	1
Rap2b	ENSMUSG00000036894	chr3	61168429	61172625	+	4197	6.272	1
Il18rap	ENSMUSG00000026068	chr1	40572207	40608550	+	6752	6.258	1
4732440D04Rik	ENSMUSG00000090031	chr1	6203374	6204671	-	413	6.254	1
Gm15956	ENSMUSG00000086935	chr18	12352277	12357184	+	502	6.254	1
Ighv1-12	ENSMUSG00000076691	chr12	115854061	115854354	-	294	6.254	1
Igkv15-103	ENSMUSG00000076523	chr6	68387462	68387919	+	347	6.254	1
Igkv8-30	ENSMUSG00000076577	chr6	70067055	70067611	-	365	6.254	1
Cenpe	ENSMUSG00000045328	chr3	134875527	134936504	+	7813	6.252	1
Slc12a2	ENSMUSG00000024597	chr18	58038332	58106475	+	6520	6.239	1
Prg4	ENSMUSG00000006014	chr1	152296542	152313295	-	4280	6.238	1
Frmd4a	ENSMUSG00000026657	chr2	3938763	4535089	+	23350	6.228	1
Specc1	ENSMUSG00000042331	chr11	61770281	62036515	+	13276	6.225	1
Lhx6	ENSMUSG00000026890	chr2	35937470	35960928	-	4862	6.219	1
Rgs1	ENSMUSG00000026358	chr1	146091102	146096372	-	3830	6.217	1
Fam169b	ENSMUSG00000074071	chr7	75418725	75507975	+	3074	6.202	1

Plxna4	ENSMUSG00000029765	chr6	32094565	32538192	-	12580	6.194	1
Tnfrsf10b	ENSMUSG00000022074	chr14	70167279	70184210	+	3171	6.194	1
Plaur	ENSMUSG00000046223	chr7	25247519	25260892	+	1396	6.193	1
Gm20544	ENSMUSG00000092569	chr18	75524986	75526742	-	690	6.191	1
Serpinb6b	ENSMUSG00000042842	chr13	33057078	33070936	+	2200	6.191	1
Ptp4a1	ENSMUSG00000026064	chr1	30997148	31006615	-	5761	6.186	1
Abcb1b	ENSMUSG00000028970	chr5	8798147	8866314	+	4344	6.186	1
Popdc3	ENSMUSG00000019848	chr10	44897904	45038258	+	2083	6.185	1
Bdnf	ENSMUSG00000048482	chr2	109514857	109567164	+	6217	6.179	1
Pion	ENSMUSG00000039934	chr5	20692085	20797519	+	3745	6.17	1
Sema3e	ENSMUSG00000063531	chr5	14025276	14256689	+	7581	6.161	1
Tpx2	ENSMUSG00000027469	chr2	152673700	152721057	+	4677	6.154	1
Magohb	ENSMUSG00000030188	chr6	131234406	131243262	-	1720	6.151	1
Hk2	ENSMUSG00000000628	chr6	82675019	82724448	-	5969	6.14	1
B4galt6	ENSMUSG00000056124	chr18	20843100	20904905	-	5808	6.121	1
Gm10226	ENSMUSG00000067929	chr17	21828827	21829081	+	255	6.119	1
Gm13834	ENSMUSG00000084894	chr6	31037609	31037912	-	137	6.119	1
Gm9222	ENSMUSG00000091094	chr12	17889003	17889338	+	336	6.119	1
Ighv14-2	ENSMUSG00000076658	chr12	115232680	115233109	-	351	6.119	1
Ighv8-12	ENSMUSG00000076731	chr12	116886172	116886572	-	315	6.119	1
F10	ENSMUSG00000031444	chr8	13037308	13056676	+	2731	6.118	1
Baiap2l1	ENSMUSG00000038859	chr5	145025395	145118981	-	3366	6.113	1
Cfd	ENSMUSG00000061780	chr10	79353598	79355401	+	923	6.097	1
Smc2	ENSMUSG00000028312	chr4	52452115	52501237	+	5583	6.096	1
Morc3	ENSMUSG00000039456	chr16	93832366	93876317	+	4178	6.093	1
Emb	ENSMUSG00000021728	chr13	118009432	118062905	+	2253	6.089	1
Nckap1l	ENSMUSG00000022488	chr15	103284256	103329240	+	5446	6.075	1
Dck	ENSMUSG00000029366	chr5	89194021	89212306	+	3078	6.068	1
Col7a1	ENSMUSG00000025650	chr9	108855631	108886920	+	10206	6.066	1
Ltbp1	ENSMUSG00000001870	chr17	75404908	75791852	+	10688	6.062	1
Pparg	ENSMUSG00000000440	chr6	115311239	115440419	+	1984	6.06	1
Cd38	ENSMUSG00000029084	chr5	44260048	44303614	+	3014	6.054	1
Msln	ENSMUSG00000063011	chr17	25885559	25891272	-	2114	6.044	1
Cdk6	ENSMUSG00000040274	chr5	3343893	3523218	+	3735	6.041	1
Ccbp2	ENSMUSG00000044534	chr9	121807473	121820182	+	3005	6.028	1
Ctss	ENSMUSG00000038642	chr3	95330708	95360325	+	1358	6.024	1
Gcnt2	ENSMUSG00000021360	chr13	40955501	41056260	+	6266	6.018	1
Hecw2	ENSMUSG00000042807	chr1	53863726	54252012	-	12941	6.013	1

Srgn	ENSMUSG00000020077	chr10	61956581	61990199	-	2196	6.002		1
Tns4	ENSMUSG00000017607	chr11	98926992	98950620	-	5167	5.995		1
Cdc25c	ENSMUSG00000044201	chr18	34892651	34911187	-	1941	5.984		1
2010005 H15Rik	ENSMUSG00000051949	chr16	36253103	36257482	+	347	5.969		1
Gm1309 1	ENSMUSG00000085213	chr4	149940978	149942653	-	469	5.969		1
Ighv1-26	ENSMUSG00000076701	chr12	116026583	116027016	-	351	5.969		1
Igkv9-120	ENSMUSG00000076515	chr6	67999977	68000450	+	353	5.969		1
Capn2	ENSMUSG00000026509	chr1	184397391	184447655	-	3226	5.942		1
Lamb1	ENSMUSG00000002900	chr12	31950099	32014509	+	9917	5.932		1
Bpifa1	ENSMUSG00000027483	chr2	153968616	153974955	+	1545	5.931		1
Emp1	ENSMUSG00000030208	chr6	135312949	135333191	+	3046	5.93		1
Atp10a	ENSMUSG00000025324	chr7	65913572	66084796	+	5469	5.928		1
Mmp8	ENSMUSG00000005800	chr9	7558429	7568486	+	2453	5.924		1
Chsy1	ENSMUSG00000032640	chr7	73254401	73318675	+	4167	5.915		1
Abtb2	ENSMUSG00000032724	chr2	103406467	103558580	+	6032	5.901		1
6330406 I15Rik	ENSMUSG00000029659	chr5	150171051	150234298	+	5499	5.896		1
Lox	ENSMUSG00000024529	chr18	52675723	52689521	-	4697	5.889		1
Glis3	ENSMUSG00000052942	chr19	28333341	28754567	-	8842	5.888		1
Zfp873	ENSMUSG00000061371	chr10	81521162	81524074	+	1893	5.878		1
Rnf2	ENSMUSG00000026484	chr1	153316536	153347953	-	3028	5.875		1
Rnd3	ENSMUSG00000017144	chr2	50985958	51004631	-	4432	5.867		1
Sarnp	ENSMUSG00000078427	chr10	128258827	128314694	+	1062	5.862		1
Steap2	ENSMUSG00000015653	chr5	5664831	5694578	-	12142	5.861		1
Pla2g7	ENSMUSG00000023913	chr17	43705047	43749150	+	2905	5.857		1
Osbpl8	ENSMUSG00000020189	chr10	110601858	110734303	+	7152	5.857		1
Fam84a	ENSMUSG00000020607	chr12	14154405	14158844	-	3379	5.843		1
Aldh1a3	ENSMUSG00000015134	chr7	73535778	73572403	-	4615	5.837		1
Hace1	ENSMUSG00000038822	chr10	45297635	45432151	+	12851	5.832		1
2310007 B03Rik	ENSMUSG00000034159	chr1	95047926	95057525	-	2368	5.829		1
Anks1b	ENSMUSG00000058589	chr10	89336417	90435730	+	10178	5.827		1
Bean1	ENSMUSG00000031872	chr8	106694413	106742992	+	3386	5.82		1
Fam167 a	ENSMUSG00000035095	chr14	64055231	64084335	+	4274	5.819		1
Pls3	ENSMUSG00000016382	chrX	73030993	73120521	-	4238	5.817		1
Lamb3	ENSMUSG00000026639	chr1	195128151	195170072	+	4365	5.814		1
B43030 6N03Rik	ENSMUSG00000043740	chr17	48455466	48466349	+	6937	5.813		1
Klrb1b	ENSMUSG00000079298	chr6	128763724	128776349	-	2228	5.813		1
Pygl	ENSMUSG00000021069	chr12	71291798	71332475	-	3828	5.809		1

Ighv1-4	ENSMUSG00000076684	chr12	115725347	115725780	-	351	5.802	1
Ighv5-4	ENSMUSG00000076635	chr12	114835657	114836116	-	353	5.802	1
Ighv5-9-1	ENSMUSG00000076643	chr12	114974322	114974781	-	350	5.802	1
Igkv12-44	ENSMUSG00000076566	chr6	69764625	69765094	-	347	5.802	1
Igkv3-10	ENSMUSG00000076596	chr6	70522627	70523224	+	356	5.802	1
Igkv6-14	ENSMUSG00000076593	chr6	70384946	70385470	-	347	5.802	1
Lepr	ENSMUSG00000057722	chr4	101390009	101487957	+	7049	5.802	1
Klf5	ENSMUSG00000005148	chr14	99697910	99712624	+	1721	5.796	1
Gja1	ENSMUSG00000050953	chr10	56097106	56110225	+	3105	5.789	1
Myef2	ENSMUSG00000027201	chr2	124910364	124949397	-	5936	5.767	1
Msn	ENSMUSG00000031207	chrX	93291381	93363891	+	6327	5.742	1
Dgkh	ENSMUSG00000034731	chr14	78969416	79124896	-	4145	5.742	1
Lama3	ENSMUSG00000024421	chr18	12492533	12741522	+	10537	5.738	1
Diap3	ENSMUSG00000022021	chr14	87055174	87541031	-	4841	5.731	1
Adcy7	ENSMUSG00000031659	chr8	90796302	90853861	+	6330	5.728	1
Foxf2	ENSMUSG00000038402	chr13	31717685	31723272	+	2363	5.726	1
Cpe	ENSMUSG00000037852	chr8	67071355	67171837	-	2087	5.718	1
Ehd3	ENSMUSG00000024065	chr17	74154181	74181433	+	3658	5.71	1
Mafb	ENSMUSG00000074622	chr2	160189413	160192801	-	3389	5.708	1
Pmaip1	ENSMUSG00000024521	chr18	66618258	66625204	+	2646	5.699	1
Fut4	ENSMUSG00000049307	chr9	14552903	14556566	-	3664	5.687	1
Car3	ENSMUSG00000027559	chr3	14863538	14872351	+	1507	5.674	1
Nox4	ENSMUSG00000030562	chr7	94394606	94547220	+	8471	5.657	1
Apbb2	ENSMUSG00000029207	chr5	66690100	67010067	-	10579	5.656	1
Myo1b	ENSMUSG00000018417	chr1	51806609	51972915	-	10792	5.649	1
Fmnl2	ENSMUSG00000036053	chr2	52716894	52993236	+	6042	5.627	1
Anln	ENSMUSG00000036777	chr9	22135658	22193650	-	5421	5.626	1
Dclk1	ENSMUSG00000027797	chr3	55046448	55342986	+	13514	5.623	1
Abcc1	ENSMUSG00000023088	chr16	14361651	14475830	+	12366	5.617	1
D330041H03Rik	ENSMUSG00000073437	chr17	24548972	24550569	-	285	5.613	1
Ighv10-3	ENSMUSG00000076686	chr12	115761652	115762098	-	359	5.613	1
Ighv14-3	ENSMUSG00000076660	chr12	115298056	115298484	-	351	5.613	1
Ighv1-64	ENSMUSG00000076726	chr12	116745756	116746188	-	351	5.613	1
Ighv1-80	ENSMUSG00000076738	chr12	117150555	117150987	-	351	5.613	1
Ighv5-17	ENSMUSG00000076650	chr12	115097360	115097827	-	348	5.613	1
Lhfp	ENSMUSG00000048332	chr3	52845450	53065601	+	2108	5.603	1
Col1a1	ENSMUSG00000001506	chr11	94797538	94814356	+	6563	5.6	1
Ttc9	ENSMUSG00000042734	chr12	82732356	82765928	+	2042	5.595	1

Zfp52	ENSMUSG00000051341	chr17	21672503	21699565	+	3164	5.589	1
Serpina3n	ENSMUSG00000021091	chr12	105644939	105652539	+	2064	5.588	1
Apobr	ENSMUSG00000042759	chr7	133728456	133732626	+	3779	5.581	1
9930013L23Rik	ENSMUSG00000052353	chr7	91081367	91235012	-	12232	5.572	1
Fgf2	ENSMUSG00000037225	chr3	37211441	37304299	+	1802	5.572	1
Narg2	ENSMUSG00000032235	chr9	69245713	69280929	+	7736	5.571	1
Zfp81	ENSMUSG00000003929	chr17	33466287	33495823	-	6639	5.571	1
4831426I19Rik	ENSMUSG00000054150	chr12	106168159	106248019	-	5532	5.566	1
Gda	ENSMUSG00000058624	chr19	21465797	21547935	-	6135	5.549	1
Reg3g	ENSMUSG00000030017	chr6	78416263	78418866	-	2114	5.54	1
Adam19	ENSMUSG00000011256	chr11	45868004	45960845	+	7499	5.525	1
Ccdc88c	ENSMUSG00000021182	chr12	102149734	102267269	-	8257	5.521	1
Zfp532	ENSMUSG00000042439	chr18	65739884	65849088	+	6586	5.505	1
Rapgef4	ENSMUSG00000049044	chr2	71819272	72095531	+	6722	5.501	1
C1galt1	ENSMUSG00000042460	chr6	7794842	7822047	+	2452	5.496	1
Egln3	ENSMUSG00000035105	chr12	55279968	55304847	-	2662	5.492	1
Saa3	ENSMUSG00000040026	chr7	53967368	53971046	-	531	5.486	1
Hells	ENSMUSG00000025001	chr19	39005405	39045541	+	7205	5.485	1
Procr	ENSMUSG00000027611	chr2	155576853	155581207	+	2031	5.479	1
Ccdc88a	ENSMUSG00000032740	chr11	29273658	29410812	+	10895	5.478	1
Fosb	ENSMUSG00000003545	chr7	19888070	19895394	-	3751	5.474	1
Exosc9	ENSMUSG00000027714	chr3	36451528	36464651	+	4711	5.467	1
Dock10	ENSMUSG00000038608	chr1	80497648	80755128	-	8038	5.45	1
Fcgr4	ENSMUSG00000059089	chr1	172949051	172959892	+	1256	5.45	1
Lpgat1	ENSMUSG00000026623	chr1	193541713	193608134	+	7610	5.449	1
Alox5	ENSMUSG00000025701	chr6	116360095	116411196	-	4106	5.446	1
Kif4	ENSMUSG00000034311	chrX	97821076	97922553	+	5514	5.446	1
Cpxm1	ENSMUSG00000027408	chr2	130216511	130223310	-	2432	5.444	1
Gm17322	ENSMUSG00000091267	chr9	57845848	57855258	+	2169	5.444	1
Bhlhe40	ENSMUSG00000030103	chr6	108610623	108616919	+	3272	5.444	1
Cd300lb	ENSMUSG00000063193	chr11	114784095	114795700	-	2917	5.44	1
Pitpnc1	ENSMUSG00000040430	chr11	107069206	107332034	-	6635	5.436	1
Lilrb4	ENSMUSG00000062593	chr10	51200446	51216419	+	1491	5.425	1
Dip2a	ENSMUSG00000020231	chr10	75722174	75808036	-	8215	5.419	1
Eps8	ENSMUSG00000015766	chr6	137425766	137603397	-	5731	5.418	1
Casp3	ENSMUSG00000031628	chr8	47702803	47724060	+	1455	5.416	1
Gatm	ENSMUSG00000027199	chr2	122420203	122437039	-	3001	5.416	1
Acsl4	ENSMUSG00000031278	chrX	138752536	138825078	-	5405	5.414	1

Mki67	ENSMUSG00000031004	chr7	142881471	142908062	-	10075	5.409	1
Nes	ENSMUSG00000004891	chr3	87775000	87784373	+	6416	5.399	1
Zdhhc17	ENSMUSG00000035798	chr10	110378838	110447122	-	4948	5.396	1
Gm20405	ENSMUSG00000092495	chr15	65689431	65689918	+	229	5.395	1
Ighv1-18	ENSMUSG00000076695	chr12	115920885	115921276	-	306	5.395	1
Ighv1-76	ENSMUSG00000091843	chr12	117086092	117086385	-	294	5.395	1
Igkv4-63	ENSMUSG00000076550	chr6	69327938	69328223	-	286	5.395	1
Igkv8-19	ENSMUSG00000076588	chr6	70290870	70291174	-	305	5.395	1
Stk10	ENSMUSG00000020272	chr11	32433305	32524587	+	5187	5.364	1
C230081A13Rik	ENSMUSG00000074305	chr9	56048938	56265857	-	10977	5.363	1
Armcx4	ENSMUSG00000049804	chrX	131221058	131231296	+	7996	5.354	1
Etohi1	ENSMUSG00000074519	chr2	177757989	177768727	+	2045	5.348	1
Wsb1	ENSMUSG00000017677	chr11	79052874	79068173	-	3987	5.342	1
Dock7	ENSMUSG00000028556	chr4	98603356	98787606	-	8115	5.329	1
Sh3pxd2b	ENSMUSG00000040711	chr11	32247820	32328173	+	9709	5.328	1
Echdc1	ENSMUSG00000019883	chr10	29032972	29066467	+	3317	5.32	1
Cd33	ENSMUSG00000004609	chr7	50782826	50788541	-	2571	5.318	1
Lif	ENSMUSG00000034394	chr11	4157560	4172517	+	4350	5.317	1
Ipo7	ENSMUSG00000066232	chr7	117161939	117198628	+	3117	5.309	1
Lamc2	ENSMUSG00000026479	chr1	154969886	155033577	-	5164	5.307	1
Hif1a	ENSMUSG00000021109	chr12	75002362	75048517	+	5896	5.299	1
Haus6	ENSMUSG00000038047	chr4	86224759	86257959	-	6459	5.298	1
Runx1	ENSMUSG00000022952	chr16	92601712	92826311	-	7798	5.279	1
Rnf24	ENSMUSG00000048911	chr2	131123800	131178628	-	6801	5.278	1
Trim59	ENSMUSG00000034317	chr3	68839210	68848677	-	3352	5.267	1
Plcl1	ENSMUSG00000038349	chr1	55463232	55808646	+	3632	5.263	1
Apobec1	ENSMUSG00000040613	chr6	122527810	122552462	-	2391	5.25	1
Col5a1	ENSMUSG00000026837	chr2	27741945	27895034	+	8490	5.241	1
Glipr1	ENSMUSG00000056888	chr10	111422504	111439687	-	1543	5.235	1
Pter	ENSMUSG00000026730	chr2	12845668	12925080	+	3705	5.223	1
Rhobtb3	ENSMUSG00000021589	chr13	76006985	76081373	-	5684	5.216	1
Apoa1	ENSMUSG00000032083	chr9	46036663	46038549	+	1236	5.213	1
E030010A14Rik	ENSMUSG00000048572	chr19	24748498	24754151	+	2462	5.213	1
Cyyr1	ENSMUSG00000041134	chr16	85421778	85553642	-	8894	5.21	1
Dyrk3	ENSMUSG00000016526	chr1	133025018	133034811	-	2072	5.206	1
Ms4a6d	ENSMUSG00000024679	chr19	11661094	11679339	-	1735	5.202	1
Arc	ENSMUSG00000022602	chr15	74499513	74503000	-	3057	5.197	1

D230004N17Rik	ENSMUSG00000087130	chr9	41283020	41423263	+	2991	5.194	1
Ncapg	ENSMUSG00000015880	chr5	46061161	46091783	+	7061	5.193	1
Ugdh	ENSMUSG00000029201	chr5	65804441	65827185	-	6799	5.182	1
Zfp748	ENSMUSG00000069204	chr13	67639580	67654065	-	4603	5.181	1
Vldlr	ENSMUSG00000024924	chr19	27290974	27328721	+	10068	5.18	1
Pdp1	ENSMUSG00000049225	chr4	11885331	11893599	-	4507	5.177	1
March1	ENSMUSG00000036469	chr8	68141799	68995536	+	7167	5.171	1
Snhg5	ENSMUSG00000032424	chr9	88415894	88417721	-	993	5.164	1
Tcp11l1	ENSMUSG00000027175	chr2	104497445	104552326	-	6877	5.147	1
Ltbp2	ENSMUSG00000002020	chr12	86124162	86217482	-	8193	5.144	1
Gm17583	ENSMUSG00000090413	chr15	89265932	89271036	+	69	5.139	1
Trdj1	ENSMUSG00000076870	chr14	54742813	54742863	+	51	5.139	1
Lrrk2	ENSMUSG00000036273	chr15	91503606	91646551	+	11834	5.137	1
Fgf11	ENSMUSG00000042826	chr11	69609570	69615359	-	3044	5.137	1
Osbpl3	ENSMUSG00000029822	chr6	50243326	50406200	-	9031	5.133	1
Akr1b8	ENSMUSG00000029762	chr6	34304119	34318463	+	1499	5.125	1
Polk	ENSMUSG00000021668	chr13	97250644	97312440	-	4289	5.122	1
Pip4k2a	ENSMUSG00000026737	chr2	18763882	18919753	-	3477	5.12	1
Dsn1	ENSMUSG00000027635	chr2	156821001	156832890	-	3354	5.106	1
Dse	ENSMUSG00000039497	chr10	33871200	33927357	-	4224	5.105	1
Galc	ENSMUSG00000021003	chr12	99440514	99497669	-	3861	5.099	1
Abca9	ENSMUSG00000041797	chr11	109962063	110029510	-	8048	5.099	1
Sp6	ENSMUSG00000038560	chr11	96874708	96886048	+	3841	5.098	1
Nid1	ENSMUSG00000005397	chr13	13529869	13604542	+	6046	5.092	1
Tank	ENSMUSG00000064289	chr2	61416642	61492228	+	6096	5.09	1
Ankrd44	ENSMUSG00000052331	chr1	54702184	54983231	-	6194	5.078	1
Rrm2	ENSMUSG00000020649	chr12	25393106	25399011	+	2771	5.078	1
Galnt3	ENSMUSG00000026994	chr2	65920823	65963051	-	4374	5.077	1
Ccdc76	ENSMUSG00000033439	chr3	116284011	116317505	-	5035	5.074	1
Ppfibp1	ENSMUSG00000016487	chr6	146837009	146980547	+	9790	5.073	1
Ppic	ENSMUSG00000024538	chr18	53565995	53577661	-	1286	5.071	1
Zdhhc13	ENSMUSG00000030471	chr7	56044373	56082810	+	2410	5.069	1
Fbln2	ENSMUSG00000064080	chr6	91162449	91222534	+	5876	5.067	1
Itgb1	ENSMUSG00000025809	chr8	131209554	131257100	+	4081	5.065	1
Sh3pxd2a	ENSMUSG00000053617	chr19	47334664	47538901	-	10464	5.061	1
Chrnb1	ENSMUSG00000041189	chr11	69597538	69609445	-	2531	5.059	1
Lyn	ENSMUSG00000042228	chr4	3605262	3740269	+	4073	5.054	1
Wdr19	ENSMUSG00000037890	chr5	65590935	65651654	+	4409	5.051	1
Haus3	ENSMUSG00000079555	chr5	34496570	34512094	-	1950	5.048	1
Eea1	ENSMUSG00000036499	chr10	95403297	95508152	+	7986	5.043	1
Rpl29	ENSMUSG00000048758	chr9	106331785	106333899	+	796	5.036	1

Lnpep	ENSMUSG00000023845	chr17	17664687	17761453	-	5181	5.025		1
Ets1	ENSMUSG00000032035	chr9	32443806	32565405	+	6422	5.015		1
Gbp7	ENSMUSG00000040253	chr3	142193302	142213047	+	5552	5.007		1
Mmp14	ENSMUSG00000000957	chr14	55050441	55060095	+	2597	5.005		1
Adap2	ENSMUSG00000020709	chr11	79967607	79992460	+	2602	4.999		1
Gls	ENSMUSG00000026103	chr1	52220292	52290076	-	15057	4.995		1
Rbms1	ENSMUSG00000026970	chr2	60588250	60801261	-	5485	4.994		1
Med7	ENSMUSG00000020397	chr11	46250427	46256223	+	2871	4.993		1
Irx3	ENSMUSG00000031734	chr8	94322424	94325966	-	2762	4.99		1
Car12	ENSMUSG00000032373	chr9	66561493	66614652	+	4539	4.986		1
Dennd2c	ENSMUSG00000007379	chr3	102906527	102973692	+	6858	4.983		1
Spag5	ENSMUSG00000002055	chr11	78115031	78135959	+	5624	4.98		1
Qk	ENSMUSG00000062078	chr17	10399336	10512245	-	9887	4.975		1
Nek6	ENSMUSG00000026749	chr2	38367163	38443561	+	4149	4.973		1
Pxdn	ENSMUSG00000020674	chr12	30622901	30702523	+	9517	4.971		1
Pmepa1	ENSMUSG00000038400	chr2	173049959	173102034	-	4913	4.964		1
Mpp6	ENSMUSG00000038388	chr6	50060240	50148597	+	2843	4.96		1
G530011O06Rik	ENSMUSG00000072844	chrX	166412975	166416849	-	2380	4.958		1
Nap1l1	ENSMUSG00000058799	chr10	110910248	110935198	+	4179	4.952		1
Fcgr2b	ENSMUSG00000026656	chr1	172890689	172906678	-	3849	4.952		1
Ampd3	ENSMUSG00000005686	chr7	117911720	117955919	+	5605	4.95		1
Lyve1	ENSMUSG00000030787	chr7	117994121	118006467	-	2607	4.949		1
Lgi2	ENSMUSG00000039252	chr5	52924758	52957542	-	6367	4.937		1
Slco2a1	ENSMUSG00000032548	chr9	102910817	102990179	+	4035	4.935		1
Angptl4	ENSMUSG00000002289	chr17	33910695	33918520	-	3286	4.932		1
Mipol1	ENSMUSG00000047022	chr12	58331412	58598186	+	9358	4.932		1
Tnfaip2	ENSMUSG00000021281	chr12	112680680	112693229	+	4984	4.928		1
Pcdh17	ENSMUSG00000035566	chr14	84843370	84936867	+	9509	4.926		1
Mboat2	ENSMUSG00000020646	chr12	25516464	25645166	+	2833	4.925		1
Csf2rb2	ENSMUSG00000071714	chr15	78112947	78136052	-	4631	4.923		1
Ppbp	ENSMUSG00000029372	chr5	91197544	91199086	+	1080	4.922		1
Hal	ENSMUSG00000020017	chr10	92951513	92982049	+	5549	4.918		1
Ccdc72	ENSMUSG00000091537	chr9	108980502	108984895	-	637	4.911		1
Atp7a	ENSMUSG00000033792	chrX	103222615	103320265	+	8734	4.908		1
Fcho2	ENSMUSG00000041685	chr13	99493362	99585404	-	5250	4.905		1
Slfn1	ENSMUSG00000078763	chr11	82930351	82936172	+	1864	4.901		1
Nbn	ENSMUSG00000028224	chr4	15885072	15919736	+	2533	4.901		1
Stambpl1	ENSMUSG00000024776	chr19	34266719	34314823	+	5621	4.9		1
Yes1	ENSMUSG00000014932	chr5	32913606	32989430	+	4537	4.9		1
Errfi1	ENSMUSG00000028967	chr4	150228028	150243001	+	3582	4.898		1
Card6	ENSMUSG00000041849	chr15	5045981	5058539	-	6101	4.89		1

D19Ertd737e	ENSMUSG00000057858	chr19	60274489	60302600	-	2035	4.882	1
Mmd	ENSMUSG00000003948	chr11	90048998	90139903	+	5386	4.879	1
Kif2c	ENSMUSG00000028678	chr4	116832244	116855244	-	4391	4.875	1
Plek	ENSMUSG00000020120	chr11	16871209	16952384	-	4486	4.875	1
Zbtb41	ENSMUSG00000033964	chr1	141318960	141349582	+	8360	4.871	1
Col1a2	ENSMUSG00000029661	chr6	4454814	4491543	+	12145	4.866	1
Klk8	ENSMUSG00000064023	chr7	51052947	51059192	+	1322	4.864	1
Dusp7	ENSMUSG00000053716	chr9	106270963	106278055	+	3356	4.863	1
Atrnl1	ENSMUSG00000054843	chr19	57685524	58207828	+	6600	4.86	1
Kif23	ENSMUSG00000032254	chr9	61765087	61794606	-	3918	4.853	1
Onecut2	ENSMUSG00000045991	chr18	64500018	64558140	+	13389	4.844	1
Retnlg	ENSMUSG00000022651	chr16	48872721	48874611	+	608	4.839	1
Gm16721	ENSMUSG00000087419	chr1	184414398	184414958	+	33	4.828	1
Fam111a	ENSMUSG00000024691	chr19	12620230	12664258	+	4125	4.823	1
Ccl6	ENSMUSG00000018927	chr11	83401384	83406589	-	1795	4.823	1
6330503K22Rik	ENSMUSG00000033904	chr7	125856066	125880538	+	8266	4.82	1
Clu	ENSMUSG00000022037	chr14	66587320	66600385	+	3438	4.811	1
Uck2	ENSMUSG00000026558	chr1	169156221	169215258	-	1331	4.809	1
Gm2382	ENSMUSG00000079427	chr9	88583443	88614636	-	815	4.809	1
Ncapg2	ENSMUSG00000042029	chr12	117643875	117702004	+	6604	4.807	1
Aff2	ENSMUSG00000031189	chrX	66613469	67121212	+	7267	4.806	1
Fam102b	ENSMUSG00000040339	chr3	108773915	108830525	-	5702	4.804	1
Ptpdc1	ENSMUSG00000038042	chr13	48673239	48721033	-	4587	4.799	1
Loxl2	ENSMUSG00000034205	chr14	70008978	70095640	+	5346	4.796	1
Tpm4	ENSMUSG00000031799	chr8	74658874	74677041	+	2203	4.795	1
9430020K01Rik	ENSMUSG00000033960	chr18	4634927	4682866	+	6730	4.791	1
Itgam	ENSMUSG00000030786	chr7	135206154	135271674	+	6377	4.785	1
Serpina3m	ENSMUSG00000079012	chr12	105625374	105632467	+	1659	4.782	1
Fam105a	ENSMUSG00000056069	chr15	27584826	27611262	-	2945	4.778	1
Rtn4	ENSMUSG00000020458	chr11	29592947	29644331	+	6991	4.77	1
Zfp760	ENSMUSG00000067928	chr17	21844708	21862603	+	4209	4.767	1
Sh3kbp1	ENSMUSG00000040990	chrX	156065204	156416001	+	10112	4.757	1
F13a1	ENSMUSG00000039109	chr13	36959047	37142113	-	6443	4.756	1
Lmln	ENSMUSG00000022802	chr16	33062607	33127781	+	6511	4.747	1
Ccr5	ENSMUSG00000079227	chr9	124036282	124062438	+	3704	4.742	1
Lrp12	ENSMUSG00000022305	chr15	39702149	39775540	-	4574	4.737	1
Lcn2	ENSMUSG00000026822	chr2	32240153	32243317	-	2302	4.733	1

Fgf18	ENSMUSG00000057967	chr11	33017430	33047400	-	1187	4.732		1
Rassf8	ENSMUSG00000030259	chr6	145695268	145769599	+	5559	4.73		1
Ust	ENSMUSG00000047712	chr10	7924551	8238623	-	4284	4.721		1
Mpeg1	ENSMUSG00000046805	chr19	12535269	12539774	+	4335	4.72		1
Vcan	ENSMUSG00000021614	chr13	89794915	89882117	-	14467	4.719		1
Gramd4	ENSMUSG00000035900	chr15	85888125	85968064	+	5567	4.717		1
A430105I19Rik	ENSMUSG00000045838	chr2	118579894	118588397	-	4779	4.709		1
Ncoa7	ENSMUSG00000039697	chr10	30356342	30522913	-	5360	4.707		1
Nup160	ENSMUSG00000051329	chr2	90517372	90576485	+	8761	4.707		1
Prune2	ENSMUSG00000039126	chr19	17030608	17298422	+	13653	4.705		1
Galnt1	ENSMUSG00000000420	chr18	24363845	24445319	+	5517	4.704		1
Atp11c	ENSMUSG00000062949	chrX	57476467	57845873	-	7333	4.698		1
Galnt6	ENSMUSG00000037280	chr15	100522244	100559807	-	3871	4.698		1
Krt13	ENSMUSG00000044041	chr11	99978641	99982880	-	1821	4.695		1
Itga6	ENSMUSG00000027111	chr2	71583673	71696473	+	9025	4.694		1
Zfp280c	ENSMUSG00000036916	chrX	45894802	45947681	-	5236	4.694		1
Kif15	ENSMUSG00000036768	chr9	122860199	122927843	+	4815	4.689		1
Adamts1	ENSMUSG00000022893	chr16	85794072	85803358	-	5302	4.687		1
Nck2	ENSMUSG00000066877	chr1	43502596	43627360	+	4193	4.686		1
Pdss1	ENSMUSG00000026784	chr2	22751042	22795786	+	4248	4.685		1
Cdkn2b	ENSMUSG00000073802	chr4	88952198	88956941	-	1393	4.685		1
Shroom4	ENSMUSG00000068270	chrX	5976972	6214566	+	8880	4.684		1
Ms4a6c	ENSMUSG00000079419	chr19	11543858	11556687	+	1848	4.684		1
Sept6	ENSMUSG00000050379	chrX	34451321	34531789	-	9882	4.677		1
Sprr2a3	ENSMUSG00000074445	chr3	92054654	92093169	+	722	4.676		1
Rap2c	ENSMUSG00000050029	chrX	48357089	48371195	-	3545	4.675		1
Nae1	ENSMUSG00000031878	chr8	107034928	107058537	-	7792	4.673		1
Slc4a7	ENSMUSG00000021733	chr14	15535539	15632457	+	7582	4.669		1
Ephx2	ENSMUSG00000022040	chr14	66703214	66743337	-	2167	4.665		1
Acot10	ENSMUSG00000047565	chr15	20594974	20596505	-	1532	4.662		1
Rb1	ENSMUSG00000022105	chr14	73583480	73725629	-	6015	4.661		1
Map4k4	ENSMUSG00000026074	chr1	39957758	40083155	+	6080	4.66		1
Tmod3	ENSMUSG00000058587	chr9	75345603	75407464	-	3578	4.657		1
Eltd1	ENSMUSG00000039167	chr3	151100851	151208050	+	4343	4.653		1
Epsti1	ENSMUSG00000022014	chr14	78304046	78397975	+	1637	4.649		1
Sept9	ENSMUSG00000059248	chr11	117060975	117223639	+	7565	4.642		1
Cxcr4	ENSMUSG00000045382	chr1	130484776	130488870	-	1808	4.636		1
Cd84	ENSMUSG00000038147	chr1	173769828	173820849	+	6272	4.635		1
Rnf217	ENSMUSG00000063760	chr10	31222119	31328990	-	1893	4.623		1
Srd5a1	ENSMUSG00000021594	chr13	69712327	69750320	-	3019	4.622		1
Nin	ENSMUSG00000021068	chr12	71112422	71212912	-	10345	4.615		1
Zbtb38	ENSMUSG00000040433	chr9	96583182	96653250	-	11070	4.615		1
Postn	ENSMUSG00000027750	chr3	54165031	54194961	+	7838	4.612		1
Mical2	ENSMUSG00000038244	chr7	119369370	119498708	+	11510	4.604		1

Jak2	ENSMUSG00000024789	chr19	29326318	29387570	+	5030	4.599		1
Syne1	ENSMUSG00000019769	chr10	4795849	5326338	+	31152	4.597		1
Meg3	ENSMUSG00000021268	chr12	110779211	110809936	+	18089	4.594		1
Arhgap11a	ENSMUSG00000041219	chr2	113670173	113688818	-	6808	4.591		1
Arap3	ENSMUSG00000024451	chr18	38132278	38157228	-	5999	4.59		1
Map2k1	ENSMUSG00000004936	chr9	64033577	64101438	-	3391	4.59		1
Rab44	ENSMUSG00000064147	chr17	29272001	29285925	+	4109	4.589		1
Syk	ENSMUSG00000021457	chr13	52678542	52744161	+	9362	4.585		1
Sfrp1	ENSMUSG00000031548	chr8	24521974	24560104	+	4372	4.584		1
Epb4.1l2	ENSMUSG00000019978	chr10	25079604	25243320	+	5340	4.581		1
Ptprc	ENSMUSG00000026395	chr1	139959438	140071882	-	5816	4.578		1
Sgcb	ENSMUSG00000029156	chr5	74023988	74038983	-	3740	4.575		1
Mall	ENSMUSG00000027377	chr2	127530122	127555668	-	2006	4.573		1
Fam107b	ENSMUSG00000026655	chr2	3531030	3699414	+	3762	4.57		1
Acap1	ENSMUSG00000001588	chr11	69695069	69709041	-	4878	4.568		1
Parvb	ENSMUSG00000022438	chr15	84062473	84146118	+	4416	4.566		1
Il1b	ENSMUSG00000027398	chr2	129190306	129196875	-	1907	4.565		1
Zfp948	ENSMUSG00000067931	chr17	21704010	21725664	+	2612	4.565		1
Myo5a	ENSMUSG00000034593	chr9	74918822	75071495	+	14803	4.563		1
Aspn	ENSMUSG00000021388	chr13	49639812	49662926	+	2346	4.562		1
Slc35d1	ENSMUSG00000028521	chr4	102843254	102887769	-	8549	4.556		1
Col5a3	ENSMUSG00000004098	chr9	20574494	20619511	-	6809	4.553		1
Irx5	ENSMUSG00000031737	chr8	94881695	94885353	+	2401	4.549		1
Lpar1	ENSMUSG00000038668	chr4	58448127	58566770	-	4289	4.543		1
Mctp2	ENSMUSG00000032776	chr7	79222718	79451481	-	5787	4.537		1
Lrrc8c	ENSMUSG00000054720	chr5	105948407	106042037	+	7654	4.532		1
Alg10b	ENSMUSG00000075470	chr15	90054742	90060985	+	3727	4.523		1
Ggta1	ENSMUSG00000035778	chr2	35255699	35318751	-	4709	4.522		1
Oxr1	ENSMUSG00000022307	chr15	41279028	41692594	+	5081	4.52		1
Ifi204	ENSMUSG00000073489	chr1	175677424	175697050	-	2362	4.517		1
Ttbk2	ENSMUSG00000090100	chr2	120558552	120676340	-	11406	4.517		1
Ccl9	ENSMUSG00000019122	chr11	83386421	83392138	-	3045	4.513		1
Cttnbp2nl	ENSMUSG00000062127	chr3	104804833	104856064	-	5000	4.51		1
Tgfbi	ENSMUSG00000035493	chr13	56710920	56740923	+	2967	4.508		1
Hgfac	ENSMUSG00000029102	chr5	35384202	35391085	+	2103	4.507		1
Dusp5	ENSMUSG00000034765	chr19	53603599	53616921	+	2473	4.507		1
A430084P05Rik	ENSMUSG00000057135	chr11	70604434	70626088	-	1080	4.506		1
1700048O20Rik	ENSMUSG00000043773	chr9	121847253	121856132	+	1642	4.503		1
Frrs1	ENSMUSG00000033386	chr3	116581145	116606668	+	2396	4.495		1
Ahnak	ENSMUSG00000069833	chr19	9063750	9151409	+	18810	4.494		1

Inpp5f	ENSMUSG00000042105	chr7	135754842	135839939	+	8324	4.493	1
Sla	ENSMUSG00000022372	chr15	66612381	66663391	-	9186	4.493	1
Wdr44	ENSMUSG00000036769	chrX	23270177	23383151	+	8353	4.492	1
Vav3	ENSMUSG00000033721	chr3	109143571	109488616	+	6028	4.49	1
Ghr	ENSMUSG00000055737	chr15	3267760	3533492	-	9053	4.49	1
Dmtf1	ENSMUSG00000042508	chr5	9118801	9161776	-	3990	4.489	1
Zfp317	ENSMUSG00000057551	chr9	19445668	19452492	+	1837	4.489	1
Pcsk5	ENSMUSG00000024713	chr19	17507883	17912122	-	8785	4.489	1
Plekha2	ENSMUSG00000031557	chr8	26149616	26212666	-	5397	4.486	1
Shprh	ENSMUSG00000090112	chr10	10869225	10937393	+	14066	4.482	1
Adarb1	ENSMUSG00000020262	chr10	76753471	76881015	-	11272	4.472	1
Tead1	ENSMUSG00000055320	chr7	119822832	120050319	+	11235	4.471	1
Zfand5	ENSMUSG00000024750	chr19	21346768	21356779	+	6193	4.459	1
Ero1l	ENSMUSG00000021831	chr14	45902879	45938237	-	4291	4.458	1
Slbp	ENSMUSG00000004642	chr5	33977601	33995223	-	4472	4.454	1
Tlr8	ENSMUSG00000040522	chrX	163680626	163702261	-	3588	4.45	1
Ptpn12	ENSMUSG00000028771	chr5	20492463	20561729	-	3509	4.45	1
D1Ertd622e	ENSMUSG00000044768	chr1	99540480	99558651	-	3739	4.448	1
Trp53i11	ENSMUSG00000068735	chr2	93027705	93041916	+	3690	4.448	1
Pfkfb3	ENSMUSG00000026773	chr2	11393057	11423728	-	7030	4.446	1
Lrig1	ENSMUSG00000030029	chr6	94554523	94650152	-	5585	4.446	1
Stc1	ENSMUSG00000014813	chr14	69647295	69659861	+	4197	4.444	1
Sept11	ENSMUSG00000058013	chr5	93522483	93605473	+	6089	4.438	1
Asap1	ENSMUSG00000022377	chr15	63918419	64214481	-	7473	4.434	1
Spon1	ENSMUSG00000038156	chr7	120909512	121186884	+	8177	4.43	1
5S_rRNA	ENSMUSG00000088371	chr1	91459935	91460049	+	115	4.429	1
5S_rRNA	ENSMUSG00000088249	chr16	84818342	84818439	+	98	4.429	1
Gm10973	ENSMUSG00000078666	chr3	90058870	90059011	+	78	4.429	1
Traj16	ENSMUSG00000076913	chr14	54822809	54822869	+	61	4.429	1
Ccr1	ENSMUSG00000025804	chr9	123876959	123883525	-	2831	4.426	1
Ankrd50	ENSMUSG00000044864	chr3	38348183	38383766	-	8488	4.424	1
Atp10d	ENSMUSG00000046808	chr5	72594568	72690014	+	7848	4.417	1
Lrrc10b	ENSMUSG00000090291	chr19	10530579	10531937	-	1359	4.417	1
Rab32	ENSMUSG00000019832	chr10	10264809	10278031	-	2108	4.415	1
Plau	ENSMUSG00000021822	chr14	21655884	21662610	+	2343	4.41	1
Shank3	ENSMUSG00000022623	chr15	89330054	89390692	+	8896	4.41	1
Dab2	ENSMUSG00000022150	chr15	6249788	6390712	+	7342	4.408	1
Acvr1	ENSMUSG00000026836	chr2	58241055	58419568	-	4829	4.408	1
Vcam1	ENSMUSG00000027962	chr3	115812938	115832606	-	4917	4.406	1
Zswim6	ENSMUSG00000032846	chr13	108514698	108680258	-	5457	4.395	1
Nedd9	ENSMUSG00000021365	chr13	41405285	41582689	-	4724	4.392	1

Pla2g4a	ENSMUSG00000056220	chr1	151676748	151808420	-		6388	4.392	1
Mastl	ENSMUSG00000026779	chr2	22971126	23011544	-		5777	4.391	1
Tet2	ENSMUSG00000040943	chr3	133126643	133207354	-		9203	4.387	1
Slc39a10	ENSMUSG00000025986	chr1	46864389	46910891	-		5959	4.377	1
Ammecr1	ENSMUSG00000042225	chrX	139285689	139401271	-		5437	4.376	1
Ttc37	ENSMUSG00000033991	chr13	76236182	76327764	+		7283	4.371	1
Tmem161b	ENSMUSG00000035762	chr13	84361901	84435571	+		2708	4.369	1
Ppp1r3b	ENSMUSG00000046794	chr8	36438795	36451178	+		4206	4.369	1
Ahsg	ENSMUSG00000022868	chr16	22892115	22899511	+		1474	4.369	1
Ccnd1	ENSMUSG00000070348	chr7	152115836	152125774	-		3835	4.367	1
Hmga1-rs1	ENSMUSG00000078249	chr11	120624108	120625733	+		1626	4.366	1
Spag9	ENSMUSG00000020859	chr11	93857405	93987399	+		11197	4.365	1
Rasa2	ENSMUSG00000032413	chr9	96439719	96532036	-		8885	4.365	1
Wipf1	ENSMUSG00000075284	chr2	73267667	73367791	-		5896	4.362	1
Hbegf	ENSMUSG00000024486	chr18	36664583	36675459	-		2373	4.356	1
Gpr161	ENSMUSG00000040836	chr1	167232756	167253061	+		3045	4.355	1
Map3k2	ENSMUSG00000024383	chr18	32322743	32396405	+		10791	4.35	1
Alkbh8	ENSMUSG00000025899	chr9	3335478	3387050	+		3659	4.349	1
Spred3	ENSMUSG00000037239	chr7	29943848	29954860	-		5997	4.347	1
Tead4	ENSMUSG00000030353	chr6	128174306	128250841	-		3596	4.347	1
Vegfa	ENSMUSG00000023951	chr17	46153942	46169326	-		4293	4.346	1
S1pr1	ENSMUSG00000045092	chr3	115413351	115417973	-		3029	4.345	1
Afap1	ENSMUSG00000029094	chr5	36235968	36346572	+		8543	4.342	1
Plod2	ENSMUSG00000032374	chr9	92437061	92503266	+		4179	4.337	1
Bach1	ENSMUSG00000025612	chr16	87699190	87733591	+		6179	4.332	1
Sdcbp2	ENSMUSG00000027456	chr2	151398358	151415741	+		1399	4.329	1
Pld1	ENSMUSG00000027695	chr3	27837602	28032284	+		8553	4.329	1
Schip1	ENSMUSG00000027777	chr3	67868864	68430404	+		2564	4.328	1
Zfp770	ENSMUSG00000040321	chr2	114019197	114027205	-		6793	4.328	1
Npc1	ENSMUSG00000024413	chr18	12348202	12394909	-		10725	4.325	1
Car9	ENSMUSG00000028463	chr4	43519838	43526601	+		2304	4.325	1
Aurkb	ENSMUSG00000020897	chr11	68859149	68865166	+		2946	4.319	1
Brca1	ENSMUSG00000017146	chr11	101350078	101413269	-		8476	4.319	1
Fnip1	ENSMUSG00000035992	chr11	54251701	54331737	+		7119	4.319	1
Tgfa	ENSMUSG00000029999	chr6	86145420	86225151	+		3762	4.315	1
Nt5e	ENSMUSG00000032420	chr9	88222447	88266927	+		3580	4.314	1
Rap2a	ENSMUSG00000051615	chr14	120877666	120906416	+		4191	4.312	1
Cast	ENSMUSG00000021585	chr13	74831691	74945958	-		3340	4.312	1
Fn1	ENSMUSG00000026193	chr1	71632094	71699774	-		8344	4.299	1
C3ar1	ENSMUSG00000040552	chr6	122797156	122806179	-		4305	4.294	1
Prc1	ENSMUSG00000038943	chr7	87439336	87461145	+		8225	4.292	1
Pign	ENSMUSG00000056536	chr1	107417716	107555585	-		4322	4.292	1

Ccdc132	ENSMUSG00000001376	chr6	3448382	3553319	+	9936	4.281	1
Kcne3	ENSMUSG00000035165	chr7	107327207	107333374	+	1314	4.281	1
Chd1	ENSMUSG00000023852	chr17	15841931	15909574	+	8721	4.28	1
Col4a1	ENSMUSG00000031502	chr8	11198423	11312826	-	6818	4.28	1
Tnfaip8	ENSMUSG00000062210	chr18	50139081	50266827	+	4400	4.271	1
Bnip2	ENSMUSG00000011958	chr9	69837273	69856124	+	3455	4.269	1
5830418K08Rik	ENSMUSG00000046111	chr9	15121359	15162232	-	11641	4.261	1
Setx	ENSMUSG00000043535	chr2	28979701	29037991	+	11380	4.254	1
Nfe2l2	ENSMUSG00000015839	chr2	75513570	75542698	-	3796	4.254	1
Smarcad1	ENSMUSG00000029920	chr6	64992661	65066044	+	6521	4.254	1
Skil	ENSMUSG00000027660	chr3	30993980	31021499	+	9694	4.252	1
Zfhx4	ENSMUSG00000025255	chr3	5218526	5415855	+	15803	4.252	1
Spsb1	ENSMUSG00000039911	chr4	149270392	149329152	-	4396	4.25	1
Sulf1	ENSMUSG00000016918	chr1	12708626	12850452	+	4608	4.248	1
Dst	ENSMUSG00000026131	chr1	33965125	34365495	+	29858	4.248	1
Rnaseh2b	ENSMUSG00000021932	chr14	62911426	62991829	+	6243	4.248	1
Stag1	ENSMUSG00000037286	chr9	100498217	100858964	+	10266	4.248	1
Anxa8	ENSMUSG00000021950	chr14	34899167	34913757	+	1880	4.247	1
Upp1	ENSMUSG00000020407	chr11	9018106	9036173	+	1961	4.246	1
Kif20a	ENSMUSG00000003779	chr18	34784278	34792919	+	3893	4.241	1
Ankrd33b	ENSMUSG00000022237	chr15	31221234	31297514	-	8706	4.24	1
Sik2	ENSMUSG00000037112	chr9	50700906	50817178	-	9539	4.24	1
Spata6	ENSMUSG00000034401	chr4	111392589	111501789	+	5616	4.237	1
Nrarp	ENSMUSG00000078202	chr2	25036278	25038850	+	2573	4.235	1
Nedd1	ENSMUSG00000019988	chr10	92147491	92185165	-	3534	4.233	1
Cntln	ENSMUSG00000038070	chr4	84530213	84777825	+	7102	4.233	1
Agfg1	ENSMUSG00000026159	chr1	82836058	82892850	+	3143	4.232	1
Rasal2	ENSMUSG00000070565	chr1	159065314	159342726	-	13739	4.23	1
6230409E13Rik	ENSMUSG00000028246	chr4	21858476	21923986	+	8362	4.23	1
Nek2	ENSMUSG00000026622	chr1	193645323	193656929	+	4516	4.23	1
Arhgef2	ENSMUSG00000028059	chr3	88411376	88451974	+	9496	4.225	1
Kif5c	ENSMUSG00000026764	chr2	49474818	49630298	+	7142	4.224	1
Fosl2	ENSMUSG00000029135	chr5	32438845	32460204	+	5855	4.222	1
Phtf2	ENSMUSG00000039987	chr5	20264481	20387942	-	6929	4.221	1
Hmga1	ENSMUSG00000046711	chr17	27693565	27700619	+	1990	4.217	1
Zbtb6	ENSMUSG00000066798	chr2	37281020	37298691	-	5080	4.216	1
Slc4a8	ENSMUSG00000023032	chr15	100592178	100654399	+	13021	4.215	1
Osm	ENSMUSG00000058755	chr11	4136423	4141029	+	2272	4.214	1
Cyp1b1	ENSMUSG00000024087	chr17	80106234	80114401	-	5207	4.214	1
Zfp53	ENSMUSG00000057409	chr17	21628220	21646629	+	1959	4.213	1

Adamts5	ENSMUSG00000022894	chr16	85858415	85901370	-	8091	4.213	1
BC024479	ENSMUSG00000042138	chr9	37296906	37331733	+	2289	4.212	1
Ddah1	ENSMUSG00000028194	chr3	145421639	145557241	+	3983	4.212	1
Itgav	ENSMUSG00000027087	chr2	83564554	83647073	+	10744	4.2	1
P2ry13	ENSMUSG00000036362	chr3	59011818	59014804	-	2524	4.199	1
Mmp19	ENSMUSG00000025355	chr10	128228009	128237880	+	3410	4.196	1
Kif2a	ENSMUSG00000021693	chr13	107749076	107812206	-	7046	4.194	1
Rdh10	ENSMUSG00000025921	chr1	16095855	16123815	+	4160	4.192	1
Pds5a	ENSMUSG00000029202	chr5	66006499	66089095	-	4160	4.189	1
Gch1	ENSMUSG00000037580	chr14	47773570	47809088	-	2775	4.189	1
Lrp1	ENSMUSG00000040249	chr10	126975217	127058204	-	17717	4.188	1
6430598A04Rik	ENSMUSG00000045348	chr5	138172111	138182835	-	4260	4.182	1
Npnt	ENSMUSG00000040998	chr3	132544709	132613255	-	5026	4.181	1
Fbn1	ENSMUSG00000027204	chr2	125126330	125333729	-	12128	4.18	1
Stard13	ENSMUSG00000016128	chr5	151840203	152036411	-	8044	4.178	1
Cul3	ENSMUSG00000004364	chr1	80261498	80337055	-	4873	4.173	1
Plxdc2	ENSMUSG00000026748	chr2	16277931	16677466	+	6947	4.173	1
Ccdc90b	ENSMUSG00000030613	chr7	99709659	99730804	+	1801	4.172	1
Flrt2	ENSMUSG00000047414	chr12	96930436	97023425	+	7721	4.172	1
Spred2	ENSMUSG00000045671	chr11	19824378	19924029	+	4619	4.171	1
2210403K04Rik	ENSMUSG00000085148	chr11	75275041	75280178	+	1924	4.17	1
Dpysl3	ENSMUSG00000024501	chr18	43480633	43597940	-	6265	4.168	1
Bmi1	ENSMUSG00000026739	chr2	18598645	18608256	+	3974	4.168	1
Xpr1	ENSMUSG00000026469	chr1	157122831	157264545	-	12695	4.166	1
Tmtc4	ENSMUSG00000041594	chr14	123318193	123383257	-	7367	4.165	1
Fblim1	ENSMUSG00000006219	chr4	141131977	141162011	-	4457	4.16	1
Acvr2a	ENSMUSG00000052155	chr2	48669629	48758789	+	5686	4.16	1
Cep192	ENSMUSG00000024542	chr18	67959761	68044822	+	8096	4.159	1
9830001H06Rik	ENSMUSG00000055485	chr2	156841535	156904990	-	7984	4.159	1
Hspa4l	ENSMUSG00000025757	chr3	40548417	40600025	+	11792	4.157	1
Rictor	ENSMUSG00000050310	chr15	6658381	6750398	+	9311	4.156	1
Dpy19l1	ENSMUSG00000043067	chr9	24216220	24307584	-	6350	4.154	1
9030625A04Rik	ENSMUSG00000044350	chr14	77424007	77436711	-	3644	4.152	1
Nup107	ENSMUSG00000052798	chr10	117187680	117229761	-	3111	4.151	1
Cbfb	ENSMUSG00000031885	chr8	107694574	107741886	+	2911	4.15	1
Bin2	ENSMUSG00000075411	chr15	100475214	100499933	-	1476	4.15	1

9030418K01Rik	ENSMUSG00000070476	chr2	178149229	178159133	+	4559	4.144	1
Cblb	ENSMUSG00000022637	chr16	52031662	52208160	+	6573	4.141	1
Smc4	ENSMUSG00000034349	chr3	68808660	68838545	+	5032	4.14	1
Ttc39b	ENSMUSG00000038172	chr4	82866204	82970159	-	10199	4.137	1
Hpx	ENSMUSG00000030895	chr7	112740127	112748630	-	1478	4.132	1
Flt1	ENSMUSG00000029648	chr5	148373180	148537587	-	11583	4.132	1
Cxcl2	ENSMUSG00000058427	chr5	91332897	91334935	+	1830	4.131	1
Amica1	ENSMUSG00000048534	chr9	44887266	44916613	+	2457	4.131	1
Dnm3os	ENSMUSG00000078190	chr1	164147754	164155681	+	7928	4.129	1
S100a9	ENSMUSG00000056071	chr3	90496554	90499643	-	522	4.128	1
Psd3	ENSMUSG00000030465	chr8	70212981	70586185	-	15325	4.125	1
Coq10b	ENSMUSG00000025981	chr1	55109614	55129545	+	1732	4.122	1
Lrch3	ENSMUSG00000022801	chr16	32914186	33015733	+	6610	4.121	1
C5ar1	ENSMUSG00000049130	chr7	16832092	16844889	-	2570	4.12	1
Syn1	ENSMUSG00000037217	chrX	20437637	20498130	-	3299	4.119	1
Itpripl2	ENSMUSG00000073859	chr7	125628628	125629997	-	1370	4.118	1
Slfn9	ENSMUSG00000069793	chr11	82793131	82805332	-	4726	4.116	1
Gfod1	ENSMUSG00000051335	chr13	43290888	43399541	-	6654	4.116	1
Als2cl	ENSMUSG00000044037	chr9	110782374	110803034	+	5848	4.115	1
Kctd11	ENSMUSG00000046731	chr11	69691767	69694487	-	2721	4.114	1
Zfp62	ENSMUSG00000046311	chr11	49016794	49032318	+	6037	4.111	1
Dennd4a	ENSMUSG00000053641	chr9	64659147	64767474	+	10545	4.107	1
Abi1	ENSMUSG00000058835	chr2	22795593	22895761	-	4017	4.106	1
Syt8	ENSMUSG00000031098	chr7	149620755	149626301	+	1894	4.104	1
Dusp18	ENSMUSG00000047205	chr11	3795243	3801299	+	4632	4.104	1
Ctsk	ENSMUSG00000028111	chr3	95303208	95313284	+	1482	4.101	1
Serpinb9	ENSMUSG00000045827	chr13	33095119	33109826	+	3494	4.094	1
Flnb	ENSMUSG00000025278	chr14	8650471	8784102	+	9100	4.093	1
Elf4	ENSMUSG00000031103	chrX	45764223	45816309	-	6148	4.092	1
Lyst	ENSMUSG00000019726	chr13	13682676	13869710	+	11814	4.087	1
Pdk4	ENSMUSG00000019577	chr6	5433351	5446309	-	3511	4.085	1
Kcnf1	ENSMUSG00000051726	chr12	17178908	17183694	-	4787	4.084	1
Nid2	ENSMUSG00000021806	chr14	20570479	20631009	+	4884	4.083	1
Akap7	ENSMUSG00000039166	chr10	24888896	25027676	-	4375	4.077	1
Pcsk9	ENSMUSG00000044254	chr4	106114934	106136934	-	3521	4.076	1
Dram2	ENSMUSG00000027900	chr3	106350611	106377763	+	2818	4.073	1
Ear2	ENSMUSG00000072596	chr14	44680319	44681199	+	719	4.066	1
Stk3	ENSMUSG00000022329	chr15	34805251	35085561	-	3237	4.06	1
Fgd6	ENSMUSG00000020021	chr10	93498746	93608084	+	8241	4.054	1
Myadm	ENSMUSG00000068566	chr7	3289039	3299346	+	3238	4.053	1
Zfp658	ENSMUSG00000056592	chr7	50817626	50830831	+	3495	4.052	1
Mcpt8	ENSMUSG00000022157	chr14	56701003	56704053	-	855	4.052	1
Igsf6	ENSMUSG00000035004	chr7	128207581	128218044	-	2133	4.05	1

Cnot6	ENSMUSG00000020362	chr11	49485005	49526225	-	6610	4.05	1
Zfp36l2	ENSMUSG00000045817	chr17	84583271	84587287	-	3530	4.044	1
Wwtr1	ENSMUSG00000027803	chr3	57259571	57379832	-	4779	4.042	1
Slc16a1	ENSMUSG00000032902	chr3	104442591	104462385	+	4426	4.04	1
Pdpn	ENSMUSG00000028583	chr4	142857334	142889467	-	1817	4.038	1
Gnb4	ENSMUSG00000027669	chr3	32479254	32515507	-	12678	4.038	1
Cit	ENSMUSG00000029516	chr5	116295287	116458956	+	15387	4.037	1
Layn	ENSMUSG00000060594	chr9	50862745	50885199	-	2246	4.037	1
Klhl28	ENSMUSG00000020948	chr12	66043427	66066523	-	2854	4.033	1
Prrx1	ENSMUSG00000026586	chr1	165175247	165243841	-	11069	4.032	1
Zfp871	ENSMUSG00000024298	chr17	32908181	32925232	-	5376	4.032	1
Syt5	ENSMUSG00000004961	chr7	4491370	4498169	-	1747	4.027	1
Thoc1	ENSMUSG00000024287	chr18	9958178	9995482	+	3855	4.024	1
Angptl2	ENSMUSG00000004105	chr2	33071589	33103237	+	6286	4.023	1
Cd93	ENSMUSG00000027435	chr2	148262376	148269299	-	6677	4.023	1
Prdm8	ENSMUSG00000035456	chr5	98609888	98616467	+	3119	4.021	1
Gm17296	ENSMUSG00000090290	chr8	128950552	128998965	-	5060	4.021	1
Gbp9	ENSMUSG00000029298	chr5	105507413	105539311	-	3633	4.02	1
Itsn2	ENSMUSG00000020640	chr12	4599814	4720756	+	6267	4.019	1
Itga5	ENSMUSG00000000555	chr15	103174720	103197194	-	4379	4.018	1
Mtap1b	ENSMUSG00000052727	chr13	100191419	100286557	-	12069	4.017	1
Epdr1	ENSMUSG00000002808	chr13	19683578	19711699	-	2386	4.01	1
Piga	ENSMUSG00000031381	chrX	160857725	160871848	+	6379	4.009	1
Stam	ENSMUSG00000026718	chr2	13995727	14069965	+	5353	4.009	1
Mospd2	ENSMUSG00000061778	chrX	161374101	161418307	-	7914	4.007	1
Clec4n	ENSMUSG00000023349	chr6	123179861	123197039	+	1420	4.007	1
Zfp518b	ENSMUSG00000046572	chr5	39059719	39075989	-	6810	4.006	1
Atad2	ENSMUSG00000022360	chr15	57925602	57966637	-	5700	4.006	1
Osmr	ENSMUSG00000022146	chr15	6763577	6824969	-	12113	4.004	1
Kirrel	ENSMUSG00000041734	chr3	86882515	86978669	-	7463	4.001	1
Elovl6	ENSMUSG00000041220	chr3	129235304	129341413	+	5951	4	1
Sdccag8	ENSMUSG00000026504	chr1	178744791	178950568	+	4129	3.998	1
Serinc1	ENSMUSG00000019877	chr10	57235580	57252336	-	5150	3.995	1
Actr2	ENSMUSG00000020152	chr11	19962307	20012916	-	5204	3.993	1
Pcdh18	ENSMUSG00000037892	chr3	49547218	49561238	-	5168	3.992	1
Tmem65	ENSMUSG00000062373	chr15	58613824	58655085	-	3747	3.992	1
Pik3r5	ENSMUSG00000020901	chr11	68245623	68311351	+	5550	3.99	1
Aldob	ENSMUSG00000028307	chr4	49548867	49562418	-	2392	3.988	1
Dock9	ENSMUSG00000025558	chr14	121941261	122196956	-	8930	3.988	1
Pibf1	ENSMUSG00000022064	chr14	99498652	99653712	+	3293	3.987	1
Arhgap6	ENSMUSG00000031355	chrX	165233031	165742367	+	12143	3.985	1
Mtap	ENSMUSG00000062937	chr4	88783026	88826985	+	3226	3.984	1

Csnk1g3	ENSMUSG00000073563	chr18	54021767	54115338	+	4394	3.98	1
Ppp3cc	ENSMUSG00000022092	chr14	70617705	70689257	-	2931	3.979	1
Trip12	ENSMUSG00000026219	chr1	84717764	84835879	-	9632	3.978	1
Pdlim1	ENSMUSG00000055044	chr19	40296731	40346106	-	1503	3.977	1
Rnf152	ENSMUSG00000047496	chr1	107173494	107253287	-	8529	3.976	1
Slfn5	ENSMUSG00000054404	chr11	82764851	82776443	+	4427	3.975	1
Agps	ENSMUSG00000042410	chr2	75670234	75769407	+	9421	3.972	1
Spred1	ENSMUSG00000027351	chr2	116947110	117008015	+	6784	3.969	1
Fam49b	ENSMUSG00000022378	chr15	63760652	63892010	-	3869	3.968	1
Nup210l	ENSMUSG00000027939	chr3	89908054	90015939	+	5681	3.968	1
Pif1	ENSMUSG00000041064	chr9	65434967	65443774	+	4388	3.967	1
Egr1	ENSMUSG00000038418	chr18	35019477	35024638	+	4484	3.963	1
Gprc5b	ENSMUSG00000008734	chr7	126115554	126138725	-	4525	3.962	1
Itgax	ENSMUSG00000030789	chr7	135273061	135294171	+	4046	3.962	1
Csf2rb	ENSMUSG00000071713	chr15	78156420	78184277	+	5069	3.961	1
Parp8	ENSMUSG00000021725	chr13	117643631	117814323	-	3088	3.96	1
Mms22l	ENSMUSG00000045751	chr4	24423598	24530097	+	6382	3.959	1
Vash1	ENSMUSG00000021256	chr12	88019650	88036630	+	4956	3.957	1
Cd109	ENSMUSG00000046186	chr9	78463353	78564060	+	6900	3.957	1
Pik3c2a	ENSMUSG00000030660	chr7	123480790	123586972	-	8042	3.95	1
Gm10516	ENSMUSG00000073476	chr1	193960780	193974895	+	2600	3.945	1
Adamts9	ENSMUSG00000030022	chr6	92722693	92893486	-	12527	3.945	1
Trio	ENSMUSG00000022263	chr15	27660407	27955603	-	12368	3.945	1
Nras	ENSMUSG00000027852	chr3	102862152	102871837	+	4624	3.942	1
Top2a	ENSMUSG00000020914	chr11	98854257	98885503	-	9070	3.941	1
Slc16a3	ENSMUSG00000025161	chr11	120809794	120822182	+	5318	3.939	1
Rab27a	ENSMUSG00000032202	chr9	72892672	72945399	+	2971	3.938	1
Lnp	ENSMUSG00000009207	chr2	74358348	74417492	-	9154	3.936	1
Rasgrp3	ENSMUSG00000071042	chr17	75835245	75928383	+	4925	3.936	1
Usp45	ENSMUSG00000040455	chr4	21694303	21765019	+	6226	3.934	1
Acap2	ENSMUSG00000049076	chr16	31092499	31201324	-	6514	3.934	1
D0H4S114	ENSMUSG00000042834	chr18	33596673	33623683	-	2513	3.931	1
Ccnyl1	ENSMUSG00000070871	chr1	64737029	64772218	+	3281	3.931	1
Itgb3	ENSMUSG00000020689	chr11	104469314	104531790	+	5901	3.928	1
Asf1a	ENSMUSG00000019857	chr10	53316767	53329019	+	2180	3.919	1
Pdgfrl	ENSMUSG00000031595	chr8	42011566	42076139	+	3126	3.919	1
Hip1	ENSMUSG00000039959	chr5	135882393	136020992	-	7850	3.916	1
Ccng2	ENSMUSG00000029385	chr5	93696283	93705257	+	5458	3.914	1
Fkbp14	ENSMUSG00000038074	chr6	54527598	54547338	-	4185	3.913	1

8430406I07Rik	ENSMUSG00000027424	chr2	144096399	144106963	+		3348	3.913	1
Alcam	ENSMUSG00000022636	chr16	52249109	52454187	-		7121	3.911	1
Cd36	ENSMUSG00000002944	chr5	17287508	17394777	-		3927	3.908	1
Zfp397	ENSMUSG00000024276	chr18	24113189	24123172	+		5487	3.903	1
Klhl11	ENSMUSG00000048732	chr11	100323928	100334055	-		2393	3.902	1
Chml	ENSMUSG00000078185	chr1	177616619	177618484	-		1866	3.902	1
Gzmc	ENSMUSG00000079186	chr14	56850238	56853493	-		934	3.902	1
Mtmr6	ENSMUSG00000021987	chr14	60884065	60921207	+		3813	3.892	1
Cdca5	ENSMUSG00000024791	chr19	6084983	6091777	+		2129	3.891	1
Stx2	ENSMUSG00000029428	chr5	129490432	129514449	-		4164	3.889	1
Pola1	ENSMUSG00000006678	chrX	90550106	90877494	-		8813	3.887	1
Rgs16	ENSMUSG00000026475	chr1	155587479	155592598	+		2339	3.886	1
Col12a1	ENSMUSG00000032332	chr9	79446798	79566638	-		15228	3.883	1
Slc19a2	ENSMUSG00000040918	chr1	166179177	166195516	+		3838	3.881	1
Gm17379	ENSMUSG00000090695	chrX	166423343	166423549	-		42	3.877	1
2210009G21Rik	ENSMUSG00000027281	chr2	136716954	136897686	+		5905	3.877	1
Tiparp	ENSMUSG00000034640	chr3	65332332	65359067	+		3985	3.873	1
Dis3	ENSMUSG00000033166	chr14	99475853	99498989	-		3722	3.87	1
Rasa1	ENSMUSG00000021549	chr13	85354304	85429091	-		7834	3.867	1
Tmem68	ENSMUSG00000028232	chr4	3476188	3502000	-		3462	3.864	1
Msh3	ENSMUSG00000014850	chr13	92981836	93124958	-		7456	3.862	1
Runx3	ENSMUSG00000070691	chr4	134676567	134733905	+		5003	3.861	1
Pde4a	ENSMUSG00000032177	chr9	20970158	21017692	+		7655	3.861	1
Smg1	ENSMUSG00000030655	chr7	125274832	125387151	-		15547	3.859	1
Med14	ENSMUSG00000064127	chrX	12252495	12339199	-		8876	3.858	1
Rufy2	ENSMUSG00000020070	chr10	62442971	62481490	+		12112	3.857	1
Parp2	ENSMUSG00000036023	chr14	51427621	51440975	+		1788	3.856	1
Abca1	ENSMUSG00000015243	chr4	53043659	53172767	-		10363	3.854	1
Zfp365	ENSMUSG00000037855	chr10	67348851	67375410	-		5205	3.853	1
Dbf4	ENSMUSG00000002297	chr5	8396973	8422716	-		6571	3.852	1
Akr1c13	ENSMUSG00000021213	chr13	4190430	4204842	+		1400	3.85	1
Map3k1	ENSMUSG00000021754	chr13	112536636	112599201	-		7447	3.848	1
Slc16a6	ENSMUSG00000041920	chr11	109312169	109334912	-		4883	3.844	1
Rnft1	ENSMUSG00000020521	chr11	86298159	86312527	+		4719	3.844	1
Cd276	ENSMUSG00000035914	chr9	58372107	58402939	-		3297	3.844	1
Fyn	ENSMUSG00000019843	chr10	39088661	39285187	+		4363	3.842	1
Ccnb2	ENSMUSG00000032218	chr9	70255496	70269361	-		1541	3.84	1
AI597468	ENSMUSG00000060935	chr10	84565372	84580492	+		3778	3.84	1

1300014 I06Rik	ENSMUSG00000021411	chr13	34719709	34744550	-	2103	3.84	1
Lig4	ENSMUSG00000049717	chr8	9970020	9977686	-	4255	3.84	1
Adamts 14	ENSMUSG00000059901	chr10	60659860	60736186	-	5197	3.836	1
Scyl2	ENSMUSG00000069539	chr10	89101466	89149030	-	10099	3.833	1
F63004 3A04Rik	ENSMUSG00000021965	chr14	58425398	58445000	-	2260	3.833	1
Epb4.1l3	ENSMUSG00000024044	chr17	69506150	69639325	+	5838	3.833	1
Nov	ENSMUSG00000037362	chr15	54577257	54585594	+	2869	3.833	1
Unc13c	ENSMUSG00000062151	chr9	73328677	73781374	-	6633	3.833	1
Ppip5k2	ENSMUSG00000040648	chr1	99602625	99666988	-	11816	3.827	1
Cdk17	ENSMUSG00000020015	chr10	92623621	92729816	+	5450	3.826	1
Mlkl	ENSMUSG00000012519	chr8	113835697	113862077	-	2303	3.826	1
Nucb2	ENSMUSG00000030659	chr7	123647888	123684098	+	1680	3.825	1
Pfkp	ENSMUSG00000021196	chr13	6579014	6648023	-	9395	3.823	1
Kit	ENSMUSG00000005672	chr5	75970941	76052747	+	5831	3.819	1
Jmjd1c	ENSMUSG00000037876	chr10	66558873	66719074	+	14397	3.817	1
Plk4	ENSMUSG00000025758	chr3	40603941	40620805	+	5863	3.813	1
Gm885	ENSMUSG00000040528	chr11	106612517	106630108	+	2307	3.811	1
Il7r	ENSMUSG00000003882	chr15	9435916	9459631	-	3208	3.81	1
Inpp5d	ENSMUSG00000026288	chr1	89516887	89617082	+	7602	3.809	1
Myo1e	ENSMUSG00000032220	chr9	70055157	70247874	+	4963	3.807	1
Dzip1l	ENSMUSG00000037784	chr9	99529915	99569675	+	4655	3.807	1
Nav1	ENSMUSG00000009418	chr1	137331157	137481932	-	12767	3.807	1
Arl4c	ENSMUSG00000049866	chr1	90569700	90598796	-	5492	3.806	1
Adamts 6	ENSMUSG00000046169	chr13	105077953	105284843	+	5022	3.805	1
Clcn5	ENSMUSG00000004317	chrX	6730936	6896484	-	11452	3.804	1
Ncs1	ENSMUSG00000062661	chr2	31101343	31151509	+	4653	3.802	1
Sec23a	ENSMUSG00000020986	chr12	60059370	60113004	-	4840	3.801	1
Usp9x	ENSMUSG00000031010	chrX	12648624	12759008	+	17363	3.798	1
Thnsl1	ENSMUSG00000048550	chr2	21127351	21136636	+	4043	3.797	1
Ptprz1	ENSMUSG00000068748	chr6	22825502	23002916	+	8068	3.789	1
Fndc1	ENSMUSG00000071984	chr17	7931434	8020167	-	9903	3.788	1
Cdc14b	ENSMUSG00000033102	chr13	64293853	64376281	-	6083	3.787	1
Esyt2	ENSMUSG00000021171	chr12	117519695	117611569	+	5539	3.787	1
Crlf1	ENSMUSG00000007888	chr8	73017057	73027980	+	1644	3.786	1
Fam82a 1	ENSMUSG00000036368	chr17	80014240	80081492	+	1802	3.784	1
Zbtb33	ENSMUSG00000048047	chrX	35542970	35550223	+	5122	3.784	1
Stac	ENSMUSG00000032502	chr9	111463941	111592852	-	2644	3.783	1
Ube2c	ENSMUSG00000001403	chr2	164595398	164604322	+	2600	3.783	1
Zfp868	ENSMUSG00000060427	chr8	72134756	72149447	-	2893	3.782	1
Rhou	ENSMUSG00000039960	chr8	126177829	126187784	+	3806	3.781	1

Tlr4	ENSMUSG00000039005	chr4	66488618	66591318	+	5556	3.78	1
Ntn4	ENSMUSG00000020019	chr10	93103426	93209952	+	3741	3.779	1
St3gal5	ENSMUSG00000056091	chr6	72047607	72104564	+	2448	3.778	1
Slc27a6	ENSMUSG00000024600	chr18	58715911	58772427	+	2485	3.776	1
Creb5	ENSMUSG00000053007	chr6	53523378	53646001	+	3038	3.776	1
Mast4	ENSMUSG00000034751	chr13	103522566	104124577	-	20337	3.771	1
Unc13d	ENSMUSG00000057948	chr11	115923409	115939275	-	4679	3.765	1
Gdf11	ENSMUSG00000025352	chr10	128319351	128328774	-	4062	3.761	1
Socs5	ENSMUSG00000037104	chr17	87507019	87536923	+	4392	3.761	1
Fam135a	ENSMUSG00000026153	chr1	24017597	24107180	-	6468	3.759	1
Pvr	ENSMUSG00000040511	chr7	20488927	20506509	-	5408	3.758	1
Ankrd1	ENSMUSG00000024803	chr19	36186455	36194334	-	1765	3.756	1
Nup54	ENSMUSG00000034826	chr5	92844566	92864245	-	2867	3.755	1
Herc4	ENSMUSG00000020064	chr10	62706659	62780622	+	3922	3.752	1
Tpp2	ENSMUSG00000041763	chr1	43990847	44059844	+	4682	3.752	1
Tfpi	ENSMUSG00000027082	chr2	84273012	84316932	-	5463	3.751	1
Il4ra	ENSMUSG00000030748	chr7	132695785	132722986	+	5125	3.75	1
2610029I01Rik	ENSMUSG00000028251	chr4	21684529	21694359	-	1588	3.748	1
Hsd17b7	ENSMUSG00000026675	chr1	171879666	171899372	-	5104	3.746	1
Nusap1	ENSMUSG00000027306	chr2	119444034	119476980	+	4059	3.744	1
Trmt11	ENSMUSG00000019792	chr10	30254031	30320555	-	2359	3.743	1
Bmpr2	ENSMUSG00000067336	chr1	59821481	59927703	+	3507	3.74	1
Smchd1	ENSMUSG00000024054	chr17	71693829	71824683	-	7581	3.74	1
Nupr1	ENSMUSG00000030717	chr7	133766763	133770524	-	2452	3.74	1
Casp4	ENSMUSG00000033538	chr9	5308828	5336783	+	3710	3.735	1
Aff4	ENSMUSG00000049470	chr11	53164335	53235332	+	13450	3.735	1
Ctgf	ENSMUSG00000019997	chr10	24315248	24318489	+	2861	3.731	1
Foxn2	ENSMUSG00000034998	chr17	88840051	88889873	+	7357	3.731	1
Cdh11	ENSMUSG00000031673	chr8	105156895	105309011	-	4216	3.731	1
Phlda1	ENSMUSG00000020205	chr10	110943342	110945705	+	1953	3.73	1
Tmem209	ENSMUSG00000029782	chr6	30429053	30459783	-	8132	3.73	1
Camsap2	ENSMUSG00000041570	chr1	138164700	138242681	-	7883	3.729	1
Melk	ENSMUSG00000035683	chr4	44313748	44377547	+	2972	3.728	1
Mgst2	ENSMUSG00000074604	chr3	51464282	51486597	+	3659	3.724	1
Gopc	ENSMUSG00000019861	chr10	52056891	52101881	-	4207	3.724	1
Ankib1	ENSMUSG00000040351	chr5	3690000	3803109	-	6610	3.724	1
Picalm	ENSMUSG00000039361	chr7	97278723	97357610	+	3846	3.72	1
Pdzd8	ENSMUSG00000074746	chr19	59370574	59420270	-	7076	3.719	1
Rps6ka3	ENSMUSG00000031309	chrX	155648239	155806176	+	8322	3.719	1
D16Ertd472e	ENSMUSG00000022864	chr16	78544257	78576902	-	2453	3.718	1

Lrrfip1	ENSMUSG00000026305	chr1	92895304	93025521	+	5848	3.716		1
Haus2	ENSMUSG00000027285	chr2	120435119	120447296	+	3218	3.714		1
Kctd9	ENSMUSG00000034327	chr14	68333994	68360367	+	4746	3.711		1
Slc22a15	ENSMUSG00000033147	chr3	101659701	101728376	-	6725	3.71		1
Klf10	ENSMUSG00000037465	chr15	38221219	38230464	-	3823	3.71		1
Gpr77	ENSMUSG00000074361	chr7	16819934	16829503	-	3561	3.705		1
Slc39a6	ENSMUSG00000024270	chr18	24738382	24762318	-	4073	3.704		1
S1pr3	ENSMUSG00000067586	chr13	51503987	51518166	+	4484	3.704		1
Rock2	ENSMUSG00000020580	chr12	16901784	16995080	+	8195	3.703		1
Zeb2	ENSMUSG00000026872	chr2	44839154	44972915	-	19659	3.701		1
Pvrl3	ENSMUSG00000022656	chr16	46387819	46498638	-	12747	3.7		1
1700025G04Rik	ENSMUSG00000032666	chr1	153731654	153937450	-	9858	3.699		1
Phf16	ENSMUSG00000037315	chrX	20002814	20097065	+	5084	3.697		1
Mphosph10	ENSMUSG00000030521	chr7	71521427	71537154	-	3686	3.697		1
Tmx4	ENSMUSG00000034723	chr2	134419921	134469881	-	10907	3.696		1
St3gal1	ENSMUSG00000013846	chr15	66934437	66945922	-	5353	3.694		1
Wrn	ENSMUSG00000031583	chr8	34344856	34495999	-	6408	3.694		1
Lmo1	ENSMUSG00000036111	chr7	116282086	116313822	-	1071	3.689		1
Elk3	ENSMUSG00000008398	chr10	92710159	92773880	-	4550	3.687		1
Rp2h	ENSMUSG00000060090	chrX	19941607	19982779	+	5150	3.681		1
Socs6	ENSMUSG00000056153	chr18	88834616	89096873	-	8225	3.681		1
Bzw1	ENSMUSG00000051223	chr1	58449980	58463392	+	2833	3.679		1
Hmmr	ENSMUSG00000020330	chr11	40514890	40546939	-	5573	3.676		1
Ano6	ENSMUSG00000064210	chr15	95621274	95805180	+	4955	3.676		1
Clasp2	ENSMUSG00000033392	chr9	113650591	113828815	+	7415	3.669		1
Dgke	ENSMUSG00000000276	chr11	88896493	88928164	-	8387	3.669		1
Sox9	ENSMUSG00000000567	chr11	112643538	112649074	+	4135	3.667		1
Emilin2	ENSMUSG00000024053	chr17	71601514	71660896	-	4152	3.666		1
Pdlim5	ENSMUSG00000028273	chr3	141903568	142058630	-	5367	3.666		1
Acvr1c	ENSMUSG00000026834	chr2	58119864	58210306	-	9292	3.666		1
Bdkrb1	ENSMUSG00000041347	chr12	106842301	106843638	+	1338	3.666		1
Med13	ENSMUSG00000034297	chr11	86080535	86171104	-	10840	3.663		1
Rbak	ENSMUSG00000061898	chr5	143933865	143942454	-	3623	3.662		1
Afap1l1	ENSMUSG00000033032	chr18	61889915	61946356	-	3680	3.658		1
Fam129a	ENSMUSG00000026483	chr1	153418316	153569069	+	7380	3.652		1
Actr3	ENSMUSG00000026341	chr1	127289483	127332067	-	2508	3.651		1
Rian	ENSMUSG00000091793	chr12	110842155	110899919	+	8197	3.649		1
Sema3c	ENSMUSG00000028780	chr5	17080099	17236086	+	7140	3.648		1
Mbnl3	ENSMUSG00000036109	chrX	48470446	48559709	-	8733	3.647		1
Nlrc3	ENSMUSG00000049871	chr16	3946933	3976632	-	4114	3.644		1
Eaf1	ENSMUSG00000021890	chr14	32308265	32323044	+	4238	3.644		1

Rnf144a	ENSMUSG00000020642	chr12	26991662	27100121	-	5163	3.642	1
Abcg2	ENSMUSG00000029802	chr6	58534517	58642863	+	4987	3.641	1
Arl13b	ENSMUSG00000022911	chr16	62793511	62846866	-	5462	3.639	1
Efnb2	ENSMUSG00000001300	chr8	8617434	8661239	-	4790	3.639	1
Eif1a	ENSMUSG00000057561	chr18	46757358	46769879	+	2879	3.638	1
Trpm7	ENSMUSG00000027365	chr2	126617301	126701966	-	11315	3.637	1
Prkd3	ENSMUSG00000024070	chr17	79348745	79420156	-	11017	3.636	1
Tnks2	ENSMUSG00000024811	chr19	36908722	36967967	+	10406	3.632	1
Rlf	ENSMUSG00000049878	chr4	120817978	120887689	-	6990	3.631	1
Sept10	ENSMUSG00000019917	chr10	58604398	58684595	-	2645	3.631	1
Rad21	ENSMUSG00000022314	chr15	51794151	51823306	-	3604	3.63	1
Pros1	ENSMUSG00000022912	chr16	62854133	62929172	+	4088	3.627	1
Prr11	ENSMUSG00000020493	chr11	86902655	86922210	-	3824	3.626	1
1810011O10Rik	ENSMUSG00000056313	chr8	25548088	25549418	-	1331	3.621	1
Fam38b	ENSMUSG00000041482	chr18	63169867	63546837	-	13036	3.62	1
Rras2	ENSMUSG00000055723	chr7	121190299	121261295	-	2272	3.619	1
Mcm6	ENSMUSG00000026355	chr1	130228168	130256233	-	4366	3.619	1
Xpo1	ENSMUSG00000020290	chr11	23156041	23198249	+	7181	3.617	1
Zbtb43	ENSMUSG00000026788	chr2	33305807	33324079	-	5225	3.616	1
Rcor1	ENSMUSG00000037896	chr12	112277561	112354112	+	5685	3.615	1
Ttll4	ENSMUSG00000033257	chr1	74708319	74748406	+	9594	3.614	1
Mbtps2	ENSMUSG00000046873	chrX	153973303	154036647	-	10105	3.612	1
Sertad2	ENSMUSG00000049800	chr11	20443256	20553024	+	6352	3.61	1
Slc7a2	ENSMUSG00000031596	chr8	41947750	42007662	+	9135	3.61	1
Adamts7	ENSMUSG00000032363	chr9	90057816	90102909	+	6373	3.609	1
Gabpa	ENSMUSG00000008976	chr16	84835170	84864024	+	5687	3.608	1
Pax9	ENSMUSG00000001497	chr12	57792754	57813809	+	5056	3.607	1
Zfp870	ENSMUSG00000057254	chr17	33019616	33023028	-	1683	3.606	1
Elmo2	ENSMUSG00000017670	chr2	165113531	165151979	-	6652	3.602	1
Ccl12	ENSMUSG00000035352	chr11	81915347	81916902	+	537	3.602	1
Pikfyve	ENSMUSG00000025949	chr1	65233324	65320586	+	6674	3.602	1
B3gnt3	ENSMUSG00000031803	chr8	74214655	74225688	-	3055	3.601	1
Frk	ENSMUSG00000019779	chr10	34203206	34331032	+	4799	3.598	1
Lin54	ENSMUSG00000035310	chr5	100870937	100929658	-	5820	3.595	1
Notch2	ENSMUSG00000027878	chr3	97817461	97954290	+	10506	3.594	1
Eif2c2	ENSMUSG00000036698	chr15	72929022	73015270	-	10957	3.592	1
Ifrd1	ENSMUSG00000001627	chr12	40928154	40975091	-	5858	3.591	1
Rbm12	ENSMUSG00000089824	chr2	155917694	155937714	-	6689	3.591	1
Sdcbp	ENSMUSG00000028249	chr4	6292797	6335570	+	6904	3.59	1
Mdm2	ENSMUSG00000020184	chr10	117125944	117147814	-	5070	3.59	1
Mmp9	ENSMUSG00000017737	chr2	164766280	164781350	+	3539	3.587	1
Nfatc1	ENSMUSG00000033016	chr18	80802944	80909810	-	7433	3.586	1
Mcm4	ENSMUSG00000022673	chr16	15623990	15637493	-	3589	3.585	1

Ednra	ENSMUSG00000031616	chr8	80186930	80248363	-	5476	3.585		1
Exoc6	ENSMUSG00000053799	chr19	37624908	37757735	+	2729	3.582		1
Loxl3	ENSMUSG00000000693	chr6	82984167	83002556	+	4196	3.58		1
Atad2b	ENSMUSG00000052812	chr12	4924210	5050853	+	4688	3.579		1
Senp5	ENSMUSG00000022772	chr16	31962592	32003373	-	4426	3.578		1
Nt5c2	ENSMUSG00000025041	chr19	46961321	47089643	-	4475	3.578		1
Car13	ENSMUSG00000027555	chr3	14641727	14663002	+	2299	3.578		1
Camk2d	ENSMUSG00000053819	chr3	126299220	126547972	+	16069	3.575		1
Trim30a	ENSMUSG00000030921	chr7	111557539	111613707	-	7884	3.57		1
Fxyd5	ENSMUSG00000009687	chr7	31817741	31827500	-	4185	3.566		1
Arl5b	ENSMUSG00000017418	chr2	14971022	15004083	+	7230	3.562		1
Wdr3	ENSMUSG00000033285	chr3	99942103	99966330	-	5218	3.559		1
Zwilch	ENSMUSG00000032400	chr9	63984951	64020911	-	4283	3.557		1
Pde4b	ENSMUSG00000028525	chr4	101760148	102279864	+	10560	3.557		1
Slmo2	ENSMUSG00000016257	chr2	174290568	174298582	-	1860	3.555		1
Nup37	ENSMUSG00000035351	chr10	87609737	87641133	+	1362	3.554		1
Col4a2	ENSMUSG00000031503	chr8	11312805	11449287	+	6619	3.553		1
Zfp563	ENSMUSG00000067424	chr17	33226255	33243148	+	2255	3.553		1
Klhl6	ENSMUSG00000043008	chr16	19946586	19983122	-	2625	3.552		1
Naip2	ENSMUSG00000078945	chr13	100914018	100972047	-	4825	3.551		1
Zfp280d	ENSMUSG00000038535	chr9	72122706	72211577	+	4475	3.55		1
Gcfc1	ENSMUSG00000022974	chr16	91014282	91044788	-	5508	3.55		1
Gm10134	ENSMUSG00000063611	chr2	28361615	28361995	+	381	3.549		1
Ptprm	ENSMUSG00000033278	chr17	67016188	67703799	-	6211	3.549		1
Purg	ENSMUSG00000049184	chr8	34496797	34527940	+	2119	3.549		1
Agpat9	ENSMUSG00000029314	chr5	101274732	101328121	+	3502	3.548		1
Snapc1	ENSMUSG00000021113	chr12	75065517	75085807	+	2125	3.546		1
Dhx36	ENSMUSG00000027770	chr3	62273961	62310926	-	4389	3.543		1
2700029M09Rik	ENSMUSG00000038005	chr8	63369215	63386377	+	1701	3.539		1
Gjb4	ENSMUSG00000046623	chr4	127028330	127031325	-	1446	3.539		1
Tcerg1	ENSMUSG00000024498	chr18	42671141	42735439	+	4503	3.536		1
Galnt7	ENSMUSG00000031608	chr8	60002625	60131829	-	5253	3.534		1
Ncf1	ENSMUSG00000015950	chr5	134695923	134705495	-	3616	3.533		1
Raph1	ENSMUSG00000026014	chr1	60540080	60623948	-	3044	3.533		1
Apaf1	ENSMUSG00000019979	chr10	90452056	90545515	-	9028	3.532		1
Meox1	ENSMUSG00000001493	chr11	101738824	101755688	-	2228	3.532		1
A630001G21Rik	ENSMUSG00000052760	chr1	87613658	87675218	-	2719	3.532		1
Xrn1	ENSMUSG00000032410	chr9	95855179	95953446	+	5499	3.532		1
Mef2a	ENSMUSG00000030557	chr7	74376049	74517744	-	6331	3.529		1
Socs3	ENSMUSG00000053113	chr11	117827393	117831361	-	2891	3.529		1
Bcl6b	ENSMUSG00000000317	chr11	70037630	70043300	-	3719	3.527		1
Dock8	ENSMUSG00000052085	chr19	25074019	25276922	+	7810	3.525		1

Tmem47	ENSMUSG00000025666	chrX	78316037	78343211	+	4025	3.525	1
Siglec1	ENSMUSG00000027322	chr2	130894956	130912501	-	7143	3.522	1
Fscn1	ENSMUSG00000029581	chr5	143722022	143734864	+	3012	3.52	1
Slc23a2	ENSMUSG00000027340	chr2	131878232	131970844	-	7951	3.518	1
Slc6a6	ENSMUSG00000030096	chr6	91634061	91709057	+	6169	3.518	1
Fam171b	ENSMUSG00000048388	chr2	83652793	83723643	+	5744	3.517	1
Leo1	ENSMUSG00000042487	chr9	75289331	75314239	+	2192	3.517	1
A130022J15Rik	ENSMUSG00000035245	chr6	97060018	97099176	-	4900	3.516	1
Dysf	ENSMUSG00000033788	chr6	83958584	84161054	+	8333	3.515	1
Heatr5a	ENSMUSG00000035181	chr12	52976860	53072308	-	7761	3.514	1
Prdm1	ENSMUSG00000038151	chr10	44156983	44248307	-	5492	3.512	1
Tmed5	ENSMUSG00000063406	chr5	108535385	108561639	-	7688	3.508	1
Qser1	ENSMUSG00000074994	chr2	104594952	104656853	-	8973	3.508	1
Zfp51	ENSMUSG00000023892	chr17	21587316	21602555	+	2600	3.506	1
Gm9938	ENSMUSG00000054178	chr19	23797769	23799524	+	1756	3.505	1
Plxna2	ENSMUSG00000026640	chr1	196446023	196643063	+	15093	3.505	1
Tmx3	ENSMUSG00000024614	chr18	90679546	90712659	+	4611	3.505	1
Ly86	ENSMUSG00000021423	chr13	37437214	37510905	+	997	3.503	1
Rcor3	ENSMUSG00000037395	chr1	193922652	193961892	-	3566	3.503	1
0610010F05Rik	ENSMUSG00000042208	chr11	23464961	23533639	-	5226	3.501	1
Uhmk1	ENSMUSG00000026667	chr1	172123551	172145528	-	8101	3.5	1
Lum	ENSMUSG00000036446	chr10	97028135	97035337	+	2075	3.499	1
Tnfsf9	ENSMUSG00000035678	chr17	57244808	57247180	+	1230	3.498	1
Ranbp2	ENSMUSG00000003226	chr10	57909600	57956903	+	9477	3.498	1
Frmd6	ENSMUSG00000048285	chr12	71926501	72003219	+	4693	3.496	1
Mettl15	ENSMUSG00000057234	chr2	108932454	109120905	-	2275	3.495	1
Zfc3h1	ENSMUSG00000034163	chr10	114822015	114869828	+	7863	3.494	1
Cnpy4	ENSMUSG00000036968	chr5	138628713	138635146	+	2399	3.494	1
Notch4	ENSMUSG00000015468	chr17	34701213	34725448	+	8061	3.493	1
Ncaph	ENSMUSG00000034906	chr2	126929545	126959690	-	4307	3.486	1
Asprv1	ENSMUSG00000033508	chr6	86578168	86579696	+	1529	3.483	1
Cd274	ENSMUSG00000016496	chr19	29441945	29462585	+	3622	3.483	1
Itgb4	ENSMUSG00000020758	chr11	115836023	115869726	+	7015	3.48	1
Sept7	ENSMUSG00000001833	chr9	25056204	25116150	+	3642	3.479	1
Dennd4c	ENSMUSG00000038024	chr4	86394459	86496507	+	8482	3.476	1
Slk	ENSMUSG00000025060	chr19	47654168	47719736	+	8284	3.475	1
Pfdn4	ENSMUSG00000052033	chr2	170321928	170344623	+	2520	3.474	1
Ckap5	ENSMUSG00000040549	chr2	91366919	91460821	+	9462	3.474	1
Mbnl2	ENSMUSG00000022139	chr14	120674891	120830919	+	4564	3.472	1
Kif3c	ENSMUSG00000020668	chr12	3365132	3406494	+	6923	3.472	1

BC013712	ENSMUSG00000037731	chr4	132337758	132352302	-	3079	3.468	1
Plag1	ENSMUSG00000003282	chr4	3828143	3865570	-	8487	3.468	1
Atad1	ENSMUSG00000013662	chr19	32747050	32786812	-	2840	3.467	1
8030462N17Rik	ENSMUSG00000047466	chr18	77743695	77952749	-	3998	3.465	1
Bmp2k	ENSMUSG00000034663	chr5	97426708	97520886	+	8480	3.465	1
Tubb6	ENSMUSG00000001473	chr18	67550371	67562402	+	1757	3.464	1
Heyl	ENSMUSG00000032744	chr4	122910799	122927118	+	5113	3.46	1
Gap43	ENSMUSG00000047261	chr16	42248555	42340764	-	1523	3.459	1
Hsd11b1	ENSMUSG00000016194	chr1	195047824	195090248	-	2708	3.459	1
Fgd3	ENSMUSG00000037946	chr13	49356923	49415680	-	6012	3.455	1
Atad5	ENSMUSG00000017550	chr11	79902902	79949296	+	7532	3.455	1
Fmr1	ENSMUSG00000000838	chrX	65931716	65971138	+	8966	3.454	1
Wwp1	ENSMUSG00000041058	chr4	19535450	19636140	-	6453	3.452	1
Baz1a	ENSMUSG00000035021	chr12	55993976	56115335	-	7159	3.45	1
Tubgcp5	ENSMUSG00000033790	chr7	63049518	63086817	+	3681	3.448	1
Bmp1	ENSMUSG00000022098	chr14	70874364	70920067	-	4156	3.444	1
Phf6	ENSMUSG00000025626	chrX	50265443	50310120	+	6833	3.444	1
Pdgfrb	ENSMUSG00000024620	chr18	61204804	61244715	+	5478	3.444	1
Snx30	ENSMUSG00000028385	chr4	59818712	59917609	+	7795	3.443	1
Cep350	ENSMUSG00000033671	chr1	157692096	157820020	-	13249	3.443	1
Cdc27	ENSMUSG00000020687	chr11	104364059	104411934	-	6248	3.443	1
Sav1	ENSMUSG00000021067	chr12	71065999	71087989	-	2524	3.441	1
Usp32	ENSMUSG00000000804	chr11	84797944	84953663	-	7680	3.44	1
Mfhas1	ENSMUSG00000070056	chr8	36650852	36742503	+	6666	3.439	1
Abcc9	ENSMUSG00000030249	chr6	142536382	142650794	-	7538	3.437	1
Tmem131	ENSMUSG00000026116	chr1	36849036	36996372	-	6546	3.434	1
Utp20	ENSMUSG00000004356	chr10	88209352	88289559	-	8831	3.431	1
Vps35	ENSMUSG00000031696	chr8	87784296	87823412	-	3180	3.43	1
Sept2	ENSMUSG00000026276	chr1	95375541	95406837	+	5022	3.43	1
Slit2	ENSMUSG00000031558	chr5	48374394	48697521	+	15091	3.43	1
Ms4a6b	ENSMUSG00000024677	chr19	11591002	11605746	+	4485	3.429	1
Kif3b	ENSMUSG00000027475	chr2	153117149	153159126	+	5635	3.428	1
Zcchc11	ENSMUSG00000034610	chr4	108132031	108232026	+	8684	3.428	1
Gnai3	ENSMUSG00000000001	chr3	107910198	107949064	-	3262	3.427	1
Nup153	ENSMUSG00000021374	chr13	46775271	46823218	-	6021	3.426	1
Memo1	ENSMUSG00000058704	chr17	74600046	74694223	-	1513	3.426	1
Hnrnph1	ENSMUSG00000007850	chr11	50190492	50200030	+	5842	3.423	1
Slc25a46	ENSMUSG00000024259	chr18	31739822	31770183	-	4468	3.419	1
Fnbp1l	ENSMUSG00000039735	chr3	122241637	122322585	-	5447	3.418	1
Gsr	ENSMUSG00000031584	chr8	34762995	34808635	+	2754	3.412	1

Dmxl1	ENSMUSG00000037416	chr18	49992667	50124739	+	11843	3.41	1
Il11	ENSMUSG00000004371	chr7	4724657	4734459	-	1330	3.409	1
Ascc3	ENSMUSG00000038774	chr10	50312475	50571008	+	7477	3.409	1
Tgfb1	ENSMUSG00000002603	chr7	26472021	26490096	+	2558	3.408	1
Uprt	ENSMUSG00000073016	chrX	101678157	101702829	+	2304	3.407	1
D15Ertd621e	ENSMUSG00000037119	chr15	58247023	58289356	+	5621	3.399	1
Vcl	ENSMUSG00000021823	chr14	21748655	21852895	+	5229	3.399	1
Cep250	ENSMUSG00000038241	chr2	155782194	155824636	+	10373	3.398	1
Fam126a	ENSMUSG00000028995	chr5	23421094	23536508	-	7107	3.396	1
Ttc21b	ENSMUSG00000034848	chr2	66022384	66094674	-	9308	3.394	1
Wnt5a	ENSMUSG00000021994	chr14	29317936	29340633	+	7822	3.391	1
Naaa	ENSMUSG00000029413	chr5	92686685	92707196	-	3721	3.39	1
Senp7	ENSMUSG00000052917	chr16	56075522	56190124	+	5493	3.39	1
Casd1	ENSMUSG00000015189	chr6	4550840	4593381	+	4062	3.39	1
Pilra	ENSMUSG00000046245	chr5	138263180	138277506	-	3116	3.389	1
Slx4	ENSMUSG00000039738	chr16	3979105	4003770	-	7657	3.388	1
Tfrc	ENSMUSG00000022797	chr16	32609006	32632880	+	6185	3.385	1
Gatsl2	ENSMUSG00000015944	chr5	134575581	134620213	+	7560	3.384	1
Matr3	ENSMUSG00000037236	chr18	35721812	35751699	+	4472	3.384	1
Krt7	ENSMUSG00000023039	chr15	101241474	101260744	+	7049	3.382	1
Gm6169	ENSMUSG00000057762	chr13	97868192	97869296	-	1105	3.381	1
Fli1	ENSMUSG00000016087	chr9	32229793	32348974	-	3108	3.381	1
Nrp2	ENSMUSG00000025969	chr1	62749859	62865269	+	9509	3.381	1
Gnpda2	ENSMUSG00000029209	chr5	69964347	69983576	-	6858	3.381	1
Tnfaip3	ENSMUSG00000019850	chr10	18720716	18735463	-	7226	3.38	1
Capza2	ENSMUSG00000015733	chr6	17586234	17616972	+	5890	3.38	1
Arhgap42	ENSMUSG00000050730	chr9	8994953	9239026	-	6313	3.378	1
Itgbl1	ENSMUSG00000032925	chr14	124059193	124374840	+	4229	3.376	1
Uba3	ENSMUSG00000030061	chr6	97133664	97155636	-	2589	3.376	1
Anxa2	ENSMUSG00000032231	chr9	69301427	69339602	+	3731	3.375	1
Ccrn4l	ENSMUSG00000023087	chr3	51028369	51055566	+	10658	3.373	1
Mns1	ENSMUSG00000032221	chr9	72286336	72306381	+	1805	3.373	1
Nmt2	ENSMUSG00000026643	chr2	3201484	3246149	+	6556	3.372	1
Dpep2	ENSMUSG00000053687	chr8	108508844	108520323	-	4540	3.372	1
Lmnb1	ENSMUSG00000024590	chr18	56867467	56913078	+	2962	3.371	1
Cdk1	ENSMUSG00000019942	chr10	68799383	68815686	-	3445	3.369	1
Prkacb	ENSMUSG00000005034	chr3	146392543	146475924	-	5461	3.365	1
Fgf7	ENSMUSG00000027208	chr2	125860394	125916921	+	3248	3.365	1
Rpf1	ENSMUSG00000028187	chr3	146169312	146184387	-	1263	3.365	1
Slc25a37	ENSMUSG00000034248	chr14	69859908	69903160	-	4162	3.364	1
Ranbp6	ENSMUSG00000074909	chr19	29882598	29887464	-	4867	3.362	1
Tns3	ENSMUSG00000020422	chr11	8331655	8564538	-	11182	3.359	1

2010109K11Rik	ENSMUSG00000090946	chr12	33063569	33067808	+	4240	3.359	1
Ccr2	ENSMUSG00000049103	chr9	124016689	124028296	+	6393	3.359	1
Gprc5a	ENSMUSG00000046733	chr6	135015669	135034727	+	2083	3.356	1
Kdelc1	ENSMUSG00000026047	chr1	44148308	44175653	-	3536	3.355	1
Mllt11	ENSMUSG00000053192	chr3	95023057	95032599	-	1923	3.355	1
Lcor	ENSMUSG00000025019	chr19	41557127	41636736	+	7628	3.354	1
Tlr2	ENSMUSG00000027995	chr3	83640195	83645689	-	3013	3.354	1
Zmym2	ENSMUSG00000021945	chr14	57505490	57579182	+	5004	3.354	1
Asah1	ENSMUSG00000031591	chr8	42425551	42460127	-	3390	3.353	1
Tipin	ENSMUSG00000032397	chr9	64129414	64152599	+	1211	3.352	1
Ffar2	ENSMUSG00000051314	chr7	31603376	31608794	-	2534	3.351	1
Fam13b	ENSMUSG00000036501	chr18	34602006	34666477	-	3343	3.35	1
Otud4	ENSMUSG00000036990	chr8	82163517	82201654	+	8127	3.348	1
Fxr1	ENSMUSG00000027680	chr3	33919001	33968264	+	2413	3.346	1
Cd47	ENSMUSG00000055447	chr16	49855731	49915123	+	5277	3.346	1
F2r	ENSMUSG00000048376	chr13	96371759	96388414	-	3307	3.345	1
Csgalnact2	ENSMUSG00000042042	chr6	118057470	118089158	-	5438	3.345	1
Etl4	ENSMUSG00000036617	chr2	19831407	20732340	+	16067	3.345	1
1110034B05Rik	ENSMUSG00000048495	chr1	57445081	57463945	-	2672	3.342	1
Hpgds	ENSMUSG00000029919	chr6	65067287	65094902	-	3586	3.342	1
Sned1	ENSMUSG00000047793	chr1	95132418	95197642	+	9491	3.339	1
Myo10	ENSMUSG00000022272	chr15	25552280	25743428	+	10122	3.339	1
Mir155	ENSMUSG00000091875	chr16	84713268	84715487	+	1261	3.335	1
Sh3glb1	ENSMUSG00000037062	chr3	144347972	144383287	-	4144	3.335	1
Myo19	ENSMUSG00000020527	chr11	84693650	84724728	+	7113	3.333	1
Hdac2	ENSMUSG00000019777	chr10	36694350	36721695	+	4482	3.332	1
Lims1	ENSMUSG00000019920	chr10	57786214	57887439	+	5183	3.331	1
Brwd3	ENSMUSG00000063663	chrX	105932355	106029711	-	12692	3.33	1
Eri1	ENSMUSG00000031527	chr8	36528319	36558587	-	5067	3.33	1
Fam179b	ENSMUSG00000035614	chr12	66066729	66123560	+	6614	3.329	1
Tnpo1	ENSMUSG00000009470	chr13	99608974	99696339	-	8581	3.326	1
Ttll7	ENSMUSG00000036745	chr3	146515331	146646973	+	8180	3.325	1
Col15a1	ENSMUSG00000028339	chr4	47221033	47326039	+	6349	3.323	1
Cxcr3	ENSMUSG00000050232	chrX	98926874	98929608	-	1731	3.32	1
Uhrf1bp1l	ENSMUSG00000019951	chr10	89207736	89282614	+	6446	3.316	1
Wnk1	ENSMUSG00000045962	chr6	119873987	119988690	-	14630	3.315	1
Aplp2	ENSMUSG00000031996	chr9	30957142	31019400	-	3694	3.315	1
Pank3	ENSMUSG00000018846	chr11	35582986	35604787	+	7376	3.314	1

9930038K12Rik	ENSMUSG00000092475	chr6	52178246	52180531	-	2286	3.313	1
2610307P16Rik	ENSMUSG00000085936	chr13	28551903	28977489	-	3293	3.312	1
Tnni2	ENSMUSG00000031097	chr7	149627713	149630315	+	1497	3.311	1
Cdkn1a	ENSMUSG00000023067	chr17	29227924	29237667	+	2223	3.311	1
Adam9	ENSMUSG00000031555	chr8	26060083	26127394	-	4011	3.307	1
Itprip	ENSMUSG00000079254	chr19	47969088	47993789	-	3764	3.307	1
Gfra2	ENSMUSG00000022103	chr14	71289927	71379645	+	3515	3.306	1
Adcyap1r1	ENSMUSG00000029778	chr6	55401972	55451445	+	6601	3.305	1
Usp25	ENSMUSG00000022867	chr16	77014314	77117024	+	4412	3.304	1
Whrn	ENSMUSG00000039137	chr4	63075944	63157025	-	7343	3.301	1
Eftud1	ENSMUSG00000038563	chr7	89797124	89926362	+	4398	3.3	1
Mthfd1l	ENSMUSG00000040675	chr10	6179460	6373423	-	4435	3.299	1
Iars2	ENSMUSG00000026618	chr1	187108605	187153275	-	8885	3.299	1
Stom	ENSMUSG00000026880	chr2	35169506	35192529	-	2820	3.298	1
Exoc5	ENSMUSG00000061244	chr14	49631821	49686328	-	9932	3.295	1
Aplnr	ENSMUSG00000044338	chr2	84976517	84980079	+	3563	3.294	1
Vbp1	ENSMUSG00000031197	chrX	72759638	72780281	+	2132	3.293	1
Ccnl1	ENSMUSG00000027829	chr3	65750073	65762171	-	5997	3.292	1
Pbk	ENSMUSG00000022033	chr14	66424674	66436659	+	1791	3.291	1
D2Ertd750e	ENSMUSG00000027331	chr2	118639739	118679693	+	5158	3.285	1
Tigd2	ENSMUSG00000049232	chr6	59158864	59162027	+	3007	3.284	1
Ccl2	ENSMUSG00000035385	chr11	81849073	81850955	+	1138	3.284	1
Iigp1	ENSMUSG00000054072	chr18	60535683	60552281	+	3121	3.284	1
Kitl	ENSMUSG00000019966	chr10	99478264	99563047	+	5844	3.283	1
Pacsin2	ENSMUSG00000016664	chr15	83206037	83295036	-	3880	3.278	1
Gpr176	ENSMUSG00000040133	chr2	118102846	118199155	-	3899	3.277	1
Osgin2	ENSMUSG00000041153	chr4	15924268	15941035	-	5571	3.275	1
Ncrna00085	ENSMUSG00000080316	chr17	17967683	17979973	+	1955	3.274	1
Phf3	ENSMUSG00000048874	chr1	30859187	30930539	-	8067	3.273	1
Dnajb4	ENSMUSG00000028035	chr3	151841475	151873243	-	4318	3.27	1
Ap1s3	ENSMUSG00000054702	chr1	79603451	79668547	-	2878	3.269	1
Herc3	ENSMUSG00000029804	chr6	58781459	58870392	+	7081	3.266	1
Lemd3	ENSMUSG00000048661	chr10	120360469	120416388	-	4809	3.266	1
Slc2a3	ENSMUSG00000003153	chr6	122677827	122751658	-	7245	3.266	1
Lclat1	ENSMUSG00000054469	chr17	73457325	73592708	+	4689	3.266	1
Adss	ENSMUSG00000015961	chr1	179693093	179726642	-	4531	3.263	1
Gldc	ENSMUSG00000024827	chr19	30172939	30249908	-	3754	3.263	1
Bcap29	ENSMUSG00000020650	chr12	32280219	32319462	-	1954	3.262	1
Ror1	ENSMUSG00000035305	chr4	99768396	100117370	+	5762	3.262	1
Plec	ENSMUSG00000022565	chr15	76001404	76063004	-	18113	3.261	1

Raet1c	ENSMUSG00000053219	chr10	21893327	21903720	+	1274	3.26		1
Mrps22	ENSMUSG00000032459	chr9	98489152	98502098	-	1213	3.258		1
Pphln1	ENSMUSG00000036167	chr15	93228781	93322344	+	4264	3.257		1
A230046K03Rik	ENSMUSG00000034560	chr10	83006686	83059214	+	5854	3.257		1
Lats1	ENSMUSG00000040021	chr10	7401007	7436256	+	7300	3.256		1
Clec4a1	ENSMUSG00000049037	chr6	122871866	122884637	+	1558	3.256		1
E2f3	ENSMUSG00000016477	chr13	29998444	30077537	-	4794	3.256		1
Rgs2	ENSMUSG00000026360	chr1	145846468	145851291	-	3627	3.255		1
Lin7c	ENSMUSG00000027162	chr2	109731010	109741160	+	4521	3.255		1
Trim12c	ENSMUSG00000057143	chr7	111487268	111501876	-	3740	3.254		1
Atp8b2	ENSMUSG00000060671	chr3	89743403	89767430	-	8561	3.253		1
Plk3	ENSMUSG00000028680	chr4	116801260	116806568	-	2531	3.253		1
B630005N14Rik	ENSMUSG00000042742	chr6	13575677	13627966	-	4139	3.251		1
Plscr2	ENSMUSG00000032372	chr9	92170440	92192573	+	3158	3.25		1
Hsd17b12	ENSMUSG00000027195	chr2	93872846	93998121	-	2407	3.249		1
Med13l	ENSMUSG00000018076	chr5	119010728	119215444	+	9320	3.249		1
Mob1b	ENSMUSG00000006262	chr5	89149880	89191083	+	10927	3.248		1
Fam108b	ENSMUSG00000047368	chr19	21727799	21760127	+	2475	3.248		1
Ext1	ENSMUSG00000061731	chr15	52895593	53177714	-	8077	3.246		1
Cryba4	ENSMUSG00000066975	chr5	112675513	112681538	-	848	3.246		1
Esco1	ENSMUSG00000024293	chr18	10566510	10610350	-	4662	3.245		1
Slc26a2	ENSMUSG00000034320	chr18	61352573	61371266	-	8445	3.245		1
Rnasel	ENSMUSG00000066800	chr1	155596556	155611351	+	4758	3.245		1
Naa38	ENSMUSG00000044155	chr6	18798579	18805751	+	4108	3.244		1
Crnkl1	ENSMUSG00000001767	chr2	145743215	145760750	-	3844	3.244		1
Zbtb1	ENSMUSG00000033454	chr12	77471253	77497937	+	12190	3.242		1
Srgap3	ENSMUSG00000030257	chr6	112667965	112897260	-	9852	3.242		1
C130050O18Rik	ENSMUSG00000044092	chr5	139881234	139891577	+	3180	3.241		1
Lrrc32	ENSMUSG00000090958	chr7	105642732	105650341	+	3817	3.237		1
Nupl1	ENSMUSG00000063895	chr14	60824707	60870344	-	2870	3.235		1
Bub1b	ENSMUSG00000040084	chr2	118423947	118467328	+	4279	3.235		1
Eno3	ENSMUSG00000060600	chr11	70470704	70476015	+	1689	3.235		1
Inhbb	ENSMUSG00000037035	chr1	121312042	121318825	-	4255	3.234		1
Ankrd32	ENSMUSG00000021597	chr13	77182349	77274734	-	5881	3.232		1
Suz12	ENSMUSG00000017548	chr11	79806608	79847625	+	4945	3.23		1
Stk38l	ENSMUSG00000001630	chr6	146673517	146727334	+	4571	3.23		1
Rab21	ENSMUSG00000020132	chr10	114726919	114752647	-	1823	3.23		1
Tm6sf1	ENSMUSG00000038623	chr7	89003887	89029320	+	5921	3.23		1

Btbd3	ENSMUSG00000062098	chr2	138082301	138415028	+	5776	3.229	1
Prickle1	ENSMUSG00000036158	chr15	93329545	93426322	-	4345	3.229	1
Dll1	ENSMUSG00000014773	chr17	15504318	15513836	-	4219	3.228	1
Hnrnph2	ENSMUSG00000045427	chrX	131135718	131141599	+	4369	3.227	1
Lgmn	ENSMUSG00000021190	chr12	103632294	103678023	-	2323	3.226	1
Tram2	ENSMUSG00000041779	chr1	20986377	21069310	-	6495	3.226	1
Tmem188	ENSMUSG00000036810	chr8	90642668	90659009	+	3563	3.226	1
Cdh5	ENSMUSG00000031871	chr8	106625525	106668402	+	3986	3.225	1
Tmem48	ENSMUSG00000028614	chr4	107040389	107088951	+	4924	3.225	1
Dhh	ENSMUSG00000023000	chr15	98723462	98728971	-	2363	3.225	1
Zc3h14	ENSMUSG00000021012	chr12	99985178	100025984	+	4184	3.222	1
Ltv1	ENSMUSG00000019814	chr10	12898446	12912943	-	1766	3.221	1
Acot9	ENSMUSG00000025287	chrX	151696986	151732197	+	2273	3.22	1
2010106G01Rik	ENSMUSG00000027366	chr2	126716127	126758971	-	8665	3.22	1
Bves	ENSMUSG00000071317	chr10	45055568	45089514	+	1607	3.219	1
Eid1	ENSMUSG00000091337	chr2	125498831	125500521	+	1691	3.218	1
Thbd	ENSMUSG00000074743	chr2	148230207	148233924	-	3718	3.216	1
Papolg	ENSMUSG00000020273	chr11	23762646	23795253	-	5491	3.216	1
Atr	ENSMUSG00000032409	chr9	95758016	95852063	+	8499	3.215	1
Strn	ENSMUSG00000024077	chr17	79049253	79136536	-	8858	3.214	1
Sparc	ENSMUSG00000018593	chr11	55208002	55233582	-	3367	3.212	1
Pus3	ENSMUSG00000032103	chr9	35366180	35374768	+	2754	3.209	1
Zcchc24	ENSMUSG00000055538	chr14	26531126	26588342	-	4375	3.208	1
Myh9	ENSMUSG00000022443	chr15	77591017	77672605	-	12928	3.207	1
Homer1	ENSMUSG00000007617	chr13	94073712	94174917	+	11340	3.205	1
Cgnl1	ENSMUSG00000032232	chr9	71474316	71619409	-	7844	3.204	1
Cybb	ENSMUSG00000015340	chrX	9012378	9064897	-	5808	3.204	1
Plbd1	ENSMUSG00000030214	chr6	136560591	136610449	-	2633	3.201	1
Gjc1	ENSMUSG00000034520	chr11	102660893	102681014	-	5003	3.2	1
Syncrip	ENSMUSG00000032423	chr9	88344567	88377412	-	7742	3.2	1
Pno1	ENSMUSG00000020116	chr11	17103201	17111571	-	1717	3.197	1
Palmd	ENSMUSG00000033377	chr3	116621174	116671905	-	3812	3.196	1
Rap1gap	ENSMUSG00000041351	chr4	137220641	137285782	+	6435	3.196	1
1110002B05Rik	ENSMUSG00000044408	chr12	55746361	55757559	-	1363	3.193	1
Zfp799	ENSMUSG00000059000	chr17	32956289	32959145	-	1944	3.192	1
Sfr1	ENSMUSG00000025066	chr19	47806246	47810078	+	1583	3.192	1
5430416N02Rik	ENSMUSG00000084877	chr5	100849861	100858535	-	3691	3.191	1

Lsm14a	ENSMUSG00000066568	chr7	35129665	35174559	-	3104	3.189	1
Psmc6	ENSMUSG00000021832	chr14	45949504	45969367	+	2180	3.187	1
Map4k3	ENSMUSG00000024242	chr17	80979852	81127433	-	4231	3.187	1
Apc	ENSMUSG00000005871	chr18	34380578	34481843	+	15750	3.183	1
Tmcc3	ENSMUSG00000020023	chr10	93774694	94053701	+	7153	3.183	1
Nedd4	ENSMUSG00000032216	chr9	72510364	72597649	+	5278	3.183	1
Diap2	ENSMUSG00000034480	chrX	126284278	127000370	+	11605	3.183	1
Stk17b	ENSMUSG00000026094	chr1	53812350	53842068	-	3293	3.183	1
Moxd1	ENSMUSG00000020000	chr10	23943323	24022596	+	3198	3.182	1
Mbd5	ENSMUSG00000036792	chr2	48805030	49177804	+	14221	3.182	1
Mbip	ENSMUSG00000021028	chr12	57429293	57446881	-	1509	3.181	1
Npr3	ENSMUSG00000022206	chr15	11769651	11835429	-	7067	3.181	1
Ints2	ENSMUSG00000018068	chr11	86024183	86071077	-	10609	3.18	1
Arrdc3	ENSMUSG00000074794	chr13	81022645	81035296	+	6259	3.178	1
Ccdc74a	ENSMUSG00000041617	chr16	17646563	17650831	+	1278	3.178	1
Syne2	ENSMUSG00000063450	chr12	76919121	77211913	+	26726	3.177	1
Ikzf1	ENSMUSG00000018654	chr11	11585006	11672929	+	5919	3.177	1
Fbxo30	ENSMUSG00000047648	chr10	11001128	11017850	+	5197	3.174	1
Ptpn13	ENSMUSG00000034573	chr5	103854211	104027378	+	8332	3.173	1
Lrrc8b	ENSMUSG00000070639	chr5	105844794	105915208	+	2841	3.173	1
Efna5	ENSMUSG00000048915	chr17	62953533	63230666	-	4032	3.171	1
Pde12	ENSMUSG00000043702	chr14	27483603	27489332	-	3529	3.171	1
Eif2c2	ENSMUSG00000093631	chr15	72926274	72927114	-	841	3.171	1
Tpr	ENSMUSG00000006005	chr1	152239968	152297065	+	10499	3.169	1
Fyttd1	ENSMUSG00000022800	chr16	32877586	32909049	+	6076	3.169	1
Casp8	ENSMUSG00000026029	chr1	58852218	58904347	+	2777	3.168	1
Zfp386	ENSMUSG00000042063	chr12	117280562	117299060	+	2081	3.167	1
1600021P15Rik	ENSMUSG00000051065	chr16	28826262	28929784	-	3285	3.167	1
Axl	ENSMUSG00000002602	chr7	26542292	26573724	-	5033	3.166	1
Racgap1	ENSMUSG00000023015	chr15	99450927	99482087	-	7087	3.166	1
Fam38a	ENSMUSG00000014444	chr8	125005598	125075229	-	9933	3.165	1
Topbp1	ENSMUSG00000032555	chr9	103207545	103252762	+	5228	3.164	1
Tspan7	ENSMUSG00000058254	chrX	10062284	10173731	+	7067	3.164	1
Taok1	ENSMUSG00000017291	chr11	77342664	77421317	-	12421	3.163	1
Adamts15	ENSMUSG00000033453	chr9	30706740	30730037	-	5928	3.162	1
Cflar	ENSMUSG00000026031	chr1	58768352	58815728	+	9478	3.161	1
Golim4	ENSMUSG00000034109	chr3	75680105	75760871	-	8977	3.158	1
Tanc1	ENSMUSG00000035168	chr2	59450099	59684206	+	14570	3.156	1
Adam17	ENSMUSG00000052593	chr12	21329370	21379493	-	7154	3.155	1
Pogk	ENSMUSG00000040596	chr1	168314753	168339994	-	8204	3.154	1

Fam33a	ENSMUSG00000020492	chr11	86922736	86938137	+	3272	3.153	1
Plk2	ENSMUSG00000021701	chr13	111185252	111191052	+	2802	3.151	1
Ncf2	ENSMUSG00000026480	chr1	154655020	154684120	+	2596	3.15	1
4933436C20Rik	ENSMUSG00000031736	chr8	94849937	94880019	-	1238	3.15	1
Bdp1	ENSMUSG00000049658	chr13	100787949	100874025	-	11005	3.15	1
A830082K12Rik	ENSMUSG00000087143	chr13	78337278	78375825	+	3503	3.149	1
Bst1	ENSMUSG00000029082	chr5	44210125	44234510	+	2443	3.149	1
Hspa4	ENSMUSG00000020361	chr11	53073316	53113959	-	5528	3.149	1
D5Ertd579e	ENSMUSG00000029190	chr5	36927437	37087263	-	8215	3.149	1
Klhl2	ENSMUSG00000031605	chr8	67218660	67373716	-	3025	3.148	1
Aoah	ENSMUSG00000021322	chr13	20885988	21116121	+	3181	3.148	1
Xiap	ENSMUSG00000025860	chrX	39412856	39462833	+	9262	3.148	1
Ift74	ENSMUSG00000028576	chr4	94281182	94359920	+	2656	3.147	1
Gm13056	ENSMUSG00000085395	chr4	140834354	140835709	+	564	3.146	1
Sirpa	ENSMUSG00000037902	chr2	129418571	129457964	+	4901	3.144	1
Pole	ENSMUSG00000007080	chr5	110715325	110766493	+	9590	3.143	1
Abcb7	ENSMUSG00000031333	chrX	101475996	101609195	-	5905	3.142	1
Hoxd8	ENSMUSG00000027102	chr2	74542672	74545990	+	2744	3.142	1
Ssfa2	ENSMUSG00000027007	chr2	79475509	79513123	+	5776	3.14	1
Net1	ENSMUSG00000021215	chr13	3881811	3917466	-	3734	3.14	1
Gm13470	ENSMUSG00000086644	chr2	46275055	46298230	-	421	3.139	1
Mdm4	ENSMUSG00000054387	chr1	134886422	134921943	-	3511	3.138	1
Appbp2	ENSMUSG00000018481	chr11	85000764	85048632	-	6463	3.137	1
Anxa5	ENSMUSG00000027712	chr3	36347847	36374765	-	1678	3.135	1
Ctnnb1	ENSMUSG00000006932	chr9	120838334	120869625	+	5099	3.135	1
F3	ENSMUSG00000028128	chr3	121426455	121437970	+	1876	3.134	1
Zfp26	ENSMUSG00000063108	chr9	20237416	20264606	-	9631	3.134	1
Rin1	ENSMUSG00000024883	chr19	5050808	5057071	+	4176	3.132	1
Il10ra	ENSMUSG00000032089	chr9	45061920	45077232	-	3498	3.13	1
Pds5b	ENSMUSG00000034021	chr5	151476314	151613265	+	9397	3.129	1
Col6a3	ENSMUSG00000048126	chr1	92663435	92740529	-	11950	3.126	1
Tgfbr1	ENSMUSG00000007613	chr4	47366094	47427803	+	6534	3.126	1
Taf1b	ENSMUSG00000059669	chr12	25183446	25243436	+	2019	3.126	1
Ano1	ENSMUSG00000031075	chr7	151774454	151937879	-	5180	3.126	1
Kcnmb4	ENSMUSG00000054934	chr10	115854917	115910934	-	3324	3.122	1
Dkk3	ENSMUSG00000030772	chr7	119259533	119302571	-	3357	3.122	1
Fcgr3	ENSMUSG00000059498	chr1	172981300	172995066	-	1741	3.122	1
Sgsm1	ENSMUSG00000042216	chr5	113672240	113739806	-	7466	3.121	1
Ms4a8a	ENSMUSG00000024730	chr19	11141962	11155593	-	1081	3.118	1
Scai	ENSMUSG00000035236	chr2	38921734	39046254	-	12180	3.118	1

Bclaf1	ENSMUSG00000037608	chr10	20032275	20062307	+	5636	3.118	1
Phtf1	ENSMUSG00000058388	chr3	103772033	103828521	+	7294	3.117	1
Inf2	ENSMUSG00000037679	chr12	113826995	113853767	+	4600	3.116	1
Eme1	ENSMUSG00000039055	chr11	94506310	94515278	-	2762	3.116	1
Abl2	ENSMUSG00000026596	chr1	158488917	158579699	+	10664	3.116	1
Fndc3b	ENSMUSG00000039286	chr3	27315084	27610229	-	11408	3.115	1
Tmem1 00	ENSMUSG00000069763	chr11	89891662	89897822	+	1767	3.114	1
Sox4	ENSMUSG00000076431	chr13	29040788	29045582	-	4795	3.112	1
Ino80c	ENSMUSG00000047989	chr18	24263258	24280454	-	2828	3.112	1
Ahctf1	ENSMUSG00000026491	chr1	181675025	181733811	-	9655	3.111	1
Pcdhb9	ENSMUSG00000051242	chr18	37560509	37563563	+	3055	3.11	1
Lgals8	ENSMUSG00000057554	chr13	12531682	12557211	-	3688	3.11	1
Tcf20	ENSMUSG00000041852	chr15	82639059	82742564	-	7347	3.11	1
B23021 9D22Rik	ENSMUSG00000045767	chr13	55794485	55804860	+	4651	3.109	1
Golga7	ENSMUSG00000015341	chr8	24351825	24367546	-	2383	3.109	1
Ogt	ENSMUSG00000034160	chrX	98835399	98879690	+	8045	3.107	1
Rg9mtd 1	ENSMUSG00000044763	chr16	56032722	56037932	-	3002	3.107	1
Abce1	ENSMUSG00000058355	chr8	82207341	82235639	-	3813	3.105	1
Ubr5	ENSMUSG00000037487	chr15	37897083	38008608	-	9790	3.105	1
Yaf2	ENSMUSG00000022634	chr15	93114709	93167366	-	1873	3.105	1
Akap11	ENSMUSG00000022016	chr14	78892053	78936667	-	9419	3.101	1
Rbl1	ENSMUSG00000027641	chr2	156971629	157030270	-	5993	3.1	1
Vps13c	ENSMUSG00000035284	chr9	67688203	67843441	+	11616	3.099	1
Top2b	ENSMUSG00000017485	chr14	17197693	17263301	+	6355	3.098	1
Utp18	ENSMUSG00000054079	chr11	93720557	93747080	-	4983	3.098	1
Trappc8	ENSMUSG00000033382	chr18	20975725	21054579	-	5482	3.096	1
Dsel	ENSMUSG00000038702	chr1	113755279	113761486	-	5754	3.095	1
Pbx3	ENSMUSG00000038718	chr2	34026977	34228660	-	6683	3.095	1
Ckap2l	ENSMUSG00000048327	chr2	129093946	129122948	-	3137	3.095	1
Dpysl2	ENSMUSG00000022048	chr14	67421701	67487525	-	4520	3.093	1
Pgm1	ENSMUSG00000029171	chr5	64484175	64519594	+	2608	3.093	1
Snx9	ENSMUSG00000002365	chr17	5841346	5931946	+	3306	3.093	1
I830077 J02Rik	ENSMUSG00000074342	chr3	105728809	105735582	-	1328	3.092	1
Mdfic	ENSMUSG00000041390	chr6	15670661	15752169	+	7427	3.091	1
Gan	ENSMUSG00000052557	chr8	119682035	119729734	+	3300	3.091	1
9930021 J03Rik	ENSMUSG00000046138	chr19	29788892	29880479	-	8134	3.091	1
Lcorl	ENSMUSG00000015882	chr5	46088420	46248854	-	10945	3.086	1
Prrc2c	ENSMUSG00000040225	chr1	164603533	164670681	-	8824	3.085	1
Cox6a2	ENSMUSG00000030785	chr7	135348950	135349880	-	658	3.084	1
Arl6ip6	ENSMUSG00000026960	chr2	53050760	53078254	+	2703	3.083	1
Patl1	ENSMUSG00000046139	chr19	11986889	12019586	+	4221	3.083	1
Smad3	ENSMUSG00000032402	chr9	63494574	63605801	-	5542	3.082	1

Gtse1	ENSMUSG00000022385	chr15	85690137	85707002	+	2779	3.082		1
Nnmt	ENSMUSG00000032271	chr9	48399982	48413258	-	1576	3.081		1
Chm	ENSMUSG00000025531	chrX	110154204	110299126	-	6778	3.077		1
Pus7l	ENSMUSG00000033356	chr15	94353119	94373978	-	3291	3.076		1
Tnks	ENSMUSG00000031529	chr8	35892233	36028744	-	6444	3.076		1
Rnf111	ENSMUSG00000032217	chr9	70273236	70351532	-	5003	3.075		1
Klf3	ENSMUSG00000029178	chr5	65194628	65221368	+	2613	3.074		1
Cd48	ENSMUSG00000015355	chr1	173612140	173635389	+	1189	3.072		1
Anapc4	ENSMUSG00000029176	chr5	53225254	53259036	+	5902	3.072		1
Dock2	ENSMUSG00000020143	chr11	34126815	34597394	-	11425	3.072		1
Uhrf2	ENSMUSG00000024817	chr19	30105003	30168212	+	8590	3.072		1
2810474 O19Rik	ENSMUSG00000032712	chr6	149257936	149284180	+	6597	3.071		1
Slc7a11	ENSMUSG00000027737	chr3	50168858	50247535	-	11245	3.07		1
Zfp605	ENSMUSG00000023284	chr5	110539111	110560756	+	3586	3.07		1
Ywhag	ENSMUSG00000051391	chr5	136384279	136410486	-	5500	3.069		1
Pkn2	ENSMUSG00000004591	chr3	142453866	142544968	-	7426	3.069		1
Plekha1	ENSMUSG00000040268	chr7	138009270	138056826	+	8527	3.069		1
Oas2	ENSMUSG00000032690	chr5	121180342	121199862	-	4451	3.067		1
Ept1	ENSMUSG00000075703	chr5	30559121	30598558	+	10855	3.064		1
Hhat	ENSMUSG00000037375	chr1	194336710	194597413	-	8922	3.064		1
Ifit2	ENSMUSG00000045932	chr19	34625184	34650909	+	3927	3.064		1
Dach1	ENSMUSG00000055639	chr14	98186066	98568984	-	7323	3.063		1
Cdc42b pa	ENSMUSG00000026490	chr1	181890603	182095734	+	18977	3.063		1
Arhgap1 9	ENSMUSG00000025154	chr19	41841078	41876574	-	5140	3.063		1
Prps2	ENSMUSG00000025742	chrX	163784254	163820681	-	3873	3.063		1
Lrrc49	ENSMUSG00000047766	chr9	60416666	60535965	-	6310	3.062		1
Gimap8	ENSMUSG00000064262	chr6	48597233	48610874	+	3700	3.061		1
Cldn25	ENSMUSG00000022744	chr16	58728023	58734364	+	2563	3.059		1
Fam45a	ENSMUSG00000024993	chr19	60887488	60912130	+	6242	3.057		1
Xpot	ENSMUSG00000034667	chr10	121024436	121063372	-	5992	3.057		1
Pi4k2b	ENSMUSG00000029186	chr5	53132826	53160583	+	3751	3.054		1
Crlf3	ENSMUSG00000017561	chr11	79859995	79894470	-	2730	3.054		1
Mex3a	ENSMUSG00000074480	chr3	88336317	88345318	+	5793	3.053		1
S100a8	ENSMUSG00000056054	chr3	90472993	90473956	+	392	3.052		1
Plaa	ENSMUSG00000028577	chr4	94234205	94269935	-	6441	3.051		1
Ier5	ENSMUSG00000056708	chr1	156943497	156946766	-	3270	3.051		1
Svil	ENSMUSG00000024236	chr18	4920538	5119297	+	10633	3.05		1
4932438 A13Rik	ENSMUSG00000037270	chr3	36762028	36951955	+	26987	3.049		1
Zfp236	ENSMUSG00000041258	chr18	82762989	82862126	-	9555	3.049		1
Ccdc66	ENSMUSG00000046753	chr14	28295598	28321646	-	2940	3.047		1

Rhbdl2	ENSMUSG00000043333	chr4	123465117	123507147	+	3070	3.046	1
Ccl8	ENSMUSG00000009185	chr11	81928687	81930301	+	511	3.045	1
Uchl1	ENSMUSG00000029223	chr5	67067337	67078470	+	1616	3.044	1
Ankrd29	ENSMUSG00000057766	chr18	12410871	12464229	-	3295	3.042	1
Wdr35	ENSMUSG00000066643	chr12	8980698	9035653	+	7273	3.042	1
Hprt	ENSMUSG00000025630	chrX	50341314	50374836	+	1289	3.042	1
Ehd4	ENSMUSG00000027293	chr2	119914911	119980342	-	4007	3.04	1
P2ry14	ENSMUSG00000036381	chr3	58918548	58934546	-	1797	3.04	1
Orc4	ENSMUSG00000026761	chr2	48758344	48805797	-	7458	3.039	1
Wdr26	ENSMUSG00000038733	chr1	183103359	183142109	-	8274	3.037	1
Golph3	ENSMUSG00000022200	chr15	12251205	12281346	+	3027	3.037	1
Sp3	ENSMUSG00000027109	chr2	72774484	72818503	-	4968	3.035	1
Ugcg	ENSMUSG00000028381	chr4	59202129	59235705	+	5212	3.034	1
Paqr8	ENSMUSG00000025931	chr1	20880691	20929632	+	5385	3.034	1
Tnfrsf12a	ENSMUSG00000023905	chr17	23812414	23814416	-	963	3.033	1
8430410K20Rik	ENSMUSG00000041124	chr9	4376612	4386861	+	2741	3.033	1
Tm4sf1	ENSMUSG00000027800	chr3	57089533	57105841	-	3355	3.032	1
Dlg3	ENSMUSG00000000881	chrX	97963061	98013749	+	5844	3.031	1
Zfp597	ENSMUSG00000039789	chr16	3858321	3884561	-	6660	3.031	1
Hnrpll	ENSMUSG00000024095	chr17	80428827	80461674	-	3116	3.031	1
Trim33	ENSMUSG00000033014	chr3	103083216	103162691	+	8868	3.031	1
Atl3	ENSMUSG00000024759	chr19	7568530	7613098	+	6698	3.03	1
Nup93	ENSMUSG00000032939	chr8	96738497	96838966	+	2882	3.027	1
Rlim	ENSMUSG00000056537	chrX	101152502	101176623	-	8917	3.026	1
Chmp2b	ENSMUSG00000004843	chr16	65539378	65562942	-	1838	3.026	1
Ankrd28	ENSMUSG00000014496	chr14	32513201	32643601	-	5229	3.026	1
Gmfb	ENSMUSG00000062014	chr14	47427824	47441917	-	4446	3.025	1
Rpe	ENSMUSG00000026005	chr1	66747405	66766379	+	3113	3.025	1
Mir17hg	ENSMUSG00000089726	chr14	115442101	115445949	+	3536	3.024	1
Gpr126	ENSMUSG00000039116	chr10	14122391	14264842	-	6505	3.024	1
Scara3	ENSMUSG00000034463	chr14	66538232	66572581	-	3405	3.023	1
Smc5	ENSMUSG00000024943	chr19	23283224	23348367	-	3462	3.023	1
Crkl	ENSMUSG00000006134	chr16	17452080	17486348	+	3863	3.022	1
Smoc2	ENSMUSG00000023886	chr17	14416513	14541797	+	2867	3.019	1
5430411K18Rik	ENSMUSG00000039840	chr18	78135239	78231750	+	9623	3.018	1
Oaf	ENSMUSG00000032014	chr9	43029361	43047899	-	2388	3.018	1
Ddhd1	ENSMUSG00000037697	chr14	46212849	46277818	-	6759	3.018	1
Gm340	ENSMUSG00000090673	chr19	41656860	41661026	+	3729	3.018	1
Irs2	ENSMUSG00000038894	chr8	10986980	11008458	-	4024	3.015	1
Pim1	ENSMUSG00000024014	chr17	29627757	29632390	+	1972	3.015	1

Cadm4	ENSMUSG00000054793	chr7	25267042	25289558	+	2161	3.015		1
Ctdspl2	ENSMUSG00000033411	chr2	121781737	121839378	+	9251	3.014		1
Dpy19l4	ENSMUSG00000045205	chr4	11188462	11249284	-	8101	3.013		1
Uxs1	ENSMUSG00000057363	chr1	43803811	43884645	-	5097	3.013		1
Rttn	ENSMUSG00000023066	chr18	89141182	89300405	+	7421	3.012		1
Map4k5	ENSMUSG00000034761	chr12	70904737	70994187	-	8614	3.012		1
Tgfbr2	ENSMUSG00000032440	chr9	115993415	116084383	-	8088	3.012		1
Acvr1b	ENSMUSG00000000532	chr15	101004556	101043032	+	3277	3.011		1
Rassf5	ENSMUSG00000026430	chr1	133072987	133141835	-	3839	3.01		1
Slc25a15	ENSMUSG00000031482	chr8	23486026	23509093	-	3451	3.01		1
Fbxo11	ENSMUSG00000005371	chr17	88390199	88464631	-	4144	3.01		1
Gli3	ENSMUSG00000021318	chr13	15555068	15821859	+	9589	3.008		1
Col4a3bp	ENSMUSG00000021669	chr13	97312690	97410122	+	5363	3.006		1
Irgm1	ENSMUSG00000046879	chr11	48675470	48685185	-	5810	3.003		1
Hook3	ENSMUSG00000037234	chr8	27131893	27229696	-	13138	3.003		1
Cpz	ENSMUSG00000036596	chr5	35844864	35868347	-	2248	2.999		1
Zfml	ENSMUSG00000030016	chr6	83864347	83936863	+	6479	2.999		1
Macf1	ENSMUSG00000028649	chr4	123026876	123361603	-	26705	2.997		1
Eif2ak2	ENSMUSG00000024079	chr17	79251904	79281913	-	5113	2.997		1
Tdrd7	ENSMUSG00000035517	chr4	45978206	46047633	+	4026	2.995		1
Ptk2	ENSMUSG00000022607	chr15	73035535	73253621	-	5120	2.995		1
Maml1	ENSMUSG00000050567	chr11	50069136	50105838	-	5553	2.994		1
Them4	ENSMUSG00000028145	chr3	94114011	94136462	+	4025	2.991		1
Cnn2	ENSMUSG00000004665	chr10	79451329	79458807	+	2460	2.991		1
Pappa2	ENSMUSG00000073530	chr1	160641862	160887870	-	9080	2.99		1
Prnp	ENSMUSG00000079037	chr2	131735664	131764165	+	2343	2.988		1
Commd8	ENSMUSG00000029213	chr5	72548239	72559422	-	3482	2.986		1
Zmiz1	ENSMUSG00000007817	chr14	26278671	26486229	+	12523	2.985		1
Manea	ENSMUSG00000040520	chr4	26251653	26274038	-	4867	2.985		1
Fam110c	ENSMUSG00000036136	chr12	31758833	31764802	+	2667	2.984		1
Pag1	ENSMUSG00000027508	chr3	9687479	9833679	-	9580	2.984		1
Exoc2	ENSMUSG00000021357	chr13	30905788	31065916	-	4427	2.984		1
Tacc3	ENSMUSG00000037313	chr5	34000777	34021644	+	3551	2.984		1
2810008M24Rik	ENSMUSG00000071180	chr13	108836618	108839340	+	1852	2.983		1
Ap4e1	ENSMUSG00000001998	chr2	126834453	126893645	+	9810	2.981		1
Prpf39	ENSMUSG00000035597	chr12	66137320	66164373	+	4401	2.981		1
Arhgap17	ENSMUSG00000030766	chr7	130422664	130513429	-	3605	2.981		1
Ocrl	ENSMUSG00000001173	chrX	45265564	45319045	+	9486	2.98		1
Rnf38	ENSMUSG00000035696	chr4	44139082	44246661	-	8336	2.98		1
Pcolce2	ENSMUSG00000015354	chr9	95538020	95598515	+	4625	2.979		1

Zhx1	ENSMUSG00000022361	chr15	57878558	57908096	-	6059	2.979	1
Kdm3a	ENSMUSG00000053470	chr6	71538968	71582899	-	4964	2.976	1
Frs2	ENSMUSG00000020170	chr10	116507185	116585530	-	5682	2.974	1
Ube2d1	ENSMUSG00000019927	chr10	70717728	70748010	-	4371	2.973	1
Trbj1-2	ENSMUSG00000076484	chr6	41484000	41484047	+	48	2.972	1
Trbj2-7	ENSMUSG00000076497	chr6	41493809	41493855	+	47	2.972	1
Olfml2b	ENSMUSG00000038463	chr1	172574663	172612920	+	3095	2.972	1
Appl1	ENSMUSG00000040760	chr14	27732174	27784418	-	8719	2.971	1
Zbp1	ENSMUSG00000027514	chr2	173032113	173044424	-	3529	2.97	1
Nnat	ENSMUSG00000067786	chr2	157385814	157388258	+	1829	2.97	1
Mtx3	ENSMUSG00000021704	chr13	93614742	93628185	+	6275	2.97	1
Ptpn9	ENSMUSG00000032290	chr9	56842775	56910612	+	4054	2.968	1
S100a11	ENSMUSG00000027907	chr3	93324410	93330209	+	511	2.967	1
Ralgps2	ENSMUSG00000026594	chr1	158734297	158869757	-	7028	2.967	1
Gpsm2	ENSMUSG00000027883	chr3	108481556	108525105	-	3787	2.964	1
Gas7	ENSMUSG00000033066	chr11	67268939	67502492	+	11342	2.963	1
Nckap1	ENSMUSG00000027002	chr2	80340669	80421537	-	4964	2.963	1
Hivep2	ENSMUSG00000015501	chr10	13686185	13871182	+	9766	2.961	1
Jak3	ENSMUSG00000031805	chr8	74200195	74214474	+	5359	2.961	1
Nt5dc3	ENSMUSG00000054027	chr10	86241750	86301134	+	5977	2.96	1
Ccdc50	ENSMUSG00000038127	chr16	27388955	27452304	+	8639	2.958	1
Setd2	ENSMUSG00000044791	chr9	110435101	110521137	+	10537	2.958	1
Impact	ENSMUSG00000024423	chr18	13130761	13151457	+	3432	2.954	1
Heatr5b	ENSMUSG00000039414	chr17	79152245	79234721	-	6801	2.954	1
Zfp260	ENSMUSG00000049421	chr7	30879796	30892641	+	4419	2.954	1
Zfr	ENSMUSG00000022201	chr15	12047586	12115438	+	5911	2.953	1
Map3k8	ENSMUSG00000024235	chr18	4331325	4353013	-	4504	2.952	1
Tshz3	ENSMUSG00000021217	chr7	37483137	37558564	+	5448	2.952	1
Usp12	ENSMUSG00000029640	chr5	147546385	147606582	-	4639	2.952	1
Npat	ENSMUSG00000033054	chr9	53345152	53382447	+	6248	2.951	1
AI429214	ENSMUSG00000074384	chr8	38056629	38058585	+	1957	2.951	1
Ptcd3	ENSMUSG00000063884	chr6	71830642	71858756	-	2585	2.95	1
Syt12	ENSMUSG00000049303	chr19	4445908	4477447	-	5539	2.949	1
5730469M10Rik	ENSMUSG00000021792	chr14	41807029	41827077	-	2948	2.949	1
Enpp1	ENSMUSG00000037370	chr10	24357720	24431965	-	10257	2.947	1
Cul5	ENSMUSG00000032030	chr9	53422687	53478119	-	8165	2.946	1
Gpcpd1	ENSMUSG00000027346	chr2	132354818	132413465	-	7289	2.946	1
L3mbtl3	ENSMUSG00000039089	chr10	25994274	26094991	-	4746	2.945	1
Mgea5	ENSMUSG00000025220	chr19	45824751	45858236	-	5076	2.944	1
Tmpo	ENSMUSG00000019961	chr10	90610316	90634759	-	6674	2.944	1
Zfp367	ENSMUSG00000044934	chr13	64234330	64254510	-	5454	2.943	1
Bcl2a1b	ENSMUSG00000089929	chr9	89094047	89102665	+	817	2.941	1

Ikbip	ENSMUSG00000019975	chr10	90545685	90565352	+	4894	2.938	1
AW549877	ENSMUSG00000041935	chr15	3932036	3945752	-	5083	2.937	1
Zmym6	ENSMUSG00000042408	chr4	126754627	126801616	+	7692	2.936	1
Pcgf5	ENSMUSG00000024805	chr19	36453557	36530682	+	1719	2.936	1
Cep290	ENSMUSG00000019971	chr10	99950923	100036289	+	8006	2.935	1
Smad7	ENSMUSG00000025880	chr18	75527183	75555589	+	4521	2.935	1
Aox1	ENSMUSG00000063558	chr1	58086775	58163257	+	8690	2.934	1
Ttc12	ENSMUSG00000040219	chr9	49245068	49294330	-	4545	2.934	1
Tdp2	ENSMUSG00000035958	chr13	24923548	24934022	+	2095	2.929	1
Fgr	ENSMUSG00000028874	chr4	132530010	132557825	+	3863	2.927	1
Kremen1	ENSMUSG00000020393	chr11	5091555	5161561	-	7760	2.927	1
Zbtb25	ENSMUSG00000056459	chr12	77448769	77470589	-	3131	2.926	1
Filip1l	ENSMUSG00000043336	chr16	57353206	57573239	+	5335	2.925	1
Gpr39	ENSMUSG00000026343	chr1	127573572	127770439	+	3015	2.925	1
Mtmr10	ENSMUSG00000030522	chr7	71432539	71485293	+	7474	2.924	1
Stag2	ENSMUSG00000025862	chrX	39502494	39630362	+	8273	2.923	1
2810046L04Rik	ENSMUSG00000049504	chr3	53267588	53285677	+	5084	2.92	1
Whsc1	ENSMUSG00000057406	chr5	34163374	34240624	+	15902	2.92	1
Cpeb2	ENSMUSG00000039782	chr5	43624702	43680963	+	7122	2.919	1
Treml2	ENSMUSG00000071068	chr17	48438864	48451859	+	4918	2.917	1
Mettl14	ENSMUSG00000028114	chr3	123067563	123088908	-	5119	2.916	1
D17H6S56E-5	ENSMUSG00000091747	chr17	35133681	35138609	-	3996	2.916	1
Hdac4	ENSMUSG00000026313	chr1	93829334	94044970	-	4091	2.916	1
Mob1a	ENSMUSG00000043131	chr6	83276010	83293770	+	4794	2.915	1
Mecom	ENSMUSG00000027684	chr3	29850221	30446930	-	10581	2.914	1
Cops2	ENSMUSG00000027206	chr2	125656040	125684875	-	3549	2.914	1
4930430F08Rik	ENSMUSG00000046567	chr10	100034908	100051893	-	2419	2.909	1
Ppwd1	ENSMUSG00000021713	chr13	104995201	105018924	-	2397	2.909	1
Pxk	ENSMUSG00000033885	chr14	8930725	8997625	+	2842	2.909	1
Caprin1	ENSMUSG00000027184	chr2	103603098	103637806	-	10219	2.908	1
Sirt1	ENSMUSG00000020063	chr10	62781753	62844452	-	5331	2.908	1
Gpd2	ENSMUSG00000026827	chr2	57090046	57223130	+	6549	2.908	1
Fam172a	ENSMUSG00000064138	chr13	77847951	78305499	+	7309	2.907	1
Cacybp	ENSMUSG00000014226	chr1	162133056	162142908	-	1442	2.904	1
Taf4a	ENSMUSG00000039117	chr2	179646851	179711351	-	4633	2.903	1
Gm7964	ENSMUSG00000063902	chr7	90904414	90905866	+	1392	2.902	1
Tra2a	ENSMUSG00000029817	chr6	49193920	49214051	-	3066	2.902	1
Hmgcr	ENSMUSG00000021670	chr13	97418922	97440891	-	7275	2.902	1
Il1rap	ENSMUSG00000022514	chr16	26581790	26730203	+	11360	2.901	1
Hltf	ENSMUSG00000002428	chr3	19957811	20018376	+	5931	2.897	1

Tmtc3	ENSMUSG00000036676	chr10	99906536	99949984	-	8939	2.897	1
Tmem184c	ENSMUSG00000031617	chr8	80119881	80134597	-	2723	2.896	1
Atp2b1	ENSMUSG00000019943	chr10	98377786	98488777	+	7145	2.896	1
Bcl2l11	ENSMUSG00000027381	chr2	127951774	127988283	+	8029	2.895	1
Mettl10	ENSMUSG00000030960	chr7	140019140	140044356	-	1764	2.895	1
Ppp1r3d	ENSMUSG00000049999	chr2	178145911	178149177	-	3267	2.894	1
Gorab	ENSMUSG00000040124	chr1	165315040	165333772	-	2509	2.894	1
Mapkapk3	ENSMUSG00000032577	chr9	107157258	107192208	-	5542	2.892	1
Ints8	ENSMUSG00000040738	chr4	11126305	11181405	-	7472	2.891	1
Cyfip1	ENSMUSG00000030447	chr7	63097115	63186070	+	8925	2.89	1
Sik1	ENSMUSG00000024042	chr17	31981193	31992737	-	4511	2.89	1
Snx4	ENSMUSG00000022808	chr16	33251542	33299648	+	2442	2.887	1
Cnr2	ENSMUSG00000062585	chr4	135451309	135476122	+	3982	2.886	1
Siah1b	ENSMUSG00000040749	chrX	160508637	160514425	-	2119	2.886	1
Heca	ENSMUSG00000039879	chr10	17588432	17667873	-	4689	2.885	1
Snx14	ENSMUSG00000032422	chr9	88271585	88333796	-	3959	2.885	1
Plce1	ENSMUSG00000024998	chr19	38598687	38859588	+	9549	2.884	1
Nxt2	ENSMUSG00000042271	chrX	138661313	138674235	+	3176	2.882	1
Gpbp1	ENSMUSG00000032745	chr13	112215888	112280319	-	3905	2.881	1
Baalc	ENSMUSG00000022296	chr15	38764688	38782810	+	2495	2.88	1
Stard9	ENSMUSG00000033705	chr2	120454862	120557633	+	19391	2.879	1
Lancl2	ENSMUSG00000062190	chr6	57652595	57689432	+	2958	2.877	1
Taf9b	ENSMUSG00000047242	chrX	103402222	103416497	-	3158	2.877	1
Slfn2	ENSMUSG00000072620	chr11	82878614	82884180	+	1664	2.876	1
Dcaf13	ENSMUSG00000022300	chr15	38944420	38978402	+	1542	2.875	1
Fnbp4	ENSMUSG00000008200	chr2	90585527	90621178	+	6583	2.875	1
Hoxa6	ENSMUSG00000043219	chr6	52155430	52161355	-	2013	2.874	1
Atrx	ENSMUSG00000031229	chrX	102992954	103124736	-	15190	2.874	1
Rdx	ENSMUSG00000032050	chr9	51855255	51896842	+	4841	2.874	1
Tmem2	ENSMUSG00000024754	chr19	21852832	21932817	+	6667	2.873	1
Heg1	ENSMUSG00000075254	chr16	33684552	33771662	+	12632	2.873	1
Ythdc2	ENSMUSG00000034653	chr18	44988319	45049374	+	6299	2.872	1
Loxl4	ENSMUSG00000025185	chr19	42668472	42687303	-	4215	2.87	1
Creb1	ENSMUSG00000025958	chr1	64579378	64651121	+	8513	2.869	1
Abhd2	ENSMUSG00000039202	chr7	86418087	86510394	+	10571	2.869	1
Nrp1	ENSMUSG00000025810	chr8	130882973	131029362	+	5907	2.868	1
Usp34	ENSMUSG00000056342	chr11	23206916	23389744	+	17637	2.868	1
Ezh2	ENSMUSG00000029687	chr6	47480273	47545340	-	6197	2.868	1
AF251705	ENSMUSG00000044811	chr11	114858035	114863194	-	1275	2.867	1
Atp1a3	ENSMUSG00000040907	chr7	25763186	25790976	-	5972	2.863	1
Myh10	ENSMUSG00000020900	chr11	68505061	68630134	+	10458	2.861	1
Noc3l	ENSMUSG00000024999	chr19	38862618	38893727	-	3977	2.861	1
Zfx	ENSMUSG00000079509	chrX	91319970	91369041	-	7759	2.859	1
Mtpn	ENSMUSG00000029840	chr6	35458906	35489888	-	3960	2.858	1

Usp53	ENSMUSG00000039701	chr3	122636519	122687422	-	5797	2.858	1
Ptprg	ENSMUSG00000021745	chr14	12386046	13074555	+	10917	2.855	1
Sulf2	ENSMUSG00000006800	chr2	165898589	165981163	-	5818	2.854	1
Garnl3	ENSMUSG00000038860	chr2	32841886	32987174	-	12210	2.854	1
Fer	ENSMUSG00000000127	chr17	64213364	64488843	+	4438	2.852	1
Zfp738	ENSMUSG00000048280	chr13	67768374	67788008	-	5217	2.851	1
Rab2b	ENSMUSG00000022159	chr14	52881434	52899220	-	4163	2.851	1
Kcnk2	ENSMUSG00000037624	chr1	191031816	191167711	-	3618	2.849	1
Zdhhc21	ENSMUSG00000028403	chr4	82444642	82505862	-	12972	2.849	1
Snx2	ENSMUSG00000034484	chr18	53336019	53380514	+	2013	2.847	1
Il2rg	ENSMUSG00000031304	chrX	98459717	98463594	-	2387	2.847	1
Fam65b	ENSMUSG00000036006	chr13	24674058	24825685	+	11662	2.847	1
Gm5528	ENSMUSG00000060125	chr1	72050862	72051344	+	483	2.846	1
Gpx8	ENSMUSG00000021760	chr13	113832971	113836596	-	1016	2.843	1
Ivns1abp	ENSMUSG00000023150	chr1	153191628	153211575	+	5037	2.843	1
1110059G10Rik	ENSMUSG00000032551	chr9	122854207	122860118	-	3361	2.842	1
Morf4l2	ENSMUSG00000031422	chrX	133267481	133278229	-	3135	2.841	1
Rod1	ENSMUSG00000028382	chr4	59484740	59562236	-	7480	2.84	1
Mxi1	ENSMUSG00000025025	chr19	53384996	53450300	+	5619	2.839	1
Ftsjd1	ENSMUSG00000046441	chr8	112741860	112748389	+	3577	2.838	1
Arih1	ENSMUSG00000025234	chr9	59236065	59334425	-	7707	2.837	1
Itch	ENSMUSG00000027598	chr2	154959245	155052591	+	9724	2.836	1
Gm9847	ENSMUSG00000050974	chr12	14501367	14501963	+	597	2.835	1
Tnfrsf21	ENSMUSG00000023915	chr17	43153504	43226137	+	3626	2.834	1
Exoc1	ENSMUSG00000036435	chr5	76958336	76999319	+	9158	2.834	1
Zfp71-rs1	ENSMUSG00000071281	chr13	67806243	67830056	-	4124	2.834	1
Rbmxl1	ENSMUSG00000037070	chr8	81029170	81032827	-	1723	2.834	1
Zfp942	ENSMUSG00000071267	chr17	22063927	22099431	-	5259	2.833	1
E2f1	ENSMUSG00000027490	chr2	154385143	154395628	-	3155	2.832	1
Adm	ENSMUSG00000030790	chr7	117771175	117773331	+	1387	2.829	1
D630037F22Rik	ENSMUSG00000038122	chr10	55734104	55948495	-	7235	2.826	1
Arhgap18	ENSMUSG00000039031	chr10	26473227	26638454	+	3884	2.824	1
Jhdm1d	ENSMUSG00000042599	chr6	39086622	39156788	-	10057	2.824	1
Ceacam10	ENSMUSG00000054169	chr7	25562223	25569668	+	1043	2.823	1
Ccnj	ENSMUSG00000025010	chr19	40905769	40923062	+	4355	2.822	1
C1galt1c1	ENSMUSG00000048970	chrX	35983962	35988264	-	1449	2.821	1
Cc2d2a	ENSMUSG00000039765	chr5	44053618	44132211	+	6776	2.82	1

Timp2	ENSMUSG00000017466	chr11	118162383	118217054	-	3896	2.819	1
Senp1	ENSMUSG00000033075	chr15	97871660	97923836	-	6039	2.819	1
Tgif1	ENSMUSG00000047407	chr17	71193545	71202886	-	3761	2.819	1
St8sia4	ENSMUSG00000040710	chr1	97484261	97564171	-	5411	2.818	1
Itgb2	ENSMUSG00000000290	chr10	76992997	77028453	+	3052	2.818	1
Col18a1	ENSMUSG00000001435	chr10	76514923	76629293	-	7021	2.817	1
Rbm7	ENSMUSG00000042396	chr9	48296814	48303413	-	1776	2.816	1
Ccdc109b	ENSMUSG00000027994	chr3	129617878	129673124	-	1318	2.816	1
Rgs19	ENSMUSG00000002458	chr2	181423124	181428682	-	2230	2.815	1
Cfh	ENSMUSG00000026365	chr1	141982432	142079988	-	6852	2.815	1
Nefl	ENSMUSG00000022055	chr14	68701920	68707152	+	3380	2.814	1
6430527G18Rik	ENSMUSG00000034168	chr12	88221653	88225764	-	4112	2.813	1
Arhgef9	ENSMUSG00000025656	chrX	92244276	92392160	-	6053	2.813	1
Brd1	ENSMUSG00000022387	chr15	88517730	88564663	-	8138	2.811	1
Smek1	ENSMUSG00000041846	chr12	102277619	102321912	-	4594	2.81	1
Cpeb4	ENSMUSG00000020300	chr11	31772211	31835634	+	6393	2.809	1
Hnrnpf	ENSMUSG00000042079	chr6	117850358	117875639	+	2813	2.809	1
Nbea	ENSMUSG00000027799	chr3	55429127	55987623	-	11147	2.808	1
Riok3	ENSMUSG00000024404	chr18	12287359	12315876	+	3661	2.807	1
Arhgap30	ENSMUSG00000048865	chr1	173319085	173340429	+	5191	2.806	1
Cnnm4	ENSMUSG00000037408	chr1	36528442	36565621	+	4582	2.805	1
Pgf	ENSMUSG00000004791	chr12	86507589	86518246	-	1582	2.805	1
Swap70	ENSMUSG00000031015	chr7	117365217	117427017	+	4032	2.804	1
Wdr43	ENSMUSG00000041057	chr17	71965555	72008371	+	3450	2.803	1
Cbwd1	ENSMUSG00000024878	chr19	24994917	25036048	-	1375	2.803	1
Nexn	ENSMUSG00000039103	chr3	151899950	151929284	-	2562	2.802	1
Il21r	ENSMUSG00000030745	chr7	132746943	132777084	+	2607	2.802	1
Mib1	ENSMUSG00000024294	chr18	10725546	10818702	+	10369	2.8	1
Myo7a	ENSMUSG00000030761	chr7	105199570	105268034	-	8524	2.8	1
Rgnef	ENSMUSG00000021662	chr13	98668550	98976120	-	6289	2.798	1
Fstl1	ENSMUSG00000022816	chr16	37776959	37836600	+	4914	2.798	1
Magi2	ENSMUSG00000040003	chr5	18732864	20210616	+	7748	2.797	1
Ubxn7	ENSMUSG00000053774	chr16	32332338	32393833	+	10318	2.795	1
Zbtb26	ENSMUSG00000050714	chr2	37287688	37298641	-	5267	2.794	1
Cd1d1	ENSMUSG00000028076	chr3	86799756	86803363	-	2570	2.793	1
Tnfsf14	ENSMUSG00000005824	chr17	57328915	57333612	-	1859	2.793	1
Acer3	ENSMUSG00000030760	chr7	105362168	105469718	-	4319	2.792	1
Mapk8	ENSMUSG00000021936	chr14	34191084	34260344	-	9255	2.792	1
Fgd5	ENSMUSG00000034037	chr6	91928872	92025998	+	6295	2.79	1
Abcg1	ENSMUSG00000024030	chr17	31194643	31252722	+	4066	2.79	1
Epn2	ENSMUSG00000001036	chr11	61330751	61393187	-	5226	2.79	1
B3galtl	ENSMUSG00000051950	chr5	150480805	150565174	+	4798	2.788	1
Siah1a	ENSMUSG00000036840	chr8	89247839	89269905	-	2107	2.788	1

Smurf2	ENSMUSG00000018363	chr11	106681380	106782029	-	6162	2.788	1
Wdr37	ENSMUSG00000021147	chr13	8802214	8871155	-	8729	2.787	1
AI98794<br>4	ENSMUSG00000056383	chr7	48629142	48648630	-	1822	2.786	1
Fgd4	ENSMUSG00000022788	chr16	16417010	16600642	-	17511	2.785	1
Mterfd3	ENSMUSG00000049038	chr10	84582181	84590746	-	1538	2.785	1
Egfr	ENSMUSG00000020122	chr11	16652206	16818161	+	11326	2.784	1
Dtx4	ENSMUSG00000039982	chr19	12540831	12576486	-	5765	2.782	1
Hivep1	ENSMUSG00000021366	chr13	42147390	42280395	+	8753	2.782	1
Was	ENSMUSG00000031165	chrX	7658579	7667624	-	2381	2.782	1
Arhgdib	ENSMUSG00000030220	chr6	136872044	136890420	-	1839	2.782	1
Aim1	ENSMUSG00000019866	chr10	43670113	43724652	-	7416	2.781	1
Tmem3<br>0a	ENSMUSG00000032328	chr9	79616750	79641314	-	5646	2.78	1
Hspd1	ENSMUSG00000025980	chr1	55134679	55145087	-	2642	2.779	1
Zfp516	ENSMUSG00000058881	chr18	83080271	83174706	+	7817	2.778	1
Vrk1	ENSMUSG00000021115	chr12	107248473	107315620	+	3796	2.778	1
Cbl	ENSMUSG00000034342	chr9	43957345	44042129	-	5116	2.778	1
Cct8	ENSMUSG00000025613	chr16	87483571	87496118	-	3088	2.777	1
Tpm3-<br>rs7	ENSMUSG00000058126	chr14	113713830	113715935	+	2106	2.775	1
Iqgap3	ENSMUSG00000028068	chr3	87885973	87924970	+	5805	2.775	1
Senp6	ENSMUSG00000034252	chr9	79914710	79992760	+	7016	2.774	1
Kras	ENSMUSG00000030265	chr6	145165219	145198759	-	5349	2.773	1
5330426<br>P16Rik	ENSMUSG00000022639	chr16	50719410	50732862	-	3526	2.773	1
Fan1	ENSMUSG00000033458	chr7	71491644	71518981	-	5289	2.773	1
Pop1	ENSMUSG00000022325	chr15	34425059	34460403	+	3795	2.772	1
Colec12	ENSMUSG00000036103	chr18	9707646	9877993	+	3322	2.772	1
Ccnd2	ENSMUSG00000000184	chr6	127075797	127101066	-	5701	2.772	1
Btbd7	ENSMUSG00000041702	chr12	104022866	104116616	-	4298	2.772	1
Zc3h12c	ENSMUSG00000035164	chr9	51920090	51976216	-	6157	2.771	1
Nrip1	ENSMUSG00000048490	chr16	76287645	76374072	-	11457	2.77	1
Mmp2	ENSMUSG00000031740	chr8	95351227	95377316	+	3038	2.77	1
Dmxl2	ENSMUSG00000041268	chr9	54212965	54349567	-	13419	2.769	1
Btbd10	ENSMUSG00000038187	chr7	120459140	120512906	-	5801	2.768	1
Stxbp3a	ENSMUSG00000027882	chr3	108596094	108643444	-	5342	2.768	1
Trip11	ENSMUSG00000021188	chr12	103072253	103151477	-	10157	2.767	1
Apba1	ENSMUSG00000024897	chr19	23833366	24024087	+	6897	2.767	1
Cep152	ENSMUSG00000068394	chr2	125388824	125450849	-	5768	2.766	1
Iws1	ENSMUSG00000024384	chr18	32227388	32263985	+	9623	2.765	1
Lztfl1	ENSMUSG00000025245	chr9	123603534	123626743	-	9249	2.764	1
Oscar	ENSMUSG00000054594	chr7	3561415	3567759	-	1847	2.764	1
Gstcd	ENSMUSG00000028018	chr3	132644717	132754997	-	4731	2.764	1

Snx5	ENSMUSG00000027423	chr2	144075859	144096642	-	4177	2.764	1
Sdc2	ENSMUSG00000022261	chr15	32850478	32964485	+	3322	2.763	1
Ralgapa1	ENSMUSG00000021027	chr12	56703916	56922154	-	8015	2.763	1
Vav1	ENSMUSG00000034116	chr17	57418523	57468659	+	4893	2.763	1
Cpt1a	ENSMUSG00000024900	chr19	3323301	3385732	+	4300	2.762	1
Fpr1	ENSMUSG00000045551	chr17	18013435	18020904	-	1324	2.761	1
Gpm6b	ENSMUSG00000031342	chrX	162676843	162826920	+	5600	2.76	1
Cpsf6	ENSMUSG00000055531	chr10	116781729	116817071	-	10510	2.76	1
Arhgap21	ENSMUSG00000036591	chr2	20769546	20890508	-	9271	2.76	1
Myo9a	ENSMUSG00000039585	chr9	59598703	59776673	+	13034	2.758	1
Cap1	ENSMUSG00000028656	chr4	122536290	122563299	-	3494	2.757	1
Rprd1a	ENSMUSG00000040446	chr18	24643463	24688705	-	4297	2.756	1
P4ha1	ENSMUSG00000019916	chr10	58786044	58836052	+	4114	2.756	1
Kcnq1	ENSMUSG00000009545	chr7	150293159	150612955	+	3087	2.756	1
Gdpd2	ENSMUSG00000019359	chrX	97925212	97934234	+	4609	2.754	1
Ss18l1	ENSMUSG00000039086	chr2	179777214	179804906	+	4343	2.753	1
Tsc22d2	ENSMUSG00000027806	chr3	58219437	58270709	+	9003	2.753	1
Rspry1	ENSMUSG00000050079	chr8	97125855	97184175	+	6425	2.753	1
Etv4	ENSMUSG00000017724	chr11	101631056	101646685	-	3104	2.753	1
Yme1l1	ENSMUSG00000026775	chr2	23011889	23054780	+	8009	2.753	1
Ahnak2	ENSMUSG00000072812	chr12	114010405	114040868	-	14163	2.752	1
Cltc	ENSMUSG00000047126	chr11	86507853	86571067	-	8499	2.751	1
Zfp182	ENSMUSG00000054737	chrX	20606065	20639442	-	5733	2.751	1
Sec63	ENSMUSG00000019802	chr10	42481302	42552320	+	8791	2.751	1
Rab10	ENSMUSG00000020671	chr12	3247430	3309969	-	3511	2.75	1
Tmsb4x	ENSMUSG00000049775	chrX	163645025	163647247	-	1371	2.75	1
Anapc10	ENSMUSG00000036977	chr8	82235719	82301220	+	2802	2.749	1
Spry2	ENSMUSG00000022114	chr14	106291166	106296036	-	2085	2.748	1
Nup205	ENSMUSG00000038759	chr6	35127445	35197590	+	6401	2.748	1
Slc25a40	ENSMUSG00000054099	chr5	8422850	8454790	+	2777	2.748	1
Osbpl11	ENSMUSG00000022807	chr16	33185157	33243397	+	4520	2.747	1
Psmd14	ENSMUSG00000026914	chr2	61549751	61638433	+	2826	2.747	1
Pdk1	ENSMUSG00000006494	chr2	71711281	71741915	+	7779	2.746	1
Fmn1	ENSMUSG00000044042	chr2	113167893	113556924	+	15534	2.745	1
Arpc5	ENSMUSG00000008475	chr1	154613672	154622727	+	2276	2.745	1
N4bp2	ENSMUSG00000037795	chr5	66154760	66221347	+	9871	2.743	1
C030046E11Rik	ENSMUSG00000038658	chr19	29596772	29681319	+	10727	2.74	1
Tmf1	ENSMUSG00000030059	chr6	97105260	97129118	-	3465	2.739	1
March7	ENSMUSG00000026977	chr2	60047944	60088733	+	10391	2.739	1
Usp14	ENSMUSG00000047879	chr18	9995430	10045117	-	5974	2.738	1

Dcp2	ENSMUSG00000024472	chr18	44540154	44584623	+	8643	2.738	1
2700023E23Rik	ENSMUSG00000053656	chr5	74489085	74491430	+	2346	2.737	1
Myc	ENSMUSG00000022346	chr15	61816946	61821929	+	2717	2.736	1
Mtss1	ENSMUSG00000022353	chr15	58772799	58913581	-	5239	2.736	1
Cacna2d1	ENSMUSG00000040118	chr5	15440509	15880329	+	8389	2.734	1
Cnot2	ENSMUSG00000020166	chr10	115922217	116018567	-	3732	2.734	1
Fst	ENSMUSG00000021765	chr13	115242470	115249159	-	2687	2.733	1
Ptbp2	ENSMUSG00000028134	chr3	119421660	119486306	-	3364	2.732	1
Tlk2	ENSMUSG00000020694	chr11	105040121	105145273	+	8070	2.732	1
Nufip2	ENSMUSG00000037857	chr11	77499641	77555294	+	5679	2.731	1
Zfpm2	ENSMUSG00000022306	chr15	40486588	40936138	+	6376	2.731	1
2610008E11Rik	ENSMUSG00000060301	chr10	78527119	78560345	-	4229	2.73	1
Dkk2	ENSMUSG00000028031	chr3	131748256	131843257	+	3694	2.73	1
Cyth3	ENSMUSG00000018001	chr5	144383316	144471119	+	4682	2.729	1
Mcm3	ENSMUSG00000041859	chr1	20793055	20810359	-	2991	2.728	1
Svs2	ENSMUSG00000040132	chr2	164061665	164064202	-	1985	2.727	1
Nfasc	ENSMUSG00000026442	chr1	134461268	134638374	-	10378	2.725	1
Nr2c2	ENSMUSG00000005893	chr6	92041384	92123051	+	9152	2.724	1
Foxm1	ENSMUSG00000001517	chr6	128312985	128325911	+	5498	2.723	1
Rpf2	ENSMUSG00000038510	chr10	39943052	39966785	-	1830	2.723	1
Tnfrsf11b	ENSMUSG00000063727	chr15	54082174	54110039	-	2818	2.723	1
Dok1	ENSMUSG00000068335	chr6	82980928	82983465	-	1977	2.723	1
Tab2	ENSMUSG00000015755	chr10	7625451	7676028	-	6135	2.723	1
Kbtbd11	ENSMUSG00000055675	chr8	15011025	15033330	+	6970	2.723	1
Dock5	ENSMUSG00000044447	chr14	68369985	68551629	-	10335	2.721	1
Ccar1	ENSMUSG00000020074	chr10	62206676	62255116	-	4478	2.721	1
Rabgap1l	ENSMUSG00000026721	chr1	162149305	162723069	-	10614	2.72	1
Cab39	ENSMUSG00000036707	chr1	87690016	87748151	+	4146	2.72	1
Zfp27	ENSMUSG00000062040	chr7	30678352	30691591	-	4224	2.719	1
Sgol1	ENSMUSG00000023940	chr17	53814111	53828658	-	3810	2.719	1
Plxnd1	ENSMUSG00000030123	chr6	115904829	115945023	-	7192	2.715	1
Pde1b	ENSMUSG00000022489	chr15	103333465	103360483	+	4267	2.714	1
Mdga1	ENSMUSG00000043557	chr17	29964901	30107032	-	9395	2.713	1
Gbe1	ENSMUSG00000022707	chr16	70314194	70569961	+	5352	2.712	1
Pcdh12	ENSMUSG00000024440	chr18	38426746	38444055	-	5703	2.712	1
Rcan3	ENSMUSG00000059713	chr4	134968223	134989768	-	5111	2.712	1
Snai2	ENSMUSG00000022676	chr16	14705945	14709488	+	2031	2.712	1
Krit1	ENSMUSG00000000600	chr5	3803165	3847487	+	6181	2.711	1
Ctnna1	ENSMUSG00000037815	chr18	35278542	35414427	+	3698	2.71	1
Tor1aip2	ENSMUSG00000079252	chr1	157882856	157900866	+	3305	2.707	1

Pigw	ENSMUSG00000045140	chr11	84689817	84693787	-	2733	2.707		1
Zrsr2	ENSMUSG00000031370	chrX	160373375	160396593	-	7194	2.706		1
Kbtbd2	ENSMUSG00000059486	chr6	56727518	56747807	-	4083	2.706		1
Col6a1	ENSMUSG00000001119	chr10	76171537	76188913	-	4393	2.705		1
Nptn	ENSMUSG00000032336	chr9	58430047	58505762	+	6158	2.703		1
Tnfaip8l1	ENSMUSG00000044469	chr17	56301900	56313378	+	2379	2.702		1
Elmod2	ENSMUSG00000035151	chr8	85836921	85856385	-	10807	2.701		1
Sf3b1	ENSMUSG00000025982	chr1	55042013	55084322	-	6192	2.701		1
Rab18	ENSMUSG00000073639	chr18	6765198	6790320	+	2353	2.7		1
Adam10	ENSMUSG00000054693	chr9	70526804	70628036	+	4929	2.699		1
Gm11428	ENSMUSG00000069792	chr11	83517493	83519770	+	533	2.698		1
Slc9a2	ENSMUSG00000026062	chr1	40738557	40825730	+	3493	2.696		1
Xrn2	ENSMUSG00000027433	chr2	146838732	146903736	+	3981	2.696		1
Fam171a1	ENSMUSG00000050530	chr2	3031496	3145078	+	4442	2.694		1
Pitrm1	ENSMUSG00000021193	chr13	6547403	6579385	+	3524	2.692		1
Ctsl	ENSMUSG00000021477	chr13	64464522	64471614	-	1975	2.692		1
Chl1	ENSMUSG00000030077	chr6	103460870	103699671	+	7953	2.691		1
Spna2	ENSMUSG00000057738	chr2	29821080	29886971	+	11428	2.69		1
Usp38	ENSMUSG00000038250	chr8	83504633	83538805	-	4684	2.69		1
Sltm	ENSMUSG00000032212	chr9	70390666	70440044	+	3707	2.688		1
Slit3	ENSMUSG00000056427	chr11	34934726	35522009	+	6067	2.686		1
Cd97	ENSMUSG00000002885	chr8	86247150	86265225	-	3757	2.686		1
Fam49a	ENSMUSG00000020589	chr12	12268945	12383165	+	4846	2.686		1
Fkbp7	ENSMUSG00000002732	chr2	76501101	76511173	-	1485	2.684		1
Ddx10	ENSMUSG00000053289	chr9	52906559	53056217	-	3374	2.684		1
Prkd1	ENSMUSG00000002688	chr12	51442218	51750210	-	3778	2.683		1
4930581F22Rik	ENSMUSG00000070315	chr9	34924313	34938507	+	3462	2.681		1
Rrm2b	ENSMUSG00000022292	chr15	37853707	37891073	-	6437	2.681		1
Ttc9c	ENSMUSG00000071660	chr19	8883565	8893823	-	3984	2.681		1
Lgals3	ENSMUSG00000050335	chr14	47987426	48005835	+	1858	2.68		1
Fmnl3	ENSMUSG00000023008	chr15	99147656	99200913	-	5021	2.679		1
Igfbp7	ENSMUSG00000036256	chr5	77778265	77837070	-	1213	2.679		1
Dnm1l	ENSMUSG00000022789	chr16	16312328	16359123	-	4185	2.678		1
Ythdf3	ENSMUSG00000047213	chr3	16083183	16117035	+	5332	2.677		1
Tbl1xr1	ENSMUSG00000027630	chr3	21975574	22115516	+	8127	2.677		1
Kpna4	ENSMUSG00000027782	chr3	68871071	68931035	-	8940	2.675		1
Zfp68	ENSMUSG00000058291	chr5	139045844	139060989	-	3834	2.674		1
Arl5a	ENSMUSG00000036093	chr2	52253471	52280421	-	5409	2.673		1
Trf	ENSMUSG00000032554	chr9	103106331	103132774	-	3310	2.672		1
Pdzrn3	ENSMUSG00000035357	chr6	101099601	101327891	-	7258	2.672		1
Parp11	ENSMUSG00000037997	chr6	127396858	127444259	+	5720	2.672		1

Il17ra	ENSMUSG00000002897	chr6	120413215	120433745	+	3970	2.671	1
Creb3l2	ENSMUSG00000038648	chr6	37277255	37392146	-	7283	2.671	1
Ms4a7	ENSMUSG00000024672	chr19	11395529	11410636	-	4453	2.67	1
Kidins220	ENSMUSG00000036333	chr12	25659797	25746142	+	7462	2.67	1
Bbs12	ENSMUSG00000051444	chr3	37211476	37220375	+	2436	2.67	1
Pstpip1	ENSMUSG00000032322	chr9	55937769	55976695	+	3830	2.67	1
Ngly1	ENSMUSG00000021785	chr14	17081794	17144440	+	5274	2.669	1
Sgpl1	ENSMUSG00000020097	chr10	60561390	60610451	-	6143	2.669	1
Rasa4	ENSMUSG00000004952	chr5	136559786	136587730	+	6191	2.668	1
Steap4	ENSMUSG00000012428	chr5	7960472	7982213	+	3140	2.667	1
Pcgf3	ENSMUSG00000033623	chr5	108890251	108935995	+	9544	2.667	1
Sh3bgrl	ENSMUSG00000031246	chrX	106290704	106393212	+	3298	2.665	1
Syngr1	ENSMUSG00000022415	chr15	79921764	79949931	+	4808	2.665	1
D030025P21Rik	ENSMUSG00000092130	chr12	86216719	86219082	+	2364	2.665	1
2700007P21Rik	ENSMUSG00000027122	chr2	106802684	106814581	-	5313	2.664	1
Nup88	ENSMUSG00000040667	chr11	70756560	70783475	-	5974	2.663	1
Utrn	ENSMUSG00000019820	chr10	12101986	12589163	-	13231	2.663	1
Dock4	ENSMUSG00000035954	chr12	41172640	41573073	+	8100	2.662	1
Utp14b	ENSMUSG00000079470	chr1	78654613	78664163	+	3755	2.661	1
Itgal	ENSMUSG00000030830	chr7	134439774	134478651	+	6380	2.66	1
Cdc7	ENSMUSG00000029283	chr5	107393341	107413451	+	4135	2.66	1
Fam188a	ENSMUSG00000026767	chr2	12268890	12341096	-	3758	2.659	1
Chd7	ENSMUSG00000041235	chr4	8617553	8794806	+	13786	2.658	1
Nup155	ENSMUSG00000022142	chr15	8059313	8109857	+	6402	2.657	1
Vcpip1	ENSMUSG00000045210	chr1	9708703	9738463	-	9749	2.657	1
Zcchc2	ENSMUSG00000038866	chr1	107886983	107930651	+	6759	2.656	1
Pppde1	ENSMUSG00000026502	chr1	180117548	180182729	+	4453	2.654	1
Met	ENSMUSG00000009376	chr6	17413800	17523980	+	7720	2.653	1
Nus1	ENSMUSG00000023068	chr10	52137353	52159998	+	4876	2.653	1
Zfyve16	ENSMUSG00000021706	chr13	93257063	93300823	-	7082	2.653	1
Wfdc2	ENSMUSG00000017723	chr2	164387913	164394010	+	1140	2.652	1
Fgf13	ENSMUSG00000031137	chrX	56315322	56821248	-	4019	2.649	1
Pnpt1	ENSMUSG00000020464	chr11	29030744	29061828	+	3516	2.649	1
Mex3d	ENSMUSG00000048696	chr10	79843100	79850404	-	3266	2.647	1
Fkbp1b	ENSMUSG00000020635	chr12	4839981	4848401	-	979	2.644	1
Kdm6a	ENSMUSG00000037369	chrX	17739701	17857062	+	8158	2.644	1
Zfp326	ENSMUSG00000029290	chr5	106305584	106344837	+	7246	2.643	1
Zfp422	ENSMUSG00000059878	chr6	116574034	116579017	-	3625	2.643	1
Kctd18	ENSMUSG00000054770	chr1	58011945	58075800	-	12094	2.643	1
Sorbs2	ENSMUSG00000031626	chr8	46593148	46913259	+	19425	2.642	1
Atg12	ENSMUSG00000032905	chr18	46892071	46901233	-	2492	2.641	1
Prkx	ENSMUSG00000035725	chrX	75006750	75041617	-	5807	2.641	1

Bicd2	ENSMUSG00000037933	chr13	49436918	49482394	+	6351	2.64	1
D3Ertd254e	ENSMUSG00000033883	chr3	36058444	36065142	+	2388	2.639	1
N4bp2l1	ENSMUSG00000041132	chr5	151374218	151397100	-	1906	2.637	1
Lamc1	ENSMUSG00000026478	chr1	155066052	155179916	-	8272	2.635	1
Seh1l	ENSMUSG00000079614	chr18	67934530	67955115	+	3516	2.635	1
Fermt1	ENSMUSG00000027356	chr2	132729915	132771772	-	6048	2.634	1
Vsig2	ENSMUSG00000001943	chr9	37346840	37351790	+	1170	2.632	1
2810408A11Rik	ENSMUSG00000018570	chr11	69710854	69714489	-	2368	2.632	1
Ndrg4	ENSMUSG00000036564	chr8	98200880	98239019	+	4887	2.632	1
Kdr	ENSMUSG00000062960	chr5	76328852	76374483	-	7278	2.63	1
Ggh	ENSMUSG00000073987	chr4	19968857	19993897	+	1996	2.629	1
Rnf6	ENSMUSG00000029634	chr5	147020768	147033131	-	4022	2.628	1
Gimap4	ENSMUSG00000054435	chr6	48634548	48642061	+	1839	2.627	1
Zfp207	ENSMUSG00000017421	chr11	80196781	80219235	+	6658	2.625	1
9630033F20Rik	ENSMUSG00000038028	chr6	127035134	127059568	-	3651	2.623	1
Csnk1e	ENSMUSG00000022433	chr15	79248282	79274349	-	6410	2.622	1
Iqgap1	ENSMUSG00000030536	chr7	87856469	87948217	-	7359	2.622	1
Eif4e	ENSMUSG00000028156	chr3	138189155	138220563	+	2882	2.622	1
B3galnt2	ENSMUSG00000039242	chr13	14046941	14091335	+	3681	2.621	1
Smek2	ENSMUSG00000020463	chr11	29072890	29120797	+	5720	2.62	1
Slc38a2	ENSMUSG00000022462	chr15	96517825	96530129	-	4626	2.619	1
Ccnc	ENSMUSG00000028252	chr4	21654848	21687069	+	5115	2.619	1
Smarce1	ENSMUSG00000037935	chr11	99070361	99092331	-	6650	2.619	1
Zbtb34	ENSMUSG00000068966	chr2	33261628	33286844	-	6604	2.618	1
Msh6	ENSMUSG00000005370	chr17	88374390	88390223	+	4254	2.617	1
Wdr36	ENSMUSG00000038299	chr18	32996879	33026074	+	3281	2.617	1
Fip1l1	ENSMUSG00000029227	chr5	74931474	74994815	+	10442	2.617	1
Rapgef6	ENSMUSG00000037533	chr11	54336349	54512787	+	13051	2.616	1
Cd209b	ENSMUSG00000065987	chr8	3917657	3926844	-	2113	2.616	1
Spink2	ENSMUSG00000053030	chr5	77634132	77640496	-	788	2.616	1
Synj1	ENSMUSG00000022973	chr16	90936337	91011553	-	8089	2.616	1
B630019K06Rik	ENSMUSG00000052364	chrX	8469479	8472103	+	2625	2.615	1
Yod1	ENSMUSG00000046404	chr1	132613904	132618632	+	3825	2.615	1
Rapgef2	ENSMUSG00000062232	chr3	78866438	78985262	-	13785	2.614	1
Timeless	ENSMUSG00000039994	chr10	127669119	127689997	+	6117	2.613	1
Zfp800	ENSMUSG00000039841	chr6	28189931	28348005	-	4827	2.612	1

Mllt3	ENSMUSG00000028496	chr4	87415829	87679268	-	12004	2.611	1
Myo1f	ENSMUSG00000024300	chr17	33692664	33744709	+	4914	2.61	1
Plekha3	ENSMUSG00000002733	chr2	76513338	76534885	+	3126	2.61	1
Riok2	ENSMUSG00000023846	chr17	17511296	17531862	+	3375	2.61	1
Gtpbp4	ENSMUSG00000021149	chr13	8971317	8995263	-	2977	2.61	1
Tbc1d1	ENSMUSG00000029174	chr5	64550690	64742725	+	7140	2.609	1
Txnrd1	ENSMUSG00000020250	chr10	82296696	82360469	+	4019	2.609	1
Fmnl1	ENSMUSG00000055805	chr11	103032421	103060215	+	4868	2.608	1
Zc3h7a	ENSMUSG00000037965	chr16	11136685	11176486	-	9160	2.607	1
Samd9l	ENSMUSG00000047735	chr6	3322257	3349571	-	5425	2.607	1
B4galt5	ENSMUSG00000017929	chr2	167123944	167174683	-	4308	2.606	1
Chn1	ENSMUSG00000056486	chr2	73448717	73613403	-	5146	2.606	1
Mgat3	ENSMUSG00000042428	chr15	80004151	80045949	+	4665	2.606	1
Rab12	ENSMUSG00000023460	chr17	66843852	66869057	-	3021	2.606	1
Orc3	ENSMUSG00000040044	chr4	34518045	34562193	-	6088	2.604	1
Zfp655	ENSMUSG00000007812	chr5	145992592	146008175	+	4518	2.603	1
Kpnb1	ENSMUSG00000001440	chr11	97021028	97049195	-	6590	2.602	1
Lrrc8d	ENSMUSG00000046079	chr5	106128988	106261455	+	12573	2.602	1
Cd86	ENSMUSG00000022901	chr16	36603955	36666167	-	3767	2.6	1
Zfp451	ENSMUSG00000042197	chr1	33817317	33871440	-	19195	2.599	1
Lxn	ENSMUSG00000047557	chr3	67261920	67267848	-	1508	2.598	1
4933424B01Rik	ENSMUSG00000040250	chr6	146498154	146526357	-	3968	2.598	1
Spopl	ENSMUSG00000026771	chr2	23361740	23427626	-	6720	2.597	1
Arntl	ENSMUSG00000055116	chr7	120350979	120457636	+	2925	2.597	1
Hat1	ENSMUSG00000027018	chr2	71227015	71279679	+	2886	2.597	1
Gm16586	ENSMUSG00000089788	chr1	180251239	180254302	-	2640	2.596	1
Ar	ENSMUSG00000046532	chrX	95345060	95518554	+	9096	2.593	1
Shf	ENSMUSG00000033256	chr2	122174628	122194898	-	2305	2.592	1
Acsl5	ENSMUSG00000024981	chr19	55327859	55371117	+	3533	2.592	1
Rbbp8	ENSMUSG00000041238	chr18	11791785	11901716	+	3921	2.591	1
Katna1	ENSMUSG00000019794	chr10	7445798	7482948	+	2195	2.591	1
Atp1b3	ENSMUSG00000032412	chr9	96233099	96264766	-	1874	2.591	1
Magee1	ENSMUSG00000031227	chrX	102315735	102319248	+	3514	2.59	1
Cdkn2a	ENSMUSG00000044303	chr4	88920377	88940557	-	1170	2.59	1
Bace1	ENSMUSG00000032086	chr9	45646663	45672482	+	6348	2.589	1
Abcc5	ENSMUSG00000022822	chr16	20331376	20426467	-	10592	2.588	1
Gmps	ENSMUSG00000027823	chr3	63780028	63826501	+	11125	2.587	1
Zfp146	ENSMUSG00000037029	chr7	30946293	30954746	-	2071	2.587	1
Zfp788	ENSMUSG00000074165	chr7	48888573	48906904	+	4350	2.587	1
Bcl6	ENSMUSG00000022508	chr16	23965138	23988938	-	6700	2.586	1
Vdr	ENSMUSG00000022479	chr15	97684856	97741061	-	5650	2.585	1
Ng23	ENSMUSG00000036185	chr17	35162904	35164961	-	1028	2.585	1
Sipa1l2	ENSMUSG00000001995	chr8	127941963	128016610	-	6365	2.585	1

Polh	ENSMUSG00000023953	chr17	46308953	46339574	-	2569	2.584	1
Zcchc14	ENSMUSG00000061410	chr8	124122603	124176801	-	8031	2.583	1
Gm6190	ENSMUSG00000091139	chr12	89591799	89592682	-	774	2.582	1
Nfat5	ENSMUSG00000003847	chr8	109817370	109903417	+	18378	2.582	1
Dcaf17	ENSMUSG00000041966	chr2	70893385	70937199	+	9806	2.582	1
Opa1	ENSMUSG00000038084	chr16	29579420	29654970	+	7875	2.581	1
Hipk3	ENSMUSG00000027177	chr2	104266638	104334603	-	7987	2.579	1
Ccnb1	ENSMUSG00000041431	chr13	101548605	101556525	-	4217	2.579	1
Gdap2	ENSMUSG00000027865	chr3	99966304	100010904	+	3507	2.578	1
Cdh13	ENSMUSG00000031841	chr8	120807633	121848821	+	11153	2.578	1
Suv420h1	ENSMUSG00000045098	chr19	3767421	3818303	+	14173	2.577	1
Syap1	ENSMUSG00000031357	chrX	159294989	159326379	-	2351	2.577	1
Ece1	ENSMUSG00000057530	chr4	137418152	137521144	+	5300	2.575	1
Itga4	ENSMUSG00000027009	chr2	79095583	79173280	+	13978	2.574	1
Golga3	ENSMUSG00000029502	chr5	110605720	110655489	+	12671	2.574	1
Dhx9	ENSMUSG00000042699	chr1	155302888	155334790	-	4616	2.572	1
Ndrg1	ENSMUSG00000005125	chr15	66760883	66801202	-	6593	2.572	1
Gimap6	ENSMUSG00000047867	chr6	48651581	48658224	-	3496	2.571	1
Foxc1	ENSMUSG00000050295	chr13	31898515	31902504	+	3990	2.571	1
Rnf150	ENSMUSG00000047747	chr8	85387255	85615170	+	9879	2.571	1
Itga3	ENSMUSG00000001507	chr11	94905788	94938115	-	5710	2.571	1
Igj	ENSMUSG00000067149	chr5	88949537	88956916	-	1814	2.571	1
Agtpbp1	ENSMUSG00000021557	chr13	59550896	59686588	-	12835	2.57	1
Dnajc13	ENSMUSG00000032560	chr9	104053927	104165260	-	7712	2.57	1
Mettl4	ENSMUSG00000055660	chr17	95126420	95149359	-	3027	2.569	1
Bbs4	ENSMUSG00000025235	chr9	59169773	59201315	-	2542	2.568	1
Ddx50	ENSMUSG00000020076	chr10	62078540	62113966	-	2759	2.567	1
Erbb2ip	ENSMUSG00000021709	chr13	104608867	104710594	-	6591	2.566	1
Rnf141	ENSMUSG00000030788	chr7	117943946	117987971	-	5861	2.566	1
Dusp10	ENSMUSG00000039384	chr1	185837181	185899515	+	3042	2.566	1
Adssl1	ENSMUSG00000011148	chr12	113858258	113879565	+	1790	2.566	1
Hnrnpa1	ENSMUSG00000046434	chr15	103070945	103074445	+	1126	2.565	1
Etv3	ENSMUSG00000003382	chr3	87329329	87344078	+	10274	2.565	1
Vgll3	ENSMUSG00000091243	chr16	65815878	65863302	+	3322	2.564	1
Ttc7b	ENSMUSG00000033530	chr12	101538981	101759032	-	3441	2.564	1
Gng2	ENSMUSG00000043004	chr14	20691781	20796849	-	4390	2.564	1
Luzp1	ENSMUSG00000001089	chr4	136025676	136110695	+	8112	2.562	1
Nkrf	ENSMUSG00000044149	chrX	34427535	34442894	-	3268	2.561	1
Fv1	ENSMUSG00000070583	chr4	147243088	147244467	+	1380	2.561	1
Igkv5-39	ENSMUSG00000076569	chr6	69850418	69850704	-	287	2.561	1
Ddx21	ENSMUSG00000020075	chr10	62042996	62065046	-	4742	2.559	1

Cramp1l	ENSMUSG00000038002	chr17	25098173	25152175	-	7642	2.559	1
Ppp4r1	ENSMUSG00000061950	chr17	66131913	66191266	+	5022	2.557	1
Top1	ENSMUSG00000070544	chr2	160471624	160548500	+	10457	2.557	1
Usp27x	ENSMUSG00000046269	chrX	6948402	6952956	-	4277	2.556	1
Cep110	ENSMUSG00000057110	chr2	34965012	35034342	+	10424	2.554	1
Blm	ENSMUSG00000030528	chr7	87599879	87680005	-	4946	2.553	1
Ppid	ENSMUSG00000027804	chr3	79395264	79407572	+	4638	2.552	1
Zxdb	ENSMUSG00000073062	chrX	91969908	91975530	+	5623	2.552	1
Cdk8	ENSMUSG00000029635	chr5	147042806	147114450	+	3881	2.551	1
Edem3	ENSMUSG00000043019	chr1	153602504	153669458	+	6619	2.551	1
Dcun1d5	ENSMUSG00000032002	chr9	7184566	7207031	+	1201	2.55	1
Bcorl1	ENSMUSG00000036959	chrX	45694535	45761226	+	8516	2.546	1
Zfp90	ENSMUSG00000031907	chr8	108939239	108949628	+	2471	2.546	1
Snapc3	ENSMUSG00000028483	chr4	83063628	83113580	+	6760	2.546	1
Dnttip2	ENSMUSG00000039756	chr3	121977332	121988187	+	2401	2.545	1
Ifi47	ENSMUSG00000078920	chr11	48890089	48910476	+	2317	2.544	1
Ccnh	ENSMUSG00000021548	chr13	85329013	85363074	+	3105	2.542	1
Lphn3	ENSMUSG00000037605	chr5	81449163	82254158	+	13276	2.54	1
Crk	ENSMUSG00000017776	chr11	75492761	75520410	+	4730	2.54	1
Stard8	ENSMUSG00000031216	chrX	96198587	96270067	+	5499	2.54	1
Twf1	ENSMUSG00000022451	chr15	94408382	94420320	-	3054	2.54	1
Galnt4	ENSMUSG00000090035	chr10	98570793	98575881	+	5089	2.54	1
Samd4	ENSMUSG00000021838	chr14	47502529	47725492	+	8529	2.539	1
Klf6	ENSMUSG00000000078	chr13	5860735	5869639	+	4217	2.538	1
Krr1	ENSMUSG00000063334	chr10	111409720	111425488	+	5958	2.537	1
Csnk1a1	ENSMUSG00000024576	chr18	61714928	61749715	+	10502	2.535	1
Zfp143	ENSMUSG00000061079	chr7	117205216	117238906	+	3017	2.535	1
Pde5a	ENSMUSG00000053965	chr3	122432076	122562292	+	8859	2.534	1
Tmem71	ENSMUSG00000036944	chr15	66357774	66392608	-	2931	2.534	1
Vamp4	ENSMUSG00000026696	chr1	164500646	164529215	+	4565	2.534	1
Fpgt	ENSMUSG00000053870	chr3	154747885	154756342	-	3490	2.533	1
Dld	ENSMUSG00000020664	chr12	32016429	32036302	-	2241	2.533	1
Cd164	ENSMUSG00000019818	chr10	41239306	41250848	+	2888	2.533	1
Pafah1b1	ENSMUSG00000020745	chr11	74487451	74538172	-	9620	2.532	1
Hnrnpm	ENSMUSG00000059208	chr17	33783178	33823805	-	4709	2.532	1
Nipbl	ENSMUSG00000022141	chr15	8241225	8394463	-	8815	2.531	1
Hnrnpu	ENSMUSG00000039630	chr1	180253205	180267928	-	5946	2.531	1
Rem2	ENSMUSG00000022176	chr14	55094937	55099271	+	2254	2.529	1
Purb	ENSMUSG00000049647	chr11	6367609	6369141	-	1533	2.528	1
Tnfrsf13b	ENSMUSG00000010142	chr11	60940257	60962874	+	2836	2.528	1
Etv1	ENSMUSG00000004151	chr12	39505967	39594802	+	5889	2.528	1
Plin2	ENSMUSG00000028494	chr4	86294290	86315964	-	3512	2.528	1

Ep300	ENSMUSG00000055024	chr15	81415781	81482507	+	11234	2.527	1
1810013L24Rik	ENSMUSG00000022507	chr16	8830193	8859009	+	4066	2.527	1
Fcer1g	ENSMUSG00000058715	chr1	173159703	173164496	-	800	2.527	1
Akap12	ENSMUSG00000038587	chr10	5987073	6080212	-	6244	2.527	1
Ccdc104	ENSMUSG00000020462	chr11	29121532	29147409	-	2744	2.527	1
Stmn2	ENSMUSG00000027500	chr3	8509527	8561604	+	1904	2.527	1
Sfpq	ENSMUSG00000028820	chr4	126698568	126714257	+	4743	2.526	1
Vps26a	ENSMUSG00000020078	chr10	61917591	61949553	-	3252	2.526	1
Nfkbiz	ENSMUSG00000035356	chr16	55811488	55839012	-	5018	2.526	1
Zfp521	ENSMUSG00000024420	chr18	13845522	14131242	-	6182	2.525	1
Arl4a	ENSMUSG00000047446	chr12	40732034	40764612	-	7701	2.524	1
Acsl3	ENSMUSG00000032883	chr1	78654400	78703562	+	3601	2.523	1
Gfra1	ENSMUSG00000025089	chr19	58310094	58530399	-	5476	2.523	1
Farp1	ENSMUSG00000025555	chr14	121434422	121682966	+	5524	2.52	1
Qpct	ENSMUSG00000024084	chr17	79451246	79489583	+	1924	2.518	1
2310008H04Rik	ENSMUSG00000041974	chr16	15889320	16146944	-	3348	2.517	1
Tor1aip2	ENSMUSG00000050565	chr1	157882533	157915991	+	6611	2.516	1
Gm12824	ENSMUSG00000070867	chr4	114079329	114287703	+	6617	2.516	1
Csnk1g1	ENSMUSG00000032384	chr9	65756781	65892816	+	11973	2.515	1
Rbmx2	ENSMUSG00000031107	chrX	46048181	46063900	+	1872	2.515	1
Slfn10-ps	ENSMUSG00000072621	chr11	82841632	82853544	-	3621	2.515	1
Nhedc2	ENSMUSG00000037994	chr3	134970664	135005731	+	2877	2.515	1
Trrap	ENSMUSG00000045482	chr5	145528961	145620647	+	15185	2.514	1
Ndufs1	ENSMUSG00000025968	chr1	63190170	63223396	-	3773	2.513	1
Rad54l2	ENSMUSG00000040661	chr9	106590413	106691544	-	9324	2.513	1
Ccdc93	ENSMUSG00000026339	chr1	123327626	123403037	+	11445	2.513	1
Hoxb3	ENSMUSG00000048763	chr11	96184640	96207999	+	3006	2.512	1
Nup98	ENSMUSG00000063550	chr7	109268968	109358634	-	7082	2.512	1
Ifnar1	ENSMUSG00000022967	chr16	91485483	91507686	+	5879	2.511	1
Klhl20	ENSMUSG00000026705	chr1	163018506	163061642	-	8909	2.511	1
Slc30a9	ENSMUSG00000029221	chr5	67698194	67749804	+	9861	2.51	1
Ube2g1	ENSMUSG00000020794	chr11	72420785	72499983	+	4579	2.509	1
Stx7	ENSMUSG00000019998	chr10	23869123	23908765	+	2500	2.509	1
Cdkn1b	ENSMUSG00000003031	chr6	134870419	134875531	+	4552	2.509	1
Tpbg	ENSMUSG00000035274	chr9	85735987	85740662	+	3722	2.508	1
Cdk14	ENSMUSG00000028926	chr5	4803391	5420312	-	15347	2.508	1
Msl2	ENSMUSG00000066415	chr9	100978451	101007114	+	4908	2.508	1
Cep57	ENSMUSG00000031922	chr9	13612236	13631551	-	4180	2.508	1

Cerk	ENSMUSG00000035891	chr15	85969558	86016571	-	4812	2.507	1
Mgat4a	ENSMUSG00000026110	chr1	37496185	37597861	-	7371	2.506	1
Scaf11	ENSMUSG00000033228	chr15	96242129	96291274	-	7724	2.505	1
Msh5	ENSMUSG00000007035	chr17	35165550	35183690	-	5526	2.505	1
Zfp141	ENSMUSG00000092416	chr7	49728756	49761096	-	4665	2.504	1
Avl9	ENSMUSG00000029787	chr6	56664921	56711905	+	7463	2.504	1
Traf3	ENSMUSG00000021277	chr12	112404581	112505364	+	7429	2.503	1
Tgds	ENSMUSG00000022130	chr14	118511133	118531987	-	2265	2.503	1
Taf1	ENSMUSG00000031314	chrX	98728073	98797128	+	8434	2.503	1
Chordc1	ENSMUSG00000001774	chr9	18096711	18118444	+	2205	2.503	1
Zfp169	ENSMUSG00000050954	chr13	48583016	48608820	-	8017	2.503	1
Plp2	ENSMUSG00000031146	chrX	7245067	7248516	-	1334	2.5	1
Hmgn5	ENSMUSG00000031245	chrX	106199873	106208743	-	1934	2.499	1
Tug1	ENSMUSG00000056579	chr11	3539797	3548815	-	6672	2.499	1
Rbmx	ENSMUSG00000031134	chrX	54636707	54646213	-	4330	2.497	1
Stk39	ENSMUSG00000027030	chr2	68048502	68310325	-	4955	2.496	1
Sash1	ENSMUSG00000015305	chr10	8442017	8605868	-	7183	2.496	1
Zfp192	ENSMUSG00000063894	chr13	21605091	21622989	-	8902	2.496	1
Insig2	ENSMUSG00000003721	chr1	123200930	123229166	-	3473	2.495	1
Il13ra1	ENSMUSG00000017057	chrX	33652105	33711254	+	3715	2.495	1
Tiam2	ENSMUSG00000023800	chr17	3326573	3519397	+	6881	2.495	1
Mdm1	ENSMUSG00000020212	chr10	117578843	117606053	+	3149	2.494	1
Gm9766	ENSMUSG00000038594	chr10	53016060	53068936	-	1345	2.493	1
Tgs1	ENSMUSG00000028233	chr4	3502022	3543766	+	5314	2.493	1
Dgkd	ENSMUSG00000070738	chr1	89749862	89841064	+	5694	2.492	1
Gtf2e2	ENSMUSG00000031585	chr8	34842386	34887645	+	1802	2.491	1
Wdr47	ENSMUSG00000040389	chr3	108394197	108448637	+	4394	2.491	1
Rsu1	ENSMUSG00000026727	chr2	12998594	13193058	-	8990	2.49	1
Cep120	ENSMUSG00000048799	chr18	53841380	53904207	-	4719	2.487	1
Fkbp5	ENSMUSG00000024222	chr17	28536040	28654469	-	6316	2.486	1
Nf1	ENSMUSG00000020716	chr11	79153195	79395114	+	15820	2.485	1
Pafah1b2	ENSMUSG00000003131	chr9	45773394	45792954	-	4241	2.485	1
Fam43a	ENSMUSG00000046546	chr16	30599809	30602883	+	3075	2.485	1
Cpd	ENSMUSG00000020841	chr11	76591926	76660520	-	8332	2.485	1
Sgk1	ENSMUSG00000019970	chr10	21601990	21719709	+	4903	2.485	1
Zfp217	ENSMUSG00000052056	chr2	169934143	169973603	-	6139	2.484	1
Ttll5	ENSMUSG00000012609	chr12	87165609	87402843	+	15833	2.484	1
Dhfr	ENSMUSG00000021707	chr13	93124738	93159008	+	5290	2.483	1
Rassf2	ENSMUSG00000027339	chr2	131815151	131855994	-	11993	2.483	1
1110012J17Rik	ENSMUSG00000052105	chr17	66686322	66799090	-	9268	2.482	1
Polr3f	ENSMUSG00000027427	chr2	144353454	144367731	+	7710	2.482	1
Fbxo38	ENSMUSG00000042211	chr18	62663723	62708397	-	4388	2.48	1
Hiatl1	ENSMUSG00000038212	chr13	65165971	65214290	-	3925	2.478	1

Atf7	ENSMUSG00000052414	chr15	102364008	102455881	-	3785	2.477	1
Xpa	ENSMUSG00000028329	chr4	46168219	46209183	-	1894	2.475	1
Ctbp2	ENSMUSG00000030970	chr7	140179246	140316037	-	6220	2.474	1
Tacc1	ENSMUSG00000065954	chr8	26265024	26311921	-	7695	2.474	1
Ptprs	ENSMUSG00000013236	chr17	56551851	56615906	-	7050	2.473	1
Il16	ENSMUSG00000001741	chr7	90791358	90894236	-	7842	2.472	1
Stxbp5	ENSMUSG00000019790	chr10	9475345	9620877	-	12975	2.472	1
Rc3h2	ENSMUSG00000075376	chr2	37225589	37278423	-	11688	2.471	1
Ociad2	ENSMUSG00000029153	chr5	73713438	73730186	-	2509	2.471	1
Edn1	ENSMUSG00000021367	chr13	42396639	42403358	+	2344	2.47	1
Rheb	ENSMUSG00000028945	chr5	24308641	24348442	-	5540	2.47	1
Aldh1a2	ENSMUSG00000013584	chr9	71063596	71144050	+	2264	2.469	1
Ccdc41	ENSMUSG00000020024	chr10	94151535	94253081	+	2986	2.469	1
Tomm70a	ENSMUSG00000022752	chr16	57121827	57156818	+	5953	2.468	1
Tbc1d23	ENSMUSG00000022749	chr16	57168979	57231579	-	3648	2.468	1
Spty2d1	ENSMUSG00000049516	chr7	54245766	54263781	-	5223	2.468	1
Eif2s1	ENSMUSG00000021116	chr12	79962806	79987997	+	3405	2.467	1
Dyrk1a	ENSMUSG00000022897	chr16	94791617	94917124	+	8257	2.466	1
Sh3rf1	ENSMUSG00000031642	chr8	63702669	63874869	+	5582	2.465	1
Tjp1	ENSMUSG00000030516	chr7	72441051	72516125	-	7360	2.465	1
Fam76b	ENSMUSG00000037808	chr9	13632160	13650985	+	4325	2.465	1
Hccs	ENSMUSG00000031352	chrX	165688125	165758304	-	5151	2.463	1
Cpsf7	ENSMUSG00000034820	chr19	10599734	10622225	+	3604	2.461	1
Gins3	ENSMUSG00000031669	chr8	98157459	98168959	+	2412	2.46	1
Thumpd2	ENSMUSG00000024246	chr17	81425667	81464425	-	2022	2.46	1
Dcbld1	ENSMUSG00000019891	chr10	51953425	52041184	+	7282	2.459	1
2410089E03Rik	ENSMUSG00000039801	chr15	8119106	8221158	+	13017	2.456	1
D730040F13Rik	ENSMUSG00000055296	chr4	56879795	56960309	-	17165	2.455	1
Clock	ENSMUSG00000029238	chr5	76639992	76733817	-	8832	2.455	1
Bgn	ENSMUSG00000031375	chrX	70728941	70741272	+	3563	2.454	1
Eps15	ENSMUSG00000028552	chr4	108952873	109060422	+	6931	2.453	1
Dbn1	ENSMUSG00000034675	chr13	55574789	55589472	-	4515	2.453	1
Prkaa1	ENSMUSG00000050697	chr15	5093861	5131899	+	4655	2.453	1
Rbm33	ENSMUSG00000048271	chr5	28643661	28745784	+	10592	2.452	1
Dync1li1	ENSMUSG00000032435	chr9	114597908	114633420	+	2616	2.451	1
Usp8	ENSMUSG00000027363	chr2	126533064	126585033	+	4223	2.45	1
Ecm1	ENSMUSG00000028108	chr3	95538070	95543492	-	2521	2.449	1
Aif1	ENSMUSG00000024397	chr17	35307936	35313013	-	1532	2.449	1

Parp4	ENSMUSG00000054509	chr14	57194456	57278631	+	6534	2.449		1
Myo9b	ENSMUSG00000004677	chr8	73796613	73884611	+	7387	2.448		1
Tbxas1	ENSMUSG00000029925	chr6	38825403	39034584	+	9094	2.447		1
Neo1	ENSMUSG00000032340	chr9	58722486	58884248	-	7387	2.446		1
Synpo	ENSMUSG00000043079	chr18	60753627	60819796	-	9533	2.446		1
Usp31	ENSMUSG00000063317	chr7	128785535	128850767	-	10198	2.445		1
Sec61a2	ENSMUSG00000025816	chr2	5792033	5816478	-	3799	2.445		1
Carf	ENSMUSG00000026017	chr1	60155091	60207876	+	3314	2.444		1
4921513D23Rik	ENSMUSG00000060657	chr16	14110626	14159367	-	6474	2.444		1
Fubp3	ENSMUSG00000026843	chr2	31428171	31473046	+	4362	2.444		1
Papd4	ENSMUSG00000042167	chr13	93917355	93962238	-	1873	2.441		1
Rgl1	ENSMUSG00000026482	chr1	154363889	154613481	-	5590	2.441		1
Chic1	ENSMUSG00000031327	chrX	100551815	100591431	+	7375	2.438		1
Arid5b	ENSMUSG00000019947	chr10	67558341	67741474	-	4635	2.437		1
Fbxo8	ENSMUSG00000038206	chr8	59029887	59071190	+	3740	2.436		1
Entpd1	ENSMUSG00000048120	chr19	40686856	40816092	+	5580	2.435		1
2510003E04Rik	ENSMUSG00000036955	chr10	62001374	62041205	-	6547	2.434		1
Tbc1d15	ENSMUSG00000020130	chr10	114634933	114688549	-	3773	2.433		1
Mga	ENSMUSG00000033943	chr2	119722964	119795317	+	15340	2.433		1
Angpt2	ENSMUSG00000031465	chr8	18690263	18741562	-	3690	2.431		1
Rab8b	ENSMUSG00000036943	chr9	66691479	66767512	-	4743	2.43		1
Smc3	ENSMUSG00000024974	chr19	53674886	53720323	+	5545	2.429		1
Fam198b	ENSMUSG00000027955	chr3	79688455	79750202	+	7083	2.427		1
Hmox1	ENSMUSG00000005413	chr8	77617490	77624495	+	2161	2.427		1
B4galnt2	ENSMUSG00000013418	chr11	95727257	95776205	-	1722	2.426		1
Cpne1	ENSMUSG00000074643	chr2	155897581	155937695	-	6096	2.425		1
Usp47	ENSMUSG00000059263	chr7	119167020	119254900	+	5636	2.425		1
Azi2	ENSMUSG00000039285	chr9	117949617	117978912	+	6439	2.425		1
Slc43a3	ENSMUSG00000027074	chr2	84776736	84798666	+	4464	2.424		1
Podnl1	ENSMUSG00000012889	chr8	86649888	86656426	+	3549	2.422		1
Csda	ENSMUSG00000030189	chr6	131314876	131338468	-	1862	2.422		1
Cyp26b1	ENSMUSG00000063415	chr6	84521938	84543740	-	4602	2.421		1
Zfp266	ENSMUSG00000060510	chr9	20299512	20325861	-	6283	2.421		1
Secisbp2l	ENSMUSG00000035093	chr2	125562722	125608606	-	8233	2.42		1
Mapk1ip1l	ENSMUSG00000021840	chr14	47917966	47948460	+	4640	2.419		1
Pecam1	ENSMUSG00000020717	chr11	106515531	106611942	-	4314	2.417		1
Star	ENSMUSG00000031574	chr8	26918946	26925782	+	3374	2.414		1

Trnt1	ENSMUSG00000013736	chr6	106719114	106732468	+	3869	2.414	1
Nbeal1	ENSMUSG00000073664	chr1	60237443	60395172	+	16084	2.414	1
Nck1	ENSMUSG00000032475	chr9	100392712	100446472	-	4526	2.414	1
Plekho2	ENSMUSG00000050721	chr9	65402193	65427847	-	3271	2.414	1
Ranbp9	ENSMUSG00000038546	chr13	43498042	43576342	-	3089	2.414	1
Cidec	ENSMUSG00000030278	chr6	113374628	113385754	-	1856	2.414	1
Klhl9	ENSMUSG00000070923	chr4	88364196	88368369	-	4174	2.41	1
Cdc73	ENSMUSG00000026361	chr1	145445927	145550023	-	11966	2.409	1
F7	ENSMUSG00000031443	chr8	13026034	13035809	+	1859	2.409	1
Cbfa2t3	ENSMUSG00000006362	chr8	125152639	125223009	-	4775	2.408	1
Lysmd2	ENSMUSG00000032184	chr9	75473539	75485580	+	1200	2.408	1
Smad2	ENSMUSG00000024563	chr18	76401234	76465385	+	3549	2.407	1
Sepsecs	ENSMUSG00000029173	chr5	53031326	53060968	-	5347	2.406	1
Six1	ENSMUSG00000051367	chr12	74137916	74154874	-	8721	2.406	1
Usp15	ENSMUSG00000020124	chr10	122550286	122634017	-	3267	2.406	1
C1qtnf1	ENSMUSG00000017446	chr11	118289517	118311277	+	3131	2.405	1
Map2k4	ENSMUSG00000033352	chr11	65501745	65601799	-	4664	2.405	1
Ksr1	ENSMUSG00000018334	chr11	78826942	78959909	-	6750	2.403	1
Rbms3	ENSMUSG00000039607	chr9	116481864	117539031	-	10413	2.402	1
Akt3	ENSMUSG00000019699	chr1	178950204	179188334	-	4980	2.402	1
Pnn	ENSMUSG00000020994	chr12	60167906	60175004	+	3469	2.4	1
Dock1	ENSMUSG00000058325	chr7	141862370	142365322	+	6807	2.4	1
3110043O21Rik	ENSMUSG00000028300	chr4	35138534	35173424	-	4603	2.399	1
Arap2	ENSMUSG00000037999	chr5	62993684	63157437	-	8716	2.398	1
Ash1l	ENSMUSG00000028053	chr3	88769734	88883295	+	11268	2.398	1
Gm9917	ENSMUSG00000053666	chr9	107461141	107470746	-	5406	2.398	1
Eif3a	ENSMUSG00000024991	chr19	60837019	60866596	-	5176	2.397	1
Peli1	ENSMUSG00000020134	chr11	20991294	21050326	+	6553	2.396	1
Med23	ENSMUSG00000019984	chr10	24589792	24633487	+	6692	2.396	1
Mapre1	ENSMUSG00000027479	chr2	153567010	153599046	+	7811	2.396	1
Zfp846	ENSMUSG00000058192	chr9	20385769	20399638	+	2943	2.396	1
Robo4	ENSMUSG00000032125	chr9	37209482	37221608	+	4107	2.395	1
2610101N10Rik	ENSMUSG00000032407	chr9	95357313	95412415	-	7736	2.394	1
Tmem106a	ENSMUSG00000034947	chr11	101443556	101453102	+	3138	2.394	1
C130039O16Rik	ENSMUSG00000042507	chr12	85490126	85559831	-	7611	2.393	1
Zfp28	ENSMUSG00000062861	chr7	6336080	6347755	+	2502	2.393	1
Incenp	ENSMUSG00000024660	chr19	9946787	9974023	-	3229	2.392	1
Als2cr4	ENSMUSG00000038079	chr1	59157438	59177203	-	2076	2.391	1
Psrc1	ENSMUSG00000068744	chr3	108186757	108191149	+	3012	2.391	1

Api5	ENSMUSG00000027193	chr2	94251839	94278293	-	4545	2.391	1
Igf1r	ENSMUSG00000005533	chr7	75097713	75371484	+	4476	2.391	1
Hnrnpk	ENSMUSG00000021546	chr13	58492503	58504704	-	5634	2.39	1
Ndfip2	ENSMUSG00000053253	chr14	105657928	105708515	+	11415	2.389	1
Zcchc4	ENSMUSG00000029179	chr5	53166648	53215904	+	10890	2.388	1
Mfsd8	ENSMUSG00000025759	chr3	40621992	40650807	-	3184	2.388	1
Nfam1	ENSMUSG00000058099	chr15	82828151	82863736	-	3222	2.387	1
Pum2	ENSMUSG00000020594	chr12	8680940	8759387	+	7260	2.385	1
Twsg1	ENSMUSG00000024098	chr17	66272406	66300527	-	4046	2.384	1
Strap	ENSMUSG00000030224	chr6	137683599	137700453	+	3090	2.384	1
Rock1	ENSMUSG00000024290	chr18	10064399	10181790	-	6187	2.383	1
Mll5	ENSMUSG00000029004	chr5	22940259	23010053	+	7494	2.383	1
Lyz2	ENSMUSG00000069516	chr10	116714390	116719377	-	1313	2.381	1
Lrch1	ENSMUSG00000068015	chr14	75154480	75347684	-	5747	2.381	1
Psmd12	ENSMUSG00000020720	chr11	107340798	107365676	+	2649	2.381	1
Lpar6	ENSMUSG00000033446	chr14	73637698	73640165	+	2468	2.381	1
Igf1	ENSMUSG00000020053	chr10	87321010	87399792	+	9921	2.38	1
Gm16372	ENSMUSG00000057160	chr12	25178072	25178521	-	450	2.38	1
Adamts2	ENSMUSG00000036545	chr11	50415586	50621075	+	9336	2.379	1
Pak2	ENSMUSG00000022781	chr16	32016376	32079428	-	6795	2.378	1
Il17rd	ENSMUSG00000040717	chr14	27852187	27920472	+	9162	2.378	1
Nt5dc2	ENSMUSG00000071547	chr14	31948039	31952307	+	1407	2.377	1
Lrmp	ENSMUSG00000030263	chr6	145064173	145123454	+	4070	2.376	1
Rap1b	ENSMUSG00000052681	chr10	117251527	117282991	-	2024	2.374	1
Sccpdh	ENSMUSG00000038936	chr1	181598341	181617320	+	5213	2.372	1
Rftn1	ENSMUSG00000039316	chr17	50131582	50329999	-	4504	2.372	1
Cttn	ENSMUSG00000031078	chr7	151621638	151656914	-	4192	2.371	1
Csf1r	ENSMUSG00000024621	chr18	61265226	61291803	+	5229	2.371	1
Gm10567	ENSMUSG00000073738	chr1	9950252	9952890	-	2639	2.37	1
Fhl2	ENSMUSG00000008136	chr1	43179919	43220806	-	1563	2.37	1
Lats2	ENSMUSG00000021959	chr14	58260914	58377225	-	7303	2.37	1
Zcchc6	ENSMUSG00000035248	chr13	59873240	59924508	-	5551	2.368	1
2210018M11Rik	ENSMUSG00000035401	chr7	105739125	105805079	-	4047	2.368	1
Akirin1	ENSMUSG00000023075	chr4	123411802	123427588	-	3450	2.368	1
Fbxo34	ENSMUSG00000037536	chr14	48092236	48151637	+	3726	2.368	1
Fubp1	ENSMUSG00000028034	chr3	151873422	151899794	+	6599	2.367	1
Vtcn1	ENSMUSG00000051076	chr3	100629382	100699602	+	1379	2.366	1
Phip	ENSMUSG00000032253	chr9	82759766	82869096	-	10743	2.366	1
Tbc1d12	ENSMUSG00000048720	chr19	38911069	38994413	+	4209	2.365	1
Cnih	ENSMUSG00000015759	chr14	47395257	47408086	-	1570	2.365	1
Snhg4	ENSMUSG00000090430	chr18	35713064	35717970	+	1151	2.364	1

Emr1	ENSMUSG00000004730	chr17	57498109	57622952	+	3245	2.363	1
Ddx3x	ENSMUSG00000000787	chrX	12858096	12871178	+	5277	2.363	1
Zfp719	ENSMUSG00000030469	chr7	50834980	50848605	+	4706	2.362	1
Nampt	ENSMUSG00000020572	chr12	33505200	33538234	+	4547	2.362	1
Ism1	ENSMUSG00000074766	chr2	139503914	139584317	+	2944	2.361	1
Mynn	ENSMUSG00000037730	chr3	30501009	30517414	+	4152	2.361	1
Pus10	ENSMUSG00000020280	chr11	23565674	23632876	+	4325	2.36	1
Cept1	ENSMUSG00000040774	chr3	106305178	106350720	-	3116	2.36	1
Atp13a3	ENSMUSG00000022533	chr16	30312509	30388923	-	9559	2.36	1
Erg	ENSMUSG00000040732	chr16	95580776	95808200	-	3466	2.36	1
Chst2	ENSMUSG00000033350	chr9	95301348	95307689	-	6239	2.36	1
Eif2ak4	ENSMUSG00000005102	chr2	118214353	118300970	+	7458	2.359	1
Ccdc88b	ENSMUSG00000047810	chr19	6919113	6932701	-	4995	2.359	1
Cnot7	ENSMUSG00000031601	chr8	41577894	41601201	-	5821	2.359	1
Ankrd17	ENSMUSG00000055204	chr5	90656215	90795211	-	10054	2.356	1
Ddx52	ENSMUSG00000020677	chr11	83755564	83776590	+	3332	2.355	1
2810403A07Rik	ENSMUSG00000028060	chr3	88489716	88516855	+	3776	2.354	1
Pole2	ENSMUSG00000020974	chr12	70302766	70329177	-	1695	2.354	1
Helb	ENSMUSG00000020228	chr10	119520664	119550043	-	5011	2.354	1
Cdkn2aip	ENSMUSG00000038069	chr8	48794698	48799285	-	3494	2.354	1
Klhl24	ENSMUSG00000062901	chr16	20097627	20127817	+	6683	2.35	1
P4ha3	ENSMUSG00000051048	chr7	107434030	107468209	+	3043	2.35	1
Xrcc4	ENSMUSG00000021615	chr13	89913632	90229213	-	4213	2.349	1
4931428F04Rik	ENSMUSG00000014837	chr8	107804310	107813428	-	3552	2.349	1
Fgfr3	ENSMUSG00000054252	chr5	34064373	34079716	+	6880	2.349	1
1700066M21Rik	ENSMUSG00000038323	chr1	57434464	57442267	+	3008	2.348	1
Fbxo28	ENSMUSG00000047539	chr1	184243233	184271737	-	4913	2.347	1
Sc4mol	ENSMUSG00000031604	chr8	67196936	67212589	-	6144	2.347	1
Prex1	ENSMUSG00000039621	chr2	166391842	166539332	-	10266	2.346	1
Lilra5	ENSMUSG00000070873	chr7	4189356	4195065	+	2109	2.346	1
Kif3a	ENSMUSG00000018395	chr11	53380881	53417746	+	5869	2.346	1
Anp32e	ENSMUSG00000015749	chr3	95733169	95751313	+	3566	2.346	1
Grb10	ENSMUSG00000020176	chr11	11830511	11938686	-	9164	2.345	1
Stxbp1	ENSMUSG00000026797	chr2	32643122	32702759	-	4306	2.345	1
Midn	ENSMUSG00000035621	chr10	79611017	79621113	+	4307	2.344	1
Abi2	ENSMUSG00000026782	chr1	60466539	60537999	+	5890	2.344	1
Atxn1	ENSMUSG00000046876	chr13	45650262	46060345	-	5431	2.343	1
Prkdc	ENSMUSG00000022672	chr16	15637959	15842332	+	12674	2.343	1
Clip2	ENSMUSG00000063146	chr5	134965256	135028304	-	4995	2.342	1

Zfp191	ENSMUSG00000051469	chr18	24168203	24179311	-	6271	2.341	1
Flnc	ENSMUSG00000068699	chr6	29383153	29411888	+	9543	2.341	1
Taf2	ENSMUSG00000037343	chr15	54846684	54903707	-	5033	2.34	1
Cnksr3	ENSMUSG00000015202	chr10	3134304	3227478	+	3837	2.34	1
Zfp238	ENSMUSG00000063659	chr1	179374792	179380891	+	5102	2.34	1
Fam117b	ENSMUSG00000041040	chr1	59969850	60042190	+	5690	2.339	1
Snx6	ENSMUSG00000005656	chr12	55847336	55896690	-	1928	2.339	1
Zw10	ENSMUSG00000032264	chr9	48863737	48886877	+	2844	2.338	1
Hk1	ENSMUSG00000037012	chr10	61731603	61842656	-	8532	2.338	1
Man2a2	ENSMUSG00000038886	chr7	87495300	87516072	-	5048	2.338	1
Magi1	ENSMUSG00000045095	chr6	93625449	94233316	-	7883	2.337	1
Hoxa5	ENSMUSG00000038253	chr6	52151753	52154586	-	1877	2.337	1
Rnf13	ENSMUSG00000036503	chr3	57539993	57639347	+	4059	2.335	1
Svs4	ENSMUSG00000016998	chr2	164101688	164104195	-	731	2.335	1
Aste1	ENSMUSG00000032567	chr9	105297722	105308088	+	3284	2.335	1
Relt	ENSMUSG00000008318	chr7	107994362	108011960	-	3279	2.331	1
Zfp281	ENSMUSG00000041483	chr1	138521478	138526630	+	4937	2.331	1
Mll1	ENSMUSG00000002028	chr9	44611438	44689379	-	16670	2.331	1
Eif1ax	ENSMUSG00000067194	chrX	155810110	155827860	+	5266	2.33	1
Nr3c1	ENSMUSG00000024431	chr18	39570199	39650955	-	7425	2.327	1
Gabpb1	ENSMUSG00000027361	chr2	126453178	126502073	-	7752	2.326	1
Dcstamp	ENSMUSG00000022303	chr15	39577478	39592480	+	1993	2.325	1
Setdb2	ENSMUSG00000071350	chr14	60020846	60059721	-	4047	2.323	1
Etnk1	ENSMUSG00000030275	chr6	143115750	143157062	+	6429	2.323	1
Ahsa2	ENSMUSG00000020288	chr11	23387882	23398030	-	4532	2.322	1
Zfp7	ENSMUSG00000033669	chr15	76709689	76722822	+	2769	2.322	1
1110020G09Rik	ENSMUSG00000022253	chr15	9001009	9040244	+	3731	2.322	1
Kctd3	ENSMUSG00000026608	chr1	190794977	190831719	-	3703	2.32	1
Usp6nl	ENSMUSG00000039046	chr2	6243713	6367436	+	8059	2.319	1
Lipa	ENSMUSG00000024781	chr19	34566808	34601964	-	2969	2.318	1
Pcf11	ENSMUSG00000041328	chr7	99792222	99818443	-	6412	2.318	1
Slc39a14	ENSMUSG00000022094	chr14	70703274	70751232	-	10552	2.316	1
Plcb2	ENSMUSG00000040061	chr2	118533253	118554174	-	6597	2.313	1
Far1	ENSMUSG00000030759	chr7	120657348	120715025	+	8055	2.312	1
Kcnk6	ENSMUSG00000046410	chr7	30006947	30017541	-	4150	2.311	1
Ccl11	ENSMUSG00000020676	chr11	81871325	81876457	+	1082	2.308	1
Cdk5r1	ENSMUSG00000048895	chr11	80290548	80294681	+	4134	2.308	1
Pcnt	ENSMUSG00000001151	chr10	75813999	75905657	-	9530	2.308	1
Prim1	ENSMUSG00000025395	chr10	127452228	127467093	+	3864	2.308	1
Prpf18	ENSMUSG00000039449	chr2	4543104	4573159	-	5228	2.306	1
Ythdc1	ENSMUSG00000035851	chr5	87233246	87265684	+	10352	2.306	1
Cat	ENSMUSG00000027187	chr2	103294006	103325317	-	2613	2.306	1
Idh3a	ENSMUSG00000032279	chr9	54434288	54452468	+	2528	2.305	1

Wdr75	ENSMUSG00000025995	chr1	45852011	45880464	+	4717	2.304		1
Large	ENSMUSG00000004383	chr8	75338498	75877439	-	7883	2.303		1
Zfand6	ENSMUSG00000030629	chr7	91763564	91827861	-	1658	2.303		1
Lrpprc	ENSMUSG00000024120	chr17	85104587	85190129	-	8504	2.303		1
Cadm1	ENSMUSG00000032076	chr9	47338256	47665720	+	9734	2.302		1
Slc43a2	ENSMUSG00000038178	chr11	75345196	75391077	+	10042	2.302		1
Ola1	ENSMUSG00000027108	chr2	72930858	73056981	-	3561	2.301		1
Lcp2	ENSMUSG00000002699	chr11	33946920	33992295	+	4459	2.3		1
Ppp4r2	ENSMUSG00000052144	chr6	100783632	100818711	+	3641	2.299		1
Vwa5a	ENSMUSG00000023186	chr9	38525853	38550922	+	4899	2.298		1
Ehbp1l1	ENSMUSG00000024937	chr19	5707376	5726317	-	6440	2.298		1
Eif2c3	ENSMUSG00000028842	chr4	126008948	126106800	-	16571	2.298		1
Vps4b	ENSMUSG00000009907	chr1	108660648	108693305	-	8384	2.297		1
Cdkl3	ENSMUSG00000020389	chr11	51817723	51903286	+	5360	2.297		1
Nfxl1	ENSMUSG00000072889	chr5	72904540	72950923	-	4919	2.295		1
Pik3ca	ENSMUSG00000027665	chr3	32296593	32367408	+	9068	2.294		1
Zmpste24	ENSMUSG00000043207	chr4	120731842	120770846	-	7145	2.291		1
Fgl2	ENSMUSG00000039899	chr5	20878460	20884192	+	3788	2.291		1
Rnf214	ENSMUSG00000042790	chr9	45670496	45714977	-	6381	2.291		1
Snx25	ENSMUSG00000038291	chr8	47118615	47237513	-	4440	2.29		1
Maff	ENSMUSG00000042622	chr15	79177971	79189502	+	2119	2.289		1
Chd9	ENSMUSG00000056608	chr8	93352734	93578407	+	13054	2.289		1
Cbx5	ENSMUSG00000009575	chr15	103021975	103070247	-	9006	2.289		1
Calu	ENSMUSG00000029767	chr6	29298069	29327110	+	4733	2.288		1
Asgr2	ENSMUSG00000040963	chr11	69906146	69919689	+	1446	2.288		1
Adora2b	ENSMUSG00000018500	chr11	62062486	62079955	+	1845	2.288		1
Naalad2	ENSMUSG00000043943	chr9	18126395	18207439	-	4749	2.288		1
Snx24	ENSMUSG00000024535	chr18	53405316	53550477	+	1880	2.288		1
Cbfa2t2	ENSMUSG00000038533	chr2	154262217	154365092	+	8650	2.287		1
Cul4b	ENSMUSG00000031095	chrX	35884800	35929373	-	5527	2.287		1
Sgtb	ENSMUSG00000042743	chr13	104899821	104931815	+	3072	2.287		1
Cdon	ENSMUSG00000038119	chr9	35228713	35315236	+	7714	2.286		1
Mzt1	ENSMUSG00000033186	chr14	99433763	99445355	-	2256	2.286		1
Dnalc1	ENSMUSG00000042523	chr12	85455278	85488448	+	6976	2.285		1
Pde4dip	ENSMUSG00000038170	chr3	97493747	97692630	-	16508	2.284		1
Zbtb37	ENSMUSG00000043467	chr1	162938925	162964980	-	4815	2.283		1
Slc22a23	ENSMUSG00000038267	chr13	34271027	34437051	-	6386	2.283		1
Micall1	ENSMUSG00000033039	chr15	78939413	78967328	+	6691	2.283		1
Smarcc1	ENSMUSG00000032481	chr9	110034541	110142682	+	6646	2.282		1
Pkn3	ENSMUSG00000026785	chr2	29933204	29946542	+	4255	2.281		1
Topors	ENSMUSG00000036822	chr4	40206634	40216883	-	3856	2.281		1

Dpp8	ENSMUSG00000032393	chr9	64880265	64930457	+	4968	2.28		1
Ss18	ENSMUSG00000037013	chr18	14782707	14841423	-	4523	2.28		1
Mob4	ENSMUSG00000025979	chr1	55188075	55211743	+	4010	2.28		1
Slain2	ENSMUSG00000036087	chr5	73305543	73370068	+	4974	2.279		1
Pkd2	ENSMUSG00000034462	chr5	104888469	104934838	+	10169	2.278		1
Arfgef1	ENSMUSG00000067851	chr1	10127652	10222751	-	10429	2.278		1
Tmem106b	ENSMUSG00000029571	chr6	13019759	13039269	+	6931	2.277		1
Cdk12	ENSMUSG00000003119	chr11	98064373	98139818	+	11730	2.277		1
Dido1	ENSMUSG00000038914	chr2	180392669	180444704	-	13307	2.277		1
Ppp1r18	ENSMUSG00000034595	chr17	36002538	36012541	+	4272	2.274		1
Cdv3	ENSMUSG00000032803	chr9	103255432	103268110	-	4552	2.274		1
Zc3h15	ENSMUSG00000027091	chr2	83484592	83504779	+	2789	2.273		1
Bod1l	ENSMUSG00000061755	chr5	42178781	42235407	-	10389	2.273		1
Dopey1	ENSMUSG00000034973	chr9	86360761	86449413	+	9374	2.27		1
Tbk1	ENSMUSG00000020115	chr10	120983511	121023850	-	3031	2.27		1
Mad2l1	ENSMUSG00000029910	chr6	66485384	66491027	+	2203	2.27		1
Atf2	ENSMUSG00000027104	chr2	73654566	73730696	-	8921	2.27		1
Pdgfra	ENSMUSG00000029231	chr5	75548316	75594229	+	6737	2.269		1
Prpf4b	ENSMUSG00000021413	chr13	34967363	34994746	+	4465	2.269		1
Hipk1	ENSMUSG00000008730	chr3	103543738	103595486	-	8591	2.269		1
Zfp639	ENSMUSG00000027667	chr3	32414217	32419607	+	1458	2.268		1
Trim35	ENSMUSG00000022043	chr14	66915868	66930261	+	4620	2.268		1
Iffo2	ENSMUSG00000041025	chr4	139086463	139176297	+	6214	2.267		1
BC030336	ENSMUSG00000046096	chr7	127821134	127878368	+	4842	2.266		1
Zfp329	ENSMUSG00000057894	chr7	13390326	13404207	-	6742	2.266		1
Pnpla8	ENSMUSG00000036257	chr12	45322357	45413330	+	3947	2.266		1
9130008F23Rik	ENSMUSG00000054951	chr17	41012431	41017507	-	2159	2.266		1
Fndc3a	ENSMUSG00000033487	chr14	72937753	73109810	-	9944	2.265		1
Ssr3	ENSMUSG00000027828	chr3	65183577	65196545	-	4172	2.263		1
Ahr	ENSMUSG00000019256	chr12	36182646	36219710	-	6173	2.263		1
Dhx15	ENSMUSG00000029169	chr5	52541448	52581758	-	3009	2.263		1
4933421E11Rik	ENSMUSG00000056260	chr3	106487905	106539495	+	4835	2.262		1
Cep135	ENSMUSG00000036403	chr5	77017723	77075491	+	7188	2.261		1
Gnl2	ENSMUSG00000028869	chr4	124693829	124732624	+	3683	2.261		1
Etfa	ENSMUSG00000032314	chr9	55302315	55360050	-	1835	2.261		1
Zfp664	ENSMUSG00000079215	chr5	125343061	125383063	+	5729	2.26		1
Glt8d2	ENSMUSG00000020251	chr10	82113178	82153395	-	5773	2.259		1
4632411B12Rik	ENSMUSG00000010453	chr1	36392575	36426026	-	4741	2.258		1
Ddx19b	ENSMUSG00000033658	chr8	113527088	113555651	-	6742	2.257		1
Copb1	ENSMUSG00000030754	chr7	121359073	121398225	-	3346	2.256		1

Rbm26	ENSMUSG00000022119	chr14	105505968	105576544	-	8095	2.256	1
Wdfy1	ENSMUSG00000073643	chr1	79698837	79772718	-	6308	2.256	1
A430033K04Rik	ENSMUSG00000056014	chr5	139064087	139090052	+	3190	2.255	1
Cetn3	ENSMUSG00000021537	chr13	81922291	81936142	+	999	2.255	1
Spnb2	ENSMUSG00000020315	chr11	29999395	30168175	-	17492	2.255	1
Gm2058	ENSMUSG00000074903	chr7	46843888	46847049	+	2944	2.255	1
Plagl2	ENSMUSG00000051413	chr2	153053495	153067162	-	5735	2.254	1
Prrg3	ENSMUSG00000033361	chrX	69207963	69218061	+	6087	2.254	1
Nfyb	ENSMUSG00000020248	chr10	82211446	82226889	-	5068	2.252	1
Usp33	ENSMUSG00000025437	chr3	152009442	152056576	+	5960	2.251	1
Uevld	ENSMUSG00000043262	chr7	54178909	54213897	-	7226	2.251	1
Clic4	ENSMUSG00000037242	chr4	134769884	134828729	-	6572	2.25	1
Klhl7	ENSMUSG00000028986	chr5	23606408	23667041	+	3419	2.25	1
Ppil4	ENSMUSG00000015757	chr10	7512689	7542933	+	6450	2.249	1
Znrf2	ENSMUSG00000058446	chr6	54766910	54843494	+	6176	2.248	1
BC048403	ENSMUSG00000053684	chr10	121176993	121189934	+	3136	2.248	1
St3gal6	ENSMUSG00000022747	chr16	58468238	58524356	-	6080	2.248	1
Erc1	ENSMUSG00000030172	chr6	119520822	119798168	-	9711	2.248	1
Zfp715	ENSMUSG00000012640	chr7	50551567	50568664	-	5119	2.248	1
Cspp1	ENSMUSG00000056763	chr1	10028299	10126849	+	9939	2.247	1
Sphk1	ENSMUSG00000061878	chr11	116392239	116397988	+	4774	2.247	1
Osbpl9	ENSMUSG00000028559	chr4	108733750	108874877	-	7006	2.246	1
Eif4g2	ENSMUSG00000005610	chr7	118211499	118226544	-	9931	2.246	1
Chmp5	ENSMUSG00000028419	chr4	40895440	40912336	+	1519	2.246	1
Sec24b	ENSMUSG00000001052	chr3	129685677	129764471	-	6020	2.244	1
G2e3	ENSMUSG00000035293	chr12	52449048	52477973	+	7886	2.244	1
Tmsb10	ENSMUSG00000079523	chr6	72907341	72908742	-	1276	2.243	1
Snai1	ENSMUSG00000042821	chr2	167363695	167368314	+	1621	2.242	1
Rb1cc1	ENSMUSG00000025907	chr1	6196278	6266729	+	14690	2.242	1
1700049G17Rik	ENSMUSG00000070709	chr7	28692411	28714479	-	7518	2.242	1
Naa20	ENSMUSG00000002728	chr2	145727835	145742161	+	3705	2.242	1
Nphp3	ENSMUSG00000032558	chr9	103904874	103946060	+	6498	2.241	1
Rsl1d1	ENSMUSG00000005846	chr16	11193063	11203424	-	2258	2.24	1
Dlg5	ENSMUSG00000021782	chr14	24953175	25065142	-	8509	2.239	1
Perp	ENSMUSG00000019851	chr10	18564877	18576879	+	1901	2.239	1
Xdh	ENSMUSG00000024066	chr17	74233248	74299522	-	4692	2.238	1
Ermp1	ENSMUSG00000046324	chr19	29682704	29722905	-	9338	2.238	1
Gm12940	ENSMUSG00000085334	chr4	126710975	126726642	+	2680	2.238	1
Npm1	ENSMUSG00000057113	chr11	33052287	33063206	-	2665	2.237	1
Zfp609	ENSMUSG00000040524	chr9	65539391	65675371	-	9075	2.235	1
Ints12	ENSMUSG00000028016	chr3	132754804	132773952	+	7257	2.234	1

Col14a1	ENSMUSG00000022371	chr15	55139305	55352358	+	7010	2.234	1
Gm5454	ENSMUSG00000047643	chr13	104146330	104147784	+	1455	2.234	1
Capn7	ENSMUSG00000021893	chr14	32149824	32185172	+	4733	2.234	1
Pdcd6ip	ENSMUSG00000032504	chr9	113560862	113617377	-	6903	2.233	1
Zfp275	ENSMUSG00000031365	chrX	70587959	70604419	+	6746	2.233	1
Cchcr1	ENSMUSG00000040312	chr17	35654045	35667960	+	5278	2.231	1
Pam	ENSMUSG00000026335	chr1	99691691	99992223	-	7998	2.231	1
Fbxw7	ENSMUSG00000028086	chr3	84619190	84783120	+	7046	2.229	1
Cstf2	ENSMUSG00000031256	chrX	130593726	130621358	+	9180	2.229	1
Panx1	ENSMUSG00000031934	chr9	14809605	14849922	-	2997	2.229	1
BC027231	ENSMUSG00000036208	chr16	44724414	44737397	+	5741	2.228	1
2610039C10Rik	ENSMUSG00000022978	chr16	90719557	90727649	-	2863	2.226	1
Skap2	ENSMUSG00000059182	chr6	51809165	51962548	-	1638	2.226	1
Ssh2	ENSMUSG00000037926	chr11	77029789	77273722	+	9531	2.226	1
Ino80d	ENSMUSG00000040865	chr1	63004992	63161241	-	14665	2.225	1
Ube2w	ENSMUSG00000025939	chr1	16559863	16609570	-	2797	2.223	1
Nr2f1	ENSMUSG00000069171	chr13	78328234	78339018	-	4752	2.221	1
Bnip3	ENSMUSG00000078566	chr7	146082519	146101202	-	2265	2.22	1
Bcar3	ENSMUSG00000028121	chr3	122122698	122233098	+	3298	2.22	1
Lifr	ENSMUSG00000054263	chr15	7079559	7147489	+	10330	2.219	1
Thap6	ENSMUSG00000060466	chr5	92392359	92401068	+	3000	2.219	1
Naa50	ENSMUSG00000022698	chr16	44139943	44163479	+	4653	2.218	1
Synj2	ENSMUSG00000023805	chr17	5941280	6044290	+	8342	2.217	1
Vta1	ENSMUSG00000019868	chr10	14374561	14425366	-	2263	2.217	1
Pcm1	ENSMUSG00000031592	chr8	42325113	42419440	+	8497	2.216	1
Gimap9	ENSMUSG00000051124	chr6	48626128	48629113	+	1642	2.215	1
Chst15	ENSMUSG00000030930	chr7	139427463	139508911	-	5388	2.215	1
Bcr	ENSMUSG00000009681	chr10	74523641	74647668	+	6537	2.215	1
Arf2	ENSMUSG00000062421	chr11	103828053	103846651	+	2998	2.214	1
Mitd1	ENSMUSG00000026088	chr1	37931646	37947256	-	2929	2.214	1
Rsf1	ENSMUSG00000035623	chr7	104728406	104841292	+	12635	2.214	1
Amigo2	ENSMUSG00000048218	chr15	97074505	97077718	-	2871	2.213	1
Tmem20	ENSMUSG00000044026	chr19	38470470	38480097	+	3471	2.213	1
Zfp407	ENSMUSG00000048410	chr18	84297419	84759117	-	10293	2.212	1
Dyrk2	ENSMUSG00000028630	chr10	118296405	118305959	-	2137	2.212	1
6330416G13Rik	ENSMUSG00000045917	chr4	63219815	63247391	+	4109	2.212	1
Atxn1l	ENSMUSG00000069895	chr8	112250351	112261639	-	7526	2.21	1
Snap23	ENSMUSG00000027287	chr2	120393407	120426991	+	4293	2.21	1

3110052M02Rik	ENSMUSG00000035868	chr17	21794549	21799507	+	1383	2.209	1
Mmgt1	ENSMUSG00000061273	chrX	53838689	53851246	-	4234	2.209	1
Slc7a6	ENSMUSG00000031904	chr8	108692775	108722604	+	3730	2.208	1
Ubr1	ENSMUSG00000027272	chr2	120686005	120796451	-	9889	2.208	1
Sos2	ENSMUSG00000034801	chr12	70684748	70782839	-	5615	2.207	1
Atg4c	ENSMUSG00000028550	chr4	98860625	98926478	+	3351	2.207	1
Mospd1	ENSMUSG00000023074	chrX	50697775	50723679	-	2085	2.206	1
Kdsr	ENSMUSG00000009905	chr1	108616987	108656319	-	5582	2.205	1
Gm6788	ENSMUSG00000090737	chr19	28837651	28837920	-	270	2.205	1
Mfap3l	ENSMUSG00000031647	chr8	63111624	63155526	+	6837	2.203	1
D14Abb1e	ENSMUSG00000040651	chr14	28242033	28296738	+	7607	2.202	1
Aif1l	ENSMUSG00000001864	chr2	31805780	31828962	+	3911	2.201	1
Prdm10	ENSMUSG00000042496	chr9	31088123	31186128	+	6075	2.201	1
Cp	ENSMUSG00000003617	chr3	19857054	19909145	+	9566	2.199	1
Magi3	ENSMUSG00000052539	chr3	103817182	104024297	-	6773	2.199	1
4931406P16Rik	ENSMUSG00000066571	chr7	35021726	35098570	-	7174	2.199	1
Aftph	ENSMUSG00000049659	chr11	20585087	20641592	-	6979	2.196	1
Hps3	ENSMUSG00000027615	chr3	19895945	19935315	-	9103	2.195	1
Cytip	ENSMUSG00000026832	chr2	57981548	58047943	-	6744	2.195	1
Eif2s3y	ENSMUSG00000069049	chrY	346986	365290	+	4166	2.195	1
Ncoa2	ENSMUSG00000005886	chr1	13129186	13364164	-	14344	2.194	1
Cwc27	ENSMUSG00000021715	chr13	105421220	105607222	-	2873	2.193	1
Hectd1	ENSMUSG00000035247	chr12	52844709	52930523	-	8988	2.193	1
Gtf2e1	ENSMUSG00000022828	chr16	37509876	37539875	-	6285	2.192	1
Ccdc6	ENSMUSG00000048701	chr10	69559869	69655948	+	6385	2.192	1
Denr	ENSMUSG00000023106	chr5	124357184	124378841	+	2844	2.191	1
Vill	ENSMUSG00000038775	chr9	118961896	118980643	+	4409	2.191	1
Tifab	ENSMUSG00000049625	chr13	56275064	56280246	-	3498	2.189	1
Tcf4	ENSMUSG00000053477	chr18	69503800	69847621	+	12250	2.188	1
Atp2c1	ENSMUSG00000032570	chr9	105305869	105429649	-	7580	2.188	1
Fam126b	ENSMUSG00000038174	chr1	58579650	58643167	-	9343	2.187	1
Ptk2b	ENSMUSG00000059456	chr14	66772094	66899889	-	7398	2.186	1
2210008F06Rik	ENSMUSG00000091461	chr11	77303356	77304911	+	1082	2.185	1
Igsf3	ENSMUSG00000042035	chr3	101181048	101266983	+	7330	2.185	1
Rfx7	ENSMUSG00000037674	chr9	72380047	72470744	+	8231	2.184	1
Rapgef5	ENSMUSG00000041992	chr12	118754952	118995451	+	3987	2.184	1
Mtr	ENSMUSG00000021311	chr13	12279086	12350267	-	4304	2.183	1
Lasp1	ENSMUSG00000038366	chr11	97660314	97700078	+	6495	2.183	1
Man1a2	ENSMUSG00000008763	chr3	100366130	100489426	-	8184	2.183	1

Rnf130	ENSMUSG00000020376	chr11	49838848	49918260	+	1558	2.181	1
Zfp280b	ENSMUSG00000049764	chr10	75495357	75505979	+	5139	2.181	1
Papd7	ENSMUSG00000034575	chr13	69636837	69672717	-	3972	2.18	1
Rnf146	ENSMUSG00000038876	chr10	29063984	29082248	-	4454	2.18	1
Rsrc2	ENSMUSG00000029422	chr5	124178439	124199421	-	5473	2.18	1
Cited2	ENSMUSG00000039910	chr10	17443034	17445479	+	1968	2.179	1
F	ENSMUSG00000027274	chr2	136699516	136717125	-	3227	2.179	1
Itsn1	ENSMUSG00000022957	chr16	91729526	91920842	+	22539	2.179	1
Tslp	ENSMUSG00000024379	chr18	32975037	32979453	+	1133	2.178	1
Uba2	ENSMUSG00000052997	chr7	34925708	34954618	-	4092	2.178	1
Cpsf2	ENSMUSG00000041781	chr12	103214184	103244203	+	5315	2.178	1
Aasdhppt	ENSMUSG00000025894	chr9	4294793	4309494	-	2870	2.178	1
Cd300ld	ENSMUSG00000034641	chr11	114843588	114851236	-	2645	2.176	1
Mat1a	ENSMUSG00000037798	chr14	41918670	41937701	+	3208	2.175	1
Scpep1	ENSMUSG00000000278	chr11	88785334	88816779	-	2097	2.174	1
Bhlhe41	ENSMUSG00000030256	chr6	145806763	145814078	-	6262	2.173	1
AI480653	ENSMUSG00000047434	chr16	30955713	31081518	-	3412	2.172	1
Ywhae	ENSMUSG00000020849	chr11	75546371	75579347	+	2568	2.172	1
Naa16	ENSMUSG00000022020	chr14	79734011	79790585	-	6145	2.171	1
Zfp292	ENSMUSG00000039967	chr4	34750362	34830209	-	9986	2.17	1
Aff1	ENSMUSG00000029313	chr5	104121393	104284341	+	9458	2.17	1
Mtmr2	ENSMUSG00000031918	chr9	13552854	13610925	+	4969	2.169	1
Zadh2	ENSMUSG00000049090	chr18	84257550	84266906	+	3321	2.168	1
2200002D01Rik	ENSMUSG00000030587	chr7	30031580	30033451	-	1530	2.168	1
Fhl3	ENSMUSG00000032643	chr4	124377945	124385855	+	1683	2.167	1
Atp6v1a	ENSMUSG00000052459	chr16	44085515	44139818	-	6044	2.167	1
Spc25	ENSMUSG00000005233	chr2	69031952	69044251	-	3188	2.167	1
Lrp11	ENSMUSG00000019796	chr10	7309598	7345281	+	3418	2.166	1
Mettl7b	ENSMUSG00000025347	chr10	128395333	128398044	-	1227	2.165	1
Ldb2	ENSMUSG00000039706	chr5	44863372	45190946	-	2628	2.165	1
Dclre1a	ENSMUSG00000025077	chr19	56603649	56622516	-	4093	2.164	1
Ptpn2	ENSMUSG00000024539	chr18	67825165	67884249	-	3125	2.164	1
AI662270	ENSMUSG00000087107	chr11	83037078	83040106	+	1489	2.163	1
Vps13a	ENSMUSG00000046230	chr19	16691253	16855417	-	9866	2.163	1
Mfsd7b	ENSMUSG00000066595	chr1	192829711	192850069	-	4087	2.162	1
Rest	ENSMUSG00000029249	chr5	77694516	77715457	+	7254	2.161	1
Bmpr1a	ENSMUSG00000021796	chr14	35223920	35316527	-	6351	2.161	1
Recql	ENSMUSG00000030243	chr6	142298862	142335607	-	7510	2.16	1
Rbfox2	ENSMUSG00000033565	chr15	76909420	77137483	-	7412	2.16	1
Kpna3	ENSMUSG00000021929	chr14	61984023	62058784	-	4175	2.16	1
Gfpt2	ENSMUSG00000020363	chr11	49607680	49652115	+	5591	2.16	1

Gatad2b	ENSMUSG00000042390	chr3	90145576	90162042	+	1906	2.159		1
Azin1	ENSMUSG00000037458	chr15	38417185	38449021	-	6231	2.159		1
Zfp946	ENSMUSG00000071266	chr17	22561189	22593656	+	3504	2.158		1
Rab31	ENSMUSG00000056515	chr17	66001066	66122092	-	3813	2.158		1
Qrich1	ENSMUSG00000006673	chr9	108419418	108462498	+	3362	2.158		1
Stk4	ENSMUSG00000018209	chr2	163896058	163981260	+	5860	2.157		1
Pyroxd1	ENSMUSG00000041671	chr6	142294174	142311777	+	4933	2.157		1
Csf1	ENSMUSG00000014599	chr3	107543966	107563387	-	5262	2.156		1
Fcho1	ENSMUSG00000070000	chr8	74232286	74249615	-	4520	2.155		1
Slc35a5	ENSMUSG00000022664	chr16	45139686	45158819	-	4616	2.155		1
Rad51l1	ENSMUSG00000059060	chr12	80398338	80904257	+	2068	2.154		1
Zfp160	ENSMUSG00000067942	chr17	21145905	21165816	+	3791	2.153		1
Stx12	ENSMUSG00000028879	chr4	132409416	132440424	-	3188	2.152		1
Btk	ENSMUSG00000031264	chrX	131076875	131118109	-	3149	2.151		1
Rnf170	ENSMUSG00000013878	chr8	27229840	27254343	+	5032	2.15		1
Fam176a	ENSMUSG00000035104	chr6	81991037	82043093	+	2805	2.149		1
Rhoj	ENSMUSG00000046768	chr12	76409309	76502443	+	6484	2.148		1
Atf1	ENSMUSG00000023027	chr15	100058250	100091675	+	3060	2.148		1
Ubtd2	ENSMUSG00000044949	chr11	32355370	32416687	+	1012	2.147		1
Clk1	ENSMUSG00000026034	chr1	58467033	58480910	-	8139	2.147		1
Mterfd1	ENSMUSG00000021519	chr13	67007904	67034024	-	4652	2.146		1
Kctd10	ENSMUSG00000001098	chr5	114813576	114830524	-	4046	2.145		1
Cfl2	ENSMUSG00000062929	chr12	55959804	55963864	-	2940	2.145		1
Sgms2	ENSMUSG00000050931	chr3	131021903	131047858	-	5873	2.144		1
Cd68	ENSMUSG00000018774	chr11	69477715	69479655	-	1257	2.142		1
Cdk13	ENSMUSG00000041297	chr13	17807796	17896931	-	7081	2.142		1
Pdgfb	ENSMUSG00000000489	chr15	79826330	79845238	-	2918	2.142		1
Zfp949	ENSMUSG00000032425	chr9	88442858	88465924	+	4135	2.142		1
Nudcd2	ENSMUSG00000020328	chr11	40547169	40553548	+	2054	2.141		1
Ncam1	ENSMUSG00000039542	chr9	49310257	49607027	-	10121	2.141		1
Rcn2	ENSMUSG00000032320	chr9	55889652	55909689	+	4745	2.14		1
Spats2	ENSMUSG00000051934	chr15	98957009	99043637	+	3065	2.14		1
2700089E24Rik	ENSMUSG00000072704	chr6	133055257	133057765	+	2509	2.14		1
Ulk2	ENSMUSG00000004798	chr11	61589151	61668575	-	5977	2.139		1
Snx13	ENSMUSG00000020590	chr12	35731861	35832141	+	7435	2.138		1
Gnl3	ENSMUSG00000042354	chr14	31825620	31832275	-	1883	2.138		1
Pdgfd	ENSMUSG00000032006	chr9	6168612	6378843	+	3119	2.137		1
Zbtb44	ENSMUSG00000047412	chr9	30838229	30883464	+	9071	2.136		1
Hoxb8	ENSMUSG00000056648	chr11	96143219	96146639	+	2645	2.135		1
Atp11b	ENSMUSG00000037400	chr3	35653056	35755196	+	6616	2.133		1

Pip4k2b	ENSMUSG00000018547	chr11	97576471	97606018	-	5204	2.133	1
Hnrnpa3	ENSMUSG00000059005	chr2	75497318	75507464	+	5396	2.133	1
4933427 D14Rik	ENSMUSG00000020807	chr11	71967431	72020961	-	5697	2.133	1
Wasl	ENSMUSG00000029684	chr6	24563805	24615009	-	4923	2.132	1
Qtrtd1	ENSMUSG00000022704	chr16	43861520	43926922	-	4629	2.132	1
Trim16	ENSMUSG00000047821	chr11	62633733	62690964	+	6954	2.132	1
Socs2	ENSMUSG00000020027	chr10	94847996	94879814	-	9532	2.131	1
Ywhab	ENSMUSG00000018326	chr2	163820696	163844324	+	3382	2.13	1
Luc7l3	ENSMUSG00000020863	chr11	94149204	94183302	-	7278	2.129	1
Vps13d	ENSMUSG00000020220	chr4	144562529	144784898	-	24757	2.129	1
Akap10	ENSMUSG00000047804	chr11	61684809	61743759	-	6456	2.128	1
Glce	ENSMUSG00000032252	chr9	61905055	61918413	-	4626	2.127	1
Mpp5	ENSMUSG00000021112	chr12	79849934	79941698	+	5512	2.127	1
Prep	ENSMUSG00000019849	chr10	44787012	44878803	+	2729	2.126	1
Cpne3	ENSMUSG00000028228	chr4	19446401	19497255	-	7131	2.125	1
Cdr2	ENSMUSG00000030878	chr7	128100550	128125826	-	2782	2.124	1
Slc30a1	ENSMUSG00000037434	chr1	193730646	193737126	+	5244	2.123	1
Cirh1a	ENSMUSG00000041438	chr8	109417540	109446993	+	2248	2.123	1
2700081 O15Rik	ENSMUSG00000053080	chr19	7492115	7500394	+	5216	2.122	1
Rgs4	ENSMUSG00000038530	chr1	171671608	171677773	-	3098	2.119	1
Arid4a	ENSMUSG00000048118	chr12	72116977	72199579	+	5615	2.119	1
Ski	ENSMUSG00000029050	chr4	154528184	154596701	-	5562	2.119	1
Lancl3	ENSMUSG00000047344	chrX	8777028	8845211	+	3991	2.118	1
Cdc40	ENSMUSG00000038446	chr10	40551432	40602949	-	2323	2.116	1
Rnf145	ENSMUSG00000019189	chr11	44332466	44379022	+	7786	2.116	1
Brix1	ENSMUSG00000022247	chr15	10404534	10415702	-	2731	2.115	1
Fam160 b1	ENSMUSG00000033478	chr19	57435499	57464084	+	5630	2.115	1
E43002 5E21Rik	ENSMUSG00000022350	chr15	59163553	59205722	-	4090	2.115	1
Tmem1 84b	ENSMUSG00000009035	chr15	79191114	79233733	-	3361	2.113	1
1810029 B16Rik	ENSMUSG00000025591	chr8	68998229	69010429	-	4752	2.113	1
Dll4	ENSMUSG00000027314	chr2	119151520	119161698	+	3852	2.112	1
Fam48a	ENSMUSG00000027751	chr3	54497027	54532684	+	3763	2.112	1
Igdcc4	ENSMUSG00000032816	chr9	64949293	64985747	+	6366	2.112	1
Fam168 b	ENSMUSG00000037503	chr1	34870064	34899910	-	5219	2.11	1
Alg14	ENSMUSG00000039887	chr3	120994735	121064929	+	910	2.109	1

Jmy	ENSMUSG00000021690	chr13	94204165	94269644	-	4559	2.109	1
Cyld	ENSMUSG00000036712	chr8	91220927	91275844	+	8070	2.108	1
Hipk2	ENSMUSG00000061436	chr6	38644389	38826189	-	8882	2.108	1
Nol11	ENSMUSG00000018433	chr11	107027977	107050695	-	5357	2.106	1
Ubxn2b	ENSMUSG00000028243	chr4	6118245	6148835	+	6642	2.106	1
Plcl2	ENSMUSG00000038910	chr17	50648872	50827818	+	4050	2.105	1
Wac	ENSMUSG00000024283	chr18	7868830	7973545	+	5962	2.103	1
Fbxl3	ENSMUSG00000022124	chr14	103479456	103498783	-	5103	2.102	1
Slc38a9	ENSMUSG00000047789	chr13	113450984	113523106	+	3049	2.102	1
Steap3	ENSMUSG00000026389	chr1	122087334	122169282	-	5715	2.101	1
Nedd4l	ENSMUSG00000024589	chr18	65047410	65377480	+	9213	2.101	1
Fem1b	ENSMUSG00000032244	chr9	62639639	62659455	-	6488	2.101	1
Tmem168	ENSMUSG00000029569	chr6	13530687	13558100	-	5825	2.1	1
Thsd1	ENSMUSG00000031480	chr8	23337785	23371806	+	4422	2.1	1
Sult1a1	ENSMUSG00000030711	chr7	133816379	133819946	-	1507	2.099	1
Ptpra	ENSMUSG00000027303	chr2	130276014	130380036	+	3612	2.098	1
Vmp1	ENSMUSG00000018171	chr11	86397367	86497338	-	3862	2.098	1
Nr1d2	ENSMUSG00000021775	chr14	19036568	19071641	-	5009	2.098	1
C1d	ENSMUSG00000000581	chr11	17157582	17169179	+	3995	2.097	1
Il34	ENSMUSG00000031750	chr8	113265729	113329824	-	1959	2.097	1
Fundc1	ENSMUSG00000025040	chrX	17133690	17149451	-	2353	2.097	1
Gyg	ENSMUSG00000019528	chr3	20021970	20055216	-	2357	2.096	1
mt-Co1	ENSMUSG00000064351	chrM	5328	6872	+	1545	2.095	1
Khdrbs1	ENSMUSG00000028790	chr4	129380408	129419547	-	6419	2.093	1
Shoc2	ENSMUSG00000024976	chr19	54018796	54107758	+	4203	2.093	1
Cbll1	ENSMUSG00000020659	chr12	32170158	32184481	-	3543	2.093	1
Hiat1	ENSMUSG00000089911	chr3	116334082	116384178	-	4087	2.092	1
Cdc37l1	ENSMUSG00000024780	chr19	29064857	29099323	+	5238	2.091	1
Galnt11	ENSMUSG00000038072	chr5	24728700	24771736	+	2993	2.091	1
Hspa9	ENSMUSG00000024359	chr18	35097068	35114011	-	3932	2.091	1
Plekhf2	ENSMUSG00000049969	chr4	10915809	10935074	-	3039	2.09	1
Atm	ENSMUSG00000034218	chr9	53247254	53344845	-	10339	2.09	1
Gas5	ENSMUSG00000053332	chr1	162964553	162968670	+	3760	2.089	1
Cebpg	ENSMUSG00000056216	chr7	35831441	35841522	-	4935	2.089	1
Pcsk6	ENSMUSG00000030513	chr7	73006620	73195226	+	5144	2.089	1
Erich1	ENSMUSG00000051978	chr8	14027565	14090327	-	1579	2.089	1
Fcrlb	ENSMUSG00000070524	chr1	172837404	172843072	-	1284	2.089	1
Nat2	ENSMUSG00000051147	chr8	70018766	70026479	+	1509	2.089	1
Prpsap2	ENSMUSG00000020528	chr11	61543152	61575590	-	1962	2.087	1
Ddx59	ENSMUSG00000026404	chr1	138311848	138336735	+	2953	2.084	1
Scfd1	ENSMUSG00000020952	chr12	52478567	52551083	+	2180	2.084	1
Pilrb1	ENSMUSG00000066684	chr5	138293375	138299277	-	1375	2.084	1
Efnb1	ENSMUSG00000031217	chrX	96331469	96344330	+	3452	2.084	1

Dars	ENSMUSG00000026356	chr1	130260285	130313993	-	2507	2.083		1
Ywhah	ENSMUSG00000018965	chr5	33361465	33370615	+	3700	2.082		1
Evi5	ENSMUSG00000011831	chr5	108173814	108304126	-	8060	2.082		1
Prdm4	ENSMUSG00000035529	chr10	85354715	85379887	-	4514	2.081		1
Gm9581	ENSMUSG00000091562	chr16	59556678	59600712	-	4033	2.081		1
Sepp1	ENSMUSG00000064373	chr15	3218547	3230508	+	2845	2.08		1
Tmem132c	ENSMUSG00000034324	chr5	127722178	128046163	+	5850	2.08		1
Hnrpdl	ENSMUSG00000029328	chr5	100462596	100468683	-	4818	2.079		1
Eml5	ENSMUSG00000051166	chr12	100024814	100139694	-	10136	2.079		1
Jak1	ENSMUSG00000028530	chr4	100824972	100937887	-	7227	2.079		1
Wisp1	ENSMUSG00000005124	chr15	66722882	66754763	+	5518	2.079		1
Cd300a	ENSMUSG00000034652	chr11	114751355	114765968	+	4738	2.078		1
AI606181	ENSMUSG00000074873	chr19	41667853	41670648	+	2796	2.077		1
Cdk5rap2	ENSMUSG00000039298	chr4	69877890	70071477	-	13018	2.077		1
Lmbrd2	ENSMUSG00000039704	chr15	9070377	9128074	+	3863	2.076		1
Gpr116	ENSMUSG00000056492	chr17	43526415	43596506	+	8655	2.076		1
Ncapd2	ENSMUSG00000038252	chr6	125118027	125141613	-	4521	2.075		1
March6	ENSMUSG00000039100	chr15	31385654	31460792	-	6252	2.074		1
9230110C19Rik	ENSMUSG00000053070	chr9	8021672	8042823	-	1592	2.073		1
Kdm5b	ENSMUSG00000042207	chr1	136456748	136529454	+	7760	2.072		1
Bdkrb2	ENSMUSG00000021070	chr12	106801382	106831281	+	1680	2.072		1
Mtdh	ENSMUSG00000022255	chr15	34012449	34072141	+	12096	2.072		1
Rnaseh1	ENSMUSG00000020630	chr12	29334467	29344456	+	2469	2.072		1
Usp1	ENSMUSG00000028560	chr4	98590501	98602234	+	3733	2.071		1
Nab1	ENSMUSG00000002881	chr1	52514138	52557523	-	4083	2.071		1
Mrps31	ENSMUSG00000031533	chr8	23521812	23540137	+	1536	2.07		1
Fam83d	ENSMUSG00000027654	chr2	158593829	158612373	+	3991	2.07		1
Sh3bgrl2	ENSMUSG00000032261	chr9	83441934	83532408	+	6223	2.07		1
Ehd2	ENSMUSG00000074364	chr7	16532326	16552916	-	5100	2.07		1
Gpsm3	ENSMUSG00000034786	chr17	34726751	34728699	+	1343	2.069		1
Gtf2a1	ENSMUSG00000020962	chr12	92794356	92828927	-	5587	2.069		1
Suv39h1	ENSMUSG00000039231	chrX	7638297	7651886	-	5270	2.069		1
Fbxl16	ENSMUSG00000025738	chr17	25946030	25958189	+	3478	2.068		1
Spata5	ENSMUSG00000027722	chr3	37318818	37478018	+	3788	2.068		1
Ngef	ENSMUSG00000026259	chr1	89373409	89470445	-	3905	2.067		1
Zfp167	ENSMUSG00000063488	chr9	122797589	122805242	+	2228	2.066		1
Pla1a	ENSMUSG00000002847	chr16	38396205	38433225	-	1972	2.062		1
Lrrc58	ENSMUSG00000034158	chr16	37868475	37888944	+	8604	2.061		1
Ddx5	ENSMUSG00000020719	chr11	106641669	106650499	-	5775	2.06		1

Kif5b	ENSMUSG00000006740	chr18	6201000	6242172	-	7395	2.06	1
Hoxb7	ENSMUSG00000038721	chr11	96145571	96151650	+	4084	2.059	1
Rasa3	ENSMUSG00000031453	chr8	13566950	13677603	-	5093	2.059	1
Gpr125	ENSMUSG00000029090	chr5	50351190	50450235	-	4475	2.059	1
Nmd3	ENSMUSG00000027787	chr3	69525907	69552964	+	2803	2.058	1
Mis12	ENSMUSG00000040599	chr11	70833113	70840873	+	3003	2.058	1
Gtf2ird1	ENSMUSG00000023079	chr5	134833539	134932581	-	4073	2.056	1
Gnl3l	ENSMUSG00000025266	chrX	147417684	147451865	-	5570	2.054	1
Tet3	ENSMUSG00000034832	chr6	83312367	83391672	-	11086	2.054	1
Rhobtb1	ENSMUSG00000019944	chr10	68614182	68754539	+	6078	2.054	1
9130023 H24Rik	ENSMUSG00000062944	chr7	135377965	135381545	-	3581	2.053	1
Fkbp3	ENSMUSG00000020949	chr12	66163423	66174931	-	947	2.053	1
Oxsr1	ENSMUSG00000036737	chr9	119147550	119231545	-	5350	2.052	1
Tmem1 70b	ENSMUSG00000087370	chr13	41701585	41736716	+	7160	2.052	1
Trim25	ENSMUSG00000000275	chr11	88860690	88881607	+	5756	2.052	1
Hnrnpd	ENSMUSG00000000568	chr5	100387445	100407957	-	4863	2.05	1
Dip2c	ENSMUSG00000048264	chr13	9275774	9668174	+	8244	2.049	1
Pten	ENSMUSG00000013663	chr19	32831987	32900650	+	11507	2.049	1
Psma1	ENSMUSG00000030751	chr7	121408120	121419632	-	2103	2.048	1
Setd8	ENSMUSG00000049327	chr5	124889939	124912317	+	4035	2.048	1
Fam98b	ENSMUSG00000027349	chr2	117075475	117097276	+	3390	2.047	1
Smcr8	ENSMUSG00000049323	chr11	60591027	60601789	+	8077	2.047	1
Clasp1	ENSMUSG00000064302	chr1	120285635	120506009	+	7932	2.046	1
Plekha8	ENSMUSG00000005225	chr6	54545105	54595833	+	7005	2.045	1
Ywhaz	ENSMUSG00000022285	chr15	36700525	36724302	-	3999	2.045	1
Sbf2	ENSMUSG00000038371	chr7	117451527	117758436	-	12203	2.044	1
Rab6a	ENSMUSG00000030704	chr7	107755924	107789782	+	3728	2.044	1
Trib1	ENSMUSG00000032501	chr15	59479905	59488105	+	5699	2.043	1
Slc6a8	ENSMUSG00000019558	chrX	70918489	70927841	+	5210	2.043	1
Fam160 a1	ENSMUSG00000051000	chr3	85463983	85550188	-	5063	2.043	1
Fez2	ENSMUSG00000056121	chr17	78768552	78817471	-	4688	2.043	1
Zfp229	ENSMUSG00000061544	chr17	21870691	21885935	+	4525	2.042	1
Phf20l1	ENSMUSG00000072501	chr15	66409128	66473502	+	3373	2.042	1
Nipa2	ENSMUSG00000030452	chr7	63186657	63217846	-	5386	2.041	1
Fam199 x	ENSMUSG00000042595	chrX	133584133	133617042	+	8299	2.04	1
Dlc1	ENSMUSG00000031523	chr8	37630805	38015490	-	14274	2.04	1
Fam92a	ENSMUSG00000028218	chr4	12080556	12099162	-	4677	2.04	1
Cpne2	ENSMUSG00000034361	chr8	97056890	97094429	+	3198	2.039	1
Med12	ENSMUSG00000079487	chrX	98469369	98492804	+	7704	2.039	1
Bmp6	ENSMUSG00000039004	chr13	38436995	38592183	+	3586	2.039	1

Ptprk	ENSMUSG00000019889	chr10	27794626	28317203	+	7078	2.038	1
Zkscan3	ENSMUSG00000021327	chr13	21478874	21494624	-	7825	2.037	1
Fibin	ENSMUSG00000074971	chr2	110201082	110203150	-	2069	2.037	1
Rerg	ENSMUSG00000030222	chr6	137003346	137119018	-	2563	2.037	1
Tmem60	ENSMUSG00000045435	chr5	20388009	20392688	+	1147	2.037	1
Ipo9	ENSMUSG00000041879	chr1	137278889	137327076	-	8233	2.036	1
Gpatch8	ENSMUSG00000034621	chr11	102337229	102417706	-	8178	2.035	1
Appl2	ENSMUSG00000020263	chr10	83062778	83111483	-	5949	2.034	1
Scaf4	ENSMUSG00000022983	chr16	90229202	90284670	-	4379	2.034	1
Abr	ENSMUSG00000017631	chr11	76230236	76435816	-	9730	2.032	1
Mtx2	ENSMUSG00000027099	chr2	74663860	74716488	+	3675	2.032	1
Xpo7	ENSMUSG00000022100	chr14	71054053	71166435	-	15121	2.031	1
Hcls1	ENSMUSG00000022831	chr16	36935069	36963298	+	2525	2.03	1
Fam120a	ENSMUSG00000038014	chr13	48974588	49063386	-	5105	2.029	1
Lrrc57	ENSMUSG00000027286	chr2	120429974	120435256	-	2227	2.029	1
Tbc1d19	ENSMUSG00000039178	chr5	54200866	54295619	+	2688	2.027	1
Slmap	ENSMUSG00000021870	chr14	27232654	27354417	-	8054	2.026	1
Impa1	ENSMUSG00000027531	chr3	10313540	10331439	-	3455	2.025	1
Rybp	ENSMUSG00000072872	chr6	100178560	100237435	-	4458	2.025	1
Trbc2	ENSMUSG00000076498	chr6	41496729	41498180	+	521	2.024	1
Sparcl1	ENSMUSG00000029309	chr5	104508130	104543107	-	3114	2.023	1
Ireb2	ENSMUSG00000032293	chr9	54711562	54760341	+	5808	2.023	1
Ptgfrn	ENSMUSG00000027864	chr3	100844155	100914201	-	5902	2.022	1
Abcd4	ENSMUSG00000021240	chr12	85943481	85958416	-	2726	2.022	1
4933407C03Rik	ENSMUSG00000034390	chr8	119780919	119985403	+	4732	2.021	1
Tspan3	ENSMUSG00000032324	chr9	55983692	56008877	-	3232	2.021	1
Ltn1	ENSMUSG00000052299	chr16	87376896	87432851	-	7838	2.021	1
Traf6	ENSMUSG00000027164	chr2	101518586	101541826	+	7883	2.02	1
Aqr	ENSMUSG00000040383	chr2	113926906	114012760	-	11805	2.019	1
BC005537	ENSMUSG00000019132	chr13	24893526	24905882	+	3665	2.019	1
Larp4b	ENSMUSG00000033499	chr13	9093151	9172332	+	4684	2.018	1
Gm13157	ENSMUSG00000078495	chr4	147128083	147183895	-	2910	2.018	1
Zfp148	ENSMUSG00000022811	chr16	33380861	33503989	+	9545	2.015	1
Ccng1	ENSMUSG00000020326	chr11	40562054	40568813	-	3512	2.015	1
Cd9	ENSMUSG00000030342	chr6	125410284	125444809	-	2044	2.015	1
Pggt1b	ENSMUSG00000024477	chr18	46399603	46440504	-	2736	2.014	1
Slc37a2	ENSMUSG00000032122	chr9	37036734	37063323	-	5079	2.014	1
Blcap	ENSMUSG00000067787	chr2	157382098	157397010	-	3043	2.011	1

BC002059	ENSMUSG00000060149	chr17	17088503	17111119	+	1486	2.011		1
Ly96	ENSMUSG00000025779	chr1	16678537	16699686	+	603	2.009		1
Spg11	ENSMUSG00000033396	chr2	121879256	121944122	-	8042	2.008		1
Katnal1	ENSMUSG00000041298	chr5	149685908	149806799	-	11881	2.007		1
Atf7ip	ENSMUSG00000030213	chr6	136467372	136555898	+	4525	2.007		1
Ankrd16	ENSMUSG00000047909	chr2	11699503	11711952	+	5922	2.007		1
Nek7	ENSMUSG00000026393	chr1	140381291	140516273	-	3366	2.007		1
Cry1	ENSMUSG00000020038	chr10	84594447	84647799	-	3014	2.004		1
Enox1	ENSMUSG00000022012	chr14	77556623	78121214	+	3146	2.003		1
Fam3c	ENSMUSG00000029672	chr6	22256520	22306243	-	3713	2.003		1
Robo1	ENSMUSG00000022883	chr16	72663394	73046340	+	9547	2.003		1
Ubr3	ENSMUSG00000044308	chr2	69735303	69862070	+	9719	2.003		1
Cdyl	ENSMUSG00000059288	chr13	35751731	35965933	+	3950	2.002		1
Acp1	ENSMUSG00000044573	chr12	31578191	31596477	-	3221	2.001		1
Nvl	ENSMUSG00000026516	chr1	183023554	183074288	-	3060	2.001		1
Txndc9	ENSMUSG00000058407	chr1	38040712	38054191	-	5846	2.001		1
Pigm	ENSMUSG00000050229	chr1	174306662	174314230	+	7569	2		1
Tbck	ENSMUSG00000028030	chr3	132347108	132501470	+	4108	2		1
Sptlc2	ENSMUSG00000021036	chr12	88646008	88729305	-	8115	2		1
Hlx	ENSMUSG00000039377	chr1	186551019	186556498	-	2278	1.999		1
Papola	ENSMUSG00000021111	chr12	107018512	107077154	+	6603	1.999		1
Ift57	ENSMUSG00000032965	chr16	49699346	49765239	+	2862	1.999		1
Ap1ar	ENSMUSG00000074238	chr3	127510182	127540410	-	2391	1.997		1
Rcor2	ENSMUSG00000024968	chr19	7341815	7349715	+	3135	1.997		1
Vezt	ENSMUSG00000036099	chr10	93401910	93498562	-	20631	1.996		1
AA987161	ENSMUSG00000058093	chr13	67690380	67710644	-	4861	1.995		1
1200009I06Rik	ENSMUSG00000021280	chr12	112655228	112669393	+	4268	1.994		1
Ncapd3	ENSMUSG00000035024	chr9	26837760	26902896	+	5503	1.994		1
4921524J17Rik	ENSMUSG00000036934	chr8	87932658	87956740	-	1595	1.993		1
Adnp2	ENSMUSG00000053950	chr18	80323077	80348221	-	4985	1.993		1
Coro1c	ENSMUSG00000004530	chr5	114292445	114358767	-	9664	1.992		1
Prmt3	ENSMUSG00000030505	chr7	57033716	57113635	+	3361	1.992		1
Pcyt1a	ENSMUSG00000005615	chr16	32431007	32475156	+	5751	1.992		1
Mtap6	ENSMUSG00000055407	chr7	106415957	106485647	+	6958	1.991		1
Ttc3	ENSMUSG00000040785	chr16	94592225	94690829	+	15134	1.991		1
Snx18	ENSMUSG00000042364	chr13	114382388	114408772	-	4446	1.99		1
Plekha7	ENSMUSG00000045659	chr7	123267008	123451890	-	5539	1.99		1
Trp53bp2	ENSMUSG00000026510	chr1	184339303	184392563	+	4361	1.989		1
Cse1l	ENSMUSG00000002718	chr2	166731540	166771889	+	5273	1.988		1
Scn7a	ENSMUSG00000034810	chr2	66511482	66622971	-	7338	1.987		1
Cstf3	ENSMUSG00000027176	chr2	104430680	104505586	+	5879	1.986		1

Malt1	ENSMUSG00000032688	chr18	65590617	65638477	+	5106	1.986	1
Nsun2	ENSMUSG00000021595	chr13	69672624	69774658	+	5737	1.986	1
Sh2b3	ENSMUSG00000042594	chr5	122265497	122286868	-	4734	1.986	1
Naa15	ENSMUSG00000063273	chr3	51219938	51279907	+	6090	1.985	1
Arhgap22	ENSMUSG00000063506	chr14	34027212	34183122	+	3878	1.985	1
Wapal	ENSMUSG00000041408	chr14	35487114	35561169	+	6623	1.984	1
Mtap7d1	ENSMUSG00000028849	chr4	125909411	125933587	-	3652	1.984	1
Gtf3c3	ENSMUSG00000041303	chr1	54454425	54495815	-	3005	1.983	1
Bahcc1	ENSMUSG00000039741	chr11	120094261	120153610	+	11118	1.983	1
Ercc6	ENSMUSG00000054051	chr14	33326707	33394175	+	8422	1.982	1
Fam149a	ENSMUSG00000070044	chr8	46422071	46467645	-	5954	1.982	1
Ankrd13c	ENSMUSG00000039988	chr3	157610378	157670288	+	3149	1.981	1
Ublcp1	ENSMUSG00000041231	chr11	44268073	44284000	-	2542	1.981	1
Ryk	ENSMUSG00000032547	chr9	102737247	102810635	+	4411	1.978	1
1810033B17Rik	ENSMUSG00000013974	chr8	3665762	3668905	+	1132	1.978	1
Myst3	ENSMUSG00000031540	chr8	23970007	24053731	+	9180	1.977	1
Sh2b2	ENSMUSG00000005057	chr5	136694019	136720773	-	2848	1.976	1
BC024659	ENSMUSG00000091264	chr13	41345229	41371943	+	4464	1.976	1
Dynlt1a	ENSMUSG00000092074	chr17	6310547	6317474	-	742	1.976	1
Lin9	ENSMUSG00000058729	chr1	182571498	182620816	+	3205	1.976	1
Mapk1	ENSMUSG00000063358	chr16	16983475	17047546	+	7110	1.976	1
1110018G07Rik	ENSMUSG00000042350	chr12	86259098	86311850	-	6044	1.975	1
Spice1	ENSMUSG00000043065	chr16	44347234	44388610	+	5416	1.975	1
Ypel5	ENSMUSG00000039770	chr17	73186044	73200535	+	2869	1.975	1
Zfp87	ENSMUSG00000071285	chr13	67616722	67622261	-	2556	1.975	1
Gxylt1	ENSMUSG00000036197	chr15	93070173	93105610	-	6722	1.974	1
Sos1	ENSMUSG00000024241	chr17	80793092	80879793	-	8919	1.973	1
Gtf2h2	ENSMUSG00000021639	chr13	101230173	101262534	-	4372	1.973	1
Kdm4c	ENSMUSG00000028397	chr4	73888401	74051764	+	5823	1.973	1
Guf1	ENSMUSG00000029208	chr5	69948163	69967211	+	9114	1.971	1
Gm1818	ENSMUSG00000091277	chr12	49655169	49660958	-	2399	1.971	1
Cmklr1	ENSMUSG00000042190	chr5	114062363	114100435	-	3408	1.971	1
Tmeff1	ENSMUSG00000028347	chr4	48598046	48676003	+	2549	1.97	1
Impad1	ENSMUSG00000066324	chr4	4689631	4720502	-	6543	1.97	1
Arhgef12	ENSMUSG00000059495	chr9	42771925	42913801	-	10483	1.97	1
Vezf1	ENSMUSG00000018377	chr11	87881781	87898231	+	4601	1.969	1
Lbr	ENSMUSG00000004880	chr1	183745447	183772532	-	3551	1.968	1
Chd2	ENSMUSG00000078671	chr7	80571577	80686632	-	9531	1.968	1

Ccdc109a	ENSMUSG00000009647	chr10	58909732	59079440	-	6936	1.968	1
Cd2ap	ENSMUSG00000061665	chr17	42929900	43013373	-	5399	1.968	1
Cdc45	ENSMUSG00000000028	chr16	18780540	18812080	-	2252	1.967	1
Rap1gds1	ENSMUSG00000028149	chr3	138588870	138738163	-	3663	1.967	1
Esf1	ENSMUSG00000045624	chr2	139945619	139996300	-	4471	1.965	1
Cdr2l	ENSMUSG00000050910	chr11	115243230	115257446	+	3700	1.964	1
Pcnx	ENSMUSG00000021140	chr12	82961017	83101911	+	12169	1.962	1
B230120H23Rik	ENSMUSG00000004085	chr2	72123694	72280667	+	10175	1.962	1
Aebp1	ENSMUSG00000020473	chr11	5761950	5772091	+	4058	1.961	1
Dtx2	ENSMUSG00000004947	chr5	136470670	136508742	+	7032	1.961	1
Ppp2r5c	ENSMUSG00000017843	chr12	111685518	111821271	+	4588	1.961	1
Fzd5	ENSMUSG00000045005	chr1	64777134	64784324	-	6912	1.961	1
Bbx	ENSMUSG00000022641	chr16	50191957	50432503	-	14205	1.961	1
Nav2	ENSMUSG00000052512	chr7	56501559	56865457	+	15867	1.96	1
Rpap3	ENSMUSG00000022466	chr15	97505536	97536253	-	2260	1.96	1
Casp8ap2	ENSMUSG00000028282	chr4	32702426	32740240	+	6826	1.96	1
Cilp	ENSMUSG00000042254	chr9	65112987	65128413	+	4297	1.96	1
Taf15	ENSMUSG00000020680	chr11	83286588	83320245	+	2919	1.959	1
Fgfr1	ENSMUSG00000031565	chr8	26624126	26686186	+	8061	1.958	1
Cenpa	ENSMUSG00000029177	chr5	30969150	30977200	+	2503	1.958	1
Ch25h	ENSMUSG00000050370	chr19	34548276	34549625	-	1350	1.957	1
Gpr114	ENSMUSG00000061577	chr8	97447594	97467190	+	3147	1.957	1
Actn1	ENSMUSG00000015143	chr12	81268529	81361358	-	3805	1.956	1
Ik	ENSMUSG00000024474	chr18	36904310	36917293	+	1996	1.956	1
Tax1bp1	ENSMUSG00000004535	chr6	52663723	52716774	+	6609	1.956	1
4632415L05Rik	ENSMUSG00000048106	chr3	19794886	19795962	+	1077	1.956	1
Clk4	ENSMUSG00000020385	chr11	51075232	51095268	+	5797	1.955	1
Dicer1	ENSMUSG00000041415	chr12	105925952	105990162	-	10057	1.955	1
Ubp1	ENSMUSG00000009741	chr9	113840052	113886317	+	3850	1.955	1
Hsp90aa1	ENSMUSG00000021270	chr12	111928815	111940938	-	3931	1.955	1
Mprip	ENSMUSG00000005417	chr11	59474807	59594362	+	13370	1.954	1
Cenpl	ENSMUSG00000026708	chr1	163000898	163016855	+	5040	1.952	1
Notch3	ENSMUSG00000038146	chr17	32257765	32303825	-	8044	1.951	1
Slc2a10	ENSMUSG00000027661	chr2	165329287	165345417	+	3570	1.951	1
Gtf2b	ENSMUSG00000028271	chr3	142428190	142446567	+	1271	1.951	1
Pdcl3	ENSMUSG00000026078	chr1	39044659	39054081	+	1706	1.951	1
Dusp16	ENSMUSG00000030203	chr6	134665488	134742643	-	7653	1.95	1
Gdap10	ENSMUSG00000059937	chr12	33506737	33511769	+	5003	1.95	1

Cyr61	ENSMUSG00000028195	chr3	145309935	145312949	-	2028	1.949	1
Psip1	ENSMUSG00000028484	chr4	83101584	83132363	-	5082	1.949	1
Drosha	ENSMUSG00000022191	chr15	12754570	12865045	+	5934	1.948	1
Ftsj3	ENSMUSG00000020706	chr11	106110456	106117393	-	4358	1.948	1
Itpripl1	ENSMUSG00000074825	chr2	126964508	126969178	-	3738	1.947	1
N4bp2l2	ENSMUSG00000029655	chr5	151410582	151468187	-	12262	1.947	1
Slc2a1	ENSMUSG00000028645	chr4	118781316	118809935	+	2853	1.947	1
Vapa	ENSMUSG00000024091	chr17	65929396	65962895	-	1640	1.946	1
Zbtb2	ENSMUSG00000075327	chr10	5958433	5979467	+	3338	1.944	1
Sbno1	ENSMUSG00000038095	chr5	124818711	124875923	-	10197	1.943	1
Zc3hav1	ENSMUSG00000029826	chr6	38255286	38304603	-	6518	1.942	1
Sc5d	ENSMUSG00000032018	chr9	42062260	42072383	-	2328	1.942	1
Nsf	ENSMUSG00000034187	chr11	103683096	103815370	-	8346	1.941	1
Rnf19a	ENSMUSG00000022280	chr15	36169689	36212902	-	4276	1.941	1
Cpeb1	ENSMUSG00000025586	chr7	88491912	88600351	-	4192	1.939	1
Ssb	ENSMUSG00000068882	chr2	69699619	69709903	+	2164	1.939	1
A630007B06Rik	ENSMUSG00000035173	chr19	56861971	56896680	-	6899	1.939	1
Ttc13	ENSMUSG00000037300	chr8	127195235	127245883	-	10190	1.939	1
C1s	ENSMUSG00000038521	chr6	124480363	124586998	-	3490	1.937	1
Mtcp1	ENSMUSG00000031200	chrX	72655783	72661927	-	4478	1.936	1
Unc45b	ENSMUSG00000018845	chr11	82724052	82756905	+	4493	1.934	1
Zfp445	ENSMUSG00000047036	chr9	122761032	122771246	-	2961	1.932	1
Vps13b	ENSMUSG00000037646	chr15	35301301	35860983	+	13482	1.932	1
Ercc5	ENSMUSG00000026048	chr1	44204589	44238105	+	4495	1.931	1
Gfm1	ENSMUSG00000027774	chr3	67234018	67280451	+	4419	1.93	1
BC016423	ENSMUSG00000033799	chr13	3565282	3610354	-	8341	1.93	1
Fzd4	ENSMUSG00000049791	chr7	96552876	96558620	+	3685	1.929	1
Mfap5	ENSMUSG00000030116	chr6	122455863	122479308	+	3589	1.929	1
Klc1	ENSMUSG00000021288	chr12	112997060	113046055	+	4072	1.929	1
Wnt7a	ENSMUSG00000030093	chr6	91313975	91361357	-	3172	1.928	1
6530402F18Rik	ENSMUSG00000079499	chr2	29100627	29108526	-	5511	1.927	1
Arid2	ENSMUSG00000033237	chr15	96117949	96235894	+	11208	1.926	1
Taf1d	ENSMUSG00000031939	chr9	15110658	15121441	+	1698	1.925	1
Etaa1	ENSMUSG00000016984	chr11	17838759	17853878	-	4185	1.925	1
Myo18a	ENSMUSG00000000631	chr11	77576748	77679482	+	14849	1.923	1
Dnajb6	ENSMUSG00000029131	chr5	30062228	30113018	+	5721	1.923	1
Arid4b	ENSMUSG00000039219	chr13	14155499	14291870	+	13705	1.923	1
Zmynd17	ENSMUSG00000021815	chr14	21302086	21316123	-	1829	1.923	1
Pcdhga9	ENSMUSG00000023036	chr18	37833989	38001527	+	28980	1.923	1
Poc1b	ENSMUSG00000019952	chr10	98569670	98660708	+	3748	1.922	1

Pask	ENSMUSG00000026274	chr1	95205347	95240059	-	9060	1.922	1
Sntb2	ENSMUSG00000041308	chr8	109459650	109538092	+	4423	1.922	1
Fam20a	ENSMUSG00000020614	chr11	109531063	109583593	-	3634	1.922	1
Smc6	ENSMUSG00000020608	chr12	11272692	11326591	+	5603	1.922	1
Epm2aip1	ENSMUSG00000046785	chr9	111174349	111181593	+	7245	1.921	1
Ppp2r1b	ENSMUSG00000032058	chr9	50653406	50702334	+	6449	1.921	1
Atxn2	ENSMUSG00000042605	chr5	122161346	122266502	+	7161	1.92	1
Cyb5r3	ENSMUSG00000018042	chr15	82983924	83003022	-	4298	1.919	1
Jdp2	ENSMUSG00000034271	chr12	86940366	86980827	+	1757	1.918	1
Cct4	ENSMUSG00000007739	chr11	22890519	22903780	+	4201	1.918	1
Paip1	ENSMUSG00000025451	chr13	120217408	120247025	+	3456	1.916	1
Fcgr1	ENSMUSG00000015947	chr3	96086832	96098179	-	2615	1.916	1
Hoxd10	ENSMUSG00000050368	chr2	74529147	74533163	+	2651	1.916	1
Zcchc9	ENSMUSG00000021621	chr13	91936138	91947301	-	1532	1.915	1
Slc12a4	ENSMUSG00000017765	chr8	108467490	108489974	-	4250	1.915	1
Taf4b	ENSMUSG00000054321	chr18	14941754	15058868	+	5158	1.914	1
Rpl24	ENSMUSG00000022601	chr16	55966388	56009026	+	6029	1.914	1
Wasf2	ENSMUSG00000028868	chr4	132686420	132755671	+	6287	1.914	1
Itpr3	ENSMUSG00000042644	chr17	27194249	27259168	+	8990	1.913	1
Dimt1	ENSMUSG00000021692	chr13	107737209	107750304	+	2545	1.913	1
Nkap	ENSMUSG00000016409	chrX	34666790	34690741	+	4326	1.913	1
Spire2	ENSMUSG00000010154	chr8	125856613	125893415	+	2330	1.913	1
Pogz	ENSMUSG00000038902	chr3	94641489	94686248	+	9218	1.912	1
Gnpda1	ENSMUSG00000052102	chr18	38487212	38498657	-	2392	1.912	1
Phactr2	ENSMUSG00000062866	chr10	12933201	13194202	-	6010	1.911	1
Cep76	ENSMUSG00000073542	chr18	67777051	67800990	-	3979	1.911	1
Ankra2	ENSMUSG00000021661	chr13	99033113	99044705	+	3510	1.911	1
Sdc1	ENSMUSG00000020592	chr12	8778129	8800521	+	3809	1.91	1
Dip2b	ENSMUSG00000023026	chr15	99869095	100049904	+	9553	1.909	1
5730559C18Rik	ENSMUSG00000041605	chr1	138110108	138130841	-	3105	1.908	1
Mapk11	ENSMUSG00000053137	chr15	88972916	88980036	-	2440	1.908	1
Fam71f2	ENSMUSG00000079652	chr6	29229593	29240680	+	1276	1.907	1
Igkv1-110	ENSMUSG00000076520	chr6	68220521	68221261	+	359	1.907	1
Fig4	ENSMUSG00000038417	chr10	40907978	41023047	-	3263	1.907	1
Nfkb1	ENSMUSG00000028163	chr3	135247619	135354511	-	4794	1.907	1
Mios	ENSMUSG00000042447	chr6	8159222	8186274	+	6552	1.907	1
Xpo4	ENSMUSG00000021952	chr14	58199839	58284267	-	7266	1.907	1
Stk24	ENSMUSG00000063410	chr14	121685565	121778556	-	2676	1.906	1
Ggps1	ENSMUSG00000021302	chr13	14144637	14155668	-	2391	1.905	1
Faim	ENSMUSG00000032463	chr9	98886792	98902437	+	1073	1.905	1

Prpf40a	ENSMUSG00000061136	chr2	52993738	53050318	-	10273	1.904	1
Ddx6	ENSMUSG00000032097	chr9	44412975	44448814	+	6065	1.903	1
Tial1	ENSMUSG00000030846	chr7	135583291	135605231	-	5861	1.902	1
Ccdc55	ENSMUSG00000037958	chr11	76857794	76891937	-	3628	1.902	1
Mrpl1	ENSMUSG00000029486	chr5	96638512	96695746	+	4295	1.902	1
D8Ertd82e	ENSMUSG00000050271	chr8	37157882	37210841	+	9606	1.901	1
Utp15	ENSMUSG00000041747	chr13	99016800	99032947	-	4071	1.9	1
Rnf14	ENSMUSG00000060450	chr18	38456289	38477501	+	3515	1.899	1
Ncoa6	ENSMUSG00000038369	chr2	155216401	155299630	-	9735	1.898	1
Agt	ENSMUSG00000031980	chr8	127080434	127093606	-	1891	1.897	1
Dennd2a	ENSMUSG00000038456	chr6	39412377	39507866	-	5429	1.895	1
Sec24a	ENSMUSG00000036391	chr11	51505765	51577136	-	10703	1.895	1
Tspan8	ENSMUSG00000034127	chr10	115254340	115286949	+	1522	1.894	1
Zfp518a	ENSMUSG00000049164	chr19	40985983	40992437	+	6455	1.894	1
Zcchc10	ENSMUSG00000018239	chr11	53138181	53146803	+	1391	1.894	1
Srsf1	ENSMUSG00000018379	chr11	87860875	87867257	+	5710	1.893	1
Dennd1b	ENSMUSG00000056268	chr1	140860013	141072620	+	13293	1.893	1
2610507B11Rik	ENSMUSG00000010277	chr11	78075254	78104125	+	8798	1.893	1
Rngtt	ENSMUSG00000028274	chr4	33397286	33589589	+	7094	1.892	1
Apool	ENSMUSG00000025525	chrX	109424943	109486364	+	3522	1.892	1
She	ENSMUSG00000046280	chr3	89635292	89662756	+	5733	1.891	1
Plxna1	ENSMUSG00000030084	chr6	89266310	89312607	-	9025	1.889	1
Zfp709	ENSMUSG00000056019	chr8	74411163	74414811	+	2181	1.889	1
Mthfs	ENSMUSG00000066442	chr9	89094217	89135064	+	3067	1.889	1
Zc3h8	ENSMUSG00000027387	chr2	128752004	128769813	-	2459	1.889	1
Ccl24	ENSMUSG00000004814	chr5	136045807	136048913	-	1289	1.889	1
Dag1	ENSMUSG00000039952	chr9	108108286	108166255	-	4563	1.887	1
Mcart1	ENSMUSG00000045973	chr4	45408795	45421633	-	5505	1.886	1
Taf9	ENSMUSG00000052293	chr13	101421298	101426014	+	1333	1.885	1
Ino80	ENSMUSG00000034154	chr2	119198778	119303423	-	6806	1.885	1
Enpep	ENSMUSG00000028024	chr3	128972093	129035638	-	4706	1.884	1
4930402H24Rik	ENSMUSG00000027309	chr2	130531936	130732142	-	10659	1.883	1
Brms1l	ENSMUSG00000012076	chr12	56937353	56970722	+	2566	1.883	1
3110001I22Rik	ENSMUSG00000079737	chr16	13672022	13678498	+	1722	1.883	1
Hnrnpa2b1	ENSMUSG00000004980	chr6	51410433	51419893	-	4125	1.882	1
BC018507	ENSMUSG00000034525	chr13	70727567	70776512	-	7675	1.882	1
Klraq1	ENSMUSG00000034709	chr17	88929464	88987705	+	3133	1.882	1
Ccr7	ENSMUSG00000037944	chr11	99005510	99016391	-	1978	1.882	1

Glud1	ENSMUSG00000021794	chr14	35123913	35158330	+	4089	1.88		1
Tdrd3	ENSMUSG00000022019	chr14	87816390	87945315	+	5834	1.879		1
Ktn1	ENSMUSG00000021843	chr14	48283431	48356238	+	4484	1.878		1
Mtmr12	ENSMUSG00000039458	chr15	12134783	12201995	+	4785	1.878		1
1110018 J18Rik	ENSMUSG00000021482	chr13	64391124	64414018	-	2992	1.877		1
Ell2	ENSMUSG00000001542	chr13	75844932	75909804	+	3647	1.876		1
Plekhg2	ENSMUSG00000037552	chr7	29144623	29157681	-	6860	1.876		1
Tmed7	ENSMUSG00000033184	chr18	46719889	46757189	-	8839	1.875		1
Tada1	ENSMUSG00000026563	chr1	168309228	168323752	+	4110	1.874		1
2700050 L05Rik	ENSMUSG00000039990	chr7	140829226	140864654	+	5992	1.874		1
Ankle2	ENSMUSG00000029501	chr5	110660060	110685666	+	5218	1.871		1
Acly	ENSMUSG00000020917	chr11	100337667	100389314	-	5280	1.87		1
Rgmb	ENSMUSG00000048027	chr17	15941626	15963550	-	4224	1.87		1
Fam175 a	ENSMUSG00000035234	chr5	101234211	101249984	-	3011	1.869		1
Psme4	ENSMUSG00000040850	chr11	30671726	30780361	+	8382	1.869		1
Trim39	ENSMUSG00000045409	chr17	36395818	36409192	-	7223	1.869		1
Tln2	ENSMUSG00000052698	chr9	67064894	67407510	-	12511	1.868		1
Atp2a2	ENSMUSG00000029467	chr5	122903531	122952183	-	5674	1.868		1
Zwint	ENSMUSG00000019923	chr10	72117593	72137712	+	4434	1.868		1
Ccny	ENSMUSG00000024286	chr18	9314042	9450148	-	4017	1.868		1
Chaf1a	ENSMUSG00000002835	chr17	56179827	56206973	+	2844	1.867		1
Wdr67	ENSMUSG00000022364	chr15	57743754	57801623	+	6367	1.867		1
Unc5b	ENSMUSG00000020099	chr10	60225358	60294329	-	5852	1.866		1
Vps54	ENSMUSG00000020128	chr11	21138892	21221139	+	7907	1.866		1
Lima1	ENSMUSG00000023022	chr15	99608901	99705859	-	7166	1.865		1
Zfp568	ENSMUSG00000074221	chr7	30768974	30813301	+	7183	1.864		1
Tmem1 65	ENSMUSG00000029234	chr5	76612905	76638270	+	2588	1.864		1
Cnot1	ENSMUSG00000036550	chr8	98243351	98331366	-	8394	1.863		1
Stam2	ENSMUSG00000055371	chr2	52547183	52601315	-	6007	1.863		1
Kif21b	ENSMUSG00000041642	chr1	138027966	138074575	+	10813	1.863		1
Trib2	ENSMUSG00000020601	chr12	15798533	15823683	-	4290	1.863		1
Dot1l	ENSMUSG00000061589	chr10	80217951	80258206	+	8714	1.862		1
Pde7b	ENSMUSG00000019990	chr10	20117810	20444884	-	7232	1.862		1
Scaf8	ENSMUSG00000046201	chr17	3114972	3198855	+	5866	1.862		1
Rad50	ENSMUSG00000020380	chr11	53463021	53520821	-	5414	1.862		1
Alox5ap	ENSMUSG00000060063	chr5	150076634	150099621	+	883	1.862		1
Mkl1	ENSMUSG00000042292	chr15	80842711	81021187	-	6124	1.862		1
Arhgef1 1	ENSMUSG00000041977	chr3	87421481	87541956	+	7918	1.861		1
Nhlrc3	ENSMUSG00000042997	chr3	53252510	53267245	-	5601	1.861		1
Sgk3	ENSMUSG00000025915	chr1	9788211	9892637	+	5760	1.861		1

Slc2a9	ENSMUSG00000005107	chr5	38740512	38894382	-	5083	1.861		1
Lass6	ENSMUSG00000027035	chr2	68699498	68952339	+	7956	1.861		1
Arl11	ENSMUSG00000043157	chr14	61928590	61930773	+	1482	1.86		1
Wdfy3	ENSMUSG00000043940	chr5	102261972	102498940	-	15354	1.86		1
Rev3l	ENSMUSG00000019841	chr10	39451924	39595017	+	13293	1.86		1
Ebf1	ENSMUSG00000057098	chr11	44430819	44821593	+	9756	1.859		1
Trmt5	ENSMUSG00000034442	chr12	74380999	74387698	-	2069	1.858		1
Cul2	ENSMUSG00000024231	chr18	3382986	3436698	+	4461	1.858		1
Tlr3	ENSMUSG00000031639	chr8	46481019	46495962	-	4396	1.857		1
B3gnt9-ps	ENSMUSG00000069920	chr8	107776538	107779053	-	2516	1.857		1
Rreb1	ENSMUSG00000039087	chr13	37870269	38043871	+	13991	1.856		1
Cops3	ENSMUSG00000019373	chr11	59631297	59653340	-	3454	1.856		1
Pex13	ENSMUSG00000020283	chr11	23546479	23565959	-	4644	1.855		1
Lrrc17	ENSMUSG00000039883	chr5	21049345	21081718	+	2186	1.855		1
Csf3r	ENSMUSG00000028859	chr4	125701794	125722219	+	4144	1.855		1
Nop14	ENSMUSG00000036693	chr5	34981181	35002797	-	2734	1.855		1
Ppp1r15b	ENSMUSG00000046062	chr1	135027720	135036377	+	5611	1.853		1
Cand1	ENSMUSG00000020114	chr10	118636311	118677111	-	8357	1.851		1
Ubr2	ENSMUSG00000023977	chr17	47065241	47147481	-	7747	1.85		1
Fam103a1	ENSMUSG00000038646	chr7	88907811	88914377	+	1741	1.85		1
Wnt11	ENSMUSG00000015957	chr7	105983622	106003705	+	3633	1.849		1
Zfp566	ENSMUSG00000078768	chr7	30862356	30875529	-	1692	1.848		1
Stk38	ENSMUSG00000024006	chr17	29107830	29144890	-	8489	1.848		1
Ogdhl	ENSMUSG00000021913	chr14	33135205	33161006	+	3449	1.848		1
1700081L11Rik	ENSMUSG00000018412	chr11	104194543	104330175	-	7121	1.846		1
Lrp6	ENSMUSG00000030201	chr6	134396494	134516983	-	9822	1.846		1
Mre11a	ENSMUSG00000031928	chr9	14589098	14641567	+	5902	1.845		1
Ikbkb	ENSMUSG00000031537	chr8	23769684	23817061	-	19088	1.844		1
Cd40	ENSMUSG00000017652	chr2	164881127	164898448	+	3406	1.843		1
Rc3h1	ENSMUSG00000040423	chr1	162836549	162905109	+	11308	1.843		1
Ccdc25	ENSMUSG00000022035	chr14	66456139	66485441	+	2227	1.842		1
Mcl1	ENSMUSG00000038612	chr3	95462643	95467099	+	3485	1.842		1
Fastkd5	ENSMUSG00000079043	chr2	130439576	130455763	-	3015	1.841		1
Tapt1	ENSMUSG00000046985	chr5	44566403	44617845	-	3487	1.84		1
Cdkl2	ENSMUSG00000029403	chr5	92435100	92472068	-	4967	1.839		1
Nln	ENSMUSG00000021710	chr13	104813488	104899694	-	3925	1.839		1
Slc16a11	ENSMUSG00000040938	chr11	70026254	70029919	+	2554	1.838		1
Nop58	ENSMUSG00000026020	chr1	59741853	59768968	+	2587	1.837		1
Fem1c	ENSMUSG00000033319	chr18	46664260	46685625	-	2866	1.837		1
Pdcl	ENSMUSG00000009030	chr2	37205594	37214852	-	3751	1.834		1
Ap1g1	ENSMUSG00000031731	chr8	112302454	112385258	+	4835	1.832		1

Klhdc5	ENSMUSG00000040102	chr6	147039663	147061300	+	6284	1.831	1
Insr	ENSMUSG00000005534	chr8	3150922	3279617	-	9669	1.83	1
Atxn7	ENSMUSG00000021738	chr14	14845005	14939810	+	6847	1.829	1
Ptpla	ENSMUSG00000063275	chr2	13897190	13977762	-	1129	1.829	1
Vrk2	ENSMUSG00000064090	chr11	26371322	26493999	-	6591	1.829	1
Paip2	ENSMUSG00000037058	chr18	35758271	35776840	+	2789	1.829	1
Snx29	ENSMUSG00000071669	chr16	11405741	11755565	+	5246	1.828	1
Gmnn	ENSMUSG00000006715	chr13	24843714	24853792	-	1465	1.827	1
Rap1gap2	ENSMUSG00000038807	chr11	74196858	74403688	-	6635	1.827	1
Fnip2	ENSMUSG00000061175	chr3	79259893	79371718	-	8367	1.826	1
Sema5a	ENSMUSG00000022231	chr15	32174568	32626096	+	12070	1.826	1
Stau2	ENSMUSG00000025920	chr1	16218755	16510193	-	6747	1.826	1
Gabbr1	ENSMUSG00000024462	chr17	37182911	37212012	+	7793	1.825	1
Tfap2a	ENSMUSG00000021359	chr13	40811044	40829192	-	3243	1.825	1
Rnf139	ENSMUSG00000037075	chr15	58720784	58733945	+	4348	1.825	1
Hs3st3b1	ENSMUSG00000070407	chr11	63699294	63735792	-	4911	1.825	1
Calm2	ENSMUSG00000036438	chr17	87832752	87846275	-	1467	1.824	1
Nsmce2	ENSMUSG00000059586	chr15	59205753	59433238	+	3281	1.824	1
Dennd5a	ENSMUSG00000035901	chr7	117037294	117103984	-	8677	1.823	1
Mll2	ENSMUSG00000048154	chr15	98662100	98701614	-	19805	1.822	1
Parg	ENSMUSG00000021911	chr14	33015135	33110736	+	5778	1.822	1
Ufd1l	ENSMUSG00000005262	chr16	18811872	18835354	+	4336	1.822	1
Pbx1	ENSMUSG00000052534	chr1	170050130	170362401	-	13350	1.822	1
Tmem40	ENSMUSG00000059900	chr6	115679149	115712484	-	2196	1.821	1
Srcap	ENSMUSG00000053877	chr7	134655541	134679890	+	4914	1.821	1
Eif3m	ENSMUSG00000027170	chr2	104839813	104857237	-	2590	1.819	1
Sfpi1	ENSMUSG00000002111	chr2	90922547	90955913	+	2695	1.819	1
Tmem87b	ENSMUSG00000014353	chr2	128643854	128679997	+	8395	1.818	1
Scaper	ENSMUSG00000034007	chr9	55397690	55767412	-	5549	1.817	1
Gpr157	ENSMUSG00000047875	chr4	149461474	149480036	+	4900	1.817	1
Gab2	ENSMUSG00000004508	chr7	104230261	104457450	+	5997	1.817	1
Tbce	ENSMUSG00000039233	chr13	14090216	14131905	-	4540	1.816	1
Rnf11	ENSMUSG00000028557	chr4	109123703	109157333	-	4263	1.816	1
Asxl1	ENSMUSG00000042548	chr2	153171581	153229743	+	7431	1.815	1
Elavl1	ENSMUSG00000040028	chr8	4284782	4325100	-	6030	1.814	1
Dcun1d4	ENSMUSG00000051674	chr5	73872241	73952033	+	5803	1.814	1
Ptplad2	ENSMUSG00000028497	chr4	88042048	88084832	-	6756	1.813	1
Dnajc21	ENSMUSG00000044224	chr15	10376511	10400271	-	4777	1.812	1
Tfec	ENSMUSG00000029553	chr6	16783381	16848441	-	1779	1.812	1
Zfp120	ENSMUSG00000068134	chr2	149940142	149962444	-	4376	1.811	1

Klf4	ENSMUSG00000003032	chr4	55540013	55545347	-	3060	1.81	1
Samd8	ENSMUSG00000021770	chr14	22569753	22617947	+	7638	1.807	1
Aebp2	ENSMUSG00000030232	chr6	140570970	140626993	+	8751	1.807	1
Zfp35	ENSMUSG00000063281	chr18	24148133	24163877	+	3040	1.806	1
1700034H14Rik	ENSMUSG00000024645	chr18	85116686	85120916	-	1340	1.806	1
Gm4353	ENSMUSG00000091900	chr7	123226376	123228133	-	1544	1.806	1
Tsc22d1	ENSMUSG00000022010	chr14	76814768	76907572	+	8994	1.805	1
Fus	ENSMUSG00000030795	chr7	135110971	135125882	+	5690	1.805	1
Cul1	ENSMUSG00000029686	chr6	47403397	47476138	+	4058	1.804	1
Zmym4	ENSMUSG00000042446	chr4	126539069	126645379	-	8253	1.804	1
Crybg3	ENSMUSG00000022723	chr16	59490601	59555578	-	7488	1.803	1
3110003A17Rik	ENSMUSG00000078453	chr10	17731069	17743058	-	807	1.803	1
Fam160b2	ENSMUSG00000022095	chr14	70983103	70999642	-	4033	1.802	1
Ddhd2	ENSMUSG00000061313	chr8	26835796	26865068	-	4585	1.8	1
Psmd1	ENSMUSG00000026229	chr1	87960962	88059363	+	5019	1.8	1
Plscr1	ENSMUSG00000032369	chr9	92144895	92166813	+	1692	1.8	1
Rhot1	ENSMUSG00000017686	chr11	80022521	80081409	+	7077	1.799	1
Chac2	ENSMUSG00000020309	chr11	30876707	30886350	-	1315	1.799	1
Cwf19l2	ENSMUSG00000025898	chr9	3404085	3479236	+	3953	1.799	1
Tspan18	ENSMUSG00000027217	chr2	93041917	93174662	-	7778	1.798	1
Fads3	ENSMUSG00000024664	chr19	10116038	10134161	+	3269	1.797	1
Zfp943	ENSMUSG00000053347	chr17	22099519	22208135	+	5277	1.797	1
Ighj1	ENSMUSG00000076621	chr12	114667992	114668044	-	53	1.797	1
Abi3bp	ENSMUSG00000035258	chr16	56477957	56690241	+	4971	1.796	1
Zzz3	ENSMUSG00000039068	chr3	152058437	152125790	+	8462	1.796	1
Nasp	ENSMUSG00000028693	chr4	116273657	116300556	-	7528	1.795	1
Adamtsl4	ENSMUSG00000015850	chr3	95480124	95491840	-	4198	1.795	1
Morf4l1	ENSMUSG00000062270	chr9	89986509	90009658	-	1981	1.794	1
Klhdc2	ENSMUSG00000020978	chr12	70397668	70411674	+	5438	1.794	1
Tmem33	ENSMUSG00000037720	chr5	67651804	67682700	+	7098	1.792	1
Casp2	ENSMUSG00000029863	chr6	42214984	42232507	+	6986	1.792	1
Setd5	ENSMUSG00000034269	chr6	113027359	113103429	+	12481	1.792	1
Cdc14a	ENSMUSG00000033502	chr3	115975471	116126950	-	11335	1.792	1
Slc35e3	ENSMUSG00000060181	chr10	117170735	117183414	-	3482	1.791	1
Sacm1l	ENSMUSG00000025240	chr9	123439000	123501716	+	3433	1.791	1
Krt80	ENSMUSG00000037185	chr15	101177875	101200556	-	4290	1.791	1
Lysmd3	ENSMUSG00000035840	chr13	81796805	81810887	+	3124	1.79	1
Otud1	ENSMUSG00000043415	chr2	19579689	19582217	+	2529	1.789	1

Fat1	ENSMUSG00000070047	chr8	46035562	46137611	+	14778	1.788	1
Fam63b	ENSMUSG00000042444	chr9	70446821	70504981	-	8081	1.784	1
Arhgap23	ENSMUSG00000049807	chr11	97276847	97363716	+	6637	1.783	1
Ddr2	ENSMUSG00000026674	chr1	171902439	172040632	-	8424	1.783	1
Hoxa9	ENSMUSG00000038227	chr6	52173099	52181088	-	3492	1.783	1
Sh3tc2	ENSMUSG00000045629	chr18	62112729	62184019	+	5754	1.782	1
Ano8	ENSMUSG00000034863	chr8	73999922	74009966	-	3753	1.782	1
Afg3l2	ENSMUSG00000024527	chr18	67564421	67608820	-	3085	1.779	1
Cmtm3	ENSMUSG00000031875	chr8	106864494	106871572	+	1616	1.779	1
Irf2bp2	ENSMUSG00000051495	chr8	129112196	129117336	-	4530	1.779	1
Ylpm1	ENSMUSG00000021244	chr12	86337271	86411465	+	9914	1.778	1
Etv5	ENSMUSG00000013089	chr16	22381382	22439792	-	4103	1.778	1
Tnfrsf1b	ENSMUSG00000028599	chr4	144803366	144836773	-	3818	1.778	1
AI314976	ENSMUSG00000040771	chr17	48549339	48556591	+	1336	1.777	1
Ctps	ENSMUSG00000028633	chr4	120212473	120242881	-	3316	1.776	1
Wdr60	ENSMUSG00000042050	chr12	117445523	117501498	-	3725	1.775	1
Pf4	ENSMUSG00000029373	chr5	91201461	91202407	+	586	1.774	1
Ippk	ENSMUSG00000021385	chr13	49516647	49558359	+	2894	1.774	1
Iars	ENSMUSG00000037851	chr13	49777469	49829636	+	5272	1.773	1
Gtf2h1	ENSMUSG00000006599	chr7	54051473	54079170	+	3069	1.773	1
Srsf2	ENSMUSG00000034120	chr11	116711211	116714408	-	2965	1.773	1
Rusc2	ENSMUSG00000035969	chr4	43394851	43439960	+	7923	1.772	1
Smndc1	ENSMUSG00000025024	chr19	53453704	53465063	-	2125	1.771	1
1700020I14Rik	ENSMUSG00000085438	chr2	119420033	119433238	+	8306	1.77	1
Phf14	ENSMUSG00000029629	chr6	11857809	12031197	+	4547	1.77	1
Kpna1	ENSMUSG00000022905	chr16	35978836	36037217	+	8486	1.77	1
Rtn4rl2	ENSMUSG00000050896	chr2	84712081	84726867	-	1506	1.77	1
A530017D24Rik	ENSMUSG00000072847	chr11	61678070	61682270	-	3324	1.768	1
Gpr137b-ps	ENSMUSG00000075118	chr13	12711351	12742564	-	2997	1.768	1
Dhx33	ENSMUSG00000040620	chr11	70797593	70817939	-	5414	1.766	1
Hoxa11	ENSMUSG00000038210	chr6	52192107	52196213	-	2689	1.765	1
Pou2f1	ENSMUSG00000026565	chr1	167795285	167932809	-	16601	1.763	1
Sdhaf2	ENSMUSG00000024668	chr19	10575003	10599699	-	3096	1.763	1
Fhdc1	ENSMUSG00000041842	chr3	84247947	84284351	-	4448	1.762	1
Gnptab	ENSMUSG00000035311	chr10	87841877	87910074	+	5883	1.761	1
Lrig2	ENSMUSG00000032913	chr3	104257907	104315840	-	7752	1.761	1
Ppp1r12a	ENSMUSG00000019907	chr10	107599249	107714631	+	3479	1.76	1
Pdpk1	ENSMUSG00000024122	chr17	24210647	24287891	-	10935	1.759	1
Asxl2	ENSMUSG00000037486	chr12	3426857	3506852	+	14571	1.759	1
Tubb2b	ENSMUSG00000045136	chr13	34218877	34222223	-	1922	1.759	1

Mrc2	ENSMUSG00000020695	chr11	105153957	105212453	+	6674	1.759	1
Zfp74	ENSMUSG00000059975	chr7	30715834	30739108	-	5628	1.757	1
Bicc1	ENSMUSG00000014329	chr10	70385580	70622448	-	7441	1.757	1
Usp24	ENSMUSG00000028514	chr4	105988818	106113927	+	12350	1.757	1
Anubl1	ENSMUSG00000042213	chr6	116214240	116280316	+	3601	1.756	1
Zyg11b	ENSMUSG00000034636	chr4	107902329	107973701	-	9057	1.756	1
Arpc2	ENSMUSG00000006304	chr1	74283071	74314783	+	1794	1.755	1
Tpm3	ENSMUSG00000027940	chr3	89876571	89904824	+	6085	1.754	1
Cox18	ENSMUSG00000035505	chr5	90643749	90653026	-	2009	1.753	1
Lbh	ENSMUSG00000024063	chr17	73267645	73291282	+	3207	1.753	1
Fam84b	ENSMUSG00000072568	chr15	60650551	60656635	-	5563	1.751	1
Msrb3	ENSMUSG00000051236	chr10	120218152	120336157	-	4048	1.751	1
Chuk	ENSMUSG00000025199	chr19	44147824	44181970	-	5088	1.75	1
Ncl	ENSMUSG00000026234	chr1	88241294	88256030	-	8257	1.75	1
8430408G22Rik	ENSMUSG00000048489	chr6	116600702	116602865	+	1317	1.75	1
Zzef1	ENSMUSG00000055670	chr11	72609728	72740622	+	16363	1.75	1
Il33	ENSMUSG00000024810	chr19	29999604	30035208	+	2886	1.75	1
Gcc2	ENSMUSG00000038039	chr10	57718245	57768347	+	9630	1.749	1
Ppard	ENSMUSG00000002250	chr17	28369699	28438414	+	4631	1.748	1
Lonrf1	ENSMUSG00000039633	chr8	37279118	37312570	-	3930	1.748	1
Fzd6	ENSMUSG00000022297	chr15	38837879	38869732	+	4081	1.748	1
Nsd1	ENSMUSG00000021488	chr13	55311143	55419686	+	13123	1.748	1
Tmem119	ENSMUSG00000054675	chr5	114243738	114250525	-	2315	1.748	1
Srgap2	ENSMUSG00000026425	chr1	133181828	133423938	-	8681	1.747	1
Dok3	ENSMUSG00000035711	chr13	55624596	55629899	-	1546	1.745	1
Lmbrd1	ENSMUSG00000073725	chr1	24685383	24823144	+	5390	1.744	1
Sik3	ENSMUSG00000034135	chr9	45820903	46032277	+	7102	1.743	1
Sdad1	ENSMUSG00000029415	chr5	92713036	92739050	-	5020	1.743	1
Rin3	ENSMUSG00000044456	chr12	103521258	103629065	+	6835	1.742	1
Trip13	ENSMUSG00000021569	chr13	74049910	74075215	-	2267	1.741	1
Ddx46	ENSMUSG00000021500	chr13	55736388	55782617	+	5747	1.74	1
Mtmr9	ENSMUSG00000035078	chr14	64142453	64162790	-	2402	1.739	1
Pard3	ENSMUSG00000025812	chr8	129587793	130136186	+	14486	1.739	1
Nsl1	ENSMUSG00000062510	chr1	192886891	192908437	+	3387	1.739	1
Celf2	ENSMUSG00000002107	chr2	6460751	7002348	-	12268	1.738	1
Tecpr2	ENSMUSG00000021275	chr12	112127474	112210604	+	7830	1.737	1
Hcfc2	ENSMUSG00000020246	chr10	82158905	82205173	+	5485	1.736	1
Crim1	ENSMUSG00000024074	chr17	78599588	78775932	+	5995	1.736	1
Ptpn21	ENSMUSG00000021009	chr12	99914951	99975615	-	5738	1.735	1
Dram1	ENSMUSG00000020057	chr10	87785549	87841825	-	3045	1.734	1
Mtfr1	ENSMUSG00000027601	chr3	19087327	19120817	+	4372	1.733	1
Polr2b	ENSMUSG00000029250	chr5	77739172	77778349	+	4667	1.733	1
Dusp14	ENSMUSG00000018648	chr11	83861543	83882763	-	1945	1.733	1

Mtbp	ENSMUSG00000022369	chr15	55388963	55457978	+	5481	1.733	1
Gbgt1	ENSMUSG00000026829	chr2	28352411	28360935	+	1337	1.733	1
Ube2t	ENSMUSG00000026429	chr1	136859142	136870739	+	1784	1.732	1
Gm7251	ENSMUSG00000069236	chr13	49900313	49901320	-	1008	1.732	1
Ubxn2a	ENSMUSG00000020634	chr12	4885838	4914511	-	2820	1.731	1
Mfsd1	ENSMUSG00000027775	chr3	67386690	67408153	+	2978	1.731	1
Laptm5	ENSMUSG00000028581	chr4	130469040	130492056	+	3788	1.731	1
Map2k6	ENSMUSG00000020623	chr11	110260436	110386836	+	3444	1.731	1
Ppp2r2a	ENSMUSG00000022052	chr14	67633511	67691272	-	2971	1.729	1
Exosc8	ENSMUSG00000027752	chr3	54532600	54539315	-	3968	1.729	1
Rif1	ENSMUSG00000036202	chr2	51928357	51977903	+	10527	1.729	1
Epas1	ENSMUSG00000024140	chr17	87153247	87232750	+	5309	1.728	1
Rps6ka2	ENSMUSG00000023809	chr17	7374464	7507662	+	5677	1.727	1
Serpinb6a	ENSMUSG00000060147	chr13	34009787	34094663	-	5409	1.727	1
Cav1	ENSMUSG00000007655	chr6	17256335	17291452	+	3444	1.725	1
Epc2	ENSMUSG00000069495	chr2	49307006	49407468	+	4697	1.725	1
Ofd1	ENSMUSG00000040586	chrX	162827965	162878636	-	4453	1.725	1
Myst4	ENSMUSG00000021767	chr14	22319076	22491354	+	9566	1.725	1
Elk4	ENSMUSG00000026436	chr1	133904184	133929189	+	5483	1.725	1
Thumpd1	ENSMUSG00000030942	chr7	126858610	126864279	-	2653	1.723	1
Atl2	ENSMUSG00000059811	chr17	80247732	80295463	-	4146	1.723	1
Tmem70	ENSMUSG00000025940	chr1	16655288	16668356	+	1820	1.723	1
Wdr1	ENSMUSG00000005103	chr5	38918053	38952905	-	2948	1.723	1
Kdm3b	ENSMUSG00000038773	chr18	34936662	34999024	+	7717	1.722	1
Gm15445	ENSMUSG00000085311	chr1	137049540	137050383	+	603	1.721	1
Strn3	ENSMUSG00000020954	chr12	52709528	52792901	-	4223	1.721	1
Tfdp1	ENSMUSG00000038482	chr8	13338751	13378395	+	2542	1.72	1
Slc15a3	ENSMUSG00000024737	chr19	10914217	10933852	+	5491	1.72	1
Rbm3	ENSMUSG00000031167	chrX	7716101	7723006	-	4698	1.72	1
Rassf4	ENSMUSG00000042129	chr6	116583026	116623854	-	6344	1.72	1
Gosr1	ENSMUSG00000010392	chr11	76540104	76577081	-	4289	1.72	1
8430427H17Rik	ENSMUSG00000061411	chr2	153233198	153355707	-	9083	1.718	1
2410042D21Rik	ENSMUSG00000027132	chr2	112219368	112254397	+	4051	1.718	1
Rpap2	ENSMUSG00000033773	chr5	108026392	108090857	+	3893	1.717	1
Rab5a	ENSMUSG00000017831	chr17	53618559	53646996	+	2357	1.717	1
Nudcd1	ENSMUSG00000038736	chr15	44206773	44259853	-	5806	1.716	1
Hpse	ENSMUSG00000035273	chr5	101108503	101148735	-	3516	1.716	1

Nlgn2	ENSMUSG00000051790	chr11	69636624	69651286	-	5785	1.716	1
Zfp790	ENSMUSG00000011427	chr7	30601091	30617061	+	4661	1.716	1
Golm1	ENSMUSG00000021556	chr13	59736357	59777145	-	3693	1.715	1
Gtpbp10	ENSMUSG00000040464	chr5	5537454	5559538	-	4744	1.715	1
A430104N18Rik	ENSMUSG00000084796	chr11	87569079	87570772	+	269	1.715	1
Il10rb	ENSMUSG00000022969	chr16	91406409	91426079	+	3808	1.715	1
Tra2b	ENSMUSG00000022858	chr16	22244622	22266078	-	5383	1.715	1
Fzd1	ENSMUSG00000044674	chr5	4753842	4758216	-	4375	1.714	1
Pcmtd1	ENSMUSG00000051285	chr1	7079001	7163707	+	5288	1.714	1
Akap8	ENSMUSG00000024045	chr17	32440621	32458098	-	3695	1.713	1
Mdk	ENSMUSG00000027239	chr2	91769962	91772454	-	1337	1.711	1
Cdo1	ENSMUSG00000033022	chr18	46872859	46887996	-	1530	1.711	1
Socs1	ENSMUSG00000038037	chr16	10783901	10785629	-	1220	1.71	1
Gsk3b	ENSMUSG00000022812	chr16	38089087	38246170	+	9831	1.71	1
Rac1	ENSMUSG00000001847	chr5	144266346	144288904	-	2652	1.709	1
Socs4	ENSMUSG00000048379	chr14	47896606	47915773	+	6873	1.709	1
Kdelc2	ENSMUSG00000034487	chr9	53192128	53209972	+	3644	1.709	1
Serinc3	ENSMUSG00000017707	chr2	163449008	163470867	-	3840	1.708	1
Bbip1	ENSMUSG00000084957	chr19	54003757	54019117	-	2653	1.708	1
Nfya	ENSMUSG00000023994	chr17	48526210	48549231	-	7315	1.708	1
Ing2	ENSMUSG00000063049	chr8	48752532	48760910	-	3019	1.708	1
Baz2a	ENSMUSG00000040054	chr10	127496046	127566359	+	8488	1.708	1
Stmn1	ENSMUSG00000028832	chr4	134024235	134029758	+	1922	1.707	1
Scoc	ENSMUSG00000063253	chr8	85958392	85982290	-	2062	1.707	1
Slc38a6	ENSMUSG00000044712	chr12	74387766	74455036	+	7783	1.706	1
Rnf115	ENSMUSG00000028098	chr3	96531534	96595078	+	2265	1.705	1
Acadm	ENSMUSG00000062908	chr3	153585321	153607596	-	2932	1.705	1
Fermt2	ENSMUSG00000037712	chr14	46078467	46149793	-	3747	1.705	1
Atp6ap2	ENSMUSG00000031007	chrX	12164927	12194175	+	2618	1.704	1
Obfc1	ENSMUSG00000042694	chr19	47575528	47611528	-	2349	1.703	1
Coro2a	ENSMUSG00000028337	chr4	46549809	46615074	-	6459	1.703	1
Heph	ENSMUSG00000031209	chrX	93650698	93769824	+	7662	1.701	1
Btbd1	ENSMUSG00000025103	chr7	88936961	88974317	-	3000	1.7	1
Tom1l1	ENSMUSG00000020541	chr11	90504779	90549680	-	7749	1.699	1
Tbcel	ENSMUSG00000037287	chr9	42220399	42315892	-	5556	1.698	1
Tcf12	ENSMUSG00000032228	chr9	71692059	71959626	-	4861	1.697	1
Ppp1cb	ENSMUSG00000014956	chr5	32761347	32819806	+	7004	1.697	1
Rabgap1	ENSMUSG00000035437	chr2	37298799	37421974	+	7607	1.696	1
Phactr4	ENSMUSG00000066043	chr4	131911838	131978404	-	4960	1.696	1
Dcun1d1	ENSMUSG00000027708	chr3	35791900	35836367	-	4431	1.695	1
Lass2	ENSMUSG00000015714	chr3	95118714	95127510	+	3665	1.695	1

Orc6	ENSMUSG00000031697	chr8	87823531	87832177	+	1653	1.694		1
Amotl2	ENSMUSG00000032531	chr9	102619002	102635748	+	6289	1.694		1
Gm608	ENSMUSG00000068284	chr16	44173359	44227578	+	13050	1.693		1
Ciita	ENSMUSG00000022504	chr16	10480237	10527657	+	6208	1.692		1
Ifih1	ENSMUSG00000026896	chr2	62433855	62484312	-	8165	1.691		1
Mysm1	ENSMUSG00000062627	chr4	94608731	94645791	-	8946	1.691		1
Tia1	ENSMUSG00000071337	chr6	86354213	86383397	+	12945	1.69		1
Plekhg1	ENSMUSG00000040624	chr10	6379238	6606177	-	8739	1.69		1
Tcp1	ENSMUSG00000068039	chr17	13108567	13117933	+	5030	1.689		1
Svep1	ENSMUSG00000028369	chr4	58055314	58219731	-	16117	1.688		1
Wdfy4	ENSMUSG00000051506	chr14	33772733	33998694	-	10512	1.688		1
Lpcat1	ENSMUSG00000021608	chr13	73604645	73653870	+	4131	1.687		1
Itga1	ENSMUSG00000042284	chr13	115748287	115892172	-	8001	1.687		1
Cenpn	ENSMUSG00000031756	chr8	119445640	119465403	+	1768	1.686		1
Ptar1	ENSMUSG00000074925	chr19	23761890	23795619	+	2064	1.686		1
Zufsp	ENSMUSG00000039531	chr10	33638953	33671065	-	2640	1.686		1
Acbd5	ENSMUSG00000026781	chr2	22923688	22970030	+	4155	1.686		1
Arhgef4 0	ENSMUSG00000004562	chr14	52604508	52625922	+	5646	1.686		1
Ifi203	ENSMUSG00000039997	chr1	175850538	175872803	-	6706	1.686		1
Rad17	ENSMUSG00000021635	chr13	101387120	101421014	-	2846	1.683		1
Ppp3cb	ENSMUSG00000021816	chr14	21318586	21365795	-	6018	1.683		1
Ddx19a	ENSMUSG00000015023	chr8	113498896	113521700	-	2957	1.683		1
Zmym1	ENSMUSG00000043872	chr4	126724338	126738396	-	4180	1.682		1
Hoxa3	ENSMUSG00000079560	chr6	52118509	52163335	-	6566	1.682		1
Fam96a	ENSMUSG00000032381	chr9	65974418	65986762	+	1240	1.681		1
Arsa	ENSMUSG00000022620	chr15	89302907	89307856	-	3680	1.681		1
Hs2st1	ENSMUSG00000040151	chr3	144094067	144233145	-	4916	1.68		1
Rhbdd1	ENSMUSG00000026142	chr1	82313027	82441941	+	3884	1.679		1
Armc8	ENSMUSG00000032468	chr9	99378810	99469318	-	4628	1.678		1
Esd	ENSMUSG00000021996	chr14	75132104	75150572	+	3366	1.677		1
Ttc17	ENSMUSG00000027194	chr2	94140924	94246846	-	8984	1.677		1
Rbm39	ENSMUSG00000027620	chr2	155972975	156005974	-	6946	1.677		1
Nol8	ENSMUSG00000021392	chr13	49748719	49774384	+	4242	1.676		1
2810407 C02Rik	ENSMUSG00000075700	chr3	58380558	58397055	+	3436	1.676		1
Uap1	ENSMUSG00000026670	chr1	172072069	172105088	-	3779	1.676		1
Map3k6	ENSMUSG00000028862	chr4	132796733	132808844	+	4604	1.675		1
Capza1	ENSMUSG00000070372	chr3	104625697	104667423	-	3532	1.675		1
Nuak1	ENSMUSG00000020032	chr10	83833650	83903342	-	5625	1.674		1
Smurf1	ENSMUSG00000038780	chr5	145637364	145726716	-	6336	1.674		1
Cct2	ENSMUSG00000034024	chr10	116488054	116500870	-	1962	1.673		1
Orc2	ENSMUSG00000026037	chr1	58519615	58561953	-	3857	1.673		1
Lsm14b	ENSMUSG00000039108	chr2	179759692	179770170	+	2892	1.673		1
Sap30	ENSMUSG00000031609	chr8	59961504	59966657	-	1178	1.67		1

Mcts2	ENSMUSG00000042814	chr2	152512884	152513676	+	793	1.67	1
Gdi2	ENSMUSG00000021218	chr13	3537321	3565507	+	2681	1.669	1
Slc36a4	ENSMUSG00000043885	chr9	15514182	15546828	+	5563	1.669	1
Ids	ENSMUSG00000035847	chrX	67596244	67618260	-	5102	1.668	1
Herc1	ENSMUSG00000038664	chr9	66198257	66356582	+	27464	1.668	1
Usp22	ENSMUSG00000042506	chr11	60965287	60988557	-	4886	1.667	1
Pdha1	ENSMUSG00000031299	chrX	156560141	156576345	-	3774	1.666	1
Birc5	ENSMUSG00000017716	chr11	117710565	117717069	+	3414	1.665	1
Prps1l3	ENSMUSG00000079104	chr12	58331399	58340869	+	1720	1.665	1
Gm17491	ENSMUSG00000078859	chr8	23590763	23593802	+	2309	1.663	1
Ing3	ENSMUSG00000029670	chr6	21899571	21926038	+	4806	1.662	1
Tpp1	ENSMUSG00000030894	chr7	112893334	112900738	-	3538	1.662	1
Gtf2f1	ENSMUSG00000002658	chr17	57142826	57150723	-	1731	1.661	1
4933411K20Rik	ENSMUSG00000031631	chr8	47237125	47280944	+	5157	1.66	1
Dclre1b	ENSMUSG00000027845	chr3	103604528	103613367	-	8152	1.66	1
Hnrnph3	ENSMUSG00000020069	chr10	62477412	62486965	-	3151	1.66	1
Podxl	ENSMUSG00000025608	chr6	31469488	31513981	-	5488	1.66	1
Trim65	ENSMUSG00000054517	chr11	115983160	115992442	-	5883	1.659	1
2310028H24Rik	ENSMUSG00000028439	chr4	41464724	41516571	-	3724	1.658	1
Gramd2	ENSMUSG00000074259	chr9	59527951	59566681	+	6138	1.656	1
Arhgap12	ENSMUSG00000041225	chr18	6024446	6136096	-	5049	1.654	1
Txndc17	ENSMUSG00000020803	chr11	72021049	72023517	+	1324	1.654	1
Kcna5	ENSMUSG00000045534	chr6	126482571	126485573	-	3003	1.654	1
Eif4g3	ENSMUSG00000028760	chr4	137548937	137764423	+	11042	1.653	1
Sp1	ENSMUSG00000001280	chr15	102236574	102266835	+	8325	1.653	1
Mafg	ENSMUSG00000051510	chr11	120486431	120494914	-	5613	1.652	1
Extl2	ENSMUSG00000027963	chr3	115710380	115731934	+	3446	1.651	1
Uaca	ENSMUSG00000034485	chr9	60642355	60728177	+	4428	1.651	1
Cdyl2	ENSMUSG00000031758	chr8	119098301	119256891	-	2410	1.65	1
1600012H06Rik	ENSMUSG00000050088	chr17	15080189	15096575	+	3294	1.648	1
Col6a2	ENSMUSG00000020241	chr10	76058507	76086375	-	8326	1.647	1
Ube2e1	ENSMUSG00000021774	chr14	19115236	19164373	-	4457	1.647	1
Rapgef1	ENSMUSG00000039844	chr2	29475240	29596498	+	7389	1.646	1
Fktn	ENSMUSG00000028414	chr4	53726870	53778657	+	6489	1.646	1
2900026A02Rik	ENSMUSG00000051339	chr5	113515343	113592371	-	5876	1.646	1
Ikzf5	ENSMUSG00000040167	chr7	138532166	138554035	-	4885	1.646	1

Psmd6	ENSMUSG00000021737	chr14	14944698	14953498	-	2326	1.646	1
Fam57a	ENSMUSG00000069808	chr11	75993173	76028329	+	6615	1.646	1
Serinc5	ENSMUSG00000021703	chr13	93381093	93481899	+	5316	1.645	1
Zfp429	ENSMUSG00000078994	chr13	67490263	67500703	-	2033	1.645	1
Prrc2b	ENSMUSG00000039262	chr2	32006602	32090057	+	11264	1.644	1
Celf4	ENSMUSG00000024268	chr18	25636133	25912484	-	3998	1.644	1
Ncoa4	ENSMUSG00000056234	chr14	32973051	32993041	+	4452	1.644	1
Lypla1	ENSMUSG00000025903	chr1	4797869	4876851	+	7341	1.642	1
Trim8	ENSMUSG00000025034	chr19	46576138	46590934	+	3230	1.64	1
Nek9	ENSMUSG00000034290	chr12	86640464	86680312	-	5386	1.64	1
Aplf	ENSMUSG00000030051	chr6	87578423	87622162	-	3278	1.639	1
Tfb2m	ENSMUSG00000026492	chr1	181458186	181476398	-	2804	1.639	1
Slc35a1	ENSMUSG00000028293	chr4	34610506	34634687	-	2293	1.638	1
Plekhg3	ENSMUSG00000052609	chr12	77634547	77680026	+	5042	1.638	1
Rhoh	ENSMUSG00000029204	chr5	66254808	66287939	+	4960	1.637	1
Hjurp	ENSMUSG00000044783	chr1	90155690	90174208	-	9026	1.637	1
Ddx3y	ENSMUSG00000069045	chrY	597158	623056	-	4640	1.637	1
Sh3d19	ENSMUSG00000028082	chr3	85888356	85934444	+	6431	1.636	1
Pvrl1	ENSMUSG00000032012	chr9	43552659	43615537	+	5054	1.636	1
Naf1	ENSMUSG00000014907	chr8	69384116	69414463	+	2990	1.636	1
Usp43	ENSMUSG00000020905	chr11	67668025	67735655	-	4745	1.635	1
Eif2a	ENSMUSG00000027810	chr3	58329743	58361423	+	6118	1.635	1
Hspg2	ENSMUSG00000028763	chr4	137024634	137126545	+	14357	1.635	1
Mapkbp1	ENSMUSG00000033902	chr2	119798435	119853144	+	7602	1.633	1
Srsf3	ENSMUSG00000071172	chr17	29169618	29180311	+	5109	1.633	1
Lpp	ENSMUSG00000033306	chr16	24393436	24992662	+	15994	1.632	1
Eda2r	ENSMUSG00000034457	chrX	94529179	94572555	-	5891	1.632	1
Sema4c	ENSMUSG00000026121	chr1	36605487	36615194	-	4590	1.631	1
Ccdc47	ENSMUSG00000078622	chr11	106058722	106077658	-	6414	1.63	1
Eapp	ENSMUSG00000054302	chr12	55771327	55796884	-	4448	1.628	1
Bms1	ENSMUSG00000030138	chr6	118333401	118369435	-	4286	1.627	1
Acyp2	ENSMUSG00000060923	chr11	30405991	30549587	-	863	1.627	1
Arhgef17	ENSMUSG00000032875	chr7	108018260	108080675	-	10394	1.626	1
Nphp1	ENSMUSG00000027378	chr2	127566468	127614633	-	2708	1.624	1
Smarca5	ENSMUSG00000031715	chr8	83222406	83263396	-	7777	1.624	1
Crcp	ENSMUSG00000025532	chr5	130505160	130536659	+	1579	1.624	1
Mtrf1l	ENSMUSG00000019774	chr10	4522631	4534654	+	3261	1.623	1
Mpp1	ENSMUSG00000031402	chrX	72355072	72376355	-	5343	1.623	1
Dnmt1	ENSMUSG00000004099	chr9	20711650	20757317	-	5264	1.622	1
Eml1	ENSMUSG00000058070	chr12	109609167	109777827	+	7436	1.621	1
Dr1	ENSMUSG00000029265	chr5	108697916	108709545	+	3478	1.62	1

Foxj3	ENSMUSG00000032998	chr4	119209609	119301724	+	10973	1.62	1
Zkscan5	ENSMUSG00000055991	chr5	145965431	145982619	+	4114	1.62	1
Pcgf6	ENSMUSG00000025050	chr19	47108109	47125380	-	4085	1.62	1
Atg5	ENSMUSG00000038160	chr10	43988164	44084097	+	2364	1.62	1
Tob2	ENSMUSG00000048546	chr15	81678700	81688756	-	3994	1.619	1
Bcl3	ENSMUSG00000053175	chr7	20393811	20408119	-	2485	1.619	1
Ankrd52	ENSMUSG00000014498	chr10	127814171	127845760	+	8401	1.619	1
Wipi1	ENSMUSG00000041895	chr11	109434645	109473281	-	4410	1.618	1
Rac2	ENSMUSG00000033220	chr15	78389599	78403213	-	3036	1.618	1
Mbd2	ENSMUSG00000024513	chr18	70727843	70785785	+	3214	1.617	1
1700029F09Rik	ENSMUSG00000026049	chr1	44143458	44159286	-	1926	1.617	1
Sass6	ENSMUSG00000027959	chr3	116297926	116333902	+	4241	1.617	1
Flt4	ENSMUSG00000020357	chr11	49422765	49466241	+	8232	1.617	1
Mfsd6	ENSMUSG00000041439	chr1	52713130	52784306	-	6816	1.617	1
Fos	ENSMUSG00000021250	chr12	86814840	86818223	+	2352	1.617	1
Nek1	ENSMUSG00000031644	chr8	63471992	63610143	+	7886	1.616	1
Ibtk	ENSMUSG00000035941	chr9	85580963	85642830	-	5637	1.616	1
BC005764	ENSMUSG00000035835	chr10	79323220	79337379	-	4357	1.616	1
Gzf1	ENSMUSG00000027439	chr2	148506759	148518685	+	4472	1.615	1
Gng12	ENSMUSG00000036402	chr6	66846391	66971344	+	4694	1.615	1
AI597479	ENSMUSG00000010290	chr1	43155555	43172792	+	3458	1.615	1
Parp3	ENSMUSG00000023249	chr9	106372653	106379280	-	3699	1.615	1
2210404O09Rik	ENSMUSG00000078546	chr17	22016537	22045059	-	1744	1.615	1
Fbxw11	ENSMUSG00000020271	chr11	32542724	32646816	+	4327	1.614	1
Arhgap10	ENSMUSG00000037148	chr8	79774265	80041806	-	3038	1.613	1
Arhgap5	ENSMUSG00000035133	chr12	53617064	53668839	+	6643	1.613	1
Ptprb	ENSMUSG00000020154	chr10	115712579	115826594	+	13200	1.613	1
Eya2	ENSMUSG00000017897	chr2	165480798	165597227	+	2730	1.612	1
Ptgr2	ENSMUSG00000072946	chr12	85626182	85656782	+	5929	1.612	1
Ppfia1	ENSMUSG00000037519	chr7	151662660	151739634	-	5340	1.609	1
4933426M11Rik	ENSMUSG00000021133	chr12	81891546	81981819	+	5037	1.608	1
Rnpc3	ENSMUSG00000027981	chr3	113307985	113333067	-	6627	1.608	1
Cnst	ENSMUSG00000038949	chr1	181476501	181557609	+	8859	1.608	1
Rbm6	ENSMUSG00000032582	chr9	107675896	107775150	-	3983	1.608	1
Rps6ka5	ENSMUSG00000021180	chr12	101787988	101963238	-	4406	1.608	1
Crem	ENSMUSG00000063889	chr18	3266046	3337746	-	5937	1.607	1
Med21	ENSMUSG00000030291	chr6	146591069	146599124	+	2102	1.607	1

Niacr1	ENSMUSG00000045502	chr5	124313581	124315518	-	1938	1.607	1
Zfp874a	ENSMUSG00000069206	chr13	67527195	67552560	-	3441	1.607	1
Hilpda	ENSMUSG00000043421	chr6	29222488	29225446	+	1103	1.605	1
Rrp15	ENSMUSG00000001305	chr1	188544857	188573237	-	3239	1.605	1
Gspt1	ENSMUSG00000062203	chr16	11216336	11254418	-	7401	1.605	1
AI848100	ENSMUSG00000040297	chr1	163746243	163806792	-	6308	1.605	1
Homer3	ENSMUSG00000003573	chr8	72806726	72818260	+	3440	1.604	1
6720463M24Rik	ENSMUSG00000022070	chr14	99445646	99473756	+	2673	1.603	1
5730455O13Rik	ENSMUSG00000054237	chr19	38262971	38298628	-	1402	1.603	1
Sumo1	ENSMUSG00000026021	chr1	59696278	59727678	-	1736	1.6	1
Uck2	ENSMUSG00000053664	chr1	169153958	169154050	-	93	1.6	1
Uchl5	ENSMUSG00000018189	chr1	145624408	145654596	+	1851	1.6	1
Sgms1	ENSMUSG00000040451	chr19	32197217	32464204	-	10309	1.6	1
Lmtk2	ENSMUSG00000038970	chr5	144861305	144949073	+	8114	1.598	1
Ubqln2	ENSMUSG00000050148	chrX	149932775	149936080	+	3306	1.598	1
Tspan9	ENSMUSG00000030352	chr6	127911414	128093612	-	4732	1.597	1
Stab1	ENSMUSG00000042286	chr14	31952199	31981827	-	10288	1.597	1
Ppp1r2	ENSMUSG00000047714	chr16	31251623	31275363	-	4440	1.596	1
Zmynd11	ENSMUSG00000021156	chr13	9684082	9764567	-	7055	1.596	1
Wt1	ENSMUSG00000016458	chr2	104966686	105013773	+	6195	1.596	1
Fzd7	ENSMUSG00000041075	chr1	59538991	59543791	+	4801	1.595	1
Yy1	ENSMUSG00000021264	chr12	110031183	110054842	+	2902	1.595	1
Fam178a	ENSMUSG00000036097	chr19	45005609	45058277	+	7224	1.595	1
Huwe1	ENSMUSG00000025261	chrX	148235350	148369960	+	23509	1.594	1
Rabep1	ENSMUSG00000020817	chr11	70658265	70756607	+	5848	1.594	1
Abhd5	ENSMUSG00000032540	chr9	122260726	122290642	+	3618	1.594	1
Ccz1	ENSMUSG00000029617	chr5	144748785	144775722	-	1762	1.592	1
Hspa14	ENSMUSG00000051396	chr2	3406122	3430086	-	8083	1.591	1
Vma21	ENSMUSG00000073131	chrX	69061263	69085096	+	7303	1.591	1
Mier3	ENSMUSG00000032727	chr13	112476386	112508804	+	5846	1.59	1
Nbr1	ENSMUSG00000017119	chr11	101413463	101443265	+	6814	1.59	1
Ccnt2	ENSMUSG00000026349	chr1	129670741	129704638	+	12345	1.59	1
Mcmbp	ENSMUSG00000048170	chr7	135839955	135884009	-	5107	1.588	1
Zfp426	ENSMUSG00000059475	chr9	20272993	20297190	-	4888	1.588	1
Yap1	ENSMUSG00000053110	chr9	7931999	8004596	-	9257	1.588	1
Avpr1a	ENSMUSG00000020123	chr10	121885555	121890509	+	2671	1.587	1
Ttll3	ENSMUSG00000030276	chr6	113339254	113364577	+	5059	1.586	1
Il1rl2	ENSMUSG00000070942	chr1	40381472	40422316	+	1950	1.586	1
Pglyrp1	ENSMUSG00000030413	chr7	19470039	19475787	+	681	1.585	1
Rabggtb	ENSMUSG00000038975	chr3	153570253	153575930	-	1619	1.585	1

Itga9	ENSMUSG00000039115	chr9	118515808	118810121	+	7691	1.585		1
Eif4enif1	ENSMUSG00000020454	chr11	3102395	3144591	+	6344	1.585		1
Ppp1r9a	ENSMUSG00000032827	chr6	4852917	5115661	+	14531	1.584		1
AI314180	ENSMUSG00000050812	chr4	58811783	58925621	-	11447	1.583		1
Zfp938	ENSMUSG00000062931	chr10	81687595	81704025	-	2128	1.582		1
Cdc5l	ENSMUSG00000023932	chr17	45528841	45570656	-	2989	1.582		1
S100a14	ENSMUSG00000042306	chr3	90330778	90332755	+	1086	1.582		1
Cwc22	ENSMUSG00000027014	chr2	77719216	77784432	-	6822	1.581		1
Foxp1	ENSMUSG00000030067	chr6	98875332	99472715	-	14266	1.58		1
Fam83g	ENSMUSG00000042377	chr11	61497593	61523453	+	6659	1.58		1
B130055M24Rik	ENSMUSG00000086502	chr7	134592150	134603874	+	1988	1.579		1
Gmeb1	ENSMUSG00000028901	chr4	131776940	131817517	-	7857	1.579		1
Xrcc5	ENSMUSG00000026187	chr1	72353995	72441526	+	2555	1.579		1
Naa25	ENSMUSG00000042719	chr5	121847945	121894387	+	8298	1.579		1
Sec22c	ENSMUSG00000061536	chr9	121589165	121614608	-	6301	1.579		1
Zfp180	ENSMUSG00000057101	chr7	24866943	24892717	+	3886	1.578		1
Zfp867	ENSMUSG00000054519	chr11	59274699	59285976	-	3419	1.577		1
Zdhhc5	ENSMUSG00000034075	chr2	84528127	84555337	-	5047	1.577		1
Mink1	ENSMUSG00000020827	chr11	70376383	70427985	+	6325	1.577		1
B4galt1	ENSMUSG00000028413	chr4	40751635	40801038	-	4234	1.577		1
Ndel1	ENSMUSG00000018736	chr11	68634936	68685360	-	3253	1.576		1
R3hdm1	ENSMUSG00000056211	chr1	129999883	130134312	+	6758	1.576		1
Dapp1	ENSMUSG00000028159	chr3	137593971	137644509	-	3284	1.575		1
Dcun1d3	ENSMUSG00000048787	chr7	126996677	127039805	-	6203	1.575		1
1500031L02Rik	ENSMUSG00000035790	chr16	32099886	32108155	+	1830	1.575		1
Tmem194	ENSMUSG00000040195	chr10	127103957	127138105	+	7822	1.575		1
BC032203	ENSMUSG00000036568	chr17	46935065	46968362	-	6579	1.573		1
Mrpl35	ENSMUSG00000052962	chr6	71762997	71773804	-	3715	1.572		1
Baz2b	ENSMUSG00000026987	chr2	59737420	60047896	-	17605	1.572		1
Prcp	ENSMUSG00000061119	chr7	100023763	100083083	+	2956	1.572		1
Arhgef7	ENSMUSG00000031511	chr8	11728105	11835219	+	13075	1.572		1
Pfn2	ENSMUSG00000027805	chr3	57645817	57651486	-	3017	1.571		1
Mkln1	ENSMUSG00000025609	chr6	31348735	31466811	+	22296	1.571		1
5730455P16Rik	ENSMUSG00000057181	chr11	80173995	80191536	-	4839	1.57		1
Rrp1	ENSMUSG00000061032	chr10	77863107	77875788	-	2008	1.57		1

Phax	ENSMUSG00000008301	chr18	56722097	56747366	+	2223	1.57	1
Camkk1	ENSMUSG00000020785	chr11	72832510	72855575	+	3728	1.569	1
Tars	ENSMUSG00000022241	chr15	11313418	11329413	-	2636	1.568	1
Sqle	ENSMUSG00000022351	chr15	59146632	59162747	+	5546	1.567	1
Mtmr1	ENSMUSG00000015214	chrX	68617935	68672371	+	5859	1.567	1
Mtfmt	ENSMUSG00000059183	chr9	65283589	65300861	+	3655	1.566	1
Tulp4	ENSMUSG00000034377	chr17	6106437	6251128	+	18054	1.565	1
2610301B20Rik	ENSMUSG00000059482	chr4	10801645	10826572	+	2391	1.565	1
Mical3	ENSMUSG00000003178	chr6	120951381	121031627	-	3315	1.563	1
Btf3l4	ENSMUSG00000028568	chr4	108486900	108506219	-	4546	1.563	1
Abhd13	ENSMUSG00000040396	chr8	9977707	9992155	+	5191	1.563	1
Ncoa3	ENSMUSG00000027678	chr2	165818136	165898742	+	7895	1.563	1
Fam149b	ENSMUSG00000039599	chr14	21167384	21202711	+	4335	1.562	1
Lyar	ENSMUSG00000067367	chr5	38611709	38625545	+	2401	1.56	1
Xrcc2	ENSMUSG00000028933	chr5	25195630	25211643	-	5216	1.559	1
Tmem87a	ENSMUSG00000033808	chr2	120181045	120229852	-	7534	1.558	1
2410002O22Rik	ENSMUSG00000021711	chr13	104932229	104968549	-	5148	1.557	1
C330007P06Rik	ENSMUSG00000006423	chrX	34363732	34414106	-	7431	1.557	1
Nipa1	ENSMUSG00000047037	chr7	63232937	63275324	-	3183	1.557	1
Rbm27	ENSMUSG00000024491	chr18	42435007	42501196	+	6431	1.556	1
4933433P14Rik	ENSMUSG00000044715	chr12	106923562	106941257	+	2790	1.556	1
Il1r1	ENSMUSG00000026072	chr1	40281925	40373043	+	5056	1.556	1
Ttc33	ENSMUSG00000022151	chr15	5135525	5168336	+	2163	1.556	1
Vprbp	ENSMUSG00000040325	chr9	106724205	106785649	+	8091	1.556	1
Rhoq	ENSMUSG00000024143	chr17	87362422	87399409	+	4151	1.555	1
Pcnp	ENSMUSG00000071533	chr16	56007358	56029852	-	3115	1.555	1
Zfp106	ENSMUSG00000027288	chr2	120332556	120389579	-	11036	1.554	1
Enpp3	ENSMUSG00000019989	chr10	24493620	24556001	-	2815	1.554	1
Tab3	ENSMUSG00000035476	chrX	82819361	82879808	+	6639	1.552	1
Rbpj	ENSMUSG00000039191	chr5	53947018	54048684	+	5817	1.551	1
Cald1	ENSMUSG00000029761	chr6	34548500	34725473	+	7687	1.551	1
Sec23ip	ENSMUSG00000055319	chr7	135888384	135928350	+	4578	1.55	1
Mndal	ENSMUSG00000090272	chr1	175787352	175872622	-	1907	1.549	1
Prmt2	ENSMUSG00000020230	chr10	75669967	75700610	-	3177	1.549	1
Ncor2	ENSMUSG00000029478	chr5	125497523	125659589	-	10479	1.549	1
Asb1	ENSMUSG00000026311	chr1	93437142	93456167	+	6072	1.549	1
Crabp2	ENSMUSG00000004885	chr3	87752588	87757298	+	1322	1.549	1

Bend7	ENSMUSG00000048186	chr2	4638877	4723188	+	4194	1.548	1
Mcam	ENSMUSG00000032135	chr9	43942552	43950810	+	3350	1.543	1
Srek1	ENSMUSG00000032621	chr13	104531695	104564539	-	4280	1.542	1
Ssh1	ENSMUSG00000042121	chr5	114387103	114443903	-	8892	1.542	1
Brwd1	ENSMUSG00000022914	chr16	96213699	96304133	-	20280	1.541	1
Vangl1	ENSMUSG00000027860	chr3	101960624	102008616	-	4038	1.541	1
Mphosph6	ENSMUSG00000031843	chr8	120315545	120325829	-	1054	1.54	1
Gemin6	ENSMUSG00000055760	chr17	80623781	80627837	+	1428	1.539	1
Gm12355	ENSMUSG00000078134	chr11	98485665	98486975	-	1311	1.539	1
Leprot	ENSMUSG00000035212	chr4	101320323	101331969	+	1898	1.538	1
Rala	ENSMUSG00000008859	chr13	17972409	18036073	-	2684	1.538	1
Bag5	ENSMUSG00000049792	chr12	112947699	112951468	-	2508	1.537	1
Mocs2	ENSMUSG00000015536	chr13	115608452	115619214	+	2485	1.537	1
Klhdc8a	ENSMUSG00000042115	chr1	134195203	134203934	+	4269	1.537	1
Antxr1	ENSMUSG00000033420	chr6	87083848	87285769	-	5401	1.536	1
AU041133	ENSMUSG00000078435	chr10	81600998	81614756	+	1494	1.536	1
Ndufaf4	ENSMUSG00000028261	chr4	24825230	24832148	+	3902	1.535	1
Las1l	ENSMUSG00000057421	chrX	93130674	93152301	-	8143	1.535	1
Tspyl1	ENSMUSG00000047514	chr10	34001996	34004691	+	2696	1.535	1
Akirin2	ENSMUSG00000028291	chr4	34498186	34514157	+	1711	1.534	1
Acaca	ENSMUSG00000020532	chr11	83943174	84215153	+	10189	1.533	1
Fut8	ENSMUSG00000021065	chr12	78340052	78577325	+	3388	1.531	1
Thrap3	ENSMUSG00000043962	chr4	125841326	125880004	-	9234	1.531	1
Fryl	ENSMUSG00000070733	chr5	73411561	73647857	-	14668	1.531	1
Lamp2	ENSMUSG00000016534	chrX	35754534	35809631	-	9552	1.53	1
Ralgapb	ENSMUSG00000027652	chr2	158235584	158324989	+	12992	1.529	1
Dbr1	ENSMUSG00000032469	chr9	99476218	99484882	+	2450	1.527	1
4632419I22Rik	ENSMUSG00000085208	chr11	86014872	86117945	+	6498	1.527	1
Rraga	ENSMUSG00000070934	chr4	86221577	86223185	+	1609	1.526	1
Maml2	ENSMUSG00000031925	chr9	13424434	13513832	+	5993	1.526	1
Zbtb7a	ENSMUSG00000035011	chr10	80597965	80615740	+	6808	1.526	1
Lrp5	ENSMUSG00000024913	chr19	3584828	3686564	-	9738	1.526	1
Otud6b	ENSMUSG00000040550	chr4	14736645	14753734	-	3387	1.526	1
Prpf3	ENSMUSG00000015748	chr3	95634047	95659808	-	5661	1.525	1
Arid3a	ENSMUSG00000019564	chr10	79389788	79417763	+	5562	1.525	1
Cx3cr1	ENSMUSG00000052336	chr9	119957811	119977400	-	3729	1.525	1
Krcc1	ENSMUSG00000053012	chr6	71221713	71235312	+	1870	1.524	1
Hnrnpul2	ENSMUSG00000071659	chr19	8893891	8908632	+	5726	1.524	1
Ninl	ENSMUSG00000068115	chr2	150760255	150865118	-	5958	1.523	1
Dlat	ENSMUSG00000000168	chr9	50442738	50467885	-	4886	1.523	1
Irak3	ENSMUSG00000020227	chr10	119578704	119639186	-	3676	1.523	1

Lama4	ENSMUSG00000019846	chr10	38685321	38829994	+	12068	1.523	1
Ctsc	ENSMUSG00000030560	chr7	95426595	95459398	+	5801	1.523	1
BC030867	ENSMUSG00000034773	chr11	102110196	102126501	+	3032	1.523	1
Diap1	ENSMUSG00000024456	chr18	38003255	38095130	-	6358	1.522	1
Mier1	ENSMUSG00000028522	chr4	102786995	102838359	+	12513	1.521	1
Zfp617	ENSMUSG00000066880	chr8	74432534	74458528	+	3024	1.521	1
Zfp46	ENSMUSG00000051351	chr4	135840598	135849857	+	5212	1.52	1
Fbxl20	ENSMUSG00000020883	chr11	97943868	98011717	-	12150	1.52	1
Crbn	ENSMUSG00000005362	chr6	106728243	106750068	-	5179	1.52	1
St3gal4	ENSMUSG00000032038	chr9	34854164	34924395	-	2029	1.52	1
Rnf213	ENSMUSG00000070327	chr11	119254414	119348732	+	16967	1.519	1
Lgals9	ENSMUSG00000001123	chr11	78776476	78798448	-	2137	1.519	1
Acer2	ENSMUSG00000038007	chr4	86520300	86580726	+	7159	1.519	1
Rcan2	ENSMUSG00000039601	chr17	43938800	44176465	+	3823	1.518	1
Lactb2	ENSMUSG00000025937	chr1	13614926	13650627	-	3051	1.518	1
Rab11fip2	ENSMUSG00000040022	chr19	59978787	60019557	-	6080	1.518	1
Snn	ENSMUSG00000037972	chr16	11066391	11075076	+	2902	1.518	1
Prkcb	ENSMUSG00000052889	chr7	129432265	129777916	+	4749	1.517	1
Snapin	ENSMUSG00000001018	chr3	90291948	90294955	-	2290	1.517	1
S100pbp	ENSMUSG00000040928	chr4	128825249	128866971	-	9448	1.516	1
Slc35e2	ENSMUSG00000042202	chr4	154975525	154997449	+	6574	1.516	1
Cldn12	ENSMUSG00000046798	chr5	5505109	5514958	-	4075	1.516	1
Ddi2	ENSMUSG00000078515	chr4	141233464	141279334	-	9553	1.515	1
Zfp187	ENSMUSG00000022228	chr13	21534044	21545599	-	4371	1.515	1
Dync1i2	ENSMUSG00000027012	chr2	71049763	71101360	+	8280	1.515	1
Ldlr	ENSMUSG00000032193	chr9	21528038	21554360	+	4513	1.515	1
Tfg	ENSMUSG00000022757	chr16	56690445	56717563	-	2292	1.515	1
Dnaja1	ENSMUSG00000028410	chr4	40669183	40684182	+	8629	1.514	1
Mphosph9	ENSMUSG00000038126	chr5	124700968	124777981	-	8544	1.514	1
Thoc7	ENSMUSG00000053453	chr14	14781528	14793739	-	883	1.513	1
Trim44	ENSMUSG00000027189	chr2	102140276	102247985	-	5645	1.513	1
Pcmtd2	ENSMUSG00000027589	chr2	181572559	181592166	+	4183	1.512	1
Dlg1	ENSMUSG00000022770	chr16	31663529	31873436	+	8957	1.511	1
Zfyve26	ENSMUSG00000066440	chr12	80333334	80397269	-	9368	1.511	1
Ttl	ENSMUSG00000027394	chr2	128891678	128922019	+	5945	1.51	1
Ston1	ENSMUSG00000033855	chr17	88997024	89061926	+	4521	1.51	1
4933403F05Rik	ENSMUSG00000038121	chr18	68419841	68459987	-	9813	1.509	1
Nfkbie	ENSMUSG00000023947	chr17	45692665	45700118	+	2422	1.509	1
Cfdp1	ENSMUSG00000031954	chr8	114292373	114378210	-	1215	1.508	1
Fam175b	ENSMUSG00000030965	chr7	140050908	140076794	+	5430	1.507	1

Gm15051	ENSMUSG00000087658	chr6	52106901	52112118	+	1025	1.507	1
Gipc2	ENSMUSG00000039131	chr3	151756497	151829194	-	3584	1.506	1
Inpp4a	ENSMUSG00000026113	chr1	37356710	37467581	+	7840	1.506	1
Zfand2a	ENSMUSG00000053581	chr5	139947165	139960503	-	3407	1.505	1
Ndst1	ENSMUSG00000054008	chr18	60845632	60908304	-	6597	1.504	1
Elf2	ENSMUSG00000037174	chr3	51059153	51144580	-	4388	1.504	1
Hp	ENSMUSG00000031722	chr8	112099030	112103072	-	1343	1.504	1
Nfkbid	ENSMUSG00000036931	chr7	31206751	31213765	+	2346	1.503	1
Gapdh	ENSMUSG00000057666	chr6	125111871	125116485	-	2253	1.503	1
Fbxo3	ENSMUSG00000027180	chr2	103867878	103903397	+	7237	1.502	1
Lrrc25	ENSMUSG00000049988	chr8	73140743	73144749	+	1273	1.502	1
Nxf1	ENSMUSG00000010097	chr19	8831593	8845399	+	2283	1.5	1
Zfyve20	ENSMUSG00000014550	chr6	92136706	92164919	-	5757	1.5	1
Trim23	ENSMUSG00000021712	chr13	104968877	104993452	+	5747	1.499	1
Pbrm1	ENSMUSG00000042323	chr14	31832324	31934778	+	8846	1.498	1
S100a10	ENSMUSG00000041959	chr3	93359002	93368565	+	1212	1.497	1
Gm15639	ENSMUSG00000086398	chr16	45745287	45745985	-	670	1.497	1
2310057M21Rik	ENSMUSG00000040177	chr7	138486232	138506212	-	2214	1.496	1
Mak16	ENSMUSG00000031578	chr8	32269940	32279196	-	1857	1.495	1
Ddx42	ENSMUSG00000020705	chr11	106078240	106110453	+	4228	1.494	1
Syt13	ENSMUSG00000027220	chr2	92755255	92796215	+	3761	1.493	1
Ralb	ENSMUSG00000004451	chr1	121366882	121401371	-	4734	1.491	1
Ripk1	ENSMUSG00000021408	chr13	34094232	34127039	+	6138	1.491	1
Mxd1	ENSMUSG00000001156	chr6	86597038	86619153	-	4662	1.49	1
mt-Cytb	ENSMUSG00000064370	chrM	14145	15288	+	1144	1.49	1
Asah2	ENSMUSG00000024887	chr19	32059144	32135959	-	4682	1.489	1
Umps	ENSMUSG00000022814	chr16	33954868	33967124	-	3484	1.489	1
Pja2	ENSMUSG00000024083	chr17	64630354	64681256	-	4751	1.489	1
Hsph1	ENSMUSG00000029657	chr5	150416862	150438951	-	7042	1.488	1
2810002D19Rik	ENSMUSG00000045464	chr2	94246308	94251839	+	2040	1.487	1
Mon2	ENSMUSG00000034602	chr10	122429117	122513561	-	9303	1.487	1
Rasgrp1	ENSMUSG00000027347	chr2	117105729	117168737	-	5598	1.485	1
Paxip1	ENSMUSG00000002221	chr5	28067207	28117879	-	5052	1.485	1
Ntan1	ENSMUSG00000022681	chr16	13819344	13835544	+	2174	1.484	1
Dusp11	ENSMUSG00000030002	chr6	85892262	85911661	-	7639	1.484	1
Mlxip	ENSMUSG00000038342	chr5	123844807	123915306	+	8213	1.482	1
Hcfc1	ENSMUSG00000031386	chrX	71188131	71211696	-	8360	1.481	1
Tpm1	ENSMUSG00000032366	chr9	66870397	66897213	-	6485	1.481	1
Gm10495	ENSMUSG00000073376	chr17	66668199	66668886	-	467	1.48	1

Pom121	ENSMUSG00000053293	chr5	135852011	135870416	-	5581	1.48	1
Ube2e3	ENSMUSG00000027011	chr2	78708281	78761450	+	4390	1.479	1
Slc20a2	ENSMUSG00000037656	chr8	23587172	23680079	+	3649	1.479	1
Ttc14	ENSMUSG00000027677	chr3	33698877	33713782	+	9776	1.478	1
Hus1	ENSMUSG00000020413	chr11	8893140	8911194	-	6450	1.476	1
B930095G15Rik	ENSMUSG00000085133	chr14	121647928	121650719	+	2792	1.475	1
5031414D18Rik	ENSMUSG00000034959	chr14	75415834	75452339	+	2651	1.475	1
Epm2a	ENSMUSG00000055493	chr10	11063202	11179442	+	3287	1.474	1
Grk5	ENSMUSG00000003228	chr19	60965652	61168456	+	3165	1.474	1
Klf13	ENSMUSG00000052040	chr7	71031237	71083801	-	6597	1.474	1
1110037F02Rik	ENSMUSG00000040720	chr4	11413105	11477831	+	7138	1.473	1
Npepps	ENSMUSG00000001441	chr11	97067156	97141952	-	7206	1.473	1
Nhsl1	ENSMUSG00000039835	chr10	18110128	18253698	+	11056	1.473	1
Herc2	ENSMUSG00000030451	chr7	63305525	63487164	+	15440	1.472	1
C78339	ENSMUSG00000069237	chr13	46764859	46773425	+	3859	1.472	1
Gpr4	ENSMUSG00000044317	chr7	19797887	19809525	+	2884	1.472	1
Ptafr	ENSMUSG00000056529	chr4	132119982	132138598	+	3637	1.471	1
Slc30a7	ENSMUSG00000054414	chr3	115641891	115710324	-	9020	1.471	1
Pde4d	ENSMUSG00000021699	chr13	109240142	110743669	+	12006	1.47	1
Metap1	ENSMUSG00000005813	chr3	138121924	138152346	-	2549	1.469	1
Phc1	ENSMUSG00000040669	chr6	122267749	122290579	-	5297	1.469	1
Mapre2	ENSMUSG00000024277	chr18	23910834	24052362	+	10202	1.468	1
Wnt4	ENSMUSG00000036856	chr4	136833404	136855641	+	4194	1.468	1
Amn1	ENSMUSG00000068250	chr6	149105669	149137234	-	4300	1.467	1
Zbtb39	ENSMUSG00000044617	chr10	127176594	127184391	+	5946	1.466	1
Ttyh2	ENSMUSG00000034714	chr11	114536745	114582291	+	3739	1.466	1
Zfp830	ENSMUSG00000046010	chr11	82577847	82581124	+	3278	1.466	1
Zswim4	ENSMUSG00000035671	chr8	86734577	86760954	-	4625	1.464	1
Dnajc2	ENSMUSG00000029014	chr5	21263085	21291069	-	5029	1.464	1
Tex10	ENSMUSG00000028345	chr4	48443730	48486331	-	4382	1.463	1
Adcy3	ENSMUSG00000020654	chr12	4133103	4213525	+	6246	1.463	1
Clec10a	ENSMUSG00000000318	chr11	69980125	69984336	+	1852	1.461	1
Cwc15	ENSMUSG00000004096	chr9	14305061	14315021	+	1111	1.461	1
Nktr	ENSMUSG00000032525	chr9	121628287	121665959	+	8743	1.46	1
Ctif	ENSMUSG00000052928	chr18	75590878	75857350	-	6173	1.46	1
Ehbp1	ENSMUSG00000042302	chr11	21905831	22242295	-	5709	1.46	1
Tspan6	ENSMUSG00000067377	chrX	130425607	130432968	-	2060	1.46	1
Tnfrsf14	ENSMUSG00000042333	chr4	154296319	154302672	-	1158	1.459	1
Dem1	ENSMUSG00000028629	chr4	120593801	120597622	-	2008	1.458	1

Rnf19b	ENSMUSG00000028793	chr4	128735515	128763130	+	2872	1.458	1
BC028528	ENSMUSG00000038543	chr3	95687877	95695928	-	1402	1.456	1
C1qtnf3	ENSMUSG00000058914	chr15	10882087	10909905	+	2557	1.456	1
C430048L16Rik	ENSMUSG00000052137	chr4	12016586	12023390	+	3468	1.456	1
Ctnnd1	ENSMUSG00000034101	chr2	84440228	84490922	-	7319	1.455	1
Arhgap31	ENSMUSG00000022799	chr16	38598453	38713387	-	11141	1.455	1
Snrnp48	ENSMUSG00000021431	chr13	38296808	38319532	+	1789	1.455	1
Cdc42bpb	ENSMUSG00000021279	chr12	112531183	112615929	-	6718	1.454	1
H2-DMb2	ENSMUSG00000037548	chr17	34280252	34288498	+	1710	1.453	1
Btaf1	ENSMUSG00000040565	chr19	37000569	37088547	+	8509	1.452	1
Tlk1	ENSMUSG00000041997	chr2	70550464	70663785	-	6490	1.451	1
Dhx40	ENSMUSG00000018425	chr11	86582348	86621198	-	4183	1.45	1
Ube2j1	ENSMUSG00000028277	chr4	33118391	33139338	+	3530	1.45	1
Ints6	ENSMUSG00000035161	chr14	63295167	63379949	-	4993	1.45	1
Git2	ENSMUSG00000041890	chr5	115177416	115225526	-	7227	1.448	1
Amot	ENSMUSG00000041688	chrX	141880968	141939724	-	8402	1.448	1
Ctsb	ENSMUSG00000021939	chr14	63741302	63764758	+	4738	1.447	1
Smad5	ENSMUSG00000021540	chr13	56804371	56843738	+	7420	1.447	1
Zmat3	ENSMUSG00000027663	chr3	32233718	32264600	-	8088	1.447	1
Atp6v1h	ENSMUSG00000033793	chr1	5073254	5152630	+	1976	1.447	1
Lcp1	ENSMUSG00000021998	chr14	75530908	75630649	+	9504	1.447	1
Herpud2	ENSMUSG00000008429	chr9	24912574	24956280	-	2744	1.446	1
Il6st	ENSMUSG00000021756	chr13	113265216	113297068	+	5207	1.446	1
Arcn1	ENSMUSG00000032096	chr9	44549647	44575928	-	6084	1.446	1
Chdh	ENSMUSG00000015970	chr14	30822209	30853652	+	6157	1.446	1
Bnip3l	ENSMUSG00000022051	chr14	67604076	67627714	-	3859	1.444	1
Mcm3ap	ENSMUSG00000001150	chr10	75931672	75978601	+	6426	1.444	1
Tubgcp4	ENSMUSG00000027263	chr2	120996390	121024506	+	8395	1.442	1
2900089D17Rik	ENSMUSG00000087484	chr5	145002559	145017055	-	2583	1.442	1
Slc8a1	ENSMUSG00000054640	chr17	81785359	82048947	-	3068	1.442	1
Cdca8	ENSMUSG00000028873	chr4	124595709	124616555	-	5177	1.441	1
Snap29	ENSMUSG00000022765	chr16	17406096	17430919	+	3429	1.441	1
Pan3	ENSMUSG00000029647	chr5	148241737	148360078	+	7316	1.441	1
1600029D21Rik	ENSMUSG00000032068	chr9	50302630	50313744	+	1986	1.44	1
Id2	ENSMUSG00000020644	chr12	25778666	25780957	-	1270	1.44	1
Tmx1	ENSMUSG00000021072	chr12	71554082	71569027	+	3299	1.439	1

Rnmt	ENSMUSG00000009535	chr18	68460009	68484506	+	7339	1.439	1
Akap13	ENSMUSG00000066406	chr7	82600420	82899495	+	15207	1.436	1
Degs1	ENSMUSG00000038633	chr1	184205903	184212935	-	2591	1.436	1
Zfp54	ENSMUSG00000023882	chr17	21560191	21572604	+	2832	1.435	1
Zkscan1	ENSMUSG00000029729	chr5	138526312	138549050	+	8541	1.435	1
Htt	ENSMUSG00000029104	chr5	35104389	35255183	+	18808	1.435	1
Tec	ENSMUSG00000029217	chr5	73146957	73259722	-	3698	1.434	1
Per2	ENSMUSG00000055866	chr1	93312561	93355907	-	5841	1.434	1
March5	ENSMUSG00000023307	chr19	37282033	37296629	+	2396	1.434	1
Cep70	ENSMUSG00000056267	chr9	99143887	99200820	+	2577	1.433	1
Mettl5	ENSMUSG00000051730	chr2	69709255	69723672	-	2437	1.433	1
Slc44a1	ENSMUSG00000028412	chr4	53453285	53635350	+	6953	1.433	1
Mark1	ENSMUSG00000026620	chr1	186720322	186823428	-	4546	1.432	1
Ep400	ENSMUSG00000029505	chr5	111093392	111199736	-	13971	1.432	1
Cmtm6	ENSMUSG00000032434	chr9	114640321	114658461	+	3352	1.431	1
Ctdp1	ENSMUSG00000033323	chr18	80604698	80666434	-	3936	1.431	1
Ppp2ca	ENSMUSG00000020349	chr11	51912183	51941280	+	7490	1.43	1
Zfp955a	ENSMUSG00000008686	chr17	33378464	33391985	-	1638	1.43	1
Fbxl4	ENSMUSG00000040410	chr4	22284690	22361238	+	4107	1.43	1
Arhgef3	ENSMUSG00000021895	chr14	28051225	28217090	+	3770	1.428	1
Tbc1d8b	ENSMUSG00000042473	chrX	136219535	136287944	+	5211	1.427	1
Daam1	ENSMUSG00000034574	chr12	72932065	73093354	+	6004	1.426	1
Rpia	ENSMUSG00000053604	chr6	70715714	70742226	-	5359	1.426	1
Srxn1	ENSMUSG00000032802	chr2	151931252	151937112	+	2820	1.426	1
Tbc1d24	ENSMUSG00000036473	chr17	24312376	24342507	-	8063	1.426	1
Rbm17	ENSMUSG00000037197	chr2	11507066	11525780	-	2363	1.425	1
Ppp3r1	ENSMUSG00000033953	chr11	17059266	17100378	+	3228	1.424	1
Tm9sf3	ENSMUSG00000025016	chr19	41285332	41338494	-	8105	1.424	1
Arid5a	ENSMUSG00000037447	chr1	36364578	36380874	+	7700	1.424	1
Fancm	ENSMUSG00000055884	chr12	66176593	66233045	+	7775	1.422	1
Nos3	ENSMUSG00000028978	chr5	23870628	23890292	+	4132	1.421	1
Hspb11	ENSMUSG00000063172	chr4	106926198	106952543	+	2071	1.42	1
Arl1	ENSMUSG00000060904	chr10	88193603	88206839	+	2369	1.418	1
Tbc1d14	ENSMUSG00000029192	chr5	36833253	36935925	-	9802	1.418	1
Map3k7	ENSMUSG00000028284	chr4	32051072	32110442	+	6423	1.418	1
Zfp935	ENSMUSG00000055228	chr13	62555683	62568128	-	1239	1.417	1
Hsf2	ENSMUSG00000019878	chr10	57206191	57232939	+	2667	1.417	1
Abcc4	ENSMUSG00000032849	chr14	118881914	119105441	-	5729	1.414	1
Ywhaq	ENSMUSG00000076432	chr12	21395932	21423498	-	3061	1.413	1
Klf11	ENSMUSG00000020653	chr12	25336236	25347639	+	4228	1.412	1
Eed	ENSMUSG00000030619	chr7	97103166	97129493	-	2218	1.411	1
Ltc4s	ENSMUSG00000020377	chr11	50049971	50052118	-	736	1.411	1
Cyb5r4	ENSMUSG00000032872	chr9	86916849	86972609	+	6781	1.41	1

Zfp58	ENSMUSG00000071291	chr13	67591103	67627087	-	2733	1.409	1
Napg	ENSMUSG00000024581	chr18	63137490	63159104	+	5139	1.409	1
Commd 10	ENSMUSG00000042705	chr18	47118515	47393938	+	3450	1.409	1
Siglece	ENSMUSG00000030474	chr7	50906440	50915531	-	1849	1.409	1
Thsd7a	ENSMUSG00000032625	chr6	12261610	12699410	-	13737	1.409	1
Anpep	ENSMUSG00000039062	chr7	86966689	86993096	-	3879	1.408	1
Fam193 a	ENSMUSG00000037210	chr5	34713062	34829104	+	5055	1.408	1
Cln3	ENSMUSG00000030720	chr7	133714721	133729331	-	5781	1.408	1
Cstf2t	ENSMUSG00000053536	chr19	31157331	31161080	+	3750	1.407	1
Trp53in p1	ENSMUSG00000028211	chr4	11083578	11101526	+	6475	1.407	1
Arxes2	ENSMUSG00000048040	chrX	132528359	132529898	+	1540	1.406	1
Ythdf2	ENSMUSG00000040025	chr4	131740827	131768218	-	4572	1.405	1
Usp7	ENSMUSG00000022710	chr16	8688815	8792401	-	8648	1.404	1
Rbm18	ENSMUSG00000026889	chr2	35971599	35992293	-	5740	1.402	1
Stra6	ENSMUSG00000032327	chr9	57911595	58001803	+	5829	1.402	1
BC0463 31	ENSMUSG00000039523	chr4	153349303	153382841	+	5279	1.401	1
Zfp322a	ENSMUSG00000046351	chr13	23444972	23461077	-	5197	1.401	1
Cxcr7	ENSMUSG00000044337	chr1	92100555	92113326	+	3348	1.399	1
Orai2	ENSMUSG00000039747	chr5	136623334	136646526	-	3875	1.398	1
Zfp36l1	ENSMUSG00000021127	chr12	81208747	81214000	-	2984	1.398	1
Mtor	ENSMUSG00000028991	chr4	147822720	147931792	+	10109	1.397	1
Mcts1	ENSMUSG00000000355	chrX	35953755	35966699	+	1667	1.395	1
Pspc1	ENSMUSG00000021938	chr14	57341278	57397153	-	4003	1.394	1
Gdpd3	ENSMUSG00000030703	chr7	133909928	133919157	+	1066	1.394	1
Egln1	ENSMUSG00000031987	chr8	127432496	127473154	-	3515	1.394	1
Rcsd1	ENSMUSG00000040723	chr1	167579076	167638225	-	2670	1.394	1
Grb2	ENSMUSG00000059923	chr11	115505359	115569911	-	2916	1.394	1
Plcg1	ENSMUSG00000016933	chr2	160557036	160601496	+	7857	1.393	1
2310003 L22Rik	ENSMUSG00000027384	chr2	139996382	140030988	+	3355	1.393	1
Mtf2	ENSMUSG00000029267	chr5	108494693	108538023	+	5263	1.393	1
2700078 E11Rik	ENSMUSG00000033417	chr19	60600599	60656926	-	5790	1.393	1
Csnk2a 1	ENSMUSG00000074698	chr2	152052575	152107588	+	7089	1.391	1
Ttf2	ENSMUSG00000033222	chr3	100742783	100773586	-	6564	1.389	1
Gatad1	ENSMUSG00000007415	chr5	3632932	3647934	-	4125	1.389	1
Cdc42s e2	ENSMUSG00000052298	chr11	54530958	54601177	-	3661	1.389	1
Rrm1	ENSMUSG00000030978	chr7	109590209	109618285	+	3997	1.388	1
Utp6	ENSMUSG00000035575	chr11	79745823	79775892	-	4517	1.387	1
Shank2	ENSMUSG00000037541	chr7	151187833	151610399	+	10681	1.387	1
Mef2c	ENSMUSG00000005583	chr13	83643033	83806684	+	7396	1.386	1

Rnf216	ENSMUSG00000045078	chr5	143752572	143874673	-	4645	1.386	1
Zfp84	ENSMUSG00000046185	chr7	30553571	30564840	+	4113	1.385	1
Ret	ENSMUSG00000030110	chr6	118101766	118147762	-	7345	1.385	1
D18Ertd653e	ENSMUSG00000024544	chr18	68092911	68415203	+	2644	1.385	1
Atrn	ENSMUSG00000027312	chr2	130732231	130856069	+	13208	1.385	1
Skiv2l2	ENSMUSG00000016018	chr13	113657988	113717588	-	3309	1.384	1
Cd24a	ENSMUSG00000047139	chr10	43298975	43304068	+	1813	1.384	1
Setdb1	ENSMUSG00000015697	chr3	95127447	95161124	-	6853	1.383	1
Megf9	ENSMUSG00000039270	chr4	70088099	70196029	-	8226	1.383	1
Ccdc75	ENSMUSG00000050668	chr17	79234856	79247639	+	4199	1.382	1
Sin3a	ENSMUSG00000042557	chr9	56919847	56976173	+	5400	1.382	1
Stx17	ENSMUSG00000061455	chr4	48137787	48199379	+	5935	1.381	1
Arl6	ENSMUSG00000022722	chr16	59612775	59639217	-	2098	1.381	1
Dcaf6	ENSMUSG00000026571	chr1	167259642	167390596	-	3297	1.379	1
Gm11110	ENSMUSG00000079414	chr17	57231458	57245365	-	1013	1.379	1
Stk35	ENSMUSG00000037885	chr2	129626253	129658023	+	6195	1.379	1
Dek	ENSMUSG00000021377	chr13	47180144	47201589	-	3133	1.378	1
Prpf38b	ENSMUSG00000027881	chr3	108705585	108714645	-	4750	1.378	1
Irf5	ENSMUSG00000029771	chr6	29476625	29491869	+	2903	1.378	1
Ybx1	ENSMUSG00000028639	chr4	118950586	118967209	-	2531	1.378	1
Tmem206	ENSMUSG00000026627	chr1	193149791	193174793	+	2246	1.377	1
Slc25a32	ENSMUSG00000022299	chr15	38925737	38944262	-	2945	1.377	1
Nup43	ENSMUSG00000040034	chr10	7387301	7398679	+	2560	1.375	1
Wdr48	ENSMUSG00000032512	chr9	119804004	119835703	+	3836	1.374	1
1190002H23Rik	ENSMUSG00000022018	chr14	79688563	79701452	-	918	1.374	1
Rnf4	ENSMUSG00000029110	chr5	34688360	34696083	+	2639	1.374	1
Rbm28	ENSMUSG00000029701	chr6	29073573	29115006	-	5666	1.372	1
Ghitm	ENSMUSG00000041028	chr14	37934004	37948452	-	2578	1.372	1
Selplg	ENSMUSG00000048163	chr5	114267807	114280510	-	2604	1.371	1
Snrpa1	ENSMUSG00000030512	chr7	73205185	73219468	+	3082	1.371	1
Fbxl14	ENSMUSG00000030019	chr6	119429686	119433904	+	4219	1.371	1
Dnajc5	ENSMUSG00000000826	chr2	181255190	181289838	+	6953	1.37	1
Mrps2	ENSMUSG00000035772	chr2	28323586	28326698	+	2919	1.37	1
Foxn3	ENSMUSG00000033713	chr12	100433190	100688321	-	4651	1.37	1
Epc1	ENSMUSG00000024240	chr18	6435949	6516106	-	4475	1.37	1
Il1r2	ENSMUSG00000026073	chr1	40141613	40182064	+	1337	1.369	1
Dcaf7	ENSMUSG00000049354	chr11	105898186	105920638	+	6133	1.369	1
Mpzl1	ENSMUSG00000026566	chr1	167522312	167564672	-	2356	1.369	1
Slc4a1ap	ENSMUSG00000029141	chr5	31829368	31856415	+	4759	1.369	1
Ate1	ENSMUSG00000030850	chr7	137535040	137663475	-	4875	1.369	1

Plekha5	ENSMUSG00000030231	chr6	140372620	140544804	+	5171	1.368		1
Sub1	ENSMUSG00000022205	chr15	11911094	11926738	-	3930	1.368		1
Mark3	ENSMUSG00000007411	chr12	112812721	112894438	+	3415	1.367		1
Timm9	ENSMUSG00000021079	chr12	72224160	72237662	-	1018	1.367		1
Mtpap	ENSMUSG00000024234	chr18	4375590	4397328	+	2651	1.367		1
2810422O20Rik	ENSMUSG00000041396	chr1	165925020	165927374	+	1513	1.366		1
Tmem194b	ENSMUSG00000043015	chr1	52687549	52708763	+	3564	1.366		1
BC013529	ENSMUSG00000040006	chr10	7487745	7512598	-	1884	1.366		1
Cct6a	ENSMUSG00000029447	chr5	130293315	130321696	+	1599	1.364		1
Plk1	ENSMUSG00000030867	chr7	129302951	129313389	+	2203	1.363		1
Slc35a3	ENSMUSG00000027957	chr3	116372387	116415749	-	10173	1.363		1
Pard6b	ENSMUSG00000044641	chr2	167906504	167926703	+	3399	1.362		1
Snrpe	ENSMUSG00000090553	chr1	135500448	135506868	-	1372	1.362		1
Rpp30	ENSMUSG00000024800	chr19	36158206	36179262	+	1155	1.362		1
Agl	ENSMUSG00000033400	chr3	116442917	116511084	-	12803	1.362		1
Epb4.1l1	ENSMUSG00000027624	chr2	156246645	156368950	+	11534	1.36		1
Cenpj	ENSMUSG00000064128	chr14	57145598	57190683	-	4363	1.36		1
Pik3cd	ENSMUSG00000039936	chr4	149023277	149076680	-	6259	1.359		1
Thoc2	ENSMUSG00000037475	chrX	39148168	39273851	-	12666	1.359		1
Cdc42	ENSMUSG00000006699	chr4	136875611	136913635	-	2966	1.359		1
Trak2	ENSMUSG00000026028	chr1	58957293	59030274	-	7088	1.358		1
Emp2	ENSMUSG00000022505	chr16	10281842	10314061	-	3440	1.358		1
Kdm6b	ENSMUSG00000018476	chr11	69212010	69227177	-	6954	1.357		1
Rab7	ENSMUSG00000079477	chr6	87949100	87995264	-	2610	1.357		1
Tardbp	ENSMUSG00000041459	chr4	147986491	148001128	-	7477	1.356		1
BC006779	ENSMUSG00000027580	chr2	180962320	180976732	-	11529	1.354		1
Gen1	ENSMUSG00000051235	chr12	11246547	11272593	-	4468	1.352		1
Arnt	ENSMUSG00000015522	chr3	95238310	95301162	+	8001	1.352		1
B230312A22Rik	ENSMUSG00000036002	chr4	43045286	43059092	-	4129	1.351		1
Als2	ENSMUSG00000026024	chr1	59219770	59294075	-	6868	1.351		1
C3	ENSMUSG00000024164	chr17	57343393	57367559	-	5143	1.351		1
Golga4	ENSMUSG00000038708	chr9	118415393	118491640	+	7643	1.351		1
Ube2v2	ENSMUSG00000022674	chr16	15552770	15594617	-	4275	1.351		1
Kdm1a	ENSMUSG00000036940	chr4	136106455	136158638	-	5179	1.35		1
Ngf	ENSMUSG00000027859	chr3	102273851	102324936	+	1187	1.35		1
Pptc7	ENSMUSG00000038582	chr5	122734374	122774290	+	4719	1.349		1
Golt1b	ENSMUSG00000030245	chr6	142335741	142352378	+	2818	1.349		1
Kif22	ENSMUSG00000030677	chr7	134171246	134185934	-	2063	1.349		1

D4Wsu53e	ENSMUSG00000037266	chr4	134479507	134483586	+	3349	1.349		1
Mdn1	ENSMUSG00000058006	chr4	32744094	32862182	+	21826	1.347		1
Fam65a	ENSMUSG00000038604	chr8	108129129	108146094	+	4109	1.346		1
Nrbf2	ENSMUSG00000075000	chr10	66729437	66748053	-	1860	1.346		1
Trim13	ENSMUSG00000035235	chr14	62217063	62224783	+	1699	1.346		1
Tceal8	ENSMUSG00000051579	chrX	132703523	132706881	-	2563	1.345		1
BC003331	ENSMUSG00000006010	chr1	152208435	152240210	-	4084	1.345		1
Ecscr	ENSMUSG00000073599	chr18	35872740	35882010	-	1680	1.345		1
Hspa8	ENSMUSG00000015656	chr9	40609067	40613283	+	4156	1.345		1
Rtf1	ENSMUSG00000027304	chr2	119500804	119561143	+	5731	1.344		1
Dna2	ENSMUSG00000036875	chr10	62409774	62436933	+	5883	1.344		1
Rnf103	ENSMUSG00000052656	chr6	71443888	71475014	+	4348	1.344		1
Fam36a	ENSMUSG00000026500	chr1	180249261	180252824	+	883	1.343		1
BC026590	ENSMUSG00000038827	chr4	56815217	56822473	+	5574	1.343		1
Rhebl1	ENSMUSG00000023755	chr15	98708209	98711835	-	1940	1.342		1
Vamp3	ENSMUSG00000028955	chr4	150421409	150432072	-	2889	1.342		1
Aggf1	ENSMUSG00000021681	chr13	96120638	96145307	-	3697	1.341		1
Wnt9a	ENSMUSG00000000126	chr11	59120430	59147054	+	3329	1.34		1
Trdmt1	ENSMUSG00000026723	chr2	13430641	13466291	-	3831	1.34		1
Numb	ENSMUSG00000021224	chr12	85134984	85262884	-	3823	1.34		1
Litaf	ENSMUSG00000022500	chr16	10959368	11066250	-	2642	1.339		1
Ppat	ENSMUSG00000029246	chr5	77342274	77380603	-	4421	1.338		1
Crebzf	ENSMUSG00000051451	chr7	97591239	97596227	+	4989	1.338		1
Ric8b	ENSMUSG00000035620	chr10	84380361	84479191	+	3068	1.338		1
Azi1	ENSMUSG00000039781	chr11	119925744	119948141	-	3833	1.338		1
Lzic	ENSMUSG00000028990	chr4	148859338	148870777	+	1866	1.338		1
Igfbp4	ENSMUSG00000017493	chr11	98902558	98915706	+	4416	1.338		1
Cables2	ENSMUSG00000038990	chr2	179993245	180008201	-	4597	1.336		1
Ttc35	ENSMUSG00000022337	chr15	43308775	43359323	+	1245	1.336		1
Serpina3g	ENSMUSG00000041481	chr12	105474455	105480149	+	2306	1.336		1
Fam69a	ENSMUSG00000029270	chr5	108337072	108416104	-	2892	1.335		1
Arhgef6	ENSMUSG00000031133	chrX	54484662	54591906	-	8552	1.334		1
AW112010	ENSMUSG00000075010	chr19	11122103	11125056	-	774	1.334		1
Zfp369	ENSMUSG00000021514	chr13	65380122	65405529	+	5896	1.334		1
Orc5	ENSMUSG00000029012	chr5	21992303	22056247	-	2825	1.334		1
D030016E14Rik	ENSMUSG00000038102	chr8	48575469	48618824	-	6482	1.333		1
Olfm1	ENSMUSG00000026833	chr2	28048512	28086256	+	3553	1.333		1
Tsc1	ENSMUSG00000026812	chr2	28496748	28546687	+	11833	1.332		1

Rab3gap2	ENSMUSG00000039318	chr1	187028047	187110622	+	6985	1.331	1
Pitpna	ENSMUSG00000017781	chr11	75401599	75442306	+	4109	1.329	1
Marcks	ENSMUSG00000069662	chr10	36853049	36858732	-	4186	1.328	1
Zfp952	ENSMUSG00000053390	chr17	33130074	33142402	+	4368	1.328	1
Snx7	ENSMUSG00000028007	chr3	117484415	117571854	-	2171	1.327	1
Nono	ENSMUSG00000031311	chrX	98624657	98643930	+	4375	1.327	1
Nfatc3	ENSMUSG00000031902	chr8	108582740	108654437	+	6197	1.323	1
Zcchc7	ENSMUSG00000035649	chr4	44768749	44945087	+	9612	1.323	1
1700054N08Rik	ENSMUSG00000031971	chr8	126364731	126384109	-	2767	1.323	1
Zmynd8	ENSMUSG00000039671	chr2	165609654	165724516	-	11041	1.323	1
Msl3l2	ENSMUSG00000047669	chr10	55826723	55836686	+	2140	1.322	1
Smtnl2	ENSMUSG00000045667	chr11	72202666	72225215	-	3815	1.322	1
Slc10a6	ENSMUSG00000029321	chr5	104034730	104058422	-	2124	1.321	1
Baz1b	ENSMUSG00000002748	chr5	135663134	135721999	+	10794	1.32	1
Ssbp1	ENSMUSG00000029911	chr6	40421351	40431822	+	3319	1.32	1
Nup188	ENSMUSG00000052533	chr2	30141917	30199786	+	6241	1.318	1
Srsf5	ENSMUSG00000021134	chr12	82046491	82051494	+	3988	1.317	1
Ubl3	ENSMUSG00000001687	chr5	149316207	149364365	-	2668	1.317	1
Phf12	ENSMUSG00000037791	chr11	77796256	77844041	+	5094	1.316	1
Traf3ip1	ENSMUSG00000034292	chr1	93391245	93425880	+	3496	1.316	1
Arl6ip1	ENSMUSG00000030654	chr7	125262405	125273139	-	2134	1.314	1
Pkp1	ENSMUSG00000026413	chr1	137767972	137815784	-	5024	1.314	1
Tubb3	ENSMUSG00000062380	chr8	125935324	125945915	+	1868	1.313	1
Snx29	ENSMUSG00000079738	chr16	11322985	11403929	+	1205	1.312	1
Cav2	ENSMUSG00000000058	chr6	17231185	17239115	+	3335	1.311	1
Ppp2r3c	ENSMUSG00000021022	chr12	56381801	56403987	-	2395	1.311	1
Grpel2	ENSMUSG00000024580	chr18	61872094	61885985	-	4030	1.31	1
Ankfy1	ENSMUSG00000020790	chr11	72503508	72585648	+	11332	1.309	1
Cd302	ENSMUSG00000060703	chr2	60090050	60122545	-	1354	1.309	1
Rab1	ENSMUSG00000020149	chr11	20101435	20126859	+	3813	1.307	1
Rgs12	ENSMUSG00000029101	chr5	35292094	35382293	+	7910	1.306	1
Spen	ENSMUSG00000040761	chr4	141023805	141094512	-	14089	1.305	1
Aak1	ENSMUSG00000057230	chr6	86799511	86941858	+	8272	1.305	1
Kank2	ENSMUSG00000032194	chr9	21571217	21602990	-	4853	1.304	1
Acot7	ENSMUSG00000028937	chr4	151552243	151645964	+	2124	1.304	1
Ahcyl1	ENSMUSG00000027893	chr3	107466036	107499478	-	6690	1.303	1
Nceh1	ENSMUSG00000027698	chr3	27081887	27183530	+	5253	1.303	1
E2f5	ENSMUSG00000027552	chr3	14578671	14606308	+	1748	1.303	1
Isg20l2	ENSMUSG00000048039	chr3	87734236	87744608	+	2893	1.303	1
Rpl5	ENSMUSG00000058558	chr5	108329521	108338024	+	3920	1.302	1
Tle4	ENSMUSG00000024642	chr19	14522562	14672473	-	4630	1.301	1
Lrrc8a	ENSMUSG00000007476	chr2	30093235	30137668	+	5693	1.301	1
Pgk1	ENSMUSG00000062070	chrX	103382439	103399038	+	2244	1.3	1

Efr3a	ENSMUSG00000015002	chr15	65618596	65705378	+	11101	1.3	1
Dapk1	ENSMUSG00000021559	chr13	60703308	60864546	+	5934	1.299	1
Scara5	ENSMUSG00000022032	chr14	66285240	66383663	+	6093	1.299	1
Slc11a1	ENSMUSG00000026177	chr1	74421777	74432636	+	3697	1.298	1
Smap1	ENSMUSG00000026155	chr1	23851685	23929156	-	3575	1.298	1
Stard4	ENSMUSG00000024378	chr18	33359009	33373516	-	5586	1.298	1
Ntng2	ENSMUSG00000035513	chr2	29050061	29108525	-	3971	1.297	1
Fgfr1op	ENSMUSG00000069135	chr17	8358366	8389669	+	4008	1.296	1
Etfdh	ENSMUSG00000027809	chr3	79407710	79432785	-	6771	1.296	1
Tbc1d2b	ENSMUSG00000037410	chr9	90096885	90165642	-	8781	1.295	1
Ccdc58	ENSMUSG00000075229	chr16	36071746	36092204	+	1225	1.295	1
Rpl22l1	ENSMUSG00000039221	chr3	28704358	28706274	+	489	1.294	1
Rev1	ENSMUSG00000026082	chr1	38109632	38186507	-	4261	1.293	1
Uimc1	ENSMUSG00000025878	chr13	55129241	55201661	-	5085	1.292	1
Itpr1	ENSMUSG00000030102	chr6	108163112	108501103	+	10434	1.291	1
Batf	ENSMUSG00000034266	chr12	87027619	87050037	+	955	1.291	1
Atxn7l2	ENSMUSG00000048997	chr3	108005140	108013852	-	4848	1.291	1
Rsad2	ENSMUSG00000020641	chr12	27127618	27141317	-	4328	1.291	1
Arhgef1 5	ENSMUSG00000052921	chr11	68756657	68770982	-	6482	1.291	1
Trim26	ENSMUSG00000024457	chr17	36974079	36996343	+	6850	1.289	1
Cdk2	ENSMUSG00000025358	chr10	128134995	128142107	-	2432	1.289	1
Pacrg	ENSMUSG00000037196	chr17	10595878	11033177	-	1572	1.288	1
Trmt6	ENSMUSG00000037376	chr2	132629943	132641791	-	5384	1.287	1
Cog3	ENSMUSG00000034893	chr14	76102158	76154300	-	4222	1.284	1
RP23-102L5.1	ENSMUSG00000093677	chr15	58612016	58613506	-	1491	1.284	1
Ptpn7	ENSMUSG00000031506	chr1	137029302	137041897	+	3384	1.283	1
Kdm4a	ENSMUSG00000033326	chr4	117809562	117852648	-	5782	1.283	1
Kcnj8	ENSMUSG00000030247	chr6	142513459	142519876	-	2188	1.283	1
Bptf	ENSMUSG00000040481	chr11	106894395	106993441	-	13907	1.283	1
Gabpb2	ENSMUSG00000038766	chr3	94985688	95021864	-	15538	1.282	1
Nt5c3	ENSMUSG00000029780	chr6	56832394	56873926	-	4250	1.282	1
Mtrr	ENSMUSG00000034617	chr13	68699657	68720998	-	4424	1.281	1
Rmnd5a	ENSMUSG00000002222	chr6	71338628	71390631	-	6783	1.279	1
Utp14a	ENSMUSG00000063785	chrX	45610039	45635630	+	4130	1.279	1
Palld	ENSMUSG00000058056	chr8	63992043	64381487	-	8479	1.277	1
Oma1	ENSMUSG00000035069	chr4	102986417	103044473	+	2144	1.277	1
Gltscr1	ENSMUSG00000070808	chr7	16556611	16584844	-	5360	1.277	1
Dync1h1	ENSMUSG00000018707	chr12	111839662	111905155	+	15658	1.277	1
Spast	ENSMUSG00000024068	chr17	74738327	74790453	+	4696	1.275	1
Nnt	ENSMUSG00000025453	chr13	120124255	120197804	-	7845	1.275	1
Sorcs2	ENSMUSG00000029093	chr5	36359829	36740788	-	8803	1.275	1
Foxk1	ENSMUSG00000056493	chr5	142877451	142937968	+	7445	1.275	1

Ubr4	ENSMUSG00000066036	chr4	138908524	139045503	+	17997	1.274	1
Ebag9	ENSMUSG00000022339	chr15	44451187	44472570	+	1561	1.274	1
Cluap1	ENSMUSG00000014232	chr16	3908801	3941147	+	2279	1.274	1
Mtif2	ENSMUSG00000020459	chr11	29426408	29445279	+	3868	1.274	1
Ewsr1	ENSMUSG00000009079	chr11	4969692	4999269	-	5907	1.273	1
Arid3b	ENSMUSG00000004661	chr9	57638160	57684600	-	7976	1.273	1
5430417L22Rik	ENSMUSG00000074918	chr2	118571494	118574546	+	3053	1.273	1
Gnpnat1	ENSMUSG00000037722	chr14	45996009	46008477	-	2629	1.273	1
Mtap4	ENSMUSG00000032479	chr9	109833962	109986451	+	12938	1.271	1
Pdcd10	ENSMUSG00000027835	chr3	75320412	75360778	-	2939	1.271	1
D10Wsu102e	ENSMUSG00000020255	chr10	82822966	82831580	+	3509	1.27	1
Sec16b	ENSMUSG00000026589	chr1	159436859	159498556	+	5410	1.269	1
Tmem19	ENSMUSG00000069520	chr10	114777795	114799318	-	3010	1.269	1
Bend3	ENSMUSG00000038214	chr10	43198946	43235202	+	6028	1.268	1
Sh3bp2	ENSMUSG00000054520	chr5	34868487	34906274	+	4264	1.268	1
Ppig	ENSMUSG00000042133	chr2	69560602	69592069	+	8122	1.267	1
2610001J05Rik	ENSMUSG00000052419	chr6	13817735	13821518	-	3194	1.267	1
Pias1	ENSMUSG00000032405	chr9	62727884	62828690	-	4698	1.265	1
Mycbp2	ENSMUSG00000033004	chr14	103512628	103746031	-	19251	1.262	1
Kcnj2	ENSMUSG00000041695	chr11	110927478	110938135	+	5444	1.261	1
B3galnt1	ENSMUSG00000043300	chr3	69378108	69402783	-	1901	1.261	1
Ranbp1	ENSMUSG00000005732	chr16	18239877	18248825	-	1666	1.261	1
Slc22a4	ENSMUSG00000020334	chr11	53796625	53841592	-	2553	1.261	1
4930518I15Rik	ENSMUSG00000074629	chr2	156682352	156683682	-	1331	1.26	1
Tmed8	ENSMUSG00000034111	chr12	88507192	88541179	-	7179	1.26	1
Nudt4	ENSMUSG00000020029	chr10	95009641	95026801	-	3231	1.26	1
Slc31a1	ENSMUSG00000066150	chr4	62021761	62052803	+	4280	1.259	1
Arpc1b	ENSMUSG00000029622	chr5	145875084	145891574	+	5103	1.259	1
Bivm	ENSMUSG00000041684	chr1	44175802	44201615	+	3984	1.258	1
Eln	ENSMUSG00000029675	chr5	135179651	135223111	-	2583	1.257	1
Pldn	ENSMUSG00000005804	chr2	122564239	122575211	+	3899	1.256	1
Zfp512	ENSMUSG00000062761	chr5	31754809	31784122	+	3293	1.254	1
Fam76a	ENSMUSG00000028878	chr4	132455128	132478473	-	3088	1.253	1
Slc16a13	ENSMUSG00000044367	chr11	70030291	70034496	-	2531	1.253	1
Terf1	ENSMUSG00000025925	chr1	15795739	15833551	+	1674	1.252	1
Ppp2r5a	ENSMUSG00000026626	chr1	193175860	193220920	-	3082	1.252	1
Ddx26b	ENSMUSG00000035967	chrX	53708034	53761020	+	7173	1.25	1

Grsf1	ENSMUSG00000044221	chr5	89088473	89105196	-	3036	1.25	1
Dab2ip	ENSMUSG00000026883	chr2	35413786	35586514	+	8366	1.25	1
Lyrm5	ENSMUSG00000040370	chr6	145159654	145165459	+	2682	1.249	1
Rad23b	ENSMUSG00000028426	chr4	55362915	55405109	+	5733	1.249	1
Ammecr1l	ENSMUSG00000041915	chr18	31919534	31942381	+	3481	1.249	1
Tdp1	ENSMUSG00000021177	chr12	101122727	101193429	+	5464	1.248	1
Dnmbp	ENSMUSG00000025195	chr19	43922457	43986882	-	4900	1.248	1
Smg7	ENSMUSG00000042772	chr1	154684126	154749776	-	6097	1.248	1
Fbxw17	ENSMUSG00000037816	chr13	50513233	50529149	+	3818	1.248	1
Cd300lg	ENSMUSG00000017309	chr11	101902823	101916934	+	2737	1.248	1
Eif3e	ENSMUSG00000022336	chr15	43081586	43114282	-	1541	1.246	1
mt-Nd1	ENSMUSG00000064341	chrM	2751	3707	+	957	1.246	1
C330018D20Rik	ENSMUSG00000024592	chr18	57115487	57135022	-	3233	1.244	1
Btg1	ENSMUSG00000036478	chr10	96079635	96085447	+	5010	1.244	1
Atxn2l	ENSMUSG00000032637	chr7	133635224	133646816	-	4431	1.243	1
Nsg1	ENSMUSG00000029126	chr5	38528432	38550706	-	2140	1.242	1
Ube2k	ENSMUSG00000029203	chr5	65928472	65990227	+	5173	1.242	1
AW554918	ENSMUSG00000033632	chr18	25327500	25625822	+	7014	1.241	1
Trim24	ENSMUSG00000029833	chr6	37820811	37916296	+	8537	1.241	1
Arl8b	ENSMUSG00000030105	chr6	108733053	108773717	+	2928	1.241	1
Tiprl	ENSMUSG00000040843	chr1	167145053	167167074	-	1886	1.24	1
Thumpd3	ENSMUSG00000030264	chr6	112996219	113018267	+	3888	1.24	1
Ube2i	ENSMUSG00000015120	chr17	25398861	25411567	-	5474	1.239	1
Hps5	ENSMUSG00000014418	chr7	54015836	54051434	-	6482	1.239	1
Mff	ENSMUSG00000026150	chr1	82721465	82748969	+	4091	1.238	1
Gm16793	ENSMUSG00000087118	chr8	36645626	36652026	-	1611	1.238	1
Nemf	ENSMUSG00000020982	chr12	70412531	70458153	-	3711	1.237	1
Gdi1	ENSMUSG00000015291	chrX	71550337	71557201	+	4296	1.236	1
Bfsp1	ENSMUSG00000027420	chr2	143652264	143688909	-	2390	1.235	1
Fam115a	ENSMUSG00000036667	chr6	42618001	42660071	-	9622	1.234	1
Chst14	ENSMUSG00000074916	chr2	118752233	118754321	+	2089	1.233	1
Bbs9	ENSMUSG00000035919	chr9	22280159	22692724	+	6243	1.232	1
Kcne4	ENSMUSG00000047330	chr1	78813502	78816599	+	2484	1.232	1
Lmbr1	ENSMUSG00000010721	chr5	29556354	29704930	-	4953	1.231	1
Oxsm	ENSMUSG00000021786	chr14	17071170	17082322	-	4082	1.231	1
Sema6b	ENSMUSG00000001227	chr17	56262508	56279766	-	3977	1.231	1
Rars	ENSMUSG00000018848	chr11	35621883	35648008	-	3228	1.229	1
Celf1	ENSMUSG00000005506	chr2	90780539	90859654	+	8446	1.229	1
Tmem167	ENSMUSG00000012422	chr13	90228728	90283512	+	10807	1.228	1

Pabpc1	ENSMUSG00000022283	chr15	36525416	36538728	-	5768	1.228	1
Atg3	ENSMUSG00000022663	chr16	45158898	45188651	+	3154	1.227	1
Prr9	ENSMUSG00000056270	chr3	91926126	91927869	-	1183	1.226	1
4833420G17Rik	ENSMUSG00000062822	chr13	120251566	120274924	+	2574	1.226	1
Gmcl1	ENSMUSG00000001157	chr6	86641762	86683372	-	3823	1.226	1
Sf3a3	ENSMUSG00000028902	chr4	124392020	124409704	+	2785	1.224	1
Bcor	ENSMUSG00000040363	chrX	11613866	11737481	-	7190	1.224	1
Vwf	ENSMUSG00000001930	chr6	125496792	125636697	+	16064	1.223	1
Golga1	ENSMUSG00000026754	chr2	38871781	38921061	-	5392	1.221	1
Mitf	ENSMUSG00000035158	chr6	97757052	97971352	+	6233	1.221	1
Spop	ENSMUSG00000057522	chr11	95275394	95354720	+	3159	1.22	1
Ankrd49	ENSMUSG00000031931	chr9	14584653	14587408	-	1753	1.219	1
Igkv14-126	ENSMUSG00000076509	chr6	67846171	67846636	+	353	1.219	1
Pde8a	ENSMUSG00000025584	chr7	88358482	88479419	+	4045	1.219	1
Zc3h13	ENSMUSG00000022000	chr14	75684180	75744233	+	6163	1.219	1
Mrpl42	ENSMUSG00000062981	chr10	94943440	94964561	-	666	1.219	1
Tmem181b-ps	ENSMUSG00000079733	chr17	6439626	6489526	-	2585	1.218	1
Nkd2	ENSMUSG00000021567	chr13	73955982	73985079	-	4728	1.218	1
Tln1	ENSMUSG00000028465	chr4	43544391	43575563	-	9801	1.217	1
Etf1	ENSMUSG00000024360	chr18	35062446	35091657	-	3698	1.217	1
Upf2	ENSMUSG00000043241	chr2	5872515	5977749	+	5474	1.215	1
Zfp157	ENSMUSG00000036898	chr5	138882696	138901922	+	8782	1.215	1
Dennd1c	ENSMUSG00000002668	chr17	57205479	57217933	-	2520	1.215	1
Scarf1	ENSMUSG00000038188	chr11	75327042	75340084	+	2887	1.215	1
Ttc19	ENSMUSG00000042298	chr11	62094975	62141953	+	4675	1.214	1
Ncor1	ENSMUSG00000018501	chr11	62129928	62272043	-	12820	1.213	1
Ube3a	ENSMUSG00000025326	chr7	66484122	66562097	+	5097	1.212	1
Txlng	ENSMUSG00000038344	chrX	159216851	159267386	-	7541	1.211	1
Pon3	ENSMUSG00000029759	chr6	5170852	5206286	-	3385	1.211	1
Nlk	ENSMUSG00000017376	chr11	78380670	78510927	-	5499	1.211	1
Irf2	ENSMUSG00000031627	chr8	47825099	47932812	+	2484	1.21	1
Mettl6	ENSMUSG00000021891	chr14	32291506	32308216	-	2436	1.208	1
S100a6	ENSMUSG00000001025	chr3	90416816	90418336	+	719	1.207	1
Atp6v1c1	ENSMUSG00000022295	chr15	38591659	38622198	+	2097	1.206	1
Praf2	ENSMUSG00000031149	chrX	7305565	7308190	+	1339	1.206	1
Rsl1	ENSMUSG00000058900	chr13	67274143	67284062	+	1648	1.204	1
Sms	ENSMUSG00000071708	chrX	153881787	153930219	-	6010	1.202	1
Tbc1d8	ENSMUSG00000003134	chr1	39428344	39535592	-	4436	1.202	1
Mmrn2	ENSMUSG00000041445	chr14	35188690	35217473	+	3935	1.201	1
Rab2a	ENSMUSG00000047187	chr4	8462791	8534925	+	2133	1.201	1

Trappc6b	ENSMUSG00000020993	chr12	60144080	60162448	-	1254	1.2	1
Gcap14	ENSMUSG00000058690	chr14	37688122	37781950	-	7552	1.2	1
Rab23	ENSMUSG00000004768	chr1	33776727	33799409	+	8273	1.2	1
Pip5k1c	ENSMUSG00000034902	chr10	80755708	80782718	+	5730	1.199	1
Nkx2-3	ENSMUSG00000044220	chr19	43686815	43690381	+	2111	1.199	1
Lgr4	ENSMUSG00000050199	chr2	109757804	109854414	+	5884	1.199	1
Rtn3	ENSMUSG00000024758	chr19	7500391	7557779	-	5030	1.199	1
Usp37	ENSMUSG00000033364	chr1	74482084	74590860	-	7196	1.198	1
Trip4	ENSMUSG00000032386	chr9	65678943	65756601	-	6188	1.197	1
Dgcr8	ENSMUSG00000022718	chr16	18254041	18289342	-	7286	1.196	1
Serpinf1	ENSMUSG00000000753	chr11	75223271	75236203	-	2359	1.195	1
1110050K14Rik	ENSMUSG00000087156	chr8	123578782	123606932	-	2968	1.194	1
Pak1	ENSMUSG00000030774	chr7	104937051	105060891	+	3762	1.193	1
Hp1bp3	ENSMUSG00000028759	chr4	137772211	137800598	+	8433	1.192	1
Frmd4b	ENSMUSG00000030064	chr6	97236861	97567535	-	11521	1.192	1
Clip1	ENSMUSG00000049550	chr5	124027804	124134627	-	9801	1.191	1
Mdc1	ENSMUSG00000061607	chr17	35978460	35996615	+	7421	1.191	1
Rnf169	ENSMUSG00000058761	chr7	107068764	107128968	-	7157	1.19	1
Olfml2a	ENSMUSG00000046618	chr2	38787498	38819273	+	5579	1.189	1
Klhl5	ENSMUSG00000054920	chr5	65498807	65559427	+	4866	1.189	1
Snrk	ENSMUSG00000038145	chr9	122026401	122078820	+	5763	1.188	1
Chd6	ENSMUSG00000057133	chr2	160772714	160934811	-	19192	1.188	1
Wdr7	ENSMUSG00000040560	chr18	63868349	64149414	+	7109	1.187	1
Zfp12	ENSMUSG00000029587	chr5	143996842	144010513	+	5821	1.187	1
Cfp	ENSMUSG00000001128	chrX	20502580	20508681	-	1736	1.186	1
Shroom2	ENSMUSG00000045180	chrX	149044052	149204008	-	8375	1.186	1
Ccdc59	ENSMUSG00000019897	chr10	105278123	105284889	+	4314	1.185	1
A930001C03Rik	ENSMUSG00000087132	chr19	4439003	4448332	+	2008	1.183	1
Papd5	ENSMUSG00000036779	chr8	90723112	90783621	+	4937	1.182	1
Akap9	ENSMUSG00000040407	chr5	3928054	4080209	+	15863	1.182	1
Kif16b	ENSMUSG00000038844	chr2	142443210	142727267	-	6635	1.182	1
Elf1	ENSMUSG00000036461	chr14	79881001	79982283	+	4328	1.182	1
Mettl9	ENSMUSG00000030876	chr7	128177959	128220348	+	1822	1.182	1
Rasgef1b	ENSMUSG00000029333	chr5	99646445	99681946	-	3261	1.181	1
Aifm2	ENSMUSG00000020085	chr10	61178011	61202008	+	4121	1.181	1
Ddx20	ENSMUSG00000027905	chr3	105481178	105490492	-	4152	1.181	1
Tm9sf2	ENSMUSG00000025544	chr14	122506260	122558826	+	3107	1.181	1
Pank2	ENSMUSG00000037514	chr2	131088231	131124924	+	4936	1.18	1
Fbxo22	ENSMUSG00000032309	chr9	55056732	55072240	+	2394	1.176	1
Cd83	ENSMUSG00000015396	chr13	43880476	43898499	+	2238	1.176	1

Uchl3	ENSMUSG00000022111	chr14	102053184	102095341	+	1672	1.176	1
Atg2b	ENSMUSG00000041341	chr12	106851749	106923451	-	10317	1.175	1
Ankhd1	ENSMUSG00000024483	chr18	36719641	36825571	+	9853	1.174	1
Sipa1l3	ENSMUSG00000030583	chr7	30105397	30290479	-	7809	1.174	1
Parva	ENSMUSG00000030770	chr7	119571019	119735206	+	10157	1.174	1
1110059 E24Rik	ENSMUSG00000035171	chr19	21671803	21727281	-	1428	1.173	1
Snx20	ENSMUSG00000031662	chr8	91150727	91160027	-	1434	1.173	1
Fgfbp1	ENSMUSG00000048373	chr5	44370104	44373001	-	1154	1.173	1
Rbp4	ENSMUSG00000024990	chr19	38191110	38199811	-	1297	1.173	1
Zdhhc20	ENSMUSG00000021969	chr14	58451539	58509099	-	5209	1.172	1
Gtf3c4	ENSMUSG00000035666	chr2	28677819	28695880	-	7230	1.172	1
Trove2	ENSMUSG00000018199	chr1	145597920	145624198	-	9541	1.17	1
Zfp318	ENSMUSG00000015597	chr17	46520680	46557869	+	8597	1.169	1
Btg2	ENSMUSG00000020423	chr1	135971747	135975697	-	2743	1.169	1
Acpl2	ENSMUSG00000043587	chr9	96723763	96793088	-	4412	1.168	1
Ddx58	ENSMUSG00000040296	chr4	40150806	40186861	-	7050	1.168	1
Ctr9	ENSMUSG00000005609	chr7	118172465	118199891	+	6545	1.167	1
Cdk19	ENSMUSG00000038481	chr10	40069114	40203624	+	5807	1.167	1
Setd7	ENSMUSG00000037111	chr3	51319241	51364800	-	7790	1.167	1
S100a4	ENSMUSG00000001020	chr3	90407692	90409967	+	587	1.166	1
Zfp944	ENSMUSG00000033972	chr17	22474956	22498367	-	2998	1.165	1
Sfrp2	ENSMUSG00000027996	chr3	83570243	83578238	+	2153	1.163	1
Msi2	ENSMUSG00000069769	chr11	88152884	88579827	-	10663	1.163	1
Zranb2	ENSMUSG00000028180	chr3	157197124	157211354	+	4301	1.163	1
Rptor	ENSMUSG00000025583	chr11	119464219	119760890	+	13471	1.163	1
Whsc1l1	ENSMUSG00000054823	chr8	26712073	26830139	+	16513	1.162	1
Kdm2a	ENSMUSG00000054611	chr19	4316144	4398285	-	12569	1.161	1
2610002 M06Rik	ENSMUSG00000031242	chrX	104978090	105011673	-	5842	1.161	1
Crtap	ENSMUSG00000032431	chr9	114284252	114299793	-	1772	1.161	1
Hmbox1	ENSMUSG00000021972	chr14	65430438	65568707	-	9703	1.16	1
Setbp1	ENSMUSG00000024548	chr18	78947119	79306130	-	9953	1.16	1
Phospho2	ENSMUSG00000027088	chr2	69627680	69638062	+	2970	1.16	1
Lgals1	ENSMUSG00000068220	chr15	78757155	78760895	+	800	1.158	1
Rcbtb1	ENSMUSG00000035469	chr14	59820046	59856102	+	6010	1.158	1
Sh3bp1	ENSMUSG00000022436	chr15	78730097	78749462	+	3752	1.157	1
Amotl1	ENSMUSG00000013076	chr9	14346410	14447973	-	9927	1.157	1
Dnajc7	ENSMUSG00000014195	chr11	100444132	100481482	-	4863	1.156	1
Dhcr24	ENSMUSG00000034926	chr4	106233643	106261718	+	4028	1.156	1
Aurka	ENSMUSG00000027496	chr2	172181690	172196035	-	2531	1.155	1
Cul4a	ENSMUSG00000031446	chr8	13105621	13147940	+	6408	1.154	1

Apcdd1	ENSMUSG00000071847	chr18	63081981	63112849	+	2799	1.154	1
Mrpl50	ENSMUSG00000044018	chr4	49525468	49533965	-	2114	1.153	1
1700020O03Rik	ENSMUSG00000021254	chr12	87582819	87632364	+	3974	1.153	1
2700038G22Rik	ENSMUSG00000086802	chr5	23356415	23360742	+	1261	1.153	1
BC021614	ENSMUSG00000058216	chr19	4057478	4059295	-	685	1.151	1
Fam46a	ENSMUSG00000032265	chr9	85214046	85220731	-	5479	1.149	1
Wdfy2	ENSMUSG00000014547	chr14	63456527	63575723	+	2011	1.149	1
Glipr2	ENSMUSG00000028480	chr4	43970273	43991990	+	2320	1.148	1
Ripk2	ENSMUSG00000041135	chr4	16049880	16090794	-	2816	1.148	1
Ube3c	ENSMUSG00000039000	chr5	29895782	30002617	+	5033	1.148	1
Prdm15	ENSMUSG00000014039	chr16	98013074	98073457	-	10547	1.148	1
Zfp277	ENSMUSG00000055917	chr12	41041633	41172377	-	3189	1.148	1
Cyth4	ENSMUSG00000018008	chr15	78427477	78452449	+	2791	1.147	1
Phc3	ENSMUSG00000037652	chr3	30798217	30868337	-	14670	1.147	1
Dkc1	ENSMUSG00000031403	chrX	72341193	72355116	+	4121	1.147	1
Zscan21	ENSMUSG00000037017	chr5	138558131	138575493	+	3198	1.147	1
Wdr13	ENSMUSG00000031166	chrX	7700427	7710018	-	5277	1.146	1
Urb2	ENSMUSG00000031976	chr8	126545408	126572405	+	6153	1.146	1
Trps1	ENSMUSG00000038679	chr15	50486307	50721587	-	13325	1.145	1
Ahdc1	ENSMUSG00000037692	chr4	132567175	132634025	+	8444	1.144	1
Zfp866	ENSMUSG00000043090	chr8	72285225	72315077	-	5601	1.144	1
Atg4a	ENSMUSG00000079418	chrX	137491446	137598813	+	2661	1.144	1
Ubap2l	ENSMUSG00000042520	chr3	89803511	89856397	-	5446	1.144	1
1810055G02Rik	ENSMUSG00000035372	chr19	3708333	3717881	+	1882	1.143	1
3110002H16Rik	ENSMUSG00000024410	chr18	12327226	12348506	+	5768	1.142	1
Rbm25	ENSMUSG00000010608	chr12	84973184	85024073	+	4244	1.141	1
Stat3	ENSMUSG00000004040	chr11	100746412	100800854	-	9527	1.139	1
Ube4b	ENSMUSG00000028960	chr4	148702525	148800858	-	7084	1.139	1
Cdkn3	ENSMUSG00000037628	chr14	47380216	47391200	+	1088	1.139	1
Rbpms2	ENSMUSG00000032387	chr9	65477455	65508335	+	2041	1.139	1
Hmg20a	ENSMUSG00000032329	chr9	56266451	56344691	+	3691	1.138	1
Zfp764	ENSMUSG00000045757	chr7	134547182	134550336	-	2508	1.137	1
Nfic	ENSMUSG00000055053	chr10	80858931	80893750	-	6534	1.136	1
D030056L22Rik	ENSMUSG00000047044	chr19	18787683	18792918	+	1677	1.136	1
Mcm9	ENSMUSG00000058298	chr10	53256135	53350245	-	4898	1.136	1

Gtf3c6	ENSMUSG00000019837	chr10	39969009	39977471	-	1020	1.135	1
Hnrnpul 1	ENSMUSG00000040725	chr7	26506517	26539749	-	3589	1.135	1
Nom1	ENSMUSG00000001569	chr5	29761204	29780046	+	4895	1.134	1
Rnf144b	ENSMUSG00000038068	chr13	47218089	47343360	+	4694	1.134	1
Plscr4	ENSMUSG00000032377	chr9	92352216	92388348	+	4639	1.134	1
Taf9	ENSMUSG00000078941	chr13	101421298	101436368	+	988	1.133	1
A73001 7L22Rik	ENSMUSG00000085566	chr2	130698795	130732132	-	6012	1.133	1
Dcaf5	ENSMUSG00000049106	chr12	81436834	81537588	-	5707	1.133	1
Pot1b	ENSMUSG00000024174	chr17	55791323	55851926	-	3499	1.133	1
Ebna1b p2	ENSMUSG00000028729	chr4	118293404	118300381	+	3553	1.132	1
Usp46	ENSMUSG00000054814	chr5	74394487	74464455	-	5533	1.132	1
Rrs1	ENSMUSG00000061024	chr1	9535489	9537535	+	2047	1.131	1
Cog5	ENSMUSG00000035933	chr12	32339734	32622495	+	2997	1.13	1
Hnrnpr	ENSMUSG00000066037	chr4	135866857	135896596	+	3155	1.13	1
E13020 3B14Rik	ENSMUSG00000050666	chr14	33669942	33752673	+	2741	1.129	1
Ddx27	ENSMUSG00000017999	chr2	166840693	166860447	+	4817	1.129	1
Rfxap	ENSMUSG00000036615	chr3	54607038	54611713	-	2161	1.127	1
Ppp2r5e	ENSMUSG00000021051	chr12	76551870	76697232	-	4853	1.127	1
1110067 D22Rik	ENSMUSG00000042363	chr11	20723579	20731059	-	4115	1.126	1
Ankrd10	ENSMUSG00000031508	chr8	11611583	11635754	-	3950	1.126	1
D19Bwg 1357e	ENSMUSG00000041360	chr19	27463192	27504310	-	3462	1.125	1
Zfp37	ENSMUSG00000028389	chr4	61850574	61869480	-	3692	1.125	1
Pwwp2a	ENSMUSG00000044950	chr11	43495500	43534993	+	5508	1.124	1
Hist1h2 bc	ENSMUSG00000018102	chr13	23776068	23784357	+	736	1.124	1
Rfc1	ENSMUSG00000029191	chr5	65653091	65726909	-	6000	1.123	1
Slc7a7	ENSMUSG00000000958	chr14	54989128	55032856	-	2270	1.123	1
Ugp2	ENSMUSG00000001891	chr11	21221141	21271204	-	3069	1.122	1
Birc6	ENSMUSG00000024073	chr17	74927635	75103113	+	16212	1.122	1
2610029 G23Rik	ENSMUSG00000031226	chrX	102275095	102312429	+	6098	1.122	1
Lass5	ENSMUSG00000023021	chr15	99563937	99603316	-	4919	1.121	1
Pik3r6	ENSMUSG00000046207	chr11	68316521	68366200	+	3622	1.121	1
Trmt2b	ENSMUSG00000067369	chrX	130756878	130811523	-	4648	1.121	1
Kifap3	ENSMUSG00000026585	chr1	165709714	165847240	+	4012	1.12	1
Ptprj	ENSMUSG00000025314	chr2	90269911	90420804	-	8378	1.12	1

Pcna	ENSMUSG00000027342	chr2	132074898	132079050	-	2400	1.12	1
3110057O12Rik	ENSMUSG00000037818	chr3	40650892	40742060	+	5593	1.119	1
Pfdn1	ENSMUSG00000024346	chr18	36563333	36614149	-	1077	1.118	1
St3gal2	ENSMUSG00000031749	chr8	113443822	113496380	+	5041	1.118	1
Mrpl39	ENSMUSG00000022889	chr16	84717821	84735987	-	2533	1.118	1
St7	ENSMUSG00000029534	chr6	17642933	17893025	+	9109	1.117	1
Erlin2	ENSMUSG00000031483	chr8	28134331	28149896	+	3874	1.116	1
Csf2ra	ENSMUSG00000059326	chr19	61300305	61304321	-	1849	1.116	1
Rgs10	ENSMUSG00000030844	chr7	135517135	135562272	-	1711	1.115	1
Myo1g	ENSMUSG00000020437	chr11	6406551	6420968	-	6957	1.114	1
Limk1	ENSMUSG00000029674	chr5	135131909	135164460	-	7154	1.114	1
Uvrag	ENSMUSG00000035354	chr7	106035253	106289654	-	3438	1.113	1
Slc29a3	ENSMUSG00000020100	chr10	60174820	60215542	-	5834	1.113	1
Dusp1	ENSMUSG00000024190	chr17	26642535	26645464	-	2697	1.112	1
Ano10	ENSMUSG00000037949	chr9	122084997	122203492	-	2641	1.111	1
Elovl5	ENSMUSG00000032349	chr9	77765171	77832326	+	3522	1.111	1
Exosc1	ENSMUSG00000034321	chr19	41997470	42007804	-	1622	1.109	1
Pms2	ENSMUSG00000079109	chr5	144670833	144698178	+	8442	1.109	1
Fbxl17	ENSMUSG00000023965	chr17	63406801	63849366	-	5526	1.109	1
Ctage5	ENSMUSG00000021000	chr12	60230707	60292570	+	9323	1.108	1
Tbp	ENSMUSG00000014767	chr17	15636852	15665343	+	13399	1.108	1
Pum1	ENSMUSG00000028580	chr4	130219236	130337479	+	6008	1.108	1
Ank3	ENSMUSG00000069601	chr10	68996970	69490184	+	10620	1.107	1
Lair1	ENSMUSG00000055541	chr7	3958675	4014806	-	3776	1.107	1
Dhx57	ENSMUSG00000035051	chr17	80637644	80689816	-	5077	1.106	1
Zfp961	ENSMUSG00000052446	chr8	74474937	74517307	+	3950	1.106	1
Rrn3	ENSMUSG00000022682	chr16	13780792	13814933	+	3597	1.106	1
Stx1a	ENSMUSG00000007207	chr5	135499352	135526970	+	2666	1.106	1
Dcaf10	ENSMUSG00000035572	chr4	45354973	45392631	+	7305	1.105	1
Actr6	ENSMUSG00000019948	chr10	89174718	89195040	-	1703	1.105	1
2700049A03Rik	ENSMUSG00000034601	chr12	72237835	72344290	+	5879	1.104	1
Bahd1	ENSMUSG00000040007	chr2	118726113	118750264	+	4664	1.104	1
Tstd2	ENSMUSG00000035495	chr4	46127618	46151566	-	5570	1.103	1
Mon1b	ENSMUSG00000078908	chr8	116159850	116166305	+	2109	1.103	1
Ggnbp2	ENSMUSG00000020530	chr11	84645863	84684319	-	4726	1.102	1
Lrrn4cl	ENSMUSG00000071656	chr19	8925275	8928399	+	2583	1.101	1
Flna	ENSMUSG00000031328	chrX	71468800	71495159	-	9400	1.1	1
Slc25a16	ENSMUSG00000071253	chr10	62383381	62409246	+	4799	1.1	1
Hey1	ENSMUSG00000040289	chr3	8663362	8667039	-	2203	1.1	1
Msx1	ENSMUSG00000048450	chr5	38211724	38215822	-	1935	1.099	1
Cdca4	ENSMUSG00000047832	chr12	114058448	114067600	-	2417	1.099	1
Ptch1	ENSMUSG00000021466	chr13	63610261	63674474	-	6576	1.099	1

Dnm1	ENSMUSG00000026825	chr2	32163991	32208849	-	5242	1.098	1
Rnf44	ENSMUSG00000034928	chr13	54780762	54795268	-	5200	1.098	1
Pold4	ENSMUSG00000024854	chr19	4231899	4233631	+	910	1.098	1
Anp32b	ENSMUSG00000028333	chr4	46463774	46485529	+	1716	1.097	1
C4b	ENSMUSG00000073418	chr17	34865325	34880827	-	5695	1.095	1
Siae	ENSMUSG00000001942	chr9	37421432	37455903	+	3433	1.094	1
Kcnk5	ENSMUSG00000023243	chr14	20959280	21001004	-	3364	1.094	1
Atf7	ENSMUSG00000071584	chr15	102359067	102359456	-	390	1.094	1
Il20rb	ENSMUSG00000044244	chr9	100358138	100387207	-	4206	1.093	1
Card9	ENSMUSG00000026928	chr2	26207696	26216438	-	2293	1.093	1
Rragc	ENSMUSG00000028646	chr4	123594689	123614240	+	3341	1.093	1
Hmgb3	ENSMUSG00000015217	chrX	68809093	68813851	+	2234	1.093	1
Cnn3	ENSMUSG00000053931	chr3	121129459	121161123	+	2003	1.093	1
Fgfr1op2	ENSMUSG00000040242	chr6	146525725	146547720	+	4619	1.092	1
BC068281	ENSMUSG00000051721	chr12	4850109	4863731	+	3131	1.092	1
Svs5	ENSMUSG00000017004	chr2	164158476	164160132	+	646	1.092	1
Tlr9	ENSMUSG00000045322	chr9	106124929	106129207	+	3471	1.092	1
Ddit3	ENSMUSG00000025408	chr10	126727830	126733344	+	1713	1.09	1
Wdr89	ENSMUSG00000045690	chr12	76732510	76770489	-	2017	1.09	1
Usp28	ENSMUSG00000032267	chr9	48793490	48850622	+	4497	1.09	1
Wdr70	ENSMUSG00000039828	chr15	7823055	8049209	-	2151	1.089	1
Canx	ENSMUSG00000020368	chr11	50107969	50139175	-	4187	1.089	1
Sumf1	ENSMUSG00000030101	chr6	108057022	108135576	-	3405	1.089	1
Paics	ENSMUSG00000029247	chr5	77380332	77396534	+	3139	1.089	1
Blzf1	ENSMUSG00000026577	chr1	166219931	166237620	-	5336	1.089	1
Zc3hav1l	ENSMUSG00000047749	chr6	38237396	38249259	-	6536	1.089	1
2810029C07Rik	ENSMUSG00000091750	chr12	112810532	112812613	-	1950	1.089	1
Cxadr	ENSMUSG00000022865	chr16	78301741	78360030	+	6309	1.088	1
Pdik1l	ENSMUSG00000050890	chr4	133830917	133843810	-	5810	1.087	1
Adamts10	ENSMUSG00000024299	chr17	33661149	33690727	+	5453	1.087	1
Insig1	ENSMUSG00000045294	chr5	28397952	28405201	+	2647	1.087	1
Eno1	ENSMUSG00000063524	chr4	149610830	149622988	+	5379	1.087	1
Camsap1	ENSMUSG00000026933	chr2	25782358	25838802	-	8368	1.086	1
Igbp1	ENSMUSG00000031221	chrX	97689630	97711464	+	5717	1.086	1
Uhrf1	ENSMUSG00000001228	chr17	56442744	56462909	+	4259	1.086	1
Arhgap32	ENSMUSG00000041444	chr9	31923721	32076031	+	16874	1.085	1
Rab27b	ENSMUSG00000024511	chr18	70138785	70301259	-	10679	1.085	1
Dclre1c	ENSMUSG00000026648	chr2	3341403	3381402	+	9316	1.084	1
Ppia	ENSMUSG00000071866	chr11	6315446	6319820	+	3508	1.083	1
Nab2	ENSMUSG00000025402	chr10	127097974	127105624	-	2941	1.083	1

Lyrm2	ENSMUSG00000045854	chr4	32887228	32888534	+	1140	1.082	1
Hoxb6	ENSMUSG00000000690	chr11	96153790	96162883	+	2076	1.081	1
Prpf8	ENSMUSG00000020850	chr11	75300279	75322951	+	7500	1.081	1
Slc25a14	ENSMUSG00000031105	chrX	45976590	46015471	+	2364	1.081	1
Thap2	ENSMUSG00000020137	chr10	114807022	114821491	-	3408	1.08	1
Dnmt3a	ENSMUSG00000020661	chr12	3806007	3914443	+	16642	1.08	1
Larp4	ENSMUSG00000023025	chr15	99800505	99846782	+	6746	1.078	1
Lrrc40	ENSMUSG00000063052	chr3	157699626	157731442	+	4575	1.075	1
Aatf	ENSMUSG00000018697	chr11	84236357	84327024	-	2869	1.074	1
Ptp4a2	ENSMUSG00000028788	chr4	129488463	129527247	+	4377	1.073	1
Snrpb2	ENSMUSG00000008333	chr2	142888775	142898589	+	2857	1.073	1
Luc7l	ENSMUSG00000024188	chr17	26389855	26422449	+	7509	1.073	1
Fam64a	ENSMUSG00000020808	chr11	71855534	71860872	+	2529	1.072	1
Myo5b	ENSMUSG00000025885	chr18	74600590	74931147	+	9775	1.071	1
Wars2	ENSMUSG00000004233	chr3	98944991	99043109	+	5946	1.071	1
Mpdz	ENSMUSG00000028402	chr4	80924404	81088719	-	12440	1.07	1
Tmco1	ENSMUSG00000052428	chr1	169238801	169264109	+	4474	1.069	1
Utp11l	ENSMUSG00000028907	chr4	124355404	124370844	-	4711	1.069	1
Zfp319	ENSMUSG00000046556	chr8	97850037	97855850	-	4106	1.069	1
Dync1li2	ENSMUSG00000035770	chr8	106941574	106966947	-	4633	1.069	1
Cyth1	ENSMUSG00000017132	chr11	117993333	118109906	-	3970	1.068	1
Cnrip1	ENSMUSG00000044629	chr11	16951589	16979374	+	4003	1.068	1
Plk1s1	ENSMUSG00000074749	chr2	146681600	146795833	+	2278	1.067	1
Actb	ENSMUSG00000029580	chr5	143664794	143668433	-	2679	1.067	1
Dhx8	ENSMUSG00000034931	chr11	101594233	101628672	+	5032	1.067	1
Ergic2	ENSMUSG00000030304	chr6	148127601	148160896	-	6727	1.067	1
Fstl3	ENSMUSG00000020325	chr10	79240017	79245375	+	2446	1.065	1
Map3k5	ENSMUSG00000071369	chr10	19654278	19862559	+	6262	1.065	1
Stat1	ENSMUSG00000026104	chr1	52176282	52218704	+	5245	1.065	1
Malat1	ENSMUSG00000092341	chr19	5795690	5802671	-	6982	1.065	1
Snrnp200	ENSMUSG00000003660	chr2	127034122	127066187	+	7819	1.063	1
Phf2	ENSMUSG00000038025	chr13	48897119	48966254	-	5260	1.062	1
Rab5b	ENSMUSG00000000711	chr10	128114249	128133324	-	3166	1.062	1
Zfp113	ENSMUSG00000037007	chr5	138580930	138596972	-	6754	1.061	1
Ttc39c	ENSMUSG00000024424	chr18	12758435	12895559	+	5197	1.06	1
Fads1	ENSMUSG00000010663	chr19	10257378	10271360	+	3456	1.06	1
Anapc1	ENSMUSG00000014355	chr2	128435840	128513127	-	16714	1.059	1
Arl15	ENSMUSG00000042348	chr13	114584713	114947669	+	3364	1.059	1
Gmip	ENSMUSG00000036246	chr8	72332578	72345769	+	5552	1.059	1
Eif5	ENSMUSG00000021282	chr12	112776312	112794270	+	4105	1.059	1
Zxdc	ENSMUSG00000034430	chr6	90319486	90353484	+	8564	1.058	1
Sbds	ENSMUSG00000025337	chr5	130721601	130731400	-	2033	1.058	1
Rps13	ENSMUSG00000090862	chr7	123475021	123477704	-	1377	1.058	1

4930523 C07Rik	ENSMUSG00000090394	chr1	161974515	162008559	+	5431	1.057	1
Pear1	ENSMUSG00000028073	chr3	87551812	87628181	-	6731	1.056	1
Endod1	ENSMUSG00000037419	chr9	14158434	14185951	-	4539	1.055	1
Stau1	ENSMUSG00000039536	chr2	166773049	166821799	-	6065	1.055	1
C1qc	ENSMUSG00000036896	chr4	136445719	136448980	-	1931	1.054	1
9530068 E07Rik	ENSMUSG00000036275	chr11	52209930	52222230	+	3296	1.054	1
Ctcf	ENSMUSG00000005698	chr8	108160468	108206822	+	4860	1.053	1
Kctd5	ENSMUSG00000016946	chr17	24184701	24210452	-	2751	1.053	1
Plxnb2	ENSMUSG00000036606	chr15	88985979	89011218	-	7319	1.053	1
Gprasp1	ENSMUSG00000043384	chrX	132277272	132338013	+	8122	1.053	1
Pramef8	ENSMUSG00000046862	chr4	143002329	143010994	+	4176	1.052	1
Yars2	ENSMUSG00000022792	chr16	16303058	16309733	+	3149	1.052	1
Cdca2	ENSMUSG00000048922	chr14	68294388	68333898	-	5715	1.051	1
Clcn4-2	ENSMUSG00000000605	chr7	7235027	7252222	-	4578	1.051	1
Klf7	ENSMUSG00000025959	chr1	64076021	64168856	-	8133	1.051	1
5430435 G22Rik	ENSMUSG00000052688	chr1	133585272	133610119	+	2727	1.05	1
Nufip1	ENSMUSG00000022009	chr14	76510698	76537186	+	3974	1.05	1
Ostm1	ENSMUSG00000038280	chr10	42303628	42422265	+	7211	1.049	1
Wls	ENSMUSG00000028173	chr3	159502701	159598797	+	3304	1.048	1
Rbm15	ENSMUSG00000048109	chr3	107128863	107136591	-	3819	1.048	1
Ssrp1	ENSMUSG00000027067	chr2	84877355	84887266	+	3788	1.048	1
Prkar2b	ENSMUSG00000002997	chr12	32643341	32746161	-	3652	1.048	1
Cyp2j6	ENSMUSG00000052914	chr4	96182829	96220352	-	3605	1.047	1
4632428 N05Rik	ENSMUSG00000020101	chr10	59809599	59835432	+	5014	1.047	1
Rwdd1	ENSMUSG00000019782	chr10	33716361	33739430	-	1601	1.047	1
mt-Nd4	ENSMUSG00000064363	chrM	10167	11544	+	1378	1.046	1
Mgst1	ENSMUSG00000008540	chr6	138088837	138105276	+	2013	1.046	1
Cd14	ENSMUSG00000051439	chr18	36884728	36886390	-	1572	1.045	1
Braf	ENSMUSG00000002413	chr6	39553236	39675462	-	13862	1.044	1
Ubxn8	ENSMUSG00000052906	chr8	34730058	34752448	-	2830	1.044	1
R3hdm2	ENSMUSG00000025404	chr10	126817383	126936440	+	5987	1.042	1
Eml4	ENSMUSG00000032624	chr17	83750271	83879701	+	5482	1.041	1
Foxo3	ENSMUSG00000048756	chr10	41901647	41996561	-	7571	1.041	1
Tmem1 38	ENSMUSG00000024666	chr19	10644968	10651852	-	1590	1.041	1
Prkci	ENSMUSG00000037643	chr3	30894669	30951663	+	5333	1.04	1
Capg	ENSMUSG00000056737	chr6	72494385	72512977	+	2529	1.04	1
Zfp945	ENSMUSG00000059142	chr17	22983664	23004101	-	10173	1.039	1

Ahi1	ENSMUSG00000019986	chr10	20672391	20800234	+	5094	1.038	1
Ptges3	ENSMUSG00000071072	chr10	127496010	127514328	+	2219	1.038	1
Zc3h7b	ENSMUSG00000022390	chr15	81575278	81626699	+	5958	1.038	1
Rfwd2	ENSMUSG00000040782	chr1	161162457	161277711	+	5056	1.037	1
Fam162a	ENSMUSG00000003955	chr16	36043930	36071601	-	635	1.035	1
Rab35	ENSMUSG00000029518	chr5	116081938	116097745	+	2799	1.034	1
Armcx5	ENSMUSG00000072969	chrX	132277272	132281861	+	4247	1.034	1
Chfr	ENSMUSG00000014668	chr5	110564859	110600991	+	3321	1.033	1
Abhd15	ENSMUSG00000000686	chr11	77328623	77352109	+	3035	1.033	1
Lrrn2	ENSMUSG00000026443	chr1	134776850	134836582	+	3595	1.033	1
mt-Nd5	ENSMUSG00000064367	chrM	11742	13565	+	1824	1.032	1
Tsku	ENSMUSG00000049580	chr7	105499178	105509838	-	2859	1.032	1
Tbc1d4	ENSMUSG00000033083	chr14	101841577	102008408	-	7679	1.031	1
BC031781	ENSMUSG00000038806	chr1	182781282	182798228	+	3173	1.03	1
Nek4	ENSMUSG00000021918	chr14	31764647	31802004	+	4158	1.03	1
Sucla2	ENSMUSG00000022110	chr14	73925126	73995949	+	3988	1.03	1
Synrg	ENSMUSG00000034940	chr11	83777930	83858080	+	8585	1.029	1
Adcy9	ENSMUSG00000005580	chr16	4287529	4420498	-	5197	1.029	1
Rab13	ENSMUSG00000027935	chr3	90017617	90030307	+	1651	1.026	1
Srgap1	ENSMUSG00000020121	chr10	121218047	121484371	-	9132	1.026	1
Vcp	ENSMUSG00000028452	chr4	42992835	43013379	-	4851	1.025	1
Serbp1	ENSMUSG00000036371	chr6	67216973	67239296	+	6685	1.025	1
Hspa13	ENSMUSG00000032932	chr16	75755435	75767066	-	5894	1.025	1
Ighg2b	ENSMUSG00000076613	chr12	114544600	114546144	-	1010	1.025	1
Gm6180	ENSMUSG00000053038	chr8	43332189	43332805	+	617	1.025	1
Il6ra	ENSMUSG00000027947	chr3	89673246	89717084	-	3343	1.024	1
Srbd1	ENSMUSG00000024135	chr17	86384005	86544515	-	3705	1.024	1
Rwdd4a	ENSMUSG00000031568	chr8	48619018	48638179	+	2985	1.024	1
AU019823	ENSMUSG00000059820	chr9	50413345	50425569	-	5321	1.023	1
Trim56	ENSMUSG00000043279	chr5	137581514	137592079	-	9281	1.022	1
Slc38a1	ENSMUSG00000023169	chr15	96401849	96473344	-	8391	1.022	1
Gm4907	ENSMUSG00000068113	chrX	23421830	23484733	+	1641	1.021	1
Dnajc14	ENSMUSG00000025354	chr10	128242690	128256502	+	4271	1.021	1
Dennd5b	ENSMUSG00000030313	chr6	148936593	149050202	-	10621	1.02	1
Tsc22d3	ENSMUSG00000031431	chrX	137074067	137135198	-	4341	1.018	1
Lsm12	ENSMUSG00000020922	chr11	102023811	102046610	-	2388	1.017	1
2010002N04Rik	ENSMUSG00000038059	chr18	60633847	60661637	-	1984	1.016	1
Rsbn1l	ENSMUSG00000039968	chr5	20398846	20457640	-	5550	1.016	1

Tusc3	ENSMUSG00000039530	chr8	40068899	40250466	+	1543	1.016	1
Acbd3	ENSMUSG00000026499	chr1	182656174	182684334	+	3470	1.016	1
Zfp654	ENSMUSG00000047141	chr16	64780177	64786147	-	4741	1.016	1
0610010K14Rik	ENSMUSG00000020831	chr11	70048708	70051416	-	1896	1.014	1
Oip5	ENSMUSG00000072980	chr2	119435248	119444205	-	1778	1.014	1
Nup133	ENSMUSG00000039509	chr8	126421023	126473165	-	5808	1.013	1
Ythdf1	ENSMUSG00000038848	chr2	180639082	180655654	-	3316	1.013	1
Gm16973	ENSMUSG00000086985	chr14	57315117	57320105	-	2421	1.013	1
Tceb1	ENSMUSG00000079658	chr1	16632847	16646946	-	954	1.013	1
6720456H20Rik	ENSMUSG00000036339	chr14	49065803	49134834	+	8189	1.012	1
Zfp828	ENSMUSG00000047710	chr8	13869641	13881639	+	4040	1.012	1
Ap3b1	ENSMUSG00000021686	chr13	95128915	95336271	+	4020	1.011	1
Rprd2	ENSMUSG00000028106	chr3	95563796	95622876	-	9823	1.011	1
Tmem41b	ENSMUSG00000047554	chr7	117115701	117130443	-	5316	1.011	1
Hsd17b4	ENSMUSG00000024507	chr18	50287855	50355923	+	3404	1.01	1
Mettl21d	ENSMUSG00000049882	chr12	70678615	70684015	-	1027	1.01	1
Sod3	ENSMUSG00000072941	chr5	52755185	52762644	+	3570	1.01	1
Pgam1	ENSMUSG00000011752	chr19	41986361	41993154	+	1831	1.01	1
Lin52	ENSMUSG00000085793	chr12	85792458	85872483	+	2144	1.01	1
Pgrmc1	ENSMUSG00000006373	chrX	34138201	34146074	+	1857	1.009	1
Acadl	ENSMUSG00000026003	chr1	66877413	66909851	-	2005	1.009	1
Smn1	ENSMUSG00000021645	chr13	100894807	100907645	+	2592	1.008	1
Tmem5	ENSMUSG00000034620	chr10	121515170	121534427	-	5614	1.006	1
Dpm1	ENSMUSG00000078919	chr2	168034548	168056091	-	7489	1.005	1
Thbs3	ENSMUSG00000028047	chr3	89019102	89030759	+	4823	1.005	1
2200002K05Rik	ENSMUSG00000031937	chr9	15043489	15063837	+	1968	1.004	1
Magoh	ENSMUSG00000028609	chr4	107552360	107560029	+	770	1.003	1
Alg11	ENSMUSG00000063362	chr8	23171193	23182099	+	4960	1.003	1
Ap3m1	ENSMUSG00000021824	chr14	21850664	21871730	-	7015	1.002	1
Supt16h	ENSMUSG00000035726	chr14	52780094	52816914	-	4491	1	0
Atf7ip	ENSMUSG00000053935	chr6	136556350	136559373	+	3024	0.999	0
Rtkn	ENSMUSG00000034930	chr6	83085457	83102573	+	3943	0.998	0
Ier3	ENSMUSG00000003541	chr17	35958629	35959868	+	1131	0.998	0
Larp1	ENSMUSG00000037331	chr11	57822566	57875533	+	6826	0.998	0
Xpc	ENSMUSG00000030094	chr6	91439299	91465878	-	5490	0.995	0
Anxa7	ENSMUSG00000021814	chr14	21274483	21299355	-	3031	0.995	0
Gm505	ENSMUSG00000031986	chr8	127421786	127427713	+	2116	0.995	0
Add3	ENSMUSG00000025026	chr19	53214935	53321889	+	4938	0.992	0

Nol7	ENSMUSG00000063200	chr13	43493745	43498227	+	1811	0.992	0
Klf12	ENSMUSG00000072294	chr14	100269869	100549015	-	5724	0.991	0
Myo1c	ENSMUSG00000017774	chr11	75464006	75488134	+	7307	0.991	0
Gm4885	ENSMUSG00000070035	chr7	79817704	79819164	+	1359	0.991	0
Cyp51	ENSMUSG00000001467	chr5	4081145	4104746	-	4286	0.991	0
Slc9a5	ENSMUSG00000014786	chr8	107872158	107893781	+	5222	0.99	0
Osbpl1a	ENSMUSG00000044252	chr18	12913823	13100350	-	7433	0.99	0
Zfp746	ENSMUSG00000057691	chr6	48012394	48036592	-	3651	0.989	0
St13	ENSMUSG00000022403	chr15	81194099	81230507	-	3457	0.987	0
Luc7l2	ENSMUSG00000029823	chr6	38501334	38559470	+	12084	0.987	0
Ptpn11	ENSMUSG00000043733	chr5	121580542	121641406	-	7492	0.986	0
Tnfrsf11a	ENSMUSG00000026321	chr1	107677300	107744558	+	5022	0.985	0
Osbpl6	ENSMUSG00000042359	chr2	76244565	76438704	+	10063	0.985	0
Fam129b	ENSMUSG00000026796	chr2	32731634	32780774	+	4672	0.985	0
Parp16	ENSMUSG00000032392	chr9	65062497	65087026	+	2708	0.984	0
Cnot6l	ENSMUSG00000034724	chr5	96499352	96593190	-	10313	0.984	0
Cish	ENSMUSG00000032578	chr9	107198357	107205115	+	6419	0.983	0
Ctxn1	ENSMUSG00000048644	chr8	4257648	4259274	-	1224	0.983	0
Tbl1x	ENSMUSG00000025246	chrX	74756352	74908322	+	7052	0.983	0
Ctps2	ENSMUSG00000031360	chrX	159339170	159470440	+	4720	0.983	0
Hic2	ENSMUSG00000050240	chr16	17233665	17263523	+	6354	0.981	0
Dqx1	ENSMUSG00000009145	chr6	83007838	83017204	+	3149	0.978	0
Clcc1	ENSMUSG00000027884	chr3	108456831	108481758	+	4209	0.978	0
Tfam	ENSMUSG00000003923	chr10	70688212	70701028	-	4438	0.978	0
Rundc3b	ENSMUSG00000040570	chr5	8490334	8622952	-	3824	0.977	0
Trpv4	ENSMUSG00000014158	chr5	115072161	115108430	-	4076	0.976	0
Foxk2	ENSMUSG00000039275	chr11	121116808	121171210	+	5591	0.975	0
Unkl	ENSMUSG00000015127	chr17	25325342	25371388	+	5673	0.975	0
Rorb	ENSMUSG00000036192	chr19	19005095	19185686	-	11076	0.975	0
Gm6457	ENSMUSG00000053740	chr18	14728622	14730186	+	1524	0.974	0
Sh3bp5	ENSMUSG00000021892	chr14	32173066	32249264	-	3194	0.974	0
Fgd2	ENSMUSG00000024013	chr17	29497859	29516605	+	4134	0.973	0
Kif18b	ENSMUSG00000051378	chr11	102766843	102786438	-	3572	0.973	0
Gphn	ENSMUSG00000047454	chr12	79327642	79785754	+	3397	0.973	0
Terf2	ENSMUSG00000031921	chr8	109593300	109620447	-	4597	0.972	0
Pon2	ENSMUSG00000032667	chr6	5214147	5248455	-	4655	0.971	0
Sdc3	ENSMUSG00000025743	chr4	130348452	130382234	+	5989	0.971	0
Lnx2	ENSMUSG00000016520	chr5	147828231	147888162	-	4557	0.97	0
Arhgap29	ENSMUSG00000039831	chr3	121656147	121719595	+	8361	0.969	0
Mecp2	ENSMUSG00000031393	chrX	71271931	71331029	-	10559	0.969	0

4933407H18Rik	ENSMUSG00000037355	chr5	33721200	33762403	+	8664	0.969	0
6720401G13Rik	ENSMUSG00000085396	chrX	47908921	47988498	-	1439	0.969	0
Ccdc45	ENSMUSG00000018372	chr11	106650566	106681244	+	4199	0.969	0
Dcp1a	ENSMUSG00000021962	chr14	31292751	31340242	+	5616	0.968	0
Slc12a6	ENSMUSG00000027130	chr2	112105982	112246476	+	10873	0.968	0
Phf10	ENSMUSG00000023883	chr17	15081999	15129820	-	5010	0.967	0
Slc35f5	ENSMUSG00000026342	chr1	127457593	127492261	+	2597	0.967	0
Zfp958	ENSMUSG00000058748	chr8	4625840	4629380	+	1401	0.966	0
Slc12a7	ENSMUSG00000017756	chr13	73870542	73954191	+	5744	0.966	0
Vps37a	ENSMUSG00000031600	chr8	41597137	41636488	+	6445	0.965	0
Figf	ENSMUSG00000031380	chrX	160811310	160840582	+	2408	0.965	0
Dph5	ENSMUSG00000033554	chr3	115591083	115637279	+	3765	0.965	0
Gm4540	ENSMUSG00000092072	chr3	105837628	105837921	+	294	0.965	0
Tmem88	ENSMUSG00000045377	chr11	69210021	69211736	-	1582	0.964	0
Zfp9	ENSMUSG00000072623	chr6	118411968	118429338	-	3968	0.964	0
Zfp874b	ENSMUSG00000059839	chr13	67572450	67585189	-	3771	0.963	0
Ccnf	ENSMUSG00000072082	chr17	24359494	24388292	-	3834	0.963	0
Rbm5	ENSMUSG00000032580	chr9	107642826	107673333	-	3087	0.963	0
Mid2	ENSMUSG00000000266	chrX	137199138	137302254	+	8114	0.962	0
Tek	ENSMUSG00000006386	chr4	94405980	94541667	+	4712	0.962	0
Rps6kc1	ENSMUSG00000089872	chr1	192524081	192735649	-	9741	0.961	0
Abl1	ENSMUSG00000026842	chr2	31543896	31659747	+	11596	0.96	0
Dbi	ENSMUSG00000026385	chr1	122009857	122017655	-	1422	0.959	0
Maf	ENSMUSG00000055435	chr8	118206842	118231694	-	8241	0.959	0
Ccnl2	ENSMUSG00000029068	chr4	155186598	155198652	+	5278	0.959	0
Tmem43	ENSMUSG00000030095	chr6	91423697	91438453	+	3741	0.959	0
Maea	ENSMUSG00000079562	chr5	33678214	33715942	+	2170	0.958	0
Ubqln1	ENSMUSG00000005312	chr13	58277524	58316997	-	3662	0.957	0
Acad9	ENSMUSG00000027710	chr3	35964901	35993866	+	8323	0.956	0
Hoxa7	ENSMUSG00000038236	chr6	52164490	52171853	-	2877	0.955	0
Papss2	ENSMUSG00000024899	chr19	32694495	32741677	+	3635	0.954	0
Ankrd26	ENSMUSG00000007827	chr6	118452582	118512274	-	5704	0.954	0
Hoxb2	ENSMUSG00000075588	chr11	96212946	96217003	+	3304	0.953	0
Birc2	ENSMUSG00000057367	chr9	7818236	7835255	-	3130	0.952	0
Grlf1	ENSMUSG00000058230	chr7	17079822	17200342	-	8393	0.952	0
Gm17329	ENSMUSG00000091342	chr11	94810494	94811645	-	141	0.952	0
Lrrfip2	ENSMUSG00000032497	chr9	111020615	111128172	+	3533	0.951	0

Fam168a	ENSMUSG00000029461	chr7	107855165	107990142	+	6659	0.951	0
Cmpk1	ENSMUSG00000028719	chr4	114631941	114659846	-	3420	0.95	0
Ikbkap	ENSMUSG00000028431	chr4	56762552	56815203	-	7399	0.949	0
Emp3	ENSMUSG00000040212	chr7	53173393	53176796	-	972	0.949	0
Pdk3	ENSMUSG00000035232	chrX	91009946	91077540	-	2561	0.949	0
Ets2	ENSMUSG00000022895	chr16	95923682	95942658	+	4710	0.949	0
Phf17	ENSMUSG00000025764	chr3	41359656	41420784	+	5950	0.948	0
Aoc2	ENSMUSG00000078651	chr11	101186377	101191016	+	2543	0.947	0
Fermt3	ENSMUSG00000024965	chr19	7073448	7093959	-	2587	0.946	0
Cisd2	ENSMUSG00000028165	chr3	135069376	135086889	-	4360	0.945	0
Zfp933	ENSMUSG00000059423	chr4	147197095	147222475	-	4270	0.944	0
Slc41a2	ENSMUSG00000034591	chr10	82693593	82800627	-	5349	0.944	0
Sephs1	ENSMUSG00000026662	chr2	4802610	4831603	+	5925	0.943	0
Rnf138	ENSMUSG00000024317	chr18	21159842	21186724	+	3110	0.943	0
Usp3	ENSMUSG00000032376	chr9	66362444	66440949	-	6848	0.942	0
Igfbp5	ENSMUSG00000026185	chr1	72904506	72921458	-	6006	0.942	0
Sfmbt1	ENSMUSG00000006527	chr14	31528035	31635899	+	8629	0.941	0
Ilf3	ENSMUSG00000032178	chr9	21172387	21209794	+	4858	0.94	0
Stx16	ENSMUSG00000027522	chr2	173901809	173925272	+	3968	0.94	0
Plekhb2	ENSMUSG00000026123	chr1	34906804	34936425	+	8004	0.94	0
Mest	ENSMUSG00000051855	chr6	30673547	30698457	+	3937	0.939	0
Gsto1	ENSMUSG00000025068	chr19	47929460	47939280	+	1968	0.939	0
Cnot4	ENSMUSG00000038784	chr6	34972066	35083737	-	7185	0.939	0
Calm1	ENSMUSG00000001175	chr12	101437645	101448016	+	4698	0.939	0
Brd8	ENSMUSG00000003778	chr18	34758269	34784255	-	10633	0.939	0
9230112E08Rik	ENSMUSG00000070461	chr2	180718937	180720296	+	1360	0.936	0
1700094D03Rik	ENSMUSG00000078667	chr3	89866718	89872269	+	3097	0.935	0
Haus1	ENSMUSG00000041840	chr18	77996306	78006519	-	1004	0.935	0
3830406C13Rik	ENSMUSG00000033111	chr14	13116723	13135745	+	2553	0.935	0
Ndufa4l2	ENSMUSG00000040280	chr10	126951995	126954208	+	960	0.935	0
Poc5	ENSMUSG00000021671	chr13	97158249	97185542	+	3033	0.934	0
Gm11818	ENSMUSG00000055963	chr4	12833985	12908618	+	1486	0.934	0
Tmem57	ENSMUSG00000028826	chr4	134358674	134409260	-	6744	0.934	0
Tnpo3	ENSMUSG00000012535	chr6	29490827	29559887	-	10246	0.932	0
Pja1	ENSMUSG00000034403	chrX	96661073	96666612	-	3324	0.932	0
Bcl2	ENSMUSG00000057329	chr1	108434755	108610851	-	7191	0.931	0

4933439F18Rik	ENSMUSG00000018415	chr11	60230647	60264429	+	4864	0.93		0
Usp42	ENSMUSG00000051306	chr5	144471194	144493149	-	6238	0.93		0
Parp14	ENSMUSG00000034422	chr16	35832960	35871630	-	9306	0.93		0
Trp53bp1	ENSMUSG00000043909	chr2	121019017	121097143	-	14164	0.93		0
4922501C03Rik	ENSMUSG00000056919	chr9	87084408	87150371	-	9151	0.929		0
Wwc2	ENSMUSG00000031563	chr8	48911437	49075897	-	6546	0.928		0
Slc41a1	ENSMUSG00000013275	chr1	133724070	133745442	+	4912	0.928		0
Slpi	ENSMUSG00000017002	chr2	164179806	164214831	-	1180	0.927		0
Fkbp10	ENSMUSG00000001555	chr11	100277011	100286138	+	2935	0.927		0
Tada2a	ENSMUSG00000018651	chr11	83892336	83943102	-	3062	0.927		0
Polr2a	ENSMUSG00000005198	chr11	69547499	69572139	-	7067	0.927		0
Dnaja2	ENSMUSG00000031701	chr8	88061539	88079170	-	2839	0.925		0
2310037I24Rik	ENSMUSG00000022992	chr15	98349243	98364669	-	2420	0.924		0
Atp11a	ENSMUSG00000031441	chr8	12757014	12868728	+	11150	0.924		0
Morc4	ENSMUSG00000031434	chrX	136356171	136406216	-	6051	0.923		0
Pde4d	ENSMUSG00000074661	chr13	110744317	110746171	+	1855	0.923		0
Ccdc127	ENSMUSG00000021578	chr13	74487757	74503231	+	9559	0.923		0
Nde1	ENSMUSG00000022678	chr16	14163368	14193021	+	3205	0.923		0
Cebpz	ENSMUSG00000024081	chr17	79318346	79336410	-	4074	0.923		0
Lrba	ENSMUSG00000028080	chr3	86028602	86586615	+	9954	0.922		0
Tjp2	ENSMUSG00000024812	chr19	24169013	24299444	-	4613	0.921		0
Rfc4	ENSMUSG00000022881	chr16	23114016	23127810	-	1534	0.92		0
Fbxo45	ENSMUSG00000035764	chr16	32230198	32247244	-	4179	0.919		0
Clpx	ENSMUSG00000015357	chr9	65142067	65178465	+	3494	0.918		0
Ube2q2	ENSMUSG00000032307	chr9	54996661	55055336	+	4305	0.917		0
Tmem37	ENSMUSG00000050777	chr1	121963954	121970651	-	1311	0.916		0
Naa30	ENSMUSG00000036282	chr14	49791901	49810706	+	6970	0.916		0
Smcr7l	ENSMUSG00000022412	chr15	80064510	80083796	+	5541	0.916		0
Spin1	ENSMUSG00000021395	chr13	51196267	51247915	+	4480	0.915		0
Srsf4	ENSMUSG00000028911	chr4	131429532	131457640	+	3580	0.914		0
Trac	ENSMUSG00000076928	chr14	54840196	54842772	+	413	0.914		0
Wrnip1	ENSMUSG00000021400	chr13	32893907	32914478	+	2633	0.912		0
Ddx17	ENSMUSG00000055065	chr15	79358137	79377171	-	4761	0.911		0
Sema4b	ENSMUSG00000030539	chr7	87331727	87371280	+	5434	0.911		0
Slc10a7	ENSMUSG00000031684	chr8	81033234	81257904	+	3630	0.911		0
Neat1	ENSMUSG00000092274	chr19	5824708	5845478	-	20771	0.91		0
Pfkfb2	ENSMUSG00000026409	chr1	132585620	132625830	-	10224	0.909		0
Glrx	ENSMUSG00000021591	chr13	75977316	75987599	+	1345	0.909		0

Rps6kb 1	ENSMUSG00000020516	chr11	86312373	86358307	-	8658	0.909	0
Exoc6b	ENSMUSG00000033769	chr6	84568480	85019507	-	11381	0.908	0
Ppp2r3a	ENSMUSG00000043154	chr9	101007322	101154162	-	7458	0.908	0
Zfp462	ENSMUSG00000060206	chr4	54957920	55096435	+	10702	0.908	0
Fam108 c	ENSMUSG00000038459	chr7	91257866	91300403	-	2415	0.906	0
Tnrc18	ENSMUSG00000039477	chr5	143512088	143579341	-	6912	0.905	0
Gypc	ENSMUSG00000090523	chr18	32687976	32719688	-	2331	0.904	0
Tpt1	ENSMUSG00000060126	chr14	76244900	76248332	+	2380	0.903	0
Otud7b	ENSMUSG00000038495	chr3	95908450	95965052	+	8497	0.903	0
Mfap1b	ENSMUSG00000048222	chr2	121285971	121299803	-	3362	0.903	0
Spns2	ENSMUSG00000040447	chr11	72265140	72303463	-	4124	0.902	0
Gpkow	ENSMUSG00000031148	chrX	7274257	7287388	+	3737	0.902	0
Rcbtb2	ENSMUSG00000022106	chr14	73522844	73607650	+	9489	0.902	0
Glis2	ENSMUSG00000014303	chr16	4594713	4624924	+	4373	0.902	0
1110021 L09Rik	ENSMUSG00000037455	chr10	23516792	23547774	+	3252	0.901	0
Pitpnb	ENSMUSG00000050017	chr5	111759783	111817379	+	2761	0.901	0
Minpp1	ENSMUSG00000024896	chr19	32560259	32589860	+	2619	0.901	0
Ptpn14	ENSMUSG00000026604	chr1	191552147	191700574	+	11573	0.901	0
Alkbh5	ENSMUSG00000042650	chr11	60349883	60372014	+	5987	0.901	0
Fam20b	ENSMUSG00000033557	chr1	158608676	158649132	-	8389	0.901	0
Zcrb1	ENSMUSG00000022635	chr15	93216528	93228765	-	3074	0.9	0
Smad4	ENSMUSG00000024515	chr18	73798663	73863434	-	3712	0.9	0
Itm2a	ENSMUSG00000031239	chrX	104592438	104598715	-	1744	0.9	0
Vkorc1l1	ENSMUSG00000066735	chr5	130417979	130460615	+	2972	0.898	0
Sar1a	ENSMUSG00000020088	chr10	61143058	61156043	+	2611	0.898	0
Arfip1	ENSMUSG00000074513	chr3	84300015	85691438	-	3705	0.897	0
Rsbn1	ENSMUSG00000044098	chr3	103718043	103770559	+	6861	0.897	0
G3bp2	ENSMUSG00000029405	chr5	92481172	92512761	-	4677	0.897	0
Ercc3	ENSMUSG00000024382	chr18	32399954	32429805	+	2835	0.896	0
Myo6	ENSMUSG00000033577	chr9	80012838	80159536	+	9388	0.896	0
Ppl	ENSMUSG00000039457	chr16	5086384	5132574	-	6277	0.895	0
Pnrc2	ENSMUSG00000028675	chr4	135426833	135429765	-	2213	0.894	0
Tmem2 12	ENSMUSG00000043164	chr3	27764988	27795290	-	716	0.894	0
Smc1a	ENSMUSG00000041133	chrX	148450971	148497237	+	5769	0.893	0
1110054 O05Rik	ENSMUSG00000038544	chr4	59782509	59814461	-	5995	0.892	0
Fam83b	ENSMUSG00000032358	chr9	76338512	76393611	-	3136	0.892	0
Meis1	ENSMUSG00000020160	chr11	18780433	18918961	-	9465	0.892	0
Atg14	ENSMUSG00000037526	chr14	48160569	48188109	-	3450	0.89	0

AI467606	ENSMUSG00000045165	chr7	134234950	134237563	+	1918	0.889	0
Armc10	ENSMUSG00000038525	chr5	21151631	21168512	+	3407	0.888	0
Rbbp6	ENSMUSG00000030779	chr7	130109200	130146071	+	8752	0.888	0
Dtna	ENSMUSG00000024302	chr18	23573916	23818215	+	9409	0.887	0
Wtap	ENSMUSG00000060475	chr17	13159662	13185412	-	5651	0.887	0
Ube4a	ENSMUSG00000059890	chr9	44731214	44773683	-	8635	0.887	0
1700001L05Rik	ENSMUSG00000075511	chr15	83187990	83197707	-	2270	0.887	0
Dnajc8	ENSMUSG00000054405	chr4	132091465	132109657	+	2555	0.886	0
Nars	ENSMUSG00000024587	chr18	64659319	64676211	-	2632	0.886	0
Krt10	ENSMUSG00000019761	chr11	99246568	99250678	-	2094	0.885	0
Lrg1	ENSMUSG00000037095	chr17	56259103	56261369	-	1351	0.885	0
Commd3	ENSMUSG00000051154	chr2	18594011	18597858	+	2330	0.884	0
Rpl36a	ENSMUSG00000079435	chrX	131120193	131122601	+	1655	0.884	0
Eif5b	ENSMUSG00000026083	chr1	38054855	38112424	+	7797	0.884	0
Zmynd15	ENSMUSG00000040829	chr11	70272935	70279704	+	4409	0.884	0
Nup50	ENSMUSG00000016619	chr15	84753831	84773385	+	4896	0.884	0
H2afy	ENSMUSG00000015937	chr13	56174980	56237722	-	6809	0.884	0
Uso1	ENSMUSG00000029407	chr5	92566964	92631820	+	3897	0.883	0
Cln5	ENSMUSG00000022125	chr14	103469433	103476796	+	2396	0.882	0
Mast2	ENSMUSG00000003810	chr4	115979367	116136788	-	7645	0.88	0
Sar1b	ENSMUSG00000020386	chr11	51577189	51605427	+	1255	0.88	0
Meis2	ENSMUSG00000027210	chr2	115688800	115891575	-	11969	0.88	0
Med6	ENSMUSG00000002679	chr12	82674544	82695995	-	1447	0.879	0
Elp4	ENSMUSG00000027167	chr2	105537477	105744721	-	7025	0.879	0
Dnajc19	ENSMUSG00000027679	chr3	33954942	33980243	-	3998	0.879	0
Atxn7l1	ENSMUSG00000020564	chr12	33832558	34060524	+	12523	0.879	0
Itfg1	ENSMUSG00000031703	chr8	88241456	88364848	-	3355	0.878	0
Pknox1	ENSMUSG00000006705	chr17	31701694	31744625	+	6888	0.878	0
Nt5dc1	ENSMUSG00000039480	chr10	34008094	34138358	-	3192	0.878	0
Tgfb3	ENSMUSG00000021253	chr12	87397694	87419991	-	3384	0.878	0
Set	ENSMUSG00000054766	chr2	29912898	29928097	+	4070	0.878	0
Pik3c3	ENSMUSG00000033628	chr18	30432401	30507780	+	4466	0.877	0
Pigg	ENSMUSG00000029263	chr5	108741628	108778374	+	4573	0.876	0
Eri2	ENSMUSG00000030929	chr7	126912193	126937572	-	7170	0.875	0
Tmco3	ENSMUSG00000038497	chr8	13288013	13322924	+	4575	0.875	0
Chd4	ENSMUSG00000063870	chr6	125045999	125080609	+	8019	0.875	0
Ccnt1	ENSMUSG00000011960	chr15	98369120	98401354	-	9157	0.874	0
Sspn	ENSMUSG00000030255	chr6	145880161	145913743	+	4996	0.873	0
Tmem154	ENSMUSG00000056498	chr3	84470114	84508497	+	4738	0.873	0
Tmem18	ENSMUSG00000043061	chr12	31269308	31276078	+	2952	0.872	0

Ankrd11	ENSMUSG00000035569	chr8	125407221	125565897	-	11702	0.872	0
Ppapdc 2	ENSMUSG00000040105	chr19	29038410	29041288	+	2879	0.872	0
Timp3	ENSMUSG00000020044	chr10	85763117	85812251	+	8144	0.872	0
Cox15	ENSMUSG00000040018	chr19	43807744	43827490	-	4909	0.87	0
5730494 N06Rik	ENSMUSG00000027341	chr2	132065228	132073543	-	3267	0.869	0
Zfp330	ENSMUSG00000031711	chr8	85287520	85298059	-	1675	0.869	0
Tecpr1	ENSMUSG00000066621	chr5	144955311	144984484	-	9665	0.868	0
Parp6	ENSMUSG00000025237	chr9	59465091	59498086	+	2703	0.868	0
Thy1	ENSMUSG00000032011	chr9	43851467	43856662	+	1735	0.868	0
Pwp1	ENSMUSG00000001785	chr10	85334576	85351841	+	2730	0.868	0
Ehmt1	ENSMUSG00000036893	chr2	24646289	24775129	-	7115	0.868	0
Mfn1	ENSMUSG00000027668	chr3	32428387	32478161	+	5905	0.867	0
Hbp1	ENSMUSG00000002996	chr12	32611119	32635400	-	6167	0.866	0
Ube2n	ENSMUSG00000074781	chr10	94977779	95008292	+	3868	0.866	0
Ppp1cc	ENSMUSG00000004455	chr5	122608287	122625282	+	3424	0.865	0
Hsp90b 1	ENSMUSG00000020048	chr10	86152954	86168254	-	4032	0.864	0
Pmp22	ENSMUSG00000018217	chr11	62942484	62973049	+	2354	0.861	0
Mageh1	ENSMUSG00000047238	chrX	149470709	149472106	-	1398	0.86	0
Rftn2	ENSMUSG00000025978	chr1	55227003	55283626	-	5871	0.86	0
Eef1e1	ENSMUSG00000001707	chr13	38737567	38750897	-	1032	0.86	0
Prkar1a	ENSMUSG00000020612	chr11	109510719	109530970	+	4599	0.859	0
1110034 A24Rik	ENSMUSG00000020973	chr12	70286878	70299416	-	3411	0.858	0
Gtdc1	ENSMUSG00000036890	chr2	44419932	44783177	-	7958	0.858	0
Tes	ENSMUSG00000029552	chr6	17015149	17055828	+	3199	0.858	0
Atp5j	ENSMUSG00000022890	chr16	84828111	84835870	-	3130	0.858	0
Pdgfa	ENSMUSG00000025856	chr5	139451968	139473324	-	3119	0.858	0
Tubgcp3	ENSMUSG00000000759	chr8	12614277	12672248	-	8568	0.857	0
Sypl	ENSMUSG00000020570	chr12	33638756	33663915	+	7535	0.855	0
Slc5a3	ENSMUSG00000089774	chr16	92058567	92087718	+	10913	0.855	0
Tspan5	ENSMUSG00000028152	chr3	138405159	138567397	+	3917	0.855	0
Kifc1	ENSMUSG00000079553	chr17	34012611	34027606	-	3407	0.855	0
Zmym5	ENSMUSG00000040123	chr14	57409425	57430553	-	5482	0.853	0
Nat10	ENSMUSG00000027185	chr2	103561413	103601427	-	7199	0.853	0
Vps36	ENSMUSG00000031479	chr8	23303281	23331315	+	3706	0.852	0
Nr6a1	ENSMUSG00000063972	chr2	38578890	38783208	-	7504	0.852	0
Rfesd	ENSMUSG00000043190	chr13	76138983	76156160	-	2862	0.852	0
Cops5	ENSMUSG00000025917	chr1	10014683	10028208	-	3334	0.852	0
Arhgap2 6	ENSMUSG00000036452	chr18	38761188	39535938	+	17331	0.852	0
Rnf34	ENSMUSG00000029474	chr5	123300197	123318954	+	1954	0.852	0
Ipo11	ENSMUSG00000042590	chr13	107584519	107726995	-	4400	0.851	0

Gpr153	ENSMUSG00000042804	chr4	151648341	151659446	+	3909	0.851	0
Sox18	ENSMUSG00000046470	chr2	181404541	181406345	-	1620	0.851	0
Zfp142	ENSMUSG00000026135	chr1	74611701	74634820	-	11355	0.85	0
Rnf20	ENSMUSG00000028309	chr4	49644878	49669759	+	7288	0.849	0
Hmgxb4	ENSMUSG00000034518	chr8	77517255	77555877	+	5227	0.849	0
Itih4	ENSMUSG00000021922	chr14	31699662	31715167	+	3318	0.848	0
Got1	ENSMUSG00000025190	chr19	43574242	43599095	-	2539	0.848	0
0610007 P08Rik	ENSMUSG00000021470	chr13	63916548	64001610	+	7681	0.847	0
Inha	ENSMUSG00000032968	chr1	75503652	75506941	+	1475	0.845	0
Phlpp1	ENSMUSG00000044340	chr1	108068329	108290827	+	6736	0.845	0
Dhx29	ENSMUSG00000042426	chr13	113717938	113759639	+	4405	0.845	0
N4bp1	ENSMUSG00000031652	chr8	89365038	89409157	-	6469	0.843	0
Sec24d	ENSMUSG00000039234	chr3	122970414	123068553	+	3856	0.843	0
9030624 J02Rik	ENSMUSG00000030982	chr7	125883740	125986480	+	8969	0.842	0
Atxn10	ENSMUSG00000016541	chr15	85166768	85294266	+	2479	0.841	0
Ccdc10 2a	ENSMUSG00000063605	chr8	97426769	97441998	-	2504	0.841	0
Sf3b3	ENSMUSG00000033732	chr8	113334139	113370703	-	4552	0.84	0
Mphosp h8	ENSMUSG00000079184	chr14	57287085	57316267	+	3586	0.839	0
C03003 7D09Rik	ENSMUSG00000087574	chr11	88579959	88590207	+	4199	0.838	0
Ttyh3	ENSMUSG00000036565	chr5	141096540	141124985	-	4650	0.837	0
Btbd19	ENSMUSG00000073771	chr4	116791824	116798271	-	2853	0.836	0
Camkk2	ENSMUSG00000029471	chr5	123183213	123229391	-	3177	0.836	0
Irak2	ENSMUSG00000060477	chr6	113588461	113645003	+	5537	0.835	0
Pgm3	ENSMUSG00000056131	chr9	86446083	86465449	-	4582	0.835	0
Npr2	ENSMUSG00000028469	chr4	43644807	43664116	+	4667	0.835	0
Prosc	ENSMUSG00000031485	chr8	28153027	28166603	+	4534	0.834	0
Samd4b	ENSMUSG00000037513	chr7	29184568	29208857	-	4311	0.833	0
1300010 F03Rik	ENSMUSG00000058997	chr14	79248985	79602117	+	8702	0.833	0
Dusp2	ENSMUSG00000027368	chr2	127161895	127164112	+	2003	0.831	0
Ipp	ENSMUSG00000028696	chr4	116180154	116210848	+	2134	0.831	0
Zfp472	ENSMUSG00000053600	chr17	33102759	33116178	+	2376	0.83	0
Isoc1	ENSMUSG00000024601	chr18	58819136	58839224	+	2563	0.828	0
Gm1534 4	ENSMUSG00000084899	chr10	86154864	86155840	-	421	0.827	0
Ostf1	ENSMUSG00000024725	chr19	18653818	18706279	-	4784	0.826	0
Man2b2	ENSMUSG00000029119	chr5	37198160	37221892	-	3528	0.825	0
Spag7	ENSMUSG00000018287	chr11	70477273	70482918	-	1480	0.824	0
Rai1	ENSMUSG00000062115	chr11	59918515	60012699	+	9591	0.823	0

Lama5	ENSMUSG00000015647	chr2	179911078	179960564	-	11404	0.823		0
Plek2	ENSMUSG00000021118	chr12	79989678	80007951	-	1583	0.822		0
BC048507	ENSMUSG00000064063	chr13	67964274	67964856	+	583	0.822		0
Fkbp1a	ENSMUSG00000032966	chr2	151368219	151387428	+	3857	0.82		0
Rara	ENSMUSG00000037992	chr11	98789132	98836256	+	4573	0.819		0
Fkbp9	ENSMUSG00000029781	chr6	56782053	56829352	+	3314	0.819		0
Fnbp1	ENSMUSG00000075415	chr2	30881726	30997528	-	10046	0.819		0
Sfrs18	ENSMUSG00000028248	chr4	21774730	21803622	+	10538	0.818		0
Slc41a3	ENSMUSG00000030089	chr6	90554719	90596406	+	2812	0.818		0
Snapc5	ENSMUSG00000032398	chr9	64027104	64030491	+	757	0.818		0
Srsf11	ENSMUSG00000055436	chr3	157673437	157699603	-	7135	0.816		0
Htatsf1	ENSMUSG00000067873	chrX	54306760	54320360	+	4879	0.816		0
Ero1lb	ENSMUSG00000057069	chr13	12658150	12701793	+	4237	0.815		0
Stambp	ENSMUSG00000006906	chr6	83493200	83522498	-	2157	0.814		0
Nupl2	ENSMUSG00000048439	chr5	23670781	23689831	+	3768	0.814		0
Mrpl32	ENSMUSG00000015672	chr13	14702568	14705304	-	768	0.813		0
Myct1	ENSMUSG00000046916	chr10	4739754	4752813	-	2775	0.812		0
Plxna3	ENSMUSG00000031398	chrX	71574405	71590028	+	7880	0.811		0
Cyp4f18	ENSMUSG00000003484	chr8	74512381	74533525	-	1855	0.81		0
Asf1b	ENSMUSG00000005470	chr8	86479406	86494096	+	2338	0.807		0
Helz	ENSMUSG00000020721	chr11	107409244	107555140	+	15044	0.805		0
Fchsd2	ENSMUSG00000030691	chr7	108257289	108432919	+	9199	0.805		0
Copb2	ENSMUSG00000032458	chr9	98464150	98488794	+	3043	0.803		0
Gna13	ENSMUSG00000020611	chr11	109224145	109262683	+	6421	0.802		0
Rhbdf2	ENSMUSG00000020806	chr11	116459479	116488333	-	5293	0.8		0
Anp32a	ENSMUSG00000032249	chr9	62189100	62226619	+	3166	0.799		0
Gnb1	ENSMUSG00000029064	chr4	154865470	154933378	+	4268	0.799		0
Stat6	ENSMUSG00000002147	chr10	127080042	127098013	+	4402	0.798		0
Fbxl5	ENSMUSG00000039753	chr5	44135855	44212878	-	9788	0.797		0
Ccni	ENSMUSG00000063015	chr5	93610959	93635521	-	3303	0.796		0
Heatr1	ENSMUSG00000050244	chr13	12487665	12530944	+	6537	0.796		0
Helq	ENSMUSG00000035266	chr5	101191164	101227619	-	5061	0.794		0
Slc26a11	ENSMUSG00000039908	chr11	119216871	119242393	+	3610	0.792		0
Mut	ENSMUSG00000023921	chr17	41071656	41098582	+	3297	0.791		0
Bag3	ENSMUSG00000030847	chr7	135667097	135690491	+	2591	0.791		0
BC005561	ENSMUSG00000079065	chr5	104937371	104951402	+	5181	0.791		0
Gpatch4	ENSMUSG00000028069	chr3	87847030	87859915	+	1768	0.791		0
Erap1	ENSMUSG00000021583	chr13	74777320	74829983	+	4674	0.791		0
Wdr82	ENSMUSG00000020257	chr9	106073260	106094747	+	5017	0.79		0
9930104L06Rik	ENSMUSG00000044730	chr4	124614292	124621900	+	5592	0.79		0

Setd6	ENSMUSG00000031671	chr8	98239815	98242910	+	1869	0.79	0
Ppp6r3	ENSMUSG00000024908	chr19	3454933	3575749	-	4952	0.789	0
Pex1	ENSMUSG00000005907	chr5	3596066	3637232	+	7740	0.789	0
Tnrc6a	ENSMUSG00000052707	chr7	130267770	130338802	+	8247	0.789	0
Abca7	ENSMUSG00000035722	chr10	79459239	79478317	+	6752	0.788	0
Dlst	ENSMUSG00000004789	chr12	86451783	86475041	+	2781	0.788	0
Cdadc1	ENSMUSG00000021982	chr14	60179733	60216673	-	1901	0.788	0
Slc9a6	ENSMUSG00000060681	chrX	53862934	53917407	+	4986	0.787	0
Gapvd1	ENSMUSG00000026867	chr2	34531704	34625210	-	8441	0.787	0
Lpin2	ENSMUSG00000024052	chr17	71531900	71599157	+	12233	0.787	0
Llph	ENSMUSG00000020224	chr10	119664126	119669127	+	1517	0.786	0
Cyp7b1	ENSMUSG00000039519	chr3	17971950	18143338	-	2166	0.785	0
Ift88	ENSMUSG00000040040	chr14	58042899	58136773	+	4628	0.785	0
Cnih2	ENSMUSG00000024873	chr19	5092871	5098418	-	1237	0.783	0
1190002 N15Rik	ENSMUSG00000045414	chr9	94418283	94438500	-	3950	0.782	0
Crtc3	ENSMUSG00000030527	chr7	87731513	87833763	-	9850	0.782	0
Bcl10	ENSMUSG00000028191	chr3	145587322	145597247	+	1780	0.782	0
Kif1b	ENSMUSG00000063077	chr4	148550428	148681802	-	17608	0.779	0
Zfp202	ENSMUSG00000025602	chr9	39999901	40021189	+	3877	0.779	0
Tusc1	ENSMUSG00000054000	chr4	93000839	93002202	-	1364	0.778	0
Smo	ENSMUSG00000001761	chr6	29685694	29711365	+	3779	0.777	0
Dact1	ENSMUSG00000044548	chr12	72410871	72421094	+	3761	0.777	0
Osr1	ENSMUSG00000048387	chr12	9581248	9588305	+	2010	0.777	0
Lsg1	ENSMUSG00000022538	chr16	30560580	30587678	-	5555	0.777	0
Ppp1r7	ENSMUSG00000026275	chr1	95239431	95270066	+	10495	0.776	0
Zfp295	ENSMUSG00000046962	chr16	98168599	98183786	-	6020	0.776	0
Znhit6	ENSMUSG00000074182	chr3	145239172	145268209	+	2813	0.776	0
Aatk	ENSMUSG00000025375	chr11	119868627	119908481	-	7159	0.775	0
Pak1ip1	ENSMUSG00000038683	chr13	41096392	41108388	+	4049	0.774	0
Lrrc16a	ENSMUSG00000021338	chr13	24104213	24372664	-	8989	0.774	0
1110028 C15Rik	ENSMUSG00000026004	chr1	66765817	66864169	-	6395	0.774	0
Qrsl1	ENSMUSG00000019863	chr10	43593994	43621551	-	3442	0.774	0
Ccdc64	ENSMUSG00000041609	chr5	116098184	116181630	-	5932	0.774	0
Actg1	ENSMUSG00000062825	chr11	120207004	120209856	-	2658	0.773	0
Zfyve1	ENSMUSG00000042628	chr12	84887891	84938097	-	3869	0.771	0
Rfk	ENSMUSG00000024712	chr19	17468533	17475839	+	2603	0.771	0
Ddx55	ENSMUSG00000029389	chr5	125002873	125019669	+	5121	0.771	0
Txnrd3	ENSMUSG00000000811	chr6	89593982	89625523	+	2836	0.769	0
Eif4a2	ENSMUSG00000022884	chr16	23107517	23114209	+	5338	0.768	0
Notch1	ENSMUSG00000026923	chr2	26313423	26372183	-	14232	0.767	0
Tnrc6c	ENSMUSG00000025571	chr11	117515603	117624753	+	12843	0.766	0

4930455F23Rik	ENSMUSG00000026578	chr1	166205716	166217978	+	1909	0.766	0
Tmem90b	ENSMUSG00000074736	chr2	149654947	149830128	+	2463	0.765	0
Isca1	ENSMUSG00000044792	chr13	59856770	59871171	-	4288	0.764	0
Entpd7	ENSMUSG00000025192	chr19	43764162	43808187	+	8963	0.763	0
Frat2	ENSMUSG00000047604	chr19	41920468	41922622	-	2155	0.763	0
Gm14325	ENSMUSG00000078863	chr2	177566496	177575041	-	1616	0.763	0
Mtrf1	ENSMUSG00000022022	chr14	79797579	79823457	+	1862	0.763	0
Mxra8	ENSMUSG00000029070	chr4	155213789	155218197	+	2918	0.763	0
Cyp4v3	ENSMUSG00000079057	chr8	46355025	46418570	-	6246	0.762	0
Ccdc34	ENSMUSG00000027160	chr2	109857974	110013517	+	6558	0.761	0
Apoc4	ENSMUSG00000074336	chr7	20263443	20266765	-	464	0.761	0
Gnai2	ENSMUSG00000032562	chr9	107516461	107537673	-	2185	0.761	0
Emcn	ENSMUSG00000054690	chr3	137004031	137095149	+	5409	0.76	0
Heatr2	ENSMUSG00000025857	chr5	139626177	139662464	+	3514	0.76	0
Papss1	ENSMUSG00000028032	chr3	131227732	131306634	+	2599	0.759	0
Chmp4c	ENSMUSG00000027536	chr3	10366907	10391005	+	1856	0.759	0
Gjb5	ENSMUSG00000042357	chr4	127032053	127035425	-	1721	0.759	0
Cdc16	ENSMUSG00000038416	chr8	13757676	13781938	+	3103	0.757	0
42986	ENSMUSG00000018398	chr11	53332759	53363067	+	6087	0.757	0
Riok1	ENSMUSG00000021428	chr13	38129109	38153298	+	2489	0.756	0
Stt3b	ENSMUSG00000032437	chr9	115151725	115219539	-	4195	0.756	0
Ccdc111	ENSMUSG00000038225	chr8	47660933	47702566	-	6309	0.756	0
Rab11a	ENSMUSG00000004771	chr9	64563106	64585565	-	2992	0.756	0
Uspl1	ENSMUSG00000041264	chr5	149995926	150027010	+	6648	0.756	0
Polr1a	ENSMUSG00000049553	chr6	71859047	71929350	+	6577	0.756	0
Paf1	ENSMUSG00000003437	chr7	29177970	29184407	+	3438	0.756	0
Usp48	ENSMUSG00000043411	chr4	137149670	137214452	+	9754	0.754	0
Esam	ENSMUSG00000001946	chr9	37335663	37345904	+	2867	0.753	0
Ssr1	ENSMUSG00000021427	chr13	38063270	38086059	-	9321	0.753	0
Pfas	ENSMUSG00000020899	chr11	68799199	68824788	-	7951	0.752	0
Kif1c	ENSMUSG00000020821	chr11	70514050	70545466	+	7053	0.752	0
Bag4	ENSMUSG00000037316	chr8	26875010	26895681	-	4798	0.751	0
1200014J11Rik	ENSMUSG00000020783	chr11	72861285	72893719	+	8318	0.751	0
Tm7sf3	ENSMUSG00000040234	chr6	146550874	146591346	-	3438	0.75	0
1700021F05Rik	ENSMUSG00000019797	chr10	43244927	43260800	-	1339	0.749	0
Ppp6c	ENSMUSG00000026753	chr2	39049874	39081971	-	4219	0.749	0
Csnk1d	ENSMUSG00000025162	chr11	120819779	120852644	-	5853	0.748	0
Ptrf	ENSMUSG00000004044	chr11	100818047	100832201	-	5223	0.747	0
Dcun1d2	ENSMUSG00000038506	chr8	13255963	13288126	-	3034	0.746	0

Rgag4	ENSMUSG00000049191	chrX	99261883	99266643	-	4761	0.746		0
Brd4	ENSMUSG00000024002	chr17	32333219	32421667	-	8402	0.746		0
Eny2	ENSMUSG00000022338	chr15	44259657	44269231	+	2429	0.745		0
Ppm1a	ENSMUSG00000021096	chr12	73862198	73895927	+	5400	0.745		0
Ube2d3	ENSMUSG00000078578	chr3	135101261	135130142	+	2675	0.744		0
Ramp3	ENSMUSG00000041046	chr11	6558524	6577478	+	1380	0.744		0
Snrpd1	ENSMUSG00000002477	chr18	10617794	10628228	+	837	0.743		0
2310035C23Rik	ENSMUSG00000026319	chr1	107560438	107651708	+	6805	0.743		0
Gpc6	ENSMUSG00000058571	chr14	117324519	118378751	+	9428	0.742		0
Irak4	ENSMUSG00000059883	chr15	94374074	94412246	+	5032	0.742		0
Med1	ENSMUSG00000018160	chr11	98013468	98054607	-	8753	0.741		0
Usp40	ENSMUSG00000005501	chr1	89841696	89905126	-	5316	0.741		0
Myo1d	ENSMUSG00000035441	chr11	80295628	80593527	-	6465	0.741		0
Rab22a	ENSMUSG00000027519	chr2	173485261	173532844	+	7206	0.74		0
Cenph	ENSMUSG00000045273	chr13	101529629	101545854	-	1784	0.74		0
Fzd8	ENSMUSG00000036904	chr18	9212854	9216199	+	3346	0.74		0
Sfxn1	ENSMUSG00000021474	chr13	54167214	54203715	+	2811	0.74		0
Eid2	ENSMUSG00000046058	chr7	29052900	29054184	+	1285	0.739		0
Neurl1b	ENSMUSG00000034413	chr17	26551910	26583283	+	6184	0.738		0
Mapkapk5	ENSMUSG00000029454	chr5	121968622	121995914	-	6209	0.737		0
Brpf3	ENSMUSG00000063952	chr17	28938035	28975491	+	7194	0.737		0
Hnrnpab	ENSMUSG00000020358	chr11	51412372	51420349	-	3004	0.736		0
Rqcd1	ENSMUSG00000026174	chr1	74552632	74577418	+	3828	0.733		0
Zfp592	ENSMUSG00000005621	chr7	88138567	88190050	+	7414	0.733		0
Clk2	ENSMUSG00000068917	chr3	88968717	88980843	+	5299	0.733		0
Dync2h1	ENSMUSG00000047193	chr9	6928503	7184446	-	16161	0.732		0
S100a16	ENSMUSG00000074457	chr3	90341176	90347073	+	1617	0.732		0
Heatr6	ENSMUSG00000000976	chr11	83567198	83597256	+	6419	0.732		0
Ralgds	ENSMUSG00000026821	chr2	28368645	28408601	+	6159	0.731		0
Sh3tc1	ENSMUSG00000036553	chr5	36039829	36071925	-	5715	0.731		0
Senp8	ENSMUSG00000051705	chr9	59582066	59598456	-	3792	0.731		0
Tnks1bp1	ENSMUSG00000033955	chr2	84888179	84913205	+	6184	0.73		0
Tatdn1	ENSMUSG00000050891	chr15	58721708	58765285	-	1463	0.73		0
Masp1	ENSMUSG00000022887	chr16	23449490	23520676	-	6017	0.729		0
Ushbp1	ENSMUSG00000034911	chr8	73908173	73919700	-	3549	0.729		0
Ttpal	ENSMUSG00000017679	chr2	163428050	163444749	+	5951	0.728		0
Adcy4	ENSMUSG00000022220	chr14	56387895	56402879	-	3500	0.727		0
Ccl4	ENSMUSG00000018930	chr11	83476086	83478185	+	660	0.726		0
Terf2ip	ENSMUSG00000033430	chr8	114535259	114544428	+	3599	0.726		0
Gpc4	ENSMUSG00000031119	chrX	49406198	49518429	-	2651	0.726		0
Msl3	ENSMUSG00000031358	chrX	165092049	165111830	-	2885	0.725		0

Prkrir	ENSMUSG00000030753	chr7	105851613	105866572	+	4125	0.724	0
Tle3	ENSMUSG00000032280	chr9	61220190	61266297	+	11629	0.722	0
Tti1	ENSMUSG00000027650	chr2	157807539	157854169	-	4129	0.722	0
Mapkap 1	ENSMUSG00000038696	chr2	34262291	34480470	+	4061	0.721	0
Loxl1	ENSMUSG00000032334	chr9	58135530	58161019	-	3338	0.721	0
Mudeng	ENSMUSG00000036291	chr14	49686170	49707398	+	3165	0.719	0
Ctsh	ENSMUSG00000032359	chr9	89948990	89970927	+	3452	0.718	0
Ncbp2	ENSMUSG00000022774	chr16	31948599	31961867	+	2305	0.718	0
Jam3	ENSMUSG00000031990	chr9	26904969	26963003	-	2537	0.717	0
Xpnpep 3	ENSMUSG00000022401	chr15	81230568	81287237	+	6117	0.716	0
Gtf3c2	ENSMUSG00000029144	chr5	31453515	31482517	-	7033	0.716	0
Spg20	ENSMUSG00000036580	chr3	54916030	54941244	+	5362	0.715	0
Cd22	ENSMUSG00000030577	chr7	31650421	31665361	-	4316	0.714	0
Pttg1ip	ENSMUSG00000009291	chr10	77044465	77061477	+	3426	0.714	0
Lrrk1	ENSMUSG00000015133	chr7	73371798	73533236	-	9505	0.714	0
Birc3	ENSMUSG00000032000	chr9	7848699	7873186	-	5009	0.713	0
Armcx1	ENSMUSG00000033460	chrX	131252502	131256456	+	3227	0.713	0
Lat2	ENSMUSG00000040751	chr5	135076138	135090893	-	1505	0.713	0
Lta4h	ENSMUSG00000015889	chr10	92916141	92947641	+	2066	0.713	0
Golga5	ENSMUSG00000021192	chr12	103708120	103736112	+	2667	0.713	0
App	ENSMUSG00000022892	chr16	84954685	85173952	-	3359	0.713	0
Traip	ENSMUSG00000032586	chr9	107853294	107874599	+	2697	0.712	0
Slc7a1	ENSMUSG00000041313	chr5	149138986	149211480	-	7527	0.712	0
Zfp110	ENSMUSG00000058638	chr7	13420110	13435926	+	4116	0.711	0
Cfl1	ENSMUSG00000056201	chr19	5490485	5494029	+	1148	0.711	0
Tmcc1	ENSMUSG00000030126	chr6	115968636	116143504	-	7252	0.711	0
Sp4	ENSMUSG00000025323	chr12	119473406	119539913	-	5770	0.711	0
1190005 F20Rik	ENSMUSG00000053286	chr1	153275778	153305312	+	2664	0.71	0
Rgs3	ENSMUSG00000059810	chr4	62220881	62364052	+	9818	0.71	0
Mettl16	ENSMUSG00000010554	chr11	74584332	74642027	+	13817	0.71	0
Zfp608	ENSMUSG00000052713	chr18	55047699	55152245	-	8095	0.709	0
Pxmp3	ENSMUSG00000040374	chr3	5560188	5576239	-	2270	0.709	0
Wbp11	ENSMUSG00000030216	chr6	136762175	136776754	-	5029	0.708	0
Rrad	ENSMUSG00000031880	chr8	107151966	107155221	-	1575	0.708	0
Srrm2	ENSMUSG00000039218	chr17	23940154	23961706	+	8867	0.708	0
Gas8	ENSMUSG00000040220	chr8	126042839	126060422	+	1448	0.707	0
C2cd3	ENSMUSG00000047248	chr7	107520743	107618668	+	8941	0.707	0
Ier5l	ENSMUSG00000089762	chr2	30328168	30329719	-	1552	0.705	0
Icmt	ENSMUSG00000039662	chr4	151671336	151681230	+	5287	0.705	0
2510009 E07Rik	ENSMUSG00000043391	chr16	21649118	21694738	-	5437	0.705	0
Dvl3	ENSMUSG00000003233	chr16	20517055	20532593	+	4899	0.704	0
Mknk1	ENSMUSG00000028708	chr4	115511803	115551855	+	4502	0.703	0

Immt	ENSMUSG00000052337	chr6	71781325	71827382	+	5104	0.702		0
Pld2	ENSMUSG00000020828	chr11	70353566	70371612	+	6168	0.702		0
Jkamp	ENSMUSG00000005078	chr12	73186576	73202448	+	2692	0.702		0
Agtr1a	ENSMUSG00000049115	chr13	30428310	30474736	+	2467	0.701		0
Eif2s2	ENSMUSG00000074656	chr2	154697146	154718671	-	6755	0.701		0
Ncf4	ENSMUSG00000071715	chr15	78075231	78093010	+	2021	0.701		0
Apoe	ENSMUSG00000002985	chr7	20281458	20284515	-	1980	0.7		0
Aplp1	ENSMUSG00000006651	chr7	31220001	31230580	-	2369	0.699		0
Cd209d	ENSMUSG00000031495	chr8	3871827	3878555	-	916	0.699		0
Fam151 b	ENSMUSG00000034334	chr13	93219577	93253986	-	1381	0.699		0
Brpf1	ENSMUSG00000001632	chr6	113257175	113274851	+	6732	0.699		0
Mrgpre	ENSMUSG00000048965	chr7	150964270	150970405	-	3759	0.699		0
Jun	ENSMUSG00000052684	chr4	94715725	94718913	-	3189	0.698		0
Pxn	ENSMUSG00000029528	chr5	115956685	116005996	+	8195	0.698		0
Mlh3	ENSMUSG00000021245	chr12	86575479	86611541	-	5542	0.698		0
Sorbs1	ENSMUSG00000025006	chr19	40369539	40588269	-	4919	0.697		0
Foxo1	ENSMUSG00000044167	chr3	52072259	52154031	+	5552	0.696		0
2500003 M10Rik	ENSMUSG00000001017	chr3	90302878	90313420	-	3637	0.693		0
Fry	ENSMUSG00000056602	chr5	150921242	151300327	+	14213	0.692		0
Kat2b	ENSMUSG00000000708	chr17	53706186	53812045	+	4867	0.69		0
Dynll1	ENSMUSG00000009013	chr5	115747119	115751008	-	2926	0.69		0
2010321 M09Rik	ENSMUSG00000034263	chr9	64808639	64834781	+	3208	0.688		0
Med30	ENSMUSG00000038622	chr15	52544000	52561986	+	871	0.688		0
Ube2f	ENSMUSG00000034343	chr1	93146888	93185454	+	4624	0.687		0
Gpbp1l1	ENSMUSG00000034042	chr4	116230263	116266505	+	4176	0.687		0
Slc16a9	ENSMUSG00000037762	chr10	69707848	69748716	+	3869	0.686		0
Csnk2a 2	ENSMUSG00000046707	chr8	97969996	98012727	-	3773	0.686		0
L2hgdh	ENSMUSG00000020988	chr12	70791423	70825861	-	3338	0.685		0
Tirap	ENSMUSG00000032041	chr9	34992136	35007876	-	4929	0.685		0
Col16a1	ENSMUSG00000040690	chr4	129725084	129776527	+	6750	0.685		0
Wdr59	ENSMUSG00000031959	chr8	113972684	114046007	-	5222	0.685		0
Armc1	ENSMUSG00000027599	chr3	19032146	19063065	-	2929	0.684		0
Nqo2	ENSMUSG00000046949	chr13	34056528	34080334	+	3974	0.683		0
Cux1	ENSMUSG00000029705	chr5	136724005	137043360	-	13482	0.683		0
Cryzl1	ENSMUSG00000058240	chr16	91689567	91729220	-	4353	0.683		0
2610035 D17Rik	ENSMUSG00000087259	chr11	112905209	113063152	-	2121	0.683		0
Psme3	ENSMUSG00000078652	chr11	101177527	101184851	+	2949	0.682		0

Rasl11a	ENSMUSG00000029641	chr5	147656647	147659302	+	1126	0.682	0
Tada2b	ENSMUSG00000029196	chr5	36816319	36826934	-	3791	0.682	0
Mdfi	ENSMUSG00000032717	chr17	47952277	47971640	-	3092	0.682	0
Atic	ENSMUSG00000026192	chr1	71603724	71626205	+	5512	0.681	0
Zfp869	ENSMUSG00000054648	chr8	72230231	72234623	-	1590	0.68	0
Mkl2	ENSMUSG00000009569	chr16	13256574	13417622	+	8990	0.679	0
Utp3	ENSMUSG00000070697	chr5	88983508	88985108	+	1601	0.678	0
Cdc42ep3	ENSMUSG00000036533	chr17	79733365	79754431	-	1967	0.678	0
Dnajc9	ENSMUSG00000021811	chr14	21203860	21208132	-	1631	0.678	0
Slc25a4	ENSMUSG00000031633	chr8	47292151	47296638	-	2353	0.677	0
Cenpt	ENSMUSG00000036672	chr8	108368578	108375908	-	1767	0.677	0
Hyal1	ENSMUSG00000010051	chr9	107479258	107485174	+	3804	0.677	0
Spcs3	ENSMUSG00000054408	chr8	55605787	55615351	-	3467	0.676	0
Crip1	ENSMUSG00000006360	chr12	114390223	114392088	+	445	0.675	0
Hist2h2be	ENSMUSG00000068854	chr3	96025044	96027661	+	2618	0.674	0
Hgsnat	ENSMUSG00000037260	chr8	27054931	27087216	-	2679	0.674	0
Psmd10	ENSMUSG00000031429	chrX	137482968	137491267	-	1469	0.674	0
Ccdc136	ENSMUSG00000029769	chr6	29346309	29376955	+	4153	0.673	0
Actn4	ENSMUSG00000054808	chr7	29678267	29747359	-	5026	0.673	0
Zfp939	ENSMUSG00000030424	chr7	46728732	46732703	+	927	0.673	0
C80913	ENSMUSG00000030421	chr7	38745012	38804571	-	3450	0.673	0
Tmem128	ENSMUSG00000067365	chr5	38651424	38660861	+	1942	0.673	0
Tacc2	ENSMUSG00000030852	chr7	137720998	137908298	+	9745	0.672	0
Ubr7	ENSMUSG00000041712	chr12	103996177	104015911	+	3255	0.672	0
Gclc	ENSMUSG00000032350	chr9	77602342	77642292	+	3423	0.67	0
C330019G07Rik	ENSMUSG00000054280	chr5	33131867	33196905	-	11004	0.67	0
Extl3	ENSMUSG00000021978	chr14	65670897	65716943	-	5977	0.67	0
Rab28	ENSMUSG00000029128	chr5	42016215	42099394	-	1767	0.669	0
Sike1	ENSMUSG00000027854	chr3	102799651	102807837	+	4627	0.669	0
Rab33b	ENSMUSG00000027739	chr3	51287888	51299827	+	3165	0.669	0
Ndufab1	ENSMUSG00000030869	chr7	129228917	129245400	-	6883	0.668	0
Mnt	ENSMUSG00000000282	chr11	74644422	74659227	+	4627	0.664	0
Srpk2	ENSMUSG00000062604	chr5	23009174	23182266	-	6737	0.663	0
Prss23	ENSMUSG00000039405	chr7	96656295	96666096	-	3226	0.663	0
Jarid2	ENSMUSG00000038518	chr13	44824843	45017012	+	9715	0.662	0
Hmha1	ENSMUSG00000035697	chr10	79479398	79494217	+	4478	0.661	0
Tcea1	ENSMUSG00000033813	chr1	4847775	4887987	+	2844	0.661	0
Gjb3	ENSMUSG00000042367	chr4	127002479	127008088	-	1966	0.66	0
Ighv3-6	ENSMUSG00000076672	chr12	115526363	115526793	-	350	0.656	0

Fam18b	ENSMUSG00000014177	chr11	62692957	62730282	+	2235	0.655	0
Serac1	ENSMUSG00000015659	chr17	6042196	6079741	-	4576	0.655	0
Zfand3	ENSMUSG00000044477	chr17	30142032	30346964	+	2682	0.655	0
Taok3	ENSMUSG00000061288	chr5	117570138	117725228	+	10952	0.654	0
Nsmaf	ENSMUSG00000028245	chr4	6323354	6381418	-	8372	0.654	0
Ap2b1	ENSMUSG00000035152	chr11	83112526	83218537	+	8755	0.654	0
Khsrp	ENSMUSG00000007670	chr17	57160481	57170930	-	3990	0.653	0
Sco1	ENSMUSG00000069844	chr11	66866172	66880572	+	3925	0.653	0
Plekhm3	ENSMUSG00000051344	chr1	64832557	65003398	-	10826	0.652	0
Espl1	ENSMUSG00000058290	chr15	102126724	102154787	+	6630	0.651	0
Ube2d2	ENSMUSG00000091896	chr18	35931213	35966826	+	2483	0.65	0
Rell1	ENSMUSG00000047881	chr5	64300135	64360144	-	3863	0.649	0
Hira	ENSMUSG00000022702	chr16	18877130	18970402	+	5277	0.647	0
Arsb	ENSMUSG00000042082	chr13	94541634	94712971	+	4298	0.647	0
Samd14	ENSMUSG00000047181	chr11	94871193	94887401	+	3379	0.647	0
S1pr2	ENSMUSG00000043895	chr9	20770396	20781237	-	2815	0.646	0
Zc3h11a	ENSMUSG00000026464	chr1	135516448	135557957	-	4395	0.646	0
AB124611	ENSMUSG00000057191	chr9	21330620	21349777	+	1447	0.645	0
mt-Nd2	ENSMUSG00000064345	chrM	3914	4951	+	1038	0.645	0
Crot	ENSMUSG00000003623	chr5	8966033	8997324	-	5456	0.645	0
Rnf168	ENSMUSG00000014074	chr16	32277545	32301520	+	4462	0.645	0
Sema3g	ENSMUSG00000021904	chr14	32031046	32043536	+	4538	0.644	0
Mcf2l	ENSMUSG00000031442	chr8	12873806	13020905	+	12761	0.644	0
Gm16104	ENSMUSG00000085327	chr10	80872320	80874324	+	401	0.644	0
Ube2o	ENSMUSG00000020802	chr11	116399054	116442761	-	5288	0.644	0
Hk3	ENSMUSG00000025877	chr13	55107346	55122746	-	3682	0.644	0
Plekhn1	ENSMUSG00000078485	chr4	155595565	155602651	-	2210	0.643	0
Fam164a	ENSMUSG00000043542	chr3	7503426	7553848	+	3361	0.642	0
Med4	ENSMUSG00000022109	chr14	73909856	73918352	+	1304	0.642	0
Exoc3l	ENSMUSG00000043251	chr8	107813824	107819998	-	2497	0.641	0
Fyco1	ENSMUSG00000025241	chr9	123698628	123761020	-	8061	0.64	0
Smad1	ENSMUSG00000031681	chr8	81862294	81923417	-	5814	0.64	0
Prkag2	ENSMUSG00000028944	chr5	24368562	24606460	-	6480	0.64	0
Zfp963	ENSMUSG00000092260	chr8	72265538	72273869	-	2299	0.639	0
Trem2	ENSMUSG00000023992	chr17	48485726	48493472	+	4946	0.639	0
1200011M11Rik	ENSMUSG00000020495	chr11	86891234	86900276	-	3218	0.638	0
Zfp507	ENSMUSG00000044452	chr7	36557365	36588008	-	7118	0.637	0

5730480H06Rik	ENSMUSG00000093459	chr5	48786812	48805533	+	1735	0.636	0
Slc30a5	ENSMUSG00000021629	chr13	101572604	101603382	-	3178	0.636	0
Tspan2	ENSMUSG00000027858	chr3	102538452	102576215	+	4585	0.636	0
Ptprn2	ENSMUSG00000056553	chr12	117724193	118516640	+	5210	0.636	0
Ints10	ENSMUSG00000031864	chr8	71317828	71353309	+	6706	0.636	0
Ormdl1	ENSMUSG00000026097	chr1	53353939	53367153	+	4711	0.634	0
Coil	ENSMUSG00000033983	chr11	88831566	88852927	+	3520	0.634	0
Apol9a	ENSMUSG00000068246	chr15	77234220	77241483	-	1545	0.633	0
Setd1b	ENSMUSG00000038384	chr5	123592202	123617444	+	10874	0.632	0
Exoc3l2	ENSMUSG00000011263	chr7	20074405	20082109	+	2201	0.632	0
Rpl23a	ENSMUSG00000058546	chr11	77994434	77997086	-	847	0.631	0
Dazap2	ENSMUSG00000000346	chr15	100446069	100451184	+	1838	0.631	0
A930001N09Rik	ENSMUSG00000048249	chr17	26852595	26913580	+	8564	0.63	0
Hoxb4	ENSMUSG00000038692	chr11	96177998	96182952	+	4149	0.629	0
Zfp161	ENSMUSG00000049672	chr17	69732390	69740090	+	4231	0.629	0
Ly6a	ENSMUSG00000075602	chr15	74825308	74828064	-	819	0.628	0
6330578E17Rik	ENSMUSG00000026112	chr1	37473929	37486948	-	3863	0.628	0
6030419C18Rik	ENSMUSG00000091354	chr9	58336770	58337735	+	537	0.628	0
9030425E11Rik	ENSMUSG00000032024	chr9	40494045	40593402	+	4591	0.627	0
Sema3f	ENSMUSG00000034684	chr9	107583833	107612806	-	3486	0.626	0
Cox7b	ENSMUSG00000031231	chrX	103211039	103217789	+	1214	0.625	0
Gpr64	ENSMUSG00000031298	chrX	156828622	156936002	+	5278	0.625	0
Fancf	ENSMUSG00000092118	chr7	59115947	59117637	-	1691	0.624	0
Mbd1	ENSMUSG00000024561	chr18	74427926	74442339	+	2897	0.623	0
Supt7l	ENSMUSG00000053134	chr5	31816942	31830180	-	5347	0.621	0
Pcdh1	ENSMUSG00000051375	chr18	38345569	38371707	-	8454	0.621	0
D6Wsu163e	ENSMUSG00000030347	chr6	126889984	126925722	+	2238	0.62	0
Senp2	ENSMUSG00000022855	chr16	22009557	22049342	+	4518	0.62	0
Mbnl1	ENSMUSG00000027763	chr3	60277032	60433670	+	5644	0.619	0
Ints7	ENSMUSG00000037461	chr1	193399642	193446551	+	4204	0.617	0
Srfbp1	ENSMUSG00000024528	chr18	52625347	52650392	+	1463	0.616	0
Vps37b	ENSMUSG00000066278	chr5	124454650	124482269	-	2512	0.616	0
Pdcd11	ENSMUSG00000025047	chr19	47165256	47205633	+	6041	0.615	0
Pik3cb	ENSMUSG00000032462	chr9	98938821	99041040	-	5445	0.615	0
Mmp11	ENSMUSG00000000901	chr10	75385967	75400678	-	2645	0.615	0
Tsr1	ENSMUSG00000038335	chr11	74711573	74722844	+	3837	0.615	0

Ifit3	ENSMUSG00000074896	chr19	34658021	34663221	+	1745	0.613	0
2210404 J11Rik	ENSMUSG00000036552	chr17	15178605	15201196	+	4291	0.613	0
Vps8	ENSMUSG00000033653	chr16	21423191	21644753	+	8672	0.613	0
Csl	ENSMUSG00000046934	chr10	99220339	99222292	-	1954	0.612	0
Yeats2	ENSMUSG00000041215	chr16	20141136	20232646	+	6116	0.611	0
Snrnp70	ENSMUSG00000063511	chr7	52631824	52651062	-	1710	0.61	0
Khnyn	ENSMUSG00000047153	chr14	56503806	56515618	+	3876	0.609	0
Nme7	ENSMUSG00000026575	chr1	166243010	166334805	+	1217	0.608	0
Sbk1	ENSMUSG00000042978	chr7	133416133	133438513	+	4092	0.607	0
Gm1049 7	ENSMUSG00000073394	chr17	44872794	44874561	+	792	0.606	0
Scgb3a 2	ENSMUSG00000038791	chr18	43923979	43927047	+	838	0.604	0
D43004 2O09Rik	ENSMUSG00000032743	chr7	132851390	133018311	+	7342	0.604	0
C13002 2K22Rik	ENSMUSG00000034083	chr6	91828047	91849842	+	9193	0.604	0
Lsm11	ENSMUSG00000044847	chr11	45741771	45758437	-	6453	0.603	0
Rabl3	ENSMUSG00000022827	chr16	37539971	37572471	+	6369	0.603	0
3110056 K07Rik	ENSMUSG00000085622	chr12	72092602	72116819	-	1504	0.602	0
Htra1	ENSMUSG00000006205	chr7	138079625	138129174	+	2879	0.602	0
Cmas	ENSMUSG00000030282	chr6	142705206	142724234	+	2783	0.602	0
Faf2	ENSMUSG00000025873	chr13	54723145	54765429	+	5084	0.601	0
Cdca3	ENSMUSG00000023505	chr6	124779565	124783719	+	2866	0.6	0
Dpy19l3	ENSMUSG00000043671	chr7	36470184	36539473	-	11173	0.6	0
Map3k3	ENSMUSG00000020700	chr11	105945927	106016760	+	4023	0.6	0
Sae1	ENSMUSG00000052833	chr7	16905583	16973146	-	2531	0.599	0
Pex7	ENSMUSG00000020003	chr10	19579735	19627495	-	2351	0.599	0
Junb	ENSMUSG00000052837	chr8	87500811	87502617	-	1807	0.599	0
Sfswap	ENSMUSG00000029439	chr5	130007106	130077258	+	3968	0.598	0
Clp1	ENSMUSG00000027079	chr2	84562261	84567507	-	2672	0.597	0
Gbp2	ENSMUSG00000028270	chr3	142283566	142300972	+	2516	0.597	0
Efemp2	ENSMUSG00000024909	chr19	5473973	5481853	+	2648	0.597	0
Xaf1	ENSMUSG00000040483	chr11	72115131	72127235	+	4689	0.597	0
Mfap2	ENSMUSG00000060572	chr4	140566333	140571899	+	1211	0.596	0
Twistnb	ENSMUSG00000020561	chr12	34114489	34124245	+	2324	0.595	0
Cdk3-ps	ENSMUSG00000092300	chr11	116077317	116081601	+	1548	0.595	0
Cpped1	ENSMUSG00000065979	chr16	11803814	11909538	-	5205	0.594	0
Narf	ENSMUSG00000000056	chr11	121098567	121117170	+	4806	0.593	0
Fggy	ENSMUSG00000028573	chr4	95224198	95593630	+	5844	0.592	0
Phlpp2	ENSMUSG00000031732	chr8	112392442	112468571	+	8449	0.591	0

Copa	ENSMUSG00000026553	chr1	174012660	174052461	+	9435	0.59	0
Mmadhc	ENSMUSG00000026766	chr2	50135401	50152321	-	2012	0.59	0
Rap1a	ENSMUSG00000068798	chr3	105530185	105604248	-	2401	0.59	0
Carm1	ENSMUSG00000032185	chr9	21351338	21393931	+	4324	0.589	0
Snx10	ENSMUSG00000038301	chr6	51473900	51540678	+	4911	0.589	0
Phf15	ENSMUSG00000020387	chr11	51626957	51671155	-	10823	0.588	0
Sptlc1	ENSMUSG00000021468	chr13	53428085	53472734	-	2622	0.587	0
Ralgps1	ENSMUSG00000038831	chr2	32988941	33227006	-	7653	0.587	0
Surf6	ENSMUSG00000036160	chr2	26744148	26758399	-	4071	0.587	0
Oasl2	ENSMUSG00000029561	chr5	115346943	115362243	+	3881	0.586	0
Nudt16	ENSMUSG00000032565	chr9	105031668	105034135	-	1606	0.585	0
Snhg1	ENSMUSG00000090867	chr19	8797986	8800932	+	2947	0.584	0
A430107O13Rik	ENSMUSG00000062980	chr6	21935916	22206404	+	8279	0.584	0
Txnl1	ENSMUSG00000024583	chr18	63822455	63852013	-	2558	0.584	0
Tmem198b	ENSMUSG00000047090	chr10	128237092	128241426	-	3363	0.584	0
Gm7935	ENSMUSG00000075609	chr15	73910438	73911986	-	1549	0.583	0
Fam185a	ENSMUSG00000047221	chr5	20930776	20987942	+	3894	0.583	0
Dnajb14	ENSMUSG00000074212	chr3	137530639	137579521	+	12553	0.583	0
Mpzl2	ENSMUSG00000032092	chr9	44850508	44862098	+	3107	0.583	0
Mrps18c	ENSMUSG00000016833	chr5	101227646	101233490	+	1076	0.582	0
Kctd6	ENSMUSG00000021752	chr14	9046662	9056081	+	3253	0.582	0
Gm5263	ENSMUSG00000090323	chr1	148267564	148268004	+	363	0.582	0
Igkc	ENSMUSG00000076609	chr6	70676429	70676751	+	323	0.582	0
Ptplad1	ENSMUSG00000033629	chr9	64834792	64869524	-	2731	0.581	0
5033414D02Rik	ENSMUSG00000016495	chr19	29423089	29441880	-	3799	0.581	0
B230354K17Rik	ENSMUSG00000073393	chr17	45570801	45579493	+	4897	0.581	0
Arfgef2	ENSMUSG00000074582	chr2	166631088	166723552	+	8770	0.581	0
Cplx2	ENSMUSG00000025867	chr13	54472713	54485278	+	4925	0.58	0
Kcnd1	ENSMUSG00000009731	chrX	7399415	7415406	+	5287	0.58	0
Dchs1	ENSMUSG00000036862	chr7	112901503	112936168	-	11070	0.58	0
Reep3	ENSMUSG00000019873	chr10	66474256	66559605	-	3023	0.579	0
Kdm5a	ENSMUSG00000030180	chr6	120314117	120394592	+	14185	0.579	0
Pdia3	ENSMUSG00000027248	chr2	121239511	121264423	+	3139	0.579	0
Exd2	ENSMUSG00000032705	chr12	81564082	81599122	+	3323	0.578	0
Dgat2	ENSMUSG00000030747	chr7	106302173	106331223	-	2251	0.577	0
Rbm34	ENSMUSG00000033931	chr8	129471075	129494979	-	3429	0.576	0

Sez6l2	ENSMUSG00000030683	chr7	134085658	134114120	+	4619	0.575	0
Gns	ENSMUSG00000034707	chr10	120802146	120834301	+	3855	0.575	0
Ints4	ENSMUSG00000025133	chr7	104629466	104689907	+	3243	0.574	0
Gm15800	ENSMUSG00000042744	chr5	121670228	121818586	+	16416	0.573	0
Dok2	ENSMUSG00000022102	chr14	71165843	71178302	+	2902	0.572	0
Shb	ENSMUSG00000044813	chr4	45436150	45545342	-	3809	0.571	0
Tie1	ENSMUSG00000033191	chr4	118143796	118162666	-	4917	0.569	0
42983	ENSMUSG00000072214	chr16	18621904	18630004	-	2136	0.569	0
Arl8a	ENSMUSG00000026426	chr1	137043401	137052846	+	2299	0.568	0
Eif4g1	ENSMUSG00000045983	chr16	20668386	20692957	+	7227	0.567	0
Hnrnpc	ENSMUSG00000060373	chr14	52693055	52723703	-	2843	0.567	0
Glb1	ENSMUSG00000079260	chr9	114310223	114316394	+	2921	0.566	0
Eif4a1	ENSMUSG00000059796	chr11	69480438	69485925	-	4334	0.564	0
Mrfap1	ENSMUSG00000055302	chr5	37186106	37188011	-	1648	0.563	0
Emilin1	ENSMUSG00000029163	chr5	31215775	31223651	+	4938	0.563	0
Zbtb49	ENSMUSG00000029127	chr5	38580974	38611696	-	7750	0.563	0
Wee1	ENSMUSG00000031016	chr7	117265864	117285626	+	1941	0.561	0
Hibch	ENSMUSG00000041426	chr1	52901857	52977830	+	2426	0.56	0
1600027N09Rik	ENSMUSG00000027569	chr2	180316009	180321009	+	3499	0.559	0
Reps1	ENSMUSG00000019854	chr10	17775667	17844961	+	5301	0.559	0
Evc2	ENSMUSG00000050248	chr5	37729717	37816294	+	5858	0.558	0
Gm12258	ENSMUSG00000072915	chr11	58672935	58675458	+	2524	0.558	0
Ppm1j	ENSMUSG00000002228	chr3	104583974	104588936	+	1721	0.558	0
Rdh14	ENSMUSG00000020621	chr12	10397586	10402368	+	1472	0.557	0
Ubxn4	ENSMUSG00000026353	chr1	130140758	130175954	+	4093	0.557	0
Nr2c1	ENSMUSG00000005897	chr10	93610768	93659956	+	4607	0.557	0
2310044G17Rik	ENSMUSG00000034157	chr12	88288364	88306312	+	4027	0.556	0
Gpr124	ENSMUSG00000031486	chr8	28196313	28233890	+	5499	0.556	0
Zfp438	ENSMUSG00000050945	chr18	5210029	5334437	-	3209	0.556	0
Copg2	ENSMUSG00000025607	chr6	30697554	30846794	-	4477	0.556	0
Dlg4	ENSMUSG00000020886	chr11	69830587	69859034	+	4657	0.555	0
Zfyve9	ENSMUSG00000034557	chr4	108310071	108453403	-	6459	0.554	0
Smug1	ENSMUSG00000036061	chr15	102983738	102988383	-	3231	0.554	0
Cnnm2	ENSMUSG00000064105	chr19	46836086	46953285	+	3558	0.552	0
Fam73a	ENSMUSG00000054942	chr3	151936423	152003371	-	5355	0.552	0
Numbl	ENSMUSG00000063160	chr7	28043452	28067163	+	8673	0.552	0
Tbccd1	ENSMUSG00000004462	chr16	22813288	22857642	-	2702	0.552	0
Sppl3	ENSMUSG00000029550	chr5	115461146	115548799	+	4240	0.55	0
Prorsd1	ENSMUSG00000032673	chr11	29411757	29415033	-	2128	0.55	0
Ptbp1	ENSMUSG00000006498	chr10	79317172	79327516	+	4748	0.549	0
Rtp4	ENSMUSG00000033355	chr16	23610005	23614308	+	1573	0.549	0

Srpk1	ENSMUSG00000004865	chr17	28724593	28759466	-	9647	0.548	0
Ipo8	ENSMUSG00000040029	chr6	148719205	148779989	-	10626	0.547	0
Gm3222	ENSMUSG00000079311	chr17	14846073	14847062	+	990	0.547	0
Mfng	ENSMUSG00000018169	chr15	78586312	78603905	-	2839	0.547	0
C77080	ENSMUSG00000050390	chr4	128896822	128938648	-	5770	0.546	0
Yipf4	ENSMUSG00000024072	chr17	74888854	74899617	+	2320	0.545	0
Cenpw	ENSMUSG00000075266	chr10	29915817	29920346	-	1510	0.545	0
Stt3a	ENSMUSG00000032116	chr9	36538860	36575264	-	7017	0.545	0
Ide	ENSMUSG00000056999	chr19	37343233	37412342	-	5823	0.542	0
Ubtf	ENSMUSG00000020923	chr11	102165874	102181056	-	6552	0.541	0
Ing1	ENSMUSG00000045969	chr8	11556066	11563250	+	2944	0.541	0
Srek1ip 1	ENSMUSG00000021716	chr13	105582564	105629354	+	3254	0.541	0
2900076 A07Rik	ENSMUSG00000086033	chr7	88673678	88676365	+	701	0.541	0
Slc37a3	ENSMUSG00000029924	chr6	39284769	39327706	-	4065	0.54	0
Phf13	ENSMUSG00000047777	chr4	151363742	151370367	-	3205	0.54	0
Lrrcc1	ENSMUSG00000027550	chr3	14533788	14572658	+	7492	0.54	0
Sox7	ENSMUSG00000063060	chr14	64562511	64569569	+	3298	0.54	0
Arhgap2 7	ENSMUSG00000034255	chr11	103192811	103225006	-	6417	0.539	0
Cdk7	ENSMUSG00000069089	chr13	101466982	101500897	-	8386	0.539	0
H3f3a	ENSMUSG00000060743	chr1	182730963	182744074	-	3504	0.539	0
Klhdc1	ENSMUSG00000051890	chr12	70342163	70385619	+	3137	0.537	0
Hbs1l	ENSMUSG00000019977	chr10	21015785	21088695	+	4893	0.537	0
Plvap	ENSMUSG00000034845	chr8	74021653	74035668	-	2015	0.537	0
Bag2	ENSMUSG00000042215	chr1	33802329	33814639	-	2252	0.534	0
Prkch	ENSMUSG00000021108	chr12	74685783	74879172	+	4617	0.533	0
Zcchc8	ENSMUSG00000029427	chr5	124148311	124171053	-	4286	0.533	0
Rabif	ENSMUSG00000042229	chr1	136391225	136406451	+	3961	0.532	0
Map2k5	ENSMUSG00000058444	chr9	63011576	63225659	-	2310	0.529	0
Dera	ENSMUSG00000030225	chr6	137703063	137786410	+	1740	0.529	0
Cd63	ENSMUSG00000025351	chr10	128345975	128349872	+	1031	0.529	0
Rbm15b	ENSMUSG00000045365	chr9	106786316	106789331	-	3016	0.529	0
Slco2b1	ENSMUSG00000030737	chr7	106806314	106859850	-	5612	0.528	0
Olfml3	ENSMUSG00000027848	chr3	103537623	103541924	-	4014	0.528	0
5031439 G07Rik	ENSMUSG00000036046	chr15	84774366	84818981	-	9604	0.528	0
Tceb3	ENSMUSG00000028668	chr4	135559283	135577678	-	4708	0.528	0
Zc3h18	ENSMUSG00000017478	chr8	124900509	124941260	+	9190	0.528	0
Chchd3	ENSMUSG00000053768	chr6	32740976	33010260	-	2926	0.525	0
Klhl12	ENSMUSG00000026455	chr1	136352108	136387595	+	6555	0.524	0
Hdac8	ENSMUSG00000067567	chrX	99479978	99700698	-	4839	0.524	0

Polr2m	ENSMUSG00000032199	chr9	71326244	71333742	-	2424	0.524	0
Pus7	ENSMUSG00000057541	chr5	23246466	23289529	-	5276	0.524	0
Dctn4	ENSMUSG00000024603	chr18	60685868	60718416	+	5115	0.522	0
Mki67ip	ENSMUSG00000026377	chr1	120218416	120230399	+	1965	0.522	0
Ash2l	ENSMUSG00000031575	chr8	26926663	26958166	-	5231	0.521	0
Mtmr11	ENSMUSG00000045934	chr3	95965927	95975641	+	3780	0.521	0
Adk	ENSMUSG00000039197	chr14	21871855	22267769	+	1701	0.52	0
Gde1	ENSMUSG00000033917	chr7	125832059	125849292	-	1692	0.519	0
Galntl2	ENSMUSG00000021903	chr14	32842175	32875383	+	6326	0.517	0
Uqcrb	ENSMUSG00000021520	chr13	67001553	67006314	-	876	0.517	0
Cacnb2	ENSMUSG00000057914	chr2	14525933	14909535	+	9224	0.516	0
Pias2	ENSMUSG00000025423	chr18	77303947	77394447	+	5457	0.516	0
Scyl3	ENSMUSG00000026584	chr1	165859231	165885257	+	5809	0.515	0
Sdccag3	ENSMUSG00000026927	chr2	26238320	26244836	-	3589	0.515	0
Sdc4	ENSMUSG00000017009	chr2	164249747	164269387	-	2547	0.514	0
Stx4a	ENSMUSG00000030805	chr7	134967808	134992533	+	2364	0.512	0
Slc36a1	ENSMUSG00000020261	chr11	55017852	55049832	+	5626	0.512	0
Gng11	ENSMUSG00000032766	chr6	3953943	3958445	+	880	0.51	0
Prps1	ENSMUSG00000031432	chrX	136991152	137010679	+	1993	0.51	0
Parvg	ENSMUSG00000022439	chr15	84154456	84173408	+	3090	0.51	0
Fut10	ENSMUSG00000046152	chr8	32297803	32372210	+	5007	0.51	0
Tsga14	ENSMUSG00000029790	chr6	30603457	30643749	-	4999	0.51	0
Cbr4	ENSMUSG00000031641	chr8	63966531	63982279	+	3047	0.51	0
Dars2	ENSMUSG00000026709	chr1	162970732	163000789	-	5195	0.51	0
BC020535	ENSMUSG00000027474	chr2	152891691	152907471	+	2407	0.509	0
Taf5	ENSMUSG00000025049	chr19	47142238	47157969	+	3258	0.509	0
Sox17	ENSMUSG00000025902	chr1	4481009	4486494	-	3154	0.509	0
Letm2	ENSMUSG00000037363	chr8	26688964	26707959	-	2098	0.509	0
E130102H24Rik	ENSMUSG00000086782	chr4	101019136	101028853	-	741	0.508	0
Olfm2	ENSMUSG00000032172	chr9	20472144	20532673	-	1826	0.508	0
Lrrc33	ENSMUSG00000052384	chr16	32142871	32165680	-	4177	0.507	0
B230307C23Rik	ENSMUSG00000080717	chr16	98218488	98231165	+	1298	0.507	0
Prpf4	ENSMUSG00000066148	chr4	62069831	62088024	+	5671	0.506	0
Banp	ENSMUSG00000025316	chr8	124473650	124553158	+	6632	0.506	0
Dpp9	ENSMUSG00000001229	chr17	56326105	56358312	-	3633	0.505	0
Igkv3-4	ENSMUSG00000076600	chr6	70621783	70622371	+	359	0.505	0
Nop56	ENSMUSG00000027405	chr2	130100166	130105049	+	4099	0.503	0
Map3k14	ENSMUSG00000020941	chr11	103081076	103128786	-	6550	0.503	0
Mars2	ENSMUSG00000046994	chr1	55294021	55296902	+	2882	0.502	0
Tgfbrap1	ENSMUSG00000070939	chr1	43104114	43155467	-	6633	0.502	0

4833442J19Rik	ENSMUSG00000039958	chr6	149088371	149099693	+	2496	0.502	0
Syde1	ENSMUSG00000032714	chr10	78047248	78054709	-	3288	0.501	0
Mrpl47	ENSMUSG00000037531	chr3	32624319	32636069	-	3420	0.501	0
Rrp1b	ENSMUSG00000058392	chr17	32173045	32199810	+	7702	0.5	0
Rchy1	ENSMUSG00000029397	chr5	92377930	92392094	-	2894	0.497	0
Pop4	ENSMUSG00000030423	chr7	39047839	39056367	-	1214	0.496	0
Zhx3	ENSMUSG00000035877	chr2	160574444	160698734	-	12710	0.495	0
Sft2d1	ENSMUSG00000073468	chr17	8503967	8589717	+	3580	0.494	0
Lig1	ENSMUSG00000056394	chr7	13862632	13896782	+	4866	0.493	0
Rbm22	ENSMUSG00000024604	chr18	60720390	60732464	+	5338	0.493	0
Ankrd13b	ENSMUSG00000037907	chr11	77283987	77303180	-	3839	0.493	0
Eya3	ENSMUSG00000028886	chr4	132194902	132280680	+	5647	0.492	0
Gm5900	ENSMUSG00000059039	chr7	112098429	112098803	-	375	0.491	0
Glyr1	ENSMUSG00000022536	chr16	5013999	5050003	-	3348	0.491	0
Ankrd13a	ENSMUSG00000041870	chr5	115224686	115256209	+	4098	0.491	0
Specc1l	ENSMUSG00000033444	chr10	74674818	74775144	+	6075	0.49	0
Txn1	ENSMUSG00000028367	chr4	57956245	57969283	-	1051	0.49	0
Fbln7	ENSMUSG00000027386	chr2	128688717	128722770	+	3228	0.49	0
Tsn	ENSMUSG00000026374	chr1	120195094	120207709	-	3040	0.49	0
9430016H08Rik	ENSMUSG00000025971	chr1	57463172	57474797	+	3540	0.489	0
Tmem38b	ENSMUSG00000028420	chr4	53838917	53874891	+	3807	0.489	0
Gm8181	ENSMUSG00000048188	chr18	43330759	43332247	+	1439	0.489	0
Basp1	ENSMUSG00000045763	chr15	25293040	25343519	-	1847	0.489	0
Hck	ENSMUSG00000003283	chr2	152934204	152977177	+	2092	0.489	0
Paqr7	ENSMUSG00000037348	chr4	134052612	134066150	+	4259	0.488	0
Lgals3bp	ENSMUSG00000033880	chr11	118254065	118263406	-	2847	0.488	0
Tmem81	ENSMUSG00000048174	chr1	134402807	134405214	+	1740	0.487	0
Cln8	ENSMUSG00000026317	chr8	14881335	14901720	+	7606	0.486	0
Shc2	ENSMUSG00000020312	chr10	79080685	79100663	-	5537	0.486	0
Sipa1	ENSMUSG00000056917	chr19	5651185	5663707	-	3868	0.484	0
Dio3	ENSMUSG00000075707	chr12	111517278	111519307	+	2030	0.483	0
Wdr61	ENSMUSG00000061559	chr9	54562542	54595757	-	4678	0.483	0
Gnaq	ENSMUSG00000024639	chr19	16207321	16461953	+	6185	0.482	0
Dnajc18	ENSMUSG00000024350	chr18	35830757	35862798	-	5169	0.48	0
Prkab2	ENSMUSG00000038205	chr3	97462116	97477735	+	5096	0.48	0
Hmgn1	ENSMUSG00000040681	chr16	96342225	96349336	-	3221	0.479	0
Cnbp	ENSMUSG00000030057	chr6	87792609	87801100	-	2536	0.479	0
Dnajb1	ENSMUSG00000005483	chr8	86132074	86135801	+	2471	0.479	0

Cd34	ENSMUSG00000016494	chr1	196765015	196787486	+	2621	0.479	0
Cdt1	ENSMUSG00000006585	chr8	125091915	125097028	+	2189	0.478	0
Klf2	ENSMUSG00000055148	chr8	74842932	74845555	+	1847	0.478	0
Pdcd5	ENSMUSG00000030417	chr7	36427001	36432540	-	2486	0.478	0
Lgals7	ENSMUSG00000053522	chr7	29648872	29651303	+	927	0.477	0
Pdhx	ENSMUSG00000010914	chr2	102861232	102913670	-	3930	0.476	0
Rbbp9	ENSMUSG00000027428	chr2	144368001	144376605	-	2362	0.475	0
Agpat4	ENSMUSG00000023827	chr17	12311570	12412511	+	2701	0.474	0
Lbp	ENSMUSG00000016024	chr2	158132229	158158588	+	4831	0.474	0
Prpf6	ENSMUSG00000002455	chr2	181326573	181390365	+	3859	0.474	0
0610030E20Rik	ENSMUSG00000058706	chr6	72297311	72303152	+	4632	0.474	0
Brcc3	ENSMUSG00000031201	chrX	72661967	72699340	+	7889	0.472	0
BC067068	ENSMUSG00000036009	chr10	105200241	105278436	-	2692	0.472	0
Bfar	ENSMUSG00000022684	chr16	13671951	13703705	+	4451	0.471	0
Cnih4	ENSMUSG00000062169	chr1	183081062	183099125	+	3574	0.471	0
Spg21	ENSMUSG00000032388	chr9	65308744	65336277	+	2690	0.47	0
Rbl2	ENSMUSG00000031666	chr8	93593956	93647743	+	6963	0.47	0
Scarf2	ENSMUSG00000012017	chr16	17797375	17808380	+	3302	0.47	0
Eif4b	ENSMUSG00000058655	chr15	101904204	101927603	+	3853	0.47	0
Fahd1	ENSMUSG00000045316	chr17	24985841	24987247	-	1407	0.47	0
Omp	ENSMUSG00000074006	chr7	105291870	105294012	-	2143	0.469	0
Lhfpl2	ENSMUSG00000045312	chr13	94827751	94965364	+	8276	0.468	0
Setd3	ENSMUSG00000056770	chr12	109344641	109417524	-	6035	0.467	0
Ogfod1	ENSMUSG00000033009	chr8	96561098	96591821	+	5846	0.467	0
Sugt1	ENSMUSG00000022024	chr14	79987498	80029562	+	1951	0.466	0
Sash3	ENSMUSG00000031101	chrX	45499613	45514742	+	2677	0.466	0
Pkm2	ENSMUSG00000032294	chr9	59504383	59527182	+	2391	0.466	0
Gm16001	ENSMUSG00000085433	chr5	124561118	124562250	-	1040	0.465	0
C1qa	ENSMUSG00000036887	chr4	136451832	136454718	-	1040	0.464	0
Phrf1	ENSMUSG00000038611	chr7	148414683	148448649	+	6765	0.462	0
Peli2	ENSMUSG00000021846	chr14	48740544	48880558	+	7091	0.462	0
Myst2	ENSMUSG00000038909	chr11	95135573	95171560	-	5536	0.462	0
Taf7	ENSMUSG00000051316	chr18	37800556	37803858	-	2891	0.462	0
Pinx1	ENSMUSG00000021958	chr14	64479149	64538696	+	1351	0.462	0
Xpo6	ENSMUSG00000000131	chr7	133245229	133344015	-	9692	0.459	0
6330418K02Rik	ENSMUSG00000085227	chr5	138705271	138707889	+	1339	0.459	0
Zfp263	ENSMUSG00000022529	chr16	3744093	3750790	+	4521	0.459	0
Polr3e	ENSMUSG00000030880	chr7	128061258	128090946	+	4234	0.458	0
Chic2	ENSMUSG00000029229	chr5	75397051	75440799	-	6712	0.458	0
Xpo5	ENSMUSG00000067150	chr17	46339804	46379248	+	3709	0.458	0
Adat2	ENSMUSG00000019808	chr10	13272700	13283182	+	1466	0.457	0
Ablim3	ENSMUSG00000032735	chr18	61959049	62071506	-	4560	0.457	0

Sec16a	ENSMUSG00000026924	chr2	26264951	26300736	-	9198	0.457		0
Serpinh 1	ENSMUSG00000070436	chr7	106493885	106501740	-	2435	0.455		0
Wbp4	ENSMUSG00000022023	chr14	79859744	79881075	-	3452	0.455		0
Snx1	ENSMUSG00000032382	chr9	65935940	65974394	-	4339	0.455		0
Zfp839	ENSMUSG00000021271	chr12	112088489	112107312	+	2829	0.453		0
Clec1a	ENSMUSG00000033082	chr6	129339308	129402018	-	5237	0.453		0
Ube2b	ENSMUSG00000020390	chr11	51798999	51814264	-	2126	0.453		0
Mll3	ENSMUSG00000038056	chr5	24777612	25004601	-	20296	0.452		0
Rsrc1	ENSMUSG00000034544	chr3	66785312	67162318	+	8279	0.452		0
Adar	ENSMUSG00000027951	chr3	89518944	89557368	+	6786	0.451		0
Rpl23	ENSMUSG00000071415	chr11	97638841	97643751	-	1770	0.451		0
Fam174 a	ENSMUSG00000051185	chr1	97210205	97231861	+	2107	0.45		0
Iqsec1	ENSMUSG00000034312	chr6	90609592	90760117	-	7412	0.45		0
Prkar2a	ENSMUSG00000032601	chr9	108594474	108651843	+	4190	0.448		0
Phf21a	ENSMUSG00000058318	chr2	91933274	92204823	+	8561	0.448		0
Zfp408	ENSMUSG00000075040	chr2	91483826	91489948	-	4008	0.447		0
Agpat5	ENSMUSG00000031467	chr8	18846277	18891361	+	10783	0.447		0
Usp16	ENSMUSG00000025616	chr16	87454948	87483762	+	9454	0.446		0
Hrsp12	ENSMUSG00000022323	chr15	34413776	34425010	-	3192	0.444		0
Hspa1b	ENSMUSG00000090877	chr17	35093381	35096183	-	2803	0.443		0
Zfp82	ENSMUSG00000058447	chr7	30841057	30857842	-	2101	0.441		0
Mrrf	ENSMUSG00000026887	chr2	35991909	36046167	+	2853	0.441		0
l7Rn6	ENSMUSG00000062797	chr7	97066039	97089709	-	2933	0.441		0
Camk2n 1	ENSMUSG00000046447	chr4	138010229	138016038	+	4444	0.441		0
F2rl1	ENSMUSG00000021678	chr13	96281687	96295195	-	2754	0.44		0
Fbxo32	ENSMUSG00000022358	chr15	58011634	58046433	-	2722	0.44		0
Pfkl	ENSMUSG00000020277	chr10	77449692	77472828	-	4395	0.439		0
Ppp1r10	ENSMUSG00000039220	chr17	36053379	36069226	+	5619	0.439		0
Bcar1	ENSMUSG00000031955	chr8	114234367	114267748	-	3295	0.439		0
A43011 0N23Rik	ENSMUSG00000035279	chr7	4877446	4896423	+	4284	0.438		0
Hoxb5	ENSMUSG00000038700	chr11	96163909	96167434	+	2812	0.438		0
Thada	ENSMUSG00000024251	chr17	84589396	84865536	-	7888	0.438		0
Ankrd27	ENSMUSG00000034867	chr7	36371266	36424256	+	4292	0.437		0
Prdm2	ENSMUSG00000057637	chr4	142697294	142802898	-	7694	0.436		0
Tor1aip 1	ENSMUSG00000026466	chr1	157851729	157883610	-	5367	0.436		0
Med10	ENSMUSG00000021598	chr13	69948760	69954972	+	862	0.436		0
D9Ertd4 02e	ENSMUSG00000046603	chr9	122714657	122745452	+	3126	0.436		0
Sun1	ENSMUSG00000036817	chr5	139676591	139725794	+	7596	0.435		0
Gse1	ENSMUSG00000031822	chr8	123012766	123105290	+	7326	0.435		0
Pdss2	ENSMUSG00000038240	chr10	42941292	43184688	+	5963	0.435		0

Rpl4	ENSMUSG00000032399	chr9	64021194	64026369	+	1422	0.434		0
Sp2	ENSMUSG00000018678	chr11	96814654	96844273	-	3415	0.433		0
5730419 I09Rik	ENSMUSG00000030279	chr6	142959440	143048627	-	4475	0.433		0
Ghdc	ENSMUSG00000017747	chr11	100627346	100632271	-	2692	0.432		0
Snx12	ENSMUSG00000046032	chrX	98293125	98417902	-	2953	0.432		0
Ddx31	ENSMUSG00000026806	chr2	28695926	28761091	+	3534	0.43		0
Gigyf2	ENSMUSG00000048000	chr1	89223573	89347371	+	12301	0.429		0
Letm1	ENSMUSG00000005299	chr5	34082322	34125466	-	9650	0.429		0
Higd1a	ENSMUSG00000038412	chr9	121757678	121767118	-	1873	0.429		0
P2ry6	ENSMUSG00000048779	chr7	108086152	108112905	-	1938	0.429		0
Ahcyl2	ENSMUSG00000029772	chr6	29718011	29862310	+	13634	0.429		0
Ppp1r9b	ENSMUSG00000038976	chr11	94852349	94868213	+	5083	0.428		0
Erlec1	ENSMUSG00000020311	chr11	30829784	30854335	-	4250	0.428		0
Tmem5 5a	ENSMUSG00000028221	chr4	14791223	14842323	+	2339	0.427		0
Kif13a	ENSMUSG00000021375	chr13	46844456	47025087	-	7001	0.426		0
Gm1651 6	ENSMUSG00000042549	chr11	60734419	60745369	-	1291	0.426		0
Asb7	ENSMUSG00000030509	chr7	73789451	73834482	-	6226	0.426		0
E2f6	ENSMUSG00000057469	chr12	16817771	16833549	+	2454	0.426		0
Donson	ENSMUSG00000022960	chr16	91677513	91689010	-	2834	0.426		0
Efha2	ENSMUSG00000039478	chr8	41392812	41471662	+	6153	0.426		0
Zfp626	ENSMUSG00000030604	chr7	28592223	28603811	+	1197	0.426		0
Hoxa10	ENSMUSG00000000938	chr6	52181196	52190853	-	3838	0.425		0
Mef2d	ENSMUSG00000001419	chr3	87946294	87976008	+	5985	0.424		0
Rabgef1	ENSMUSG00000025340	chr5	130647668	130690212	+	4168	0.424		0
Smpdl3 a	ENSMUSG00000019872	chr10	57514141	57531636	+	2065	0.424		0
Gm1194 4	ENSMUSG00000084941	chr11	3208999	3220725	+	873	0.424		0
Rundc3 a	ENSMUSG00000006575	chr11	102254717	102263869	+	2941	0.423		0
Rufy4	ENSMUSG00000061815	chr1	74172115	74194797	+	2329	0.422		0
Ccdc43	ENSMUSG00000020925	chr11	102546000	102559096	-	3341	0.422		0
Eif2ak3	ENSMUSG00000031668	chr6	70794509	70855239	+	5428	0.421		0
Cant1	ENSMUSG00000025575	chr11	118267603	118280400	-	4358	0.421		0
Aifm1	ENSMUSG00000036932	chrX	45828121	45866740	-	4006	0.42		0
Zfp647	ENSMUSG00000054967	chr15	76741281	76748440	-	1608	0.42		0
Igf2r	ENSMUSG00000023830	chr17	12875272	12962530	-	9438	0.419		0
C23005 2I12Rik	ENSMUSG00000030493	chr7	36177294	36181836	-	1824	0.418		0
Tcp11l2	ENSMUSG00000020034	chr10	84039371	84077104	+	3438	0.417		0
Efha1	ENSMUSG00000021973	chr14	58535117	58618099	-	2310	0.417		0
Arf4	ENSMUSG00000021877	chr14	27457560	27484570	+	2813	0.416		0
Meaf6	ENSMUSG00000028863	chr4	124762378	124790482	+	5211	0.416		0
Ptpn1	ENSMUSG00000027540	chr2	167757557	167804885	+	5533	0.415		0

Cep164	ENSMUSG00000043987	chr9	45575029	45636774	-	5980	0.415	0
Tspan12	ENSMUSG00000029669	chr6	21721395	21802515	-	2626	0.413	0
Fastkd2	ENSMUSG00000025962	chr1	63777188	63801229	+	5873	0.413	0
Cs	ENSMUSG00000005683	chr10	127774888	127799535	+	2828	0.412	0
Glb1l	ENSMUSG00000026200	chr1	75194818	75207353	-	5107	0.412	0
Cenpq	ENSMUSG00000023919	chr17	41060000	41071996	-	2604	0.41	0
Thap1	ENSMUSG00000037214	chr8	27268684	27273412	+	1328	0.408	0
Gm2a	ENSMUSG00000000594	chr11	54911617	54926531	+	4127	0.407	0
Sec62	ENSMUSG00000027706	chr3	30691798	30720185	+	3966	0.406	0
Csrnp2	ENSMUSG00000044636	chr15	100310001	100325670	-	4126	0.405	0
Cherp	ENSMUSG00000052488	chr8	74984388	74999132	-	3794	0.401	0
Trak1	ENSMUSG00000032536	chr9	121206620	121384034	+	5514	0.401	0
D230037D09Rik	ENSMUSG00000046675	chr12	103981970	103985771	+	3369	0.4	0
Tmem135	ENSMUSG00000039428	chr7	96288233	96552732	-	6104	0.4	0
Elp2	ENSMUSG00000024271	chr18	24762462	24798008	+	3466	0.399	0
Ctnnbip1	ENSMUSG00000028988	chr4	148892345	148940546	+	2792	0.398	0
Pkp2	ENSMUSG00000041957	chr16	16213411	16272805	+	5360	0.398	0
Slc25a17	ENSMUSG00000022404	chr15	81149351	81191195	-	1667	0.397	0
Carhsp1	ENSMUSG00000008393	chr16	8658680	8672246	-	2849	0.396	0
Poglut1	ENSMUSG00000034064	chr16	38525221	38550340	-	4658	0.396	0
Zfp825	ENSMUSG00000069208	chr13	74617965	74620391	-	822	0.396	0
Erlin1	ENSMUSG00000025198	chr19	44109434	44144275	-	4961	0.394	0
Rbbp7	ENSMUSG00000031353	chrX	159198334	159217024	+	2909	0.394	0
Nrd1	ENSMUSG00000053510	chr4	108673260	108734382	+	7140	0.392	0
Ttc27	ENSMUSG00000024078	chr17	75117090	75262910	+	2820	0.392	0
Zfp697	ENSMUSG00000050064	chr3	98186404	98235871	+	5163	0.391	0
Rprd1b	ENSMUSG00000027651	chr2	157854233	157958033	+	6499	0.391	0
Pura	ENSMUSG00000043991	chr18	36440751	36461175	+	14943	0.39	0
Mboat1	ENSMUSG00000038732	chr13	30228367	30338586	+	3474	0.39	0
Pa2g4	ENSMUSG00000025364	chr10	127994822	128002990	-	2751	0.39	0
Dnajb11	ENSMUSG00000004460	chr16	22857918	22879707	+	3159	0.39	0
Higd1b	ENSMUSG00000020928	chr11	102697163	102699354	+	847	0.39	0
Tmem86b	ENSMUSG00000045282	chr7	4579644	4582084	-	1220	0.39	0
Serping1	ENSMUSG00000023224	chr2	84605544	84615601	-	2325	0.389	0
Zfp606	ENSMUSG00000030386	chr7	13063642	13081584	+	7680	0.389	0
Farp2	ENSMUSG00000034066	chr1	95408656	95518553	+	5236	0.389	0
Dnajc27	ENSMUSG00000020657	chr12	4082612	4110602	+	4665	0.388	0
Prr7	ENSMUSG00000034686	chr13	55565628	55574516	+	1360	0.388	0

Znrf3	ENSMUSG00000041961	chr11	5176327	5344850	-	7893	0.387	0
2010204K13Rik	ENSMUSG00000063018	chrX	6988943	7022269	-	1583	0.387	0
Bzw2	ENSMUSG00000020547	chr12	36818433	36883412	-	1860	0.386	0
1200011I18Rik	ENSMUSG00000022008	chr14	76486039	76510622	-	5448	0.386	0
Ifitm1	ENSMUSG00000025491	chr7	148153120	148155724	+	1094	0.385	0
Becn1	ENSMUSG00000035086	chr11	101147266	101163600	-	7065	0.384	0
Pex3	ENSMUSG00000019809	chr10	13243648	13272948	-	4740	0.383	0
Metap1d	ENSMUSG00000041921	chr2	71291333	71363251	+	4537	0.381	0
Asb8	ENSMUSG00000048175	chr15	97965068	97996056	-	2410	0.381	0
Klhl21	ENSMUSG00000073700	chr4	151382912	151391789	+	3986	0.38	0
Adrbk2	ENSMUSG00000042249	chr5	113339498	113444811	-	6797	0.38	0
Noa1	ENSMUSG00000036285	chr5	77723199	77739109	-	4037	0.38	0
Rab14	ENSMUSG00000026878	chr2	35035725	35056640	-	5431	0.38	0
Cyp20a1	ENSMUSG00000049439	chr1	60400167	60444904	+	2286	0.379	0
Sf3b4	ENSMUSG00000068856	chr3	95976255	95981487	+	2162	0.379	0
Fbxo42	ENSMUSG00000028920	chr4	140703828	140759977	+	5934	0.379	0
Hars	ENSMUSG00000001380	chr18	36926184	36942859	-	1987	0.378	0
Ncbp1	ENSMUSG00000028330	chr4	46151485	46185275	+	3386	0.377	0
Ndufaf1	ENSMUSG00000027305	chr2	119481182	119488563	-	1501	0.376	0
Gngt2	ENSMUSG00000038811	chr11	95698530	95707048	+	1877	0.376	0
Ifngr1	ENSMUSG00000020009	chr10	19311755	19330035	+	2847	0.376	0
Rhob	ENSMUSG00000054364	chr12	8504569	8506791	-	2223	0.375	0
Rab9	ENSMUSG00000079316	chrX	162895185	162917799	-	1749	0.374	0
Pigk	ENSMUSG00000039047	chr3	152377064	152643372	+	9367	0.374	0
Pik3r4	ENSMUSG00000032571	chr9	105545325	105589985	+	4884	0.372	0
Gm5506	ENSMUSG00000059040	chr18	48204989	48208032	+	1757	0.372	0
Hs6st1	ENSMUSG00000045216	chr1	36125245	36163291	+	3719	0.371	0
Pvt1	ENSMUSG00000072566	chr15	61869542	62209651	+	5595	0.371	0
Rnase6	ENSMUSG00000021880	chr14	51748743	51750797	+	1072	0.37	0
Gm7334	ENSMUSG00000044645	chr17	50837852	50839205	+	1354	0.37	0
Pigb	ENSMUSG00000079469	chr9	72863503	72887506	-	2193	0.369	0
Atp6v1g1	ENSMUSG00000039105	chr4	63205806	63211784	+	1135	0.369	0
Tbpl1	ENSMUSG00000071359	chr10	22423685	22451744	-	3271	0.368	0
Mtmr3	ENSMUSG00000034354	chr11	4380871	4494866	-	7588	0.368	0
Atg10	ENSMUSG00000021619	chr13	91074954	91363592	-	1609	0.367	0
Akr1e1	ENSMUSG00000045410	chr13	4590003	4608420	-	3555	0.367	0
Kdm5d	ENSMUSG00000056673	chrY	234231	280254	+	5489	0.366	0
Cyb5d1	ENSMUSG00000044795	chr11	69205427	69209133	-	1605	0.365	0
Sgce	ENSMUSG00000004631	chr6	4624350	4697204	-	3086	0.365	0
Ccdc91	ENSMUSG00000030301	chr6	147424393	147581134	+	2859	0.365	0

Metap2	ENSMUSG00000036112	chr10	93321234	93353947	-	4990	0.363		0
Fam116a	ENSMUSG00000040818	chr14	27399064	27452880	+	5710	0.362		0
Stard3nl	ENSMUSG00000003062	chr13	19449546	19487621	-	1283	0.362		0
Dazap1	ENSMUSG00000069565	chr10	79724231	79751153	+	2944	0.361		0
Atp1a1	ENSMUSG00000033161	chr3	101380142	101408586	-	4547	0.361		0
Pex11a	ENSMUSG00000030545	chr7	86880843	86888017	-	2368	0.361		0
Eif2s3x	ENSMUSG00000035150	chrX	91434046	91458201	-	3812	0.36		0
5730528L13Rik	ENSMUSG00000039693	chr4	48552807	48574791	+	1973	0.359		0
Gramd1b	ENSMUSG00000040111	chr9	40100818	40338968	-	9721	0.359		0
Pcyox1	ENSMUSG00000029998	chr6	86336000	86347144	-	4797	0.358		0
Nolc1	ENSMUSG00000015176	chr19	46150353	46160018	+	3634	0.358		0
Pacs2	ENSMUSG00000021143	chr12	114252719	114312612	+	5722	0.357		0
Rassf1	ENSMUSG00000010067	chr9	107453886	107464594	+	5425	0.357		0
Sat1	ENSMUSG00000025283	chrX	151647675	151650992	-	2774	0.357		0
Ly6c2	ENSMUSG00000022584	chr15	74938590	74942114	-	796	0.357		0
Ipo5	ENSMUSG00000030662	chr14	121310416	121347264	+	4591	0.353		0
G3bp1	ENSMUSG00000018583	chr11	55283187	55318340	+	7064	0.353		0
Gpatch2	ENSMUSG00000039210	chr1	189039387	189175583	+	7606	0.352		0
Plcb3	ENSMUSG00000024960	chr19	7028204	7044279	-	4231	0.352		0
6030458C11Rik	ENSMUSG00000022195	chr15	12737932	12754412	-	3974	0.351		0
Taf13	ENSMUSG00000048100	chr3	108374616	108386140	+	3155	0.351		0
Madd	ENSMUSG00000040687	chr2	90977517	91023994	-	9086	0.349		0
Cybasc3	ENSMUSG00000034445	chr19	10651944	10669758	+	3017	0.349		0
BC052040	ENSMUSG00000040282	chr2	115407452	115604504	+	4349	0.348		0
Ctbs	ENSMUSG00000028189	chr3	146113434	146128813	+	2892	0.347		0
Rapgef3	ENSMUSG00000022469	chr15	97575201	97598403	-	6336	0.347		0
Txnip	ENSMUSG00000038393	chr3	96361880	96365806	+	3031	0.347		0
Lag3	ENSMUSG00000030124	chr6	124854379	124861723	-	2109	0.346		0
Psen1	ENSMUSG00000019969	chr12	85029152	85076149	+	3115	0.346		0
4930453N24Rik	ENSMUSG00000059920	chr16	64751577	64770987	-	5948	0.345		0
Dnajb5	ENSMUSG00000036052	chr4	42962686	42972297	+	5936	0.345		0
Skp1a	ENSMUSG00000036309	chr11	52045497	52060360	+	2211	0.343		0
Polr3k	ENSMUSG00000038628	chr2	181599042	181605535	+	3124	0.343		0
Odc1	ENSMUSG00000011179	chr12	17551679	17558308	+	2547	0.343		0
mt-Rnr1	ENSMUSG00000064337	chrM	70	1024	+	955	0.342		0
Taf3	ENSMUSG00000025782	chr2	9836179	9970223	-	6389	0.342		0
Cmbl	ENSMUSG00000022235	chr15	31498546	31519874	+	1228	0.342		0

Nrm	ENSMUSG00000059791	chr17	35998263	36002347	+	1466	0.342	0
Wdr11	ENSMUSG00000042055	chr7	136735377	136779252	+	10372	0.34	0
1110004F10Rik	ENSMUSG00000030663	chr7	123236815	123248724	+	1495	0.339	0
Zc3h4	ENSMUSG00000059273	chr7	16986230	17023043	+	6210	0.339	0
1700056N10Rik	ENSMUSG00000084792	chr16	17047613	17049204	+	1043	0.339	0
Tpst2	ENSMUSG00000029344	chr5	112705711	112744381	+	2789	0.338	0
Ttll1	ENSMUSG00000022442	chr15	83314202	83341323	-	2918	0.338	0
Mapk7	ENSMUSG00000001034	chr11	61302314	61307908	-	4394	0.338	0
Klhl18	ENSMUSG00000054792	chr9	110328431	110379198	-	5100	0.337	0
Kri1	ENSMUSG00000035047	chr9	21077902	21092415	-	2599	0.337	0
Ccdc86	ENSMUSG00000024732	chr19	11015971	11023756	-	3367	0.337	0
Vdac2	ENSMUSG00000021771	chr14	22650491	22665101	+	4658	0.336	0
Pdzd4	ENSMUSG00000002006	chrX	71038698	71070308	-	3970	0.335	0
Mat2b	ENSMUSG00000042032	chr11	40492816	40508705	-	5001	0.335	0
Pan2	ENSMUSG00000005682	chr10	127740391	127758403	+	4444	0.335	0
Vopp1	ENSMUSG00000037788	chr6	57677801	57775148	-	9715	0.334	0
Usp39	ENSMUSG00000056305	chr6	72268672	72295178	-	2425	0.334	0
Tmem127	ENSMUSG00000034850	chr2	127073644	127086843	+	4811	0.333	0
Tspan14	ENSMUSG00000037824	chr14	41719734	41780096	-	2475	0.332	0
Kin	ENSMUSG00000037262	chr2	10002220	10014433	+	1705	0.33	0
Arid1a	ENSMUSG00000007880	chr4	133234923	133312684	-	11476	0.329	0
Sipa1l1	ENSMUSG00000042700	chr12	83270407	83552769	+	8723	0.329	0
Asb3	ENSMUSG00000020305	chr11	30854395	31002704	+	5275	0.329	0
Ap4m1	ENSMUSG00000019518	chr5	138613230	138619936	+	3640	0.328	0
Doc2b	ENSMUSG00000020848	chr11	75582468	75609551	-	4361	0.328	0
Exosc10	ENSMUSG00000017264	chr4	147932538	147956510	+	3788	0.328	0
St7l	ENSMUSG00000045576	chr3	104667421	104730812	+	7583	0.328	0
Fbxo4	ENSMUSG00000022184	chr15	3915445	3929573	-	2047	0.327	0
Eprs	ENSMUSG00000026615	chr1	187186974	187252234	+	7195	0.327	0
Cetn2	ENSMUSG00000031347	chrX	70158871	70163750	-	1646	0.326	0
Fam189a1	ENSMUSG00000030518	chr7	71900982	72301456	-	5298	0.326	0
Son	ENSMUSG00000022961	chr16	91647751	91679466	+	14907	0.326	0
Rasal3	ENSMUSG00000052142	chr17	32527604	32540528	-	4897	0.326	0
Suclg2	ENSMUSG00000061838	chr6	95423006	95668792	-	3392	0.325	0
Adamtsl1	ENSMUSG00000066113	chr4	85699819	86074289	+	10870	0.325	0
Nhlrc2	ENSMUSG00000025078	chr19	56622751	56673336	+	3934	0.325	0
Mbtd1	ENSMUSG00000059474	chr11	93747166	93808299	+	13896	0.325	0
1810030O07Rik	ENSMUSG00000044148	chrX	12232005	12250794	-	2699	0.325	0

Sfrp5	ENSMUSG00000018822	chr19	42272461	42276742	-	1900	0.325	0
Wipf2	ENSMUSG00000038013	chr11	98724952	98766354	+	7819	0.323	0
Golph3l	ENSMUSG00000046519	chr3	95392856	95423164	+	2836	0.322	0
Bmp8b	ENSMUSG00000002384	chr4	122782408	122809342	+	3880	0.321	0
Mapk1ip1	ENSMUSG00000041775	chr7	146027506	146037956	-	1883	0.321	0
Cox11	ENSMUSG00000020544	chr11	90499487	90506245	+	1423	0.321	0
Agap1	ENSMUSG00000055013	chr1	91351381	91791857	+	9586	0.319	0
Tatdn2	ENSMUSG00000056952	chr6	113647044	113661063	+	3548	0.318	0
Wdr12	ENSMUSG00000026019	chr1	60133712	60155489	-	4872	0.317	0
Nr2f2	ENSMUSG00000030551	chr7	77496832	77511621	-	4863	0.316	0
Ppm1b	ENSMUSG00000061130	chr17	85356081	85423331	+	4971	0.315	0
Man2a1	ENSMUSG00000024085	chr17	64950076	65104450	+	7223	0.315	0
Morc2a	ENSMUSG00000034543	chr11	3549497	3590480	+	6013	0.313	0
Zfp652	ENSMUSG00000075595	chr11	95573987	95696429	+	15444	0.313	0
Phka1	ENSMUSG00000034055	chrX	99709314	99839585	-	6259	0.311	0
Kdm2b	ENSMUSG00000029475	chr5	123320677	123465089	-	11818	0.309	0
Toe1	ENSMUSG00000028688	chr4	116466915	116480245	-	2979	0.309	0
Polr3g	ENSMUSG00000035834	chr13	81812842	81850012	-	3936	0.308	0
Ppp2cb	ENSMUSG00000009630	chr8	34710093	34730266	+	1826	0.308	0
Rfwd3	ENSMUSG00000033596	chr8	113794844	113824122	-	4443	0.307	0
Mad1l1	ENSMUSG00000029554	chr5	140484643	140797506	-	3649	0.306	0
Tgfb2	ENSMUSG00000039239	chr1	188447065	188529871	-	4842	0.305	0
Eid2b	ENSMUSG00000070705	chr7	29062725	29065148	+	2424	0.305	0
Sox13	ENSMUSG00000070643	chr1	135278880	135320954	-	4312	0.305	0
Rdh11	ENSMUSG00000066441	chr12	80275324	80293280	-	3456	0.304	0
Rars2	ENSMUSG00000028292	chr4	34562206	34607416	+	2771	0.304	0
Zfp706	ENSMUSG00000062397	chr15	36926786	36937157	-	4413	0.301	0
Ttc8	ENSMUSG00000021013	chr12	100158784	100221448	+	2749	0.301	0
Ran	ENSMUSG00000029430	chr5	129525944	129530198	+	2646	0.3	0
Rps27a	ENSMUSG00000020460	chr11	29445846	29448109	-	1972	0.3	0
Txndc16	ENSMUSG00000021830	chr14	45754123	45840003	-	10360	0.299	0
Clcn6	ENSMUSG00000029016	chr4	147378368	147412930	-	5934	0.299	0
Zc3h6	ENSMUSG00000042851	chr2	128793138	128844299	+	5670	0.299	0
Ttc23	ENSMUSG00000030555	chr7	74792296	74871462	+	6343	0.298	0
Kpna6	ENSMUSG00000003731	chr4	129321224	129350011	-	6729	0.298	0
Nipsnap3b	ENSMUSG00000015247	chr4	53024752	53034932	+	1687	0.297	0
Ighv1-39	ENSMUSG00000076706	chr12	116152810	116153243	-	351	0.296	0
Phka2	ENSMUSG00000031295	chrX	156940098	157036810	+	6068	0.296	0
Acp2	ENSMUSG00000002103	chr2	91043042	91054255	+	5186	0.295	0
Parp9	ENSMUSG00000022906	chr16	35938556	35972691	+	4872	0.295	0
Gtf2a2	ENSMUSG00000033543	chr9	69860357	69870673	+	4047	0.294	0
Gdpd1	ENSMUSG00000061666	chr11	86847369	86887564	-	2336	0.294	0
Acin1	ENSMUSG00000022185	chr14	55260998	55305768	-	8643	0.294	0

Fam101b	ENSMUSG00000020846	chr11	75832696	75841284	-	3512	0.294	0
Nfe2l1	ENSMUSG00000038615	chr11	96678728	96691282	-	5441	0.294	0
Furin	ENSMUSG00000030530	chr7	87533471	87550322	-	9178	0.294	0
Srrt	ENSMUSG00000037364	chr5	137736932	137748902	-	3024	0.292	0
Hrct1	ENSMUSG00000071001	chr4	43740070	43741511	+	1442	0.291	0
Ikbkg	ENSMUSG00000004221	chrX	71638629	71699193	+	8110	0.291	0
Asnsd1	ENSMUSG00000026095	chr1	53382351	53409596	-	4674	0.29	0
Otud3	ENSMUSG00000041161	chr4	138451294	138469915	-	1723	0.29	0
Ankrd37	ENSMUSG00000050914	chr8	47082256	47085221	-	1403	0.289	0
Ppm1f	ENSMUSG00000026181	chr16	16896562	16927457	+	4925	0.289	0
Tyrobp	ENSMUSG00000030579	chr7	31198807	31202596	+	564	0.289	0
Dnm2	ENSMUSG00000033335	chr9	21229352	21312203	+	5323	0.288	0
Ppap2b	ENSMUSG00000028517	chr4	104829952	104905369	+	3159	0.286	0
Cars	ENSMUSG00000010755	chr7	150743135	150785995	-	4651	0.286	0
St6galnac4	ENSMUSG00000079442	chr2	32442615	32455218	+	4035	0.284	0
Phf8	ENSMUSG00000041229	chrX	147955215	148068402	+	9318	0.283	0
Osbp	ENSMUSG00000024687	chr19	12040334	12068595	+	4590	0.282	0
Rnase4	ENSMUSG00000021876	chr14	51710752	51725826	+	1996	0.281	0
Cacna1d	ENSMUSG00000015968	chr14	30853135	31304342	-	9347	0.281	0
Triobp	ENSMUSG00000033088	chr15	78778154	78836299	+	10701	0.279	0
Anxa11	ENSMUSG00000021866	chr14	26661669	26706290	+	4108	0.278	0
Ggn	ENSMUSG00000031493	chr7	29955239	29958952	+	2643	0.277	0
Lix1l	ENSMUSG00000049288	chr3	96405056	96429275	+	2509	0.277	0
Susd1	ENSMUSG00000038578	chr4	59327555	59451505	-	4766	0.277	0
Rbpms	ENSMUSG00000031586	chr8	34893116	35040313	-	2776	0.276	0
Impa2	ENSMUSG00000024525	chr18	67448907	67478800	+	1661	0.276	0
Kctd20	ENSMUSG00000005936	chr17	29088895	29106494	+	4379	0.275	0
Amfr	ENSMUSG00000031751	chr8	96495488	96536563	-	3953	0.275	0
Upf3b	ENSMUSG00000036572	chrX	34631673	34650335	-	4017	0.274	0
Pmpca	ENSMUSG00000026926	chr2	26244859	26252642	+	4282	0.273	0
Dock6	ENSMUSG00000032198	chr9	21604628	21657079	-	6691	0.273	0
Laptm4b	ENSMUSG00000022257	chr15	34167781	34214050	+	1897	0.271	0
Aasdh	ENSMUSG00000055923	chr5	77302684	77334539	-	6287	0.271	0
Gm10110	ENSMUSG00000062093	chr14	90296351	90298273	-	1848	0.27	0
Snrpd3	ENSMUSG00000020180	chr10	74980787	74998185	+	604	0.269	0
2310022B05Rik	ENSMUSG00000031983	chr8	127159666	127187269	-	3297	0.268	0
Zfp384	ENSMUSG00000038346	chr6	124959163	124987883	+	6372	0.268	0
Gpihbp1	ENSMUSG00000022579	chr15	75427088	75428642	+	796	0.267	0
Steap1	ENSMUSG00000015652	chr5	5736317	5749326	-	1377	0.267	0
Cwf19l1	ENSMUSG00000025200	chr19	44183134	44210366	-	3574	0.267	0

Ube2l3	ENSMUSG00000038965	chr16	17152106	17202742	-	5599	0.267	0
Tarsl2	ENSMUSG00000030515	chr7	72789784	72836977	+	4095	0.266	0
Maoa	ENSMUSG00000025037	chrX	16196824	16264944	+	4167	0.264	0
Arpp19	ENSMUSG00000007656	chr9	74885421	74908120	+	4957	0.263	0
Nsdhl	ENSMUSG00000031349	chrX	70163896	70203853	+	2853	0.263	0
Eif3j	ENSMUSG00000027236	chr2	121854282	121882334	+	5830	0.263	0
Cebpb	ENSMUSG00000056501	chr2	167514415	167515918	+	1504	0.262	0
Tpd52	ENSMUSG00000027506	chr3	8928626	9004723	-	4454	0.261	0
Nfil3	ENSMUSG00000056749	chr13	53062578	53076442	-	2026	0.26	0
Zfp644	ENSMUSG00000049606	chr5	106929913	107126306	-	9786	0.259	0
Plxnb1	ENSMUSG00000053646	chr9	108997950	109022425	+	8952	0.259	0
Prickle2	ENSMUSG00000030020	chr6	92320886	92656139	-	8637	0.259	0
Acot13	ENSMUSG00000006717	chr13	24909817	24923386	-	721	0.258	0
Dhx38	ENSMUSG00000037993	chr8	112071917	112089501	-	4197	0.258	0
Chst1	ENSMUSG00000027221	chr2	92439864	92455407	+	2680	0.258	0
Ppm1d	ENSMUSG00000020525	chr11	85124746	85160568	+	3173	0.258	0
Asph	ENSMUSG00000028207	chr4	9375216	9596491	-	16113	0.258	0
Mllt10	ENSMUSG00000026743	chr2	17976898	18134017	+	5875	0.257	0
Ccdc80	ENSMUSG00000022665	chr16	45093515	45128037	+	4140	0.257	0
Six4	ENSMUSG00000034460	chr12	74200596	74215024	-	7035	0.256	0
Pgam5	ENSMUSG00000029500	chr5	110688149	110698932	-	2373	0.255	0
Sap130	ENSMUSG00000024260	chr18	31794025	31882715	+	4030	0.254	0
Rab3ip	ENSMUSG00000064181	chr10	116342837	116387436	-	3397	0.252	0
Prx	ENSMUSG00000053198	chr7	28284343	28305233	+	5579	0.252	0
Psmd11	ENSMUSG00000017428	chr11	80242117	80286750	+	6401	0.251	0
M6pr	ENSMUSG00000007458	chr6	122258738	122267698	+	4170	0.251	0
Nucks1	ENSMUSG00000026434	chr1	133807035	133832898	+	6096	0.25	0
Fam133b	ENSMUSG00000058503	chr5	3543844	3569810	+	1555	0.249	0
Rps3a	ENSMUSG00000028081	chr3	85941862	85946624	-	977	0.249	0
Rpl7	ENSMUSG00000043716	chr1	16091376	16094743	-	2687	0.248	0
Dgkq	ENSMUSG00000004815	chr5	109075712	109098691	-	7189	0.247	0
Kif21a	ENSMUSG00000022629	chr15	90763707	90880382	-	6501	0.247	0
Enpp4	ENSMUSG00000023961	chr17	44233257	44242758	-	4572	0.247	0
Dnajc1	ENSMUSG00000026740	chr2	18117281	18314457	-	11651	0.247	0
Fbxo18	ENSMUSG00000058594	chr2	11664200	11699209	-	5115	0.247	0
Eml2	ENSMUSG00000040811	chr7	19761770	19791831	+	3540	0.246	0
Rarres1	ENSMUSG00000049404	chr3	67282894	67319445	-	1325	0.246	0
Bpnt1	ENSMUSG00000026617	chr1	187156028	187181656	+	2263	0.245	0
Polr3d	ENSMUSG00000000776	chr14	70838555	70843278	-	2049	0.245	0
Tcf3	ENSMUSG00000020167	chr10	79872259	79896392	-	4039	0.244	0
Csrnp1	ENSMUSG00000032515	chr9	119880284	119886368	-	2870	0.243	0
2210012G02Rik	ENSMUSG00000028619	chr4	106806760	106851721	-	7193	0.242	0
Lactb	ENSMUSG00000032370	chr9	66803200	66823291	-	2031	0.242	0

Ube2r2	ENSMUSG00000036241	chr4	41082776	41140413	+	4189	0.241	0
Gpr133	ENSMUSG00000044017	chr5	129602625	129710474	+	8321	0.241	0
Polr3a	ENSMUSG00000025280	chr14	25267916	25306268	-	4683	0.241	0
Wsb2	ENSMUSG00000029364	chr5	117807313	117828610	+	5857	0.241	0
Atp9b	ENSMUSG00000024566	chr18	80931816	81130797	-	4500	0.239	0
Rbm45	ENSMUSG00000042369	chr2	76208041	76221825	+	1898	0.239	0
Add1	ENSMUSG00000029106	chr5	34916313	34974957	+	6066	0.239	0
Kbtbd4	ENSMUSG00000005505	chr2	90744897	90751783	+	3139	0.237	0
G6pd2	ENSMUSG00000089992	chr5	62200082	62201716	+	1635	0.237	0
Samhd1	ENSMUSG00000027639	chr2	156923265	156961001	-	9154	0.237	0
Rpl9	ENSMUSG00000047215	chr5	65779603	65782682	-	3080	0.236	0
Lpl	ENSMUSG00000015568	chr8	71404390	71431347	+	6810	0.233	0
Ttc32	ENSMUSG00000066637	chr12	9036803	9043200	+	1000	0.232	0
Dusp3	ENSMUSG00000003518	chr11	101832457	101848327	-	4682	0.232	0
Creb3l1	ENSMUSG00000027230	chr2	91822485	91864659	-	2603	0.229	0
Aoc3	ENSMUSG00000019326	chr11	101191919	101203252	+	4621	0.229	0
Snx3	ENSMUSG00000019804	chr10	42221836	42255187	+	1413	0.229	0
Cobll1	ENSMUSG00000034903	chr2	64926396	65077460	-	8342	0.228	0
Cdc23	ENSMUSG00000024370	chr18	34790605	34811389	-	9500	0.227	0
Chaf1b	ENSMUSG00000022945	chr16	93884146	93906360	+	5001	0.227	0
Dpt	ENSMUSG00000026574	chr1	166726863	166754397	+	1636	0.226	0
C1qb	ENSMUSG00000036905	chr4	136436044	136442102	-	1088	0.226	0
Tnrc6b	ENSMUSG00000047888	chr15	80590016	80771516	+	17043	0.226	0
Odf2	ENSMUSG00000026790	chr2	29744741	29787266	+	9922	0.226	0
Adcy6	ENSMUSG00000022994	chr15	98420425	98438064	-	6017	0.225	0
Tlcd2	ENSMUSG00000038217	chr11	75275217	75284401	+	1936	0.225	0
Sorbs3	ENSMUSG00000022091	chr14	70580275	70607444	-	2914	0.224	0
Fam109b	ENSMUSG00000049687	chr15	82169372	82176173	+	3311	0.224	0
Ptpru	ENSMUSG00000028909	chr4	131324372	131394203	-	7113	0.224	0
Cep97	ENSMUSG00000022604	chr16	55900001	55934968	-	9163	0.222	0
Kcnab2	ENSMUSG00000028931	chr4	151764851	151851980	-	5578	0.221	0
Ado	ENSMUSG00000057134	chr10	67007260	67011703	-	4444	0.221	0
Pdap1	ENSMUSG00000029623	chr5	145889638	145901107	-	3201	0.22	0
Eif2ak1	ENSMUSG00000029613	chr5	144578657	144665125	+	5675	0.22	0
Ifitm5	ENSMUSG00000025489	chr7	148134862	148136138	-	708	0.219	0
Dctd	ENSMUSG00000031562	chr8	49184446	49238587	+	3409	0.219	0
Scnn1g	ENSMUSG00000000216	chr7	128877993	128911989	+	2991	0.218	0
4732418C07Rik	ENSMUSG00000034210	chr4	115410349	115449932	+	8201	0.218	0
1810035L17Rik	ENSMUSG00000021040	chr12	88784846	88793156	+	3104	0.217	0
Brap	ENSMUSG00000029458	chr5	122110572	122137257	+	6284	0.217	0
Hnrnpa0	ENSMUSG00000007836	chr13	58227240	58229917	-	2678	0.217	0

Csde1	ENSMUSG00000068823	chr3	102824469	102862112	+	4201	0.216		0
Hexb	ENSMUSG00000021665	chr13	97946287	97968312	-	1904	0.215		0
Cyb5d2	ENSMUSG00000057778	chr11	72590734	72609644	-	5143	0.215		0
Pik3r3	ENSMUSG00000028698	chr4	115894223	115975661	+	5300	0.214		0
Msl1	ENSMUSG00000052915	chr11	98656830	98669173	+	5433	0.213		0
Dcn	ENSMUSG00000019929	chr10	96942245	96980777	+	1937	0.213		0
Mllt4	ENSMUSG00000068036	chr17	13897546	14043157	+	10226	0.212		0
Fgd1	ENSMUSG00000025265	chrX	147480693	147524064	+	5784	0.211		0
Ppp1r13l	ENSMUSG00000040734	chr7	19945098	19963882	+	3604	0.21		0
Clint1	ENSMUSG00000006169	chr11	45665507	45724127	+	6981	0.21		0
Mto1	ENSMUSG00000032342	chr9	78296015	78323155	+	4078	0.21		0
Med15	ENSMUSG00000012114	chr16	17651301	17723040	-	3546	0.209		0
Sprr1a	ENSMUSG00000050359	chr3	92287874	92289817	-	806	0.208		0
Clic1	ENSMUSG00000007041	chr17	35186911	35195694	+	1799	0.207		0
Alas1	ENSMUSG00000032786	chr9	106135786	106150457	-	4310	0.206		0
C1qtnf6	ENSMUSG00000022440	chr15	78353776	78360055	-	2402	0.205		0
Pdxp	ENSMUSG00000068221	chr15	78744349	78749947	+	2001	0.204		0
Mark4	ENSMUSG00000030397	chr7	20010816	20043843	-	3049	0.203		0
Abcd3	ENSMUSG00000028127	chr3	121461827	121518220	-	4089	0.203		0
Crebbp	ENSMUSG00000022521	chr16	4084048	4213404	-	7508	0.203		0
Ccdc126	ENSMUSG00000050786	chr6	49269273	49291581	+	2421	0.203		0
Nars2	ENSMUSG00000018995	chr7	104100015	104213271	+	4213	0.203		0
Glt8d1	ENSMUSG00000021916	chr14	31814579	31825627	+	2521	0.202		0
Rbbp5	ENSMUSG00000026439	chr1	134373944	134402242	+	3632	0.202		0
Pdpr	ENSMUSG00000033624	chr8	113618530	113660974	+	5028	0.201		0
Mid1ip1	ENSMUSG00000008035	chrX	10292139	10296816	+	2128	0.2		0
Mpzl3	ENSMUSG00000070305	chr9	44863269	44885519	+	6387	0.199		0
Fam123b	ENSMUSG00000050332	chrX	92615657	92640211	-	8496	0.198		0
Psma4	ENSMUSG00000032301	chr9	54798597	54805837	+	2370	0.198		0
Ptma	ENSMUSG00000026238	chr1	88423311	88427273	+	1192	0.198		0
Dagla	ENSMUSG00000035735	chr19	10319755	10379367	-	5747	0.198		0
1110057K04Rik	ENSMUSG00000037669	chr12	8214913	8292564	+	2772	0.197		0
Eftud2	ENSMUSG00000020929	chr11	102699787	102742299	-	8395	0.197		0
H3f3b	ENSMUSG00000016559	chr11	115883226	115889276	-	2976	0.196		0
Ascc2	ENSMUSG00000020412	chr11	4537750	4585702	+	4964	0.196		0
Rrbp1	ENSMUSG00000027422	chr2	143773131	143836999	-	8786	0.196		0
Spry1	ENSMUSG00000037211	chr3	37538869	37543520	+	2906	0.196		0
Ddx1	ENSMUSG00000037149	chr12	13226114	13256019	-	2507	0.195		0
Gm9762	ENSMUSG00000037096	chr3	78770278	78770721	-	444	0.195		0
Smpd4	ENSMUSG00000005899	chr16	17619447	17644921	+	8926	0.194		0
Slc25a13	ENSMUSG00000015112	chr6	5991219	6167173	-	3136	0.193		0

Fam114a1	ENSMUSG00000029185	chr5	65361310	65433113	+	2964	0.191	0
Zbtb10	ENSMUSG00000069114	chr3	9250602	9285333	+	7417	0.19	0
Wdr20a	ENSMUSG00000037957	chr12	111976154	112042450	+	3268	0.19	0
2310004I24Rik	ENSMUSG00000020910	chr11	66851407	66866096	-	1977	0.19	0
Snx27	ENSMUSG00000028136	chr3	94301466	94386638	-	6321	0.189	0
Dctn6	ENSMUSG00000031516	chr8	35153474	35171850	-	2853	0.189	0
Zranb1	ENSMUSG00000030967	chr7	140122825	140178074	+	5431	0.188	0
Zfp772	ENSMUSG00000066838	chr7	7154833	7162506	-	2569	0.188	0
Nudt17	ENSMUSG00000028100	chr3	96509815	96512483	-	1362	0.188	0
Gmpr2	ENSMUSG00000002326	chr14	56291072	56297587	+	1543	0.188	0
Secisbp2	ENSMUSG00000035139	chr13	51747066	51779413	+	4626	0.187	0
Ankrd12	ENSMUSG00000034647	chr17	66316841	66426429	-	11508	0.185	0
Nf2	ENSMUSG00000009073	chr11	4665848	4749539	-	6826	0.185	0
Pprc1	ENSMUSG00000055491	chr19	46119376	46147405	+	7516	0.184	0
Ubap1	ENSMUSG00000028437	chr4	41296029	41337558	+	3583	0.183	0
Pcbd1	ENSMUSG00000020098	chr10	60552091	60557076	+	809	0.183	0
Ckap4	ENSMUSG00000046841	chr10	83989050	83996781	-	2954	0.183	0
Stip1	ENSMUSG00000024966	chr19	7095186	7114516	-	2173	0.183	0
Fam125b	ENSMUSG00000038740	chr2	33585473	33743466	-	5292	0.182	0
Rph3al	ENSMUSG00000020847	chr11	75644501	75751931	-	3814	0.182	0
Zfp36	ENSMUSG00000044786	chr7	29161803	29164274	-	1792	0.181	0
Cbx6	ENSMUSG00000089715	chr15	79616783	79665118	-	10502	0.181	0
Gm17388	ENSMUSG00000091448	chr4	135911689	135913957	+	1919	0.181	0
Entpd5	ENSMUSG00000021236	chr12	85714807	85749979	-	6039	0.18	0
Fam118a	ENSMUSG00000022434	chr15	84867492	84893258	+	2543	0.18	0
Phkb	ENSMUSG00000036879	chr8	88364858	88585275	+	7417	0.179	0
Synj2bp	ENSMUSG00000090935	chr12	82575339	82633898	-	6028	0.179	0
Cdkn2c	ENSMUSG00000028551	chr4	109333481	109339794	-	2686	0.179	0
Opn3	ENSMUSG00000026525	chr1	177592552	177622907	-	1913	0.179	0
Spire1	ENSMUSG00000024533	chr18	67647863	67770443	-	5628	0.178	0
Cask	ENSMUSG00000031012	chrX	13094206	13428493	-	9166	0.178	0
2810021J22Rik	ENSMUSG00000020491	chr11	58680718	58696790	+	3931	0.178	0
Pold3	ENSMUSG00000030726	chr7	107230621	107270075	-	4134	0.177	0
Slc25a30	ENSMUSG00000022003	chr14	76161806	76186844	-	1706	0.176	0
F11r	ENSMUSG00000038235	chr1	173367666	173394734	+	2663	0.176	0
Sart1	ENSMUSG00000039148	chr19	5377523	5388703	-	5091	0.175	0
Tsg101	ENSMUSG00000014402	chr7	54144319	54175339	-	2730	0.173	0
Ccdc117	ENSMUSG00000020482	chr11	5428890	5442190	-	3372	0.173	0

Acat1	ENSMUSG00000032047	chr9	53388627	53418487	-	3758	0.171	0
Eng	ENSMUSG00000026814	chr2	32502115	32538189	+	3870	0.169	0
Zbtb24	ENSMUSG00000019826	chr10	41170189	41185385	+	2865	0.169	0
Rbm15b	ENSMUSG00000074102	chr9	106783260	106784620	-	1361	0.168	0
Arsk	ENSMUSG00000021592	chr13	76197870	76236108	-	5681	0.167	0
Smarcc2	ENSMUSG00000025369	chr10	127896292	127927229	+	5027	0.166	0
Zdhhc9	ENSMUSG00000036985	chrX	45525146	45562055	-	6733	0.166	0
Supt6h	ENSMUSG00000002052	chr11	78020248	78059489	-	7095	0.164	0
1700017B05Rik	ENSMUSG00000032300	chr9	57100129	57110406	-	4934	0.162	0
2310003F16Rik	ENSMUSG00000027245	chr2	121279026	121284408	+	5278	0.16	0
2810021B07Rik	ENSMUSG00000012429	chr13	17787247	17790939	+	2672	0.159	0
Ranbp10	ENSMUSG00000037415	chr8	108292208	108351250	-	5308	0.159	0
Timm17a	ENSMUSG00000062580	chr1	137198109	137210355	-	1574	0.159	0
Dctn5	ENSMUSG00000030868	chr7	129276555	129292558	+	2649	0.158	0
Hinfp	ENSMUSG00000032119	chr9	44103756	44113754	-	2100	0.158	0
Ptpn3	ENSMUSG00000038764	chr4	57203713	57314709	-	7242	0.157	0
D19Wsu162e	ENSMUSG00000047731	chr19	46673574	46731879	+	4463	0.157	0
Bud13	ENSMUSG00000032077	chr9	46091091	46106866	+	3980	0.156	0
Gatc	ENSMUSG00000029536	chr5	115783251	115791187	-	1794	0.156	0
Chek2	ENSMUSG00000029521	chr5	111269036	111303152	+	2247	0.155	0
Yipf5	ENSMUSG00000024487	chr18	40364519	40379053	-	2329	0.155	0
Cercam	ENSMUSG00000039787	chr2	29724684	29738360	+	3383	0.154	0
Pigb	ENSMUSG00000032203	chr9	72856288	72863464	-	2866	0.154	0
Snw1	ENSMUSG00000021039	chr12	88790886	88813249	-	2231	0.154	0
Rab3gap1	ENSMUSG00000036104	chr1	129765350	129840445	+	7006	0.153	0
Trip10	ENSMUSG00000019487	chr17	57388874	57402846	+	2184	0.152	0
Dis3l	ENSMUSG00000032396	chr9	64154564	64189095	-	5457	0.151	0
Zfp646	ENSMUSG00000049739	chr7	135020310	135029510	+	6727	0.151	0
Scarb2	ENSMUSG00000029426	chr5	92872899	92934634	-	2110	0.15	0
Mrps9	ENSMUSG00000060679	chr1	42908078	42962527	+	2361	0.15	0
1700021K19Rik	ENSMUSG00000035629	chr16	32821793	32877852	-	7459	0.15	0
Setd1a	ENSMUSG00000042308	chr7	134920184	134943636	+	6795	0.149	0
Homez	ENSMUSG00000057156	chr14	55471573	55489798	-	5907	0.149	0
Exoc4	ENSMUSG00000029763	chr6	33199085	33923979	+	12640	0.148	0
Rnf41	ENSMUSG00000025373	chr10	127848672	127878494	+	4374	0.148	0
Crmp1	ENSMUSG00000029121	chr5	37633319	37683371	+	3355	0.147	0

Cic	ENSMUSG00000005442	chr7	26052723	26079178	+	10300	0.147	0
Osgepl1	ENSMUSG00000026096	chr1	53370468	53383187	+	6147	0.147	0
Serf1	ENSMUSG00000021643	chr13	100877952	100884526	+	1315	0.146	0
Leng8	ENSMUSG00000035545	chr7	4088641	4099775	+	6391	0.146	0
Exosc3	ENSMUSG00000028322	chr4	45329485	45355604	-	1324	0.145	0
Ern1	ENSMUSG00000020715	chr11	106255964	106349166	-	8173	0.144	0
Nsun3	ENSMUSG00000050312	chr16	62732270	62786644	-	4816	0.142	0
Lpar2	ENSMUSG00000031861	chr8	72346328	72355001	+	5837	0.141	0
Scnn1a	ENSMUSG00000030340	chr6	125270677	125294961	+	4132	0.141	0
Pik3r1	ENSMUSG00000041417	chr13	102450716	102538172	-	7396	0.141	0
4930579 G24Rik	ENSMUSG00000027811	chr3	79433001	79436739	+	1967	0.141	0
Etv6	ENSMUSG00000030199	chr6	133985718	134220176	+	5983	0.137	0
Taf12	ENSMUSG00000028899	chr4	131830290	131851681	+	4038	0.136	0
Iffo1	ENSMUSG00000038271	chr6	125095259	125111800	+	5691	0.136	0
Ing5	ENSMUSG00000026283	chr1	95700542	95718678	+	4539	0.135	0
Ccdc12 3	ENSMUSG00000023072	chr7	36182054	36223708	+	5088	0.135	0
Mcph1	ENSMUSG00000039842	chr8	18595131	18803189	+	7806	0.135	0
Tprkb	ENSMUSG00000054226	chr6	85865732	85880278	+	3491	0.134	0
Max	ENSMUSG00000059436	chr12	78038256	78063235	-	2005	0.134	0
Snupn	ENSMUSG00000055334	chr9	56798685	56831013	+	1391	0.133	0
Nfx1	ENSMUSG00000028423	chr4	40917939	40973026	+	8564	0.133	0
Smap2	ENSMUSG00000032870	chr4	120640922	120689852	-	3160	0.133	0
Cenpo	ENSMUSG00000020652	chr12	4196004	4234294	-	7263	0.133	0
Alg2	ENSMUSG00000039740	chr4	47477939	47487205	-	3450	0.133	0
Tmem2 2	ENSMUSG00000070287	chr9	100452612	100471504	-	1580	0.132	0
Trp53	ENSMUSG00000059552	chr11	69393861	69405375	+	4045	0.129	0
Iqce	ENSMUSG00000036555	chr5	141137781	141178332	-	12037	0.129	0
Dnaja4	ENSMUSG00000032285	chr9	54546680	54564122	+	3589	0.129	0
Eci2	ENSMUSG00000021417	chr13	35069617	35118963	-	2615	0.128	0
Pcsk7	ENSMUSG00000035382	chr9	45714652	45737775	+	3785	0.127	0
Fxr2	ENSMUSG00000018765	chr11	69446492	69466799	+	3766	0.127	0
Mapk9	ENSMUSG00000020366	chr11	49660253	49699923	+	6784	0.127	0
Hmgb1	ENSMUSG00000066551	chr5	149858278	149865089	-	4944	0.126	0
Fut11	ENSMUSG00000039357	chr14	21514190	21519419	+	3344	0.126	0
Psma3	ENSMUSG00000060073	chr12	72075608	72097334	+	3894	0.126	0
Lrrc59	ENSMUSG00000020869	chr11	94491081	94506530	+	3073	0.126	0
Cmtm8	ENSMUSG00000041012	chr9	114698463	114753270	-	1064	0.123	0
Lepre1	ENSMUSG00000028641	chr4	118905520	118921580	+	3856	0.122	0
Tfdp2	ENSMUSG00000032411	chr9	96096694	96224065	+	7459	0.122	0
Taf5l	ENSMUSG00000038697	chr8	126520218	126545209	-	3033	0.122	0
Rhod	ENSMUSG00000041845	chr19	4425459	4439432	-	1714	0.121	0
Anks6	ENSMUSG00000066191	chr4	47028541	47070178	-	5237	0.12	0
Uty	ENSMUSG00000068457	chrY	433304	582202	-	7687	0.12	0

Akap1	ENSMUSG00000018428	chr11	88692106	88725900	-	4390	0.12	0
Arf3	ENSMUSG00000051853	chr15	98568052	98593645	-	3837	0.12	0
Timm8a1	ENSMUSG00000048007	chrX	131071795	131076404	-	1421	0.12	0
Tmem8b	ENSMUSG00000078716	chr4	43681843	43705540	+	5553	0.119	0
Ctso	ENSMUSG00000028015	chr3	81736523	81760647	+	4573	0.119	0
Wiz	ENSMUSG00000024050	chr17	32491000	32526384	-	9242	0.119	0
Hgs	ENSMUSG00000025793	chr11	120328949	120345298	+	3278	0.118	0
Srp72	ENSMUSG00000036323	chr5	77403708	77428962	+	5717	0.117	0
Fastkd3	ENSMUSG00000021532	chr13	68721123	68731156	+	2378	0.117	0
Rnps1	ENSMUSG00000034681	chr17	24551620	24562840	+	1920	0.117	0
Ccndbp1	ENSMUSG00000023572	chr2	120834139	120842640	+	3469	0.117	0
Sh2d3c	ENSMUSG00000059013	chr2	32576575	32611032	+	5233	0.117	0
Scd2	ENSMUSG00000025203	chr19	44368166	44381354	+	5453	0.116	0
Clcn2	ENSMUSG00000022843	chr16	20703037	20717819	-	6022	0.116	0
Atp8b1	ENSMUSG00000039529	chr18	64688633	64820654	-	6440	0.115	0
Mrps5	ENSMUSG00000027374	chr2	127412958	127432565	+	6578	0.115	0
Mettl21a	ENSMUSG00000025956	chr1	64653047	64663816	-	3005	0.115	0
Tbc1d2	ENSMUSG00000039813	chr4	46617262	46663081	-	5425	0.115	0
Pdcd6	ENSMUSG00000021576	chr13	74440569	74454774	-	1524	0.114	0
Mapk8ip3	ENSMUSG00000024163	chr17	25029098	25073922	-	14546	0.114	0
Abi3	ENSMUSG00000018381	chr11	95691386	95703790	-	4155	0.113	0
Ablim1	ENSMUSG00000025085	chr19	57107223	57389409	-	11965	0.11	0
Qsox2	ENSMUSG00000036327	chr2	26064157	26093045	-	4644	0.11	0
Cotl1	ENSMUSG00000031827	chr8	122333125	122364479	-	1628	0.108	0
Srsf6	ENSMUSG00000016921	chr2	162757264	162762857	+	3795	0.108	0
Golga2	ENSMUSG00000002546	chr2	32142904	32163441	+	5505	0.108	0
Tep1	ENSMUSG00000006281	chr14	51443737	51490235	-	8168	0.107	0
Pcmt1	ENSMUSG00000019795	chr10	7349171	7400934	-	4718	0.105	0
Atpbd4	ENSMUSG00000057147	chr2	114342152	114480700	-	7407	0.105	0
C030048B08Rik	ENSMUSG00000040302	chr5	3583978	3596585	-	3071	0.105	0
Zfp3	ENSMUSG00000043602	chr11	70577711	70586430	+	2061	0.104	0
Vps53	ENSMUSG00000017288	chr11	75859728	75993149	-	5203	0.101	0
Me2	ENSMUSG00000024556	chr18	73929694	73975046	-	2455	0.101	0
Prickle3	ENSMUSG00000031145	chrX	7234386	7245312	+	4902	0.1	0
Psmd2	ENSMUSG00000006998	chr16	20651725	20663487	+	4038	0.1	0
Rilpl1	ENSMUSG00000029392	chr5	124943091	124981400	-	2251	0.1	0
Atp6v1d	ENSMUSG00000021114	chr12	79941712	79962625	-	3676	0.098	0
Tmem111	ENSMUSG00000030286	chr6	113464868	113481632	-	2422	0.097	0

Fam120b	ENSMUSG00000014763	chr17	15533210	15570544	+	4518	0.095	0
Rint1	ENSMUSG00000028999	chr5	23293529	23326187	+	6972	0.095	0
Snrpf	ENSMUSG00000020018	chr10	93045774	93052451	-	861	0.095	0
Slc4a11	ENSMUSG00000074796	chr2	130509849	130523255	-	4998	0.095	0
Hspa5	ENSMUSG00000026864	chr2	34627490	34632051	+	3513	0.095	0
2310010G23Rik	ENSMUSG00000090647	chrX	34354766	34357180	-	1020	0.095	0
1700109H08Rik	ENSMUSG00000008307	chr5	3571566	3584356	+	880	0.095	0
Sugp2	ENSMUSG00000036054	chr8	72758125	72803814	+	14428	0.094	0
Hsp90ab1	ENSMUSG00000023944	chr17	45704724	45710220	-	2999	0.094	0
Rsl24d1	ENSMUSG00000032215	chr9	72961233	72971140	+	1716	0.091	0
Dnajc16	ENSMUSG00000040697	chr4	141316104	141346846	-	6510	0.091	0
Glrx2	ENSMUSG00000018196	chr1	145586159	145596806	+	5249	0.091	0
Fam50a	ENSMUSG00000001962	chrX	71558372	71565488	+	2288	0.091	0
Hvcn1	ENSMUSG00000064267	chr5	122656813	122692306	+	3563	0.089	0
Mfap1a	ENSMUSG00000068479	chr2	121317647	121332401	-	4198	0.086	0
Plod1	ENSMUSG00000019055	chr4	147283862	147310876	-	6765	0.086	0
Vps41	ENSMUSG00000041236	chr13	18809161	18958678	+	3195	0.084	0
Rtn4ip1	ENSMUSG00000019864	chr10	43621613	43667668	+	3135	0.084	0
Paox	ENSMUSG00000025464	chr7	147311556	147320233	+	1765	0.083	0
Zfp1	ENSMUSG00000055835	chr8	114167343	114194908	+	1886	0.083	0
Fcf1	ENSMUSG00000021243	chr12	86311847	86324253	+	1117	0.082	0
Tmem63b	ENSMUSG00000036026	chr17	45797120	45823854	-	5098	0.081	0
Rab6b	ENSMUSG00000032549	chr9	103014404	103087600	+	4734	0.08	0
Shisa4	ENSMUSG00000041889	chr1	137267634	137271814	-	2418	0.079	0
Cog6	ENSMUSG00000027742	chr3	52786045	52821145	-	3154	0.078	0
Leprotl1	ENSMUSG00000031513	chr8	35198626	35210087	-	2879	0.077	0
Pea15a	ENSMUSG00000013698	chr1	174126859	174136935	-	3861	0.077	0
Ggt5	ENSMUSG00000006344	chr10	75052126	75079713	+	4410	0.076	0
Nrf1	ENSMUSG00000058440	chr6	29997988	30103458	+	10282	0.075	0
Eps15l1	ENSMUSG00000006276	chr8	74864900	74945373	-	3182	0.075	0
Tcof1	ENSMUSG00000024613	chr18	60973409	61008625	-	5266	0.075	0
Hdac7	ENSMUSG00000022475	chr15	97623095	97674933	-	5530	0.074	0
Atp5s	ENSMUSG00000054894	chr12	70825949	70845645	+	1485	0.073	0
Emid1	ENSMUSG00000034164	chr11	5006268	5052260	-	2890	0.073	0
Apip	ENSMUSG00000010911	chr2	102913832	102932801	+	1000	0.073	0
Ndufs4	ENSMUSG00000021764	chr13	115078003	115178302	-	1534	0.071	0
Ttc28	ENSMUSG00000033209	chr5	111308822	111718800	+	12316	0.07	0

Kdelr2	ENSMUSG00000079111	chr5	144165499	144182951	+	1846	0.069	0
Irgm2	ENSMUSG00000069874	chr11	58028530	58036282	+	3475	0.069	0
Adra1b	ENSMUSG00000050541	chr11	43588108	43714712	-	3382	0.069	0
Rasl10a	ENSMUSG00000034209	chr11	4958131	4960388	+	1505	0.067	0
Ldlrap1	ENSMUSG00000037295	chr4	134300650	134323939	-	3829	0.066	0
Pisd	ENSMUSG00000023452	chr5	33078949	33128295	-	3814	0.066	0
Zdhhc6	ENSMUSG00000024982	chr19	55364080	55390522	-	2563	0.064	0
Hr	ENSMUSG00000022096	chr14	70952019	70973355	+	6919	0.064	0
Zkscan6	ENSMUSG00000018347	chr11	65620677	65642741	+	2371	0.063	0
Cox7c	ENSMUSG00000017778	chr13	86184421	86186509	-	1131	0.063	0
Stk40	ENSMUSG00000042608	chr4	125781201	125818273	+	4117	0.062	0
Clcn3	ENSMUSG00000004319	chr8	63389186	63462097	-	17071	0.06	0
Tshz1	ENSMUSG00000046982	chr18	84181019	84255796	-	5656	0.059	0
Gosr2	ENSMUSG00000020946	chr11	103538163	103559212	-	3266	0.058	0
Clec16a	ENSMUSG00000068663	chr16	10545457	10744971	+	8343	0.058	0
AK157302	ENSMUSG00000078139	chr13	21587092	21589015	+	1924	0.058	0
Surf2	ENSMUSG00000014873	chr2	26771887	26775703	+	3408	0.057	0
Sec14l1	ENSMUSG00000020823	chr11	116976482	117020582	+	7176	0.057	0
Vti1b	ENSMUSG00000021124	chr12	80257004	80273654	-	1612	0.056	0
Galk2	ENSMUSG00000027207	chr2	125684845	125810035	+	6088	0.056	0
Bin1	ENSMUSG00000024381	chr18	32536871	32595390	+	2451	0.055	0
Zbtb12	ENSMUSG00000049823	chr17	35016428	35033787	+	1995	0.054	0
Vapb	ENSMUSG00000054455	chr2	173563012	173609837	+	7131	0.053	0
Zfp560	ENSMUSG00000045519	chr9	20149580	20189621	-	4981	0.053	0
Sult2b1	ENSMUSG00000003271	chr7	52985353	53014982	-	1888	0.051	0
2010001M06Rik	ENSMUSG00000085042	chr5	135487984	135489123	-	701	0.05	0
Amd1	ENSMUSG00000075232	chr10	40007265	40021992	-	3203	0.05	0
Sec31a	ENSMUSG00000035325	chr5	100790669	100845253	-	4461	0.05	0
Zfp91	ENSMUSG00000024695	chr19	12838150	12870616	-	10046	0.05	0
Eif2c1	ENSMUSG00000041530	chr4	126112256	126145827	-	8226	0.047	0
Hirip3	ENSMUSG00000042606	chr7	134005486	134008891	+	3023	0.047	0
Atmin	ENSMUSG00000047388	chr8	119467293	119484345	+	4877	0.047	0
Rfx5	ENSMUSG00000005774	chr3	94757997	94765483	+	5497	0.047	0
Zfp703	ENSMUSG00000085795	chr8	28087808	28091933	+	3304	0.046	0
Cntrob	ENSMUSG00000032782	chr11	69112989	69137375	-	6758	0.046	0
Vgll4	ENSMUSG00000030315	chr6	114810646	114920012	-	4156	0.046	0
Sdha	ENSMUSG00000021577	chr13	74459702	74487728	-	3883	0.046	0
mt-Rnr2	ENSMUSG00000064339	chrM	1094	2675	+	1582	0.046	0
Tpst1	ENSMUSG00000034118	chr5	130549196	130611599	+	2789	0.044	0
Ier3ip1	ENSMUSG00000090000	chr18	77168756	77180344	+	1894	0.044	0
Safb2	ENSMUSG00000042625	chr17	56700388	56724008	-	8275	0.041	0

Cggbp1	ENSMUSG00000054604	chr16	64851822	64859333	+	4522	0.041		0
H2afy2	ENSMUSG00000020086	chr10	61201391	61246895	-	2197	0.041		0
Acyp1	ENSMUSG00000008822	chr12	86613348	86629388	-	1380	0.041		0
Tmem1 99	ENSMUSG00000051232	chr11	78320557	78325674	-	1902	0.039		0
Ppme1	ENSMUSG00000030718	chr7	107475247	107520406	-	2361	0.038		0
Brd7	ENSMUSG00000031660	chr8	90854938	90886093	-	5244	0.037		0
2810004 N23Rik	ENSMUSG00000031984	chr8	127363255	127386929	-	1382	0.037		0
Ccnk	ENSMUSG00000021258	chr12	109424580	109441569	+	2463	0.036		0
Tmem1 32a	ENSMUSG00000024736	chr19	10932312	10944430	-	4069	0.036		0
Mnat1	ENSMUSG00000021103	chr12	74224704	74374975	+	2505	0.035		0
Grasp	ENSMUSG00000000531	chr15	101054638	101063186	+	2013	0.035		0
Muc1	ENSMUSG00000042784	chr3	89032979	89037303	+	2346	0.035		0
Reep1	ENSMUSG00000052852	chr6	71657555	71760704	+	3924	0.035		0
Per1	ENSMUSG00000020893	chr11	68908719	68923462	+	5631	0.034		0
Enah	ENSMUSG00000022995	chr1	183834576	183950111	-	4288	0.034		0
Blvra	ENSMUSG00000001999	chr2	126896401	126922820	+	2446	0.031		0
Spef1	ENSMUSG00000027329	chr2	130995997	131013018	-	2747	0.031		0
Rarg	ENSMUSG00000001288	chr15	102065369	102087948	-	4109	0.031		0
Mrpl18	ENSMUSG00000057388	chr17	13104215	13109211	-	1398	0.03		0
Zbtb4	ENSMUSG00000018750	chr11	69579414	69597525	+	8117	0.029		0
4933434 E20Rik	ENSMUSG00000027942	chr3	89855558	89867263	+	3858	0.029		0
Cnot3	ENSMUSG00000035632	chr7	3596892	3612710	+	4340	0.029		0
1810022 K09Rik	ENSMUSG00000078784	chr3	14606320	14611221	-	528	0.029		0
Parp12	ENSMUSG00000038507	chr6	39036409	39068348	-	3995	0.028		0
D1Bwg0 212e	ENSMUSG00000003135	chr1	39591837	39603726	+	3500	0.028		0
Pex12	ENSMUSG00000018733	chr11	83108144	83116088	-	3536	0.027		0
Trappc1 0	ENSMUSG00000000374	chr10	77649470	77707387	-	5048	0.025		0
Gon4l	ENSMUSG00000054199	chr3	88639153	88772903	+	7894	0.025		0
Pnpla7	ENSMUSG00000036833	chr2	24831553	24909577	+	7501	0.024		0
Smtn	ENSMUSG00000020439	chr11	3417526	3440615	-	4380	0.024		0
Lss	ENSMUSG00000033105	chr10	75994333	76019883	+	10787	0.023		0
Dnaja3	ENSMUSG00000004069	chr16	4639989	4707695	+	3912	0.022		0
Mras	ENSMUSG00000032470	chr9	99285841	99337300	-	4470	0.022		0
Tagln2	ENSMUSG00000026547	chr1	174430178	174437511	+	2082	0.022		0
Cxcl14	ENSMUSG00000021508	chr13	56390008	56397912	-	1823	0.021		0
Rhoa	ENSMUSG00000007815	chr9	108208536	108240270	+	2126	0.021		0
Flii	ENSMUSG00000002812	chr11	60527625	60540765	-	5039	0.02		0
Sp100	ENSMUSG00000026222	chr1	87546563	87606573	+	7556	0.02		0

Tmem62	ENSMUSG00000054484	chr2	120802753	120833588	+	3348	0.02		0
Usp11	ENSMUSG00000031066	chrX	20281032	20297665	+	4732	0.02		0
Tspan11	ENSMUSG00000030351	chr6	127837607	127903049	+	4380	0.019		0
Plscr3	ENSMUSG00000019461	chr11	69659878	69665560	+	3148	0.019		0
Pts	ENSMUSG00000032067	chr9	50329723	50336746	-	1035	0.019		0
Rhoc	ENSMUSG00000002233	chr3	104591929	104597377	+	1292	0.019		0
Ipmk	ENSMUSG00000060733	chr10	70810511	70896075	+	7093	0.019		0
Tubgcp6	ENSMUSG00000051786	chr15	88928787	88953517	-	7965	0.018		0
Mta1	ENSMUSG00000021144	chr12	114336489	114375417	+	3104	0.018		0
Ercc4	ENSMUSG00000022545	chr16	13109777	13150710	+	6730	0.017		0
Pdxdc1	ENSMUSG00000022680	chr16	13833241	13903224	-	8009	0.016		0
Ces1d	ENSMUSG00000056973	chr8	95689967	95721737	-	2369	0.016		0
Ccdc125	ENSMUSG00000048924	chr13	101439436	101467194	+	2704	0.016		0
S100a13	ENSMUSG00000042312	chr3	90318357	90328503	+	715	0.016		0
Gtf2f2	ENSMUSG00000067995	chr14	76296744	76410672	-	1467	0.014		0
Tpcn1	ENSMUSG00000032741	chr5	120984169	121038622	-	4645	0.014		0
1110002N22Rik	ENSMUSG00000046909	chr11	79950180	79955677	-	1570	0.014		0
Vdac3	ENSMUSG00000008892	chr8	23687573	23704230	-	1339	0.014		0
Mgl2	ENSMUSG00000040950	chr11	69943831	69951052	+	2158	0.014		0
Bccip	ENSMUSG00000030983	chr7	140901016	140912828	+	2038	0.013		0
Sf3a1	ENSMUSG00000002129	chr11	4060353	4082544	+	6147	0.012		0
Marcksl1	ENSMUSG00000047945	chr4	129190825	129193229	+	1605	0.012		0
Aco1	ENSMUSG00000028405	chr4	40090114	40146042	+	5197	0.012		0
Crispld2	ENSMUSG00000031825	chr8	122516338	122576693	+	4777	0.011		0
Rtcd1	ENSMUSG00000000339	chr3	116191881	116211126	-	1880	0.011		0
G0s2	ENSMUSG00000009633	chr1	195098355	195099382	-	842	0.01		0
Lig3	ENSMUSG00000020697	chr11	82594610	82617776	+	7281	0.01		0
C230096C10Rik	ENSMUSG00000078517	chr4	138908502	138934645	+	6683	0.008		0
Zbtb40	ENSMUSG00000060862	chr4	136535647	136604716	-	10710	0.008		0
Dstyk	ENSMUSG00000042046	chr1	134314030	134363536	+	6385	0.007		0
Trerf1	ENSMUSG00000064043	chr17	47277891	47496407	+	4544	0.004		0
Ercc1	ENSMUSG00000003549	chr7	19930127	19941873	+	3111	0.004		0
Hexim1	ENSMUSG00000048878	chr11	102977639	102981039	+	3401	0.003		0
Bcas2	ENSMUSG00000005687	chr3	102975578	102983089	+	4390	0.003		0
Dtx3l	ENSMUSG00000049502	chr16	35926597	35939237	-	5724	0.003		0
Rab11fip5	ENSMUSG00000051343	chr6	85284957	85324628	-	6170	0.002		0

1110004E09Rik	ENSMUSG00000022972	chr16	90926054	90935172	-	1360	0.002		0
Exoc3	ENSMUSG00000034152	chr13	74307253	74346148	-	4596	0.002		0
Gtpbp8	ENSMUSG00000022668	chr16	44736881	44746476	-	3527	0.001		0
Copg	ENSMUSG00000030058	chr6	87837808	87863589	+	10905	8E-04		0
Tjap1	ENSMUSG00000012296	chr17	46394825	46419962	-	2557	-3E-04		0
Acadsb	ENSMUSG00000030861	chr7	138554115	138589725	+	4474	-5E-04		0
Ufsp2	ENSMUSG00000031634	chr8	47060882	47082312	+	2566	-0.002		0
Dnajb9	ENSMUSG00000014905	chr12	45306884	45311055	-	1865	-0.003		0
Upf3a	ENSMUSG00000038398	chr8	13785615	13798538	+	1402	-0.003		0
Oraov1	ENSMUSG00000031072	chr7	152101005	152116653	+	4400	-0.004		0
Mrps33	ENSMUSG00000029918	chr6	39751803	39760987	-	1236	-0.004		0
Scgb1a1	ENSMUSG00000024653	chr19	9158126	9162448	-	476	-0.005		0
Unc50	ENSMUSG00000026111	chr1	37487015	37495969	+	1548	-0.007		0
Mcm2	ENSMUSG00000002870	chr6	88833469	88848774	-	3370	-0.009		0
Cep63	ENSMUSG00000032534	chr9	102488908	102528864	-	7450	-0.01		0
Dsp	ENSMUSG00000054889	chr13	38243197	38290446	+	9625	-0.011		0
Cnot10	ENSMUSG00000056167	chr9	114494996	114549192	-	2853	-0.011		0
Stard7	ENSMUSG00000027367	chr2	127095954	127124670	+	5569	-0.012		0
Slc45a4	ENSMUSG00000079020	chr15	73407854	73476192	-	8471	-0.013		0
Dstn	ENSMUSG00000015932	chr2	143741056	143769060	+	1911	-0.013		0
Il3ra	ENSMUSG00000068758	chr14	15179009	15188004	+	1394	-0.013		0
Ptpn23	ENSMUSG00000036057	chr9	110287593	110310714	-	5336	-0.013		0
Tmtc1	ENSMUSG00000030306	chr6	148180952	148392911	-	8578	-0.015		0
Jmjd4	ENSMUSG00000036819	chr11	59263547	59272069	+	7115	-0.016		0
Nap1l4	ENSMUSG00000059119	chr7	150699484	150734997	-	2285	-0.017		0
Pld4	ENSMUSG00000052160	chr12	113998866	114007197	+	1978	-0.018		0
Mybbp1a	ENSMUSG00000040463	chr11	72254857	72265270	+	5803	-0.018		0
Ctnnbl1	ENSMUSG00000027649	chr2	157563137	157717350	+	2705	-0.018		0
Traf4	ENSMUSG00000017386	chr11	77972001	77979091	-	3292	-0.018		0
Socs7	ENSMUSG00000038485	chr11	97223865	97259856	+	7265	-0.02		0
Ccdc115	ENSMUSG00000042111	chr1	34493516	34496517	-	1704	-0.02		0
Sgpp1	ENSMUSG00000021054	chr12	76815235	76836716	-	3324	-0.02		0
Glg1	ENSMUSG00000003316	chr8	113678321	113783116	-	7090	-0.02		0
Fam171a2	ENSMUSG00000034685	chr11	102298295	102308996	-	3119	-0.021		0
Angpt4	ENSMUSG00000027460	chr2	151736943	151771073	+	2338	-0.021		0
BC017643	ENSMUSG00000039294	chr11	121083902	121090636	-	4213	-0.021		0
Dut	ENSMUSG00000027203	chr2	125072926	125084344	+	2410	-0.022		0
Zfp623	ENSMUSG00000050846	chr15	75771382	75779807	+	2463	-0.022		0
Adh5	ENSMUSG00000028138	chr3	138106057	138118463	+	2801	-0.023		0
Pip5k1a	ENSMUSG00000028126	chr3	94862452	94910852	-	4383	-0.024		0

Cdh3	ENSMUSG00000061048	chr8	109034791	109080808	+	3995	-0.024	0
Nuak2	ENSMUSG00000009772	chr1	134212703	134230065	+	3920	-0.026	0
Atp6v1b2	ENSMUSG00000006273	chr8	71612545	71637610	+	5305	-0.026	0
Atp5f1	ENSMUSG00000000563	chr3	105745616	105763017	-	4105	-0.026	0
Tubb5	ENSMUSG00000001525	chr17	35970871	35975251	-	3322	-0.026	0
Med19	ENSMUSG00000027080	chr2	84518559	84528372	+	2059	-0.027	0
Car5b	ENSMUSG00000031373	chrX	160414754	160465929	-	6354	-0.028	0
Otud5	ENSMUSG00000031154	chrX	7418490	7453752	+	5200	-0.028	0
Tanc2	ENSMUSG00000053580	chr11	105451300	105790618	+	16494	-0.028	0
Gripap1	ENSMUSG00000031153	chrX	7366891	7397693	+	5502	-0.029	0
Cabin1	ENSMUSG00000020196	chr10	75108855	75227102	-	7913	-0.029	0
Slc17a5	ENSMUSG00000049624	chr9	78384295	78435848	-	3356	-0.029	0
Sri	ENSMUSG00000003161	chr5	8046078	8069379	+	3734	-0.029	0
Krt8	ENSMUSG00000049382	chr15	101827142	101834773	-	1805	-0.03	0
Gm13705	ENSMUSG00000086382	chr2	73422907	73427265	+	701	-0.03	0
2210404O07Rik	ENSMUSG00000078439	chr10	80855796	80858137	+	1058	-0.03	0
Rbm4b	ENSMUSG00000033760	chr19	4756525	4765938	+	1831	-0.03	0
Zbtb5	ENSMUSG00000049657	chr4	45004114	45025284	-	5606	-0.031	0
Slu7	ENSMUSG00000020409	chr11	43247246	43261483	+	4048	-0.034	0
Gpatch1	ENSMUSG00000063808	chr7	36061555	36103459	-	3295	-0.035	0
D6Wsu116e	ENSMUSG00000024104	chr6	116158051	116212686	+	4300	-0.035	0
Znrf1	ENSMUSG00000033545	chr8	114059997	114149930	+	7018	-0.036	0
Nisch	ENSMUSG00000021910	chr14	31984114	32030132	-	13202	-0.036	0
Lonp2	ENSMUSG00000047866	chr8	89147942	89247772	+	5346	-0.037	0
Ptrh1	ENSMUSG00000053746	chr2	32631306	32645691	+	1719	-0.037	0
Ubn2	ENSMUSG00000038538	chr6	38383925	38474825	+	21472	-0.037	0
Serpinb8	ENSMUSG00000026315	chr1	109486583	109505486	+	3220	-0.038	0
Cript	ENSMUSG00000024146	chr17	87424890	87435150	+	1712	-0.039	0
Gm10093	ENSMUSG00000061062	chr17	78890905	78892881	+	1977	-0.039	0
Srsf10	ENSMUSG00000028676	chr4	135411662	135425823	+	7003	-0.04	0
Zfp61	ENSMUSG00000050605	chr7	25072327	25086251	-	3820	-0.04	0
Atp6v0a2	ENSMUSG00000038023	chr5	125079673	125204824	+	5303	-0.04	0
Ddx18	ENSMUSG00000001674	chr1	123450412	123464566	-	2483	-0.041	0
Ubap2	ENSMUSG00000028433	chr4	41141346	41222177	-	4802	-0.041	0
Gfpt1	ENSMUSG00000029992	chr6	86992840	87042191	+	7419	-0.041	0
Ext2	ENSMUSG00000027198	chr2	93535788	93662725	-	6674	-0.042	0
Ccdc8	ENSMUSG00000041117	chr7	17578057	17582864	+	4808	-0.042	0
Tmem186	ENSMUSG00000043140	chr16	8633977	8637805	-	2736	-0.043	0

Dnajc24	ENSMUSG00000027166	chr2	105806866	105843706	-	1369	-0.043	0
Foxj2	ENSMUSG00000003154	chr6	122769932	122795384	+	5283	-0.043	0
Fyb	ENSMUSG00000022148	chr15	6529871	6613321	+	5194	-0.044	0
Pml	ENSMUSG00000036986	chr9	58064986	58097593	-	6468	-0.045	0
Ssx2ip	ENSMUSG00000036825	chr3	146067606	146103103	+	4195	-0.045	0
Actl6a	ENSMUSG00000027671	chr3	32607468	32625890	+	2764	-0.045	0
Lgals4	ENSMUSG00000053964	chr7	29618813	29626722	+	2370	-0.048	0
Ick	ENSMUSG00000009828	chr9	77956999	78019914	+	11524	-0.048	0
9130011 E15Rik	ENSMUSG00000039901	chr19	45892634	46072978	-	2946	-0.049	0
Kdm1b	ENSMUSG00000038080	chr13	47138868	47179982	+	4829	-0.05	0
Rdh5	ENSMUSG00000025350	chr10	128350649	128359944	-	2809	-0.05	0
C2	ENSMUSG00000024371	chr17	34993357	35035210	-	4777	-0.05	0
Lamp1	ENSMUSG00000031447	chr8	13159135	13175338	+	2255	-0.05	0
Ctsz	ENSMUSG00000016256	chr2	174252994	174264540	-	1441	-0.051	0
Mgat2	ENSMUSG00000043998	chr12	70285145	70287759	+	2615	-0.052	0
Hoxd13	ENSMUSG00000001819	chr2	74506367	74509656	+	2483	-0.052	0
Adal	ENSMUSG00000027259	chr2	120966164	120982416	+	5317	-0.052	0
Lman1	ENSMUSG00000041891	chr18	66140408	66182234	-	6411	-0.053	0
Nfrkb	ENSMUSG00000042185	chr9	31193777	31228918	+	9953	-0.055	0
Igtp	ENSMUSG00000078853	chr11	58013058	58021093	+	2022	-0.056	0
Qsox1	ENSMUSG00000033684	chr1	157625285	157660013	-	6159	-0.056	0
Inpp1	ENSMUSG00000026102	chr1	52846257	52874532	-	2474	-0.057	0
Prdx1	ENSMUSG00000028691	chr4	116358149	116373427	+	3043	-0.057	0
Tcf7l2	ENSMUSG00000024985	chr19	55816300	56008144	+	8739	-0.058	0
Sbno2	ENSMUSG00000035673	chr10	79520161	79565443	-	4531	-0.059	0
Kctd15	ENSMUSG00000030499	chr7	35424033	35440528	-	2991	-0.059	0
Plcd3	ENSMUSG00000020937	chr11	102931618	102962972	-	3393	-0.059	0
Gpam	ENSMUSG00000024978	chr19	55142142	55201824	-	6346	-0.059	0
Mina	ENSMUSG00000022724	chr16	59471601	59492287	+	5290	-0.06	0
Fam98a	ENSMUSG00000002017	chr17	75936426	75951286	-	3479	-0.061	0
Cgref1	ENSMUSG00000029161	chr5	31235519	31247853	-	1328	-0.062	0
D19Ertd 386e	ENSMUSG00000025184	chr19	42593249	42666833	+	7397	-0.063	0
Nostrin	ENSMUSG00000034738	chr2	68973857	69027387	+	1888	-0.063	0
Arhgap3 9	ENSMUSG00000033697	chr15	76554415	76648600	-	7367	-0.064	0
Gpc3	ENSMUSG00000055653	chrX	49625603	49967127	-	2690	-0.064	0
Fbrsl1	ENSMUSG00000043323	chr5	110790773	110877522	-	5003	-0.064	0
Ly6c1	ENSMUSG00000079018	chr15	74875447	74879260	-	840	-0.064	0
Prpf38a	ENSMUSG00000063800	chr4	108235777	108251941	-	5298	-0.067	0
Rpp38	ENSMUSG00000049950	chr2	3246221	3249915	-	1042	-0.067	0
Tmem6 3a	ENSMUSG00000026519	chr1	182872475	182905243	+	4498	-0.067	0
Sufu	ENSMUSG00000025231	chr19	46471386	46563294	+	6945	-0.068	0

Polr1e	ENSMUSG00000028318	chr4	45031455	45049437	+	3897	-0.069	0
Dynlt3	ENSMUSG00000031176	chrX	9231393	9240129	-	2576	-0.072	0
Zfp64	ENSMUSG00000027551	chr2	168718831	168781087	-	5926	-0.072	0
Heatr3	ENSMUSG00000031657	chr8	90661754	90695926	+	4497	-0.073	0
RP23-85E24.1	ENSMUSG00000093514	chr17	71203107	71225305	+	443	-0.074	0
Xkr8	ENSMUSG00000037752	chr4	132280812	132288461	-	4312	-0.075	0
9930014A18Rik	ENSMUSG00000044513	chr15	60654520	60663348	+	5523	-0.075	0
Trim37	ENSMUSG00000018548	chr11	86940579	87034185	+	7214	-0.075	0
Capn5	ENSMUSG00000035547	chr7	105270069	105326784	-	5485	-0.075	0
Itpr2	ENSMUSG00000030287	chr6	146056821	146450745	-	13501	-0.076	0
Fkbp15	ENSMUSG00000066151	chr4	61961376	62021582	-	7515	-0.076	0
Tmed2	ENSMUSG00000029390	chr5	124990704	125000515	+	2199	-0.077	0
Zfhx2	ENSMUSG00000040721	chr14	55679099	55711161	-	10562	-0.077	0
Dennd4b	ENSMUSG00000042404	chr3	90069107	90084591	+	6031	-0.077	0
Srsf7	ENSMUSG00000024097	chr17	80599429	80606629	-	2375	-0.078	0
Acox1	ENSMUSG00000020777	chr11	116033202	116060359	-	5082	-0.078	0
Rbm19	ENSMUSG00000029594	chr5	120566519	120648394	+	3086	-0.08	0
Strbp	ENSMUSG00000026915	chr2	37338748	37559379	-	18487	-0.08	0
Arid1b	ENSMUSG00000069729	chr17	4994332	5347656	+	12600	-0.081	0
2700097O09Rik	ENSMUSG00000062198	chr12	56146648	56181097	-	4939	-0.082	0
Nmnat1	ENSMUSG00000028992	chr4	148841681	148859311	-	3037	-0.082	0
Limd1	ENSMUSG00000025239	chr9	123387819	123430666	+	4849	-0.082	0
Gpnmb	ENSMUSG00000029816	chr6	48986517	49020928	+	4611	-0.082	0
Clasrp	ENSMUSG00000061028	chr7	20166393	20189817	-	2153	-0.083	0
Mtf1	ENSMUSG00000028890	chr4	124479348	124527044	+	8907	-0.084	0
Cnot8	ENSMUSG00000020515	chr11	57917655	57932096	+	2636	-0.085	0
Ppp3ca	ENSMUSG00000028161	chr3	136333088	136600691	+	5512	-0.086	0
4833439L19Rik	ENSMUSG00000025871	chr13	54652579	54666796	-	3220	-0.089	0
Zfp526	ENSMUSG00000046541	chr7	26006470	26012526	+	3354	-0.092	0
Klf9	ENSMUSG00000033863	chr19	23215716	23241401	+	3263	-0.092	0
Bcl9l	ENSMUSG00000063382	chr9	44307219	44318471	+	5304	-0.092	0
Rg9mtd3	ENSMUSG00000035601	chr4	45309999	45329003	+	2641	-0.093	0
Oxct1	ENSMUSG00000022186	chr15	3976383	4105344	+	3701	-0.093	0
Inpp5e	ENSMUSG00000026925	chr2	26251769	26264723	-	4301	-0.093	0
Sf1	ENSMUSG00000024949	chr19	6363690	6378030	+	6211	-0.093	0
Fam40a	ENSMUSG00000014601	chr3	107415450	107434628	-	3234	-0.094	0
Mob3a	ENSMUSG00000003348	chr10	80148001	80164565	-	2644	-0.094	0
Slc4a2	ENSMUSG00000028962	chr5	23929655	23946768	+	6195	-0.094	0

Sesn1	ENSMUSG00000038332	chr10	41530380	41628230	+	3327	-0.098		0
Fen1	ENSMUSG00000024742	chr19	10273622	10278659	-	2422	-0.099		0
Gm7204	ENSMUSG00000048164	chr16	48218693	48219565	+	873	-0.1		0
Rpp14	ENSMUSG00000023156	chr14	8912827	8924360	+	2467	-0.1		0
Cc2d1b	ENSMUSG00000028582	chr4	108292545	108306728	+	4771	-0.101		0
Diexf	ENSMUSG00000016181	chr1	194930597	194956445	-	5548	-0.101		0
Smarcd1	ENSMUSG00000023018	chr15	99532718	99544426	+	3153	-0.101		0
Ndfip1	ENSMUSG00000024425	chr18	38578629	38624053	+	930	-0.103		0
Nek8	ENSMUSG00000017405	chr11	77979608	77990177	-	5222	-0.103		0
1200016B10Rik	ENSMUSG00000052748	chr1	153214829	153275585	-	9099	-0.104		0
Kdm5c	ENSMUSG00000025332	chrX	148667563	148709078	+	7110	-0.104		0
Gm16008	ENSMUSG00000087381	chr4	155397836	155405752	-	942	-0.105		0
Me1	ENSMUSG00000032418	chr9	86474970	86589947	-	3577	-0.105		0
Ralgapa2	ENSMUSG00000037110	chr2	146065615	146337762	-	18358	-0.106		0
Reep4	ENSMUSG00000033589	chr14	70945430	70948739	+	1295	-0.106		0
Wdr53	ENSMUSG00000022787	chr16	32247313	32257169	+	1523	-0.107		0
Mccc1	ENSMUSG00000027709	chr3	35858234	35899600	-	10442	-0.109		0
Gpx7	ENSMUSG00000028597	chr4	108072995	108079566	-	1290	-0.11		0
Zfp959	ENSMUSG00000003198	chr17	56031607	56038007	+	1620	-0.111		0
Gm16836	ENSMUSG00000087471	chr14	51490039	51498678	+	2650	-0.111		0
Gk5	ENSMUSG00000041440	chr9	96019781	96085046	+	6065	-0.112		0
Pgs1	ENSMUSG00000017715	chr11	117847606	117885325	+	5333	-0.113		0
D230025D16Rik	ENSMUSG00000031889	chr8	107749045	107776953	+	4704	-0.115		0
Poli	ENSMUSG00000038425	chr18	70668334	70690274	-	3863	-0.116		0
Brf1	ENSMUSG00000011158	chr12	114198073	114238832	-	2755	-0.117		0
Rpl15	ENSMUSG00000012405	chr14	19100337	19103905	-	2397	-0.118		0
Gcc1	ENSMUSG00000029708	chr6	28366091	28378390	-	5287	-0.119		0
Hist1h4i	ENSMUSG00000060639	chr13	22132685	22133221	-	537	-0.12		0
Cxx1b	ENSMUSG00000067924	chrX	50972355	50973585	-	1231	-0.122		0
Bcl2l2	ENSMUSG00000089682	chr14	55502214	55507071	+	4462	-0.122		0
Tmem59l	ENSMUSG00000035964	chr8	73007775	73011257	-	1482	-0.123		0
Cmc1	ENSMUSG00000039163	chr9	117974213	118059198	-	499	-0.123		0
Rbks	ENSMUSG00000029136	chr5	31926812	32000000	-	1489	-0.123		0
Fam35a	ENSMUSG00000041471	chr14	35050227	35123689	-	3446	-0.124		0
Agap2	ENSMUSG00000025422	chr10	126515963	126530225	+	5633	-0.125		0
Igkv6-32	ENSMUSG00000076576	chr6	70024018	70024578	-	347	-0.125		0
Gatsl3	ENSMUSG00000020424	chr11	4118228	4122412	+	1574	-0.126		0

Coq3	ENSMUSG00000028247	chr4	21806820	21839309	+	2997	-0.126	0
Gm20457	ENSMUSG00000092187	chr7	71482436	71490850	-	682	-0.126	0
Rrp8	ENSMUSG00000030888	chr7	112880721	112885890	-	3072	-0.131	0
Wdr33	ENSMUSG00000024400	chr18	31963711	32068641	+	6413	-0.132	0
Rere	ENSMUSG00000039852	chr4	149655755	149996075	+	8525	-0.136	0
Fech	ENSMUSG00000024588	chr18	64616204	64648720	-	2901	-0.137	0
Tm9sf4	ENSMUSG00000068040	chr2	152987039	153036202	+	7281	-0.137	0
Frg1	ENSMUSG00000031590	chr8	42482811	42502472	-	1085	-0.137	0
Ncoa5	ENSMUSG00000039804	chr2	164825857	164860367	-	4383	-0.138	0
AW209491	ENSMUSG00000039182	chr13	14722525	14730469	+	2010	-0.138	0
Zfp629	ENSMUSG00000045639	chr7	134750545	134759311	-	6723	-0.138	0
Hn1l	ENSMUSG00000024165	chr17	25079101	25097581	-	2933	-0.139	0
Jup	ENSMUSG00000001552	chr11	100230272	100259077	-	6436	-0.14	0
Vti1a	ENSMUSG00000024983	chr19	55390817	55701797	+	7969	-0.141	0
Aen	ENSMUSG00000030609	chr7	86040740	86056095	+	5687	-0.141	0
Bag1	ENSMUSG00000028416	chr4	40883431	40895314	-	1336	-0.141	0
Eif2b3	ENSMUSG00000028683	chr4	116692007	116759911	+	6553	-0.143	0
Tmem123	ENSMUSG00000050912	chr9	7764041	7794333	+	3109	-0.143	0
Smg6	ENSMUSG00000038290	chr11	74739325	74977950	+	8453	-0.144	0
D930016D06Rik	ENSMUSG00000090739	chr5	104954754	104983230	+	4509	-0.145	0
Tyro3	ENSMUSG00000027298	chr2	119623469	119643840	+	6226	-0.146	0
Tusc5	ENSMUSG00000046275	chr11	76493310	76512166	+	3302	-0.147	0
Nudt2	ENSMUSG00000028443	chr4	41412184	41427959	+	845	-0.148	0
Ifnar2	ENSMUSG00000022971	chr16	91373028	91405834	+	3329	-0.148	0
Manba	ENSMUSG00000028164	chr3	135148575	135234368	+	6971	-0.148	0
Slc40a1	ENSMUSG00000025993	chr1	45964915	45982439	-	3380	-0.148	0
Map2k3	ENSMUSG00000018932	chr11	60745535	60766313	+	3314	-0.15	0
Ulk1	ENSMUSG00000029512	chr5	111213507	111239116	-	5213	-0.151	0
Rcc1	ENSMUSG00000028896	chr4	131887834	131909520	-	3848	-0.152	0
Mob3c	ENSMUSG00000028709	chr4	115500697	115508790	+	2975	-0.152	0
Hba-a2	ENSMUSG00000069917	chr11	32196489	32197301	+	670	-0.153	0
Ccne1	ENSMUSG00000002068	chr7	38883003	38892553	-	2662	-0.153	0
Cebpa	ENSMUSG00000034957	chr7	35904312	35906945	+	2634	-0.154	0
Rpl30	ENSMUSG00000058600	chr15	34370260	34373395	-	3020	-0.154	0
Nfatc4	ENSMUSG00000023411	chr14	56443632	56452780	+	4019	-0.154	0
BC005624	ENSMUSG00000026851	chr2	30828353	30837721	-	2624	-0.154	0
Ell	ENSMUSG00000070002	chr8	73063574	73116757	+	3085	-0.155	0
Apex2	ENSMUSG00000025269	chrX	146954062	147024411	-	8744	-0.155	0
Ltb4r1	ENSMUSG00000046908	chr14	56384799	56387331	+	1394	-0.156	0
Nynrin	ENSMUSG00000075592	chr14	56472873	56493572	+	7436	-0.156	0
Cdc25b	ENSMUSG00000027330	chr2	131012685	131024233	+	3728	-0.16	0

Dmwd	ENSMUSG00000030410	chr7	19661598	19668125	+	3206	-0.16	0
Ppap2a	ENSMUSG00000021759	chr13	113591102	113658089	+	1635	-0.16	0
Whsc2	ENSMUSG00000029111	chr5	34239585	34279062	-	4927	-0.161	0
Fitm2	ENSMUSG00000048486	chr2	163292115	163298365	-	3967	-0.161	0
Bok	ENSMUSG00000026278	chr1	95582271	95592339	+	1432	-0.164	0
P2rx7	ENSMUSG00000029468	chr5	123093920	123141441	+	5524	-0.165	0
Gm11948	ENSMUSG00000087055	chr11	3253576	3254914	-	539	-0.165	0
Nup214	ENSMUSG00000001855	chr2	31829956	31909495	+	9659	-0.166	0
Mapk14	ENSMUSG00000053436	chr17	28828287	28885349	+	4361	-0.166	0
Gnrh1	ENSMUSG00000015812	chr14	68363286	68367492	+	521	-0.166	0
Zdhhc3	ENSMUSG00000025786	chr9	122975278	123022323	-	11846	-0.167	0
Ephx4	ENSMUSG00000033805	chr5	107831755	107859054	+	1646	-0.167	0
4833418N02Rik	ENSMUSG00000085287	chr17	87674226	87682154	-	2762	-0.167	0
Enpp2	ENSMUSG00000022425	chr15	54670453	54751701	-	3473	-0.168	0
Tmem167b	ENSMUSG00000068732	chr3	108359343	108365384	-	2948	-0.168	0
A230050P20Rik	ENSMUSG00000038884	chr9	20673086	20678751	+	3417	-0.171	0
Pomp	ENSMUSG00000029649	chr5	148672204	148687354	+	734	-0.171	0
Srrm1	ENSMUSG00000028809	chr4	134876399	134909236	-	7368	-0.172	0
Hdgfrp3	ENSMUSG00000025104	chr7	89026137	89079359	-	9575	-0.172	0
5730409E04Rik	ENSMUSG00000073755	chr4	126287062	126291615	+	2891	-0.173	0
Nudc	ENSMUSG00000028851	chr4	133088457	133101911	-	1425	-0.173	0
Gtf3c1	ENSMUSG00000032777	chr7	132784469	132851294	-	6960	-0.173	0
Ccdc84	ENSMUSG00000043923	chr9	44218249	44226090	-	1156	-0.175	0
Tmc8	ENSMUSG00000050106	chr11	117643390	117654424	+	3056	-0.177	0
Mthfd1	ENSMUSG00000021048	chr12	77356219	77420807	+	3324	-0.179	0
Mchr1	ENSMUSG00000050164	chr15	81065929	81069393	+	2188	-0.179	0
Snx19	ENSMUSG00000031993	chr9	30234693	30274311	+	5762	-0.179	0
Per3	ENSMUSG00000028957	chr4	150377761	150418774	-	9608	-0.181	0
Zfp810	ENSMUSG00000066829	chr9	22082669	22094985	-	1386	-0.183	0
Gm3200	ENSMUSG00000078636	chr7	107909319	107910320	-	1002	-0.184	0
Scarb1	ENSMUSG00000037936	chr5	125757457	125821464	-	6291	-0.184	0
Ndufaf2	ENSMUSG00000068184	chr13	108842784	108948819	-	679	-0.184	0
Prdx5	ENSMUSG00000024953	chr19	6981187	6984596	-	2141	-0.185	0
Gins4	ENSMUSG00000031546	chr8	24337082	24348140	-	1364	-0.185	0
Pi4ka	ENSMUSG00000041720	chr16	17280444	17406407	-	11257	-0.185	0
Sec22b	ENSMUSG00000027879	chr3	97705113	97727199	+	3709	-0.189	0
Zfp319	ENSMUSG00000074140	chr8	97846914	97849163	-	2250	-0.189	0
Rassf3	ENSMUSG00000025795	chr10	120847406	120913306	-	3425	-0.19	0
Ap2a2	ENSMUSG00000002957	chr7	148748079	148818910	+	4639	-0.19	0
Zbtb20	ENSMUSG00000022708	chr16	42875994	43642715	+	30730	-0.191	0

D4Ertd22e	ENSMUSG00000040842	chr4	140668916	140695690	-	3327	-0.191	0
Tmem181a	ENSMUSG00000038141	chr17	6270475	6305783	+	1869	-0.192	0
Hdac4	ENSMUSG00000073617	chr1	93825362	93827753	-	2392	-0.192	0
Mycbp	ENSMUSG00000028647	chr4	123582075	123589512	+	2934	-0.192	0
Slc39a13	ENSMUSG00000002105	chr2	90901948	90910574	-	4869	-0.194	0
Psmc1	ENSMUSG00000021178	chr12	101348396	101361574	+	1518	-0.194	0
Cpsf3	ENSMUSG00000054309	chr12	21292158	21320917	+	2560	-0.196	0
Tnfaip8l2	ENSMUSG00000013707	chr3	94943443	94946282	-	1122	-0.196	0
Ankmy2	ENSMUSG00000036188	chr12	36883711	36923878	+	2446	-0.197	0
Tmem126b	ENSMUSG00000030614	chr7	97616704	97624511	-	3984	-0.197	0
Hspbap1	ENSMUSG00000022849	chr16	35770472	35828532	+	4590	-0.197	0
Aimp1	ENSMUSG00000028029	chr3	132323462	132346843	-	1122	-0.197	0
Gm9853	ENSMUSG00000051554	chr8	106875211	106876739	-	1529	-0.198	0
Crocc	ENSMUSG00000040860	chr4	140572552	140616465	-	8266	-0.199	0
4932415G12Rik	ENSMUSG00000086635	chr10	94136238	94151358	-	4135	-0.2	0
Nfe2	ENSMUSG00000058794	chr15	103078643	103088834	-	2988	-0.2	0
Tmem164	ENSMUSG00000047045	chrX	139115949	139278037	+	6851	-0.2	0
Cxcl16	ENSMUSG00000018920	chr11	70267485	70273486	-	3021	-0.201	0
Rpa3	ENSMUSG00000012483	chr6	8205936	8209173	-	1136	-0.202	0
Mat2a	ENSMUSG00000053907	chr6	72382793	72389552	-	3389	-0.202	0
2410004B18Rik	ENSMUSG00000036873	chr3	145600978	145607793	+	1937	-0.202	0
Isg20	ENSMUSG00000039236	chr7	86058310	86065248	+	1826	-0.202	0
X99384	ENSMUSG00000020092	chr10	60782405	60846271	-	4274	-0.203	0
Tagap1	ENSMUSG00000052031	chr17	7159314	7165505	-	2358	-0.203	0
Vasn	ENSMUSG00000039646	chr16	4639941	4650802	+	3007	-0.203	0
Wdr41	ENSMUSG00000042015	chr13	95746299	95793269	+	5030	-0.205	0
Scamp1	ENSMUSG00000021687	chr13	94971265	95055812	-	4582	-0.206	0
Cdk11b	ENSMUSG00000029062	chr4	154998963	155024047	+	3752	-0.206	0
Nat15	ENSMUSG00000005982	chr16	3872375	3904770	+	3765	-0.207	0
Rbp7	ENSMUSG00000028996	chr4	148823796	148829087	-	718	-0.208	0
Gys1	ENSMUSG00000003865	chr7	52690214	52711989	+	4789	-0.208	0
4930506M07Rik	ENSMUSG00000041362	chr19	59047848	59150559	-	4136	-0.21	0
Parn	ENSMUSG00000022685	chr16	13538057	13668263	-	2902	-0.21	0
Kif13b	ENSMUSG00000060012	chr14	65271439	65425133	+	5532	-0.21	0

6330409N04Rik	ENSMUSG00000021930	chr14	62150830	62175723	-	4002	-0.211	0
Med17	ENSMUSG00000031935	chr9	15064796	15084311	-	3899	-0.212	0
Htra3	ENSMUSG00000029096	chr5	35994682	36022431	-	3868	-0.212	0
Elmo1	ENSMUSG00000041112	chr13	20182488	20698397	+	3834	-0.212	0
Ccdc90a	ENSMUSG00000021371	chr13	43635791	43655495	-	2275	-0.213	0
E330009J07Rik	ENSMUSG00000037172	chr6	40351374	40386134	-	7511	-0.213	0
Trim21	ENSMUSG00000030966	chr7	109706436	109713983	-	2775	-0.214	0
Sall3	ENSMUSG00000024565	chr18	81163115	81183317	-	6886	-0.214	0
Zyx	ENSMUSG00000029860	chr6	42299827	42310145	+	3741	-0.214	0
Tlr5	ENSMUSG00000079164	chr1	184884919	184906175	+	3525	-0.215	0
AA986860	ENSMUSG00000042510	chr1	132628553	132641198	+	2908	-0.217	0
Taco1	ENSMUSG00000001983	chr11	105927375	105934926	+	1383	-0.217	0
2310003C23Rik	ENSMUSG00000027573	chr2	180444822	180453438	+	2464	-0.217	0
Arrdc4	ENSMUSG00000042659	chr7	75881931	75894127	-	3989	-0.217	0
Brd3	ENSMUSG00000026918	chr2	27301099	27363182	-	7336	-0.217	0
Ppp2r2d	ENSMUSG00000041769	chr7	146037762	146074740	+	5903	-0.218	0
Tollip	ENSMUSG00000025139	chr7	149060718	149104412	-	4333	-0.219	0
Smyd3	ENSMUSG00000055067	chr1	180882091	181448172	-	8360	-0.219	0
Porcn	ENSMUSG00000031169	chrX	7770974	7783651	-	3000	-0.22	0
Rab34	ENSMUSG00000002059	chr11	78001932	78005695	+	2385	-0.221	0
Yipf6	ENSMUSG00000047694	chrX	96131655	96144356	+	7149	-0.221	0
Casp6	ENSMUSG00000027997	chr3	129604343	129617021	+	1676	-0.223	0
Gm9887	ENSMUSG00000052673	chr12	70472822	70473445	-	624	-0.224	0
Hadhb	ENSMUSG00000059447	chr5	30481788	30511133	+	6595	-0.225	0
Usp36	ENSMUSG00000033909	chr11	118120965	118151558	-	7689	-0.225	0
Mettl2	ENSMUSG00000020691	chr11	104987739	105001708	+	3399	-0.225	0
Arhgef10	ENSMUSG00000071176	chr8	14911663	15001085	+	9210	-0.225	0
Chmp3	ENSMUSG00000053119	chr6	71493791	71532603	+	3250	-0.226	0
Crls1	ENSMUSG00000027357	chr2	132672402	132692521	+	2082	-0.226	0
Slc25a12	ENSMUSG00000027010	chr2	71109120	71205806	-	7342	-0.226	0
Angel2	ENSMUSG00000026634	chr1	192748991	192770841	+	5592	-0.227	0
Ccdc130	ENSMUSG00000004994	chr8	86781694	86794279	-	2694	-0.228	0
Pcgf2	ENSMUSG00000018537	chr11	97550137	97561811	-	3442	-0.228	0
Axin1	ENSMUSG00000024182	chr17	26275631	26332756	+	3830	-0.228	0
Hoxa13	ENSMUSG00000038203	chr6	52207693	52210874	-	2467	-0.229	0
Tctn2	ENSMUSG00000029386	chr5	125048758	125077658	+	8229	-0.23	0
Apoo-ps	ENSMUSG00000049233	chr13	108204221	108204817	-	597	-0.23	0

Mta2	ENSMUSG00000071646	chr19	9016365	9026793	+	4093	-0.231	0
Ogfrl1	ENSMUSG00000026158	chr1	23373263	23390014	-	4844	-0.231	0
Rab3il1	ENSMUSG00000024663	chr19	10076159	10112870	+	4543	-0.231	0
Gpn3	ENSMUSG00000029464	chr5	122821885	122832911	+	2508	-0.231	0
Zfp809	ENSMUSG00000057982	chr9	22030274	22047641	+	1287	-0.231	0
Lamtor3	ENSMUSG00000091512	chr3	137581490	137591729	+	1295	-0.232	0
Psmg2	ENSMUSG00000024537	chr18	67801253	67813816	+	1302	-0.233	0
Arhgap9	ENSMUSG00000040345	chr10	126759172	126766999	+	2324	-0.233	0
Pofut1	ENSMUSG00000046020	chr2	153067269	153095983	+	9912	-0.233	0
Slc7a6os	ENSMUSG00000033106	chr8	108724338	108734833	-	1525	-0.233	0
2610507I01Rik	ENSMUSG00000085882	chr11	59013338	59015887	-	1360	-0.234	0
Prdx4	ENSMUSG00000025289	chrX	151758461	151775297	-	2106	-0.234	0
Ddx51	ENSMUSG00000029504	chr5	111082470	111089515	+	5248	-0.234	0
Tgm1	ENSMUSG00000022218	chr14	56318846	56332329	-	3006	-0.234	0
Tram1	ENSMUSG00000025935	chr1	13554783	13579945	-	2888	-0.237	0
Nadk	ENSMUSG00000029063	chr4	154936487	154965110	+	4283	-0.237	0
Tmem126a	ENSMUSG00000030615	chr7	97599210	97605739	-	1018	-0.238	0
Jmjd6	ENSMUSG00000056962	chr11	116698746	116704763	-	4698	-0.239	0
Frmd8	ENSMUSG00000024816	chr19	5849702	5875274	-	5082	-0.239	0
Lonp1	ENSMUSG00000041168	chr17	56753724	56766326	-	2963	-0.239	0
Nol9	ENSMUSG00000028948	chr4	151413430	151435603	+	4956	-0.24	0
Prkce	ENSMUSG00000045038	chr17	86567125	87057259	+	7261	-0.24	0
Gnas	ENSMUSG00000027523	chr2	174109821	174172245	+	11186	-0.241	0
Sel1l	ENSMUSG00000020964	chr12	93044483	93087597	-	8555	-0.241	0
Rab11fip1	ENSMUSG00000031488	chr8	28249245	28285120	-	7967	-0.241	0
Rnf8	ENSMUSG00000090083	chr17	29751735	29840304	+	8157	-0.241	0
Fah	ENSMUSG00000030630	chr7	91733669	91755232	-	2103	-0.242	0
1600014C10Rik	ENSMUSG00000054676	chr7	38968236	38982582	+	3448	-0.242	0
Nckap5l	ENSMUSG00000023009	chr15	99252466	99288179	-	5002	-0.243	0
Cldn15	ENSMUSG00000001739	chr5	137442486	137451728	+	2124	-0.244	0
Bcl7a	ENSMUSG00000029438	chr5	123793843	123824091	+	8580	-0.245	0
Hdgf	ENSMUSG00000004897	chr3	87710243	87720054	+	2873	-0.245	0
Usp10	ENSMUSG00000031826	chr8	122434260	122481460	+	5456	-0.246	0
Gsg2	ENSMUSG00000050107	chr11	72948987	72951796	-	2810	-0.247	0
Anapc5	ENSMUSG00000029472	chr5	123237478	123271348	-	2750	-0.247	0
Eif3c	ENSMUSG00000030738	chr7	133690426	133709880	-	2883	-0.247	0
Pcbp2	ENSMUSG00000056851	chr15	102301063	102330490	+	2868	-0.248	0
Rgs14	ENSMUSG00000052087	chr13	55471093	55486048	+	2490	-0.248	0
Rbms2	ENSMUSG00000040043	chr10	127566526	127617353	-	5609	-0.249	0
Syt7	ENSMUSG00000024743	chr19	10463580	10527671	+	6966	-0.249	0

Taok2	ENSMUSG00000059981	chr7	134009192	134028217	-	9247	-0.251	0
Pdlim7	ENSMUSG00000021493	chr13	55597156	55615037	-	4849	-0.253	0
Zfp740	ENSMUSG00000046897	chr15	102034079	102046037	+	4822	-0.253	0
Cyp2f2	ENSMUSG00000052974	chr7	27904928	27918679	+	2025	-0.253	0
Ensa	ENSMUSG00000038619	chr3	95428902	95436024	+	2947	-0.253	0
Gm6158	ENSMUSG00000090381	chr14	24889296	24889859	+	564	-0.255	0
Gm7676	ENSMUSG00000068631	chr8	13896311	13896631	+	321	-0.256	0
Ube2e2	ENSMUSG00000058317	chr14	19406089	19726781	-	4671	-0.257	0
Polg	ENSMUSG00000039176	chr7	86593677	86611248	-	6845	-0.257	0
Atf6	ENSMUSG00000026663	chr1	172634588	172797902	-	7463	-0.257	0
Ighm	ENSMUSG00000076617	chr12	114659078	114660941	-	1367	-0.258	0
Tctn1	ENSMUSG00000038593	chr5	122691296	122714469	-	4856	-0.259	0
Tubgcp2	ENSMUSG00000025474	chr7	147181854	147222289	-	3125	-0.259	0
Tmem170	ENSMUSG00000031953	chr8	114388798	114400575	-	1943	-0.259	0
Prepl	ENSMUSG00000024127	chr17	85462817	85489607	-	3830	-0.26	0
Scrn3	ENSMUSG00000008226	chr2	73150658	73175875	+	4815	-0.268	0
Grrp1	ENSMUSG00000050105	chr4	133807025	133810021	-	1275	-0.269	0
Polr1b	ENSMUSG00000027395	chr2	128926731	128952330	+	5805	-0.27	0
Smarca4	ENSMUSG00000032187	chr9	21420613	21508674	+	6475	-0.271	0
Utp23	ENSMUSG00000022313	chr15	51708975	51716160	+	2992	-0.271	0
Sema4d	ENSMUSG00000021451	chr13	51796615	51889116	-	6449	-0.272	0
Slc9a9	ENSMUSG00000031129	chr9	94570328	95130864	+	5679	-0.274	0
Eif1	ENSMUSG00000035530	chr11	100181199	100183410	+	2098	-0.275	0
Irgq	ENSMUSG00000041037	chr7	25315667	25323618	+	6123	-0.275	0
Rfc3	ENSMUSG00000033970	chr5	152445331	152453817	-	3611	-0.275	0
Efemp1	ENSMUSG00000020467	chr11	28753204	28826743	+	2037	-0.275	0
Fgfrl1	ENSMUSG00000008090	chr5	109123248	109135969	+	2656	-0.276	0
Sf3b2	ENSMUSG00000024853	chr19	5273923	5295455	-	3231	-0.276	0
Fbxo33	ENSMUSG00000035329	chr12	60301642	60320712	-	4466	-0.276	0
Ift122	ENSMUSG00000030323	chr6	115803488	115876717	+	7953	-0.276	0
Rgp1	ENSMUSG00000028468	chr4	43591587	43600359	+	6199	-0.277	0
Folr2	ENSMUSG00000032725	chr7	108988512	108993831	-	1057	-0.277	0
Cbx1	ENSMUSG00000018666	chr11	96650441	96669954	+	4273	-0.277	0
Tasp1	ENSMUSG00000039033	chr2	139659216	139892541	-	3652	-0.279	0
Dnajb12	ENSMUSG00000020109	chr10	59342304	59362050	+	3369	-0.28	0
Tgoln1	ENSMUSG00000056429	chr6	72558426	72566994	-	5013	-0.282	0
Acp5	ENSMUSG00000001348	chr9	21931175	21940190	-	1612	-0.282	0
Mrpl13	ENSMUSG00000022370	chr15	55365649	55389303	-	7936	-0.283	0
Mst1r	ENSMUSG00000032584	chr9	107809220	107822714	+	4706	-0.285	0
Tnip1	ENSMUSG00000020400	chr11	54724289	54776419	-	3537	-0.285	0
Zscan29	ENSMUSG00000050619	chr2	120984009	120996861	-	7169	-0.285	0

Lrig3	ENSMUSG00000020105	chr10	125403275	125452415	+	4050	-0.287		0
Prr12	ENSMUSG00000046574	chr7	52283077	52308251	-	6879	-0.288		0
Prmt6	ENSMUSG00000049300	chr3	110049027	110053916	-	4197	-0.289		0
Gas1	ENSMUSG00000052957	chr13	60275766	60278726	-	2961	-0.289		0
Urb1	ENSMUSG00000039929	chr16	90751772	90810658	-	8190	-0.29		0
2310030N02Rik	ENSMUSG00000050323	chr4	10978192	11003352	-	1235	-0.291		0
Gclm	ENSMUSG00000028124	chr3	121948475	121972902	+	5543	-0.291		0
Rtel1	ENSMUSG00000038685	chr2	181054444	181091321	+	6616	-0.291		0
Vps29	ENSMUSG00000029462	chr5	122804378	122814993	+	3089	-0.292		0
Ebi3	ENSMUSG00000003206	chr17	56092046	56096444	+	1170	-0.296		0
Icosl	ENSMUSG00000000732	chr10	77532105	77546000	+	2842	-0.296		0
Gm13476	ENSMUSG00000052248	chr2	44966658	44969607	+	1388	-0.296		0
Arsg	ENSMUSG00000020604	chr11	109334688	109434644	+	3906	-0.298		0
Irs1	ENSMUSG00000055980	chr1	82229676	82287991	-	9144	-0.301		0
Pnrc1	ENSMUSG00000040128	chr4	33332398	33377138	-	2095	-0.302		0
Zfhx3	ENSMUSG00000038872	chr8	111238544	111485536	+	16439	-0.302		0
Cyp2b10	ENSMUSG00000030483	chr7	26682639	26711643	+	2112	-0.305		0
Map3k12	ENSMUSG00000023050	chr15	102328080	102347495	-	8986	-0.306		0
Ccdc163	ENSMUSG00000028689	chr4	116381114	116423811	+	2519	-0.306		0
Ap4b1	ENSMUSG00000032952	chr3	103613443	103625948	+	2907	-0.307		0
Fes	ENSMUSG00000053158	chr7	87522641	87532832	-	2765	-0.31		0
Cad	ENSMUSG00000013629	chr5	31357153	31380852	+	8164	-0.311		0
Lins	ENSMUSG00000053091	chr7	73834775	73862140	+	5874	-0.312		0
Ptcd2	ENSMUSG00000021650	chr13	100089604	100114633	-	1675	-0.312		0
Ezr	ENSMUSG00000052397	chr17	6942390	6987133	-	3290	-0.312		0
Cwc25	ENSMUSG00000018541	chr11	97606795	97627870	-	5053	-0.313		0
B3gnt1	ENSMUSG00000047379	chr19	5038826	5041134	+	2035	-0.313		0
Phc2	ENSMUSG00000028796	chr4	128331982	128430125	+	7030	-0.315		0
E130311K13Rik	ENSMUSG00000048581	chr3	63718606	63733394	-	2398	-0.319		0
Gimap1	ENSMUSG00000090019	chr6	48668606	48702599	+	2124	-0.319		0
Pawr	ENSMUSG00000035873	chr10	107769245	107851447	+	1828	-0.319		0
Bcl2l1	ENSMUSG00000007659	chr2	152593052	152657464	-	4734	-0.322		0
Zfp259	ENSMUSG00000032078	chr9	46081147	46090726	+	3583	-0.322		0
Gna12	ENSMUSG00000000149	chr5	141234362	141306385	-	3416	-0.324		0
U2af1	ENSMUSG00000061613	chr17	31784027	31795699	-	974	-0.324		0
Gcn1l1	ENSMUSG00000041638	chr5	116015263	116072665	+	15919	-0.324		0
Vhl	ENSMUSG00000033933	chr6	113573953	113581627	+	2997	-0.325		0
Polr3b	ENSMUSG00000034453	chr10	84085182	84189922	+	4829	-0.329		0
Mrps15	ENSMUSG00000028861	chr4	125724169	125732776	+	1693	-0.331		0
Wscd1	ENSMUSG00000020811	chr11	71563422	71603149	+	4287	-0.332		0

Zscan2	ENSMUSG00000038797	chr7	88005806	88021402	+	2712	-0.333	0
6030446N20Rik	ENSMUSG00000049411	chr18	12139812	12279989	-	3336	-0.333	0
Spata2	ENSMUSG00000047030	chr2	167306633	167318387	-	4510	-0.334	0
Naca	ENSMUSG00000061315	chr10	127472402	127485693	+	7034	-0.335	0
Chmp7	ENSMUSG00000034190	chr14	70118055	70132296	-	1356	-0.336	0
Gnpat	ENSMUSG00000031985	chr8	127386933	127413957	+	3587	-0.336	0
Bet1	ENSMUSG00000032757	chr6	4026899	4036972	-	3656	-0.337	0
BC034090	ENSMUSG00000033722	chr1	157059603	157079870	-	3797	-0.337	0
Magt1	ENSMUSG00000031232	chrX	103163423	103207245	-	4672	-0.339	0
Tfe3	ENSMUSG00000000134	chrX	7339686	7352328	+	9261	-0.339	0
Bcl2l13	ENSMUSG00000009112	chr6	120786248	120842860	+	6933	-0.342	0
Zfp747	ENSMUSG00000054381	chr7	134516078	134519564	-	2657	-0.344	0
Pole4	ENSMUSG00000030042	chr6	82568986	82655359	-	2509	-0.344	0
Nacc1	ENSMUSG00000001910	chr8	87194382	87211761	-	4300	-0.345	0
Trim32	ENSMUSG00000051675	chr4	65266020	65277272	+	3229	-0.345	0
Sephs2	ENSMUSG00000049091	chr7	134416075	134417433	-	1359	-0.345	0
Nbeal2	ENSMUSG00000056724	chr9	110527293	110556665	-	9554	-0.345	0
Ruvbl1	ENSMUSG00000030079	chr6	88415403	88447566	+	1899	-0.346	0
Chd8	ENSMUSG00000053754	chr14	52817826	52857466	-	12599	-0.348	0
Tmx2	ENSMUSG00000050043	chr2	84511473	84519297	-	2330	-0.349	0
Llgl1	ENSMUSG00000020536	chr11	60513225	60527688	+	4862	-0.349	0
Rdh13	ENSMUSG00000008435	chr7	4376372	4397251	-	6595	-0.349	0
Plekhm1	ENSMUSG00000034247	chr11	103226402	103273978	-	5132	-0.35	0
Ahcy	ENSMUSG00000027597	chr2	154885046	154900233	-	2805	-0.35	0
Pnpla6	ENSMUSG00000004565	chr8	3515384	3544266	+	4496	-0.352	0
Taf11	ENSMUSG00000024218	chr17	28038067	28046187	-	2290	-0.352	0
Btrc	ENSMUSG00000025217	chr19	45438224	45607831	+	6323	-0.353	0
Med8	ENSMUSG00000006392	chr4	118081942	118088387	+	3001	-0.353	0
Sbf1	ENSMUSG00000036529	chr15	89118667	89145742	-	6826	-0.354	0
Tmem231	ENSMUSG00000031951	chr8	114435918	114457691	-	2887	-0.355	0
Gltp	ENSMUSG00000011884	chr5	115119412	115140993	-	1757	-0.355	0
Mgp	ENSMUSG00000030218	chr6	136820957	136824326	-	598	-0.355	0
Mast3	ENSMUSG00000031833	chr8	73302016	73320845	-	5549	-0.356	0
Tm2d1	ENSMUSG00000028563	chr4	98022061	98049997	-	1124	-0.356	0
Tshz2	ENSMUSG00000047907	chr2	169458513	169897316	+	7817	-0.357	0
Pgrmc2	ENSMUSG00000049940	chr3	40870248	40886968	-	3242	-0.357	0
Necap1	ENSMUSG00000030327	chr6	122824492	122838952	+	2400	-0.357	0
Ifitm2	ENSMUSG00000060591	chr7	148140736	148141882	-	651	-0.359	0
Tubg2	ENSMUSG00000045007	chr11	101017221	101023101	+	1754	-0.359	0
Unk	ENSMUSG00000020770	chr11	115891636	115922528	+	4438	-0.361	0
Apoa5	ENSMUSG00000032079	chr9	46076716	46080002	+	2518	-0.361	0
AL593857.1	ENSMUSG00000090625	chr2	174155820	174172213	-	1484	-0.362	0

Foxs1	ENSMUSG00000074676	chr2	152757634	152758944	-	1311	-0.362	0
Slc25a5	ENSMUSG00000016319	chrX	34335646	34338802	+	1646	-0.363	0
Adpgk	ENSMUSG00000025236	chr9	59139379	59166197	+	2554	-0.363	0
Tnfrsf1a	ENSMUSG00000030341	chr6	125299380	125312502	+	2598	-0.365	0
Nacc2	ENSMUSG00000026932	chr2	25911055	25978740	-	7815	-0.365	0
Larp7	ENSMUSG00000027968	chr3	127239872	127256266	-	1880	-0.366	0
Tmed10	ENSMUSG00000021248	chr12	86681564	86715667	-	3526	-0.367	0
Gemin5	ENSMUSG00000037275	chr11	57933504	57982041	-	6618	-0.368	0
1110008 L16Rik	ENSMUSG00000021023	chr12	56403624	56483478	+	3117	-0.368	0
Rpl21	ENSMUSG00000041453	chr5	147644466	147648608	+	3400	-0.369	0
Cnnm3	ENSMUSG00000001138	chr1	36568712	36585082	+	5558	-0.37	0
Arl3	ENSMUSG00000025035	chr19	46605599	46647575	-	822	-0.371	0
Sft2d2	ENSMUSG00000040848	chr1	167104472	167124564	-	5526	-0.373	0
Smox	ENSMUSG00000027333	chr2	131317232	131351658	+	3894	-0.373	0
Arih2	ENSMUSG00000064145	chr9	108505273	108551711	-	3924	-0.374	0
Mkrn1	ENSMUSG00000029922	chr6	39347803	39370461	-	4439	-0.374	0
BC0176 47	ENSMUSG00000037750	chr11	77908162	77970202	+	3490	-0.374	0
Kank1	ENSMUSG00000032702	chr19	25311465	25508987	+	6167	-0.375	0
Wdr5b	ENSMUSG00000034379	chr16	36041276	36043059	+	1784	-0.375	0
Dnajc25	ENSMUSG00000070972	chr4	59007934	59038445	+	4542	-0.376	0
Atxn7l3b	ENSMUSG00000074748	chr10	112362494	112366082	-	3589	-0.376	0
Por	ENSMUSG00000005514	chr5	136145903	136211196	+	4021	-0.377	0
Bmyc	ENSMUSG00000049086	chr2	25562399	25563237	+	839	-0.377	0
Tk1	ENSMUSG00000025574	chr11	117676840	117687406	-	5198	-0.378	0
Slc25a4 5	ENSMUSG00000024818	chr19	5877808	5885766	+	2650	-0.378	0
Fam19a 5	ENSMUSG00000054863	chr15	87374904	87589766	+	2705	-0.379	0
Parm1	ENSMUSG00000034981	chr5	91946641	92055920	+	5068	-0.379	0
Enox2	ENSMUSG00000031109	chrX	46362884	46641436	-	4356	-0.379	0
Tmem1 58	ENSMUSG00000054871	chr9	123168176	123169907	-	1732	-0.38	0
Fam69b	ENSMUSG00000036186	chr2	26483977	26492017	+	2476	-0.38	0
Mcm7	ENSMUSG00000029730	chr5	138605811	138613650	-	4426	-0.382	0
Zfp410	ENSMUSG00000042472	chr12	85658064	85684673	+	2370	-0.382	0
2010012 O05Rik	ENSMUSG00000062376	chr19	46764396	46777872	+	2176	-0.383	0
Tmem1 73	ENSMUSG00000024349	chr18	35893332	35900208	-	2302	-0.383	0
Lfng	ENSMUSG00000029570	chr5	141083295	141091497	+	2282	-0.383	0
Gm1686 7	ENSMUSG00000085092	chr14	69981561	70162426	-	3368	-0.384	0

Mtch2	ENSMUSG00000027282	chr2	90687312	90706967	+	8490	-0.384		0
Ap3d1	ENSMUSG00000020198	chr10	80169723	80204956	-	4730	-0.385		0
Src	ENSMUSG00000027646	chr2	157244180	157297598	+	4371	-0.386		0
Npff	ENSMUSG00000023052	chr15	102354274	102355052	-	442	-0.387		0
Srp9	ENSMUSG00000026511	chr1	184054868	184062538	+	1380	-0.387		0
Zfp930	ENSMUSG00000059897	chr8	71750242	71752879	+	1323	-0.387		0
Esyt1	ENSMUSG00000025366	chr10	127947304	127962926	-	3827	-0.387		0
1500003 O03Rik	ENSMUSG00000014077	chr2	119373433	119412763	+	3499	-0.388		0
5730494 M16Rik	ENSMUSG00000024269	chr18	25285724	25327508	-	6840	-0.39		0
Ephb3	ENSMUSG00000005958	chr16	21204828	21223378	+	6289	-0.39		0
B4galt2	ENSMUSG00000028541	chr4	117541865	117556092	-	5031	-0.39		0
Inpp5b	ENSMUSG00000028894	chr4	124419094	124478755	+	4657	-0.391		0
Hoxd9	ENSMUSG00000043342	chr2	74535737	74538265	+	2183	-0.393		0
Arhgef1	ENSMUSG00000040940	chr7	25687931	25711611	+	7495	-0.393		0
Pdcd7	ENSMUSG00000041837	chr9	65193875	65207450	+	2455	-0.394		0
D93001 5E06Rik	ENSMUSG00000033767	chr3	83702131	83844083	-	4978	-0.394		0
Chd3	ENSMUSG00000018474	chr11	69156775	69182928	-	8463	-0.396		0
Sftpb	ENSMUSG00000056370	chr6	72254619	72262926	+	1131	-0.396		0
Tyms	ENSMUSG00000025747	chr5	30384742	30400157	-	6089	-0.396		0
Lsp1	ENSMUSG00000018819	chr7	149646714	149680772	+	5174	-0.397		0
Ube2h	ENSMUSG00000039159	chr6	30161289	30254539	-	4780	-0.397		0
Cryz	ENSMUSG00000028199	chr3	154259675	154286146	+	2968	-0.397		0
Smarcb 1	ENSMUSG00000000902	chr10	75359514	75384362	-	8133	-0.398		0
Grk6	ENSMUSG00000074886	chr13	55546695	55562286	+	3241	-0.398		0
Zfp251	ENSMUSG00000022526	chr15	76682578	76701865	-	3497	-0.398		0
Lpin3	ENSMUSG00000027412	chr2	160706406	160731738	+	3694	-0.399		0
Znfx1	ENSMUSG00000039501	chr2	166861293	166888515	-	8189	-0.399		0
Daxx	ENSMUSG00000002307	chr17	34046359	34052534	+	3058	-0.399		0
Casc3	ENSMUSG00000078676	chr11	98666219	98695128	+	4303	-0.399		0
Zc3h12a	ENSMUSG00000042677	chr4	124795667	124805084	-	2882	-0.399		0
Mylip	ENSMUSG00000038175	chr13	45485111	45507388	+	3062	-0.4		0
Prr14	ENSMUSG00000030822	chr7	134603125	134620273	+	3456	-0.401		0
Nol3	ENSMUSG00000014776	chr8	107800347	107805839	+	2869	-0.401		0
Sh3bp4	ENSMUSG00000036206	chr1	90966990	91051643	+	5112	-0.402		0
Casc4	ENSMUSG00000060227	chr2	121692706	121761956	+	5858	-0.403		0
Rnf7	ENSMUSG00000051234	chr9	96371360	96379094	-	1324	-0.404		0
Foxo4	ENSMUSG00000042903	chrX	98449867	98456212	+	3485	-0.404		0
Pi4k2a	ENSMUSG00000025178	chr19	42164925	42196708	+	3771	-0.405		0
Blmh	ENSMUSG00000020840	chr11	76738311	76800881	+	5891	-0.405		0
Lsm3	ENSMUSG00000090846	chr6	91466029	91472614	+	631	-0.405		0

Ptk7	ENSMUSG00000023972	chr17	46701400	46766453	-	4709	-0.407	0
Cd200	ENSMUSG00000022661	chr16	45382248	45409166	-	3321	-0.408	0
0610037 P05Rik	ENSMUSG00000022677	chr16	14299337	14317468	-	1465	-0.408	0
Acot2	ENSMUSG00000021226	chr12	85328811	85334823	+	2196	-0.409	0
Myl12a	ENSMUSG00000024048	chr17	71342996	71352218	-	3201	-0.409	0
Mertk	ENSMUSG00000014361	chr2	128524692	128628630	+	4336	-0.409	0
Cxxc5	ENSMUSG00000046668	chr18	35989472	36021341	+	2271	-0.41	0
Syf2	ENSMUSG00000028821	chr4	134486813	134493463	+	2043	-0.41	0
Ppp1r1b	ENSMUSG00000061718	chr11	98209718	98219110	+	2928	-0.41	0
Arl6ip5	ENSMUSG00000035199	chr6	97160683	97183309	+	1442	-0.411	0
Aldh9a1	ENSMUSG00000026687	chr1	169280122	169298661	+	2833	-0.411	0
Tbc1d5	ENSMUSG00000023923	chr17	50872452	51318674	-	5679	-0.412	0
Fundc2	ENSMUSG00000031198	chrX	72627738	72641798	+	2785	-0.413	0
Psma2	ENSMUSG00000015671	chr13	14705521	14719674	+	2668	-0.413	0
Hibadh	ENSMUSG00000029776	chr6	52496222	52590383	-	2226	-0.414	0
Plcg2	ENSMUSG00000034330	chr8	120022191	120159040	+	4296	-0.414	0
Ube2z	ENSMUSG00000014349	chr11	95908746	95926702	-	3997	-0.414	0
Idh1	ENSMUSG00000025950	chr1	65205190	65233053	-	2530	-0.416	0
Ncoa1	ENSMUSG00000020647	chr12	4247363	4484060	-	7328	-0.417	0
Trim68	ENSMUSG00000073968	chr7	109826094	109835840	-	2389	-0.417	0
Rhpn2	ENSMUSG00000030494	chr7	36119194	36177304	+	3873	-0.418	0
Exosc2	ENSMUSG00000039356	chr2	31526235	31536870	+	2160	-0.419	0
Ssbp4	ENSMUSG00000070003	chr8	73121390	73132213	-	1474	-0.419	0
Zdhhc8	ENSMUSG00000060166	chr16	18220847	18235229	-	4867	-0.42	0
Fam53b	ENSMUSG00000030956	chr7	139903765	140005569	-	6911	-0.421	0
Eif4e3	ENSMUSG00000093661	chr6	99575129	99616765	-	2515	-0.421	0
Shc1	ENSMUSG00000042626	chr3	89222365	89233937	+	5633	-0.422	0
4930427 A07Rik	ENSMUSG00000037466	chr12	114394632	114413736	+	3646	-0.422	0
Nub1	ENSMUSG00000028954	chr5	24191633	24216373	+	3455	-0.422	0
Inadl	ENSMUSG00000061859	chr4	98062476	98386294	+	14870	-0.423	0
Uhrf1bp 1	ENSMUSG00000039512	chr17	27993435	28036985	+	8708	-0.423	0
Tgif2	ENSMUSG00000062175	chr2	156665813	156681306	+	3818	-0.426	0
Alg8	ENSMUSG00000035704	chr7	104520116	104540663	+	2795	-0.427	0
Foxa1	ENSMUSG00000035451	chr12	58641618	58647108	-	3188	-0.427	0
Alas2	ENSMUSG00000025270	chrX	146981918	147005181	+	2403	-0.427	0
Med26	ENSMUSG00000045248	chr8	75018457	75072209	-	3040	-0.428	0
Mamld1	ENSMUSG00000059401	chrX	68303431	68409231	+	4799	-0.428	0
Safb	ENSMUSG00000071054	chr17	56724405	56745717	+	3195	-0.429	0
Scd1	ENSMUSG00000037071	chr19	44468941	44482199	-	4836	-0.431	0
Golgb1	ENSMUSG00000034243	chr16	36875226	36933171	+	12639	-0.431	0

Crtc2	ENSMUSG00000027936	chr3	90058085	90068047	+	4094	-0.431		0
Dhx58	ENSMUSG00000017830	chr11	100556018	100565585	-	3452	-0.432		0
Bace2	ENSMUSG00000040605	chr16	97578335	97660619	+	3736	-0.432		0
H2-Q4	ENSMUSG00000035929	chr17	35516562	35522235	+	2670	-0.433		0
Cmtm7	ENSMUSG00000032436	chr9	114665955	114690963	-	988	-0.433		0
Ttc30b	ENSMUSG00000075273	chr2	75773920	75776519	-	2600	-0.434		0
Sftpa1	ENSMUSG00000021789	chr14	41945071	41949741	+	2860	-0.434		0
Gart	ENSMUSG00000022962	chr16	91621431	91647197	-	3992	-0.435		0
Prune	ENSMUSG00000015711	chr3	95057596	95085998	-	3618	-0.435		0
Arhgap1	ENSMUSG00000027247	chr2	91490017	91512483	+	4275	-0.436		0
Pdhb	ENSMUSG00000021748	chr14	8998505	9005506	-	1485	-0.436		0
Meis3	ENSMUSG00000041420	chr7	16760439	16771859	+	2755	-0.436		0
Tspyl3	ENSMUSG00000074671	chr2	153048116	153051177	-	3062	-0.436		0
Ak2	ENSMUSG00000028792	chr4	128669202	128688773	+	2140	-0.438		0
Gng5	ENSMUSG00000068523	chr3	146162798	146168536	+	2738	-0.438		0
Arpc3	ENSMUSG00000029465	chr5	122841887	122856188	+	2581	-0.438		0
Fbf1	ENSMUSG00000020776	chr11	116003599	116029480	-	7751	-0.439		0
Prkcd	ENSMUSG00000021948	chr14	31408542	31439396	-	4983	-0.441		0
Mfap3	ENSMUSG00000020522	chr11	57332166	57347317	+	5801	-0.441		0
Mum1	ENSMUSG00000020156	chr10	79689179	79706648	+	10361	-0.442		0
1500011H22Rik	ENSMUSG00000029463	chr5	122814589	122822373	-	1388	-0.442		0
Pacrgl	ENSMUSG00000029089	chr5	48763631	48779995	+	1464	-0.443		0
Wdr77	ENSMUSG00000000561	chr3	105762287	105787518	+	6644	-0.444		0
Prrc2a	ENSMUSG00000024393	chr17	35286021	35301842	-	7635	-0.444		0
Numa1	ENSMUSG00000066306	chr7	109082688	109163473	+	7339	-0.444		0
Reck	ENSMUSG00000028476	chr4	43888402	43957678	+	4779	-0.446		0
Dcp1b	ENSMUSG00000041477	chr6	119125270	119171632	+	6338	-0.446		0
Gm4840	ENSMUSG00000090893	chr18	46515047	46516621	-	1575	-0.446		0
2310008H09Rik	ENSMUSG00000030980	chr7	125985736	125999768	-	5871	-0.447		0
Tti2	ENSMUSG00000031577	chr8	32260788	32275174	+	5076	-0.447		0
Vps33b	ENSMUSG00000030534	chr7	87414535	87436465	+	3343	-0.447		0
Snip1	ENSMUSG00000050213	chr4	124743916	124751286	+	2428	-0.447		0
Airn	ENSMUSG00000078247	chr17	12934177	13052988	+	3583	-0.448		0
Cdk9	ENSMUSG00000009555	chr2	32561304	32568596	-	4611	-0.449		0
Usp18	ENSMUSG00000030107	chr6	121195924	121220934	+	1770	-0.449		0
Tomm34	ENSMUSG00000018322	chr2	163879276	163896905	-	3040	-0.449		0
Mrpl3	ENSMUSG00000032563	chr9	104955569	104979796	+	3569	-0.449		0
Pacs1	ENSMUSG00000024855	chr19	5133688	5273119	-	4378	-0.449		0
Zswim7	ENSMUSG00000014243	chr11	62080726	62094877	-	732	-0.45		0
Tmem69	ENSMUSG00000055900	chr4	116224238	116228541	-	2069	-0.45		0

Plod3	ENSMUSG00000004846	chr5	137462889	137472518	+	4519	-0.45	0
Med31	ENSMUSG00000020801	chr11	72025226	72029094	-	1730	-0.45	0
Aga	ENSMUSG00000031521	chr8	54597080	54608776	+	1266	-0.451	0
Hsdl2	ENSMUSG00000028383	chr4	59594435	59631565	+	2799	-0.451	0
Dtwd1	ENSMUSG00000023330	chr2	125977877	125991015	+	1350	-0.451	0
Camk2g	ENSMUSG00000021820	chr14	21554097	21613310	-	4825	-0.454	0
Maml3	ENSMUSG00000061143	chr3	51491533	51908813	-	6536	-0.454	0
Ambra1	ENSMUSG00000040506	chr2	91570291	91759006	+	6143	-0.454	0
Idua	ENSMUSG00000033540	chr5	109089350	109113576	+	4204	-0.455	0
Ccdc15 7	ENSMUSG00000051427	chr11	4041126	4060296	-	5589	-0.456	0
Psmd5	ENSMUSG00000026869	chr2	34707608	34726459	-	3426	-0.456	0
Thoc3	ENSMUSG00000025872	chr13	54560198	54570210	-	2428	-0.458	0
Atxn7l3	ENSMUSG00000059995	chr11	102150614	102157945	-	4121	-0.459	0
Elp3	ENSMUSG00000022031	chr14	66149292	66211837	-	2770	-0.459	0
Lysmd4	ENSMUSG00000043831	chr7	74367494	74373342	+	2898	-0.46	0
Samd10	ENSMUSG00000038605	chr2	181329923	181333917	-	2448	-0.462	0
Slc29a2	ENSMUSG00000024891	chr19	5024006	5031971	+	2472	-0.462	0
Tmem1 89	ENSMUSG00000090213	chr2	167468108	167515322	-	3265	-0.463	0
Mllt6	ENSMUSG00000038437	chr11	97524728	97546777	+	7451	-0.464	0
Zscan22	ENSMUSG00000054715	chr7	13483164	13494432	+	3501	-0.464	0
Ip6k2	ENSMUSG00000032599	chr9	108686127	108708664	+	2845	-0.464	0
Kifc3	ENSMUSG00000031788	chr8	97623732	97666440	-	3758	-0.465	0
Tcf7	ENSMUSG00000000782	chr11	52065873	52096681	-	2790	-0.465	0
Cldn5	ENSMUSG00000041378	chr16	18776940	18778353	+	1414	-0.465	0
Gsta3	ENSMUSG00000025934	chr1	21230670	21255442	+	1392	-0.466	0
Atp2b4	ENSMUSG00000026463	chr1	135599255	135697618	-	9027	-0.466	0
Plrg1	ENSMUSG00000027998	chr3	82859444	82876277	+	2816	-0.466	0
E2f4	ENSMUSG00000014859	chr8	107821563	107829269	+	1992	-0.467	0
Nmi	ENSMUSG00000026946	chr2	51804007	51829014	-	2001	-0.467	0
Fbrs	ENSMUSG00000042423	chr7	134628735	134635027	+	2650	-0.467	0
C2cd2l	ENSMUSG00000032120	chr9	44117320	44128365	-	4397	-0.468	0
Snhg7	ENSMUSG00000091299	chr2	26492697	26495764	-	2465	-0.468	0
Fhod1	ENSMUSG00000014778	chr8	107853063	107871853	-	4666	-0.469	0
Pigp	ENSMUSG00000022940	chr16	94580370	94592622	-	1888	-0.469	0
Dhrs3	ENSMUSG00000066026	chr4	144482730	144518112	+	2927	-0.47	0
Ociad1	ENSMUSG00000029152	chr5	73684033	73705316	+	4132	-0.47	0
Ak3	ENSMUSG00000024782	chr19	29095323	29122451	-	3120	-0.471	0
Abca2	ENSMUSG00000026944	chr2	25284223	25304060	+	8351	-0.472	0
Lmna	ENSMUSG00000028063	chr3	88285070	88313878	-	4252	-0.472	0
Mtmr4	ENSMUSG00000018401	chr11	87405664	87429804	+	6240	-0.473	0
Galm	ENSMUSG00000035473	chr17	80526811	80584441	+	2476	-0.473	0

Gm10524	ENSMUSG00000073519	chr18	82862341	82875566	+	1984	-0.473	0
1700052N19Rik	ENSMUSG00000061759	chr10	5891400	5914074	-	8243	-0.473	0
Uqcc	ENSMUSG00000005882	chr2	155672630	155756046	-	6044	-0.474	0
Cdan1	ENSMUSG00000027284	chr2	120541890	120675864	-	11421	-0.474	0
Mov10	ENSMUSG00000002227	chr3	104597754	104621481	-	4223	-0.474	0
Rhobtb2	ENSMUSG00000022075	chr14	70184797	70212310	-	5511	-0.474	0
Mbd6	ENSMUSG00000025409	chr10	126719012	126726074	-	4455	-0.474	0
Unc13b	ENSMUSG00000028456	chr4	43071825	43277745	+	13507	-0.475	0
Top3b	ENSMUSG00000022779	chr16	16870829	16893083	+	8340	-0.477	0
Ppa2	ENSMUSG00000028013	chr3	132973074	133041199	+	2535	-0.478	0
Brd2	ENSMUSG00000024335	chr17	34248965	34259262	-	7112	-0.48	0
Abhd10	ENSMUSG00000033157	chr16	45729838	45743068	-	3710	-0.481	0
Xrcc6	ENSMUSG00000022471	chr15	81818265	81870515	+	4476	-0.482	0
Vps4a	ENSMUSG00000031913	chr8	109555122	109569655	+	2522	-0.482	0
Uba1	ENSMUSG00000001924	chrX	20235452	20260305	+	4819	-0.483	0
Ilf2	ENSMUSG00000001016	chr3	90280123	90292301	+	1973	-0.483	0
Ighv1-42	ENSMUSG00000076707	chr12	116175324	116175617	-	294	-0.484	0
Elac1	ENSMUSG00000036941	chr18	73894692	73914133	-	4971	-0.485	0
Slc31a2	ENSMUSG00000066152	chr4	61923596	61959445	+	2033	-0.486	0
Atg9a	ENSMUSG00000033124	chr1	75177440	75188497	-	4131	-0.486	0
Capzb	ENSMUSG00000028745	chr4	138748814	138847733	+	3299	-0.486	0
Mboat7	ENSMUSG00000035596	chr7	3629392	3645125	-	7716	-0.487	0
Igkv1-117	ENSMUSG00000076516	chr6	68071085	68071821	+	359	-0.487	0
1500015O10Rik	ENSMUSG00000026051	chr1	43787447	43799423	+	902	-0.487	0
Ighj4	ENSMUSG00000076618	chr12	114666725	114666778	-	54	-0.487	0
Tmem17	ENSMUSG00000049904	chr11	22412091	22419237	+	1568	-0.488	0
Gm15265	ENSMUSG00000086288	chr3	94802722	94808791	-	974	-0.488	0
Map2k7	ENSMUSG00000002948	chr8	4238740	4251423	+	5359	-0.489	0
Usp35	ENSMUSG00000035713	chr7	104457890	104480530	-	4902	-0.489	0
Trim14	ENSMUSG00000039853	chr4	46506653	46549013	-	4442	-0.489	0
Arl10	ENSMUSG00000025870	chr13	54676376	54682489	+	1729	-0.489	0
Ror2	ENSMUSG00000021464	chr13	53204681	53381493	-	4242	-0.489	0
Gar1	ENSMUSG00000028010	chr3	129527830	129534314	-	1714	-0.489	0
Txndc15	ENSMUSG00000021497	chr13	55816011	55827582	+	1521	-0.49	0
Zfp710	ENSMUSG00000048897	chr7	87169700	87237637	+	4803	-0.491	0
Micall2	ENSMUSG00000036718	chr5	140182647	140212290	-	4147	-0.491	0
Ankrd40	ENSMUSG00000020864	chr11	94189315	94203155	+	4255	-0.493	0

Slc25a36	ENSMUSG00000032449	chr9	96976016	97011576	-	6796	-0.497	0
Wbp7	ENSMUSG00000006307	chr7	31353877	31373745	-	9596	-0.497	0
Glod4	ENSMUSG00000017286	chr11	75934489	76057227	-	6005	-0.497	0
Evi5l	ENSMUSG00000011832	chr8	4166567	4211257	+	8524	-0.498	0
Inpp5a	ENSMUSG00000025477	chr7	146575008	146765551	+	3529	-0.499	0
Tcirg1	ENSMUSG00000001750	chr19	3896050	3907133	-	4470	-0.499	0
Rpl17	ENSMUSG00000062328	chr18	75160131	75163035	+	775	-0.5	0
Ltbp3	ENSMUSG00000024940	chr19	5740904	5758532	+	5199	-0.501	0
Col6a5	ENSMUSG00000091345	chr9	105758400	105862974	-	9294	-0.501	0
Ndnl2	ENSMUSG00000070520	chr7	72011938	72017883	-	5946	-0.501	0
Mfge8	ENSMUSG00000030605	chr7	86278655	86293946	-	2125	-0.503	0
Ahsa1	ENSMUSG00000021037	chr12	88607696	88614947	+	1347	-0.503	0
Arf6	ENSMUSG00000044147	chr12	70473137	70476966	+	3730	-0.506	0
Gm15832	ENSMUSG00000085894	chr1	39604415	39608847	-	1832	-0.506	0
Mxra7	ENSMUSG00000020814	chr11	116664376	116689360	-	2391	-0.507	0
Cyba	ENSMUSG00000006519	chr8	124948672	124956840	-	758	-0.507	0
Fbxw2	ENSMUSG00000035949	chr2	34660034	34681831	-	6661	-0.507	0
Hmgn3	ENSMUSG00000066456	chr9	83003555	83040292	-	1859	-0.509	0
Gm20554	ENSMUSG00000093587	chr13	72755368	72766012	-	10405	-0.509	0
Eif4h	ENSMUSG00000040731	chr5	135095747	135115218	-	2428	-0.51	0
3110062M04Rik	ENSMUSG00000046806	chr6	34821771	34828060	-	4269	-0.511	0
Vps33a	ENSMUSG00000029434	chr5	123978773	124023024	-	4107	-0.512	0
Vac14	ENSMUSG00000010936	chr8	113142503	113244296	+	3346	-0.515	0
Mgat1	ENSMUSG00000020346	chr11	49057693	49076532	+	4733	-0.515	0
Nsfl1c	ENSMUSG00000027455	chr2	151319918	151337150	+	3967	-0.519	0
Zfp341	ENSMUSG00000059842	chr2	154439033	154472557	+	3299	-0.519	0
Rufy3	ENSMUSG00000029291	chr5	88994065	89080415	+	6415	-0.52	0
Micu1	ENSMUSG00000020111	chr10	59165225	59326880	+	9234	-0.52	0
C2cd2	ENSMUSG00000045975	chr16	98076817	98144243	-	6555	-0.523	0
Arrb1	ENSMUSG00000018909	chr7	106683976	106755281	+	11175	-0.524	0
Txlna	ENSMUSG00000053841	chr4	129303327	129318309	-	5343	-0.525	0
Ciz1	ENSMUSG00000039205	chr2	32207847	32235746	+	7437	-0.526	0
Tcf19	ENSMUSG00000050410	chr17	35649679	35653769	-	1782	-0.526	0
Rfx1	ENSMUSG00000031706	chr8	86590735	86620891	+	4172	-0.528	0
Fchsd1	ENSMUSG00000038524	chr18	38117085	38129428	-	5473	-0.528	0
Dtx3	ENSMUSG00000040415	chr10	126627434	126632784	-	2572	-0.528	0
Gm9182	ENSMUSG00000091240	chr10	128052412	128053677	+	1042	-0.528	0
Acox3	ENSMUSG00000029098	chr5	35925689	35958001	+	10343	-0.529	0
Wwc1	ENSMUSG00000018849	chr11	35651902	35794029	-	4960	-0.529	0
Gigyf1	ENSMUSG00000029714	chr5	137960108	137969162	+	5437	-0.53	0
Coro2b	ENSMUSG00000041729	chr9	62267299	62384851	-	5919	-0.532	0
Dhx32	ENSMUSG00000030986	chr7	140912625	140974409	-	4173	-0.532	0

Dok4	ENSMUSG00000040631	chr8	97387728	97400212	-	2604	-0.533	0
Eif3l	ENSMUSG00000033047	chr15	78905653	78924830	+	1893	-0.534	0
Fastkd1	ENSMUSG00000027086	chr2	69524872	69551573	-	5853	-0.535	0
Cdc42ep5	ENSMUSG00000063838	chr7	4102862	4116462	-	1288	-0.536	0
Slc25a25	ENSMUSG00000026819	chr2	32270007	32306965	-	4632	-0.537	0
Eif4e2	ENSMUSG00000026254	chr1	89110489	89137063	+	7497	-0.537	0
P4ha2	ENSMUSG00000018906	chr11	53913597	53945167	+	6239	-0.537	0
Ppip5k1	ENSMUSG00000033526	chr2	121136297	121181132	-	12486	-0.539	0
Sema3b	ENSMUSG00000057969	chr9	107500005	107511560	-	4250	-0.54	0
Kcmf1	ENSMUSG00000055239	chr6	72791108	72849973	-	3251	-0.54	0
Ppp1r15a	ENSMUSG00000040435	chr7	52778286	52781638	-	2937	-0.541	0
Agpat3	ENSMUSG00000001211	chr10	77731923	77815234	-	7577	-0.541	0
Rasip1	ENSMUSG00000044562	chr7	52882858	52894463	+	3202	-0.541	0
Znrd1as	ENSMUSG00000036214	chr17	37095537	37102567	+	1360	-0.542	0
Nfatc2	ENSMUSG00000027544	chr2	168301910	168427157	-	10578	-0.542	0
Rexo4	ENSMUSG00000052406	chr2	26809083	26819906	-	3662	-0.542	0
Sap18	ENSMUSG00000021963	chr14	58417017	58423817	+	3781	-0.543	0
Dap	ENSMUSG00000039168	chr15	31154140	31204093	+	1481	-0.544	0
Dnase1l1	ENSMUSG00000019088	chrX	71518556	71527676	-	2121	-0.544	0
Arglu1	ENSMUSG00000040459	chr8	8666577	8690537	-	1816	-0.545	0
Sfxn3	ENSMUSG00000025212	chr19	45122091	45130873	+	2941	-0.545	0
Bloc1s2	ENSMUSG00000057506	chr19	44213756	44220936	-	878	-0.547	0
Metrnl	ENSMUSG00000039208	chr11	121562858	121577620	+	1586	-0.547	0
Map3k4	ENSMUSG00000014426	chr17	12420489	12511614	-	5391	-0.548	0
Wisp2	ENSMUSG00000027656	chr2	163646597	163658882	+	1924	-0.548	0
Lmcd1	ENSMUSG00000057604	chr6	112223752	112280419	+	1730	-0.549	0
Ppm1g	ENSMUSG00000029147	chr5	31505041	31522918	-	2165	-0.55	0
Pcgf1	ENSMUSG00000069678	chr6	83027863	83030849	+	1471	-0.55	0
Fam192a	ENSMUSG00000031774	chr8	97098843	97125626	-	1797	-0.552	0
Gpr107	ENSMUSG00000000194	chr2	31007836	31075068	+	10235	-0.552	0
Exog	ENSMUSG00000042787	chr9	119354041	119374635	+	3887	-0.552	0
Tnfrsf19	ENSMUSG00000060548	chr14	61582671	61665692	-	4958	-0.552	0
Tnfaip1	ENSMUSG00000017615	chr11	78336352	78349834	-	3781	-0.552	0
Syngap1	ENSMUSG00000067629	chr17	27094363	27107590	+	2484	-0.554	0
Mkrn2	ENSMUSG00000000439	chr6	115551949	115568688	+	2247	-0.554	0
Nckipsd	ENSMUSG00000032598	chr9	108710730	108720783	+	2937	-0.555	0
3200002M19Rik	ENSMUSG00000030649	chr7	109029839	109050359	+	4737	-0.556	0

Cbx2	ENSMUSG00000025577	chr11	118884276	118892584	+	4257	-0.557	0
Mta3	ENSMUSG00000055817	chr17	84105503	84220856	+	5319	-0.558	0
Tox4	ENSMUSG00000016831	chr14	52898821	52916066	+	6940	-0.56	0
Tspan32	ENSMUSG00000000244	chr7	150190951	150205549	+	2900	-0.56	0
Pou6f1	ENSMUSG00000009739	chr15	100405749	100430415	-	6960	-0.561	0
Rps12	ENSMUSG00000061983	chr10	23504990	23507015	-	517	-0.562	0
Ddx23	ENSMUSG00000003360	chr15	98475565	98493320	-	5452	-0.562	0
Klhdc10	ENSMUSG00000029775	chr6	30351868	30405179	+	8443	-0.562	0
Usp54	ENSMUSG00000034235	chr14	21368134	21437576	-	8921	-0.563	0
Vtn	ENSMUSG00000017344	chr11	78312593	78315826	+	2030	-0.564	0
Phf20	ENSMUSG00000038116	chr2	156022202	156135688	+	10046	-0.564	0
Pcolce	ENSMUSG00000029718	chr5	138046331	138055012	-	3823	-0.565	0
Arhgef19	ENSMUSG00000028919	chr4	140795490	140813479	+	3992	-0.565	0
BC023829	ENSMUSG00000073139	chrX	67712928	67730355	-	2513	-0.566	0
Lrrc47	ENSMUSG00000029028	chr4	153385840	153395622	+	3510	-0.567	0
Hdhd2	ENSMUSG00000025421	chr18	77168865	77211641	+	4822	-0.567	0
Pitpnm1	ENSMUSG00000024851	chr19	4099998	4113965	+	6889	-0.567	0
Calcoco1	ENSMUSG00000023055	chr15	102537210	102552609	-	3963	-0.568	0
Tspan4	ENSMUSG00000025511	chr7	148661139	148679326	+	2178	-0.568	0
Hist3h2a	ENSMUSG00000078851	chr11	58768187	58770176	+	1990	-0.569	0
Hars2	ENSMUSG00000019143	chr18	36942662	36952216	+	4279	-0.571	0
Evc	ENSMUSG00000029122	chr5	37680337	37728133	-	7701	-0.571	0
Decr1	ENSMUSG00000028223	chr4	15844387	15872654	-	3879	-0.572	0
Pdgfc	ENSMUSG00000028019	chr3	80840338	81017962	+	5089	-0.574	0
Nfu1	ENSMUSG00000029993	chr6	86959230	86978455	+	2078	-0.574	0
Abhd6	ENSMUSG00000025277	chr14	8835416	8889069	+	2173	-0.575	0
Gcsh	ENSMUSG00000034424	chr8	119505710	119517437	-	1579	-0.576	0
Nol12	ENSMUSG00000033099	chr15	78765363	78774064	+	4317	-0.576	0
Raly	ENSMUSG00000027593	chr2	154616832	154692997	+	3292	-0.577	0
Sec11c	ENSMUSG00000024516	chr18	65960203	65977319	+	3084	-0.578	0
Elk1	ENSMUSG00000009406	chrX	20510521	20527734	-	5381	-0.578	0
Mrpl45	ENSMUSG00000018882	chr11	97177030	97191234	+	2596	-0.578	0
Foxq1	ENSMUSG00000038415	chr13	31650039	31652839	+	2801	-0.578	0
Aig1	ENSMUSG00000019806	chr10	13366860	13588786	-	9196	-0.579	0
1110038B12Rik	ENSMUSG00000092203	chr17	35087183	35089416	-	1811	-0.58	0
Gm16536	ENSMUSG00000089810	chr9	45664852	45666440	-	1589	-0.58	0
B020018G12Rik	ENSMUSG00000078184	chr1	177907908	177909245	-	1338	-0.583	0

Ufm1	ENSMUSG00000027746	chr3	53659134	53667752	-	4122	-0.585	0
Ephb4	ENSMUSG00000029710	chr5	137791337	137819897	+	4640	-0.586	0
Prkar1b	ENSMUSG00000025855	chr5	139493260	139625955	-	3810	-0.587	0
Agpat2	ENSMUSG00000026922	chr2	26448577	26459937	-	2130	-0.587	0
Fam82a2	ENSMUSG00000070730	chr2	118962737	118982770	-	3537	-0.588	0
2310021P13Rik	ENSMUSG00000021819	chr14	21526774	21542840	+	6072	-0.588	0
Rps6ka4	ENSMUSG00000024952	chr19	6903575	6915115	-	3143	-0.59	0
Ubn1	ENSMUSG00000039473	chr16	5050161	5086378	+	6455	-0.592	0
Nsun4	ENSMUSG00000028706	chr4	115704379	115726481	-	4322	-0.593	0
Fbxl18	ENSMUSG00000066640	chr5	143628625	143657100	-	12563	-0.594	0
Zfp41	ENSMUSG00000047003	chr15	75447109	75455730	+	4068	-0.594	0
Sec24c	ENSMUSG00000039367	chr14	21493543	21514072	+	4515	-0.595	0
Gfm2	ENSMUSG00000021666	chr13	97907892	97951150	+	10685	-0.596	0
Aptx	ENSMUSG00000028411	chr4	40629415	40650227	-	5886	-0.598	0
Ctdspl	ENSMUSG00000047409	chr9	118835571	118953116	+	5190	-0.598	0
Tpcn2	ENSMUSG00000048677	chr7	152439828	152469916	-	3011	-0.599	0
Scx	ENSMUSG00000034161	chr15	76287868	76289898	+	1136	-0.6	0
Setmar	ENSMUSG00000034639	chr6	108015039	108027116	+	1765	-0.6	0
9330151L19Rik	ENSMUSG00000090687	chr12	70298198	70300855	+	2546	-0.601	0
Tmcc2	ENSMUSG00000042066	chr1	134252892	134287858	-	5692	-0.601	0
Tex2	ENSMUSG00000040548	chr11	106363461	106474737	-	5576	-0.602	0
Itgb5	ENSMUSG00000022817	chr16	33829751	33949239	+	3362	-0.603	0
Ehd1	ENSMUSG00000024772	chr19	6273971	6300096	+	3726	-0.603	0
Crat	ENSMUSG00000026853	chr2	30255991	30271333	-	5388	-0.603	0
Zc3h10	ENSMUSG00000039810	chr10	127979019	127984830	-	4384	-0.604	0
H2-Q6	ENSMUSG00000073409	chr17	35561795	35567000	+	2166	-0.604	0
Gatad2a	ENSMUSG00000036180	chr8	72430968	72520278	-	5502	-0.605	0
Nfatc2ip	ENSMUSG00000030722	chr7	133526368	133540251	-	3388	-0.606	0
Tacstd2	ENSMUSG00000051397	chr6	67484053	67485816	-	1764	-0.607	0
Vipar	ENSMUSG00000021038	chr12	88579825	88607236	-	2701	-0.608	0
Trpc4ap	ENSMUSG00000038324	chr2	155460007	155518120	-	8753	-0.608	0
Nrbp1	ENSMUSG00000029148	chr5	31543237	31553939	+	3559	-0.609	0
Sepn1	ENSMUSG00000050989	chr4	134093807	134108081	-	3619	-0.61	0
Haus8	ENSMUSG00000035439	chr8	73772460	73796833	-	5278	-0.61	0
Cdca7	ENSMUSG00000055612	chr2	72314216	72324950	+	3202	-0.611	0
6030419C18Rik	ENSMUSG00000066607	chr9	58338044	58347494	+	879	-0.612	0
Gm4737	ENSMUSG00000048087	chr16	46153110	46155190	-	2081	-0.612	0
Mtap1s	ENSMUSG00000019261	chr8	73429873	73441425	+	3271	-0.613	0

Igsf8	ENSMUSG00000038034	chr1	174191772	174249972	+	5217	-0.614	0
Napsa	ENSMUSG00000002204	chr7	51827802	51842232	+	2158	-0.614	0
Naa35	ENSMUSG00000021555	chr13	59686620	59736159	+	5780	-0.615	0
Camta2	ENSMUSG00000040712	chr11	70482965	70501607	-	5207	-0.615	0
Nploc4	ENSMUSG00000039703	chr11	120241684	120299022	-	4776	-0.616	0
Dlgap4	ENSMUSG00000061689	chr2	156439441	156590099	+	8451	-0.616	0
Ak1	ENSMUSG00000026817	chr2	32477278	32502325	+	2793	-0.617	0
Nelf	ENSMUSG00000006476	chr2	24909875	24918401	+	4896	-0.617	0
Farsb	ENSMUSG00000026245	chr1	78414533	78485434	-	2444	-0.618	0
Adprh	ENSMUSG00000002844	chr16	38445479	38452769	-	1583	-0.619	0
Fam189b	ENSMUSG00000032657	chr3	88987065	88993217	+	3649	-0.62	0
Rnf10	ENSMUSG00000041740	chr5	115691421	115722907	-	4447	-0.622	0
Dcaf12	ENSMUSG00000028436	chr4	41238333	41261922	-	3442	-0.622	0
C87436	ENSMUSG00000046679	chr6	86388368	86423494	+	4092	-0.622	0
Cdca7l	ENSMUSG00000021175	chr12	119042762	119117179	+	3258	-0.623	0
Aldh1a1	ENSMUSG00000053279	chr19	20676451	20717952	+	2053	-0.623	0
Efna4	ENSMUSG00000028040	chr3	89137312	89141950	-	1599	-0.624	0
Nhp2l1	ENSMUSG00000063480	chr15	81871749	81878028	-	2130	-0.626	0
Snhg3	ENSMUSG00000085241	chr4	131903929	131909601	-	986	-0.626	0
Fnta	ENSMUSG00000015994	chr8	27109201	27126073	-	1874	-0.627	0
Pelp1	ENSMUSG00000018921	chr11	70206385	70223533	-	3561	-0.627	0
Gadd45b	ENSMUSG00000015312	chr10	80392836	80394949	+	1266	-0.628	0
Fcna	ENSMUSG00000026938	chr2	25480186	25483550	-	1389	-0.628	0
Rufy1	ENSMUSG00000020375	chr11	50202788	50244627	-	2764	-0.628	0
Samd12	ENSMUSG00000058656	chr15	53293176	53734092	-	1472	-0.628	0
Hdac6	ENSMUSG00000031161	chrX	7507246	7525015	-	5817	-0.629	0
Tnip2	ENSMUSG00000059866	chr5	34838736	34856646	-	2879	-0.63	0
Pde2a	ENSMUSG00000030653	chr7	108570212	108661340	+	4647	-0.63	0
Tuba1a	ENSMUSG00000072235	chr15	98780272	98783982	-	1647	-0.631	0
Pgcp	ENSMUSG00000039007	chr15	33012884	33524307	+	1923	-0.631	0
Snapc4	ENSMUSG00000036281	chr2	26218285	26236173	-	6622	-0.631	0
Fam53c	ENSMUSG00000034300	chr18	34918560	34933414	+	4517	-0.631	0
Adora2a	ENSMUSG00000020178	chr10	74779688	74797533	+	2534	-0.633	0
Ikbke	ENSMUSG00000042349	chr1	133150920	133176183	-	9018	-0.633	0
G6pdx	ENSMUSG00000031400	chrX	71654822	71674533	-	3080	-0.634	0
Pde6d	ENSMUSG00000026239	chr1	88439569	88479204	-	2178	-0.634	0
Eif1b	ENSMUSG00000006941	chr9	120401724	120404445	+	917	-0.635	0
Gm12319	ENSMUSG00000087523	chr11	70468243	70469995	-	357	-0.638	0
Aldh3a2	ENSMUSG00000010025	chr11	61036919	61080966	-	5619	-0.638	0

6330403L08Rik	ENSMUSG00000075585	chr5	139471010	139476530	+	5521	-0.639	0
Clec14a	ENSMUSG00000045930	chr12	59365707	59370245	-	4539	-0.64	0
Mrpl21	ENSMUSG00000024829	chr19	3282901	3292837	+	2011	-0.641	0
Traf5	ENSMUSG00000026637	chr1	193821311	193916440	-	1982	-0.641	0
Cdc20	ENSMUSG00000006398	chr4	118105506	118109957	-	1868	-0.641	0
Mpp7	ENSMUSG00000057440	chr18	7347960	7626861	-	5049	-0.642	0
Ypel2	ENSMUSG00000018427	chr11	86749927	86807209	-	4847	-0.643	0
Vps11	ENSMUSG00000032127	chr9	44156189	44169753	-	3179	-0.643	0
Hba-a1	ENSMUSG00000069919	chr11	32183511	32184465	+	812	-0.644	0
Ganab	ENSMUSG00000071650	chr19	8972580	8991153	+	3862	-0.647	0
Gipc3	ENSMUSG00000034872	chr10	80800507	80806011	-	1024	-0.648	0
Poc1a	ENSMUSG00000023345	chr9	106183392	106252852	+	2578	-0.648	0
Rps7	ENSMUSG00000061477	chr12	29315712	29320818	-	961	-0.648	0
Ift52	ENSMUSG00000017858	chr2	162843090	162871877	+	2802	-0.648	0
Prrx2	ENSMUSG00000039476	chr2	30690492	30736761	+	1951	-0.648	0
Smu1	ENSMUSG00000028409	chr4	40683575	40704956	-	3119	-0.651	0
Pigh	ENSMUSG00000021120	chr12	80181660	80190657	-	3468	-0.652	0
Car4	ENSMUSG00000000805	chr11	84771288	84779546	+	1966	-0.654	0
Ccdc23	ENSMUSG00000028643	chr4	118867911	118873903	+	1065	-0.654	0
Uba5	ENSMUSG00000032557	chr9	103948929	103965464	-	2541	-0.654	0
Dbt	ENSMUSG00000000340	chr3	116215988	116252894	+	3296	-0.655	0
Csrp2	ENSMUSG00000020186	chr10	110357232	110376678	+	997	-0.655	0
Card10	ENSMUSG00000033170	chr15	78605568	78633472	-	4952	-0.656	0
Timm44	ENSMUSG00000002949	chr8	4259731	4275913	-	3664	-0.657	0
Man1c1	ENSMUSG00000037306	chr4	134117605	134260205	-	5740	-0.657	0
Top3a	ENSMUSG00000002814	chr11	60553560	60590867	-	4599	-0.658	0
Ptprf	ENSMUSG00000033295	chr4	117880818	117964010	-	9130	-0.658	0
Fam82b	ENSMUSG00000028229	chr4	19502309	19534079	+	1963	-0.658	0
Atp6v0a1	ENSMUSG00000019302	chr11	100870766	100925033	+	6538	-0.661	0
Nudt10	ENSMUSG00000073293	chrX	5745814	5750133	-	1775	-0.661	0
Optn	ENSMUSG00000026672	chr2	4941688	4985097	-	3753	-0.661	0
Irak1	ENSMUSG00000031392	chrX	71259253	71269257	-	4716	-0.662	0
Actr8	ENSMUSG00000015971	chr14	30791523	30806407	+	2121	-0.662	0
Pofut2	ENSMUSG00000020260	chr10	76722045	76732331	+	2219	-0.663	0
Gm9781	ENSMUSG00000043424	chr18	43635074	43637450	-	2377	-0.663	0
Pabpc4	ENSMUSG00000011257	chr4	122939594	122976168	+	4031	-0.663	0
Cpox	ENSMUSG00000022742	chr16	58670321	58680504	+	3186	-0.663	0
Kdelr3	ENSMUSG00000010830	chr15	79346838	79358169	+	1414	-0.663	0
Trp53inp2	ENSMUSG00000038375	chr2	155206795	155215586	+	5257	-0.663	0
Ankrd9	ENSMUSG00000037904	chr12	112213563	112217250	-	3160	-0.664	0
Ppm1m	ENSMUSG00000020253	chr9	106096503	106102077	-	3485	-0.664	0

Nup62	ENSMUSG00000043858	chr7	52071458	52086179	+	2852	-0.667	0
Polm	ENSMUSG00000020474	chr11	5727863	5738019	-	3658	-0.668	0
Fam65c	ENSMUSG00000074577	chr2	167805664	167836118	-	4244	-0.67	0
Itih5	ENSMUSG00000025780	chr2	10075198	10178156	+	7900	-0.67	0
Gkap1	ENSMUSG00000021552	chr13	58334712	58375549	-	1518	-0.67	0
Gga3	ENSMUSG00000020740	chr11	115445569	115465365	-	6245	-0.671	0
Amz2	ENSMUSG00000020610	chr11	109287260	109314127	+	5584	-0.673	0
Ddx28	ENSMUSG00000045538	chr8	108533516	108535386	-	1871	-0.673	0
Lias	ENSMUSG00000029199	chr5	65782736	65801932	+	4570	-0.675	0
Hdgfrp2	ENSMUSG00000002833	chr17	56219057	56240019	+	2301	-0.675	0
Sesn3	ENSMUSG00000032009	chr9	14080745	14130582	+	2162	-0.677	0
Pigf	ENSMUSG00000024145	chr17	87396596	87424746	-	988	-0.677	0
Casp9	ENSMUSG00000028914	chr4	141349527	141371891	+	5197	-0.679	0
Fam113b	ENSMUSG00000044250	chr15	97077538	97216120	+	2198	-0.68	0
Spc24	ENSMUSG00000074476	chr9	21559886	21564733	-	1357	-0.681	0
Mrps25	ENSMUSG00000014551	chr6	92119519	92134027	-	6576	-0.681	0
Sun2	ENSMUSG00000042524	chr15	79554500	79572966	-	4969	-0.682	0
Scamp2	ENSMUSG00000040188	chr9	57408751	57436604	+	2341	-0.682	0
Grap	ENSMUSG00000004837	chr11	61466767	61486286	+	1793	-0.682	0
Stradb	ENSMUSG00000026027	chr1	59030366	59052559	+	7256	-0.682	0
Foxp4	ENSMUSG00000023991	chr17	48004082	48061594	-	4926	-0.682	0
Gmeb2	ENSMUSG00000038705	chr2	180986154	181022740	-	4988	-0.682	0
Khdrbs3	ENSMUSG00000022332	chr15	68758850	68923942	+	1887	-0.682	0
Hsd3b7	ENSMUSG00000042289	chr7	134929122	134947316	+	6154	-0.683	0
Fam120c	ENSMUSG00000025262	chrX	147778718	147906385	+	5823	-0.683	0
Srd5a3	ENSMUSG00000029233	chr5	76569296	76584529	+	4648	-0.683	0
Rpl10	ENSMUSG00000008682	chrX	71516151	71518474	+	2053	-0.684	0
Lcat	ENSMUSG00000035237	chr8	108463451	108467282	-	1342	-0.685	0
Zmynd19	ENSMUSG00000026974	chr2	24805312	24817595	+	4168	-0.685	0
Cyp2d22	ENSMUSG00000061740	chr15	82200958	82210690	-	2771	-0.685	0
Naa40	ENSMUSG00000024764	chr19	7300158	7315712	-	3275	-0.686	0
Sp8	ENSMUSG00000048562	chr12	120084802	120091051	+	4631	-0.687	0
2610015P09Rik	ENSMUSG00000022701	chr16	43889913	43964427	+	4230	-0.687	0
Esr1	ENSMUSG00000019768	chr10	5340927	5734948	-	13678	-0.688	0
1700012B15Rik	ENSMUSG00000079179	chr12	3236611	3249999	+	778	-0.69	0
Dopey2	ENSMUSG00000022946	chr16	93712152	93810830	+	7313	-0.69	0
Gpc1	ENSMUSG00000034220	chr1	94728222	94756775	+	3595	-0.69	0

A830007P12Rik	ENSMUSG00000059555	chr2	25048486	25052406	-	3301	-0.69	0
Ttf1	ENSMUSG00000026803	chr2	28915782	28943176	+	6023	-0.69	0
Usp19	ENSMUSG00000006676	chr9	108393020	108404670	+	5202	-0.691	0
Gm14005	ENSMUSG00000074813	chr2	128021729	128255085	-	2045	-0.691	0
Upf1	ENSMUSG00000058301	chr8	72855421	72877172	-	4616	-0.692	0
Hspa12b	ENSMUSG00000074793	chr2	130953016	130972057	+	3685	-0.692	0
Pitpnm2	ENSMUSG00000029406	chr5	124568699	124699769	-	12843	-0.692	0
Zrsr1	ENSMUSG00000044068	chr11	22873215	22876498	+	3284	-0.693	0
Mesdc2	ENSMUSG00000038503	chr7	91032976	91050042	+	5081	-0.694	0
Atp1b2	ENSMUSG00000041329	chr11	69413238	69419444	-	4085	-0.695	0
Fto	ENSMUSG00000055932	chr8	93837431	94192338	+	13026	-0.696	0
Dedd	ENSMUSG00000013973	chr1	173259276	173272462	+	5861	-0.697	0
Cdk2ap1	ENSMUSG00000029394	chr5	124795426	124804680	-	1945	-0.698	0
Sertad1	ENSMUSG00000008384	chr7	28271929	28275335	+	1344	-0.699	0
Oxnad1	ENSMUSG00000021906	chr14	32898560	32916388	+	5769	-0.7	0
Ddx11	ENSMUSG00000035842	chr17	66472860	66501507	+	4157	-0.701	0
Sec23b	ENSMUSG00000027429	chr2	144381965	144416485	+	6302	-0.701	0
Ppp6r2	ENSMUSG00000036561	chr15	89083466	89116692	+	3088	-0.702	0
Nfyc	ENSMUSG00000032897	chr4	120430043	120504177	-	2942	-0.702	0
Rbm43	ENSMUSG00000036249	chr2	51779968	51790683	-	2633	-0.702	0
Zfp598	ENSMUSG00000041130	chr17	24806697	24818960	+	3286	-0.702	0
Cxx1a	ENSMUSG00000067925	chrX	50945665	50946940	-	1276	-0.702	0
Wdr92	ENSMUSG00000078970	chr11	17082110	17135203	+	3903	-0.702	0
Ube2a	ENSMUSG00000016308	chrX	34414353	34424210	+	1662	-0.702	0
Vps26b	ENSMUSG00000031988	chr9	26812088	26837679	-	6872	-0.703	0
Boc	ENSMUSG00000022687	chr16	44485158	44559010	-	4456	-0.703	0
Cspg4	ENSMUSG00000032911	chr9	56712911	56747677	+	8050	-0.703	0
Mppe1	ENSMUSG00000062526	chr18	67385184	67405484	-	2007	-0.705	0
Szt2	ENSMUSG00000033253	chr4	118035348	118081878	-	11948	-0.705	0
3110082I17Rik	ENSMUSG00000053553	chr5	139835693	139936456	-	1429	-0.705	0
Hlcs	ENSMUSG00000040820	chr16	94350488	94535170	-	5609	-0.707	0
Nfkb2	ENSMUSG00000025225	chr19	46379227	46386580	+	3289	-0.707	0
2810006K23Rik	ENSMUSG00000047635	chr5	124778098	124791853	+	2873	-0.707	0
Tob1	ENSMUSG00000037573	chr11	94072768	94076809	+	2288	-0.709	0
Mrpl19	ENSMUSG00000030045	chr6	81907845	81915952	-	5450	-0.709	0
Hist1h1c	ENSMUSG00000036181	chr13	23830676	23832235	+	1560	-0.709	0
Exoc8	ENSMUSG00000074030	chr8	127414200	127421605	-	7406	-0.71	0
Abcf2	ENSMUSG00000028953	chr5	24071161	24083217	-	2503	-0.71	0
Cd3eap	ENSMUSG00000047649	chr7	19942198	19944832	-	1657	-0.71	0

Kcnh2	ENSMUSG00000038319	chr5	23825407	23857422	-	4845	-0.711	0
9430008 C03Rik	ENSMUSG00000085385	chr2	158181043	158187316	-	4643	-0.712	0
0610009 B22Rik	ENSMUSG00000007777	chr11	51498888	51502376	-	998	-0.713	0
H2-Aa	ENSMUSG00000036594	chr17	34406276	34424772	-	1987	-0.713	0
Arhgef5	ENSMUSG00000033542	chr6	43215643	43239319	+	5407	-0.714	0
Cog7	ENSMUSG00000034951	chr7	129023547	129125225	-	4411	-0.716	0
Pgm2	ENSMUSG00000025791	chr4	99602019	99659899	+	2921	-0.716	0
Col4a5	ENSMUSG00000031274	chrX	137909928	138123778	+	6719	-0.716	0
Zfp395	ENSMUSG00000034522	chr14	65977513	66017767	+	4386	-0.716	0
1700123 O20Rik	ENSMUSG00000040822	chr14	55305008	55309579	+	1889	-0.717	0
Rsad1	ENSMUSG00000039096	chr11	94401112	94410569	-	4391	-0.718	0
Kctd7	ENSMUSG00000034110	chr5	130620731	130631676	+	4368	-0.721	0
BC0570 79	ENSMUSG00000038368	chr4	87740533	88056915	+	11311	-0.722	0
0610009 D07Rik	ENSMUSG00000037361	chr12	4824414	4834464	+	1115	-0.722	0
Znf512b	ENSMUSG00000000823	chr2	181316808	181327508	-	6810	-0.722	0
Atat1	ENSMUSG00000024426	chr17	36034540	36047020	-	4586	-0.722	0
Adat1	ENSMUSG00000031949	chr8	114490808	114516202	-	4399	-0.723	0
Txnl4a	ENSMUSG00000057130	chr18	80403534	80420272	+	2185	-0.723	0
Cdc123	ENSMUSG00000039128	chr2	5715340	5766210	-	3463	-0.725	0
42982	ENSMUSG00000020486	chr11	87382405	87404041	+	3563	-0.725	0
Hpcal1	ENSMUSG00000071379	chr12	17697620	17798732	+	1561	-0.725	0
Hspa1a	ENSMUSG00000091971	chr17	35106144	35109069	-	2926	-0.726	0
Tmem1 75	ENSMUSG00000013495	chr5	109058789	109076789	+	3875	-0.726	0
Pdzd11	ENSMUSG00000015668	chrX	97818222	97821907	-	2127	-0.726	0
C1qbp	ENSMUSG00000018446	chr11	70791338	70796528	-	1262	-0.726	0
Sall2	ENSMUSG00000049532	chr14	52930847	52948437	-	5433	-0.727	0
Whamm	ENSMUSG00000045795	chr7	88716178	88741722	+	3135	-0.727	0
Anxa9	ENSMUSG00000015702	chr3	95100018	95111098	-	2073	-0.727	0
Gm6543	ENSMUSG00000054284	chr5	21104697	21105368	+	672	-0.727	0
Eno2	ENSMUSG00000004267	chr6	124710071	124719691	-	4705	-0.728	0
Morn4	ENSMUSG00000049670	chr19	42149429	42160860	-	1816	-0.728	0
Gm1354 0	ENSMUSG00000025201	chr2	52475252	52476121	+	870	-0.728	0
Ppm1h	ENSMUSG00000034613	chr10	122115818	122382851	+	10226	-0.729	0
Ppil3	ENSMUSG00000026035	chr1	58487838	58502330	-	2132	-0.73	0
Traf3ip2	ENSMUSG00000019842	chr10	39332740	39375113	+	3687	-0.73	0
Wnt7b	ENSMUSG00000022382	chr15	85365869	85412251	-	4413	-0.731	0

Git1	ENSMUSG00000011877	chr11	77307064	77321288	+	4838	-0.732		0
Aven	ENSMUSG00000003604	chr2	112333121	112474730	+	5001	-0.733		0
Ifngr2	ENSMUSG00000022965	chr16	91547317	91565414	+	3313	-0.734		0
Gm17586	ENSMUSG00000090998	chr11	120495082	120496439	+	908	-0.734		0
Tom1l2	ENSMUSG00000000538	chr11	60040216	60166407	-	8764	-0.734		0
BC026585	ENSMUSG00000033488	chr1	159388711	159419191	+	1618	-0.735		0
Zbtb3	ENSMUSG00000071661	chr19	8876986	8879344	+	2071	-0.736		0
1810048J11Rik	ENSMUSG00000034501	chr12	73637344	73681200	+	4420	-0.737		0
Adam15	ENSMUSG00000028041	chr3	89142464	89153932	-	6159	-0.738		0
Serp1	ENSMUSG00000027808	chr3	58325891	58329795	-	2440	-0.738		0
Rpusd2	ENSMUSG00000027324	chr2	118860526	118865505	+	2706	-0.74		0
Gbas	ENSMUSG00000029432	chr5	130230938	130264203	+	2750	-0.741		0
Dennd1a	ENSMUSG00000035392	chr2	37654510	38142910	-	5758	-0.741		0
Gadd45g	ENSMUSG00000021453	chr13	51942047	51943837	+	1173	-0.742		0
Fam110a	ENSMUSG00000027459	chr2	151795134	151805955	-	2207	-0.742		0
Kctd13	ENSMUSG00000030685	chr7	134072393	134089145	+	2487	-0.743		0
Ampd2	ENSMUSG00000027889	chr3	107876980	107889569	-	5069	-0.744		0
Tbca	ENSMUSG00000042043	chr13	95558898	95612854	+	503	-0.746		0
Gab1	ENSMUSG00000031714	chr8	83288333	83404378	-	4859	-0.748		0
Ccdc32	ENSMUSG00000039983	chr2	118843515	118855129	-	2214	-0.748		0
Mmab	ENSMUSG00000029575	chr5	114881043	114894068	-	3530	-0.75		0
Lpxn	ENSMUSG00000024696	chr19	12873096	12908297	+	1675	-0.75		0
Cd82	ENSMUSG00000027215	chr2	93259268	93303297	-	3005	-0.751		0
Chst12	ENSMUSG00000036599	chr5	140981504	141001678	+	2362	-0.753		0
9130221H12Rik	ENSMUSG00000090330	chr7	26013706	26015276	+	1465	-0.754		0
Plcd1	ENSMUSG00000010660	chr9	118980645	119002614	-	2651	-0.754		0
Tssc1	ENSMUSG00000036613	chr12	29436693	29552356	+	1692	-0.754		0
Lphn1	ENSMUSG00000013033	chr8	86424004	86465853	+	8587	-0.755		0
Rnf157	ENSMUSG00000052949	chr11	116197667	116274346	-	9537	-0.755		0
Eif4ebp1	ENSMUSG00000031490	chr8	28370799	28386128	+	981	-0.757		0
Gorasp2	ENSMUSG00000014959	chr2	70499633	70550693	+	4145	-0.757		0
Rad1	ENSMUSG00000022248	chr15	10415773	10428818	+	7400	-0.757		0
Rps25	ENSMUSG00000009927	chr9	44215797	44218488	+	683	-0.757		0
Josd1	ENSMUSG00000022426	chr15	79504680	79518302	-	3394	-0.758		0
Cdh1	ENSMUSG00000000303	chr8	109127251	109194146	+	4574	-0.759		0
Hdac1	ENSMUSG00000028800	chr4	129193348	129219957	-	3445	-0.759		0
Rab11fip3	ENSMUSG00000037098	chr17	26125981	26206354	-	6798	-0.759		0

Slc33a1	ENSMUSG00000027822	chr3	63737429	63768690	-	3548	-0.76	0
Kat2a	ENSMUSG00000020918	chr11	100566060	100573779	-	3570	-0.76	0
Cox6c	ENSMUSG00000014313	chr15	35855641	35868001	-	3567	-0.761	0
Rab5c	ENSMUSG00000019173	chr11	100576323	100599529	-	2194	-0.762	0
Rexo2	ENSMUSG00000032026	chr9	48276619	48288716	-	1075	-0.764	0
Ssbp3	ENSMUSG00000061887	chr4	106583306	106722299	+	6688	-0.764	0
Nudt21	ENSMUSG00000031754	chr8	96543303	96560939	-	1111	-0.765	0
Cdk20	ENSMUSG00000021483	chr13	64533850	64541028	+	1837	-0.766	0
Prkd2	ENSMUSG00000041187	chr7	17428283	17455810	+	3804	-0.767	0
Ralbp1	ENSMUSG00000024096	chr17	66197785	66234634	-	3771	-0.769	0
Lad1	ENSMUSG00000041782	chr1	137715175	137729918	+	2992	-0.77	0
Cdkn2aipnl	ENSMUSG00000020392	chr11	51781163	51790836	+	1958	-0.771	0
Nsa2	ENSMUSG00000060739	chr13	97899382	97907881	-	2686	-0.771	0
Tspyl2	ENSMUSG00000041096	chrX	148771395	148776968	-	2807	-0.772	0
Jub	ENSMUSG00000022178	chr14	55186309	55196253	-	3255	-0.773	0
Zmym3	ENSMUSG00000031310	chrX	98599723	98616188	-	8021	-0.774	0
Tmem50a	ENSMUSG00000028822	chr4	134453764	134470939	-	1270	-0.775	0
Eif3b	ENSMUSG00000056076	chr5	140895259	140919314	+	2968	-0.775	0
Cct3	ENSMUSG00000001416	chr3	88101038	88125689	+	4689	-0.777	0
Sdpr	ENSMUSG00000045954	chr1	51345970	51359803	+	3054	-0.777	0
Srr	ENSMUSG00000001323	chr11	74719861	74739450	-	4560	-0.778	0
Npc2	ENSMUSG00000021242	chr12	86095510	86114062	-	3261	-0.78	0
Mvp	ENSMUSG00000030681	chr7	134130374	134158135	-	2902	-0.78	0
Ccdc12	ENSMUSG00000019659	chr9	110559007	110614094	+	818	-0.782	0
Ubfd1	ENSMUSG00000030870	chr7	129210689	129225708	+	9629	-0.782	0
Glt25d1	ENSMUSG00000034807	chr8	74134923	74148808	+	3179	-0.783	0
Mid1	ENSMUSG00000035299	chrX	166123129	166443668	+	6722	-0.783	0
Wdr5	ENSMUSG00000026917	chr2	27370677	27392055	+	3596	-0.786	0
Calm3	ENSMUSG00000019370	chr7	17500728	17509463	-	2423	-0.787	0
Rangap1	ENSMUSG00000022391	chr15	81534678	81560349	-	3211	-0.787	0
Mlh1	ENSMUSG00000032498	chr9	111130732	111174112	-	4818	-0.788	0
Vasp	ENSMUSG00000030403	chr7	19842278	19857166	-	2438	-0.788	0
1700030K09Rik	ENSMUSG00000052794	chr8	74967779	74984900	+	2970	-0.791	0
Hbb-b1	ENSMUSG00000052305	chr7	110975048	110976610	-	863	-0.791	0
Coro7	ENSMUSG00000039637	chr16	4626133	4679777	-	7420	-0.792	0
Pnp	ENSMUSG00000021871	chr14	51563977	51573087	+	2942	-0.792	0
Dpf2	ENSMUSG00000024826	chr19	5896516	5913010	-	6501	-0.792	0
Wwox	ENSMUSG00000004637	chr8	116963555	117876608	+	10173	-0.794	0
Cinp	ENSMUSG00000021276	chr12	112110832	112127325	-	2185	-0.794	0
Dnajc3	ENSMUSG00000022136	chr14	119337154	119380924	+	5190	-0.795	0

9930012K11Rik	ENSMUSG00000044551	chr14	70554212	70559309	-	2588	-0.795	0
2210013O21Rik	ENSMUSG00000086316	chrX	150158133	150175807	+	1224	-0.797	0
Lphn2	ENSMUSG00000028184	chr3	148478550	148652280	-	8429	-0.797	0
Tuft1	ENSMUSG00000005968	chr3	94416679	94462794	-	3117	-0.798	0
Actr10	ENSMUSG00000021076	chr12	72038844	72065705	+	4022	-0.799	0
Clip3	ENSMUSG00000013921	chr7	31076691	31093386	+	3991	-0.799	0
Cul7	ENSMUSG00000038545	chr17	46787286	46801313	+	7604	-0.8	0
Brd9	ENSMUSG00000057649	chr13	74075286	74098342	+	2406	-0.801	0
4931406C07Rik	ENSMUSG00000031938	chr9	15087783	15106000	-	2562	-0.801	0
Ctdsp2	ENSMUSG00000078429	chr10	126415773	126437031	+	5065	-0.801	0
Man1a	ENSMUSG00000003746	chr10	53624594	53795602	-	9058	-0.805	0
Baat1	ENSMUSG00000000148	chr5	141180965	141195333	+	6179	-0.805	0
Supv3l1	ENSMUSG00000020079	chr10	61891957	61912486	-	2969	-0.806	0
Eef1a1	ENSMUSG00000037742	chr9	78326256	78336958	-	2493	-0.806	0
S100g	ENSMUSG00000040808	chrX	159399924	159402531	-	480	-0.806	0
Rnf122	ENSMUSG00000039328	chr8	32222292	32241954	+	7080	-0.807	0
2700062C07Rik	ENSMUSG00000024273	chr18	24629362	24636266	+	1040	-0.808	0
Alyref	ENSMUSG00000025134	chr11	120453435	120459679	-	3839	-0.808	0
Gm9797	ENSMUSG00000045455	chr10	11329021	11329633	+	613	-0.808	0
Bmp7	ENSMUSG00000008999	chr2	172693513	172765822	-	4019	-0.809	0
Gm10575	ENSMUSG00000073787	chr7	148647380	148653670	-	2033	-0.81	0
Cd74	ENSMUSG00000024610	chr18	60963502	60972300	+	1410	-0.811	0
Fam167b	ENSMUSG00000050493	chr4	129254059	129255793	-	959	-0.811	0
Gm12184	ENSMUSG00000078154	chr11	48638848	48640157	-	1310	-0.811	0
Pola2	ENSMUSG00000024833	chr19	5940543	5964206	-	2872	-0.811	0
Il15ra	ENSMUSG00000023206	chr2	11626914	11655613	+	4118	-0.813	0
Cct5	ENSMUSG00000022234	chr15	31520555	31531602	-	2809	-0.813	0
Ulk3	ENSMUSG00000032308	chr9	57437259	57444040	+	2826	-0.816	0
Rpl27a	ENSMUSG00000046364	chr7	116662661	116665881	+	2774	-0.817	0
Psmd7	ENSMUSG00000039067	chr8	110104281	110112382	-	1599	-0.819	0
Tada3	ENSMUSG00000048930	chr6	113316019	113327877	-	3883	-0.819	0
Aktip	ENSMUSG00000031667	chr8	93635940	93658364	-	2992	-0.82	0
Tbrg1	ENSMUSG00000011114	chr9	37456767	37464897	-	3501	-0.82	0
Inppl1	ENSMUSG00000032737	chr7	108971147	108986740	-	5095	-0.823	0
Ap1m1	ENSMUSG00000003033	chr8	74763917	74781284	+	3150	-0.824	0
Ube3b	ENSMUSG00000029577	chr5	114830637	114871177	+	5264	-0.824	0
Polr3c	ENSMUSG00000028099	chr3	96515413	96531551	-	3386	-0.824	0

Slc29a1	ENSMUSG00000023942	chr17	45722149	45736555	-	4008	-0.824	0
Gas2l1	ENSMUSG00000034201	chr11	4954135	4965330	-	6100	-0.825	0
Galntl1	ENSMUSG00000021130	chr12	81619458	81704883	+	4098	-0.825	0
Man2b1	ENSMUSG00000005142	chr8	87607168	87622638	+	4325	-0.827	0
Cryab	ENSMUSG00000032060	chr9	50560863	50564738	+	1185	-0.827	0
Serpine 2	ENSMUSG00000026249	chr1	79790772	79855271	-	3830	-0.828	0
Tmem8 6a	ENSMUSG00000010307	chr7	54306010	54310146	+	1791	-0.829	0
Dnajc17	ENSMUSG00000034278	chr2	118998236	119034531	-	1308	-0.829	0
Pkmyt1	ENSMUSG00000023908	chr17	23863303	23873696	+	2039	-0.83	0
C1ra	ENSMUSG00000055172	chr6	124462423	124473458	+	3001	-0.831	0
1110007 C09Rik	ENSMUSG00000037960	chr13	49298320	49311395	-	934	-0.831	0
1500002 O20Rik	ENSMUSG00000002210	chr7	25184638	25207797	+	3702	-0.831	0
Enoph1	ENSMUSG00000029326	chr5	100469013	100497781	+	1826	-0.834	0
Gm6843	ENSMUSG00000074780	chr10	95135625	95136085	-	426	-0.835	0
Cyp4f16	ENSMUSG00000048440	chr17	32673503	32688743	+	3017	-0.835	0
Ptgs1	ENSMUSG00000047250	chr2	36085946	36107792	+	3884	-0.837	0
Slc35e4	ENSMUSG00000048807	chr11	3807024	3814667	-	3063	-0.838	0
Zfp503	ENSMUSG00000039081	chr14	22803184	22808823	-	4213	-0.838	0
Tmem8 0	ENSMUSG00000025505	chr7	148513638	148523055	+	3313	-0.838	0
Gpr146	ENSMUSG00000044197	chr5	139853696	139872369	+	6379	-0.838	0
Cygb	ENSMUSG00000020810	chr11	116506909	116515627	-	2331	-0.838	0
Tns1	ENSMUSG00000055322	chr1	73956805	74171021	-	10832	-0.839	0
Shfm1	ENSMUSG00000042541	chr6	6508283	6528652	-	477	-0.84	0
Edc4	ENSMUSG00000036270	chr8	108404781	108418808	+	5497	-0.841	0
6030408 B16Rik	ENSMUSG00000075408	chr15	101123643	101127046	+	1155	-0.841	0
Lama2	ENSMUSG00000019899	chr10	26701094	27336748	-	9728	-0.841	0
Rfc5	ENSMUSG00000029363	chr5	117828112	117839056	-	4669	-0.841	0
Stac2	ENSMUSG00000017400	chr11	97897937	97914776	-	3139	-0.842	0
Mapkap k2	ENSMUSG00000016528	chr1	132950281	132994144	-	2591	-0.842	0
Gak	ENSMUSG00000062234	chr5	108998430	109058758	-	5938	-0.842	0
4930471 M23Rik	ENSMUSG00000029175	chr5	30950306	30962104	+	3311	-0.842	0
Hspa2	ENSMUSG00000059970	chr12	77505163	77507921	+	2520	-0.843	0
Hyal2	ENSMUSG00000010047	chr9	107471448	107475107	+	1893	-0.844	0

Marveld 1	ENSMUSG00000044345	chr19	42221890	42226185	+	3058	-0.845		0
Gm1235 2	ENSMUSG00000084835	chr11	98299351	98300360	+	760	-0.846		0
D93004 8N14Rik	ENSMUSG00000052563	chr11	51464456	51471183	+	3429	-0.847		0
Cxcl12	ENSMUSG00000061353	chr6	117118553	117131385	+	8176	-0.847		0
BC0182 42	ENSMUSG00000040563	chr9	21741477	21753344	+	2622	-0.847		0
Mafk	ENSMUSG00000018143	chr5	140267467	140278607	+	3901	-0.848		0
Srp19	ENSMUSG00000014504	chr18	34490501	34496253	+	3630	-0.848		0
Rcc2	ENSMUSG00000040945	chr4	140256456	140279135	+	4155	-0.848		0
Yeats4	ENSMUSG00000020171	chr10	116652198	116661563	-	1407	-0.85		0
Psmc2	ENSMUSG00000028932	chr5	21291101	21309605	+	5031	-0.85		0
Rexo1	ENSMUSG00000047417	chr10	80003671	80024305	-	5249	-0.85		0
2700060 E02Rik	ENSMUSG00000021807	chr14	20630624	20643045	-	1510	-0.851		0
Ezh1	ENSMUSG00000006920	chr11	101052429	101087777	-	7440	-0.852		0
Dnttip1	ENSMUSG00000017299	chr2	164571483	164594107	+	2122	-0.852		0
Cno	ENSMUSG00000060708	chr5	37138613	37139918	-	1306	-0.854		0
Camk1d	ENSMUSG00000039145	chr2	5214503	5635561	-	7427	-0.854		0
Arhgdia	ENSMUSG00000025132	chr11	120439418	120442938	-	2258	-0.855		0
Nudcd3	ENSMUSG00000053838	chr11	6005694	6100418	-	6496	-0.855		0
Rad9	ENSMUSG00000024824	chr19	4195198	4201603	-	2046	-0.856		0
Naif1	ENSMUSG00000039164	chr2	32305977	32312473	+	4650	-0.857		0
Rpl21-ps4	ENSMUSG00000072714	chr14	12060066	12060506	+	441	-0.859		0
Zfp668	ENSMUSG00000049728	chr7	135008684	135020342	-	4655	-0.859		0
Rhbdd2	ENSMUSG00000039917	chr5	136108488	136122318	+	7478	-0.86		0
Usp4	ENSMUSG00000032612	chr9	108250162	108294860	+	3668	-0.861		0
Slc9a1	ENSMUSG00000028854	chr4	132925621	132979617	+	5324	-0.862		0
Slc22a5	ENSMUSG00000018900	chr11	53678044	53705162	-	3071	-0.863		0
Podn	ENSMUSG00000028600	chr4	107687396	107704942	-	4212	-0.863		0
Arl6ip4	ENSMUSG00000029404	chr5	124566098	124568205	+	1438	-0.863		0
Aldoart2	ENSMUSG00000063129	chr12	56666226	56667883	+	1658	-0.863		0
Fam118 b	ENSMUSG00000050471	chr9	35024550	35075390	-	5981	-0.864		0
Spata5l1	ENSMUSG00000074876	chr2	122456361	122458666	+	1547	-0.864		0
Vps39	ENSMUSG00000027291	chr2	120142197	120178873	-	8605	-0.864		0
Usp30	ENSMUSG00000029592	chr5	114550342	114573515	+	3372	-0.865		0
Tgm2	ENSMUSG00000037820	chr2	157942138	157972172	-	4608	-0.865		0
Slc22a2 1	ENSMUSG00000063652	chr11	53763467	53793834	-	3645	-0.866		0
Slc6a2	ENSMUSG00000055368	chr8	95483978	95525566	+	6176	-0.866		0

Polr3gl	ENSMUSG00000028104	chr3	96381795	96398104	-	1597	-0.867	0
Ube2q1	ENSMUSG00000042572	chr3	89577531	89587919	+	3081	-0.869	0
Pfdn2	ENSMUSG00000006412	chr1	173275801	173289385	+	1937	-0.869	0
Dffb	ENSMUSG00000029027	chr4	153338558	153349235	-	2351	-0.87	0
H2-DMa	ENSMUSG00000037649	chr17	34272127	34276046	+	1641	-0.87	0
Snrpa	ENSMUSG00000061479	chr7	27972024	27981290	-	1760	-0.872	0
Cstb	ENSMUSG00000005054	chr10	77888415	77890364	+	599	-0.872	0
Rrp12	ENSMUSG00000035049	chr19	41937342	41970643	-	4319	-0.874	0
Prdx3	ENSMUSG00000024997	chr19	60939954	60950459	-	1478	-0.874	0
Pcbp1	ENSMUSG00000051695	chr6	86474493	86476165	-	1673	-0.874	0
H2afz	ENSMUSG00000037894	chr3	137527451	137529886	+	1776	-0.875	0
Glo1	ENSMUSG00000024026	chr17	30729811	30749604	-	1935	-0.878	0
Isy1	ENSMUSG00000030056	chr6	87768441	87788753	-	1620	-0.879	0
Vdac1	ENSMUSG00000020402	chr11	52174362	52202899	+	2620	-0.879	0
Ankzf1	ENSMUSG00000026199	chr1	75188709	75195962	+	5159	-0.88	0
Cox17	ENSMUSG00000046516	chr16	38347077	38362383	+	5972	-0.882	0
Slc25a3	ENSMUSG00000061904	chr10	90579319	90586804	-	5550	-0.883	0
Psma6	ENSMUSG00000021024	chr12	56485209	56519441	+	1234	-0.886	0
Id4	ENSMUSG00000021379	chr13	48356796	48359405	+	1660	-0.886	0
Slc25a20	ENSMUSG00000032602	chr9	108564429	108586972	+	1783	-0.887	0
Lmo7	ENSMUSG00000033060	chr14	102129145	102349230	+	11992	-0.888	0
Ighj3	ENSMUSG00000076619	chr12	114667296	114667343	-	48	-0.888	0
Wipi2	ENSMUSG00000029578	chr5	143105496	143146542	+	8041	-0.89	0
Psap	ENSMUSG00000004207	chr10	59740375	59765345	+	2729	-0.891	0
Ints9	ENSMUSG00000021975	chr14	65568882	65658663	+	4142	-0.891	0
Abcb10	ENSMUSG00000031974	chr8	126476359	126507022	-	4306	-0.892	0
Epb4.1	ENSMUSG00000028906	chr4	131479328	131631236	-	9118	-0.893	0
Bmf	ENSMUSG00000040093	chr2	118354493	118375423	-	5253	-0.893	0
Hadha	ENSMUSG00000025745	chr5	30444841	30481702	-	5341	-0.893	0
Tbkbp1	ENSMUSG00000038517	chr11	96997485	97012809	-	3975	-0.894	0
Klhl8	ENSMUSG00000029312	chr5	104290992	104340278	-	3491	-0.894	0
Slc16a2	ENSMUSG00000033965	chrX	100892753	101017304	-	4244	-0.899	0
Zfp622	ENSMUSG00000052253	chr15	25914121	25928237	+	2803	-0.899	0
Tmbim1	ENSMUSG00000006301	chr1	74334821	74352196	-	12659	-0.9	0
Rxra	ENSMUSG00000015846	chr2	27531960	27618477	+	5462	-0.902	0
Twist2	ENSMUSG00000007805	chr1	93698038	93744605	+	1329	-0.903	0
Nr4a1	ENSMUSG00000023034	chr15	101097277	101105223	+	2474	-0.904	0
Tgfb1i1	ENSMUSG00000030782	chr7	135390326	135398287	+	4659	-0.904	0
Fasn	ENSMUSG00000025153	chr11	120667254	120685492	-	9849	-0.904	0
Stat2	ENSMUSG00000040033	chr10	127707629	127729905	+	4406	-0.904	0
Ube2j2	ENSMUSG00000023286	chr4	155317940	155333713	+	4409	-0.905	0
Nr1d1	ENSMUSG00000020889	chr11	98629246	98636647	-	3124	-0.905	0
Atg16l1	ENSMUSG00000026289	chr1	89652445	89689003	+	9647	-0.906	0
Rhog	ENSMUSG00000073982	chr7	109387637	109398637	-	1356	-0.906	0

Unc119b	ENSMUSG00000046562	chr5	115572559	115584984	-	4603	-0.908	0
Ptdss1	ENSMUSG00000021518	chr13	67033766	67099337	+	3424	-0.908	0
Actr1b	ENSMUSG00000037351	chr1	36756047	36771267	-	3186	-0.91	0
Zfp346	ENSMUSG00000021481	chr13	55206672	55236186	+	4176	-0.911	0
Ccdc137	ENSMUSG00000049957	chr11	120319429	120325672	+	3803	-0.912	0
Leng1	ENSMUSG00000078813	chr7	3611700	3617442	-	2222	-0.916	0
Marveld2	ENSMUSG00000021636	chr13	101365912	101386926	-	3382	-0.917	0
Pard6g	ENSMUSG00000056214	chr18	80243634	80316379	+	3161	-0.92	0
Tspan17	ENSMUSG00000025875	chr13	54890738	54898137	+	1657	-0.92	0
Tchp	ENSMUSG00000002486	chr5	115157769	115172336	+	3851	-0.92	0
Letmd1	ENSMUSG00000037353	chr15	100299465	100309683	+	2579	-0.921	0
Dym	ENSMUSG00000035765	chr18	75178426	75446620	+	2485	-0.922	0
Coro1a	ENSMUSG00000030707	chr7	133843287	133851301	-	2783	-0.922	0
Shisa5	ENSMUSG00000025647	chr9	108941079	108960238	+	3204	-0.922	0
Zdhhc12	ENSMUSG00000015335	chr2	29946464	29949168	-	1785	-0.923	0
Ift81	ENSMUSG00000029469	chr5	123000213	123064527	-	3453	-0.923	0
Mthfd2	ENSMUSG00000005667	chr6	83255685	83267600	-	2722	-0.924	0
1700019E19Rik	ENSMUSG00000007867	chr12	87423513	87503408	+	831	-0.924	0
Fbln5	ENSMUSG00000021186	chr12	102984775	103057265	-	5829	-0.924	0
N6amt1	ENSMUSG00000044442	chr16	87354430	87368987	+	6135	-0.925	0
5830428H23Rik	ENSMUSG00000074733	chr19	61129743	61216743	-	4696	-0.927	0
Nudt7	ENSMUSG00000031767	chr8	116657457	116676212	+	3191	-0.927	0
Tax1bp3	ENSMUSG00000040158	chr11	72990585	72996664	+	2989	-0.927	0
Syce2	ENSMUSG00000003824	chr8	87396010	87412120	+	1968	-0.929	0
Stbd1	ENSMUSG00000047963	chr5	93032077	93035605	+	2071	-0.929	0
C1qtnf9	ENSMUSG00000071347	chr14	61386971	61399706	+	1903	-0.93	0
Ccdc22	ENSMUSG00000031143	chrX	7170935	7182643	-	3427	-0.93	0
Lars	ENSMUSG00000024493	chr18	42362004	42421781	-	3861	-0.932	0
Nfs1	ENSMUSG00000027618	chr2	155904665	155969922	-	4188	-0.932	0
Ogg1	ENSMUSG00000030271	chr6	113276966	113285062	+	2034	-0.933	0
Lrrc26	ENSMUSG00000026961	chr2	25145435	25146700	+	1190	-0.933	0
AW555464	ENSMUSG00000072825	chr12	113960385	113984802	+	6640	-0.933	0
Nol10	ENSMUSG00000061458	chr12	17355285	17437381	+	3480	-0.934	0
Lrrc20	ENSMUSG00000037151	chr10	60938581	61044974	+	2619	-0.934	0
Gm7353	ENSMUSG00000055452	chr7	3108535	3111950	-	3338	-0.934	0
Cdc25a	ENSMUSG00000032477	chr9	109778083	109796392	+	3626	-0.936	0
Vsig10	ENSMUSG00000066894	chr5	117769275	117805014	+	5122	-0.936	0

Sart3	ENSMUSG00000018974	chr5	114192455	114221658	-	3586	-0.937		0
Bcl9	ENSMUSG00000038256	chr3	97007585	97032769	-	8285	-0.937		0
Gja4	ENSMUSG00000050234	chr4	126988665	126991283	-	1685	-0.94		0
Chd1l	ENSMUSG00000028089	chr3	97364665	97414126	-	3008	-0.941		0
Thyn1	ENSMUSG00000035443	chr9	26807262	26814915	+	959	-0.942		0
Pkd1	ENSMUSG00000032855	chr17	24686895	24733459	+	14189	-0.942		0
Dph2	ENSMUSG00000028540	chr4	117561248	117564637	-	3030	-0.943		0
Fam102a	ENSMUSG00000039157	chr2	32390852	32425276	+	6066	-0.943		0
Cldn18	ENSMUSG00000032473	chr9	99589880	99617686	-	4456	-0.944		0
Pole3	ENSMUSG00000028394	chr4	62183683	62186102	-	2040	-0.945		0
Cyth2	ENSMUSG00000003269	chr7	53062007	53069951	-	3515	-0.947		0
Pwp2	ENSMUSG00000032834	chr10	77633655	77647894	-	3871	-0.947		0
Surf4	ENSMUSG00000014867	chr2	26775560	26789448	-	3281	-0.948		0
Aagab	ENSMUSG00000037257	chr9	63450462	63489696	+	2600	-0.949		0
2510012J08Rik	ENSMUSG00000034889	chr10	80783848	80788996	+	2724	-0.949		0
Hic1	ENSMUSG00000043099	chr11	74978067	74983021	-	3750	-0.95		0
Cops8	ENSMUSG00000034432	chr1	92500000	92509916	+	1843	-0.95		0
Slc35d2	ENSMUSG00000033114	chr13	64197618	64230638	-	2276	-0.951		0
Dpy30	ENSMUSG00000024067	chr17	74698814	74723284	-	974	-0.952		0
Evl	ENSMUSG00000021262	chr12	109792930	109926723	+	2695	-0.952		0
Il18bp	ENSMUSG00000070427	chr7	109163593	109166669	-	1400	-0.952		0
Sftpd	ENSMUSG00000021795	chr14	41985501	41998487	-	1390	-0.952		0
Bsdc1	ENSMUSG00000040859	chr4	129138825	129165742	+	5630	-0.953		0
Pla2g4b	ENSMUSG00000033852	chr2	119853219	119868769	+	7794	-0.954		0
Polr2d	ENSMUSG00000024258	chr18	31948813	31956296	+	956	-0.954		0
Cd81	ENSMUSG00000037706	chr7	150238644	150253839	+	2270	-0.956		0
Serinc2	ENSMUSG00000023232	chr4	129930739	129956449	-	2319	-0.956		0
Haus7	ENSMUSG00000031371	chrX	70682654	70704383	-	1438	-0.957		0
Hn1	ENSMUSG00000020737	chr11	115358667	115375701	-	2149	-0.957		0
4933439C10Rik	ENSMUSG00000072893	chr11	59319164	59327149	+	5616	-0.957		0
Nfix	ENSMUSG00000001911	chr8	87223775	87324243	-	7363	-0.959		0
Cr1l	ENSMUSG00000016481	chr1	196929983	196957764	-	1787	-0.959		0
Trpv2	ENSMUSG00000018507	chr11	62387988	62414017	+	4485	-0.96		0
Cyp4b1	ENSMUSG00000028713	chr4	115297303	115320322	-	2281	-0.96		0
Cib2	ENSMUSG00000037493	chr9	54392601	54408025	-	1433	-0.96		0
Prkca	ENSMUSG00000050965	chr11	107794701	108205242	-	8992	-0.961		0
Zmat2	ENSMUSG00000001383	chr18	36953577	36959314	+	2257	-0.963		0
Slc35b4	ENSMUSG00000018999	chr6	34103380	34127111	-	9177	-0.963		0
Kctd21	ENSMUSG00000044952	chr7	104480837	104498723	+	2966	-0.964		0
Pabpn1	ENSMUSG00000022194	chr14	55511337	55517006	+	3035	-0.964		0
Vps18	ENSMUSG00000034216	chr2	119114476	119124189	+	4786	-0.965		0

Vma21-ps	ENSMUSG00000061619	chr4	52509703	52510178	-		476	-0.965	0
Htra2	ENSMUSG00000068329	chr6	83001260	83005267	-		2764	-0.965	0
Fcrls	ENSMUSG00000015852	chr3	87054680	87067660	-		2334	-0.966	0
Tmem110	ENSMUSG00000006526	chr14	31638776	31690172	+		4058	-0.967	0
Ppp1r8	ENSMUSG00000028882	chr4	132382844	132399084	-		2924	-0.967	0
Islr	ENSMUSG00000037206	chr9	58004080	58006949	-		2220	-0.968	0
Fam134b	ENSMUSG00000022270	chr15	25773019	25903442	+		3449	-0.969	0
Wdr46	ENSMUSG00000024312	chr17	34077605	34086640	+		2442	-0.97	0
Hdlbp	ENSMUSG00000034088	chr1	95302517	95375385	-		6345	-0.971	0
Srsf9	ENSMUSG00000029538	chr5	115777186	115783089	+		1360	-0.972	0
Agxt2l2	ENSMUSG00000020359	chr11	51398259	51417991	+		9699	-0.975	0
Ighmbp2	ENSMUSG00000024831	chr19	3260924	3283017	-		5299	-0.975	0
Bri3bp	ENSMUSG00000037905	chr5	125921938	125941242	+		6975	-0.976	0
Rbm8a	ENSMUSG00000038374	chr3	96433851	96437702	+		2604	-0.976	0
Gm9493	ENSMUSG00000044424	chr19	23694232	23694810	+		579	-0.976	0
Phf23	ENSMUSG00000018572	chr11	69809279	69813511	+		3273	-0.976	0
H2-M3	ENSMUSG00000016206	chr17	37407165	37411429	+		1438	-0.977	0
2310010J17Rik	ENSMUSG00000085650	chr7	97273368	97277984	-		451	-0.98	0
Fbxo25	ENSMUSG00000038365	chr8	13907803	13940521	+		2038	-0.983	0
Gdpd5	ENSMUSG00000035314	chr7	106530059	106609493	+		3582	-0.983	0
Tmem39a	ENSMUSG00000002845	chr16	38558811	38592275	+		4094	-0.984	0
Ykt6	ENSMUSG00000002741	chr11	5855696	5867783	+		2984	-0.984	0
Ctbp1	ENSMUSG00000037373	chr5	33590372	33617618	-		2254	-0.985	0
Ints1	ENSMUSG00000029547	chr5	140227236	140251632	-		7113	-0.985	0
Pcnxl3	ENSMUSG00000054874	chr19	5664635	5688908	-		8757	-0.985	0
Dhx35	ENSMUSG00000027655	chr2	158620543	158683950	+		5170	-0.985	0
Clns1a	ENSMUSG00000025439	chr7	104845144	104867389	+		1515	-0.985	0
4930420K17Rik	ENSMUSG00000079659	chr5	9100737	9118983	+		988	-0.986	0
Cgrrf1	ENSMUSG00000055128	chr14	47451809	47473868	+		3291	-0.988	0
Stx18	ENSMUSG00000029125	chr5	38429981	38529004	+		4194	-0.988	0
Gtf2i	ENSMUSG00000060261	chr5	134713704	134790630	-		7120	-0.988	0
Ndst2	ENSMUSG00000039308	chr14	21542952	21549766	-		3531	-0.988	0
Hacl1	ENSMUSG00000021884	chr14	32366091	32454472	-		5123	-0.989	0
Gm8444	ENSMUSG00000069439	chr15	81673773	81674129	-		357	-0.989	0
AI480526	ENSMUSG00000090086	chr5	123583732	123591675	-		4725	-0.992	0
Dis3l2	ENSMUSG00000053333	chr1	88600379	88946672	+		3223	-0.994	0
Prpf40b	ENSMUSG00000023007	chr15	99125518	99147449	+		4952	-0.994	0
Gspt2	ENSMUSG00000071723	chrX	91881408	91888583	+		3781	-0.995	0

Fdx1	ENSMUSG00000032051	chr9	51751412	51771661	-	3102	-0.995	0
6530401N04Rik	ENSMUSG00000020956	chr12	53089299	53107488	-	3108	-0.996	0
Fam122a	ENSMUSG00000074922	chr19	24550992	24551846	-	855	-0.996	0
Efhd2	ENSMUSG00000040659	chr4	141414057	141430835	-	2381	-0.996	0
Egfl7	ENSMUSG00000026921	chr2	26435534	26448640	+	3581	-0.996	0
Nudt9	ENSMUSG00000029310	chr5	104475325	104494398	+	2392	-0.997	0
Lmnb2	ENSMUSG00000062075	chr10	80363948	80380990	-	4286	-0.997	0
Arhgap25	ENSMUSG00000030047	chr6	87408539	87483253	-	4250	-0.999	0
Mif	ENSMUSG00000033307	chr10	75322098	75322995	-	554	-0.999	0
Akt1	ENSMUSG00000001729	chr12	113892032	113913095	-	4098	-1	-1
Mettl8	ENSMUSG00000041975	chr2	70802618	70893663	-	5617	-1.001	-1
Rnf26	ENSMUSG00000053128	chr9	43874410	43921600	-	4546	-1.001	-1
D330012F22Rik	ENSMUSG00000050973	chr7	87377749	87385892	+	3136	-1.001	-1
Zfp467	ENSMUSG00000068551	chr6	48377696	48395824	-	4255	-1.002	-1
Caskin2	ENSMUSG00000034471	chr11	115660497	115674953	-	5177	-1.004	-1
Zdhhc24	ENSMUSG00000006463	chr19	4878668	4885397	+	2916	-1.004	-1
Lrrc1	ENSMUSG00000032352	chr9	77278630	77392677	-	3217	-1.006	-1
Mrpl33	ENSMUSG00000029142	chr5	31899308	32004345	+	7473	-1.006	-1
5730508B09Rik	ENSMUSG00000050549	chr3	127572606	127599241	-	1603	-1.007	-1
2010305A19Rik	ENSMUSG00000048351	chr4	108000745	108014149	+	3851	-1.007	-1
Ppfia4	ENSMUSG00000026458	chr1	136193360	136229505	-	6072	-1.009	-1
Fhod3	ENSMUSG00000034295	chr18	24867946	25292001	+	5408	-1.009	-1
9430015G10Rik	ENSMUSG00000059939	chr4	155484091	155501374	+	3695	-1.01	-1
Mmachc	ENSMUSG00000028690	chr4	116374884	116381011	-	2224	-1.011	-1
8430410A17Rik	ENSMUSG00000030060	chr6	87863929	87886623	+	2308	-1.012	-1
Gm3150	ENSMUSG00000078297	chr15	72743784	72744235	+	452	-1.012	-1
Aco2	ENSMUSG00000022477	chr15	81702739	81745563	+	3892	-1.013	-1
Eif4ebp2	ENSMUSG00000020091	chr10	60895245	60915417	-	1786	-1.013	-1
Senp3	ENSMUSG00000005204	chr11	69486612	69495586	-	3761	-1.014	-1
D11Wsu47e	ENSMUSG00000041623	chr11	113545724	113560632	+	2497	-1.014	-1
Sip1	ENSMUSG00000060121	chr12	60114380	60129455	+	1743	-1.015	-1

B4galt7	ENSMUSG00000021504	chr13	55701257	55711804	+	2896	-1.019		-1
H2-DMb1	ENSMUSG00000079547	chr17	34290017	34297168	+	1478	-1.02		-1
Tmem183a	ENSMUSG00000042305	chr1	136242674	136258576	-	3169	-1.022		-1
Tyw1	ENSMUSG00000056310	chr5	130731489	130817433	+	7388	-1.023		-1
Cdc42se1	ENSMUSG00000046722	chr3	95032654	95040331	+	3258	-1.024		-1
Slc35e1	ENSMUSG00000019731	chr8	75001895	75016513	-	7115	-1.024		-1
Xbp1	ENSMUSG00000020484	chr11	5420662	5425896	+	3248	-1.024		-1
Rbp1	ENSMUSG00000046402	chr9	98323380	98346970	+	2628	-1.025		-1
Zbed3	ENSMUSG00000041995	chr13	96095192	96107796	+	1894	-1.026		-1
Pmpcb	ENSMUSG00000029017	chr5	21242959	21262970	+	5141	-1.026		-1
Pdcd4	ENSMUSG00000024975	chr19	53966721	54004351	+	2556	-1.026		-1
Ei24	ENSMUSG00000062762	chr9	36586740	36604938	-	2249	-1.027		-1
Rabl2	ENSMUSG00000022621	chr15	89412964	89422354	-	2960	-1.029		-1
Tenc1	ENSMUSG00000037003	chr15	101930909	101946832	+	4856	-1.029		-1
Acap3	ENSMUSG00000029033	chr4	155265984	155281360	+	5314	-1.03		-1
Galns	ENSMUSG00000015027	chr8	125102138	125135304	-	2502	-1.03		-1
Itga7	ENSMUSG00000025348	chr10	128370869	128395333	+	4258	-1.03		-1
Ccdc142	ENSMUSG00000079511	chr6	83051611	83059933	+	3763	-1.032		-1
Ngdn	ENSMUSG00000022204	chr14	55634291	55642972	+	1198	-1.033		-1
Gpx2	ENSMUSG00000042808	chr12	77893322	77896541	-	1067	-1.033		-1
Mlec	ENSMUSG00000048578	chr5	115592990	115608188	-	6010	-1.034		-1
Lpin1	ENSMUSG00000020593	chr12	16542475	16596576	-	5725	-1.034		-1
Sec22a	ENSMUSG00000034473	chr16	35311217	35364004	-	5223	-1.035		-1
Fbxl8	ENSMUSG00000033313	chr8	107788548	107793226	+	2103	-1.037		-1
A130010J15Rik	ENSMUSG00000079144	chr1	194994243	195002497	+	2559	-1.038		-1
Fntb	ENSMUSG00000033373	chr12	77866576	78022399	+	3987	-1.038		-1
2310030G06Rik	ENSMUSG00000032062	chr9	50547796	50554626	-	1242	-1.038		-1
Neurl3	ENSMUSG00000047180	chr1	36321447	36330270	-	2583	-1.039		-1
As3mt	ENSMUSG00000003559	chr19	46781933	46815585	+	1751	-1.04		-1
Mfn2	ENSMUSG00000029020	chr4	147247708	147278813	-	5194	-1.041		-1
Cyb5r1	ENSMUSG00000026456	chr1	136302358	136308317	+	4215	-1.043		-1
Palm	ENSMUSG00000035863	chr10	79256317	79283641	+	2651	-1.043		-1
Sap30l	ENSMUSG00000020519	chr11	57615139	57623719	+	1151	-1.044		-1
Rae1	ENSMUSG00000027509	chr2	172825618	172841240	+	2554	-1.044		-1
5430405H02Rik	ENSMUSG00000085741	chr2	156678166	156688681	-	1482	-1.045		-1
Vps16	ENSMUSG00000027411	chr2	130250075	130270005	+	3727	-1.045		-1
Nif3l1	ENSMUSG00000026036	chr1	58501995	58538660	+	3842	-1.045		-1
Smarcal1	ENSMUSG00000039354	chr1	72629825	72679708	+	5728	-1.046		-1

Atn1	ENSMUSG00000004263	chr6	124689207	124706542	-	5085	-1.049	-1
Dap3	ENSMUSG00000068921	chr3	88724725	88755103	-	5885	-1.049	-1
1700086O06Rik	ENSMUSG00000086988	chr18	38398059	38410219	-	1931	-1.05	-1
2610524H06Rik	ENSMUSG00000092486	chr5	115271944	115273477	-	947	-1.052	-1
Eepd1	ENSMUSG00000036611	chr9	25289132	25411695	+	2935	-1.052	-1
Ccdc9	ENSMUSG00000041375	chr7	16859391	16872144	-	5507	-1.053	-1
BC088983	ENSMUSG00000038215	chr8	59010319	59029844	-	2817	-1.053	-1
Zfp362	ENSMUSG00000028799	chr4	128450332	128483289	-	3052	-1.053	-1
Zfp185	ENSMUSG00000031351	chrX	70232678	70276882	+	3707	-1.055	-1
Colec11	ENSMUSG00000036655	chr12	29279038	29308155	-	1383	-1.056	-1
Hopx	ENSMUSG00000059325	chr5	77515468	77544186	-	2099	-1.056	-1
Eif2b1	ENSMUSG00000029388	chr5	125020222	125029140	-	2577	-1.058	-1
Mbp	ENSMUSG00000041607	chr18	82644538	82755029	+	8403	-1.058	-1
Smarca2	ENSMUSG00000024921	chr19	26679605	26852811	+	7854	-1.058	-1
Hmox2	ENSMUSG00000004070	chr16	4726361	4766742	+	2217	-1.058	-1
Cxcl15	ENSMUSG00000029375	chr5	91223560	91232093	+	2127	-1.058	-1
Mtus1	ENSMUSG00000045636	chr8	42076268	42219080	-	10078	-1.059	-1
Ttll12	ENSMUSG00000016757	chr15	83405549	83425587	-	4010	-1.06	-1
Gins1	ENSMUSG00000027454	chr2	150731136	150757016	+	1516	-1.061	-1
Csrp2bp	ENSMUSG00000027425	chr2	144194719	144233412	+	4699	-1.062	-1
Pqlc1	ENSMUSG00000034006	chr18	80450031	80489464	+	6100	-1.062	-1
Got2	ENSMUSG00000031672	chr8	98388034	98412447	-	2585	-1.062	-1
Bend5	ENSMUSG00000028545	chr4	111087611	111132903	+	2105	-1.064	-1
Pigl	ENSMUSG00000014245	chr11	62271787	62329795	+	4707	-1.065	-1
Arpc5l	ENSMUSG00000026755	chr2	38863596	38871397	+	4438	-1.065	-1
Caml	ENSMUSG00000021501	chr13	55724366	55733772	+	1755	-1.066	-1
Sirt5	ENSMUSG00000054021	chr13	43460882	43490571	+	2167	-1.066	-1
Slc25a22	ENSMUSG00000019082	chr7	148615638	148623791	-	4041	-1.066	-1
Rrnad1	ENSMUSG00000004896	chr3	87726523	87734639	-	6342	-1.068	-1
Zfp385a	ENSMUSG00000000552	chr15	103144319	103170517	-	2395	-1.069	-1
Agbl5	ENSMUSG00000029165	chr5	31191067	31209338	+	7053	-1.07	-1
Camk1	ENSMUSG00000030272	chr6	113284118	113293978	-	6067	-1.071	-1
Scp2	ENSMUSG00000028603	chr4	107716444	107817603	-	3478	-1.073	-1
Rnf113a1	ENSMUSG00000036537	chrX	34731240	34732457	+	1218	-1.073	-1
Polg2	ENSMUSG00000020718	chr11	106629567	106640851	-	4356	-1.073	-1
Slc35b3	ENSMUSG00000021432	chr13	39024009	39052744	-	2214	-1.074	-1
Gars	ENSMUSG00000029777	chr6	54987995	55029498	+	2390	-1.077	-1

1190003 J15Rik	ENSMUSG00000025481	chr7	148020917	148023865	+	1337	-1.078	-1
Abcf1	ENSMUSG00000038762	chr17	36093764	36106706	-	4410	-1.078	-1
Fam32a	ENSMUSG00000003039	chr8	74743629	74747663	+	1910	-1.081	-1
Mical3	ENSMUSG00000051586	chr6	120881725	120953171	-	7398	-1.081	-1
Lipt2	ENSMUSG00000030725	chr7	107307787	107309875	+	1663	-1.082	-1
Clcn7	ENSMUSG00000036636	chr17	25270336	25299049	+	5292	-1.082	-1
Oas1a	ENSMUSG00000052776	chr5	121346265	121357536	-	1998	-1.082	-1
Lrrc28	ENSMUSG00000030556	chr7	74658297	74790122	-	4309	-1.083	-1
Fcgrt	ENSMUSG00000003420	chr7	52348363	52359192	-	1738	-1.084	-1
Dctn1	ENSMUSG00000031865	chr6	83115914	83150111	+	6509	-1.084	-1
Harbi1	ENSMUSG00000027243	chr2	91551009	91561702	+	5588	-1.087	-1
Ntpcr	ENSMUSG00000031851	chr8	128253863	128272135	+	2673	-1.088	-1
Pid1	ENSMUSG00000045658	chr1	84032868	84360755	-	7054	-1.089	-1
Elac2	ENSMUSG00000020549	chr11	64792540	64815571	+	3479	-1.09	-1
Scamp5	ENSMUSG00000040722	chr9	57289135	57315831	-	3190	-1.09	-1
Ncstn	ENSMUSG00000003458	chr1	173996144	174012926	-	3975	-1.09	-1
Ddit4	ENSMUSG00000020108	chr10	59412423	59414518	-	1685	-1.09	-1
Rps4x	ENSMUSG00000031320	chrX	99380280	99384733	-	1631	-1.094	-1
Pth1r	ENSMUSG00000032492	chr9	110624592	110649649	-	2727	-1.096	-1
Ppil1	ENSMUSG00000024007	chr17	29387748	29401103	-	2163	-1.096	-1
Snrnp35	ENSMUSG00000029402	chr5	124933143	124941133	+	1347	-1.096	-1
Derl2	ENSMUSG00000018442	chr11	70820666	70833343	-	8049	-1.097	-1
Bre	ENSMUSG00000052139	chr5	32000057	32387335	+	4781	-1.097	-1
BC0249 78	ENSMUSG00000078786	chr7	27980800	27990405	+	2756	-1.098	-1
Ppih	ENSMUSG00000060288	chr4	118972615	118993151	-	1894	-1.098	-1
Lace1	ENSMUSG00000038302	chr10	42032391	42198371	-	2705	-1.1	-1
Pigc	ENSMUSG00000026698	chr1	163899317	163957237	+	4002	-1.1	-1
Mpv17	ENSMUSG00000090262	chr5	31443033	31460526	-	3258	-1.1	-1
Ddb1	ENSMUSG00000024740	chr19	10680058	10704306	+	4215	-1.101	-1
Rcn3	ENSMUSG00000019539	chr7	52338283	52347591	-	1604	-1.101	-1
Cox5a	ENSMUSG00000000088	chr9	57369039	57380231	+	690	-1.101	-1
2400003 C14Rik	ENSMUSG00000031729	chr8	112195222	112217194	-	3674	-1.103	-1
Obsl1	ENSMUSG00000026211	chr1	75475885	75503027	-	8695	-1.103	-1
Mtmr14	ENSMUSG00000030269	chr6	113187837	113231386	+	2879	-1.105	-1
Leprel2	ENSMUSG00000023191	chr6	124791107	124807770	-	3930	-1.106	-1
Fn3krp	ENSMUSG00000039253	chr11	121282715	121292602	+	2520	-1.107	-1
Hsf1	ENSMUSG00000022556	chr15	76307865	76331402	+	2178	-1.107	-1
Ppa1	ENSMUSG00000020089	chr10	61111300	61136916	+	2902	-1.107	-1
Ptpn6	ENSMUSG00000004266	chr6	124670725	124688732	-	2802	-1.109	-1
Icam1	ENSMUSG00000037405	chr9	20820404	20833241	+	2540	-1.11	-1
Mtap7	ENSMUSG00000019996	chr10	19868277	20001393	+	4349	-1.11	-1

Tsc22d4	ENSMUSG00000029723	chr5	138186958	138209678	+	7516	-1.11	-1
Matn2	ENSMUSG00000022324	chr15	34236436	34366029	+	3609	-1.112	-1
Ino80e	ENSMUSG00000030689	chr7	133995094	134005891	-	4648	-1.112	-1
Ndufb3	ENSMUSG00000026032	chr1	58643228	58652808	+	763	-1.113	-1
Ruvbl2	ENSMUSG00000003868	chr7	52677222	52693466	-	2573	-1.113	-1
Hoxa11 as	ENSMUSG00000086427	chr6	52194392	52199798	+	3073	-1.115	-1
Tomm2 0	ENSMUSG00000058779	chr8	129458043	129469723	-	1409	-1.116	-1
Rcan1	ENSMUSG00000022951	chr16	92392198	92466391	-	2506	-1.117	-1
Ifitm3	ENSMUSG00000025492	chr7	148195485	148196669	-	678	-1.117	-1
AU0222 52	ENSMUSG00000078584	chr4	118876175	118905317	-	9570	-1.118	-1
Penk	ENSMUSG00000045573	chr4	4060678	4065966	-	1634	-1.12	-1
AI46413 1	ENSMUSG00000046312	chr4	41442637	41450109	-	4701	-1.121	-1
B9d2	ENSMUSG00000063439	chr7	26466177	26471577	+	1021	-1.121	-1
Gm1673	ENSMUSG00000070858	chr5	34326086	34327662	+	665	-1.121	-1
Rinl	ENSMUSG00000051735	chr7	29573988	29583982	+	2871	-1.122	-1
Top1mt	ENSMUSG00000000934	chr15	75487465	75509230	-	1903	-1.123	-1
Chrd	ENSMUSG00000006958	chr16	20733200	20742457	+	5838	-1.123	-1
Pip4k2c	ENSMUSG00000025417	chr10	126634123	126648678	-	3374	-1.124	-1
Bub3	ENSMUSG00000066979	chr7	138703905	138715412	+	2055	-1.125	-1
Igkv4-55	ENSMUSG00000076558	chr6	69557269	69557795	-	352	-1.126	-1
Rad52	ENSMUSG00000030166	chr6	119852716	119872846	+	3371	-1.126	-1
Slc39a9	ENSMUSG00000048833	chr12	81745202	81784328	+	5229	-1.128	-1
Spg7	ENSMUSG00000000738	chr8	125586842	125621660	+	4188	-1.128	-1
Pigs	ENSMUSG00000041958	chr11	78141917	78156284	+	2501	-1.129	-1
Ogdh	ENSMUSG00000020456	chr11	6191636	6256645	+	5456	-1.129	-1
Cpxm2	ENSMUSG00000030862	chr7	139234499	139346422	-	4450	-1.131	-1
Myl6b	ENSMUSG00000039824	chr10	127931213	127935741	-	1026	-1.132	-1
Acvrl1	ENSMUSG00000000530	chr15	100958953	100975767	+	4267	-1.132	-1
Clk3	ENSMUSG00000032316	chr9	57598518	57613667	-	2494	-1.132	-1
Wdr81	ENSMUSG00000045374	chr11	75254446	75268219	-	7038	-1.133	-1
Raf1	ENSMUSG00000000441	chr6	115568085	115626653	-	6363	-1.134	-1
Armcx2	ENSMUSG00000033436	chrX	131338684	131343760	-	4532	-1.135	-1
Ptger3	ENSMUSG00000040016	chr3	157229856	157307722	+	4558	-1.136	-1
Lrpap1	ENSMUSG00000029103	chr5	35434139	35448415	-	4127	-1.136	-1
Agpat6	ENSMUSG00000031545	chr8	24283418	24318925	-	3944	-1.136	-1
Pias3	ENSMUSG00000028101	chr3	96500307	96509993	+	3918	-1.137	-1
Zfp212	ENSMUSG00000052763	chr6	47870475	47882638	+	3002	-1.137	-1
St3gal3	ENSMUSG00000028538	chr4	117604759	117807519	-	2768	-1.138	-1
Scaf1	ENSMUSG00000038406	chr7	52258318	52271733	-	8368	-1.138	-1
Ccdc21	ENSMUSG00000037443	chr4	133685773	133743027	-	5269	-1.139	-1

Rbbp4	ENSMUSG00000057236	chr4	128984344	129012614	-	8744	-1.14		-1
Ninj1	ENSMUSG00000037966	chr13	49282854	49291613	+	1570	-1.141		-1
Aldoa	ENSMUSG00000030695	chr7	133938748	133944265	-	2754	-1.142		-1
Gm16314	ENSMUSG00000086965	chr16	18498994	18501751	+	865	-1.143		-1
Igkv4-57-1	ENSMUSG00000076555	chr6	69494353	69494886	-	358	-1.143		-1
2010107E04Rik	ENSMUSG00000021290	chr12	113199587	113205188	-	365	-1.143		-1
Vit	ENSMUSG00000024076	chr17	78907480	79026737	+	2631	-1.145		-1
Ccpg1	ENSMUSG00000034563	chr9	72833236	72864147	+	5113	-1.146		-1
Thg1l	ENSMUSG00000011254	chr11	45760345	45768996	-	3015	-1.147		-1
Uggt1	ENSMUSG00000037470	chr1	36198375	36301150	-	9855	-1.147		-1
Mcm5	ENSMUSG00000005410	chr8	77633427	77652338	+	3498	-1.148		-1
Ly75	ENSMUSG00000026980	chr2	60130160	60221360	-	7340	-1.149		-1
Nat6	ENSMUSG00000079334	chr9	107481218	107486379	+	3152	-1.15		-1
Lzts2	ENSMUSG00000035342	chr19	45089666	45101594	+	2825	-1.151		-1
1810037I17Rik	ENSMUSG00000054091	chr3	122627280	122629111	+	1010	-1.151		-1
Cox10	ENSMUSG00000042148	chr11	63776129	63892970	-	3037	-1.153		-1
Pigu	ENSMUSG00000038383	chr2	155072732	155183166	-	9929	-1.153		-1
Gm13375	ENSMUSG00000075514	chr2	20890681	20891624	+	854	-1.153		-1
Spata24	ENSMUSG00000024352	chr18	35816344	35821840	-	967	-1.153		-1
Smyd2	ENSMUSG00000026603	chr1	191704371	191746242	-	2717	-1.154		-1
Hbb-b2	ENSMUSG00000073940	chr7	110961038	110962510	-	703	-1.154		-1
Lrrc8e	ENSMUSG00000046589	chr8	4226827	4237470	+	3878	-1.156		-1
0610010O12Rik	ENSMUSG00000046727	chr18	36505273	36562634	+	2840	-1.158		-1
2900064A13Rik	ENSMUSG00000055943	chr2	112295154	112312319	+	5826	-1.158		-1
Isg15	ENSMUSG00000035692	chr4	155573533	155574927	-	756	-1.162		-1
Rffl	ENSMUSG00000020696	chr11	82615951	82684712	-	5922	-1.163		-1
Sirt2	ENSMUSG00000015149	chr7	29551754	29573684	+	3568	-1.164		-1
Sap30bp	ENSMUSG00000020755	chr11	115794596	115828039	+	3708	-1.165		-1
Insl6	ENSMUSG00000050957	chr19	29395837	29399846	-	705	-1.165		-1
Edem1	ENSMUSG00000030104	chr6	108778635	108809350	+	5858	-1.166		-1
Nudt19	ENSMUSG00000034875	chr7	36332204	36341323	-	2680	-1.166		-1
Gpr132	ENSMUSG00000021298	chr12	114089087	114099127	-	2439	-1.166		-1
Coq5	ENSMUSG00000041733	chr5	115729675	115746981	+	2037	-1.166		-1
Icam2	ENSMUSG00000001029	chr11	106238970	106249389	-	1904	-1.167		-1
Lrsam1	ENSMUSG00000026792	chr2	32780740	32817134	-	4545	-1.167		-1
Tbc1d20	ENSMUSG00000027465	chr2	152119564	152139732	+	4171	-1.168		-1
Noc4l	ENSMUSG00000033294	chr5	111077437	111082436	-	2505	-1.168		-1

Ccdc97	ENSMUSG00000002608	chr7	26496125	26504107	-	3939	-1.168	-1
Stc2	ENSMUSG00000020303	chr11	31257307	31270074	-	5175	-1.168	-1
Gpsm1	ENSMUSG00000026930	chr2	26171035	26203757	+	5383	-1.169	-1
BC002230	ENSMUSG00000021179	chr12	101363662	101397863	-	3723	-1.171	-1
Hps6	ENSMUSG00000074811	chr19	46077968	46080663	+	2696	-1.171	-1
Nol6	ENSMUSG00000028430	chr4	41061460	41071488	-	5242	-1.171	-1
Mrs2	ENSMUSG00000021339	chr13	25084352	25112248	-	2035	-1.173	-1
Atp6v1e1	ENSMUSG00000019210	chr6	120745263	120772703	-	1203	-1.174	-1
Tfcp2	ENSMUSG00000009733	chr15	100333230	100382439	-	3349	-1.174	-1
Aars	ENSMUSG00000031960	chr8	113557044	113581564	+	9282	-1.174	-1
Hnrnpl	ENSMUSG00000015165	chr7	29593560	29607285	+	5953	-1.176	-1
Snrpg	ENSMUSG00000057278	chr6	86321534	86328896	+	772	-1.176	-1
Mpp2	ENSMUSG00000017314	chr11	101918329	101949829	-	4807	-1.176	-1
Chmp4b	ENSMUSG00000038467	chr2	154477441	154520521	+	2027	-1.176	-1
Pcid2	ENSMUSG00000038542	chr8	13077189	13105459	-	3090	-1.178	-1
Clec11a	ENSMUSG00000004473	chr7	51559140	51562329	-	1952	-1.178	-1
Cops7b	ENSMUSG00000026240	chr1	88479479	88503553	+	3118	-1.179	-1
Fbln1	ENSMUSG00000006369	chr15	85036379	85116965	+	4368	-1.179	-1
Ggcx	ENSMUSG00000053460	chr6	72364302	72380706	+	3244	-1.182	-1
Hnf1b	ENSMUSG00000020679	chr11	83663565	83719419	+	3296	-1.182	-1
Hcst	ENSMUSG00000064109	chr7	31202732	31204873	-	441	-1.183	-1
Ndrg3	ENSMUSG00000027634	chr2	156753081	156817792	-	8388	-1.184	-1
0610007N19Rik	ENSMUSG00000090401	chr15	32170324	32174417	-	1482	-1.184	-1
Bak1	ENSMUSG00000057789	chr17	27156755	27165954	-	2407	-1.185	-1
Thoc6	ENSMUSG00000041319	chr17	23805581	23810849	-	2026	-1.185	-1
Sumf2	ENSMUSG00000025538	chr5	130322866	130339928	+	2669	-1.188	-1
Itpa	ENSMUSG00000074797	chr2	130493346	130507350	+	1963	-1.188	-1
5031425E22Rik	ENSMUSG00000073147	chr5	22937628	22939737	-	2110	-1.191	-1
Dhx30	ENSMUSG00000032480	chr9	109986824	110020334	-	9786	-1.191	-1
Rnf39	ENSMUSG00000036492	chr17	37079863	37084931	+	1795	-1.194	-1
Svip	ENSMUSG00000074093	chr7	59252535	59261388	-	3059	-1.195	-1
Glul	ENSMUSG00000026473	chr1	155747074	155756853	+	3329	-1.195	-1
Grn	ENSMUSG00000034708	chr11	102291629	102298362	+	3474	-1.196	-1
Impdh2	ENSMUSG00000062867	chr9	108462774	108467895	+	1681	-1.197	-1
Iglv1	ENSMUSG00000076934	chr16	19085110	19085553	-	351	-1.197	-1
Zbtb16	ENSMUSG00000066687	chr9	48462416	48644050	-	5100	-1.198	-1
Ndufs5	ENSMUSG00000028648	chr4	123389953	123395445	-	860	-1.198	-1
Hif1an	ENSMUSG00000036450	chr19	44637340	44650764	+	6606	-1.198	-1
Th1l	ENSMUSG00000016253	chr2	174241305	174253003	+	2883	-1.199	-1
Psmb7	ENSMUSG00000026750	chr2	38443556	38499607	-	1214	-1.203	-1

Cdc42ep4	ENSMUSG00000041598	chr11	113588164	113613195	-		3643	-1.205	-1
Gtl3	ENSMUSG00000031796	chr8	97944152	97958769	-		1292	-1.205	-1
Gm17251	ENSMUSG00000090952	chr17	33896937	33898815	+		925	-1.205	-1
Scnn1b	ENSMUSG00000030873	chr7	129008591	129062029	+		2592	-1.207	-1
Arpc4	ENSMUSG00000079426	chr6	113328107	113340442	+		2323	-1.207	-1
Rps24	ENSMUSG00000025290	chr14	25309903	25315368	+		901	-1.209	-1
Edc3	ENSMUSG00000038957	chr9	57556347	57600306	+		5804	-1.211	-1
Thoc5	ENSMUSG00000034274	chr11	4795323	4828870	+		2861	-1.211	-1
Tor1b	ENSMUSG00000026848	chr2	30808479	30814535	+		5327	-1.212	-1
Gsto2	ENSMUSG00000025069	chr19	47940035	47960814	+		1734	-1.213	-1
Abhd4	ENSMUSG00000040997	chr14	54878907	54888829	+		2391	-1.213	-1
1600002H07Rik	ENSMUSG00000024118	chr17	24351999	24357796	-		2551	-1.214	-1
Dus2l	ENSMUSG00000031901	chr8	108515237	108577740	+		3367	-1.214	-1
Med24	ENSMUSG00000017210	chr11	98565905	98590749	-		6528	-1.215	-1
Tctn3	ENSMUSG00000025008	chr19	40670936	40686723	-		3273	-1.218	-1
Stk11ip	ENSMUSG00000026213	chr1	75518104	75533910	+		5743	-1.219	-1
Rab43	ENSMUSG00000030055	chr6	87738847	87762158	-		5526	-1.22	-1
Pik3r2	ENSMUSG00000031834	chr8	73292075	73300612	-		4176	-1.221	-1
D10Wsu52e	ENSMUSG00000001783	chr10	85401382	85420568	-		2615	-1.221	-1
Tinf2	ENSMUSG00000007589	chr14	56295455	56300654	-		2959	-1.222	-1
Gabarapl2	ENSMUSG00000031950	chr8	114464603	114477512	+		2325	-1.223	-1
Dda1	ENSMUSG00000074247	chr8	73993098	73999997	+		2846	-1.224	-1
Tubg1	ENSMUSG00000035198	chr11	100981252	100987733	+		2266	-1.224	-1
Tomm40	ENSMUSG00000002984	chr7	20286662	20300787	-		4239	-1.225	-1
Rab11b	ENSMUSG00000077450	chr17	33882895	33897475	-		2643	-1.225	-1
Sigmar1	ENSMUSG00000036078	chr4	41685366	41703030	-		3136	-1.225	-1
Abca5	ENSMUSG00000018800	chr11	110130683	110199030	-		11437	-1.225	-1
Itgb7	ENSMUSG00000001281	chr15	102046426	102062366	-		2743	-1.226	-1
Fam134c	ENSMUSG00000017802	chr11	100957636	100981207	-		3589	-1.226	-1
Parp1	ENSMUSG00000026496	chr1	182499086	182531384	+		3846	-1.226	-1
Chka	ENSMUSG00000024843	chr19	3851773	3894369	+		12024	-1.227	-1
Rab4a	ENSMUSG00000019478	chr8	126329885	126359187	+		1757	-1.227	-1
Mark2	ENSMUSG00000024969	chr19	7349886	7416350	-		7948	-1.228	-1
Stat5b	ENSMUSG00000020919	chr11	100642045	100712038	-		5539	-1.229	-1
Idh3b	ENSMUSG00000027406	chr2	130105045	130110283	-		3422	-1.233	-1
Coq7	ENSMUSG00000030652	chr7	125653173	125676870	-		4003	-1.233	-1
Tsfm	ENSMUSG00000040521	chr10	126448628	126467896	-		2904	-1.234	-1
Nutf2	ENSMUSG00000008450	chr8	108384534	108404302	+		1966	-1.235	-1
Rnd1	ENSMUSG00000054855	chr15	98493852	98507892	-		2967	-1.235	-1
Lamb2	ENSMUSG00000052911	chr9	108382193	108392861	+		5624	-1.236	-1

Slc15a4	ENSMUSG00000029416	chr5	128076034	128113267	-	2943	-1.236	-1
Btf3	ENSMUSG00000021660	chr13	99079851	99086961	-	1739	-1.237	-1
Cisd3	ENSMUSG00000078695	chr11	97547140	97549935	+	833	-1.238	-1
Acad11	ENSMUSG00000090150	chr9	103942992	104029976	+	3996	-1.239	-1
Ier2	ENSMUSG00000053560	chr8	87185230	87186751	-	1522	-1.241	-1
Rpl31	ENSMUSG00000073702	chr1	39424696	39428753	+	1037	-1.242	-1
Ddx54	ENSMUSG00000029599	chr5	121063128	121078599	+	3940	-1.242	-1
Ttc4	ENSMUSG00000025413	chr4	106334861	106351549	-	3568	-1.245	-1
Paqr4	ENSMUSG00000023909	chr17	23873153	23877297	-	2507	-1.247	-1
Mrpl30	ENSMUSG00000026087	chr1	37947322	37955380	+	1969	-1.247	-1
Pias4	ENSMUSG00000004934	chr10	80616011	80630668	-	4582	-1.247	-1
Ccdc61	ENSMUSG00000074358	chr7	19476232	19495764	-	2122	-1.248	-1
Bud31	ENSMUSG00000038722	chr5	145901231	145908947	+	1609	-1.248	-1
Rnf128	ENSMUSG00000031438	chrX	136097855	136207684	+	3464	-1.25	-1
Iqgap2	ENSMUSG00000021676	chr13	96397132	96661877	-	5780	-1.251	-1
Plin4	ENSMUSG00000002831	chr17	56240014	56249225	-	5755	-1.252	-1
Lrp3	ENSMUSG00000001802	chr7	35984852	36000389	-	4765	-1.253	-1
Prkcdbp	ENSMUSG00000037060	chr7	112629121	112630711	-	1035	-1.254	-1
Mfsd7a	ENSMUSG00000029490	chr5	108870073	108877910	-	2508	-1.256	-1
Ly6d	ENSMUSG00000034634	chr15	74592486	74593997	-	691	-1.256	-1
Zfp651	ENSMUSG00000013419	chr9	121669151	121680860	+	6353	-1.256	-1
Nicn1	ENSMUSG00000032606	chr9	108192779	108198829	+	2114	-1.26	-1
Tmem1 40	ENSMUSG00000057137	chr6	34813146	34824946	+	5687	-1.26	-1
Cops4	ENSMUSG00000035297	chr5	100947328	100976822	+	5006	-1.26	-1
Osbpl2	ENSMUSG00000039050	chr2	179854011	179897385	+	4187	-1.261	-1
Plac8	ENSMUSG00000029322	chr5	100982744	101001264	-	1592	-1.261	-1
Eps8l1	ENSMUSG00000006154	chr7	4412276	4431006	+	4953	-1.262	-1
Mocos	ENSMUSG00000039616	chr18	24812192	24860057	+	2884	-1.263	-1
Itpkb	ENSMUSG00000038855	chr1	182260607	182354933	+	6256	-1.263	-1
Laptm4a	ENSMUSG00000020585	chr12	8928130	8945548	+	2113	-1.263	-1
Psmf1	ENSMUSG00000032869	chr2	151541548	151569922	-	6441	-1.264	-1
Nfia	ENSMUSG00000028565	chr4	97439425	97868605	+	10945	-1.266	-1
Poldip3	ENSMUSG00000041815	chr15	82956408	82979814	-	5679	-1.267	-1
Tmem1 50a	ENSMUSG00000055912	chr6	72305441	72309756	+	1546	-1.268	-1
Tmem5 0b	ENSMUSG00000022964	chr16	91574748	91598045	-	2446	-1.269	-1
Smad6	ENSMUSG00000036867	chr9	63800883	63869866	-	3205	-1.27	-1
Ankrd46	ENSMUSG00000048307	chr15	36407423	36426546	-	2480	-1.271	-1
Bid	ENSMUSG00000004446	chr6	120841948	120866871	-	3550	-1.271	-1
Ppp1r14 b	ENSMUSG00000056612	chr19	7049538	7051814	+	919	-1.271	-1
Fdxacb1	ENSMUSG00000037845	chr9	50576341	50581453	+	3256	-1.272	-1

Psmc4	ENSMUSG00000030603	chr7	28826726	28835120	-	2169	-1.274	-1
Wwp2	ENSMUSG00000031930	chr8	109960298	110082494	+	4561	-1.275	-1
Lcmt1	ENSMUSG00000030763	chr7	130521496	130573872	+	1369	-1.275	-1
Fam160a2	ENSMUSG00000044465	chr7	112527124	112548568	-	4170	-1.276	-1
Ttc1	ENSMUSG00000041278	chr11	43543014	43561510	-	2032	-1.276	-1
Dnajc11	ENSMUSG00000039768	chr4	151307800	151356246	+	10678	-1.277	-1
Lsm1	ENSMUSG00000037296	chr8	26896063	26914447	+	2360	-1.277	-1
Commd6	ENSMUSG00000075486	chr14	102032983	102039903	-	1322	-1.278	-1
Lrdd	ENSMUSG00000025507	chr7	148624012	148629924	-	4428	-1.281	-1
1500010J02Rik	ENSMUSG00000020898	chr11	68829413	68849975	+	6797	-1.281	-1
Ramp2	ENSMUSG00000001240	chr11	101107342	101120860	+	3301	-1.282	-1
Dynlrb1	ENSMUSG00000047459	chr2	155062269	155076013	+	1009	-1.283	-1
Zfp276	ENSMUSG00000001065	chr8	125778095	125793645	+	5530	-1.283	-1
Pdia4	ENSMUSG00000025823	chr6	47746141	47763511	-	2480	-1.284	-1
Uckl1	ENSMUSG00000089917	chr2	181303854	181319597	-	4629	-1.285	-1
Pomt1	ENSMUSG00000039254	chr2	32092110	32110525	+	6411	-1.285	-1
1810063B05Rik	ENSMUSG00000051671	chr8	128946401	128949334	+	762	-1.285	-1
Xpnpep1	ENSMUSG00000025027	chr19	53017907	53113123	-	2737	-1.286	-1
Erp44	ENSMUSG00000028343	chr4	48206195	48292430	-	3418	-1.286	-1
Ighv14-4	ENSMUSG00000076666	chr12	115414655	115415073	-	333	-1.287	-1
Snx32	ENSMUSG00000056185	chr19	5495278	5510489	-	1650	-1.289	-1
Fiz1	ENSMUSG00000061374	chr7	4958659	4966299	-	2834	-1.29	-1
Pfn1	ENSMUSG00000018293	chr11	70465352	70468146	-	1026	-1.29	-1
Actr1a	ENSMUSG00000025228	chr19	46451304	46470225	-	2742	-1.29	-1
Lefty1	ENSMUSG00000038793	chr1	182865153	182868531	+	1758	-1.291	-1
Cdc26	ENSMUSG00000066149	chr4	62044688	62069676	-	7180	-1.291	-1
Wbp2	ENSMUSG00000034341	chr11	115939887	115948309	-	3177	-1.291	-1
Tmco7	ENSMUSG00000041949	chr8	109206968	109375339	+	4409	-1.291	-1
1700029G01Rik	ENSMUSG00000073758	chr4	125827846	125840735	-	2717	-1.291	-1
Nob1	ENSMUSG00000003848	chr8	109936386	109948951	-	3486	-1.292	-1
Ttc5	ENSMUSG00000006288	chr14	51385090	51405193	-	1841	-1.294	-1
Prmt10	ENSMUSG00000037134	chr8	80073296	80105237	+	5679	-1.295	-1
Atp2a3	ENSMUSG00000020788	chr11	72774671	72806546	+	5281	-1.295	-1
Auh	ENSMUSG00000021460	chr13	52930488	53025050	-	9580	-1.296	-1
Cog2	ENSMUSG00000031979	chr8	127044667	127075908	+	4778	-1.296	-1
1600020E01Rik	ENSMUSG00000086488	chr6	86476265	86514436	+	2092	-1.298	-1
Pbx2	ENSMUSG00000034673	chr17	34728211	34734345	+	3640	-1.299	-1

Def6	ENSMUSG00000002257	chr17	28344723	28365553	+	2524	-1.299	-1
Mfap4	ENSMUSG00000042436	chr11	61298933	61302402	+	1922	-1.299	-1
Cds2	ENSMUSG00000058793	chr2	132088884	132137786	+	9125	-1.299	-1
Mau2	ENSMUSG00000031858	chr8	72540022	72566633	-	5372	-1.3	-1
Id1	ENSMUSG00000042745	chr2	152561987	152563146	+	1160	-1.301	-1
Myh14	ENSMUSG00000030739	chr7	51861173	51926213	-	7247	-1.302	-1
Ada	ENSMUSG00000017697	chr2	163552320	163575975	-	2002	-1.303	-1
Psma7	ENSMUSG00000027566	chr2	179771078	179777138	-	3072	-1.303	-1
Ppfibp2	ENSMUSG00000036528	chr7	114738565	114892096	+	4729	-1.304	-1
Mtss1l	ENSMUSG00000033763	chr8	113245376	113265300	+	5311	-1.304	-1
Arhgef2 5	ENSMUSG00000019467	chr10	126619577	126627110	-	2387	-1.304	-1
BC0164 95	ENSMUSG00000037847	chr19	18706440	18726684	+	3345	-1.305	-1
Dscr3	ENSMUSG00000022898	chr16	94719390	94748437	-	3670	-1.305	-1
Hsbp1	ENSMUSG00000031839	chr8	121868438	121872827	+	1163	-1.305	-1
Asrgl1	ENSMUSG00000024654	chr19	9184358	9210126	-	4297	-1.305	-1
Sf3a2	ENSMUSG00000020211	chr10	80260943	80267669	+	2658	-1.305	-1
1500002 C15Rik	ENSMUSG00000073681	chr4	155107973	155108359	-	387	-1.306	-1
Strada	ENSMUSG00000069631	chr11	106024644	106063482	-	5581	-1.306	-1
2010111 I01Rik	ENSMUSG00000021458	chr13	63066253	63427404	+	7403	-1.307	-1
Rpl22	ENSMUSG00000028936	chr4	151699851	151708180	+	2911	-1.308	-1
Swi5	ENSMUSG00000044627	chr2	32134336	32143595	-	2234	-1.309	-1
Zfp553	ENSMUSG00000045598	chr7	134376575	134381693	+	3373	-1.309	-1
Rbm38	ENSMUSG00000027510	chr2	172845999	172860235	+	2869	-1.31	-1
Lyrm1	ENSMUSG00000030922	chr7	127039375	127060270	+	1513	-1.313	-1
Pla2g6	ENSMUSG00000042632	chr15	79116658	79158820	-	4980	-1.313	-1
Haus4	ENSMUSG00000022177	chr14	55160622	55173198	-	1587	-1.315	-1
Spcs1	ENSMUSG00000021917	chr14	31813012	31814858	-	1546	-1.316	-1
Tgfbr3	ENSMUSG00000029287	chr5	107535589	107718648	-	6545	-1.317	-1
Hoxb9	ENSMUSG00000020875	chr11	96132771	96137909	+	2574	-1.317	-1
Col23a1	ENSMUSG00000063564	chr11	51103422	51397420	+	5736	-1.317	-1
Raver1	ENSMUSG00000010205	chr9	20878612	20896443	-	3574	-1.318	-1
Tyk2	ENSMUSG00000032175	chr9	20908521	20931790	-	4611	-1.318	-1
Ap1b1	ENSMUSG00000009090	chr11	4886827	4942732	+	4904	-1.319	-1
2010003 O02Rik	ENSMUSG00000028407	chr4	40216602	40217973	+	1054	-1.319	-1
Clta	ENSMUSG00000028478	chr4	44017324	44045718	+	1800	-1.319	-1
Cacng7	ENSMUSG00000069806	chr7	3333055	3367810	+	2212	-1.319	-1
Gm5590	ENSMUSG00000046372	chr7	46701200	46701544	-	345	-1.319	-1
Tspo	ENSMUSG00000041736	chr15	83394003	83404631	+	854	-1.32	-1
AI31680 7	ENSMUSG00000031534	chr8	23573087	23587351	-	1458	-1.321	-1

Notum	ENSMUSG00000042988	chr11	120515102	120522489	-	2408	-1.321	-1
Tbc1d9	ENSMUSG00000031709	chr8	85689251	85796838	+	5892	-1.322	-1
Tinagl1	ENSMUSG00000028776	chr4	129841698	129852366	-	4360	-1.322	-1
Cog4	ENSMUSG00000031753	chr8	113370500	113406127	+	4053	-1.322	-1
Nomo1	ENSMUSG00000030835	chr7	53289066	53339582	+	4259	-1.323	-1
Adipor1	ENSMUSG00000026457	chr1	136311955	136329928	+	5025	-1.325	-1
Ogfr	ENSMUSG00000049401	chr2	180323950	180330541	+	2580	-1.325	-1
Ntn1	ENSMUSG00000020902	chr11	68022866	68214325	-	6277	-1.326	-1
Pes1	ENSMUSG00000020430	chr11	3863978	3880007	+	3024	-1.326	-1
Slc1a4	ENSMUSG00000020142	chr11	20202183	20232716	-	3926	-1.328	-1
Cd248	ENSMUSG00000056481	chr19	5068078	5070637	+	2560	-1.329	-1
Relb	ENSMUSG00000002983	chr7	20191566	20214787	-	3892	-1.329	-1
Fancc	ENSMUSG00000021461	chr13	63406017	63598586	-	7884	-1.329	-1
1810030N24Rik	ENSMUSG00000028295	chr4	34715913	34725672	-	1510	-1.329	-1
Nfkbia	ENSMUSG00000021025	chr12	56590398	56593634	-	1577	-1.329	-1
Trim3	ENSMUSG00000036989	chr7	112752977	112782085	-	4569	-1.33	-1
C030044B11Rik	ENSMUSG00000086252	chr13	49063656	49067400	+	1780	-1.331	-1
C730034F03Rik	ENSMUSG00000079242	chr15	89260020	89261299	+	1280	-1.331	-1
Nsmce4a	ENSMUSG00000040331	chr7	137676037	137716632	-	2597	-1.332	-1
Mcfd2	ENSMUSG00000024150	chr17	87653998	87665275	-	2612	-1.332	-1
Baiap2	ENSMUSG00000025372	chr11	119804077	119868096	+	4992	-1.332	-1
Myst1	ENSMUSG00000030801	chr7	135056031	135069346	+	1573	-1.334	-1
Tmem104	ENSMUSG00000045980	chr11	115048801	115108337	+	4610	-1.335	-1
Slc35c1	ENSMUSG00000049922	chr2	92292921	92300695	-	3275	-1.335	-1
Zfp498	ENSMUSG00000070420	chr5	146044212	146052338	+	1996	-1.338	-1
Sh3bgrl3	ENSMUSG00000028843	chr4	133683321	133684704	-	821	-1.339	-1
Agap3	ENSMUSG00000023353	chr5	23957995	24007865	+	3270	-1.34	-1
Fas	ENSMUSG00000024778	chr19	34365149	34402260	+	1801	-1.343	-1
Klc2	ENSMUSG00000024862	chr19	5107746	5118560	-	4134	-1.343	-1
Gna11	ENSMUSG00000034781	chr10	80991469	81007935	-	4097	-1.345	-1
Srcap	ENSMUSG00000090663	chr7	134702900	134708780	+	3054	-1.346	-1
Rasl12	ENSMUSG00000041696	chr9	65246295	65262660	+	3877	-1.347	-1
Gba2	ENSMUSG00000028467	chr4	43579800	43591745	-	4481	-1.348	-1
Urgcp	ENSMUSG00000049680	chr11	5613420	5662379	-	5871	-1.35	-1
Gpi1	ENSMUSG00000036427	chr7	34987141	35015308	-	4497	-1.35	-1
Scamp3	ENSMUSG00000028049	chr3	88981395	88986687	+	2270	-1.352	-1
Lyplal1	ENSMUSG00000039246	chr1	187911610	187941189	-	1578	-1.354	-1
Pim3	ENSMUSG00000035828	chr15	88692616	88696150	+	2421	-1.354	-1

Fam165b	ENSMUSG00000051989	chr16	92301531	92313286	+	972	-1.354	-1
Aprt	ENSMUSG00000006589	chr8	125098537	125100807	-	866	-1.357	-1
Cox4i2	ENSMUSG00000009876	chr2	152579909	152590773	+	788	-1.357	-1
Fam20c	ENSMUSG00000025854	chr5	139230468	139286031	+	4223	-1.358	-1
Nudt5	ENSMUSG00000025817	chr2	5766065	5792941	+	6379	-1.358	-1
Zfp827	ENSMUSG00000071064	chr8	81552336	81717665	+	8944	-1.358	-1
Rps6	ENSMUSG00000028495	chr4	86500564	86503316	-	1586	-1.359	-1
Pqlc2	ENSMUSG00000028744	chr4	138849944	138866623	-	3516	-1.359	-1
Hmgxb3	ENSMUSG00000024622	chr18	61290933	61336704	-	5197	-1.359	-1
Kti12	ENSMUSG00000073775	chr4	108520462	108522017	+	1556	-1.36	-1
Mterfd2	ENSMUSG00000026273	chr1	95195788	95202492	-	5271	-1.362	-1
Cpsf4	ENSMUSG00000029625	chr5	145928082	145942910	+	3598	-1.362	-1
1110001A16Rik	ENSMUSG00000062691	chr17	79315839	79320377	+	1502	-1.362	-1
Pgd	ENSMUSG00000028961	chr4	148524100	148540880	-	6447	-1.363	-1
Pank1	ENSMUSG00000033610	chr19	34885384	34953945	-	3717	-1.364	-1
Gm8619	ENSMUSG00000086889	chr13	48966481	48970829	+	498	-1.364	-1
Igkv6-17	ENSMUSG00000076590	chr6	70321463	70321987	+	347	-1.364	-1
Gm10943	ENSMUSG00000078448	chr10	52084919	52085670	-	60	-1.366	-1
Srebf2	ENSMUSG00000022463	chr15	81977696	82035806	+	4989	-1.368	-1
Zkscan14	ENSMUSG00000029627	chr5	145955815	145962751	-	2125	-1.368	-1
Atp5a1	ENSMUSG00000025428	chr18	78012468	78021608	+	3135	-1.369	-1
Slc39a8	ENSMUSG00000053897	chr3	135488243	135551536	+	7664	-1.37	-1
Cd37	ENSMUSG00000030798	chr7	52489002	52494485	-	3538	-1.37	-1
Prpf31	ENSMUSG00000008373	chr7	3581587	3594088	+	5439	-1.371	-1
Mr1	ENSMUSG00000026471	chr1	156975008	156993910	-	2509	-1.371	-1
RP23-381B19.7	ENSMUSG00000093483	chr11	83105201	83108134	+	711	-1.371	-1
Agrn	ENSMUSG00000041936	chr4	155539399	155571536	-	7957	-1.373	-1
Ripply3	ENSMUSG00000022941	chr16	94550027	94558542	+	1531	-1.374	-1
Ubiad1	ENSMUSG00000047719	chr4	147808604	147818880	-	2992	-1.375	-1
4931414P19Rik	ENSMUSG00000022179	chr14	55202500	55224745	-	2585	-1.375	-1
B2m	ENSMUSG00000060802	chr2	121973422	121978819	+	860	-1.376	-1
Htatip2	ENSMUSG00000039745	chr7	57014476	57029369	+	1465	-1.376	-1
Nop2	ENSMUSG00000038279	chr6	125081901	125094771	+	2623	-1.376	-1
Gm1840	ENSMUSG00000043192	chr8	5639584	5640996	+	1413	-1.377	-1
Wrap53	ENSMUSG00000041346	chr11	69375260	69393757	-	2062	-1.377	-1

Dync2li1	ENSMUSG00000024253	chr17	85025836	85054898	+	1324	-1.378	-1
Mgrn1	ENSMUSG00000022517	chr16	4886252	4938296	+	3204	-1.378	-1
Hist1h1e	ENSMUSG00000051627	chr13	23713624	23714371	-	748	-1.378	-1
Phldb1	ENSMUSG00000048537	chr9	44494387	44543281	-	7179	-1.383	-1
Dgka	ENSMUSG00000025357	chr10	128157197	128181112	-	2808	-1.384	-1
Itgb1bp1	ENSMUSG00000062352	chr12	21246686	21292098	-	3321	-1.386	-1
Ddx39	ENSMUSG00000005481	chr8	86239076	86250791	+	5155	-1.388	-1
Psma5	ENSMUSG00000068749	chr3	108059844	108082892	+	1321	-1.389	-1
Bpgm	ENSMUSG00000038871	chr6	34426207	34455613	+	3588	-1.391	-1
Limk2	ENSMUSG00000020451	chr11	3244259	3309192	-	6099	-1.391	-1
Gins2	ENSMUSG00000031821	chr8	123102533	123113204	-	4097	-1.391	-1
D2hgdh	ENSMUSG00000073609	chr1	95721817	95748925	+	4138	-1.392	-1
Adipor2	ENSMUSG00000030168	chr6	119303168	119367501	-	4168	-1.393	-1
Gm4893	ENSMUSG00000070308	chr9	39972215	39972544	+	330	-1.393	-1
Apobec3	ENSMUSG00000009585	chr15	79722089	79746336	+	6083	-1.395	-1
Rnf185	ENSMUSG00000020448	chr11	3315363	3352366	-	3702	-1.395	-1
2310001A20Rik	ENSMUSG00000033096	chr2	150408816	150434303	-	2428	-1.396	-1
Alkbh3	ENSMUSG00000040174	chr2	93820707	93850964	-	3159	-1.396	-1
Rarres2	ENSMUSG00000009281	chr6	48519697	48522669	-	708	-1.397	-1
Vat1	ENSMUSG00000034993	chr11	101320059	101327544	-	2766	-1.397	-1
Arl16	ENSMUSG00000057594	chr11	120326180	120328921	-	2742	-1.398	-1
Unc93b1	ENSMUSG00000036908	chr19	3935186	3949340	+	2841	-1.4	-1
Zdhhc16	ENSMUSG00000025157	chr19	42007962	42018585	+	1830	-1.4	-1
Mipep	ENSMUSG00000021993	chr14	61403403	61524315	+	3579	-1.4	-1
Gm129	ENSMUSG00000038550	chr3	95682426	95686174	-	2261	-1.4	-1
5930416I19Rik	ENSMUSG00000048668	chr6	128307018	128312929	-	2486	-1.401	-1
Ap3m2	ENSMUSG00000031539	chr8	23897826	23916126	-	3467	-1.402	-1
Sympk	ENSMUSG00000023118	chr7	19609726	19639967	+	7777	-1.402	-1
Rad23a	ENSMUSG00000003813	chr8	87357918	87364564	-	3946	-1.403	-1
4930455C21Rik	ENSMUSG00000002846	chr16	38498429	38522747	-	1784	-1.404	-1
Nubp1	ENSMUSG00000022503	chr16	10412041	10424521	+	3637	-1.405	-1
Myl12b	ENSMUSG00000034868	chr17	71323260	71340127	-	1711	-1.407	-1
Ftsj1	ENSMUSG00000031171	chrX	7815794	7829532	-	4760	-1.407	-1
Usf2	ENSMUSG00000058239	chr7	31730271	31741822	-	2647	-1.408	-1
Alyref2	ENSMUSG00000060244	chr1	173433609	173434881	+	1273	-1.41	-1
Ndrg2	ENSMUSG00000004558	chr14	52524946	52533163	-	2157	-1.41	-1
Zcchc3	ENSMUSG00000074682	chr2	152237692	152240780	-	3089	-1.41	-1

Psat1	ENSMUSG00000024640	chr19	15979168	16021827	-	4254	-1.411		-1
Aacs	ENSMUSG00000029482	chr5	125956184	125997782	+	3668	-1.411		-1
Trim2	ENSMUSG00000027993	chr3	83964361	84109368	-	16677	-1.413		-1
Rnf126	ENSMUSG00000035890	chr10	79221260	79229666	-	1608	-1.413		-1
Cltb	ENSMUSG00000047547	chr13	54693762	54712705	-	4868	-1.413		-1
Cdk4	ENSMUSG00000006728	chr10	126500590	126504976	+	3249	-1.414		-1
Gm10800	ENSMUSG00000075014	chr2	98506704	98507458	-	660	-1.414		-1
Tbc1d13	ENSMUSG00000039678	chr2	29989266	30007533	+	4719	-1.414		-1
1810009A15Rik	ENSMUSG00000071653	chr19	8963393	8965230	+	615	-1.416		-1
Rpa1	ENSMUSG00000000751	chr11	75111668	75161826	-	5578	-1.417		-1
Hebp2	ENSMUSG00000019853	chr10	18259929	18265882	-	1910	-1.417		-1
Arv1	ENSMUSG00000031982	chr8	127246039	127258023	+	1165	-1.418		-1
2410004N09Rik	ENSMUSG00000087590	chr18	33954575	33955640	+	401	-1.42		-1
Ecd	ENSMUSG00000021810	chr14	21139081	21167343	-	3125	-1.42		-1
Iqcc	ENSMUSG00000040795	chr4	129292370	129296343	-	3188	-1.421		-1
Ldb1	ENSMUSG00000025223	chr19	46107083	46119704	-	3583	-1.422		-1
Dcaf15	ENSMUSG00000037103	chr8	86620971	86628661	-	2312	-1.423		-1
Ap2m1	ENSMUSG00000022841	chr16	20535551	20544982	+	4172	-1.423		-1
Phf5a	ENSMUSG00000061360	chr15	81695749	81702341	-	937	-1.424		-1
Gm8394	ENSMUSG00000050490	chr10	84776220	84777184	+	965	-1.425		-1
Lgi3	ENSMUSG00000033595	chr14	70930628	70938130	+	3180	-1.426		-1
Mtif3	ENSMUSG00000016510	chr5	147763149	147775376	-	5127	-1.427		-1
Prmt5	ENSMUSG00000023110	chr14	55126024	55136362	-	3962	-1.427		-1
Tex261	ENSMUSG00000014748	chr6	83718875	83725806	-	4091	-1.428		-1
Prr13	ENSMUSG00000023048	chr15	102289459	102293237	+	1562	-1.428		-1
Anks1	ENSMUSG00000024219	chr17	28046285	28199545	+	7143	-1.43		-1
Ppp5c	ENSMUSG00000003099	chr7	17589989	17613273	-	3136	-1.432		-1
Prkrip1	ENSMUSG00000039737	chr5	136653997	136674833	-	3967	-1.432		-1
Casp7	ENSMUSG00000025076	chr19	56471619	56516834	+	2350	-1.434		-1
A930039A15Rik	ENSMUSG00000078493	chr1	155752431	155764040	+	459	-1.436		-1
Kank3	ENSMUSG00000042099	chr17	33947465	33959863	+	3119	-1.438		-1
Mllt1	ENSMUSG00000024212	chr17	57032035	57074836	-	3633	-1.438		-1
Ppp1r13b	ENSMUSG00000021285	chr12	113066668	113110826	-	3846	-1.438		-1
Maged1	ENSMUSG00000025151	chrX	91780813	91787482	-	4351	-1.439		-1
Ptpmt1	ENSMUSG00000063235	chr2	90750870	90758415	-	1687	-1.44		-1
Col8a2	ENSMUSG00000056174	chr4	125964037	125991574	+	4489	-1.441		-1
Patz1	ENSMUSG00000020453	chr11	3188877	3209086	+	5904	-1.442		-1
Ppp6r1	ENSMUSG00000052296	chr7	4583103	4610552	-	3787	-1.443		-1
Ccdc71	ENSMUSG00000049305	chr9	108362849	108368276	+	3108	-1.444		-1

Gm16515	ENSMUSG00000018931	chr11	60715748	60728252	-	4247	-1.444	-1
Zfp511	ENSMUSG00000025470	chr7	147222290	147226504	+	1100	-1.445	-1
Aldoart1	ENSMUSG00000059343	chr4	72511755	72513668	-	1914	-1.445	-1
Timm8b	ENSMUSG00000039016	chr9	50412006	50413425	+	527	-1.445	-1
Supt5h	ENSMUSG00000003435	chr7	29099917	29123738	-	3509	-1.446	-1
Gm8759	ENSMUSG00000067870	chr15	90594677	90595092	-	416	-1.446	-1
Hdac3	ENSMUSG00000024454	chr18	38095498	38114642	-	4026	-1.448	-1
Trap1	ENSMUSG00000005981	chr16	4039971	4078410	-	2584	-1.449	-1
Cuedc1	ENSMUSG00000018378	chr11	87912648	88007642	+	4472	-1.451	-1
Sirt6	ENSMUSG00000034748	chr10	81084530	81090542	-	2785	-1.451	-1
Cep78	ENSMUSG00000041491	chr19	16030264	16059479	-	2548	-1.451	-1
Rab8a	ENSMUSG00000003037	chr8	74685099	74707803	+	4678	-1.453	-1
Mrps17	ENSMUSG00000034211	chr5	130221372	130224750	+	2330	-1.454	-1
Wnt2	ENSMUSG00000010797	chr6	17938940	17980585	-	2255	-1.455	-1
Imp4	ENSMUSG00000026127	chr1	34496696	34502581	+	2504	-1.455	-1
Snx15	ENSMUSG00000024787	chr19	6119399	6128304	-	2184	-1.455	-1
Rnf123	ENSMUSG00000041528	chr9	107953865	107985677	-	6754	-1.456	-1
Cstf1	ENSMUSG00000027498	chr2	172196181	172207948	+	3504	-1.457	-1
Brsk1	ENSMUSG00000035390	chr7	4642206	4667599	+	4098	-1.457	-1
Arf1	ENSMUSG00000048076	chr11	59024914	59041772	-	2089	-1.457	-1
Larp6	ENSMUSG00000034839	chr9	60560796	60586608	+	2440	-1.458	-1
Klhl22	ENSMUSG00000022750	chr16	17759711	17793475	+	5745	-1.458	-1
Psmc5	ENSMUSG00000020708	chr11	106117468	106124434	+	1394	-1.458	-1
Zfp777	ENSMUSG00000071477	chr6	47974187	47998910	-	3588	-1.459	-1
Mblac1	ENSMUSG00000049285	chr5	138635542	138636628	+	1087	-1.459	-1
Ppp1r12b	ENSMUSG00000073557	chr1	136651235	136852519	-	13853	-1.461	-1
2610019E17Rik	ENSMUSG00000093565	chr17	24665193	24665689	+	497	-1.461	-1
Itga2b	ENSMUSG00000034664	chr11	102314611	102331436	-	5454	-1.461	-1
Bola3	ENSMUSG00000045160	chr6	83299141	83310130	+	3211	-1.461	-1
Adrbk1	ENSMUSG00000024858	chr19	4286001	4306222	-	4476	-1.461	-1
Uqcrfs1	ENSMUSG00000038462	chr13	30632177	30637231	-	1363	-1.462	-1
Grina	ENSMUSG00000022564	chr15	76077237	76080334	+	1693	-1.462	-1
Txndc12	ENSMUSG00000028567	chr4	108507206	108534732	+	2253	-1.462	-1
Bcl7b	ENSMUSG00000029681	chr5	135644153	135657725	+	2404	-1.463	-1
Wfs1	ENSMUSG00000039474	chr5	37357343	37380221	-	3564	-1.463	-1
Elovl1	ENSMUSG00000006390	chr4	118100698	118105558	+	2726	-1.463	-1
1110032A03Rik	ENSMUSG00000037971	chr9	50570933	50583625	-	5293	-1.463	-1
Trim41	ENSMUSG00000040365	chr11	48619906	48630855	-	3511	-1.463	-1
Sft2d3	ENSMUSG00000044982	chr18	32068748	32071557	-	2810	-1.464	-1
Gpx3	ENSMUSG00000018339	chr11	54715955	54723879	+	1813	-1.465	-1

Rom1	ENSMUSG00000071648	chr19	9001872	9003846	-	1526	-1.466	-1
Atp8a1	ENSMUSG00000037685	chr5	68009379	68238671	-	10962	-1.467	-1
Ubc	ENSMUSG00000008348	chr5	125866335	125870572	-	2893	-1.467	-1
Mgll	ENSMUSG00000033174	chr6	88674406	88778354	+	4868	-1.467	-1
Zfp704	ENSMUSG00000040209	chr3	9427011	9610085	-	13843	-1.468	-1
Nprl3	ENSMUSG00000020289	chr11	32125628	32167707	-	7572	-1.468	-1
Sh3yl1	ENSMUSG00000020669	chr12	31596533	31645027	+	2555	-1.468	-1
Rbm47	ENSMUSG00000070780	chr5	66407788	66564356	-	6859	-1.469	-1
Ldlrad3	ENSMUSG00000048058	chr2	101790360	102026542	-	6072	-1.469	-1
Bap1	ENSMUSG00000021901	chr14	32064675	32073115	+	3406	-1.469	-1
Kctd1	ENSMUSG00000036225	chr18	15127194	15309955	-	3616	-1.47	-1
E4f1	ENSMUSG00000024137	chr17	24580737	24592256	-	2561	-1.471	-1
Hps1	ENSMUSG00000025188	chr19	42829595	42854468	-	5855	-1.471	-1
Gpr180	ENSMUSG00000022131	chr14	118536349	118563454	+	2877	-1.471	-1
Rps17	ENSMUSG00000061787	chr7	88487618	88490140	-	1274	-1.473	-1
Mthfr	ENSMUSG00000029009	chr4	147413186	147433660	+	7315	-1.474	-1
Zfp574	ENSMUSG00000045252	chr7	25862265	25868511	+	4195	-1.475	-1
mt-Nd6	ENSMUSG00000064368	chrM	13552	14070	-	519	-1.477	-1
Tpd52l2	ENSMUSG00000000827	chr2	181231847	181252671	+	4982	-1.478	-1
Rela	ENSMUSG00000024927	chr19	5637483	5648130	+	2716	-1.478	-1
Aqp1	ENSMUSG00000004655	chr6	55286426	55298549	+	2627	-1.479	-1
Maz	ENSMUSG00000030678	chr7	134165657	134170533	-	2664	-1.479	-1
Tmem120a	ENSMUSG00000039886	chr5	136211355	136220141	-	1553	-1.48	-1
Rpl7a	ENSMUSG00000062647	chr2	26766284	26768838	+	1781	-1.48	-1
Arl2bp	ENSMUSG00000031776	chr8	97190550	97198327	+	2020	-1.481	-1
Pomt2	ENSMUSG00000034126	chr12	88447816	88488852	-	5059	-1.481	-1
Gtf2h3	ENSMUSG00000029387	chr5	125029149	125047689	+	3840	-1.482	-1
Ankrd23	ENSMUSG00000067653	chr1	36587379	36592584	-	1964	-1.482	-1
Faf1	ENSMUSG00000010517	chr4	109349193	109636565	+	5093	-1.484	-1
Rai12	ENSMUSG00000018565	chr11	69781724	69796019	-	2294	-1.484	-1
Mavs	ENSMUSG00000037523	chr2	131059799	131073761	+	3345	-1.485	-1
Zfp689	ENSMUSG00000048921	chr7	134587266	134592672	-	2005	-1.485	-1
Pdia5	ENSMUSG00000022844	chr16	35397399	35490959	-	1808	-1.486	-1
Fbl	ENSMUSG00000046865	chr7	28954765	28964288	+	1161	-1.487	-1
Isyna1	ENSMUSG00000019139	chr8	73118380	73121187	+	1836	-1.488	-1
2310015A10Rik	ENSMUSG00000085644	chr12	81221535	81233831	-	4843	-1.489	-1
Bcl2l12	ENSMUSG00000003190	chr7	52246592	52254082	-	1976	-1.493	-1
Podxl2	ENSMUSG00000033152	chr6	88792552	88825038	-	3948	-1.494	-1
Trim11	ENSMUSG00000020455	chr11	58791595	58804960	+	3580	-1.494	-1
Pigq	ENSMUSG00000025728	chr17	26063372	26081455	-	5769	-1.494	-1
Trpc2	ENSMUSG00000070425	chr7	109214025	109245378	+	8549	-1.494	-1
Prkaca	ENSMUSG00000005469	chr8	86496877	86520344	+	2315	-1.494	-1
Bbs2	ENSMUSG00000031755	chr8	96591854	96622711	-	3194	-1.494	-1

Rpusd4	ENSMUSG00000032044	chr9	35075450	35085316	+	2647	-1.495	-1
Nkain1	ENSMUSG00000078532	chr4	130125705	130169623	+	2552	-1.495	-1
Ppapdc 1b	ENSMUSG00000031570	chr8	26830533	26835359	+	2546	-1.495	-1
Fbxo9	ENSMUSG00000001366	chr9	77929306	77956872	-	3103	-1.495	-1
Dcakd	ENSMUSG00000020935	chr11	102855361	102889647	-	2556	-1.496	-1
Wdr45l	ENSMUSG00000025173	chr11	121188538	121215759	-	4562	-1.497	-1
Nmt1	ENSMUSG00000020936	chr11	102889504	102930226	+	5017	-1.498	-1
Mmp23	ENSMUSG00000029061	chr4	155024764	155027493	-	2340	-1.499	-1
Mdh1	ENSMUSG00000020321	chr11	21456790	21472370	-	2567	-1.499	-1
Rnf215	ENSMUSG00000003581	chr11	4035205	4041175	+	2232	-1.503	-1
Rnaset2 a	ENSMUSG00000073469	chr17	8321456	8340697	-	1013	-1.504	-1
Ccdc53	ENSMUSG00000020056	chr10	87663842	87708897	+	1371	-1.505	-1
B23011 8H07Rik	ENSMUSG00000027165	chr2	101400938	101489689	-	4289	-1.506	-1
Prr5	ENSMUSG00000036106	chr15	84500050	84534103	+	1917	-1.506	-1
Peo1	ENSMUSG00000025209	chr19	45080563	45087252	+	3603	-1.506	-1
Atg7	ENSMUSG00000030314	chr6	114593149	114810431	+	3651	-1.507	-1
Cx3cl1	ENSMUSG00000031778	chr8	97295909	97306323	+	3708	-1.507	-1
Smg5	ENSMUSG00000001415	chr3	88140182	88166259	+	4447	-1.508	-1
Rpl3	ENSMUSG00000060036	chr15	79908211	79913836	-	1361	-1.508	-1
Dtnbp1	ENSMUSG00000057531	chr13	45017454	45097509	-	1349	-1.509	-1
Tmem1 29	ENSMUSG00000019295	chr5	33995865	34000626	-	2740	-1.509	-1
Srp68	ENSMUSG00000020780	chr11	116106480	116135531	-	2737	-1.51	-1
Zfp324	ENSMUSG00000004500	chr7	13551187	13559585	+	4776	-1.511	-1
Abcd1	ENSMUSG00000031378	chrX	70961936	70983873	+	4192	-1.514	-1
Rbm14	ENSMUSG00000006456	chr19	4784299	4811634	-	5441	-1.514	-1
H6pd	ENSMUSG00000028980	chr4	149353584	149383132	-	5236	-1.514	-1
Tmem2 01	ENSMUSG00000044700	chr4	149089484	149112153	-	8944	-1.515	-1
Fabp5	ENSMUSG00000027533	chr3	10012548	10016607	+	3567	-1.515	-1
Mocs1	ENSMUSG00000064120	chr17	49567687	49594760	+	3110	-1.516	-1
Sidt2	ENSMUSG00000034908	chr9	45745940	45763330	-	5135	-1.518	-1
Nrbp2	ENSMUSG00000075590	chr15	75916025	75920443	-	2495	-1.518	-1
Ilkap	ENSMUSG00000026309	chr1	93271984	93295360	-	1500	-1.52	-1
Clybl	ENSMUSG00000025545	chr14	122580916	122801454	+	1243	-1.521	-1
Sfi1	ENSMUSG00000023764	chr11	3031853	3093466	-	7571	-1.521	-1
Mapk13	ENSMUSG00000004864	chr17	28906252	28915643	+	1512	-1.523	-1
Mcoln1	ENSMUSG00000004567	chr8	3500457	3515232	+	3521	-1.524	-1
Gtf3c5	ENSMUSG00000026816	chr2	28421831	28439271	-	4560	-1.525	-1
Rnf149	ENSMUSG00000048234	chr1	39608141	39634250	-	2838	-1.525	-1
Rpl7l1	ENSMUSG00000063888	chr17	46910856	46919605	-	2606	-1.525	-1
Smpd1	ENSMUSG00000037049	chr7	112702874	112706902	+	2411	-1.526	-1

Gt(ROSA)26Sor	ENSMUSG00000086429	chr6	113017422	113027327	-	1695	-1.526	-1
Zfp954	ENSMUSG00000062116	chr7	7067401	7074163	-	2006	-1.527	-1
Scrn1	ENSMUSG00000019124	chr6	54455884	54516483	-	8371	-1.529	-1
B3gntl1	ENSMUSG00000046605	chr11	121477511	121534467	-	3518	-1.529	-1
Myd88	ENSMUSG00000032508	chr9	119245052	119250529	-	2576	-1.53	-1
Suv420h2	ENSMUSG00000059851	chr7	4691717	4699116	+	4001	-1.53	-1
Txndc11	ENSMUSG00000022498	chr16	11075004	11134743	-	8835	-1.532	-1
Chtf18	ENSMUSG00000019214	chr17	25855871	25864364	-	4584	-1.532	-1
Phf7	ENSMUSG00000021902	chr14	32050882	32064404	-	1984	-1.532	-1
Tnpo2	ENSMUSG00000031691	chr8	87560814	87581482	+	4990	-1.533	-1
Fam54b	ENSMUSG00000046671	chr4	134081465	134091302	-	5370	-1.533	-1
Mrps36-ps1	ENSMUSG00000021631	chr17	54005742	54006366	-	625	-1.534	-1
Zswim3	ENSMUSG00000045822	chr2	164630598	164647630	+	2708	-1.534	-1
Ift140	ENSMUSG00000024169	chr17	25153036	25236440	+	7572	-1.534	-1
Ttc15	ENSMUSG00000020628	chr12	29375494	29435331	-	5439	-1.536	-1
Snrnp40	ENSMUSG00000074088	chr4	130037379	130067274	+	2790	-1.537	-1
Man1b1	ENSMUSG00000036646	chr2	25187858	25207732	+	5391	-1.537	-1
Ildr1	ENSMUSG00000022900	chr16	36694064	36726890	+	4296	-1.539	-1
Zbtb7b	ENSMUSG00000028042	chr3	89181566	89198698	-	5591	-1.539	-1
Cradd	ENSMUSG00000045867	chr10	94637380	94786767	-	2428	-1.54	-1
Gm12841	ENSMUSG00000085278	chr4	117556226	117558600	+	338	-1.541	-1
Ddx24	ENSMUSG00000041645	chr12	104646186	104664077	-	3157	-1.542	-1
Rbx1	ENSMUSG00000022400	chr15	81296746	81306799	+	1655	-1.542	-1
Wdsub1	ENSMUSG00000026988	chr2	59690421	59720648	-	3598	-1.542	-1
9130401M01Rik	ENSMUSG00000022362	chr15	57853831	57907980	-	1683	-1.543	-1
Obfc2b	ENSMUSG00000025374	chr10	127838451	127848548	-	2507	-1.543	-1
Csnk1g2	ENSMUSG00000003345	chr10	80085525	80103516	+	2511	-1.543	-1
2410131K14Rik	ENSMUSG00000032840	chr5	118695236	118716102	+	5649	-1.543	-1
Zmiz2	ENSMUSG00000041164	chr11	6289077	6306165	+	4659	-1.544	-1
Pctp	ENSMUSG00000020553	chr11	89843979	89864208	-	2723	-1.544	-1
Snhg12	ENSMUSG00000086290	chr4	131864593	131866939	+	1626	-1.545	-1
R3hcc1	ENSMUSG00000034194	chr14	70097112	70117552	-	2995	-1.545	-1
Atoh8	ENSMUSG00000037621	chr6	72156171	72185571	-	2355	-1.545	-1
Ccdc149	ENSMUSG00000045790	chr5	52765892	52766785	-	894	-1.545	-1
Kat5	ENSMUSG00000024926	chr19	5603017	5610050	-	2356	-1.548	-1

Mrto4	ENSMUSG00000028741	chr4	138903350	138908491	-	2611	-1.549	-1
Adra1d	ENSMUSG00000027335	chr2	131371586	131388021	-	1901	-1.549	-1
Comtd1	ENSMUSG00000021773	chr14	22665083	22668199	-	2464	-1.55	-1
Flot2	ENSMUSG00000061981	chr11	77851433	77873936	+	2824	-1.551	-1
Kctd2	ENSMUSG00000016940	chr11	115281440	115292588	+	3011	-1.552	-1
Oat	ENSMUSG00000030934	chr7	139749158	139768081	-	2155	-1.552	-1
Psmd4	ENSMUSG00000005625	chr3	94836616	94846536	-	2808	-1.552	-1
Gm4980	ENSMUSG00000060814	chr7	106774004	106774684	-	681	-1.552	-1
Pnpla2	ENSMUSG00000025509	chr7	148641097	148646642	+	3020	-1.553	-1
Cmtm4	ENSMUSG00000051578	chr8	106878416	106919596	-	1298	-1.553	-1
Agfg2	ENSMUSG00000029722	chr5	138091711	138125954	-	3802	-1.554	-1
Nrg2	ENSMUSG00000060275	chr18	36177333	36357034	-	3116	-1.555	-1
BC031353	ENSMUSG00000034858	chr9	74800691	74880275	+	6456	-1.555	-1
Zdhhc18	ENSMUSG00000037553	chr4	133161214	133206069	-	5382	-1.556	-1
Afg3l1	ENSMUSG00000031967	chr8	126001803	126027816	+	4757	-1.556	-1
Adamtsl5	ENSMUSG00000043822	chr10	79801855	79811157	-	4722	-1.557	-1
Ctnnal1	ENSMUSG00000038816	chr4	56823807	56878060	-	7661	-1.558	-1
Atf4	ENSMUSG00000042406	chr15	80085614	80087970	+	1724	-1.559	-1
Tnk2	ENSMUSG00000022791	chr16	32643960	32683579	+	5496	-1.56	-1
Cab39l	ENSMUSG00000021981	chr14	60059818	60167740	+	3099	-1.561	-1
Tut1	ENSMUSG00000071645	chr19	9028340	9040697	+	2747	-1.562	-1
Anapc11	ENSMUSG00000025135	chr11	120459735	120469512	+	5057	-1.564	-1
Hddc2	ENSMUSG00000000295	chr10	31033188	31048010	+	3509	-1.564	-1
Ap4s1	ENSMUSG00000020955	chr12	52791953	52839918	+	991	-1.564	-1
Ighg2c	ENSMUSG00000076612	chr12	114523536	114527141	-	1212	-1.564	-1
Ctdnep1	ENSMUSG00000018559	chr11	69794658	69804103	+	2182	-1.565	-1
Mmp28	ENSMUSG00000020682	chr11	83254270	83276573	-	3874	-1.565	-1
Calhm2	ENSMUSG00000033033	chr19	47179843	47212784	-	4361	-1.565	-1
Msh2	ENSMUSG00000024151	chr17	88071670	88123053	+	5518	-1.567	-1
Tbl2	ENSMUSG00000005374	chr5	135625527	135639743	+	5968	-1.567	-1
Lmo2	ENSMUSG00000032698	chr2	103798143	103822035	+	2166	-1.568	-1
Ptges	ENSMUSG00000050737	chr2	30744991	30785383	-	3933	-1.569	-1
St6galnac6	ENSMUSG00000026811	chr2	32455229	32476326	+	4815	-1.571	-1
Abat	ENSMUSG00000057880	chr16	8513522	8621661	+	7748	-1.571	-1
Snrnp27	ENSMUSG00000001158	chr6	86625215	86634516	-	1767	-1.572	-1
Ldha	ENSMUSG00000063229	chr7	54096845	54110997	+	2982	-1.572	-1
Dhx16	ENSMUSG00000024422	chr17	36016764	36029615	+	4094	-1.573	-1
Ctsa	ENSMUSG00000017760	chr2	164658373	164666532	+	4715	-1.573	-1
Fth1	ENSMUSG00000024661	chr19	10057193	10059582	+	866	-1.573	-1
Tbc1d9b	ENSMUSG00000036644	chr11	49944898	49986287	+	6429	-1.574	-1

Fkrp	ENSMUSG00000048920	chr7	17394619	17400478	-	2773	-1.574	-1
Strn4	ENSMUSG00000030374	chr7	17401238	17426280	+	7825	-1.575	-1
Agtrap	ENSMUSG00000029007	chr4	147451170	147462140	-	4236	-1.575	-1
Lztr1	ENSMUSG00000022761	chr16	17508781	17526426	+	5445	-1.576	-1
Tor3a	ENSMUSG00000060519	chr1	158583748	158604487	-	3892	-1.576	-1
Rnf121	ENSMUSG00000070426	chr7	109167651	109213983	-	4427	-1.577	-1
Fbxo7	ENSMUSG00000001786	chr10	85484717	85514618	+	6180	-1.578	-1
Tsen15	ENSMUSG00000014980	chr1	154217865	154233818	-	1939	-1.578	-1
2310022A10Rik	ENSMUSG00000049643	chr7	28338314	28367113	+	2710	-1.578	-1
Iah1	ENSMUSG00000062054	chr12	21322253	21329466	+	924	-1.578	-1
Ostc	ENSMUSG00000041084	chr3	130398839	130412362	-	1058	-1.579	-1
Itfg3	ENSMUSG00000024187	chr17	26348767	26381168	-	3981	-1.579	-1
Gla	ENSMUSG00000031266	chrX	131122688	131135664	-	3265	-1.58	-1
Tfip11	ENSMUSG00000029345	chr5	112755378	112767093	+	4086	-1.58	-1
Cklf	ENSMUSG00000054400	chr8	106774803	106788836	+	2134	-1.582	-1
Chchd4	ENSMUSG00000034203	chr6	91414270	91423417	-	1245	-1.582	-1
Cdc42ep1	ENSMUSG00000049521	chr15	78673077	78681332	+	2542	-1.582	-1
Alg9	ENSMUSG00000032059	chr9	50583124	50651647	+	3889	-1.582	-1
Crebl2	ENSMUSG00000032652	chr6	134780172	134808949	+	3923	-1.582	-1
Maged2	ENSMUSG00000025268	chrX	147240913	147248888	-	3239	-1.583	-1
Rnf40	ENSMUSG00000030816	chr7	134732212	134747119	+	4154	-1.583	-1
BC022687	ENSMUSG00000037594	chr12	114047186	114054456	+	1915	-1.584	-1
Mgat4b	ENSMUSG00000036620	chr11	50024392	50048605	+	2719	-1.587	-1
2310079F23Rik	ENSMUSG00000029097	chr5	35899855	35917719	-	4151	-1.587	-1
Tomm22	ENSMUSG00000022427	chr15	79501291	79503292	+	1113	-1.588	-1
Ap1s1	ENSMUSG00000004849	chr5	137510863	137522005	-	2198	-1.589	-1
Fam59a	ENSMUSG00000042680	chr18	21285702	21458639	-	4923	-1.59	-1
Arpc1a	ENSMUSG00000029621	chr5	145844699	145869630	+	2567	-1.592	-1
Deptor	ENSMUSG00000022419	chr15	54943872	55090826	+	8874	-1.594	-1
Rasl11b	ENSMUSG00000049907	chr5	74591321	74595503	+	1799	-1.595	-1
Fzd2	ENSMUSG00000050288	chr11	102465745	102469372	+	3628	-1.595	-1
Prkag1	ENSMUSG00000067713	chr15	98643229	98661939	-	1647	-1.595	-1
Cdkn1c	ENSMUSG00000037664	chr7	150644255	150646955	-	1931	-1.596	-1
Gm9774	ENSMUSG00000042165	chr3	92232092	92233315	-	933	-1.597	-1
Atpaf2	ENSMUSG00000042709	chr11	60214128	60231959	-	2185	-1.598	-1
Darc	ENSMUSG00000037872	chr1	175262017	175263634	-	1158	-1.599	-1
Lyl1	ENSMUSG00000034041	chr8	87225356	87228614	+	1800	-1.6	-1
Dak	ENSMUSG00000034371	chr19	10662281	10679922	-	3348	-1.6	-1
Itm2b	ENSMUSG00000022108	chr14	73762038	73785078	-	1773	-1.601	-1

Gtf3a	ENSMUSG00000016503	chr5	147760233	147767190	+	2548	-1.601	-1
Dgkz	ENSMUSG00000040479	chr2	91772981	91816021	-	6270	-1.602	-1
Atrip	ENSMUSG00000025646	chr9	108960447	108976638	-	5086	-1.602	-1
Dynll2	ENSMUSG00000020483	chr11	87793027	87801035	-	2734	-1.604	-1
Rps27l	ENSMUSG00000036781	chr9	66793893	66797323	+	816	-1.604	-1
Gtpbp2	ENSMUSG00000023952	chr17	46297981	46306319	+	5305	-1.606	-1
Cyb5b	ENSMUSG00000031924	chr8	109674561	109711370	+	4294	-1.606	-1
Katnb1	ENSMUSG00000031787	chr8	97605101	97623774	+	3425	-1.606	-1
D11Wsu99e	ENSMUSG00000041629	chr11	113522633	113545469	-	2893	-1.608	-1
Akap8l	ENSMUSG00000002625	chr17	32458370	32487540	-	2089	-1.608	-1
Lrrc42	ENSMUSG00000028617	chr4	106906119	106926137	-	2139	-1.609	-1
Gm11458	ENSMUSG00000085436	chr2	164736854	164745453	+	1273	-1.61	-1
1810026J23Rik	ENSMUSG00000048429	chr9	21397166	21400390	+	3146	-1.611	-1
Tprn	ENSMUSG00000048707	chr2	25118118	25125406	+	2953	-1.611	-1
St6galnac2	ENSMUSG00000057286	chr11	116536617	116556182	-	7090	-1.612	-1
Ap2a1	ENSMUSG00000060279	chr7	52155743	52184866	-	4301	-1.612	-1
Smyd5	ENSMUSG00000033706	chr6	85381983	85396429	+	3169	-1.612	-1
Hmg20b	ENSMUSG00000020232	chr10	80808793	80813225	-	3149	-1.613	-1
Irf1	ENSMUSG00000018899	chr11	53583516	53591876	+	3976	-1.613	-1
Fads2	ENSMUSG00000024665	chr19	10138654	10175993	-	1508	-1.614	-1
Cd99l2	ENSMUSG00000035776	chrX	68673235	68746024	-	4902	-1.616	-1
Eml3	ENSMUSG00000071647	chr19	9004184	9016053	+	3161	-1.617	-1
E130012A19Rik	ENSMUSG00000043439	chr11	97488707	97491030	-	2324	-1.618	-1
Ppp1r3c	ENSMUSG00000067279	chr19	36806227	36811143	-	2719	-1.619	-1
Supt3h	ENSMUSG00000038954	chr17	44914101	45256239	+	4121	-1.619	-1
Chkb	ENSMUSG00000022617	chr15	89246836	89260339	-	5805	-1.62	-1
Trappc4	ENSMUSG00000032112	chr9	44211844	44215631	-	1401	-1.622	-1
Slc44a2	ENSMUSG00000057193	chr9	21142288	21159469	+	3440	-1.622	-1
Gps2	ENSMUSG00000023170	chr11	69727390	69730093	+	1604	-1.625	-1
Tmem85	ENSMUSG00000027131	chr2	112203168	112208184	-	1052	-1.625	-1
Itpkc	ENSMUSG00000003752	chr7	27992191	28013647	-	3644	-1.625	-1
Wibg	ENSMUSG00000064030	chr10	128184935	128203617	+	1211	-1.626	-1
Tef	ENSMUSG00000022389	chr15	81632851	81657291	+	7865	-1.626	-1
Mdp1	ENSMUSG00000002329	chr14	56276716	56279345	-	1733	-1.626	-1
Arhgef18	ENSMUSG00000004568	chr8	3393006	3456601	+	8395	-1.627	-1
Uqcrc2	ENSMUSG00000030884	chr7	127778690	127802992	+	2506	-1.628	-1
Gm5145	ENSMUSG00000071273	chr17	20707326	20708162	+	837	-1.629	-1

2410066E13Rik	ENSMUSG00000038065	chr6	54631734	54653845	+	5187	-1.63	-1
Nod1	ENSMUSG00000038058	chr6	54873936	54922606	-	4415	-1.63	-1
Tbc1d10b	ENSMUSG00000042492	chr7	134340973	134351982	-	4696	-1.63	-1
Srf	ENSMUSG00000015605	chr17	46683788	46693111	-	4093	-1.631	-1
Pigy	ENSMUSG00000043162	chr6	57636570	57642072	-	3573	-1.632	-1
Slc7a8	ENSMUSG00000022180	chr14	55341053	55400723	-	4033	-1.632	-1
Ubl7	ENSMUSG00000055720	chr9	57758793	57777775	+	1364	-1.633	-1
2310067B10Rik	ENSMUSG00000020747	chr11	115626747	115660680	+	5920	-1.636	-1
Phb	ENSMUSG00000038845	chr11	95528271	95542087	+	2805	-1.636	-1
Stk11	ENSMUSG00000003068	chr10	79578548	79593427	+	4580	-1.636	-1
Slc39a1	ENSMUSG00000052310	chr3	90052094	90057534	+	2364	-1.637	-1
Zfp335	ENSMUSG00000039834	chr2	164717382	164737257	-	4743	-1.637	-1
Repin1	ENSMUSG00000052751	chr6	48543882	48549081	+	3975	-1.639	-1
Gfra4	ENSMUSG00000027316	chr2	130865368	130868824	-	2165	-1.639	-1
Eif3h	ENSMUSG00000022312	chr15	51618104	51697027	-	1253	-1.639	-1
1700088E04Rik	ENSMUSG00000033029	chr15	78965085	78971681	-	1415	-1.64	-1
Ccdc77	ENSMUSG00000030177	chr6	120274341	120314392	-	6685	-1.641	-1
Pik3ap1	ENSMUSG00000025017	chr19	41346863	41459586	-	4486	-1.641	-1
Dcaf8	ENSMUSG00000026554	chr1	174078146	174126524	+	3913	-1.641	-1
Fh1	ENSMUSG00000026526	chr1	177530505	177555766	-	3392	-1.642	-1
Kars	ENSMUSG00000031948	chr8	114517340	114535223	-	2249	-1.643	-1
Nradd	ENSMUSG00000032491	chr9	110523641	110526897	-	1162	-1.643	-1
Polb	ENSMUSG00000031536	chr8	23738591	23763909	-	1227	-1.643	-1
Ap3s2	ENSMUSG00000063801	chr7	87020218	87065526	-	5797	-1.643	-1
Pank4	ENSMUSG00000029056	chr4	154338232	154355047	+	3487	-1.644	-1
Zkscan17	ENSMUSG00000020472	chr11	59299022	59340253	-	4573	-1.644	-1
Rps20	ENSMUSG00000028234	chr4	3758481	3762812	-	4080	-1.645	-1
Ccdc101	ENSMUSG00000030714	chr7	133792823	133816439	+	1802	-1.645	-1
Wdr55	ENSMUSG00000042660	chr18	36919893	36923362	+	1498	-1.646	-1
Ppt1	ENSMUSG00000028657	chr4	122513485	122536418	+	3675	-1.646	-1
4930444A02Rik	ENSMUSG00000037251	chr8	27091076	27104593	-	3601	-1.648	-1
Ldhd	ENSMUSG00000031958	chr8	114147685	114154274	-	5328	-1.648	-1
Txndc5	ENSMUSG00000038991	chr13	38591948	38620693	-	4492	-1.651	-1
Ndn	ENSMUSG00000033585	chr7	69493163	69494814	+	1652	-1.652	-1
Nme1	ENSMUSG00000037601	chr11	93818293	93829835	-	3342	-1.653	-1

2410091C18Rik	ENSMUSG00000024082	chr17	79336475	79347392	+	2303	-1.654	-1
Osbpl5	ENSMUSG00000037606	chr7	150874667	150942890	-	4915	-1.654	-1
Fbxo10	ENSMUSG00000048232	chr4	45047119	45097476	-	4691	-1.654	-1
Prr3	ENSMUSG00000038500	chr17	36109486	36117181	-	3783	-1.656	-1
Slc34a2	ENSMUSG00000029188	chr5	53429321	53462903	+	5011	-1.656	-1
Trim47	ENSMUSG00000020773	chr11	115967066	115988524	-	2735	-1.656	-1
Gtf2h5	ENSMUSG00000034345	chr17	6079786	6086517	+	3855	-1.657	-1
Tfap4	ENSMUSG00000005718	chr16	4544661	4559720	-	2113	-1.658	-1
Tbc1d30	ENSMUSG00000052302	chr10	120700880	120748245	-	5691	-1.658	-1
5730403B10Rik	ENSMUSG00000004071	chr16	4750348	4790292	-	5746	-1.658	-1
Adrm1	ENSMUSG00000039041	chr2	179906190	179910991	+	4457	-1.659	-1
Hook2	ENSMUSG00000052566	chr8	87514494	87527263	+	2617	-1.659	-1
Mettl3	ENSMUSG00000022160	chr14	52914343	52924803	-	3987	-1.661	-1
Cpt2	ENSMUSG00000028607	chr4	107576586	107596215	-	2391	-1.661	-1
Dvl2	ENSMUSG00000020888	chr11	69814097	69825803	+	6298	-1.664	-1
Sh2b1	ENSMUSG00000030733	chr7	133610508	133618644	-	3445	-1.665	-1
Dpp7	ENSMUSG00000026958	chr2	25207796	25211879	-	3337	-1.665	-1
Anapc16	ENSMUSG00000020107	chr10	59450657	59465860	-	1329	-1.665	-1
1810032O08Rik	ENSMUSG00000020812	chr11	116532974	116537113	+	2776	-1.665	-1
Trmu	ENSMUSG00000022386	chr15	85709742	85727824	+	3974	-1.666	-1
1300001I01Rik	ENSMUSG00000020741	chr11	74462997	74484349	+	6502	-1.666	-1
Anks3	ENSMUSG00000022515	chr16	4941420	4964330	-	2483	-1.667	-1
Echdc3	ENSMUSG00000039063	chr2	6109511	6134079	-	2685	-1.674	-1
1500012F01Rik	ENSMUSG00000074578	chr2	166888434	166891362	+	1901	-1.674	-1
Sec11a	ENSMUSG00000025724	chr7	88049775	88092666	-	4614	-1.674	-1
Esyt3	ENSMUSG00000037681	chr9	99210386	99258949	-	4491	-1.674	-1
Mgmt	ENSMUSG00000054612	chr7	144086294	144319870	+	818	-1.675	-1
Gm10687	ENSMUSG00000074408	chr9	43931851	43942568	-	1651	-1.675	-1
Gle1	ENSMUSG00000019715	chr2	29790946	29815891	+	6087	-1.677	-1
Rps26	ENSMUSG00000025362	chr10	128061586	128063562	-	447	-1.678	-1
Svs3a	ENSMUSG00000017003	chr2	164115004	164117236	+	1200	-1.679	-1
Habp4	ENSMUSG00000021476	chr13	64263174	64287844	+	2566	-1.679	-1
Lmbr1l	ENSMUSG00000022999	chr15	98734352	98748662	-	4217	-1.68	-1
Cbx4	ENSMUSG00000039989	chr11	118938887	118947535	-	5210	-1.681	-1
Spcs2	ENSMUSG00000035227	chr7	106987513	107007403	-	1391	-1.681	-1
Hint3	ENSMUSG00000019791	chr10	30327947	30338285	-	990	-1.683	-1

Ccdc120	ENSMUSG00000031150	chrX	7308840	7328031	-	4165	-1.683	-1
Tspan31	ENSMUSG00000006736	chr10	126504373	126507317	-	1516	-1.685	-1
Psmb2	ENSMUSG00000028837	chr4	126354874	126386958	+	1204	-1.685	-1
Rcn1	ENSMUSG00000005973	chr2	105226448	105239476	-	3196	-1.686	-1
Irf8	ENSMUSG00000041515	chr8	123260258	123280594	+	7055	-1.686	-1
Ifi30	ENSMUSG00000031838	chr8	73286673	73291050	-	993	-1.687	-1
Ndufa5	ENSMUSG00000023089	chr6	24468666	24478013	-	1032	-1.687	-1
Ube2l6	ENSMUSG00000027078	chr2	84638985	84650492	+	1996	-1.688	-1
Cpsf1	ENSMUSG00000034022	chr15	76426239	76438021	-	4515	-1.688	-1
Armc7	ENSMUSG00000057219	chr11	115336981	115351781	+	3069	-1.688	-1
1700037H04Rik	ENSMUSG00000027327	chr2	130972060	130985817	-	2286	-1.689	-1
Rmnd5b	ENSMUSG00000001054	chr11	51437173	51449398	-	2161	-1.689	-1
Inpp5k	ENSMUSG00000006127	chr11	75444490	75462373	+	5728	-1.689	-1
1810008A18Rik	ENSMUSG00000032977	chr10	76949401	76978558	-	2508	-1.689	-1
Apbb3	ENSMUSG00000001379	chr18	36830814	36839020	-	2061	-1.69	-1
Xylt2	ENSMUSG00000020868	chr11	94525165	94538829	-	3979	-1.691	-1
Nup85	ENSMUSG00000020739	chr11	115425748	115445299	+	3385	-1.691	-1
Muted	ENSMUSG00000038982	chr13	38684723	38726978	-	2477	-1.692	-1
Rps8	ENSMUSG00000047675	chr4	116826432	116828848	-	1726	-1.694	-1
2410002F23Rik	ENSMUSG00000045411	chr7	51502092	51518090	+	7547	-1.695	-1
Dgat1	ENSMUSG00000022555	chr15	76332445	76342381	-	3054	-1.695	-1
Flcn	ENSMUSG00000032633	chr11	59604910	59623518	-	4322	-1.698	-1
Ubac1	ENSMUSG00000036352	chr2	25854063	25877267	-	2632	-1.698	-1
Med16	ENSMUSG00000013833	chr10	79357452	79371683	-	4679	-1.699	-1
1810074P20Rik	ENSMUSG00000040359	chr4	25175747	25208968	-	5706	-1.7	-1
Spata13	ENSMUSG00000021990	chr14	61252838	61383393	+	9102	-1.701	-1
Gstp1	ENSMUSG00000060803	chr19	4035411	4037921	-	753	-1.707	-1
Atp6v0c	ENSMUSG00000024121	chr17	24300833	24306647	-	3384	-1.709	-1
Rpl12	ENSMUSG00000038900	chr2	32817079	32820016	+	2612	-1.709	-1
Aph1a	ENSMUSG00000015750	chr3	95697892	95702211	+	2863	-1.71	-1
Mrpl40	ENSMUSG00000022706	chr16	18872111	18876860	-	1214	-1.71	-1
Pex19	ENSMUSG00000003464	chr1	174056886	174066624	+	3815	-1.71	-1
Bcap31	ENSMUSG00000002015	chrX	70931517	70961514	-	1219	-1.712	-1
Trmt61b	ENSMUSG00000085492	chr17	71906367	71948095	-	2897	-1.713	-1
Cks1b	ENSMUSG00000028044	chr3	89219394	89222305	-	1126	-1.714	-1
Tbc1d10c	ENSMUSG00000040247	chr19	4184358	4191047	-	1758	-1.715	-1

Slc24a6	ENSMUSG00000032754	chr5	120961177	120984033	+	3463	-1.715		-1
Dusp19	ENSMUSG00000027001	chr2	80457202	80472518	+	2992	-1.715		-1
Phlda3	ENSMUSG00000041801	chr1	137662696	137665713	+	1559	-1.716		-1
Dctn2	ENSMUSG00000025410	chr10	126703442	126718862	+	1641	-1.717		-1
Rpl27	ENSMUSG00000063316	chr11	101303612	101306843	+	1535	-1.717		-1
Kazn	ENSMUSG00000040606	chr4	141658305	141795316	-	10721	-1.72		-1
Eif1ad	ENSMUSG00000024841	chr19	5366813	5371511	+	2469	-1.721		-1
Clptm1	ENSMUSG00000002981	chr7	20216930	20250379	-	4130	-1.722		-1
Cndp2	ENSMUSG00000024644	chr18	84836862	84855025	-	2119	-1.722		-1
Fam188b	ENSMUSG00000038022	chr6	55153377	55270216	+	4515	-1.722		-1
AA960436	ENSMUSG00000031792	chr8	97856192	97871407	+	2791	-1.723		-1
Wdr45	ENSMUSG00000039382	chrX	7299099	7305327	+	2717	-1.723		-1
Ppap2c	ENSMUSG00000052151	chr10	78989175	78996532	-	1645	-1.724		-1
Aldh6a1	ENSMUSG00000021238	chr12	85771673	85791900	-	3527	-1.724		-1
Tle1	ENSMUSG00000008305	chr4	71778176	71861953	-	8711	-1.724		-1
Hmgn2	ENSMUSG00000003038	chr4	133520653	133524565	-	3060	-1.728		-1
Gm6055	ENSMUSG00000062081	chr14	48698810	48699487	-	678	-1.728		-1
Hadh	ENSMUSG00000027984	chr3	130936337	130975019	-	4977	-1.729		-1
Lmf2	ENSMUSG00000022614	chr15	89181435	89186090	-	2906	-1.73		-1
Stx6	ENSMUSG00000026470	chr1	157005833	157050647	+	2536	-1.73		-1
Pik3ip1	ENSMUSG00000034614	chr11	3230404	3242974	+	4272	-1.73		-1
Impdh1	ENSMUSG00000003500	chr6	29150434	29166364	-	3294	-1.731		-1
Prpsap1	ENSMUSG00000015869	chr11	116332159	116355516	-	3407	-1.734		-1
Shkbp1	ENSMUSG00000089832	chr7	28127152	28141038	-	4230	-1.734		-1
Avpi1	ENSMUSG00000018821	chr19	42197763	42203549	-	1236	-1.734		-1
Usp5	ENSMUSG00000038429	chr6	124765037	124779502	-	4621	-1.735		-1
Acsf2	ENSMUSG00000076435	chr11	94418416	94463185	-	3432	-1.736		-1
Aaas	ENSMUSG00000036678	chr15	102168678	102181190	-	1816	-1.737		-1
Slc10a3	ENSMUSG00000032806	chrX	71614556	71618688	-	2023	-1.738		-1
Speg	ENSMUSG00000026207	chr1	75371872	75428895	+	12538	-1.738		-1
Ptcd1	ENSMUSG00000029624	chr5	145908383	145927977	-	3307	-1.739		-1
Asb13	ENSMUSG00000033781	chr13	3633278	3651020	+	3347	-1.739		-1
H1fx	ENSMUSG00000044927	chr6	87930416	87931476	-	1061	-1.739		-1
Dgcr14	ENSMUSG00000003527	chr16	17900804	17911441	-	2819	-1.739		-1
Mapre3	ENSMUSG00000029166	chr5	31117014	31168479	+	3407	-1.742		-1
Sqstm1	ENSMUSG00000015837	chr11	50012868	50024329	-	3356	-1.743		-1
Scn1b	ENSMUSG00000019194	chr7	31901544	31911964	-	1514	-1.743		-1
Gusb	ENSMUSG00000025534	chr5	130464881	130478919	-	4548	-1.743		-1
Dyrk1b	ENSMUSG00000002409	chr7	28964488	28972313	+	2695	-1.743		-1
Rnf181	ENSMUSG00000055850	chr6	72309708	72312715	-	2070	-1.744		-1
Tmem64	ENSMUSG00000043252	chr4	15192978	15213900	+	4670	-1.745		-1

Rpl36al	ENSMUSG00000049751	chr12	70283718	70285070	-	1353	-1.746	-1
Jtb	ENSMUSG00000027937	chr3	90035519	90039760	+	1531	-1.747	-1
Pusl1	ENSMUSG00000051557	chr4	155261988	155265890	-	2329	-1.747	-1
Cutc	ENSMUSG00000025193	chr19	43827486	43843128	+	1422	-1.747	-1
Fbxl12	ENSMUSG00000066892	chr9	20422562	20449211	-	5090	-1.747	-1
Eef1g	ENSMUSG00000071644	chr19	9041531	9052969	+	2600	-1.748	-1
Polr2h	ENSMUSG00000021018	chr16	20717738	20722340	+	1448	-1.748	-1
Sh3gl1	ENSMUSG00000003200	chr17	56156176	56176058	-	2070	-1.749	-1
2610528 E23Rik	ENSMUSG00000022748	chr16	57302312	57606979	-	1837	-1.75	-1
Snrpc	ENSMUSG00000024217	chr17	27976919	27988913	+	6217	-1.75	-1
Pafah1b 3	ENSMUSG00000005447	chr7	26080068	26083005	-	2107	-1.754	-1
Sgsh	ENSMUSG00000005043	chr11	119204739	119216850	-	4414	-1.756	-1
1110012 L19Rik	ENSMUSG00000045237	chrX	67639052	67642592	+	1094	-1.756	-1
Med18	ENSMUSG00000066042	chr4	132014646	132019836	-	1998	-1.757	-1
Taf8	ENSMUSG00000023980	chr17	47624999	47639241	-	2805	-1.757	-1
Prrc1	ENSMUSG00000024594	chr18	57514387	57552373	+	4801	-1.757	-1
Gm1416 7	ENSMUSG00000087433	chr2	151366467	151368084	-	433	-1.758	-1
Mrps10	ENSMUSG00000034729	chr17	47505836	47518366	+	8079	-1.758	-1
Ranbp3	ENSMUSG00000002372	chr17	56812680	56851192	+	2479	-1.759	-1
Plekhh1	ENSMUSG00000060716	chr12	80130150	80182635	+	6459	-1.76	-1
Tars2	ENSMUSG00000028107	chr3	95543899	95558900	-	4438	-1.76	-1
Dpep1	ENSMUSG00000019278	chr8	125710135	125725710	+	2241	-1.761	-1
B3gnt8	ENSMUSG00000059479	chr7	26412643	26414509	+	1475	-1.762	-1
Mdh2	ENSMUSG00000019179	chr5	136254350	136266268	+	2089	-1.762	-1
Suclg1	ENSMUSG00000052738	chr6	73198376	73226905	+	4617	-1.762	-1
Gna15	ENSMUSG00000034792	chr10	80965051	80986970	-	2135	-1.763	-1
Scyl1	ENSMUSG00000024941	chr19	5758427	5771401	-	2637	-1.764	-1
Dhx34	ENSMUSG00000006019	chr7	16782496	16807386	-	4596	-1.764	-1
Msrb2	ENSMUSG00000023094	chr2	19293067	19316603	+	1377	-1.765	-1
Mrpl10	ENSMUSG00000001445	chr11	96902874	96910527	+	1708	-1.765	-1
Nipal3	ENSMUSG00000028803	chr4	135001335	135050953	-	7506	-1.766	-1
Pygo2	ENSMUSG00000047824	chr3	89234136	89239050	+	3631	-1.766	-1
Gm7367	ENSMUSG00000047370	chr7	67300424	67301056	+	633	-1.767	-1
Immp1l	ENSMUSG00000042670	chr2	105744795	105805715	+	955	-1.768	-1
Hspe1	ENSMUSG00000073676	chr1	55144976	55148151	+	1474	-1.768	-1
Tcf25	ENSMUSG00000001472	chr8	125897724	125928073	+	3771	-1.769	-1
Rps26-ps1	ENSMUSG00000059775	chr8	109963051	109963463	-	413	-1.769	-1
Fbxl19	ENSMUSG00000030811	chr7	134890289	134912441	+	3409	-1.769	-1
Rer1	ENSMUSG00000029048	chr4	154448219	154460491	-	1814	-1.769	-1
L3mbtl2	ENSMUSG00000022394	chr15	81494319	81518745	+	5945	-1.771	-1

U2af2	ENSMUSG00000030435	chr7	5013745	5031534	+	2253	-1.771	-1
1110058 L19Rik	ENSMUSG00000026154	chr1	24002778	24012495	-	874	-1.771	-1
Creld1	ENSMUSG00000030284	chr6	113433291	113443332	+	3816	-1.771	-1
2810025 M15Rik	ENSMUSG00000049881	chr1	159342483	159350366	+	1193	-1.772	-1
Dtymk	ENSMUSG00000026281	chr1	95689153	95698511	-	2631	-1.772	-1
Cdk16	ENSMUSG00000031065	chrX	20265080	20277006	+	4378	-1.773	-1
Nfib	ENSMUSG00000008575	chr4	81936077	82351654	-	14957	-1.773	-1
Pcsk4	ENSMUSG00000020131	chr10	79784028	79792243	-	2958	-1.774	-1
Cdc37	ENSMUSG00000019471	chr9	20942985	20954350	-	2456	-1.775	-1
Pmvk	ENSMUSG00000027952	chr3	89263040	89272935	+	1514	-1.778	-1
Erf	ENSMUSG00000040857	chr7	26027580	26035780	-	3607	-1.778	-1
Dolk	ENSMUSG00000075419	chr2	30139749	30141874	-	2126	-1.78	-1
Ybey	ENSMUSG00000033126	chr10	75922314	75931859	-	4520	-1.781	-1
Aldh3b1	ENSMUSG00000024885	chr19	3913493	3929761	-	1875	-1.782	-1
Ung	ENSMUSG00000029591	chr5	114580395	114589332	+	2566	-1.782	-1
Hps4	ENSMUSG00000042328	chr5	112772103	112807434	+	3948	-1.783	-1
Tmem2 29b	ENSMUSG00000046157	chr12	80062782	80108614	-	4732	-1.783	-1
Gpr155	ENSMUSG00000041762	chr2	73179563	73224629	-	5915	-1.784	-1
Ndufa10	ENSMUSG00000026260	chr1	94335587	94370437	-	2272	-1.784	-1
Gpr160	ENSMUSG00000037661	chr3	30754872	30796114	+	4227	-1.784	-1
Cdcp1	ENSMUSG00000035498	chr9	123079942	123125156	-	6765	-1.786	-1
Plekhg5	ENSMUSG00000039713	chr4	151446607	151489509	+	4945	-1.786	-1
Krt4	ENSMUSG00000059668	chr15	101748967	101755166	-	2132	-1.786	-1
Ighv9-3	ENSMUSG00000076664	chr12	115378903	115379333	-	351	-1.787	-1
H2-Eb1	ENSMUSG00000060586	chr17	34442828	34453144	+	1189	-1.787	-1
Tmem5 9	ENSMUSG00000028618	chr4	106851004	106873601	+	2056	-1.789	-1
Snx21	ENSMUSG00000050373	chr2	164611323	164619316	+	4760	-1.79	-1
Rnaset2 b	ENSMUSG00000079724	chr17	7183209	7202542	+	1013	-1.791	-1
Lrrc41	ENSMUSG00000028703	chr4	115747874	115769648	+	3713	-1.791	-1
Wdr4	ENSMUSG00000024037	chr17	31631268	31656919	-	5432	-1.791	-1
Rpap1	ENSMUSG00000034032	chr2	119589040	119613273	-	6636	-1.792	-1
Commd 7	ENSMUSG00000056941	chr2	153442669	153458517	-	5493	-1.793	-1
Snd1	ENSMUSG00000001424	chr6	28425139	28885162	+	7551	-1.794	-1
G43009 5P16Rik	ENSMUSG00000074203	chr8	87246906	87250743	+	721	-1.797	-1
Zer1	ENSMUSG00000039686	chr2	29952803	29980105	-	9784	-1.8	-1
Prkab1	ENSMUSG00000029513	chr5	116463595	116474517	-	3066	-1.801	-1
Adap1	ENSMUSG00000056413	chr5	139747830	139801576	-	3239	-1.803	-1

Pkig	ENSMUSG00000035268	chr2	163484122	163551894	+	3990	-1.804		-1
Bysl	ENSMUSG00000023988	chr17	47736280	47748441	-	3854	-1.805		-1
Prelid1	ENSMUSG00000021486	chr13	55423416	55426632	+	1088	-1.805		-1
Cyp2s1	ENSMUSG00000040703	chr7	26587494	26601932	-	4938	-1.806		-1
Gm12166	ENSMUSG00000069899	chr11	45864856	45865823	-	968	-1.806		-1
Cachd1	ENSMUSG00000028532	chr4	100449280	100701825	+	6020	-1.807		-1
Cth	ENSMUSG00000028179	chr3	157557212	157588041	-	2263	-1.807		-1
Sec13	ENSMUSG00000030298	chr6	113678061	113690737	-	1663	-1.807		-1
Fkbpl	ENSMUSG00000033739	chr17	34781709	34783269	+	1336	-1.808		-1
Cpne8	ENSMUSG00000052560	chr15	90317913	90509863	-	4164	-1.808		-1
Slc37a1	ENSMUSG00000024036	chr17	31432428	31487641	+	3626	-1.808		-1
Arl4d	ENSMUSG00000034936	chr11	101526855	101529146	+	1382	-1.809		-1
Trip6	ENSMUSG00000023348	chr5	137751127	137755632	-	1923	-1.809		-1
Ndufa8	ENSMUSG00000026895	chr2	35891846	35904926	-	861	-1.81		-1
Cdipt	ENSMUSG00000030682	chr7	134119863	134124013	+	1876	-1.811		-1
Plekhm2	ENSMUSG00000028917	chr4	141181649	141220814	-	5186	-1.811		-1
Bri3	ENSMUSG00000047843	chr5	145005306	145207626	+	1648	-1.812		-1
Dhrs1	ENSMUSG00000002332	chr14	56357857	56364527	-	2760	-1.814		-1
Skiv2l	ENSMUSG00000040356	chr17	34976173	34987155	-	4977	-1.814		-1
Trappc1	ENSMUSG00000049299	chr11	69137482	69139295	+	1112	-1.814		-1
Lsm2	ENSMUSG00000007050	chr17	35118807	35122836	+	3152	-1.815		-1
Dbnl	ENSMUSG00000020476	chr11	5688491	5700965	+	2346	-1.815		-1
9130020K20Rik	ENSMUSG00000086568	chr4	135904465	135906249	+	1785	-1.816		-1
Ppp2r5d	ENSMUSG00000059409	chr17	46819940	46841951	-	2926	-1.818		-1
Vps45	ENSMUSG00000015747	chr3	95803755	95862389	-	3991	-1.818		-1
2610301G19Rik	ENSMUSG00000033712	chr14	70537984	70553618	-	3716	-1.818		-1
Fam105b	ENSMUSG00000046034	chr15	27535674	27560448	-	1491	-1.818		-1
Ccrl2	ENSMUSG00000043953	chr9	110957339	110959774	-	2110	-1.819		-1
Cox6b2	ENSMUSG00000051811	chr7	4703396	4704696	-	551	-1.819		-1
Plekho1	ENSMUSG00000015745	chr3	95792352	95799924	-	2172	-1.819		-1
Nit2	ENSMUSG00000022751	chr16	57156784	57167445	-	1280	-1.82		-1
Tulp3	ENSMUSG00000001521	chr6	128271158	128305869	-	6704	-1.82		-1
Tmem39b	ENSMUSG00000053730	chr4	129353599	129374082	-	2286	-1.821		-1
Eif2c4	ENSMUSG00000042500	chr4	126166785	126210716	-	7569	-1.821		-1
Tmem45a	ENSMUSG00000022754	chr16	56805274	56886279	-	3056	-1.821		-1
Rnf169	ENSMUSG00000092273	chr7	107083788	107114244	-	717	-1.822		-1
Dmpk	ENSMUSG00000030409	chr7	19669198	19679170	+	4346	-1.825		-1

Sorl1	ENSMUSG00000049313	chr9	41772803	41932380	-	11321	-1.825	-1
Arap1	ENSMUSG00000032812	chr7	108496581	108561100	+	7564	-1.825	-1
Psmc3ip	ENSMUSG00000019303	chr11	100953137	100956750	-	1818	-1.826	-1
Phlda2	ENSMUSG00000010760	chr7	150687450	150688446	-	757	-1.827	-1
Cxx1c	ENSMUSG00000051851	chrX	50911099	50912309	+	1141	-1.827	-1
Rnf135	ENSMUSG00000020707	chr11	79997353	80013259	+	2140	-1.828	-1
1300002E11Rik	ENSMUSG00000086651	chr16	21794420	21809110	+	3955	-1.83	-1
Vps72	ENSMUSG00000008958	chr3	94914944	94926973	+	3639	-1.831	-1
Crlf2	ENSMUSG00000033467	chr5	109983730	109988012	-	1460	-1.831	-1
Fxyd6	ENSMUSG00000066705	chr9	45178268	45204241	+	1791	-1.832	-1
Slc43a1	ENSMUSG00000027075	chr2	84679007	84703751	+	5716	-1.834	-1
1700056E22Rik	ENSMUSG00000044854	chr1	185856911	185857453	-	543	-1.834	-1
Slco4a1	ENSMUSG00000038963	chr2	180190950	180209572	+	4799	-1.835	-1
Oasl1	ENSMUSG00000041827	chr5	115373249	115387924	+	2608	-1.835	-1
Plekhh3	ENSMUSG00000035172	chr11	101023993	101032665	-	3582	-1.835	-1
Leng9	ENSMUSG00000043432	chr7	4099785	4101474	-	1690	-1.836	-1
Gm16286	ENSMUSG00000024571	chr18	80403504	80409480	+	3075	-1.836	-1
Nat14	ENSMUSG00000035285	chr7	4873853	4876608	+	1612	-1.837	-1
Eif3d	ENSMUSG00000016554	chr15	77789428	77801254	-	1910	-1.837	-1
Hsd17b11	ENSMUSG00000029311	chr5	104418781	104450938	-	3265	-1.837	-1
Snrpb	ENSMUSG00000027404	chr2	129997150	130005139	-	1976	-1.839	-1
Armc5	ENSMUSG00000042178	chr7	135380871	135388614	+	3834	-1.84	-1
Prcc	ENSMUSG00000004895	chr3	87662822	87689530	-	2102	-1.841	-1
Zfp282	ENSMUSG00000025821	chr6	47827203	47858484	+	7663	-1.842	-1
Gm10269	ENSMUSG00000091449	chr18	20841060	20841504	-	445	-1.845	-1
Pcca	ENSMUSG00000041650	chr14	122933546	123290322	+	5210	-1.845	-1
Nit1	ENSMUSG00000013997	chr1	173268139	173275777	-	4932	-1.845	-1
Necap2	ENSMUSG00000028923	chr4	140622427	140634272	-	3095	-1.845	-1
Pafah2	ENSMUSG00000037366	chr4	133952235	133983328	+	3616	-1.848	-1
Zfp580	ENSMUSG00000055633	chr7	5003134	5005324	+	1101	-1.848	-1
Tbrg4	ENSMUSG00000000384	chr11	6515601	6526070	-	3696	-1.849	-1
Dhx37	ENSMUSG00000029480	chr5	125894230	125914491	-	4758	-1.852	-1
Rrp36	ENSMUSG00000023971	chr17	46804407	46811236	-	1227	-1.853	-1
Camk2n2	ENSMUSG00000051146	chr16	20619288	20621360	-	1291	-1.854	-1
Zfyve27	ENSMUSG00000018820	chr19	42238441	42269080	+	8623	-1.854	-1
Pkp4	ENSMUSG00000026991	chr2	58998907	59193265	+	5708	-1.854	-1

2300009A05Rik	ENSMUSG00000032403	chr9	63242254	63247051	-	598	-1.855	-1
S100a1	ENSMUSG00000044080	chr3	90314956	90318314	-	694	-1.855	-1
Polr2l	ENSMUSG00000038489	chr7	148657759	148661031	-	1650	-1.857	-1
Fam136a	ENSMUSG00000057497	chr6	86315677	86320051	+	1605	-1.858	-1
Zfp428	ENSMUSG00000064264	chr7	25292025	25300701	+	1308	-1.859	-1
Selk	ENSMUSG00000042682	chr14	30781494	30788260	+	1240	-1.859	-1
Bin3	ENSMUSG00000022089	chr14	70499952	70537998	+	1712	-1.86	-1
Capns1	ENSMUSG00000001794	chr7	30971961	30980183	-	2397	-1.861	-1
Mrps30	ENSMUSG00000021731	chr13	119168917	119176059	-	1590	-1.863	-1
Ubqln4	ENSMUSG00000008604	chr3	88357638	88373647	+	3372	-1.864	-1
Ficd	ENSMUSG00000053334	chr5	114185791	114190609	+	3154	-1.864	-1
B4galnt1	ENSMUSG00000006731	chr10	126602247	126609385	+	2428	-1.866	-1
Mamstr	ENSMUSG00000042918	chr7	52895347	52901891	+	4877	-1.867	-1
Sec61a1	ENSMUSG00000030082	chr6	88453595	88468899	-	6407	-1.869	-1
Fam117a	ENSMUSG00000038893	chr11	95198332	95243186	+	3029	-1.869	-1
Fam113a	ENSMUSG00000037773	chr2	130242983	130250437	-	5072	-1.869	-1
Sra1	ENSMUSG00000006050	chr18	36826335	36830850	-	2660	-1.869	-1
Daglb	ENSMUSG00000039206	chr5	144225361	144265310	+	5677	-1.869	-1
Tpi1	ENSMUSG00000023456	chr6	124760604	124764314	-	2209	-1.87	-1
Fam114a2	ENSMUSG00000020523	chr11	57296492	57332146	-	3108	-1.87	-1
Cry2	ENSMUSG00000068742	chr2	92243803	92274200	-	6320	-1.87	-1
Purb	ENSMUSG00000091207	chr11	6374795	6375920	-	1126	-1.873	-1
Ints3	ENSMUSG00000027933	chr3	90195310	90237558	-	4452	-1.874	-1
Fam193b	ENSMUSG00000021495	chr13	55640680	55672481	-	4338	-1.874	-1
Rab38	ENSMUSG00000030559	chr7	95578783	95639976	+	1471	-1.875	-1
Rpl35	ENSMUSG00000062997	chr2	38857100	38861144	-	1241	-1.875	-1
Mms19	ENSMUSG00000025159	chr19	42015576	42055647	-	10415	-1.878	-1
BC017612	ENSMUSG00000058173	chr9	15309931	15349704	+	954	-1.878	-1
Fam173b	ENSMUSG00000039065	chr15	31531607	31550982	+	5262	-1.878	-1
Neurl4	ENSMUSG00000047284	chr11	69714574	69727322	+	5889	-1.878	-1
Foxred1	ENSMUSG00000039048	chr9	35011791	35018640	-	4064	-1.879	-1
2900092E17Rik	ENSMUSG00000030680	chr7	134144996	134163841	-	4749	-1.879	-1
Dnajc15	ENSMUSG00000022013	chr14	78226024	78274724	-	648	-1.881	-1
Tmbim6	ENSMUSG00000023010	chr15	99223313	99240480	+	3806	-1.881	-1
Mrpl16	ENSMUSG00000024683	chr19	11844881	11849434	+	1146	-1.882	-1

Abca3	ENSMUSG00000024130	chr17	24488895	24547146	+	8797	-1.883	-1
Pla2g15	ENSMUSG00000031903	chr8	108674299	108688613	+	2704	-1.884	-1
Nedd8	ENSMUSG00000010376	chr14	56281102	56290743	-	604	-1.884	-1
1810031K17Rik	ENSMUSG00000033159	chr1	75131128	75139271	-	3678	-1.885	-1
5033430I15Rik	ENSMUSG00000038152	chr13	46060622	46061869	+	721	-1.885	-1
Xrcc6bp1	ENSMUSG00000025436	chr10	126305486	126338427	-	1362	-1.886	-1
Mrps18b	ENSMUSG00000024436	chr17	36047324	36053334	-	2089	-1.887	-1
Tmem198	ENSMUSG00000051703	chr1	75476107	75482280	+	2504	-1.888	-1
Nbas	ENSMUSG00000020576	chr12	13275933	13590616	+	8219	-1.892	-1
Eef2	ENSMUSG00000034994	chr10	80639376	80645252	+	3098	-1.892	-1
2310061I04Rik	ENSMUSG00000050705	chr17	36029622	36034376	-	2414	-1.894	-1
Ddx39b	ENSMUSG00000019432	chr17	35378691	35390652	+	3783	-1.895	-1
Tnfrsf25	ENSMUSG00000024793	chr4	151490043	151494228	+	2277	-1.898	-1
Rbm10	ENSMUSG00000031060	chrX	20194629	20228027	+	3731	-1.903	-1
Ndufb5	ENSMUSG00000027673	chr3	32635912	32650488	+	2023	-1.904	-1
Btbd9	ENSMUSG00000062202	chr17	30352469	30713394	-	10036	-1.904	-1
Lpcat2	ENSMUSG00000033192	chr8	95379249	95443178	+	3377	-1.904	-1
E030030I06Rik	ENSMUSG00000055657	chr10	21864227	21868629	-	429	-1.905	-1
Slc46a3	ENSMUSG00000029650	chr5	148690013	148706391	-	2458	-1.905	-1
Tril	ENSMUSG00000043496	chr6	53765465	53770819	-	5355	-1.905	-1
Limd2	ENSMUSG00000040699	chr11	106017570	106022174	-	3707	-1.908	-1
Siah2	ENSMUSG00000036432	chr3	58478860	58496322	-	2999	-1.908	-1
Rnmtl1	ENSMUSG00000038046	chr11	76057217	76064121	+	1935	-1.909	-1
Elf3	ENSMUSG00000003051	chr1	137150151	137155049	-	2000	-1.909	-1
Tuba1b	ENSMUSG00000023004	chr15	98761856	98764996	-	1875	-1.91	-1
Fbxw8	ENSMUSG00000032867	chr5	118514990	118605467	-	4978	-1.91	-1
Nop16	ENSMUSG00000025869	chr13	54685546	54691451	-	1845	-1.911	-1
1500001M20Rik	ENSMUSG00000030316	chr6	114954399	114987894	-	5276	-1.911	-1
Arfgap1	ENSMUSG00000027575	chr2	180701930	180717231	+	5368	-1.911	-1
Cobra1	ENSMUSG00000013465	chr2	25055232	25067009	-	3432	-1.913	-1
Ilk	ENSMUSG00000030890	chr7	112885106	112891439	+	2744	-1.913	-1
Brp44	ENSMUSG00000026568	chr1	167391168	167411345	+	3804	-1.914	-1
Serf2	ENSMUSG00000074884	chr2	121274931	121284049	+	5455	-1.914	-1
Zfpm1	ENSMUSG00000049577	chr8	124806041	124861151	+	3503	-1.915	-1
Zc3hc1	ENSMUSG00000039130	chr6	30316380	30341028	-	4601	-1.915	-1
Lrp10	ENSMUSG00000022175	chr14	55083001	55090334	+	4201	-1.916	-1
Rpl26	ENSMUSG00000060938	chr11	68715085	68720491	+	1444	-1.917	-1

Flt3l	ENSMUSG00000089989	chr7	52386558	52391802	-	3712	-1.92	-1
Sin3b	ENSMUSG00000031622	chr8	75247187	75282102	+	4298	-1.92	-1
Prkra	ENSMUSG00000002731	chr2	76467955	76486072	-	2028	-1.92	-1
Adra2a	ENSMUSG00000033717	chr19	54119672	54123472	+	3801	-1.921	-1
Fmo2	ENSMUSG00000040170	chr1	164804448	164828857	-	5545	-1.921	-1
Gltpd1	ENSMUSG00000029073	chr4	155238832	155243549	-	2709	-1.922	-1
Eif4a3	ENSMUSG00000025580	chr11	119149677	119161403	-	2122	-1.922	-1
Tprgl	ENSMUSG00000029030	chr4	153531594	153534775	-	2242	-1.925	-1
Polrmt	ENSMUSG00000020329	chr10	79198868	79209326	-	4685	-1.925	-1
Zdhhc4	ENSMUSG00000001844	chr5	144078168	144090935	-	2864	-1.927	-1
1110051M20Rik	ENSMUSG00000040591	chr2	91115225	91284861	-	5202	-1.927	-1
Msto1	ENSMUSG00000068922	chr3	88709029	88717921	-	5418	-1.928	-1
Pcyox1l	ENSMUSG00000024579	chr18	61856491	61867289	-	2645	-1.928	-1
Cct7	ENSMUSG00000030007	chr6	85401499	85418471	+	2452	-1.929	-1
Mad2l1bp	ENSMUSG00000034509	chr17	46284338	46290502	-	1270	-1.93	-1
Gpn1	ENSMUSG00000064037	chr5	31797114	31815276	+	7845	-1.93	-1
Lpcat3	ENSMUSG00000004270	chr6	124613045	124654436	+	3268	-1.93	-1
Commd1	ENSMUSG00000051355	chr11	22734744	22882382	-	8388	-1.93	-1
Tnfrsf4	ENSMUSG00000029075	chr4	155387952	155390721	+	1365	-1.932	-1
Ccdc94	ENSMUSG00000003208	chr17	56098610	56107372	+	1311	-1.932	-1
Lrfn4	ENSMUSG00000045045	chr19	4611785	4615667	-	3366	-1.932	-1
Atg4b	ENSMUSG00000026280	chr1	95648077	95686372	+	6037	-1.933	-1
Mrps14	ENSMUSG00000058267	chr1	162125346	162132301	+	3258	-1.933	-1
Cxxc1	ENSMUSG00000024560	chr18	74375785	74381145	+	2618	-1.934	-1
Tbc1d7	ENSMUSG00000021368	chr13	43247113	43266728	-	1097	-1.934	-1
Gbf1	ENSMUSG00000025224	chr19	46226999	46361000	+	9062	-1.934	-1
Ndor1	ENSMUSG00000006471	chr2	25100340	25111542	-	5836	-1.934	-1
Pxmp4	ENSMUSG00000000876	chr2	154411494	154429444	-	3114	-1.934	-1
Eef1b2	ENSMUSG00000025967	chr1	63223399	63227060	+	2532	-1.935	-1
Lsm6	ENSMUSG00000031683	chr8	81328764	81345039	-	4125	-1.936	-1
Pqbp1	ENSMUSG00000031157	chrX	7471645	7476395	-	1636	-1.936	-1
Zfp821	ENSMUSG00000031728	chr8	112229449	112248832	+	2396	-1.937	-1
Bag6	ENSMUSG00000024392	chr17	35272123	35284265	+	6213	-1.937	-1
Adrb2	ENSMUSG00000045730	chr18	62337471	62339613	-	2143	-1.94	-1
2410003K15Rik	ENSMUSG00000029815	chr6	49023794	49036750	+	3079	-1.941	-1
Tm9sf1	ENSMUSG00000002320	chr14	56254802	56262643	-	3880	-1.943	-1
Rpl14	ENSMUSG00000025794	chr9	120480632	120483770	+	937	-1.944	-1
Glrx3	ENSMUSG00000031068	chr7	144629331	144660277	+	1651	-1.946	-1
Hoga1	ENSMUSG00000025176	chr19	42120100	42145443	+	2259	-1.946	-1
Plcb4	ENSMUSG00000039943	chr2	135484747	135840329	+	8970	-1.948	-1
Rft1	ENSMUSG00000052395	chr14	31467546	31504503	+	4360	-1.948	-1

Lyz1	ENSMUSG00000069515	chr10	116724852	116729924	-	1264	-1.949	-1
Jrk	ENSMUSG00000046380	chr15	74532842	74539965	-	5742	-1.949	-1
Irf7	ENSMUSG00000025498	chr7	148448605	148452380	-	3023	-1.951	-1
Gpr89	ENSMUSG00000028096	chr3	96672204	96709269	-	5365	-1.952	-1
Tmco4	ENSMUSG00000041143	chr4	138528803	138615086	+	3604	-1.952	-1
Aes	ENSMUSG00000054452	chr10	81022239	81029107	+	1452	-1.952	-1
Fam63a	ENSMUSG00000038712	chr3	95085267	95100088	+	6458	-1.953	-1
Athl1	ENSMUSG00000062031	chr7	148127290	148133563	+	3417	-1.954	-1
Tmem11	ENSMUSG00000043284	chr11	60677954	60692774	-	1745	-1.956	-1
Rpl13	ENSMUSG00000000740	chr8	125626250	125629144	+	2352	-1.956	-1
Lgi4	ENSMUSG00000036560	chr7	31844361	31855954	+	4584	-1.958	-1
Pvrl4	ENSMUSG00000006411	chr1	173300230	173318729	+	3746	-1.959	-1
Usp21	ENSMUSG00000053483	chr1	173212076	173218122	-	4756	-1.959	-1
Tmem9b	ENSMUSG00000031021	chr7	116879348	116896389	-	2002	-1.961	-1
AU021092	ENSMUSG00000051669	chr16	5211921	5222392	-	1363	-1.962	-1
Gne	ENSMUSG00000028479	chr4	44046947	44097049	-	6358	-1.964	-1
Mettl17	ENSMUSG00000004561	chr14	52504517	52512714	+	3970	-1.968	-1
Samd1	ENSMUSG00000079003	chr8	86521571	86524285	+	2071	-1.969	-1
4921531C22Rik	ENSMUSG00000085704	chr2	179710698	179713718	+	1967	-1.972	-1
Derl1	ENSMUSG00000022365	chr15	57701057	57723973	-	3145	-1.972	-1
Rp9	ENSMUSG00000032239	chr9	22215491	22272802	-	2043	-1.972	-1
Znrd1	ENSMUSG00000036315	chr17	37091303	37095504	-	817	-1.973	-1
Opa3	ENSMUSG00000052214	chr7	19813683	19841892	+	4554	-1.975	-1
Accs	ENSMUSG00000040272	chr2	93673624	93690100	-	8364	-1.976	-1
C7	ENSMUSG00000079105	chr15	4938762	5013740	-	2676	-1.976	-1
Kcnq4	ENSMUSG00000028631	chr4	120368743	120421217	-	4024	-1.976	-1
Atp5b	ENSMUSG00000025393	chr10	127520329	127527447	+	4237	-1.978	-1
Pcbp4	ENSMUSG00000023495	chr9	106356188	106366338	+	2015	-1.978	-1
Rab15	ENSMUSG00000021062	chr12	77898947	77923895	-	4614	-1.979	-1
Parl	ENSMUSG00000033918	chr16	20279893	20302460	-	3892	-1.98	-1
Ccdc51	ENSMUSG00000025645	chr9	108985008	108995877	+	2399	-1.982	-1
Pstk	ENSMUSG00000063179	chr7	138514655	138531353	+	2179	-1.982	-1
Fam100b	ENSMUSG00000050628	chr11	116295408	116300391	+	1532	-1.984	-1
Usp2	ENSMUSG00000032010	chr9	43875104	43903710	+	4807	-1.986	-1
Map4k2	ENSMUSG00000024948	chr19	6341135	6355615	+	6646	-1.987	-1
Fbxo46	ENSMUSG00000050428	chr7	19705208	19723610	+	3025	-1.987	-1
Pus1	ENSMUSG00000029507	chr5	111202686	111209678	-	2180	-1.987	-1
2810408M09Rik	ENSMUSG00000039725	chr2	165315610	165318824	-	2118	-1.988	-1
Rgl2	ENSMUSG00000041354	chr17	34066488	34074632	+	4625	-1.989	-1
Nsun5	ENSMUSG00000000916	chr5	135845823	135852675	+	3142	-1.989	-1

Tatdn3	ENSMUSG00000026632	chr1	192869705	192886811	-	2786	-1.989	-1
Nudt18	ENSMUSG00000045211	chr14	70977654	70982378	+	3716	-1.99	-1
Taf6	ENSMUSG00000036980	chr5	138619845	138628679	-	3779	-1.992	-1
Rgma	ENSMUSG00000070509	chr7	80520406	80564785	+	3782	-1.992	-1
Ypel1	ENSMUSG00000022773	chr16	17069789	17087138	+	4635	-1.992	-1
Pbxip1	ENSMUSG00000042613	chr3	89240628	89254874	+	4674	-1.993	-1
Ndufv2	ENSMUSG00000024099	chr17	66428135	66450899	-	1990	-1.993	-1
0610009O20Rik	ENSMUSG00000024442	chr18	38409903	38422283	+	2404	-1.994	-1
Exoc7	ENSMUSG00000020792	chr11	116149315	116168547	-	4367	-1.994	-1
Axin2	ENSMUSG00000000142	chr11	108781663	108812097	+	5506	-1.994	-1
Lmo4	ENSMUSG00000028266	chr3	143851494	143868184	-	3295	-1.995	-1
Pltp	ENSMUSG00000017754	chr2	164665018	164683211	-	2562	-1.996	-1
Mrpl27	ENSMUSG00000024414	chr11	94515081	94521403	+	985	-1.996	-1
Gli1	ENSMUSG00000025407	chr10	126766945	126778645	-	3782	-1.998	-1
Zfp691	ENSMUSG00000045268	chr4	118842118	118846800	-	2399	-1.999	-1
Gpd1l	ENSMUSG00000050627	chr9	114808459	114843105	-	5077	-1.999	-1
Dffa	ENSMUSG00000028974	chr4	148478255	148494756	+	3540	-2	-1
Sbsn	ENSMUSG00000046056	chr7	31536490	31541152	+	2285	-2	-1
Sfn	ENSMUSG00000047281	chr4	133156471	133158083	-	1613	-2.001	-1
Atpif1	ENSMUSG00000054428	chr4	132086470	132089574	-	1441	-2.001	-1
2510039O18Rik	ENSMUSG00000044496	chr4	147315004	147321423	+	2731	-2.001	-1
Ndufc2	ENSMUSG00000030647	chr7	104548513	104556312	+	754	-2.003	-1
Atg2a	ENSMUSG00000024773	chr19	6241668	6262335	+	7693	-2.003	-1
Nip7	ENSMUSG00000031917	chr8	109580777	109584826	+	3031	-2.004	-1
Dos	ENSMUSG00000035640	chr10	79593196	79603580	-	5046	-2.004	-1
Atf3	ENSMUSG00000026628	chr1	192994175	193007219	-	1984	-2.005	-1
Gm3452	ENSMUSG00000091498	chr12	112096775	112097104	-	330	-2.006	-1
Tsen2	ENSMUSG00000042389	chr6	115494682	115528368	+	6437	-2.006	-1
Anxa4	ENSMUSG00000029994	chr6	86686834	86743578	-	4645	-2.007	-1
Nqo1	ENSMUSG00000003849	chr8	109912125	109927106	-	1553	-2.008	-1
Jagn1	ENSMUSG00000051256	chr6	113392563	113398223	+	1201	-2.009	-1
Apoa2	ENSMUSG00000005681	chr1	173155185	173156510	+	537	-2.009	-1
Anxa6	ENSMUSG00000018340	chr11	54792610	54846947	-	2930	-2.01	-1
Unc45a	ENSMUSG00000030533	chr7	87470178	87485891	-	3991	-2.01	-1
Pamr1	ENSMUSG00000027188	chr2	102390169	102483198	+	6046	-2.011	-1
Pdcd2	ENSMUSG00000014771	chr17	15656172	15664265	-	2582	-2.015	-1
Fbxo21	ENSMUSG00000032898	chr5	118426779	118460200	+	3909	-2.016	-1
Rnf180	ENSMUSG00000021720	chr13	105921444	106061119	-	1987	-2.016	-1
Krt18	ENSMUSG00000023043	chr15	101858647	101862457	+	1400	-2.017	-1
Lpcat4	ENSMUSG00000027134	chr2	112079625	112087268	+	2823	-2.017	-1
0610007L01Rik	ENSMUSG00000053094	chr5	130695614	130719635	+	3783	-2.017	-1

Rfc2	ENSMUSG00000023104	chr5	135058560	135074198	+	1518	-2.018	-1
2210016L21Rik	ENSMUSG00000029559	chr5	115392167	115399792	+	3303	-2.018	-1
Traf7	ENSMUSG00000052752	chr17	24645507	24664883	-	5872	-2.019	-1
Atp6v0e	ENSMUSG00000015575	chr17	26813341	26836589	+	3261	-2.02	-1
Mier2	ENSMUSG00000042570	chr10	79002990	79017944	-	4497	-2.021	-1
Mlx	ENSMUSG00000017801	chr11	100948591	100953521	+	1999	-2.021	-1
A730011L01Rik	ENSMUSG00000039850	chr11	119352661	119372751	+	6138	-2.022	-1
Rpl34	ENSMUSG00000062006	chr3	130429749	130433316	-	1179	-2.022	-1
Ppp2r1a	ENSMUSG00000007564	chr17	21082275	21102880	+	3375	-2.024	-1
Rpl11	ENSMUSG00000059291	chr4	135584180	135609343	-	2697	-2.027	-1
Ergic1	ENSMUSG00000001576	chr17	26698434	26793876	+	6456	-2.027	-1
Oxa1l	ENSMUSG00000000959	chr14	54980525	54989353	+	2534	-2.029	-1
Emd	ENSMUSG00000001964	chrX	71500026	71506887	+	2517	-2.029	-1
Ube2g2	ENSMUSG00000009293	chr10	77085020	77108738	+	2431	-2.03	-1
Ceacam1	ENSMUSG00000074272	chr7	26246721	26262644	-	3736	-2.03	-1
D17Wsu92e	ENSMUSG00000056692	chr17	27888177	27957503	-	4250	-2.031	-1
Pex11b	ENSMUSG00000028102	chr3	96439299	96457024	+	5615	-2.032	-1
Eme2	ENSMUSG00000073436	chr17	25025431	25032032	-	6000	-2.032	-1
Jag2	ENSMUSG00000002799	chr12	114146801	114167706	-	4054	-2.033	-1
Sgta	ENSMUSG00000004937	chr10	80506820	80522923	-	1969	-2.033	-1
Atp6ap1	ENSMUSG00000019087	chrX	71542436	71550060	+	3026	-2.034	-1
Aldh18a1	ENSMUSG00000025007	chr19	40624747	40662953	-	4072	-2.034	-1
Chmp6	ENSMUSG00000025371	chr11	119774755	119780862	+	2432	-2.036	-1
Syvn1	ENSMUSG00000024807	chr19	6046576	6053712	+	4859	-2.038	-1
Rnf25	ENSMUSG00000026171	chr1	74640322	74647971	-	2181	-2.039	-1
Med27	ENSMUSG00000026799	chr2	29202339	29380313	+	3310	-2.041	-1
Zfp446	ENSMUSG00000033961	chr7	13563197	13569689	+	5801	-2.041	-1
Rwdd2b	ENSMUSG00000041079	chr16	87433652	87440818	-	2009	-2.041	-1
Aldh5a1	ENSMUSG00000035936	chr13	24999448	25029530	-	5647	-2.042	-1
Cd151	ENSMUSG00000025510	chr7	148653261	148657378	+	2010	-2.044	-1
Bloc1s1	ENSMUSG00000090247	chr10	128354938	128361091	-	1288	-2.044	-1
Ddx47	ENSMUSG00000030204	chr6	134961630	134973794	+	4952	-2.046	-1
Efna1	ENSMUSG00000027954	chr3	89075655	89085064	-	1830	-2.048	-1
Srebf1	ENSMUSG00000020538	chr11	60012591	60036083	-	5634	-2.049	-1
Hook1	ENSMUSG00000028572	chr4	95633931	95692104	+	8318	-2.049	-1
Ubxn1	ENSMUSG00000071655	chr19	8946049	8950153	+	1068	-2.05	-1
Fzd3	ENSMUSG00000007989	chr14	65819863	65881300	-	4227	-2.052	-1

Tmem177	ENSMUSG00000036975	chr1	121804467	121809785	-	3341	-2.052	-1
Acsl1	ENSMUSG00000018796	chr8	47556395	47621405	+	9449	-2.052	-1
Gm10762	ENSMUSG00000074807	chr2	128792329	128793461	-	1133	-2.052	-1
Ncln	ENSMUSG00000020238	chr10	80948994	80959137	-	3465	-2.053	-1
Zfp672	ENSMUSG00000049755	chr11	58128616	58136848	-	3549	-2.054	-1
Tsc2	ENSMUSG00000002496	chr17	24732882	24769574	-	6347	-2.056	-1
Megf8	ENSMUSG00000045039	chr7	26102183	26150936	+	10159	-2.056	-1
Tcf7l1	ENSMUSG00000055799	chr6	72576372	72739248	-	3323	-2.056	-1
Chmp2a	ENSMUSG00000033916	chr7	13617355	13620126	-	927	-2.056	-1
Hand2	ENSMUSG00000038193	chr8	59799780	59803314	+	2235	-2.057	-1
4930524J08Rik	ENSMUSG00000035364	chr5	100408080	100408682	+	603	-2.057	-1
Rpl6	ENSMUSG00000029614	chr5	121654490	121659250	+	2433	-2.057	-1
Polr1d	ENSMUSG00000029642	chr5	147888626	147922937	+	1985	-2.058	-1
Hsdl1	ENSMUSG00000034189	chr8	122085879	122099100	-	3168	-2.059	-1
Pold1	ENSMUSG00000038644	chr7	51788116	51804219	-	4860	-2.061	-1
Zfp692	ENSMUSG00000037243	chr11	58120571	58128129	+	2110	-2.061	-1
Gm17305	ENSMUSG00000091095	chr11	69177521	69182910	+	507	-2.061	-1
Pim2	ENSMUSG00000031155	chrX	7455388	7460558	+	2795	-2.063	-1
Prr5l	ENSMUSG00000032841	chr2	101554442	101723184	-	4967	-2.065	-1
Fancg	ENSMUSG00000028453	chr4	43015215	43023378	-	3795	-2.066	-1
D030028A08Rik	ENSMUSG00000078700	chr11	96777584	96826374	+	6230	-2.067	-1
Gm3436	ENSMUSG00000078305	chr9	70700357	70702269	+	1913	-2.067	-1
Scmh1	ENSMUSG00000000085	chr4	120077886	120202804	+	7744	-2.068	-1
Keap1	ENSMUSG00000003308	chr9	21034174	21043776	-	4470	-2.069	-1
Bloc1s3	ENSMUSG00000057667	chr7	20091163	20093680	-	1824	-2.07	-1
Pfkfb4	ENSMUSG00000025648	chr9	108893947	108934737	+	4133	-2.071	-1
Bsg	ENSMUSG00000023175	chr10	79167103	79174724	+	1752	-2.071	-1
Klhl26	ENSMUSG00000055707	chr8	72974127	73000842	-	3069	-2.072	-1
AI846148	ENSMUSG00000024970	chr19	7430954	7457516	-	3183	-2.072	-1
Sec14l3	ENSMUSG00000054986	chr11	3964844	3977739	+	2784	-2.073	-1
Dusp12	ENSMUSG00000026659	chr1	172803629	172815671	-	2743	-2.074	-1
Acat2	ENSMUSG00000023832	chr17	13135756	13153613	-	2544	-2.074	-1
Lrwd1	ENSMUSG00000029703	chr5	136598642	136611944	-	6650	-2.075	-1
Tmem55b	ENSMUSG00000035953	chr14	51545745	51550531	-	4489	-2.077	-1
Srprb	ENSMUSG00000032553	chr9	103090363	103104467	-	4231	-2.078	-1
Klhl25	ENSMUSG00000055652	chr7	82993224	83019016	+	3731	-2.079	-1
Mrpl52	ENSMUSG00000010406	chr14	55045746	55048587	+	412	-2.079	-1

Tbc1d22a	ENSMUSG00000051864	chr15	86044877	86328929	+	3253	-2.079	-1
Gm5576	ENSMUSG00000063692	chr6	78165849	78167290	+	1442	-2.08	-1
Ppil2	ENSMUSG00000022771	chr16	17086648	17111350	-	6173	-2.081	-1
Ssu72	ENSMUSG00000029038	chr4	155078909	155107988	+	2689	-2.081	-1
Rps3	ENSMUSG00000030744	chr7	106626406	106632248	-	4483	-2.081	-1
Krt19	ENSMUSG00000020911	chr11	100002124	100009979	-	1855	-2.081	-1
Sdhd	ENSMUSG00000000171	chr9	50404443	50411954	-	1287	-2.081	-1
Zfp579	ENSMUSG00000051550	chr7	4935085	4947760	-	4159	-2.082	-1
Rilp	ENSMUSG00000038195	chr11	75323596	75326670	+	1583	-2.083	-1
Hip1r	ENSMUSG00000000915	chr5	124423637	124453220	+	5022	-2.084	-1
Cog1	ENSMUSG00000018661	chr11	113510483	113528368	+	4996	-2.084	-1
Cntfr	ENSMUSG00000028444	chr4	41604529	41644121	-	2782	-2.085	-1
Ddb2	ENSMUSG00000002109	chr2	91051729	91077139	-	2736	-2.085	-1
Frs3	ENSMUSG00000023266	chr17	47825979	47841235	+	3132	-2.085	-1
Rab11fip4	ENSMUSG00000017639	chr11	79404714	79511525	+	7451	-2.085	-1
Bnip1	ENSMUSG00000024191	chr17	26918024	26929510	+	5097	-2.086	-1
Cirbp	ENSMUSG00000045193	chr10	79628730	79635531	+	2604	-2.087	-1
1110005A03Rik	ENSMUSG00000090266	chr11	116704592	116715577	+	2697	-2.087	-1
Rnpep	ENSMUSG00000041926	chr1	137159289	137180661	-	3299	-2.088	-1
Crtc1	ENSMUSG00000003575	chr8	72906254	72963478	-	11182	-2.088	-1
Nhej1	ENSMUSG00000026162	chr1	75013713	75093213	-	1053	-2.089	-1
Plbd2	ENSMUSG00000029598	chr5	120933291	120953634	-	6620	-2.09	-1
Flywch1	ENSMUSG00000040097	chr17	23889765	23908558	-	3575	-2.092	-1
Zfp296	ENSMUSG00000011267	chr7	20162636	20166005	+	1530	-2.092	-1
Cyb5	ENSMUSG00000024646	chr18	85020730	85049793	+	2143	-2.095	-1
Rpusd1	ENSMUSG00000041199	chr17	25864696	25868401	+	1703	-2.095	-1
Klf16	ENSMUSG00000035397	chr10	80029869	80040066	-	2674	-2.098	-1
Cda	ENSMUSG00000028755	chr4	137894339	137923907	-	1035	-2.098	-1
Psmd8	ENSMUSG00000030591	chr7	29959207	29965692	-	1512	-2.099	-1
Ubox5	ENSMUSG00000027300	chr2	130415738	130455774	-	5585	-2.099	-1
Anapc2	ENSMUSG00000026965	chr2	25127998	25141435	+	3445	-2.099	-1
Arfgap3	ENSMUSG00000054277	chr15	83130170	83180677	-	2618	-2.1	-1
Sigirr	ENSMUSG00000025494	chr7	148277074	148286445	-	1990	-2.1	-1
Traf2	ENSMUSG00000026942	chr2	25373502	25402460	-	5101	-2.104	-1
Fam58b	ENSMUSG00000049489	chr11	78564009	78565231	-	1223	-2.104	-1
Ciapin1	ENSMUSG00000031781	chr8	97343718	97362258	-	5691	-2.106	-1
H2-Ke2	ENSMUSG00000024309	chr17	34075766	34077288	-	1193	-2.107	-1
Scamp4	ENSMUSG00000078441	chr10	80065627	80078528	+	1802	-2.107	-1
Aldoc	ENSMUSG00000017390	chr11	78136470	78141283	+	3263	-2.108	-1
Gba	ENSMUSG00000028048	chr3	89006850	89012603	+	1950	-2.109	-1

Tmbim4	ENSMUSG00000020225	chr10	119638646	119661973	+	2581	-2.109	-1
Zfp219	ENSMUSG00000049295	chr14	52625752	52640408	-	3117	-2.111	-1
Prpf19	ENSMUSG00000024735	chr19	10969733	10980019	+	2176	-2.113	-1
2810002 N01Rik	ENSMUSG00000037787	chr12	112951472	112993191	+	3945	-2.113	-1
Rnf166	ENSMUSG00000014470	chr8	124990047	124999964	-	1985	-2.113	-1
Tbx3	ENSMUSG00000018604	chr5	120120678	120134733	+	5365	-2.113	-1
Rasgrp2	ENSMUSG00000032946	chr19	6399340	6415216	+	12384	-2.114	-1
Rras	ENSMUSG00000038387	chr7	52273348	52277016	+	1103	-2.115	-1
Alkbh4	ENSMUSG00000039754	chr5	136612016	136617485	+	2117	-2.115	-1
Hsf4	ENSMUSG00000033249	chr8	107793701	107799745	+	1753	-2.117	-1
Gpd1	ENSMUSG00000023019	chr15	99547946	99555436	+	2961	-2.118	-1
Mesdc1	ENSMUSG00000070462	chr7	91029005	91032851	-	3847	-2.119	-1
Tuba1c	ENSMUSG00000043091	chr15	98860322	98868536	+	1977	-2.119	-1
E13030 3B06Rik	ENSMUSG00000013155	chr8	108227555	108231833	-	1272	-2.121	-1
Neurl2	ENSMUSG00000039873	chr2	164656232	164658956	-	1014	-2.121	-1
Sirt4	ENSMUSG00000029524	chr5	115928019	115934734	-	2398	-2.121	-1
Klhdc4	ENSMUSG00000040263	chr8	124320213	124353469	-	3708	-2.122	-1
Zbtb45	ENSMUSG00000049600	chr7	13590285	13595171	-	2722	-2.123	-1
Scfd2	ENSMUSG00000062110	chr5	74600841	74927772	-	8127	-2.124	-1
Gm2000	ENSMUSG00000078193	chr1	158296169	158296613	+	445	-2.125	-1
Ube2s	ENSMUSG00000060860	chr7	4759616	4763942	-	988	-2.126	-1
Snx33	ENSMUSG00000032733	chr9	56765007	56776178	-	4564	-2.126	-1
Pelo	ENSMUSG00000042275	chr13	115878565	115880396	-	1600	-2.127	-1
1110038 D17Rik	ENSMUSG00000033416	chr10	74813856	74980717	-	8206	-2.127	-1
Rabepk	ENSMUSG00000070953	chr2	34633076	34655432	-	3814	-2.128	-1
Psmc3	ENSMUSG00000002102	chr2	90894166	90906526	+	2607	-2.129	-1
6720456 B07Rik	ENSMUSG00000033940	chr6	113554766	113566942	+	1092	-2.129	-1
Tsr2	ENSMUSG00000025264	chrX	147521637	147531086	-	3805	-2.129	-1
Nfkbib	ENSMUSG00000030595	chr7	29543270	29552531	-	2169	-2.13	-1
Cep68	ENSMUSG00000044066	chr11	20127040	20149432	-	5865	-2.131	-1
Atad3a	ENSMUSG00000029036	chr4	155114750	155135207	-	3770	-2.131	-1
Rps15a	ENSMUSG00000008683	chr7	125247892	125259700	-	6262	-2.136	-1
Xrcc1	ENSMUSG00000051768	chr7	25332169	25358457	+	2155	-2.136	-1
Rnf5	ENSMUSG00000015478	chr17	34738036	34740635	-	1483	-2.136	-1
Dpagt1	ENSMUSG00000032123	chr9	44134928	44141683	+	1905	-2.137	-1
1110038 F14Rik	ENSMUSG00000063236	chr15	76778969	76781159	+	1010	-2.138	-1
Mad2l2	ENSMUSG00000029003	chr4	147504493	147519808	+	2622	-2.138	-1

Smoc1	ENSMUSG00000021136	chr12	82127795	82287401	+	3496	-2.139	-1
Trub2	ENSMUSG00000039826	chr2	29616744	29644022	-	6316	-2.14	-1
Tmem8	ENSMUSG00000024180	chr17	26250261	26260199	+	3464	-2.14	-1
Ift172	ENSMUSG00000038564	chr5	31555653	31593487	-	5407	-2.141	-1
Grpel1	ENSMUSG00000029198	chr5	36807647	36816729	+	3528	-2.143	-1
Neil1	ENSMUSG00000032298	chr9	56990607	56995929	-	1688	-2.144	-1
E130317F20Rik	ENSMUSG00000091994	chr10	79314126	79317701	-	3265	-2.144	-1
Fdft1	ENSMUSG00000021273	chr14	63763990	63796630	-	2860	-2.144	-1
Zfp637	ENSMUSG00000059689	chr6	117791198	117795974	+	2565	-2.145	-1
Cdc42bpg	ENSMUSG00000024769	chr19	6306456	6325652	+	6099	-2.146	-1
Ctdsp1	ENSMUSG00000026176	chr1	74438083	74443859	+	3334	-2.146	-1
Tpra1	ENSMUSG00000002871	chr6	88852245	88862232	+	3026	-2.146	-1
Csk	ENSMUSG00000032312	chr9	57474454	57493460	-	2748	-2.147	-1
Srpx	ENSMUSG00000090084	chrX	9615103	9694835	-	2652	-2.147	-1
Vamp2	ENSMUSG00000020894	chr11	68901992	68905886	+	3809	-2.148	-1
2310045N01Rik	ENSMUSG00000002345	chr8	72663398	72691387	+	2810	-2.149	-1
Sfxn5	ENSMUSG00000033720	chr6	85163045	85283416	-	5743	-2.149	-1
Lman2l	ENSMUSG00000001143	chr1	36478910	36502116	-	5995	-2.149	-1
Fhl1	ENSMUSG00000023092	[illegible]	53984964	54046523	+	3722	-2.149	-1
Ptprcap	ENSMUSG00000045826	chr19	4154606	4156751	+	927	-2.152	-1
Med20	ENSMUSG00000092558	chr17	47748531	47787238	+	7664	-2.152	-1
Gemin7	ENSMUSG00000044709	chr7	20150295	20159172	-	1519	-2.152	-1
Psmb1	ENSMUSG00000014769	chr17	15612685	15635240	-	1036	-2.153	-1
Igsf5	ENSMUSG00000000159	chr16	96583275	96747187	+	2766	-2.155	-1
Apex1	ENSMUSG00000035960	chr14	51544643	51546814	+	1422	-2.156	-1
Rad51l3	ENSMUSG00000018841	chr11	82685466	82704116	-	4598	-2.157	-1
Mrvi1	ENSMUSG00000005611	chr7	118011780	118125975	-	6827	-2.157	-1
Commd4	ENSMUSG00000032299	chr9	57002848	57006106	-	824	-2.159	-1
Ccdc124	ENSMUSG00000007721	chr8	73392126	73397389	-	911	-2.16	-1
Dio3os	ENSMUSG00000090962	chr12	111513594	111516278	-	1281	-2.161	-1
Commd2	ENSMUSG00000036513	chr3	57446826	57455637	-	4757	-2.162	-1
Mical1	ENSMUSG00000019823	chr10	41196120	41206838	+	6047	-2.162	-1
Eif5a	ENSMUSG00000078812	chr11	69730216	69735460	-	2377	-2.163	-1
Hbxip	ENSMUSG00000087260	chr3	107081776	107086997	+	881	-2.163	-1
Rpp21	ENSMUSG00000024446	chr17	36392590	36395014	-	1218	-2.163	-1
Aqp5	ENSMUSG00000044217	chr15	99421280	99425260	+	1667	-2.164	-1
Ipo4	ENSMUSG00000002319	chr14	56244237	56256732	-	5362	-2.166	-1
Pcif1	ENSMUSG00000039849	chr2	164704804	164719954	+	6544	-2.167	-1
Ddr1	ENSMUSG00000003534	chr17	35818512	35841566	-	4774	-2.167	-1
Leprel4	ENSMUSG00000006931	chr11	100269770	100276150	-	2178	-2.167	-1

Hist1h4h	ENSMUSG00000060981	chr13	23622919	23623388	+	470	-2.169	-1
Mks1	ENSMUSG00000034121	chr11	87666717	87677305	+	4044	-2.169	-1
2400009B08Rik	ENSMUSG00000089665	chr8	3671188	3672336	-	366	-2.17	-1
Psmd3	ENSMUSG00000017221	chr11	98543868	98557293	+	4967	-2.17	-1
Echs1	ENSMUSG00000025465	chr7	147291609	147302375	-	5274	-2.172	-1
Deb1	ENSMUSG00000032526	chr9	121619507	121622039	+	684	-2.173	-1
Smyd4	ENSMUSG00000018809	chr11	75161935	75219207	+	4329	-2.174	-1
Arfrp1	ENSMUSG00000038671	chr2	181092395	181100109	-	4320	-2.175	-1
Tesk1	ENSMUSG00000028458	chr4	43454811	43460936	+	4434	-2.176	-1
Ccrl1	ENSMUSG00000079355	chr9	104000468	104028973	-	2109	-2.177	-1
Cyb561d1	ENSMUSG00000048796	chr3	107998605	108004130	-	5086	-2.178	-1
Stk25	ENSMUSG00000026277	chr1	95518479	95532299	-	3397	-2.179	-1
Med22	ENSMUSG00000015776	chr2	26760782	26766197	-	3614	-2.179	-1
Mettl7a1	ENSMUSG00000054619	chr15	100135248	100144782	+	1886	-2.18	-1
Mrpl36	ENSMUSG00000021607	chr13	73468457	73469623	+	899	-2.181	-1
Vav2	ENSMUSG00000009621	chr2	27117624	27282553	-	6915	-2.182	-1
Gm10063	ENSMUSG00000059333	chr8	37088807	37089190	+	384	-2.183	-1
Chchd7	ENSMUSG00000042198	chr4	3866035	3878193	+	4060	-2.183	-1
Exosc7	ENSMUSG00000025785	chr9	123022333	123045247	+	1843	-2.184	-1
Sumo3	ENSMUSG00000020265	chr10	77068842	77104288	+	3826	-2.185	-1
Pkdcc	ENSMUSG00000024247	chr17	83614623	83624410	+	2721	-2.187	-1
Trim27	ENSMUSG00000021326	chr13	21271314	21286593	+	4702	-2.188	-1
Chi3l1	ENSMUSG00000064246	chr1	136078753	136086758	+	2804	-2.188	-1
Ubac2	ENSMUSG00000041765	chr14	122277828	122420256	+	2153	-2.189	-1
Zfp628	ENSMUSG00000074406	chr7	4866819	4873605	+	3524	-2.191	-1
Rrp7a	ENSMUSG00000018040	chr15	82943863	82953231	-	5411	-2.192	-1
Asap3	ENSMUSG00000036995	chr4	135762280	135801131	+	4225	-2.192	-1
Slc9a3r1	ENSMUSG00000020733	chr11	115024655	115042495	+	1925	-2.193	-1
Dhrs7b	ENSMUSG00000042569	chr11	60644133	60673697	+	3325	-2.194	-1
Stoml2	ENSMUSG00000028455	chr4	43040562	43044582	-	3022	-2.194	-1
Mbtps1	ENSMUSG00000031835	chr8	122032056	122082661	-	6799	-2.194	-1
Ephb6	ENSMUSG00000029869	chr6	41555481	41570508	+	3773	-2.196	-1
Vrk3	ENSMUSG00000002205	chr7	52003783	52032885	+	3098	-2.198	-1
Epb4.9	ENSMUSG00000022099	chr14	71001070	71035785	-	5473	-2.201	-1
Lrrc45	ENSMUSG00000025145	chr11	120575239	120582442	+	3293	-2.201	-1
Sesn2	ENSMUSG00000028893	chr4	132047947	132066416	-	3475	-2.202	-1
Zfp687	ENSMUSG00000019338	chr3	94810512	94819370	-	5952	-2.203	-1
Polr3h	ENSMUSG00000022476	chr15	81745460	81756643	-	2514	-2.206	-1
Cox4nb	ENSMUSG00000031819	chr8	123177814	123192012	-	4933	-2.206	-1
Mfsd5	ENSMUSG00000045665	chr15	102109887	102112175	+	1742	-2.207	-1
Trim28	ENSMUSG00000005566	chr7	13609501	13616384	+	4044	-2.207	-1

Drg1	ENSMUSG00000020457	chr11	3087363	3166418	-	3460	-2.208	-1
Ppp1r12c	ENSMUSG00000019254	chr7	4433122	4453282	-	4992	-2.208	-1
Tead3	ENSMUSG00000002249	chr17	28468616	28487750	-	4398	-2.209	-1
Nbl1	ENSMUSG00000041120	chr4	138638199	138648908	-	2033	-2.21	-1
Det1	ENSMUSG00000030610	chr7	85967848	85992122	-	2877	-2.21	-1
Map3k11	ENSMUSG00000004054	chr19	5689131	5702862	+	3801	-2.21	-1
Gm11974	ENSMUSG00000085156	chr11	6425594	6428763	-	1359	-2.212	-1
2610034B18Rik	ENSMUSG00000039043	chr7	87070247	87080245	-	2277	-2.212	-1
Ltbp4	ENSMUSG00000040488	chr7	28090155	28122711	-	6463	-2.213	-1
Gsk3a	ENSMUSG00000057177	chr7	26013277	26022870	-	2260	-2.213	-1
3010026O09Rik	ENSMUSG00000020381	chr11	49987946	50013617	+	2039	-2.214	-1
H2afx	ENSMUSG00000049932	chr9	44142798	44144154	+	1357	-2.214	-1
1500011K16Rik	ENSMUSG00000051319	chr2	127617124	127618224	-	800	-2.215	-1
Ppdpf	ENSMUSG00000016344	chr2	180921952	180923476	+	1336	-2.215	-1
Amacr	ENSMUSG00000022244	chr15	10911511	10926379	+	2575	-2.216	-1
Adamts8	ENSMUSG00000031994	chr9	30750147	30771423	+	4613	-2.217	-1
Ltbr	ENSMUSG00000030339	chr6	125256589	125263903	-	2354	-2.217	-1
Thap11	ENSMUSG00000036442	chr8	108379003	108380850	+	1848	-2.217	-1
Mrpl15	ENSMUSG00000033845	chr1	4763287	4775820	-	5668	-2.217	-1
1810058I24Rik	ENSMUSG00000073155	chr6	35202654	35213496	+	2784	-2.218	-1
Ciao1	ENSMUSG00000003662	chr2	127066674	127073552	-	3197	-2.219	-1
Rnd2	ENSMUSG00000001313	chr11	101326313	101333167	+	1990	-2.221	-1
Sqrdl	ENSMUSG00000005803	chr2	122590973	122635305	+	4084	-2.222	-1
Pigr	ENSMUSG00000026417	chr1	132723261	132748826	+	4025	-2.223	-1
Dolpp1	ENSMUSG00000026856	chr2	30247774	30256049	+	3233	-2.224	-1
Psmb8	ENSMUSG00000024338	chr17	34334666	34338399	+	2736	-2.226	-1
Sowahb	ENSMUSG00000045314	chr5	93470149	93474048	-	3900	-2.226	-1
Mepce	ENSMUSG00000029726	chr5	138223134	138227943	-	3292	-2.227	-1
D930014E17Rik	ENSMUSG00000031023	chr7	116847204	116855703	+	1305	-2.228	-1
Apod	ENSMUSG00000022548	chr16	31296278	31314894	-	2577	-2.228	-1
Tfb1m	ENSMUSG00000036983	chr17	3519263	3557713	-	1327	-2.23	-1
Klc3	ENSMUSG00000040714	chr7	19979786	19989453	-	2619	-2.231	-1
Zfp865	ENSMUSG00000074405	chr7	4971978	4984740	+	3873	-2.231	-1
Adi1	ENSMUSG00000020629	chr12	29360096	29367040	+	1886	-2.231	-1
Ocln	ENSMUSG00000021638	chr13	101266462	101322673	-	4222	-2.231	-1
Gm13889	ENSMUSG00000087006	chr2	93795967	93797684	-	1382	-2.232	-1

Slx1b	ENSMUSG00000059772	chr7	133832982	133839298	-	3521	-2.233		-1
Wdr91	ENSMUSG00000058486	chr6	34830426	34860876	-	2990	-2.233		-1
Pppde2	ENSMUSG00000022472	chr15	81822953	81864292	-	3635	-2.235		-1
Trmt12	ENSMUSG00000037085	chr15	58704204	58708336	+	4133	-2.236		-1
Trib3	ENSMUSG00000032715	chr2	152163158	152169768	-	2125	-2.237		-1
Xylb	ENSMUSG00000035769	chr9	119266499	119302438	+	3505	-2.237		-1
Synpo2	ENSMUSG00000050315	chr3	122779437	122939067	-	9403	-2.239		-1
Sdr39u1	ENSMUSG00000022223	chr14	56516123	56519069	-	1226	-2.239		-1
B230208H17Rik	ENSMUSG00000015087	chr2	25438538	25464041	-	6379	-2.24		-1
C030046I01Rik	ENSMUSG00000035781	chr10	79372798	79379751	-	1897	-2.24		-1
H2-Ab1	ENSMUSG00000073421	chr17	34400154	34406363	+	1644	-2.241		-1
Fzr1	ENSMUSG00000020235	chr10	80828967	80841261	-	4488	-2.241		-1
Hes1	ENSMUSG00000022528	chr16	30064470	30067882	+	2037	-2.243		-1
Lemd2	ENSMUSG00000044857	chr17	27326546	27341383	-	2595	-2.243		-1
Rnaseh2a	ENSMUSG00000052926	chr8	87480509	87493666	-	3509	-2.243		-1
Vamp5	ENSMUSG00000073002	chr6	72318043	72330462	-	1648	-2.244		-1
Mrpl49	ENSMUSG00000007338	chr19	6053630	6057758	-	1729	-2.244		-1
9530082P21Rik	ENSMUSG00000086281	chr17	23880201	23891032	+	4842	-2.244		-1
2410001C21Rik	ENSMUSG00000027502	chr2	172266056	172295408	+	2644	-2.245		-1
1700011J10Rik	ENSMUSG00000063714	chr2	72817481	72826734	+	892	-2.247		-1
Nadsyn1	ENSMUSG00000031090	chr7	150981489	151008754	-	3306	-2.247		-1
Csnk2b	ENSMUSG00000024387	chr17	35253141	35258998	-	2164	-2.248		-1
Ptplb	ENSMUSG00000035376	chr16	35022507	35109261	+	3603	-2.248		-1
Wbscr22	ENSMUSG00000005378	chr5	135528827	135540829	-	3386	-2.248		-1
Rsph3b	ENSMUSG00000023806	chr17	7109030	7153070	-	2014	-2.25		-1
Trim62	ENSMUSG00000041000	chr4	128560824	128588572	+	5015	-2.251		-1
Hebp1	ENSMUSG00000042770	chr6	135087537	135118233	-	1061	-2.253		-1
Fmod	ENSMUSG00000041559	chr1	135933831	135944854	+	3306	-2.253		-1
Reep5	ENSMUSG00000005873	chr18	34504543	34533069	-	2894	-2.255		-1
Fuca2	ENSMUSG00000019810	chr10	13219346	13238841	+	6161	-2.256		-1
Gm5428	ENSMUSG00000091086	chr10	110562907	110563794	+	888	-2.256		-1
Tmem176b	ENSMUSG00000029810	chr6	48783810	48791373	-	2129	-2.258		-1
4933421O10Rik	ENSMUSG00000085037	chr4	33114117	33118298	-	3847	-2.258		-1
Hexa	ENSMUSG00000025232	chr9	59387474	59412914	+	1869	-2.258		-1

Ctu1	ENSMUSG00000038888	chr7	50927386	50933668	+	2475	-2.26	-1
Ercc8	ENSMUSG00000021694	chr13	108948925	108985558	+	7657	-2.261	-1
Minos1	ENSMUSG00000050608	chr4	138657729	138687028	-	2766	-2.261	-1
Slc3a2	ENSMUSG00000010095	chr19	8781372	8797859	-	2958	-2.262	-1
Nudt3	ENSMUSG00000024213	chr17	27716327	27760410	-	2985	-2.263	-1
Def8	ENSMUSG00000001482	chr8	125966886	125987170	+	3988	-2.265	-1
Tcea2	ENSMUSG00000059540	chr2	181415015	181422776	+	2704	-2.267	-1
Prkcsh	ENSMUSG00000003402	chr9	21807479	21818663	+	2013	-2.268	-1
Coro1b	ENSMUSG00000024835	chr19	4148619	4154035	+	2372	-2.269	-1
Isca2	ENSMUSG00000021241	chr12	86114220	86116039	+	868	-2.271	-1
Mlph	ENSMUSG00000026303	chr1	92811662	92847719	+	5357	-2.272	-1
Bax	ENSMUSG00000003873	chr7	52717065	52722268	-	1339	-2.273	-1
Dusp22	ENSMUSG00000069255	chr13	30751868	30803100	+	1743	-2.274	-1
Map2k2	ENSMUSG00000035027	chr10	80568660	80587442	+	3722	-2.275	-1
Arrb2	ENSMUSG00000060216	chr11	70246137	70254330	+	2845	-2.276	-1
Gabarap	ENSMUSG00000018567	chr11	69804645	69808453	+	1844	-2.277	-1
Scap	ENSMUSG00000032485	chr9	110235792	110287450	+	5205	-2.277	-1
Cenpm	ENSMUSG00000068101	chr15	82064209	82075177	-	1201	-2.28	-1
1110003E01Rik	ENSMUSG00000037822	chr5	65838083	65928423	-	7460	-2.281	-1
Grwd1	ENSMUSG00000053801	chr7	53080597	53086314	-	2053	-2.281	-1
Dph3	ENSMUSG00000021905	chr14	32893752	32898878	-	3555	-2.282	-1
Hs1bp3	ENSMUSG00000020605	chr12	8320239	8350630	+	2958	-2.285	-1
Eefsec	ENSMUSG00000033216	chr6	88207328	88396533	-	2759	-2.287	-1
Rab4b	ENSMUSG00000053291	chr7	27953443	27963915	-	1970	-2.287	-1
Mxd3	ENSMUSG00000021485	chr13	55426529	55431119	-	2081	-2.288	-1
Trim45	ENSMUSG00000033233	chr3	100726125	100740848	+	6005	-2.289	-1
0610007C21Rik	ENSMUSG00000013622	chr5	31350685	31357006	+	2092	-2.291	-1
Tmem161a	ENSMUSG00000002342	chr8	72696255	72707580	+	3672	-2.291	-1
9030617O03Rik	ENSMUSG00000021185	chr12	102017267	102135191	+	4434	-2.292	-1
Zfp316	ENSMUSG00000046658	chr5	144011376	144031701	-	7532	-2.294	-1
Sec14l4	ENSMUSG00000019368	chr11	3931465	3948027	+	3487	-2.294	-1
Slc25a26	ENSMUSG00000045100	chr6	94450308	94554642	+	1915	-2.294	-1
Rpsa	ENSMUSG00000032518	chr9	120036884	120041487	+	1861	-2.295	-1
AU040320	ENSMUSG00000028830	chr4	126430788	126547314	+	6259	-2.297	-1
Neurl1a	ENSMUSG00000006435	chr19	47253310	47333930	+	4411	-2.298	-1
1500011B03Rik	ENSMUSG00000072694	chr5	115258205	115263985	-	1333	-2.298	-1

Usmg5	ENSMUSG00000071528	chr19	47157961	47165115	-	341	-2.299	-1
Alg12	ENSMUSG00000035845	chr15	88635674	88649748	-	2356	-2.301	-1
Pex26	ENSMUSG00000067825	chr6	121133685	121148855	+	5667	-2.301	-1
BC031181	ENSMUSG00000036299	chr18	75165554	75169587	+	1047	-2.301	-1
Gm16537	ENSMUSG00000089671	chr15	99545974	99554905	-	702	-2.302	-1
RP23-349B16.3	ENSMUSG00000093622	chr17	71184004	71185384	+	385	-2.302	-1
Polr2g	ENSMUSG00000071662	chr19	8867619	8873047	-	868	-2.302	-1
Tmem101	ENSMUSG00000020921	chr11	102013860	102017718	-	2021	-2.304	-1
Epcam	ENSMUSG00000045394	chr17	88035319	88050446	+	2040	-2.304	-1
Thap4	ENSMUSG00000026279	chr1	95601968	95651415	-	2172	-2.304	-1
Cpsf3l	ENSMUSG00000029034	chr4	155243655	155263212	+	3067	-2.305	-1
Trappc9	ENSMUSG00000047921	chr15	72420051	72891634	-	6377	-2.306	-1
Ptrh2	ENSMUSG00000072582	chr11	86497487	86505959	+	3132	-2.308	-1
Pigx	ENSMUSG00000023791	chr16	32084504	32099824	-	2394	-2.309	-1
Otub1	ENSMUSG00000024767	chr19	7272692	7280806	-	2545	-2.309	-1
Gstm5	ENSMUSG00000004032	chr3	107698739	107701604	+	1278	-2.309	-1
Shmt2	ENSMUSG00000025403	chr10	126954195	126959491	-	2280	-2.31	-1
Cd2bp2	ENSMUSG00000042502	chr7	134335174	134339514	-	3379	-2.311	-1
Hpgd	ENSMUSG00000031613	chr8	58773349	58799843	+	1719	-2.312	-1
Cox19	ENSMUSG00000045438	chr5	139812143	139821187	-	3539	-2.313	-1
Apbb1	ENSMUSG00000037032	chr7	112707000	112722916	-	2530	-2.313	-1
Gm16062	ENSMUSG00000087249	chr11	59623582	59631993	+	771	-2.314	-1
Puf60	ENSMUSG00000002524	chr15	75900614	75911376	-	1912	-2.317	-1
Tle6	ENSMUSG00000034758	chr10	81053649	81063818	-	3088	-2.318	-1
Mrps23	ENSMUSG00000023723	chr11	88017890	88025009	+	1560	-2.319	-1
Diablo	ENSMUSG00000029433	chr5	123959774	123974185	-	6155	-2.32	-1
Banf1	ENSMUSG00000024844	chr19	5364641	5366645	-	820	-2.321	-1
2810055F11Rik	ENSMUSG00000019718	chr12	73174415	73186426	-	2679	-2.321	-1
Slc30a6	ENSMUSG00000024069	chr17	74794972	74823569	+	2132	-2.323	-1
Erbb2	ENSMUSG00000062312	chr11	98273784	98299030	+	5210	-2.324	-1
Mfsd9	ENSMUSG00000041945	chr1	40828883	40847532	-	5125	-2.324	-1
Trafd1	ENSMUSG00000042726	chr5	121821734	121835641	-	4486	-2.324	-1
Pdxk	ENSMUSG00000032788	chr10	77899489	77927720	-	5124	-2.326	-1
Wdr73	ENSMUSG00000025722	chr7	88035609	88046155	-	3787	-2.326	-1
Gm10159	ENSMUSG00000069972	chr7	95678915	95679435	-	521	-2.326	-1
Gnb2	ENSMUSG00000029713	chr5	137969355	137974738	-	3402	-2.327	-1
Gm10020	ENSMUSG00000057262	chr15	52309169	52309783	-	615	-2.328	-1
Snx11	ENSMUSG00000020876	chr11	96628869	96638873	-	4232	-2.329	-1

Gramd1a	ENSMUSG00000001248	chr7	31915146	31936069	-	2683	-2.329	-1
Slc26a6	ENSMUSG00000023259	chr9	108756368	108764472	+	2081	-2.329	-1
Araf	ENSMUSG00000001127	chrX	20374940	20437645	+	6541	-2.332	-1
Copz1	ENSMUSG00000060992	chr15	103103342	103130293	+	2436	-2.332	-1
Nagpa	ENSMUSG00000023143	chr16	5195382	5204105	-	2370	-2.333	-1
Wdr62	ENSMUSG00000037020	chr7	31025157	31065438	-	5743	-2.333	-1
Ifi27l2a	ENSMUSG00000079017	chr12	104680377	104681890	-	463	-2.333	-1
Zdhhc14	ENSMUSG00000034265	chr17	5492600	5753891	+	3115	-2.334	-1
Dhrs4	ENSMUSG00000022210	chr14	56097595	56109175	+	1390	-2.335	-1
Gm5093	ENSMUSG00000091742	chr17	46576527	46577048	-	522	-2.336	-1
Zfp358	ENSMUSG00000047264	chr8	3493138	3497208	+	1999	-2.337	-1
Stx8	ENSMUSG00000020903	chr11	67779695	68020650	+	7137	-2.337	-1
Gm5918	ENSMUSG00000086158	chr9	72827531	72833183	-	709	-2.337	-1
Slc22a18	ENSMUSG00000000154	chr7	150659626	150685239	+	1718	-2.337	-1
Prelp	ENSMUSG00000041577	chr1	135806881	135817991	-	3615	-2.339	-1
Zfp653	ENSMUSG00000038895	chr9	21859904	21875789	-	2108	-2.34	-1
Pex5	ENSMUSG00000005069	chr6	124346834	124365085	-	3943	-2.341	-1
Selenof	ENSMUSG00000037072	chr3	144233268	144260644	+	2104	-2.343	-1
Pgpep1	ENSMUSG00000056204	chr8	73173010	73183580	-	2150	-2.347	-1
Sgsm3	ENSMUSG00000042303	chr15	80808195	80842719	+	4358	-2.349	-1
Lrrc27	ENSMUSG00000015980	chr7	146398887	146428878	+	3637	-2.349	-1
Atg13	ENSMUSG00000027244	chr2	91514775	91550733	-	4368	-2.35	-1
Fam132a	ENSMUSG00000023571	chr4	155336427	155340738	+	1366	-2.35	-1
Wbp5	ENSMUSG00000042712	chrX	132779618	132781678	+	1037	-2.352	-1
Ppp1r11	ENSMUSG00000036398	chr17	37085301	37088686	-	1975	-2.352	-1
Psmd13	ENSMUSG00000025487	chr7	148067867	148084542	+	3395	-2.353	-1
Rps6kb2	ENSMUSG00000024830	chr19	4156706	4163354	-	2452	-2.354	-1
Gm10094	ENSMUSG00000061104	chr8	98349254	98350033	+	780	-2.355	-1
Gm6635	ENSMUSG00000058607	chr19	30235110	30235487	+	378	-2.355	-1
Abcc3	ENSMUSG00000020865	chr11	94204612	94254311	-	5692	-2.355	-1
Suds3	ENSMUSG00000066900	chr5	117541691	117566002	-	2418	-2.356	-1
Unc119	ENSMUSG00000002058	chr11	78156984	78162666	+	2448	-2.358	-1
Ndufs2	ENSMUSG00000013593	chr1	173164984	173177253	-	2142	-2.359	-1
Rundc1	ENSMUSG00000035007	chr11	101286399	101296987	+	3223	-2.359	-1
Nr1h3	ENSMUSG00000002108	chr2	91024218	91042991	-	2515	-2.359	-1
Pdrg1	ENSMUSG00000027472	chr2	152834626	152841163	-	2443	-2.359	-1
Prdx2	ENSMUSG00000005161	chr8	87493486	87498733	+	3050	-2.36	-1
Wdr90	ENSMUSG00000073434	chr17	25981716	25998446	-	8466	-2.361	-1

Npepl1	ENSMUSG00000039263	chr2	173935850	173948571	+	2998	-2.362	-1
Tmem98	ENSMUSG00000035413	chr11	80623677	80635535	+	1633	-2.363	-1
Tuba4a	ENSMUSG00000026202	chr1	75211549	75215828	-	2053	-2.364	-1
Txnl4b	ENSMUSG00000031723	chr8	112089792	112097951	+	1890	-2.365	-1
Lynx1	ENSMUSG00000022594	chr15	74578286	74583409	-	3952	-2.367	-1
Adck1	ENSMUSG00000021044	chr12	89598998	89700163	+	2330	-2.367	-1
A2ld1	ENSMUSG00000041625	chr14	123215445	123312979	-	1988	-2.368	-1
Timm22	ENSMUSG00000020843	chr11	76220454	76229794	+	3183	-2.37	-1
2010317E24Rik	ENSMUSG00000026955	chr2	25227841	25233733	+	3499	-2.37	-1
Rpl38	ENSMUSG00000057322	chr11	114529838	114533645	+	542	-2.371	-1
Vps37d	ENSMUSG00000043614	chr5	135548770	135554136	-	1544	-2.371	-1
Cdk5	ENSMUSG00000028969	chr5	23924068	23929348	-	2050	-2.375	-1
4632415K11Rik	ENSMUSG00000034105	chr8	122284487	122302316	-	2574	-2.377	-1
Gm561	ENSMUSG00000074754	chr2	144419790	144421102	+	471	-2.377	-1
Mfsd2a	ENSMUSG00000028655	chr4	122624093	122638431	-	2250	-2.379	-1
Thtpa	ENSMUSG00000045691	chr14	55713672	55717823	+	2779	-2.379	-1
Tmem66	ENSMUSG00000031532	chr8	35217617	35233899	+	1897	-2.379	-1
Atp5g1	ENSMUSG00000006057	chr11	95930166	95936984	-	4908	-2.381	-1
Capn1	ENSMUSG00000024942	chr19	5988546	6015825	-	3190	-2.382	-1
Kdm4b	ENSMUSG00000024201	chr17	56465485	56542293	+	4895	-2.382	-1
Gga1	ENSMUSG00000033128	chr15	78707620	78725015	+	3034	-2.382	-1
5330430P22Rik	ENSMUSG00000085255	chr11	105926391	105927619	-	644	-2.382	-1
Irf9	ENSMUSG00000002325	chr14	56222408	56228867	+	5386	-2.383	-1
Eif2b5	ENSMUSG00000003235	chr16	20498890	20510028	+	3209	-2.386	-1
Rps9	ENSMUSG00000006333	chr7	3655595	3658503	+	2526	-2.386	-1
Orai1	ENSMUSG00000049686	chr5	123465083	123480459	+	2289	-2.388	-1
Ppp4c	ENSMUSG00000030697	chr7	133929421	133935985	-	1293	-2.388	-1
Mzt2	ENSMUSG00000022671	chr16	15848534	15863462	-	1236	-2.388	-1
Slc48a1	ENSMUSG00000081534	chr15	97614786	97623123	+	2690	-2.388	-1
Tmem179b	ENSMUSG00000079437	chr19	8847012	8848957	-	848	-2.39	-1
Ehmt2	ENSMUSG00000013787	chr17	35035414	35050997	+	5489	-2.391	-1
Nxn	ENSMUSG00000020844	chr11	76070700	76212642	-	3239	-2.391	-1
Eif2d	ENSMUSG00000026427	chr1	133049758	133084235	+	7370	-2.391	-1
Wdr85	ENSMUSG00000026975	chr2	24817920	24827683	+	2365	-2.391	-1
Hspb8	ENSMUSG00000041548	chr5	116858500	116872873	-	1950	-2.394	-1
Tradd	ENSMUSG00000031887	chr8	107782186	107788509	-	1870	-2.397	-1
Gtpbp6	ENSMUSG00000033434	chr5	110532994	110537216	-	2825	-2.397	-1
Ftl1	ENSMUSG00000050708	chr7	52713314	52715254	-	1340	-2.399	-1
Rab7l1	ENSMUSG00000026433	chr1	133763801	133769464	+	1350	-2.399	-1

Gpr56	ENSMUSG00000031785	chr8	97508503	97538090	+	3333	-2.399		-1
Cldn23	ENSMUSG00000055976	chr8	36887766	36889613	-	1848	-2.401		-1
Tmem176a	ENSMUSG00000023367	chr6	48791482	48795363	+	1119	-2.401		-1
Gtf2h4	ENSMUSG00000001524	chr17	35804675	35810684	-	3313	-2.404		-1
Gamt	ENSMUSG00000020150	chr10	79720896	79723757	-	1444	-2.404		-1
Pacsin3	ENSMUSG00000027257	chr2	91096111	91104836	+	4327	-2.405		-1
Chpf	ENSMUSG00000032997	chr1	75471144	75476046	-	4020	-2.406		-1
6330403K07Rik	ENSMUSG00000018451	chr11	70845451	70847015	-	1486	-2.407		-1
Sox12	ENSMUSG00000051817	chr2	152219347	152223782	-	4172	-2.408		-1
Slc27a1	ENSMUSG00000031808	chr8	74092826	74110607	+	2795	-2.408		-1
Iqsec2	ENSMUSG00000041115	chrX	148578811	148659779	+	6980	-2.408		-1
Acaa2	ENSMUSG00000036880	chr18	74938851	74965861	+	1515	-2.408		-1
Iscu	ENSMUSG00000025825	chr5	114222757	114228297	+	2176	-2.409		-1
Rab20	ENSMUSG00000031504	chr8	11453392	11478638	-	1446	-2.409		-1
Os9	ENSMUSG00000040462	chr10	126531315	126558216	-	4049	-2.41		-1
Ptgr1	ENSMUSG00000028378	chr4	58978311	58999991	-	2296	-2.411		-1
2310036O22Rik	ENSMUSG00000041203	chr8	87550732	87554184	+	891	-2.412		-1
Fam131a	ENSMUSG00000050821	chr16	20693355	20703121	+	2853	-2.415		-1
Spns1	ENSMUSG00000030741	chr7	133513574	133520964	-	3707	-2.415		-1
Pmf1	ENSMUSG00000028066	chr3	88198061	88214238	-	996	-2.415		-1
Hfe	ENSMUSG00000006611	chr13	23793903	23802723	-	3572	-2.415		-1
Rpl28	ENSMUSG00000030432	chr7	4744476	4746164	+	1138	-2.415		-1
Rem1	ENSMUSG00000000359	chr2	152452687	152460934	+	2156	-2.417		-1
Mrpl43	ENSMUSG00000025208	chr19	45079504	45080932	-	1213	-2.417		-1
Tspyl4	ENSMUSG00000039485	chr10	34017227	34021114	+	3888	-2.419		-1
Alpl	ENSMUSG00000028766	chr4	137297648	137352299	-	4033	-2.42		-1
Aup1	ENSMUSG00000068328	chr6	83004647	83007674	+	1509	-2.421		-1
2410002I01Rik	ENSMUSG00000025377	chr11	119951845	119960045	-	3329	-2.422		-1
Gm4189	ENSMUSG00000092384	chr10	126726752	126727844	-	431	-2.423		-1
Abcb8	ENSMUSG00000028973	chr5	23899481	23915872	+	4739	-2.423		-1
Tmem146	ENSMUSG00000040828	chr17	56767566	56803879	+	2565	-2.423		-1
Gmfg	ENSMUSG00000060791	chr7	29222466	29233252	+	2817	-2.424		-1
Adc	ENSMUSG00000028789	chr4	128607477	128639686	-	4024	-2.426		-1
5730437N04Rik	ENSMUSG00000004945	chr17	5410864	5440260	-	1004	-2.427		-1
Psmd9	ENSMUSG00000029440	chr5	123678199	123700140	+	4562	-2.427		-1
Esrp2	ENSMUSG00000084128	chr8	108654219	108660874	-	4340	-2.428		-1

C330006K01Rik	ENSMUSG00000039623	chr5	142939885	142954669	+	3842	-2.428	-1
Rnf187	ENSMUSG00000020496	chr11	58745790	58752418	-	1949	-2.43	-1
Cyb561	ENSMUSG00000019590	chr11	105795016	105814650	-	3653	-2.431	-1
Eral1	ENSMUSG00000020832	chr11	77886878	77893885	-	2268	-2.432	-1
2610027L16Rik	ENSMUSG00000019297	chr14	56364530	56374337	+	3694	-2.433	-1
Pdlim2	ENSMUSG00000022090	chr14	70564025	70577488	-	4486	-2.434	-1
Stoml1	ENSMUSG00000032333	chr9	58100971	58110330	+	1987	-2.434	-1
Pskh1	ENSMUSG00000048310	chr8	108424341	108455678	+	3255	-2.436	-1
Ndufa4	ENSMUSG00000029632	chr6	11850373	11857446	-	492	-2.437	-1
Slc38a7	ENSMUSG00000036534	chr8	98359820	98377439	-	4144	-2.439	-1
Gnb5	ENSMUSG00000032192	chr9	75159202	75193678	+	2345	-2.439	-1
Snapc2	ENSMUSG00000011837	chr8	4253102	4256219	+	1513	-2.44	-1
Gm11744	ENSMUSG00000075410	chr11	116514848	116529703	+	1520	-2.441	-1
Tmem216	ENSMUSG00000024667	chr19	10608355	10630728	-	1977	-2.444	-1
Alg5	ENSMUSG00000036632	chr3	54539461	54555049	+	3537	-2.446	-1
Eif2b2	ENSMUSG00000004788	chr12	86560431	86567578	+	1885	-2.446	-1
Tarbp2	ENSMUSG00000023051	chr15	102348623	102354107	+	4549	-2.447	-1
Arl5c	ENSMUSG00000038352	chr11	97850892	97857495	-	2073	-2.449	-1
Mrpl44	ENSMUSG00000026248	chr1	79772593	79778020	+	1424	-2.45	-1
C530028O21Rik	ENSMUSG00000030329	chr6	124946712	124953114	+	2753	-2.45	-1
Dvl1	ENSMUSG00000029071	chr4	155221511	155233412	+	4996	-2.45	-1
Rpn1	ENSMUSG00000030062	chr6	88034467	88055298	+	3642	-2.452	-1
Mrpl9	ENSMUSG00000028140	chr3	94247240	94255052	+	2703	-2.452	-1
Dusp8	ENSMUSG00000037887	chr7	149265395	149281748	-	4874	-2.453	-1
Mrpl46	ENSMUSG00000030612	chr7	85920122	85928417	-	1392	-2.454	-1
Epn1	ENSMUSG00000035203	chr7	5031837	5049780	+	2810	-2.454	-1
Tmed4	ENSMUSG00000004394	chr11	6170372	6174873	-	2178	-2.456	-1
Mettl13	ENSMUSG00000026694	chr1	164462258	164478682	-	9107	-2.456	-1
2310047B19Rik	ENSMUSG00000051238	chr9	21760197	21762714	+	1846	-2.457	-1
Srpr	ENSMUSG00000032042	chr9	35007760	35055558	+	3364	-2.459	-1
Atp5c1	ENSMUSG00000025781	chr2	9977643	10002137	-	1908	-2.459	-1
Suox	ENSMUSG00000049858	chr10	128106943	128110974	-	2349	-2.461	-1
3110056O03Rik	ENSMUSG00000035206	chr10	80318020	80331450	+	3605	-2.462	-1
B4galt3	ENSMUSG00000052423	chr1	173200459	173207027	+	5467	-2.465	-1
Stim1	ENSMUSG00000030987	chr7	109416338	109585362	+	3597	-2.465	-1
Cyp46a1	ENSMUSG00000021259	chr12	109572587	109600441	+	2119	-2.465	-1

Gm15706	ENSMUSG00000086013	chr6	145199049	145200369	+	899	-2.465		-1
Vps37c	ENSMUSG00000048832	chr19	10763305	10788909	+	2415	-2.467		-1
Pcbp3	ENSMUSG00000001120	chr10	76224602	76424632	-	2933	-2.468		-1
ORF19	ENSMUSG00000024101	chr17	66460886	66469843	+	2986	-2.468		-1
Gm12454	ENSMUSG00000087142	chr4	43517300	43521619	-	1082	-2.468		-1
Tmem93	ENSMUSG00000047260	chr11	72989021	72990539	-	1415	-2.469		-1
Acot6	ENSMUSG00000043487	chr12	85441604	85452299	+	3388	-2.469		-1
Slc37a4	ENSMUSG00000032114	chr9	44205182	44211046	+	2431	-2.471		-1
Ric8	ENSMUSG00000025485	chr7	148042856	148049629	+	4742	-2.473		-1
Gm6404	ENSMUSG00000061669	chr13	116943973	116944380	+	408	-2.473		-1
Dlk2	ENSMUSG00000047428	chr17	46434370	46440220	+	2113	-2.473		-1
Nt5c	ENSMUSG00000020736	chr11	115351734	115353176	-	1140	-2.474		-1
Gm10247	ENSMUSG00000068674	chr16	8922150	8922482	-	333	-2.475		-1
Med9	ENSMUSG00000061650	chr11	59761708	59775707	+	1920	-2.476		-1
4930481B07Rik	ENSMUSG00000085956	chr3	94819547	94824060	+	1490	-2.477		-1
Tmem204	ENSMUSG00000024168	chr17	25194647	25218126	-	2022	-2.477		-1
Tbc1d22b	ENSMUSG00000042203	chr17	29686733	29743840	+	4139	-2.478		-1
2010011I20Rik	ENSMUSG00000027495	chr2	172171065	172181249	+	3617	-2.478		-1
1110008P14Rik	ENSMUSG00000039195	chr2	32232617	32237458	-	2694	-2.478		-1
Pi4kb	ENSMUSG00000038861	chr3	94778653	94810765	+	3880	-2.479		-1
Rcl1	ENSMUSG00000024785	chr19	29175865	29218333	+	1803	-2.479		-1
Pak4	ENSMUSG00000030602	chr7	29343838	29383204	-	3545	-2.48		-1
Thra	ENSMUSG00000058756	chr11	98601952	98630320	+	4614	-2.481		-1
Nkiras2	ENSMUSG00000017837	chr11	100480558	100488921	+	3578	-2.481		-1
Paip2b	ENSMUSG00000045896	chr6	83755395	83781735	-	6157	-2.481		-1
Cops6	ENSMUSG00000019494	chr5	138602299	138605874	+	2525	-2.481		-1
Gm12060	ENSMUSG00000085415	chr11	23458058	23458842	-	785	-2.481		-1
Rpsa-ps10	ENSMUSG00000047676	chr3	149735554	149736584	-	1031	-2.482		-1
Atp5g3	ENSMUSG00000018770	chr2	73746504	73749383	-	2181	-2.482		-1
Rpn2	ENSMUSG00000027642	chr2	157104753	157152055	+	4430	-2.483		-1
Mmaa	ENSMUSG00000037022	chr8	81790323	81818855	-	2843	-2.484		-1
Fam96b	ENSMUSG00000031879	chr8	107163739	107165865	-	1075	-2.484		-1
H2-D1	ENSMUSG00000073411	chr17	35399675	35404444	+	2588	-2.485		-1
Cenpb	ENSMUSG00000068267	chr2	131000918	131005803	-	4886	-2.487		-1

Il18	ENSMUSG00000039217	chr9	50383378	50389942	+	702	-2.487	-1
Tkt	ENSMUSG00000021957	chr14	31361545	31387908	+	6533	-2.487	-1
Tmem109	ENSMUSG00000034659	chr19	10945150	10956491	-	3798	-2.489	-1
Esrra	ENSMUSG00000024955	chr19	6985467	6996303	-	2539	-2.49	-1
Ngrn	ENSMUSG00000047084	chr7	87406101	87410264	+	1718	-2.49	-1
Lsm10	ENSMUSG00000050188	chr4	125773867	125775828	+	943	-2.495	-1
Ints5	ENSMUSG00000071652	chr19	8967477	8972380	+	3294	-2.497	-1
Usf1	ENSMUSG00000026641	chr1	173341444	173349273	+	2806	-2.497	-1
Stx3	ENSMUSG00000041488	chr19	11849609	11893893	-	4979	-2.499	-1
Hspb2	ENSMUSG00000038086	chr9	50559177	50560459	-	840	-2.499	-1
Clptm1l	ENSMUSG00000021610	chr13	73741554	73758051	+	2238	-2.499	-1
Cela1	ENSMUSG00000023031	chr15	100504856	100518351	-	1103	-2.501	-1
Men1	ENSMUSG00000024947	chr19	6334979	6340891	+	3458	-2.501	-1
ORF61	ENSMUSG00000013858	chr10	79437693	79447075	-	4663	-2.502	-1
Samm50	ENSMUSG00000022437	chr15	84022663	84044733	+	1673	-2.503	-1
Mrpl41	ENSMUSG00000036850	chr2	24829637	24830618	-	776	-2.506	-1
Inca1	ENSMUSG00000057054	chr11	70501863	70513657	-	1377	-2.508	-1
Tap2	ENSMUSG00000024339	chr17	34340472	34353266	+	3781	-2.509	-1
Lrrc14	ENSMUSG00000033728	chr15	76541053	76548129	+	5647	-2.51	-1
Slc19a1	ENSMUSG00000001436	chr10	76494986	76523747	+	4644	-2.51	-1
Oscp1	ENSMUSG00000042616	chr4	125735809	125769439	+	5254	-2.511	-1
Dhrs11	ENSMUSG00000034449	chr11	84634358	84642496	-	1603	-2.512	-1
Saal1	ENSMUSG00000006763	chr7	53941478	53966050	-	8108	-2.512	-1
Dnajc4	ENSMUSG00000024963	chr19	7062401	7067008	-	1086	-2.513	-1
1810043G02Rik	ENSMUSG00000020284	chr10	77441269	77449650	+	6627	-2.513	-1
Mlst8	ENSMUSG00000024142	chr17	24610497	24616023	-	3359	-2.514	-1
Chchd2	ENSMUSG00000070493	chr5	130357027	130363340	-	2269	-2.515	-1
Cox6b1	ENSMUSG00000036751	chr7	31401993	31411170	-	505	-2.515	-1
Lst1	ENSMUSG00000073412	chr17	35322040	35325384	-	415	-2.515	-1
Trit1	ENSMUSG00000028653	chr4	122693840	122732192	+	2087	-2.517	-1
Hyou1	ENSMUSG00000032115	chr9	44187573	44200452	+	7230	-2.518	-1
Tomm7	ENSMUSG00000028998	chr5	23344762	23349979	-	1125	-2.519	-1
Cdk2ap2	ENSMUSG00000024856	chr19	4097182	4099019	+	1636	-2.52	-1
Fam73b	ENSMUSG00000026858	chr2	30219753	30241041	+	6249	-2.52	-1
Arrdc2	ENSMUSG00000002910	chr8	73359054	73363619	-	2329	-2.521	-1
Brf2	ENSMUSG00000031487	chr8	28234304	28239104	-	1383	-2.522	-1
Wif1	ENSMUSG00000020218	chr10	120471016	120537706	+	2427	-2.524	-1
Dtnb	ENSMUSG00000071454	chr12	3572381	3781796	+	9603	-2.524	-1
Alkbh2	ENSMUSG00000044339	chr5	114573935	114578227	-	1115	-2.524	-1
Dirc2	ENSMUSG00000022848	chr16	35694148	35769442	-	5395	-2.525	-1
S1pr4	ENSMUSG00000044199	chr10	80961223	80962452	-	1230	-2.525	-1

Rdh12	ENSMUSG00000021123	chr12	80309901	80323652	+	2967	-2.526		-1
Slc25a44	ENSMUSG00000050144	chr3	88214420	88229061	-	3622	-2.528		-1
Klhdc3	ENSMUSG00000063576	chr17	46811499	46817879	-	2126	-2.528		-1
Abcf3	ENSMUSG00000003234	chr16	20548650	20561452	+	3557	-2.53		-1
Slc35a2	ENSMUSG00000031156	chrX	7461167	7471618	+	3081	-2.531		-1
Mlf2	ENSMUSG00000030120	chr6	124881406	124886156	+	1533	-2.531		-1
Wdr8	ENSMUSG00000029029	chr4	153516481	153542372	+	5178	-2.531		-1
H13	ENSMUSG00000019188	chr2	152495197	152534406	+	9031	-2.532		-1
Fam100a	ENSMUSG00000039568	chr16	4874778	4880315	-	5538	-2.532		-1
Gm4945	ENSMUSG00000062472	chr17	47179533	47179988	-	456	-2.534		-1
Blvrb	ENSMUSG00000040466	chr7	28232997	28251163	+	1414	-2.536		-1
Mars	ENSMUSG00000040354	chr10	126733277	126748842	-	3423	-2.536		-1
Txn2	ENSMUSG00000005354	chr15	77745477	77759436	-	4208	-2.537		-1
Ptp4a3	ENSMUSG00000059895	chr15	73553575	73589196	+	4619	-2.54		-1
Aloxe3	ENSMUSG00000020892	chr11	68939398	68962617	+	6397	-2.541		-1
Hyi	ENSMUSG00000006395	chr4	118032595	118035349	+	1229	-2.541		-1
Shroom3	ENSMUSG00000029381	chr5	93112461	93394344	+	7755	-2.541		-1
Tab1	ENSMUSG00000022414	chr15	79963584	79992131	+	2931	-2.543		-1
C920006O11Rik	ENSMUSG00000087566	chr9	78023721	78026686	+	1444	-2.544		-1
4931408A02Rik	ENSMUSG00000039903	chr16	90826964	90905354	+	3806	-2.544		-1
Mcrs1	ENSMUSG00000037570	chr15	99073249	99082392	-	1915	-2.544		-1
A630095E13Rik	ENSMUSG00000070313	chr9	36443340	36446187	-	618	-2.545		-1
Acadvl	ENSMUSG00000018574	chr11	69823685	69828913	-	2686	-2.546		-1
Rpain	ENSMUSG00000018449	chr11	70783715	70791335	+	2842	-2.548		-1
March2	ENSMUSG00000079557	chr17	33822637	33855615	-	5997	-2.549		-1
Trappc3	ENSMUSG00000028847	chr4	125939569	125953127	+	1737	-2.549		-1
Psd4	ENSMUSG00000026979	chr2	24223100	24264702	+	6014	-2.551		-1
Cbr2	ENSMUSG00000025150	chr11	120590803	120593428	-	1327	-2.551		-1
Bcl7c	ENSMUSG00000030814	chr7	134806867	134852447	-	5331	-2.551		-1
Ndufa6	ENSMUSG00000022450	chr15	82180569	82184721	-	560	-2.551		-1
Rps28	ENSMUSG00000067288	chr17	33955972	33961507	-	1464	-2.551		-1
AI661453	ENSMUSG00000034382	chr17	47573564	47607587	+	5056	-2.552		-1
Rps19bp1	ENSMUSG00000051518	chr15	80091044	80094736	-	830	-2.552		-1
Hspb1	ENSMUSG00000004951	chr5	136363789	136365433	+	1031	-2.552		-1
Lsr	ENSMUSG00000001247	chr7	31742428	31768188	-	5008	-2.553		-1
Drg2	ENSMUSG00000020537	chr11	60268093	60282256	+	2716	-2.554		-1

Sh3bp5l	ENSMUSG00000013646	chr11	58144226	58161230	+		6352	-2.554	-1
Dnajc30	ENSMUSG00000061118	chr5	135540076	135541235	+		1160	-2.556	-1
Ptdss2	ENSMUSG00000025495	chr7	148308281	148342920	+		4875	-2.556	-1
Mrps35	ENSMUSG00000040112	chr6	146991286	147019425	+		5543	-2.556	-1
Nmral1	ENSMUSG00000063445	chr16	4710059	4719356	-		3891	-2.557	-1
Sugp1	ENSMUSG00000011306	chr8	72566712	72595852	+		2287	-2.557	-1
Rilpl2	ENSMUSG00000029401	chr5	124913274	124928375	-		1391	-2.559	-1
Stk16	ENSMUSG00000026201	chr1	75207413	75212181	+		3652	-2.559	-1
Rnf186	ENSMUSG00000070661	chr4	138523027	138524280	+		1254	-2.562	-1
Fndc4	ENSMUSG00000038552	chr5	31594628	31598250	-		2261	-2.562	-1
Ptms	ENSMUSG00000030122	chr6	124863699	124867964	-		1319	-2.563	-1
Adsl	ENSMUSG00000022407	chr15	80778920	80801377	+		4398	-2.563	-1
Ivd	ENSMUSG00000027332	chr2	118687690	118708645	+		5505	-2.565	-1
Idh3g	ENSMUSG00000002010	chrX	71024302	71032236	-		2701	-2.566	-1
Rpl10a	ENSMUSG00000037805	chr17	28465416	28467977	+		2349	-2.568	-1
Serhl	ENSMUSG00000058586	chr15	82919936	82947104	+		7393	-2.568	-1
Gnl1	ENSMUSG00000024429	chr17	36116796	36126407	+		3077	-2.568	-1
Spink8	ENSMUSG00000050074	chr9	109714794	109729147	+		794	-2.568	-1
Fbxo36	ENSMUSG00000073633	chr1	84836416	84897062	+		2148	-2.57	-1
Prmt1	ENSMUSG00000052429	chr7	52231359	52241862	-		2521	-2.571	-1
Tk2	ENSMUSG00000035824	chr8	106750591	106772458	-		2400	-2.571	-1
Renbp	ENSMUSG00000031387	chrX	71167460	71176189	-		2494	-2.571	-1
Rgs11	ENSMUSG00000024186	chr17	26339896	26348269	+		4206	-2.572	-1
Sertad3	ENSMUSG00000055200	chr7	28258787	28262378	+		1324	-2.574	-1
Acbd6	ENSMUSG00000033701	chr1	157405250	157534363	+		2284	-2.575	-1
Gm10638	ENSMUSG00000074178	chr8	89269598	89270502	+		905	-2.576	-1
Tfeb	ENSMUSG00000023990	chr17	47873979	47929368	+		5669	-2.577	-1
Nsmce1	ENSMUSG00000030750	chr7	132611154	132635110	-		1329	-2.577	-1
Btd	ENSMUSG00000021900	chr14	32454214	32481765	+		2353	-2.58	-1
Adat3	ENSMUSG00000035370	chr10	80065625	80070399	+		1411	-2.582	-1
Shq1	ENSMUSG00000035378	chr6	100523075	100621151	-		2182	-2.583	-1
Gabarapl1	ENSMUSG00000030161	chr6	129483218	129492349	+		1765	-2.584	-1
Rnf31	ENSMUSG00000047098	chr14	56210545	56222530	+		4838	-2.586	-1
BC004004	ENSMUSG00000052712	chr17	29405733	29439826	+		6017	-2.586	-1
Slc12a9	ENSMUSG00000037344	chr5	137755786	137774825	-		5020	-2.586	-1
Sdhc	ENSMUSG00000058076	chr1	173057296	173080734	-		3250	-2.586	-1
Med25	ENSMUSG00000002968	chr7	52134761	52147779	-		2540	-2.587	-1
Preb	ENSMUSG00000045302	chr5	31253226	31262703	-		7176	-2.587	-1
Cacnb3	ENSMUSG00000003352	chr15	98462651	98474961	+		3120	-2.589	-1
Ethe1	ENSMUSG00000064254	chr7	25372562	25393944	+		1484	-2.59	-1
Hist2h3c2	ENSMUSG00000081058	chr3	96042040	96043050	-		1011	-2.59	-1

D630045J12Rik	ENSMUSG00000063455	chr6	38073174	38204009	-	13450	-2.591	-1
BC046404	ENSMUSG00000046417	chr11	62418386	62462025	-	2089	-2.591	-1
Il28ra	ENSMUSG00000062157	chr4	135242202	135264096	+	4182	-2.591	-1
Fam53a	ENSMUSG00000037339	chr5	33943000	33972284	-	9110	-2.593	-1
Dmap1	ENSMUSG00000009640	chr4	117347286	117354878	-	2636	-2.593	-1
Bco2	ENSMUSG00000032066	chr9	50341192	50363286	-	2118	-2.594	-1
Twf2	ENSMUSG00000023277	chr9	106105439	106117718	+	1594	-2.594	-1
Ankrd39	ENSMUSG00000079610	chr1	36595018	36604046	-	1786	-2.594	-1
Tcea3	ENSMUSG00000001604	chr4	135803644	135830813	+	1400	-2.595	-1
Ip6k1	ENSMUSG00000032594	chr9	107904832	107951111	+	6483	-2.595	-1
Manbal	ENSMUSG00000063019	chr2	157193330	157222499	+	1107	-2.595	-1
Slc27a4	ENSMUSG00000059316	chr2	29658154	29673042	+	4412	-2.596	-1
Gtf2ird2	ENSMUSG00000015942	chr5	134659889	134700225	+	5572	-2.596	-1
Mrps27	ENSMUSG00000041632	chr13	100114741	100185516	+	1848	-2.596	-1
4930579K19Rik	ENSMUSG00000051074	chr9	98463379	98464030	-	652	-2.597	-1
Rbfa	ENSMUSG00000024570	chr18	80389004	80397397	-	1377	-2.597	-1
Rpl8	ENSMUSG00000003970	chr15	76734501	76736748	+	862	-2.597	-1
BC096441	ENSMUSG00000018752	chr11	69496079	69509600	-	2232	-2.6	-1
Mustn1	ENSMUSG00000042485	chr14	31692443	31694794	+	1177	-2.6	-1
Pepd	ENSMUSG00000063931	chr7	35697398	35829729	+	3079	-2.6	-1
Dtd1	ENSMUSG00000027430	chr2	144425633	144594494	+	2353	-2.601	-1
Dnase2a	ENSMUSG00000003812	chr8	87432459	87446814	+	5389	-2.603	-1
Yrdc	ENSMUSG00000028889	chr4	124527923	124532486	+	1981	-2.604	-1
Jmjd5	ENSMUSG00000030752	chr7	132588190	132605779	+	2385	-2.608	-1
Slc11a2	ENSMUSG00000023030	chr15	100218329	100255503	-	6354	-2.61	-1
Zfp956	ENSMUSG00000045466	chr6	47903389	47915299	+	2968	-2.611	-1
Tmed1	ENSMUSG00000032180	chr9	21311824	21314631	-	1488	-2.611	-1
Mylpf	ENSMUSG00000030672	chr7	134355122	134357812	+	885	-2.612	-1
Tmem115	ENSMUSG00000010045	chr9	107436276	107440987	+	2177	-2.614	-1
2310046O06Rik	ENSMUSG00000032305	chr9	57385335	57390994	+	3055	-2.614	-1
Abcc10	ENSMUSG00000032842	chr17	46440170	46465301	-	6856	-2.615	-1
Gnb2l1	ENSMUSG00000020372	chr11	48613834	48619936	+	5119	-2.615	-1
Gm14302	ENSMUSG00000085760	chr2	164285396	164286455	-	382	-2.616	-1
Sept1	ENSMUSG00000000486	chr7	134357961	134362012	-	2060	-2.616	-1
Nr1h2	ENSMUSG00000060601	chr7	51804986	51809321	-	3366	-2.618	-1
Mrpl51	ENSMUSG00000030335	chr6	125141819	125145601	+	3357	-2.619	-1

Lyrm4	ENSMUSG00000046573	chr13	36070666	36209226	-	1515	-2.62	-1
Crip2	ENSMUSG00000006356	chr12	114378447	114383715	+	1437	-2.62	-1
Rpl37a	ENSMUSG00000046330	chr1	72757834	72760383	+	584	-2.621	-1
Flot1	ENSMUSG00000059714	chr17	35960175	35969736	+	2893	-2.623	-1
Dgcr2	ENSMUSG00000003166	chr16	17839575	17894238	-	6673	-2.623	-1
Rfxank	ENSMUSG00000036120	chr8	72654711	72663096	-	3676	-2.623	-1
AU022754	ENSMUSG00000087046	chr15	85411572	85424138	+	2976	-2.624	-1
Fam83f	ENSMUSG00000022408	chr15	80501783	80530850	+	4771	-2.625	-1
Lap3	ENSMUSG00000039682	chr5	45884613	45903913	+	3072	-2.626	-1
H2-T23	ENSMUSG00000067212	chr17	36166718	36169800	-	2889	-2.626	-1
9430038I01Rik	ENSMUSG00000040139	chr7	144567257	144602445	-	5752	-2.629	-1
Mal	ENSMUSG00000027375	chr2	127458962	127482431	-	2778	-2.63	-1
Kdelr1	ENSMUSG00000002778	chr7	53128142	53139101	+	1873	-2.63	-1
Stard3	ENSMUSG00000018167	chr11	98219682	98242426	+	3557	-2.63	-1
Tmem200b	ENSMUSG00000070720	chr4	131477686	131478540	+	855	-2.631	-1
Fbxo31	ENSMUSG00000031811	chr8	124075879	124102706	-	1819	-2.631	-1
Zgpat	ENSMUSG00000027582	chr2	181099633	181118333	+	5624	-2.631	-1
Pxmp2	ENSMUSG00000029499	chr5	110703301	110715205	-	1200	-2.632	-1
Trmt2a	ENSMUSG00000022721	chr16	18248772	18254865	+	4952	-2.632	-1
Snf8	ENSMUSG00000006058	chr11	95896199	95908744	+	2677	-2.633	-1
Calr	ENSMUSG00000003814	chr8	87365749	87370833	-	2880	-2.634	-1
0610037L13Rik	ENSMUSG00000028608	chr4	107562418	107571989	+	5633	-2.634	-1
Snta1	ENSMUSG00000027488	chr2	154202049	154233835	-	3094	-2.634	-1
Sharpin	ENSMUSG00000022552	chr15	76177470	76181541	-	3765	-2.634	-1
P2rx1	ENSMUSG00000020787	chr11	72812605	72828702	+	2625	-2.635	-1
Ubl5	ENSMUSG00000084786	chr9	20447322	20451584	+	3468	-2.635	-1
Bbc3	ENSMUSG00000002083	chr7	16894965	16903554	+	2008	-2.635	-1
Fxyd3	ENSMUSG00000057092	chr7	31853191	31861723	-	4770	-2.636	-1
1300018I17Rik	ENSMUSG00000001062	chr8	125766256	125778248	-	5153	-2.636	-1
Fdx1l	ENSMUSG00000079677	chr9	20871964	20877958	-	782	-2.637	-1
Dhrs7	ENSMUSG00000021094	chr12	73751343	73765815	-	1301	-2.638	-1
Nop10	ENSMUSG00000027133	chr2	112102083	112103426	+	1109	-2.639	-1
Rpl39	ENSMUSG00000079641	chrX	34622515	34625397	-	1434	-2.639	-1
Ecsit	ENSMUSG00000066839	chr9	21876714	21889819	-	1589	-2.64	-1
Ccdc3	ENSMUSG00000026676	chr2	5058822	5151924	+	2670	-2.64	-1
Tufm	ENSMUSG00000073838	chr7	133630869	133634244	+	1776	-2.642	-1
Ypel3	ENSMUSG00000042675	chr7	133920469	133924028	+	1848	-2.643	-1
Spock2	ENSMUSG00000058297	chr10	59568967	59597946	+	5524	-2.644	-1
Fbxw4	ENSMUSG00000040913	chr19	45652744	45734802	-	3158	-2.644	-1
Fjx1	ENSMUSG00000075012	chr2	102289525	102291949	-	2425	-2.645	-1
Nt5m	ENSMUSG00000032615	chr11	59652949	59694470	+	3361	-2.646	-1
Tex264	ENSMUSG00000040813	chr9	106561078	106588279	-	2268	-2.647	-1

B3galt6	ENSMUSG00000050796	chr4	155363575	155366787	-	3213	-2.647	-1
Rpl37	ENSMUSG00000041841	chr15	5066613	5069140	+	867	-2.648	-1
Sod2	ENSMUSG00000006818	chr17	13200705	13210985	+	3824	-2.648	-1
Ftsj2	ENSMUSG00000029557	chr5	140803628	140807852	-	1443	-2.648	-1
Ccdc134	ENSMUSG00000068114	chr15	81958352	81972630	+	2043	-2.649	-1
Stard5	ENSMUSG00000046027	chr7	90780469	90801637	+	5450	-2.649	-1
Lin37	ENSMUSG00000036845	chr7	31340460	31344857	-	1388	-2.65	-1
A430005L14Rik	ENSMUSG00000047613	chr4	153331346	153336034	+	1552	-2.652	-1
Dennd3	ENSMUSG00000036661	chr15	73342990	73402672	+	5703	-2.652	-1
Stk32c	ENSMUSG00000015981	chr7	146289537	146399206	-	2302	-2.652	-1
Snap47	ENSMUSG00000009894	chr11	59220636	59264688	-	2651	-2.653	-1
BC017158	ENSMUSG00000030780	chr7	135414893	135441684	-	5526	-2.653	-1
1110008J03Rik	ENSMUSG00000029600	chr5	121044314	121062598	-	2836	-2.655	-1
Hist1h1b	ENSMUSG00000058773	chr13	21871752	21872494	-	743	-2.656	-1
Ppp2r4	ENSMUSG00000039515	chr2	30271559	30303326	+	5188	-2.658	-1
Cyp4f17	ENSMUSG00000091586	chr17	32643407	32665839	+	1693	-2.659	-1
Ift46	ENSMUSG00000002031	chr9	44575991	44600797	+	5695	-2.66	-1
Rnf114	ENSMUSG00000006418	chr2	167318145	167341673	+	3356	-2.662	-1
Zfyve19	ENSMUSG00000068580	chr2	119034353	119042785	+	3255	-2.662	-1
Hspb7	ENSMUSG00000006221	chr4	140976694	140981226	+	2769	-2.663	-1
Sirt7	ENSMUSG00000025138	chr11	120479686	120486554	-	5148	-2.664	-1
Stk19	ENSMUSG00000061207	chr17	34960938	34973890	-	3155	-2.664	-1
Myg1	ENSMUSG00000001285	chr15	102162140	102168570	+	2823	-2.666	-1
Ppcdc	ENSMUSG00000063849	chr9	57260475	57287921	-	2747	-2.667	-1
Gsta4	ENSMUSG00000032348	chr9	78039773	78057155	+	957	-2.667	-1
Hexdc	ENSMUSG00000039307	chr11	121065747	121083969	+	4727	-2.668	-1
Pgm2l1	ENSMUSG00000030729	chr7	107375904	107427378	+	7036	-2.668	-1
Hmgcs2	ENSMUSG00000027875	chr3	98084358	98114661	+	3290	-2.668	-1
Pop7	ENSMUSG00000029715	chr5	137942666	137943746	-	1081	-2.669	-1
Bambi	ENSMUSG00000024232	chr18	3507955	3516402	+	5040	-2.67	-1
Aarsd1	ENSMUSG00000075528	chr11	101268006	101286647	-	4699	-2.671	-1
Arhgdig	ENSMUSG00000073433	chr17	26333171	26344731	-	3252	-2.671	-1
C030013C21Rik	ENSMUSG00000085078	chr11	80320912	80322608	-	795	-2.671	-1
2310009B15Rik	ENSMUSG00000079283	chr1	140748556	140753431	-	571	-2.672	-1
Armc6	ENSMUSG00000002343	chr8	72744071	72758365	-	3262	-2.673	-1
Zfp513	ENSMUSG00000043059	chr5	31501354	31504676	-	2476	-2.673	-1
Adcy5	ENSMUSG00000022840	chr16	35154963	35305823	+	7007	-2.674	-1

Ly6e	ENSMUSG00000022587	chr15	74785481	74790335	+	2301	-2.674	-1
Gm8894	ENSMUSG00000060680	chr14	56039106	56039561	-	456	-2.675	-1
Tmem180	ENSMUSG00000025227	chr19	46415611	46449742	+	2804	-2.675	-1
Hint1	ENSMUSG00000020267	chr11	54679885	54684003	+	872	-2.677	-1
2310011J03Rik	ENSMUSG00000020133	chr10	79780999	79783282	-	1649	-2.677	-1
2510002D24Rik	ENSMUSG00000071632	chr16	18836671	18840251	+	1433	-2.678	-1
Ddx41	ENSMUSG00000021494	chr13	55631771	55638019	-	2205	-2.678	-1
Med28	ENSMUSG00000015804	chr5	45911460	45920515	+	5010	-2.678	-1
Acad8	ENSMUSG00000031969	chr9	26781720	26807151	-	4143	-2.678	-1
Glb1	ENSMUSG00000045594	chr9	114310194	114383495	+	2936	-2.679	-1
Heatr7a	ENSMUSG00000022558	chr15	76210691	76283468	+	16533	-2.68	-1
Zdhhc7	ENSMUSG00000031823	chr8	122604995	122625382	-	3472	-2.681	-1
2900002K06Rik	ENSMUSG00000055188	chrX	7722555	7725090	+	971	-2.683	-1
Abt1	ENSMUSG00000036376	chr13	23513408	23515735	-	1278	-2.683	-1
Tmem120b	ENSMUSG00000054434	chr5	123518424	123567758	+	2852	-2.683	-1
Plekhg6	ENSMUSG00000038167	chr6	125312678	125330811	-	3299	-2.683	-1
Bop1	ENSMUSG00000022557	chr15	76283426	76307699	-	2499	-2.686	-1
Lass4	ENSMUSG00000008206	chr8	4493026	4525757	+	6361	-2.687	-1
Ndufc1	ENSMUSG00000037152	chr3	51208599	51212910	-	1489	-2.687	-1
Ndufa9	ENSMUSG00000000399	chr6	126771881	126799162	-	1322	-2.687	-1
Oplah	ENSMUSG00000022562	chr15	76127027	76158445	-	5596	-2.687	-1
Znhit1	ENSMUSG00000059518	chr5	137458034	137463891	-	1860	-2.688	-1
Plekhf1	ENSMUSG00000074170	chr7	39005673	39013013	-	1559	-2.688	-1
Cops7a	ENSMUSG00000030127	chr6	124908431	124915556	-	2711	-2.689	-1
Myl6	ENSMUSG00000090841	chr10	127927916	127930931	-	665	-2.693	-1
Mapk12	ENSMUSG00000022610	chr15	88961015	88971133	-	1889	-2.693	-1
Timm10	ENSMUSG00000027076	chr2	84667154	84670370	+	815	-2.693	-1
Higd2a	ENSMUSG00000025868	chr13	54691568	54692519	+	648	-2.694	-1
Cul9	ENSMUSG00000040327	chr17	46637554	46683337	-	7851	-2.695	-1
2310061C15Rik	ENSMUSG00000014633	chr8	119412585	119445355	-	2043	-2.696	-1
Ldoc1l	ENSMUSG00000055745	chr15	84383828	84387922	-	4095	-2.696	-1
Acot11	ENSMUSG00000034853	chr4	106417160	106477603	-	6080	-2.697	-1
6430548M08Rik	ENSMUSG00000031824	chr8	122638052	122689206	+	6626	-2.698	-1
Prmt7	ENSMUSG00000060098	chr8	108734836	108776694	+	6125	-2.698	-1
Rasd1	ENSMUSG00000049892	chr11	59776683	59778446	-	1611	-2.699	-1
Nudt6	ENSMUSG00000050174	chr3	37303832	37319133	-	1635	-2.699	-1

Gemin8	ENSMUSG00000040621	chrX	162608386	162628444	+	2487	-2.7	-1
Nosip	ENSMUSG00000003421	chr7	52317799	52333873	+	2920	-2.7	-1
2810410 L24Rik	ENSMUSG00000075389	chr11	120046714	120051166	-	3028	-2.701	-1
Nt5c3l	ENSMUSG00000017176	chr11	100283635	100303122	-	3420	-2.702	-1
2210016 F16Rik	ENSMUSG00000021550	chr13	58481407	58486586	-	2822	-2.702	-1
Tnni3	ENSMUSG00000035458	chr7	4469907	4475831	-	1928	-2.702	-1
Hscb	ENSMUSG00000043510	chr5	111258089	111268796	-	905	-2.703	-1
Mapk3	ENSMUSG00000063065	chr7	133903115	133909333	+	2163	-2.703	-1
March9	ENSMUSG00000040502	chr10	126492067	126497240	-	3037	-2.703	-1
Smarcd 3	ENSMUSG00000028949	chr5	24096636	24107830	-	4545	-2.704	-1
Rit1	ENSMUSG00000028057	chr3	88520760	88534971	+	3229	-2.704	-1
Polr2c	ENSMUSG00000031783	chr8	97381350	97397435	+	4736	-2.707	-1
Pet112l	ENSMUSG00000028085	chr3	85378041	85459544	+	4299	-2.707	-1
Slc35b1	ENSMUSG00000020873	chr11	95246006	95253090	+	2223	-2.711	-1
Ing4	ENSMUSG00000030330	chr6	124989778	125001517	+	6675	-2.711	-1
Slc35a4	ENSMUSG00000033272	chr18	36839233	36843512	+	2908	-2.713	-1
Egfl8	ENSMUSG00000015467	chr17	34750294	34752916	-	1761	-2.715	-1
Rhbdf1	ENSMUSG00000020282	chr11	32109585	32122300	-	5433	-2.716	-1
Nenf	ENSMUSG00000037499	chr1	193130668	193142073	-	742	-2.717	-1
Stxbp6	ENSMUSG00000046314	chr12	45953475	46175480	-	4855	-2.717	-1
Ctrl	ENSMUSG00000031896	chr8	108455894	108457762	-	888	-2.717	-1
Capn10	ENSMUSG00000026270	chr1	94830953	94844518	+	4346	-2.718	-1
Nup210	ENSMUSG00000030091	chr6	90963062	91066823	-	8213	-2.722	-1
Tubb4b	ENSMUSG00000036752	chr2	25077680	25080222	-	2088	-2.722	-1
Fau	ENSMUSG00000038274	chr19	6057893	6059516	+	561	-2.722	-1
Chchd5	ENSMUSG00000037938	chr2	128955436	128959870	+	1727	-2.722	-1
Tmub2	ENSMUSG00000034757	chr11	102146245	102150551	+	2544	-2.722	-1
2010300 C02Rik	ENSMUSG00000026090	chr1	37668522	37776930	-	5075	-2.723	-1
Wdr25	ENSMUSG00000040877	chr12	110132482	110266662	+	2742	-2.724	-1
Chchd1	ENSMUSG00000063787	chr14	21522249	21523639	+	587	-2.724	-1
Slc17a9	ENSMUSG00000023393	chr2	180459968	180476985	+	3200	-2.725	-1
Gm1760 6	ENSMUSG00000091306	chr14	55259789	55262854	+	159	-2.725	-1
1810010 H24Rik	ENSMUSG00000078607	chr11	106889432	106891757	+	1205	-2.725	-1
Dsg2	ENSMUSG00000044393	chr18	20716575	20763022	+	8268	-2.727	-1
Fam176 b	ENSMUSG00000050212	chr4	125824988	125827119	+	1942	-2.728	-1
Ppcs	ENSMUSG00000028636	chr4	119091135	119095025	-	1605	-2.728	-1

Nipsnap1	ENSMUSG00000034285	chr11	4773954	4794203	+	2649	-2.729	-1
Mxd4	ENSMUSG00000037235	chr5	34516536	34530369	-	3882	-2.73	-1
Ssh3	ENSMUSG00000034616	chr19	4261668	4269172	-	2740	-2.73	-1
Acvr2b	ENSMUSG00000061393	chr9	119311562	119342619	+	1753	-2.73	-1
Pigo	ENSMUSG00000028454	chr4	43030507	43038691	-	4283	-2.73	-1
E130309D02Rik	ENSMUSG00000039244	chr5	144062874	144077039	-	4639	-2.73	-1
Slc25a19	ENSMUSG00000020744	chr11	115475492	115489595	-	4012	-2.732	-1
Ccm2	ENSMUSG00000000378	chr11	6446890	6496747	+	3675	-2.732	-1
Mrpl22	ENSMUSG00000020514	chr11	57985163	57993067	+	981	-2.733	-1
Gata2	ENSMUSG00000015053	chr6	88143885	88157024	+	3351	-2.734	-1
3110040N11Rik	ENSMUSG00000025102	chr7	88927068	88934364	-	1231	-2.735	-1
Gnao1	ENSMUSG00000031748	chr8	96333866	96493288	+	6785	-2.739	-1
Cc2d1a	ENSMUSG00000036686	chr8	86656727	86671835	-	3710	-2.74	-1
Prosapip1	ENSMUSG00000037703	chr2	130458575	130468539	-	4252	-2.74	-1
Acd	ENSMUSG00000038000	chr8	108222058	108224995	-	1530	-2.741	-1
Ino80b	ENSMUSG00000030034	chr6	83071759	83075425	-	1503	-2.743	-1
Kctd17	ENSMUSG00000033287	chr15	78258994	78269339	+	2134	-2.744	-1
C330006A16Rik	ENSMUSG00000087679	chr2	25992334	25996041	-	2483	-2.745	-1
Irf6	ENSMUSG00000026638	chr1	194979306	194998236	+	6938	-2.745	-1
Eif6	ENSMUSG00000027613	chr2	155645568	155652661	-	2702	-2.747	-1
Gm10036	ENSMUSG00000058064	chr18	15991296	15991878	+	583	-2.747	-1
Entpd4	ENSMUSG00000022066	chr14	69955208	69985327	+	4202	-2.747	-1
Mfsd11	ENSMUSG00000020818	chr11	116713754	116736949	+	7076	-2.75	-1
Lpar5	ENSMUSG00000067714	chr6	125017938	125032490	+	1515	-2.751	-1
H47	ENSMUSG00000075701	chr7	73224535	73234298	+	1198	-2.751	-1
Coq2	ENSMUSG00000029319	chr5	101083742	101104159	-	2624	-2.753	-1
Ppp1ca	ENSMUSG00000040385	chr19	4192158	4195419	+	1431	-2.753	-1
Cox5b	ENSMUSG00000061518	chr1	36748332	36750230	+	566	-2.753	-1
Gpx1	ENSMUSG00000063856	chr9	108241607	108242671	+	847	-2.753	-1
Serpinb1a	ENSMUSG00000044734	chr13	32933961	32943054	-	1943	-2.755	-1
Eef1d	ENSMUSG00000055762	chr15	75725226	75739986	-	5897	-2.755	-1
Smcr7	ENSMUSG00000018599	chr11	60541900	60546453	+	2716	-2.755	-1
D8Ertd738e	ENSMUSG00000019362	chr8	86770136	86773660	-	659	-2.758	-1
Tapbpl	ENSMUSG00000038213	chr6	125173951	125181878	-	2352	-2.758	-1
Farsa	ENSMUSG00000003808	chr8	87380888	87393156	+	4538	-2.76	-1
Bdh2	ENSMUSG00000028167	chr3	134944185	134967390	+	1202	-2.76	-1
Gltscr2	ENSMUSG00000041560	chr7	16523187	16531457	-	1555	-2.76	-1

Ssna1	ENSMUSG00000026966	chr2	25126559	25127958	-	1276	-2.76		-1
Bcdin3d	ENSMUSG00000037525	chr15	99300515	99305161	-	1271	-2.761		-1
Tbcd	ENSMUSG00000039230	chr11	121313263	121478478	+	8096	-2.764		-1
Rpl35a	ENSMUSG00000060636	chr16	33056539	33060275	+	3526	-2.765		-1
Rps19	ENSMUSG00000040952	chr7	25669390	25674825	+	4685	-2.765		-1
Grik5	ENSMUSG00000003378	chr7	25794872	25857388	-	3836	-2.765		-1
Selm	ENSMUSG00000075702	chr11	3414687	3417354	+	1481	-2.767		-1
Gm11273	ENSMUSG00000079941	chr13	21592898	21593287	-	390	-2.767		-1
Mrgprf	ENSMUSG00000031070	chr7	152486733	152495462	+	2214	-2.768		-1
Arg2	ENSMUSG00000021125	chr12	80231764	80257288	+	1428	-2.768		-1
Col20a1	ENSMUSG00000016356	chr2	180721240	180752245	+	4193	-2.769		-1
Gm5884	ENSMUSG00000037827	chr6	128594931	128596409	+	1479	-2.77		-1
D17H6S53E	ENSMUSG00000043311	chr17	35263318	35265800	+	2093	-2.771		-1
Rpa2	ENSMUSG00000028884	chr4	132324247	132334667	+	2993	-2.771		-1
Ilvbl	ENSMUSG00000032763	chr10	78037245	78047243	+	2385	-2.773		-1
Psmb3	ENSMUSG00000069744	chr11	97564713	97574814	+	2300	-2.773		-1
Tcn2	ENSMUSG00000020432	chr11	3817195	3832162	-	2178	-2.775		-1
Fmo5	ENSMUSG00000028088	chr3	97432727	97459205	+	5675	-2.777		-1
Ctsd	ENSMUSG00000007891	chr7	149557053	149573943	-	2519	-2.779		-1
Rgl3	ENSMUSG00000040146	chr9	21775971	21793897	-	2550	-2.78		-1
Ace	ENSMUSG00000020681	chr11	105829259	105851278	+	5287	-2.781		-1
Map1lc3b	ENSMUSG00000031812	chr8	124114368	124121947	+	1712	-2.781		-1
5031434O11Rik	ENSMUSG00000085462	chr3	51363679	51371039	+	3754	-2.781		-1
Gm6396	ENSMUSG00000066955	chr8	4652064	4652480	+	417	-2.782		-1
Rplp0	ENSMUSG00000067274	chr5	116009476	116013736	+	1659	-2.783		-1
Scrib	ENSMUSG00000022568	chr15	75877588	75900214	-	5736	-2.784		-1
Slc25a11	ENSMUSG00000014606	chr11	70457698	70460981	-	2120	-2.785		-1
Psenen	ENSMUSG00000036835	chr7	31346887	31348203	-	648	-2.786		-1
Snhg9	ENSMUSG00000090101	chr17	24856476	24856910	-	183	-2.786		-1
Zfyve21	ENSMUSG00000021286	chr12	113052381	113066596	+	1307	-2.786		-1
Ndufs3	ENSMUSG00000005510	chr2	90734791	90744984	-	2178	-2.786		-1
Pdia6	ENSMUSG00000020571	chr12	17273351	17291576	+	3745	-2.787		-1
Drap1	ENSMUSG00000024914	chr19	5422805	5424979	-	1741	-2.787		-1
Tubd1	ENSMUSG00000020513	chr11	86358493	86380862	+	2070	-2.788		-1
Nme4	ENSMUSG00000024177	chr17	26228679	26232453	-	1418	-2.788		-1
Zfpl1	ENSMUSG00000024792	chr19	6076487	6084944	-	2332	-2.789		-1
Slc25a47	ENSMUSG00000048856	chr12	110089339	110095024	+	1579	-2.789		-1
Rai2	ENSMUSG00000043518	chrX	158155001	158217428	+	2403	-2.789		-1

Tcap	ENSMUSG00000007877	chr11	98245125	98246267	+	948	-2.789	-1
Aip	ENSMUSG00000024847	chr19	4114446	4125858	-	1937	-2.791	-1
Tapbp	ENSMUSG00000024308	chr17	34052844	34066235	+	3298	-2.792	-1
Cisd1	ENSMUSG00000037710	chr10	70793228	70807702	-	1084	-2.793	-1
Larp1b	ENSMUSG00000037814	chr3	40754553	40781715	+	1864	-2.798	-1
0610007 P14Rik	ENSMUSG00000021252	chr12	87156398	87165500	-	4235	-2.799	-1
Gsdmd	ENSMUSG00000022575	chr15	75692769	75697834	+	1776	-2.8	-1
Anapc7	ENSMUSG00000029466	chr5	122871702	122894921	+	5119	-2.8	-1
Cox7a2	ENSMUSG00000032330	chr9	79603048	79607660	-	754	-2.802	-1
Cox7a2l	ENSMUSG00000024248	chr17	83901259	83913673	-	1147	-2.803	-1
Rpusd3	ENSMUSG00000051169	chr6	113365313	113369342	-	2161	-2.804	-1
Bmp4	ENSMUSG00000021835	chr14	47003195	47010344	-	2224	-2.805	-1
Dpcd	ENSMUSG00000041035	chr19	45635105	45652769	+	1345	-2.805	-1
Rps16	ENSMUSG00000037563	chr7	29135671	29137715	+	807	-2.805	-1
Ihh	ENSMUSG00000006538	chr1	74991891	74998246	-	2466	-2.806	-1
Stx5a	ENSMUSG00000010110	chr19	8815903	8830559	+	5808	-2.806	-1
Mfsd12	ENSMUSG00000034854	chr10	80820236	80828970	+	3971	-2.807	-1
Pex14	ENSMUSG00000028975	chr4	148334644	148473985	-	6272	-2.807	-1
Rabep2	ENSMUSG00000030727	chr7	133572273	133592759	+	5322	-2.808	-1
Mul1	ENSMUSG00000041241	chr4	137990586	137998180	+	4000	-2.809	-1
Akt2	ENSMUSG00000004056	chr7	28376571	28425845	+	7131	-2.809	-1
Dnajb2	ENSMUSG00000026203	chr1	75233040	75241379	+	1409	-2.809	-1
Tap1	ENSMUSG00000037321	chr17	34324498	34334170	+	5476	-2.81	-1
Zfp414	ENSMUSG00000073423	chr17	33766035	33768724	+	1966	-2.811	-1
U2af1l4	ENSMUSG00000078765	chr7	31348320	31352979	+	3804	-2.811	-1
Bod1	ENSMUSG00000044502	chr11	31565181	31571919	-	1678	-2.811	-1
Sec14l2	ENSMUSG00000003585	chr11	3997042	4023418	-	3505	-2.813	-1
Nme6	ENSMUSG00000032478	chr9	109735263	109745630	+	2093	-2.813	-1
Creb3	ENSMUSG00000028466	chr4	43575204	43579932	+	2372	-2.814	-1
Nthl1	ENSMUSG00000041429	chr17	24769627	24775783	+	1080	-2.814	-1
Poldip2	ENSMUSG00000001100	chr11	78325695	78336238	+	3783	-2.815	-1
2700073 G19Rik	ENSMUSG00000021041	chr12	88783633	88784967	-	1335	-2.815	-1
Atp1b1	ENSMUSG00000026576	chr1	166367398	166388486	-	2422	-2.815	-1
Gm8730	ENSMUSG00000063696	chr8	105388679	105389777	-	1099	-2.815	-1
2510006 D16Rik	ENSMUSG00000028797	chr4	129277912	129301303	+	4273	-2.816	-1
Fndc5	ENSMUSG00000001334	chr4	128814243	128821837	+	2763	-2.818	-1
Grhl1	ENSMUSG00000020656	chr12	25257152	25302256	+	3500	-2.818	-1
2900052 L18Rik	ENSMUSG00000043993	chr11	120091116	120092899	-	1588	-2.821	-1

Mansc1	ENSMUSG00000032718	chr6	134559228	134582506	-	2373	-2.822	-1
Trp53i13	ENSMUSG00000044328	chr11	77321601	77329482	-	2157	-2.822	-1
Pcyt2	ENSMUSG00000025137	chr11	120471401	120479250	-	4346	-2.822	-1
Tmem106c	ENSMUSG00000052369	chr15	97794710	97800705	+	1573	-2.822	-1
Pemt	ENSMUSG00000000301	chr11	59784116	59859991	-	2104	-2.824	-1
Mmgt2	ENSMUSG00000048497	chr11	62462166	62479856	+	4898	-2.826	-1
Cyb561d2	ENSMUSG00000037190	chr9	107441342	107444196	-	1782	-2.827	-1
Rxrb	ENSMUSG00000039656	chr17	34168757	34175338	+	3698	-2.828	-1
Ddrgk1	ENSMUSG00000068290	chr2	130479696	130490395	-	2084	-2.828	-1
Mbd3	ENSMUSG00000035478	chr10	79855284	79862295	-	1987	-2.829	-1
Ppil6	ENSMUSG00000078451	chr10	41210245	41234092	+	1068	-2.829	-1
Slc6a9	ENSMUSG00000028542	chr4	117507111	117547803	+	5141	-2.829	-1
Mir196a-1	ENSMUSG00000092449	chr11	96126478	96126579	+	102	-2.832	-1
Adck4	ENSMUSG00000003762	chr7	28018042	28047271	+	3175	-2.833	-1
Cbx8	ENSMUSG00000025578	chr11	118897619	118902283	-	3675	-2.834	-1
Tbcb	ENSMUSG00000006095	chr7	31009150	31017291	-	1442	-2.835	-1
Zfp768	ENSMUSG00000047371	chr7	134486311	134488898	-	2315	-2.836	-1
Tmem30b	ENSMUSG00000034435	chr12	74644101	74647382	-	3282	-2.836	-1
Wnk4	ENSMUSG00000035112	chr11	101121881	101138723	+	4148	-2.838	-1
Adam11	ENSMUSG00000020926	chr11	102622753	102641576	+	5669	-2.838	-1
Triap1	ENSMUSG00000029535	chr5	115791234	115793578	+	1088	-2.841	-1
Nprl2	ENSMUSG00000010057	chr9	107444540	107448035	+	1456	-2.842	-1
Apba3	ENSMUSG00000004931	chr10	80730917	80735991	+	2106	-2.843	-1
Spryd3	ENSMUSG00000036966	chr15	101946776	101966665	-	3110	-2.844	-1
C2cd4b	ENSMUSG00000091956	chr9	67607244	67608736	+	1326	-2.844	-1
P4hb	ENSMUSG00000025130	chr11	120421612	120434567	-	2861	-2.847	-1
Tmem14c	ENSMUSG00000021361	chr13	41111619	41117944	+	945	-2.849	-1
Ccdc28b	ENSMUSG00000028795	chr4	129296518	129301191	-	2026	-2.85	-1
Nagk	ENSMUSG00000034744	chr6	83744976	83753414	+	3293	-2.85	-1
Chtf8	ENSMUSG00000046691	chr8	109407763	109417501	-	3036	-2.852	-1
6330512M04Rik	ENSMUSG00000045777	chr7	149511742	149559658	-	4789	-2.853	-1
Spr-ps1	ENSMUSG00000068303	chr6	85104999	85106228	+	621	-2.853	-1
2010107H07Rik	ENSMUSG00000058351	chr14	31902055	31942116	-	592	-2.854	-1
9430023L20Rik	ENSMUSG00000037204	chr15	101114732	101121365	+	1230	-2.855	-1
Wdtc1	ENSMUSG00000037622	chr4	132848374	132909396	-	4800	-2.858	-1
Tmc6	ENSMUSG00000025572	chr11	117627302	117643512	-	4091	-2.858	-1

Slc39a3	ENSMUSG00000046822	chr10	80491283	80500171	-	4016	-2.858	-1
Polr2e	ENSMUSG00000004667	chr10	79498694	79502540	-	2670	-2.86	-1
Ddah2	ENSMUSG00000007039	chr17	35195980	35199040	+	1593	-2.861	-1
Zswim1	ENSMUSG00000017764	chr2	164648186	164652371	+	2781	-2.861	-1
Cox8a	ENSMUSG00000035885	chr19	7289648	7292106	-	545	-2.862	-1
Psmg1	ENSMUSG00000022913	chr16	96201540	96212567	-	1344	-2.862	-1
Chrac1	ENSMUSG00000068391	chr15	72920822	72927984	+	2353	-2.862	-1
Atp5k	ENSMUSG00000050856	chr5	108862263	108863467	-	616	-2.862	-1
Sys1	ENSMUSG00000045503	chr2	164282464	164305138	+	4958	-2.865	-1
0610011L14Rik	ENSMUSG00000027628	chr2	156373320	156394708	+	3615	-2.869	-1
9130011J15Rik	ENSMUSG00000044600	chr8	75089087	75094987	-	3266	-2.87	-1
5930434B04Rik	ENSMUSG00000015488	chr2	26865446	26876609	+	4693	-2.87	-1
Abhd1	ENSMUSG00000006638	chr5	31252439	31257464	+	1758	-2.873	-1
Ccdc28a	ENSMUSG00000059554	chr10	17933482	17954804	-	2964	-2.874	-1
Rnf167	ENSMUSG00000040746	chr11	70460737	70464923	+	3539	-2.874	-1
Mpg	ENSMUSG00000020287	chr11	32126505	32132700	+	3752	-2.874	-1
Mrpl24	ENSMUSG00000019710	chr3	87723428	87727594	+	2255	-2.875	-1
Cand2	ENSMUSG00000030319	chr6	115724556	115754911	+	4810	-2.875	-1
Tmem41a	ENSMUSG00000022856	chr16	21934400	21947617	-	1354	-2.875	-1
Atf5	ENSMUSG00000038539	chr7	52067627	52072028	-	1992	-2.875	-1
Galt	ENSMUSG00000036073	chr4	41702101	41705568	+	2471	-2.875	-1
Ubtd1	ENSMUSG00000025171	chr19	42056253	42109131	+	1527	-2.876	-1
Trmt112	ENSMUSG00000038812	chr19	6984188	6985539	+	1233	-2.876	-1
Cyhr1	ENSMUSG00000053929	chr15	76473825	76490547	-	6210	-2.876	-1
Phldb3	ENSMUSG00000074277	chr7	25396347	25414307	+	2273	-2.877	-1
Znhit3	ENSMUSG00000020526	chr11	84724452	84729868	-	2136	-2.879	-1
Susd2	ENSMUSG00000006342	chr10	75099364	75106753	-	3255	-2.88	-1
Cpt1c	ENSMUSG00000007783	chr7	52214743	52230184	-	2756	-2.881	-1
Slc15a2	ENSMUSG00000022899	chr16	36750264	36785048	-	7360	-2.883	-1
Mblac2	ENSMUSG00000051098	chr13	81850416	81892274	+	3881	-2.885	-1
Chpf2	ENSMUSG00000038181	chr5	24092559	24100213	+	5371	-2.887	-1
Acp6	ENSMUSG00000028093	chr3	96962700	96980497	+	4871	-2.887	-1
Gnb1l	ENSMUSG00000000884	chr16	18498861	18566772	+	4164	-2.887	-1
Pfkm	ENSMUSG00000033065	chr15	97923020	97962878	+	3051	-2.888	-1
Josd2	ENSMUSG00000038695	chr7	51723350	51727022	+	2466	-2.889	-1
Enpp5	ENSMUSG00000023960	chr17	44215762	44223516	+	4858	-2.892	-1
Zfp775	ENSMUSG00000007216	chr6	48563179	48573226	+	4083	-2.892	-1

1700001C19Rik	ENSMUSG00000047150	chr17	47549683	47574325	-	1896	-2.892	-1
Ttc6	ENSMUSG00000046782	chr12	58665100	58838915	+	6682	-2.893	-1
Spsb3	ENSMUSG00000024160	chr17	25023588	25029097	+	2719	-2.893	-1
Idh2	ENSMUSG00000030541	chr7	87239732	87260278	-	2588	-2.895	-1
Fam108a	ENSMUSG00000003346	chr10	80046394	80053086	-	1476	-2.895	-1
Dpm2	ENSMUSG00000026810	chr2	32426378	32429099	+	1971	-2.896	-1
Gata3	ENSMUSG00000015619	chr2	9778705	9811661	-	3978	-2.897	-1
Aldh1a7	ENSMUSG00000024747	chr19	20767443	20802052	-	2066	-2.897	-1
Cys1	ENSMUSG00000062563	chr12	25350703	25366678	-	2646	-2.898	-1
Plin3	ENSMUSG00000024197	chr17	56418385	56429934	-	2159	-2.898	-1
Telo2	ENSMUSG00000024170	chr17	25236514	25252912	-	8007	-2.9	-1
Rnf220	ENSMUSG00000028677	chr4	116944068	117169657	-	8491	-2.902	-1
Vars2	ENSMUSG00000038838	chr17	35792579	35804537	-	5460	-2.903	-1
Sbp	ENSMUSG00000024128	chr17	24079342	24087147	+	871	-2.904	-1
Mpped2	ENSMUSG00000016386	chr2	106533426	106708513	+	3425	-2.905	-1
Ticam1	ENSMUSG00000047123	chr17	56408742	56416209	-	2992	-2.906	-1
Fadd	ENSMUSG00000031077	chr7	151763223	151768368	-	3903	-2.906	-1
Coq10a	ENSMUSG00000039914	chr10	127800153	127807088	-	2502	-2.908	-1
Ece2	ENSMUSG00000022842	chr16	20611666	20646558	+	6547	-2.909	-1
Calml4	ENSMUSG00000032246	chr9	62705911	62723725	+	861	-2.909	-1
Rabggta	ENSMUSG00000040472	chr14	56334710	56341094	-	2630	-2.909	-1
Tor1a	ENSMUSG00000026849	chr2	30816147	30823453	-	2002	-2.911	-1
Acaa1a	ENSMUSG00000036138	chr9	119248794	119259417	+	4414	-2.911	-1
Wrb	ENSMUSG00000023147	chr16	96367014	96379459	+	4261	-2.912	-1
Zfp688	ENSMUSG00000045251	chr7	134562481	134565582	-	2734	-2.914	-1
Osbpl7	ENSMUSG00000038534	chr11	96911942	96930218	+	5938	-2.915	-1
Flywch2	ENSMUSG00000023911	chr17	23913883	23923048	-	996	-2.915	-1
D330023K18Rik	ENSMUSG00000087269	chr2	31001977	31007811	-	1249	-2.917	-1
Dnpep	ENSMUSG00000026209	chr1	75304471	75314212	-	3204	-2.917	-1
P2ry2	ENSMUSG00000032860	chr7	108145086	108160505	-	3000	-2.918	-1
Anapc13	ENSMUSG00000035048	chr9	102528626	102536571	+	405	-2.918	-1
E2f2	ENSMUSG00000018983	chr4	135728309	135751972	+	4882	-2.918	-1
Mospd3	ENSMUSG00000037221	chr5	138037873	138042286	-	1651	-2.919	-1
Apoc1	ENSMUSG00000040564	chr7	20274833	20278007	-	537	-2.919	-1
Mrpl38	ENSMUSG00000020775	chr11	115993131	116000182	-	2550	-2.92	-1
Ube2m	ENSMUSG00000005575	chr7	13620587	13623622	-	2167	-2.92	-1
Gramd3	ENSMUSG00000001700	chr18	56559991	56663446	+	3013	-2.92	-1
Ech1	ENSMUSG00000053898	chr7	29610236	29617266	+	2284	-2.921	-1
Map3k9	ENSMUSG00000042724	chr12	82815937	82882157	-	10619	-2.922	-1
Tceb2	ENSMUSG00000055839	chr17	23961707	23965958	-	494	-2.923	-1

Vwa1	ENSMUSG00000042116	chr4	155142258	155148807	-	3623	-2.923	-1
Olfml1	ENSMUSG00000051041	chr7	114710960	114734608	+	1685	-2.924	-1
Tbc1d16	ENSMUSG00000039976	chr11	119004359	119089813	-	7119	-2.925	-1
2310003H01Rik	ENSMUSG00000025384	chr11	120230369	120240078	-	5212	-2.927	-1
Sf3b5	ENSMUSG00000078348	chr10	12728256	12728988	+	733	-2.927	-1
Rps5	ENSMUSG00000012848	chr7	13507639	13512035	+	1734	-2.929	-1
Pttg1	ENSMUSG00000020415	chr11	43233752	43239753	-	2000	-2.929	-1
Sncg	ENSMUSG00000023064	chr14	35183460	35187975	-	852	-2.931	-1
Lcmt2	ENSMUSG00000074890	chr2	120954043	120966434	-	3701	-2.933	-1
Gm15564	ENSMUSG00000086324	chr16	35966838	35983316	-	821	-2.934	-1
Trappc5	ENSMUSG00000040236	chr8	3676299	3681255	+	2546	-2.934	-1
Dnajc10	ENSMUSG00000027006	chr2	80155623	80194200	+	5004	-2.934	-1
Aimp2	ENSMUSG00000029610	chr5	144663573	144670716	-	1654	-2.935	-1
Dnalc4	ENSMUSG00000022420	chr15	79591883	79608279	-	2340	-2.935	-1
Yipf3	ENSMUSG00000071074	chr17	46385029	46389486	+	2693	-2.936	-1
Wdr83	ENSMUSG00000005150	chr8	87598934	87605205	-	2818	-2.936	-1
Dkkl1	ENSMUSG00000030792	chr7	52462895	52467253	-	903	-2.937	-1
Gm16292	ENSMUSG00000086515	chr2	70341490	70347256	-	608	-2.939	-1
Slc46a1	ENSMUSG00000020829	chr11	78279199	78285447	+	1978	-2.94	-1
Oas1c	ENSMUSG00000001166	chr5	121250102	121262523	-	2574	-2.942	-1
Gm15417	ENSMUSG00000074466	chr3	89195772	89202701	+	1669	-2.942	-1
Adhfe1	ENSMUSG00000025911	chr1	9538029	9568051	+	3326	-2.943	-1
2410016O06Rik	ENSMUSG00000046791	chr12	85291558	85293903	+	2346	-2.943	-1
Gm13648	ENSMUSG00000086549	chr4	155426824	155433911	-	1536	-2.943	-1
Eif3f	ENSMUSG00000031029	chr7	116077931	116086465	+	2891	-2.945	-1
Tmem134	ENSMUSG00000024845	chr19	4125934	4132307	+	4601	-2.946	-1
2310035K24Rik	ENSMUSG00000068264	chr2	131032814	131039250	+	1660	-2.947	-1
Zbtb22	ENSMUSG00000051390	chr17	34052849	34056269	+	2672	-2.948	-1
Fam134a	ENSMUSG00000049339	chr1	75139360	75144483	+	2557	-2.949	-1
Mif4gd	ENSMUSG00000020743	chr11	115469232	115474283	-	2010	-2.949	-1
Ucp2	ENSMUSG00000033685	chr7	107641846	107650529	+	6991	-2.952	-1
Pick1	ENSMUSG00000068206	chr15	79059603	79087104	+	7770	-2.952	-1
Vars	ENSMUSG00000007029	chr17	35137932	35153267	+	5611	-2.954	-1
Mgst3	ENSMUSG00000026688	chr1	169302097	169323972	-	1097	-2.954	-1
Gjb2	ENSMUSG00000046352	chr14	57717437	57723539	-	2406	-2.954	-1

Scnm1	ENSMUSG00000092607	chr3	94933641	94937934	-	1703	-2.954	-1
Glrx5	ENSMUSG00000021102	chr12	106270899	106279120	+	930	-2.955	-1
Wtip	ENSMUSG00000036459	chr7	34894562	34918287	-	2233	-2.956	-1
Nubp2	ENSMUSG00000039183	chr17	25019556	25023295	-	1430	-2.958	-1
Psd	ENSMUSG00000037126	chr19	46386577	46401646	-	4098	-2.959	-1
Brms1	ENSMUSG00000080268	chr19	5041393	5049911	+	1997	-2.959	-1
Tfpt	ENSMUSG00000006335	chr7	3571926	3581531	-	1197	-2.962	-1
Hist1h2ac	ENSMUSG00000069270	chr13	23773336	23775817	-	2482	-2.964	-1
Gpx4	ENSMUSG00000075706	chr10	79509911	79519184	+	1675	-2.965	-1
6230427J02Rik	ENSMUSG00000042106	chr9	107886558	107888210	-	1152	-2.965	-1
Grhl2	ENSMUSG00000022286	chr15	37162791	37293324	+	5506	-2.969	-1
Zfp523	ENSMUSG00000024220	chr17	28314152	28342831	+	3991	-2.97	-1
Cldn1	ENSMUSG00000022512	chr16	26356728	26371927	-	3247	-2.97	-1
Actr5	ENSMUSG00000037761	chr2	158450624	158464947	+	4186	-2.97	-1
Edf1	ENSMUSG00000015092	chr2	25413367	25417602	+	1317	-2.975	-1
Slc9a8	ENSMUSG00000039463	chr2	167247212	167302500	+	9095	-2.977	-1
Nmb	ENSMUSG00000025723	chr7	88047113	88049962	-	793	-2.979	-1
Rpl19	ENSMUSG00000017404	chr11	97888024	97891806	+	1022	-2.98	-1
Cdk5rap1	ENSMUSG00000027487	chr2	154161116	154198746	-	3434	-2.981	-1
Slc52a2	ENSMUSG00000022560	chr15	76369373	76372560	+	2700	-2.981	-1
Cdc34	ENSMUSG00000020307	chr10	79144940	79151139	+	1262	-2.982	-1
Snx22	ENSMUSG00000039452	chr9	65912983	65917538	-	2555	-2.983	-1
Aars2	ENSMUSG00000023938	chr17	45643790	45657791	+	3348	-2.984	-1
Atp1a2	ENSMUSG00000007097	chr1	174201840	174228195	-	6782	-2.985	-1
Ighv1-81	ENSMUSG00000076739	chr12	117158490	117158923	-	351	-2.985	-1
Dhps	ENSMUSG00000060038	chr8	87595656	87599061	+	2324	-2.987	-1
Mtch1	ENSMUSG00000024012	chr17	29469017	29484849	-	2878	-2.988	-1
Pebp1	ENSMUSG00000032959	chr5	117732660	117737634	-	1771	-2.988	-1
Ubb	ENSMUSG00000019505	chr11	62364673	62366715	+	1675	-2.989	-1
Dnlz	ENSMUSG00000075467	chr2	26203641	26207630	-	2916	-2.99	-1
Urm1	ENSMUSG00000069020	chr2	29675595	29700522	+	4275	-2.991	-1
Srrd	ENSMUSG00000029346	chr5	112766412	112772060	-	1638	-2.991	-1
Gm4987	ENSMUSG00000063816	chrX	43809018	43809523	-	506	-2.991	-1
Unc5a	ENSMUSG00000025876	chr13	55050772	55107379	+	4168	-2.992	-1
Mknk2	ENSMUSG00000020190	chr10	80128077	80139038	-	3434	-2.993	-1
Churc1	ENSMUSG00000090258	chr12	77866525	77884167	+	5693	-2.994	-1
Gm9755	ENSMUSG00000030735	chr8	70038131	70039489	-	1359	-2.995	-1
Gm5436	ENSMUSG00000042962	chr12	85599282	85599923	-	642	-2.995	-1
Mrpl37	ENSMUSG00000028622	chr4	106728479	106739473	-	4460	-2.996	-1
Psph	ENSMUSG00000029446	chr5	130271434	130293129	-	1818	-2.997	-1
Shmt1	ENSMUSG00000020534	chr11	60601606	60625220	-	3883	-2.997	-1

Cyp27a1	ENSMUSG00000026170	chr1	74760148	74784464	+	1890	-2.998	-1
Dpp3	ENSMUSG00000063904	chr19	4907229	4928287	-	2683	-2.998	-1
Sdf4	ENSMUSG00000029076	chr4	155366981	155387719	+	6115	-2.999	-1
Fbxw5	ENSMUSG00000015095	chr2	25356270	25360991	+	3194	-2.999	-1
Ufc1	ENSMUSG00000062963	chr1	173218694	173225155	-	2070	-3	-1
Gm10288	ENSMUSG00000070343	chr3	146501775	146502311	-	537	-3.001	-1
Spryd4	ENSMUSG00000051346	chr10	127646964	127648850	-	1570	-3.003	-1
Efhd1	ENSMUSG00000026255	chr1	89160938	89207414	+	2125	-3.004	-1
Bscl2	ENSMUSG00000071657	chr19	8911957	8923173	+	5897	-3.007	-1
Fbxo31	ENSMUSG00000052934	chr8	124073350	124074865	-	1516	-3.007	-1
Gm8841	ENSMUSG00000091957	chr8	108109177	108110127	-	951	-3.007	-1
Hspbp1	ENSMUSG00000063802	chr7	4612123	4636565	-	1488	-3.009	-1
BC029722	ENSMUSG00000074649	chr2	155643466	155645162	-	1615	-3.012	-1
Zfp13	ENSMUSG00000062012	chr17	23712811	23736454	-	2407	-3.013	-1
Ehhadh	ENSMUSG00000022853	chr16	21761360	21787917	-	3018	-3.013	-1
2310014L17Rik	ENSMUSG00000033967	chr7	13512765	13516421	+	3239	-3.014	-1
Paqr3	ENSMUSG00000055725	chr5	97511348	97540615	-	7566	-3.014	-1
Ccnd3	ENSMUSG00000034165	chr17	47642000	47736632	+	2255	-3.014	-1
Lrrc61	ENSMUSG00000073096	chr6	48504795	48520721	+	2913	-3.015	-1
Polr2f	ENSMUSG00000033020	chr15	78971781	78982204	+	543	-3.015	-1
Pycard	ENSMUSG00000030793	chr7	135135617	135137381	-	1405	-3.016	-1
Napa	ENSMUSG00000006024	chr7	16683807	16703324	+	3786	-3.017	-1
Sftpc	ENSMUSG00000022097	chr14	70920748	70923888	-	804	-3.018	-1
Rfng	ENSMUSG00000025158	chr11	120642060	120645521	-	2489	-3.019	-1
Hes6	ENSMUSG00000067071	chr1	93308060	93310615	-	1808	-3.022	-1
Ppox	ENSMUSG00000062729	chr1	173206121	173211317	-	4515	-3.024	-1
Ces2g	ENSMUSG00000031877	chr8	107485618	107493437	+	2859	-3.026	-1
Cdc42ep2	ENSMUSG00000045664	chr19	5917561	5918995	-	1435	-3.028	-1
Uap1l1	ENSMUSG00000026956	chr2	25215409	25221202	-	4071	-3.028	-1
Gm5321	ENSMUSG00000064231	chr7	5970624	5971070	+	447	-3.029	-1
Mus81	ENSMUSG00000024906	chr19	5482345	5488402	-	3567	-3.03	-1
Myo5c	ENSMUSG00000033590	chr9	75079821	75153258	+	6733	-3.032	-1
Depdc5	ENSMUSG00000037426	chr5	33206350	33336882	+	11772	-3.032	-1
Sgsm2	ENSMUSG00000038351	chr11	74662763	74710562	-	6884	-3.032	-1
Cds1	ENSMUSG00000029330	chr5	102194149	102252877	+	3939	-3.032	-1
Atg16l2	ENSMUSG00000047767	chr7	108437906	108450765	-	3881	-3.033	-1
Cox6a1	ENSMUSG00000041697	chr5	115795651	115798990	-	702	-3.033	-1
Nle1	ENSMUSG00000020692	chr11	82714270	82721913	-	3233	-3.034	-1
Mpdu1	ENSMUSG00000018761	chr11	69470199	69476144	-	2420	-3.034	-1
Ddit4l	ENSMUSG00000046818	chr3	137284576	137291297	+	2916	-3.034	-1

Rrp9	ENSMUSG00000041506	chr9	106378294	106387755	+	2788	-3.035	-1
Osgin1	ENSMUSG00000074063	chr8	121958024	121970156	+	2674	-3.035	-1
Eef2k	ENSMUSG00000035064	chr7	127986345	128050733	+	7166	-3.036	-1
Lman2	ENSMUSG00000021484	chr13	55445194	55464144	-	4315	-3.037	-1
H2-K1	ENSMUSG00000061232	chr17	34132962	34137278	-	2608	-3.037	-1
Mob2	ENSMUSG00000025147	chr7	149194441	149246939	-	2204	-3.039	-1
Slc27a3	ENSMUSG00000027932	chr3	90189161	90193860	-	2569	-3.039	-1
Klhdc7a	ENSMUSG00000078234	chr4	139516135	139523941	-	7807	-3.039	-1
Reep2	ENSMUSG00000038555	chr18	35000312	35007106	+	1846	-3.039	-1
2810001 G20Rik	ENSMUSG00000087497	chr11	63892986	63896761	+	1057	-3.041	-1
Gfod2	ENSMUSG00000013150	chr8	108237754	108282564	-	4608	-3.041	-1
Trnp1	ENSMUSG00000056596	chr4	133047015	133054465	-	1654	-3.042	-1
Elof1	ENSMUSG00000013822	chr9	21917433	21921592	-	1092	-3.042	-1
Pld3	ENSMUSG00000003363	chr7	28317019	28338237	-	2924	-3.043	-1
Tbcc	ENSMUSG00000036430	chr17	47027570	47029412	+	1843	-3.043	-1
Rabl5	ENSMUSG00000007987	chr5	137384020	137389114	+	911	-3.043	-1
Chst7	ENSMUSG00000037347	chrX	19636686	19674647	+	2206	-3.044	-1
4930412 M03Rik	ENSMUSG00000051008	chr11	59925721	59928732	-	1111	-3.044	-1
4930515 G01Rik	ENSMUSG00000092183	chr5	115223746	115224992	-	1247	-3.044	-1
Gtpbp1	ENSMUSG00000042535	chr15	79521320	79551908	+	3403	-3.045	-1
Nans	ENSMUSG00000028334	chr4	46502120	46516504	+	4581	-3.046	-1
Ercc2	ENSMUSG00000030400	chr7	19967359	19981043	+	4170	-3.046	-1
Ass1	ENSMUSG00000076441	chr2	31325727	31376192	+	2307	-3.048	-1
Tmem2 14	ENSMUSG00000038828	chr5	31171952	31181564	+	6024	-3.049	-1
Mrpl34	ENSMUSG00000034880	chr8	73988825	73989651	+	645	-3.05	-1
D4Bwg0 951e	ENSMUSG00000048706	chr4	80556550	80601532	+	3066	-3.051	-1
Sco2	ENSMUSG00000091780	chr15	89202113	89202880	-	768	-3.052	-1
Rps11	ENSMUSG00000003429	chr7	52377757	52379766	-	610	-3.052	-1
Csdc2	ENSMUSG00000042109	chr15	81767189	81781371	+	4623	-3.052	-1
Tmed9	ENSMUSG00000058569	chr13	55694313	55699027	+	1506	-3.053	-1
Rpl9-ps6	ENSMUSG00000062456	chr19	32540375	32541047	-	673	-3.053	-1
Rhot2	ENSMUSG00000025733	chr17	25975402	25981796	-	4444	-3.053	-1
Clic5	ENSMUSG00000023959	chr17	44325521	44417117	+	5907	-3.053	-1
Erbb3	ENSMUSG00000018166	chr10	128004579	128026708	-	6016	-3.053	-1
Mogs	ENSMUSG00000030036	chr6	83065490	83068892	+	2892	-3.054	-1
9230102 O04Rik	ENSMUSG00000079602	chr2	9804668	9811167	-	935	-3.054	-1

Ppib	ENSMUSG00000032383	chr9	65907976	65914436	+	960	-3.055	-1
Atox1	ENSMUSG00000018585	chr11	55260143	55274741	-	541	-3.055	-1
Pdp2	ENSMUSG00000048371	chr8	107115368	107120736	+	3485	-3.055	-1
Wdr34	ENSMUSG00000039715	chr2	29887066	29904401	-	2359	-3.056	-1
Cib1	ENSMUSG00000030538	chr7	87372042	87377699	-	2052	-3.057	-1
1810062G17Rik	ENSMUSG00000027713	chr3	36375057	36380898	+	390	-3.057	-1
Atp13a1	ENSMUSG00000031862	chr8	72315062	72331648	+	3926	-3.057	-1
Fam116b	ENSMUSG00000015377	chr15	89012647	89026905	-	4607	-3.059	-1
Slamf9	ENSMUSG00000026548	chr1	174405489	174408706	+	1313	-3.062	-1
Tpd52l1	ENSMUSG00000000296	chr10	31052186	31165727	-	1282	-3.062	-1
Ccdc17	ENSMUSG00000034035	chr4	116269259	116272871	+	2634	-3.063	-1
Fam98c	ENSMUSG00000030590	chr7	29937530	29941253	-	2712	-3.066	-1
Tymp	ENSMUSG00000022615	chr15	89203876	89207179	-	1416	-3.066	-1
Gm53	ENSMUSG00000078706	chr11	96112419	96126182	+	3980	-3.066	-1
Eri3	ENSMUSG00000033423	chr4	117222970	117346902	+	4855	-3.067	-1
Taf10	ENSMUSG00000043866	chr7	112887907	112892875	-	4584	-3.067	-1
Dph1	ENSMUSG00000078789	chr11	74991145	75004743	-	2690	-3.068	-1
Tle2	ENSMUSG00000034771	chr10	81037306	81053590	+	6221	-3.069	-1
2210408F21Rik	ENSMUSG00000087380	chr6	31170351	31287404	+	1476	-3.07	-1
Btbd6	ENSMUSG00000002803	chr12	114214693	114217141	+	2088	-3.072	-1
Mocs3	ENSMUSG00000074576	chr2	168056122	168058094	+	1973	-3.073	-1
2810422J05Rik	ENSMUSG00000055553	chr8	73032171	73051855	-	3176	-3.075	-1
Fam83h	ENSMUSG00000046761	chr15	75831523	75844766	-	4627	-3.075	-1
Trmt61a	ENSMUSG00000060950	chr12	112916316	112922113	+	2433	-3.08	-1
Qdpr	ENSMUSG00000015806	chr5	45825271	45841474	-	1914	-3.08	-1
Mrps7	ENSMUSG00000046756	chr11	115465239	115469350	+	2072	-3.082	-1
F8a	ENSMUSG00000078317	chrX	70473645	70476134	+	2490	-3.084	-1
2610002J02Rik	ENSMUSG00000073684	chr4	154623911	154630793	+	1740	-3.085	-1
Gpaa1	ENSMUSG00000022561	chr15	76161661	76165337	+	2846	-3.085	-1
Gps1	ENSMUSG00000025156	chr11	120645586	120650416	+	2879	-3.087	-1
Fem1a	ENSMUSG00000043683	chr17	56396216	56403031	+	6816	-3.087	-1
Pigt	ENSMUSG00000017721	chr2	164323020	164333801	+	3856	-3.087	-1
C1qtnf2	ENSMUSG00000046491	chr11	43287778	43305027	+	1308	-3.089	-1
Npm3	ENSMUSG00000056209	chr19	45822224	45824081	-	1187	-3.089	-1
Chia	ENSMUSG00000062778	chr3	105916147	105935038	+	1903	-3.091	-1
Vps52	ENSMUSG00000024319	chr17	34092757	34103929	+	3876	-3.092	-1
Spag1	ENSMUSG00000037617	chr15	36109123	36165365	+	3814	-3.093	-1
Gtpbp3	ENSMUSG00000007610	chr8	74012002	74023482	+	3984	-3.093	-1

Taldo1	ENSMUSG00000025503	chr7	148578098	148588867	+	1998	-3.094	-1
Eps8l2	ENSMUSG00000025504	chr7	148524779	148548919	+	4370	-3.096	-1
Lypd3	ENSMUSG00000057454	chr7	25421589	25426135	+	1627	-3.097	-1
B4galt4	ENSMUSG00000022793	chr16	38742377	38769162	+	3042	-3.098	-1
Nxt1	ENSMUSG00000036992	chr2	148498337	148501763	+	1116	-3.099	-1
Hmbs	ENSMUSG00000032126	chr9	44144433	44152311	-	1755	-3.099	-1
Tbc1d10a	ENSMUSG00000034412	chr11	4086792	4115508	+	2733	-3.1	-1
Gm5745	ENSMUSG00000066478	chr9	73023234	73023548	-	315	-3.1	-1
Hist1h2bk	ENSMUSG00000062727	chr13	22127690	22128168	+	479	-3.1	-1
Tmem53	ENSMUSG00000048772	chr4	116924556	116941187	+	1713	-3.1	-1
Cnp	ENSMUSG00000006782	chr11	100436218	100453043	+	3639	-3.1	-1
Retnla	ENSMUSG00000061100	chr16	48842665	48844574	+	598	-3.101	-1
Rps18	ENSMUSG00000008668	chr17	34088944	34092946	-	2491	-3.102	-1
Atp5sl	ENSMUSG00000057229	chr7	26404433	26410570	+	2916	-3.103	-1
Mrpl4	ENSMUSG00000003299	chr9	20807181	20813279	+	1285	-3.105	-1
Sat2	ENSMUSG00000069835	chr11	69435526	69437372	+	1424	-3.106	-1
Erp29	ENSMUSG00000029616	chr5	121878599	121902515	-	9060	-3.107	-1
Pik3c2b	ENSMUSG00000026447	chr1	134942244	135005264	+	9237	-3.108	-1
Pdcd2l	ENSMUSG00000002635	chr7	34969518	34981680	-	1315	-3.109	-1
Lrfn3	ENSMUSG00000036957	chr7	31140533	31147791	-	2861	-3.109	-1
2310016M24Rik	ENSMUSG00000023020	chr15	99556049	99558567	+	723	-3.112	-1
Gm11175	ENSMUSG00000080058	chr8	73223437	73223979	-	543	-3.115	-1
Mrps11	ENSMUSG00000030611	chr7	85928005	85937875	+	2172	-3.115	-1
Cdk18	ENSMUSG00000026437	chr1	134010127	134036262	-	3043	-3.116	-1
Lipe	ENSMUSG00000003123	chr7	26164546	26183729	-	4518	-3.116	-1
Phb2	ENSMUSG00000004264	chr6	124662340	124666968	+	3734	-3.117	-1
Ropn1l	ENSMUSG00000022236	chr15	31370965	31383444	-	910	-3.117	-1
Mettl22	ENSMUSG00000039345	chr16	8470881	8490777	+	2665	-3.118	-1
Pars2	ENSMUSG00000043572	chr4	106323674	106327887	+	3094	-3.119	-1
Tmem141	ENSMUSG00000026939	chr2	25475587	25477525	-	1122	-3.12	-1
Rnasek	ENSMUSG00000040904	chr11	70048708	70053344	-	1978	-3.12	-1
Klhdc8b	ENSMUSG00000032609	chr9	108349989	108354708	-	1897	-3.12	-1
Snx8	ENSMUSG00000029560	chr5	140816258	140865216	-	2671	-3.121	-1
Hectd3	ENSMUSG00000046861	chr4	116667922	116677882	+	4901	-3.122	-1
Pi16	ENSMUSG00000024011	chr17	29455822	29466358	+	3987	-3.123	-1
A430046D13Rik	ENSMUSG00000085446	chr10	127936420	127941298	+	1997	-3.125	-1
AI837181	ENSMUSG00000047423	chr19	5425144	5427313	+	1542	-3.127	-1

Jund	ENSMUSG00000071076	chr8	73221638	73224515	+	2878	-3.127	-1
Lars2	ENSMUSG00000035202	chr9	123276058	123371782	+	3881	-3.128	-1
Tmem192	ENSMUSG00000025521	chr8	67471084	67492933	+	1123	-3.129	-1
Siva1	ENSMUSG00000064326	chr12	113883039	113887363	+	782	-3.129	-1
Syngr2	ENSMUSG00000048277	chr11	117670982	117675597	+	1776	-3.129	-1
Mx1	ENSMUSG00000000386	chr16	97668642	97684514	-	3646	-3.129	-1
Gng7	ENSMUSG00000048240	chr10	80411369	80477690	-	3786	-3.13	-1
Fbxl6	ENSMUSG00000022559	chr15	76366160	76369176	-	1712	-3.13	-1
Sort1	ENSMUSG00000068747	chr3	108087009	108164429	+	7560	-3.131	-1
Fdxr	ENSMUSG00000018861	chr11	115129338	115138364	-	2806	-3.132	-1
Phkg2	ENSMUSG00000030815	chr7	134716854	134726819	+	3998	-3.133	-1
Ifrd2	ENSMUSG00000010048	chr9	107490049	107495369	+	1980	-3.135	-1
Zfp784	ENSMUSG00000043290	chr7	4986041	4990048	-	2153	-3.135	-1
Gm6728	ENSMUSG00000091408	chr6	136434643	136438660	-	1098	-3.136	-1
Rnh1	ENSMUSG00000038650	chr7	148346227	148358750	-	1911	-3.136	-1
Atp6v0b	ENSMUSG00000033379	chr4	117556931	117559938	-	2051	-3.136	-1
Synm	ENSMUSG00000030554	chr7	74875047	74904628	-	7931	-3.137	-1
Agpat1	ENSMUSG00000034254	chr17	34741207	34750394	+	3001	-3.138	-1
Nxph3	ENSMUSG00000046719	chr11	95371159	95375884	-	2099	-3.138	-1
Spint1	ENSMUSG00000027315	chr2	119063098	119075263	+	2767	-3.139	-1
Ndufb6	ENSMUSG00000071014	chr4	40217624	40226454	-	659	-3.14	-1
Fam109a	ENSMUSG00000044134	chr5	122299037	122304608	+	2335	-3.141	-1
Ocel1	ENSMUSG00000002396	chr8	73895197	73903260	+	7133	-3.142	-1
Bcs1l	ENSMUSG00000026172	chr1	74634863	74639017	+	2192	-3.146	-1
1110008F13Rik	ENSMUSG00000027637	chr2	156688864	156699299	+	2871	-3.146	-1
1110017D15Rik	ENSMUSG00000028441	chr4	41452042	41464366	-	1421	-3.148	-1
Iglc2	ENSMUSG00000076937	chr16	19198494	19200444	-	606	-3.148	-1
Rab24	ENSMUSG00000034789	chr13	55420584	55423341	-	1865	-3.151	-1
Mrpl20	ENSMUSG00000029066	chr4	155177645	155183885	+	1084	-3.151	-1
Ndufa12	ENSMUSG00000020022	chr10	93661700	93684188	+	1334	-3.152	-1
Cd209f	ENSMUSG00000051906	chr8	4102787	4105843	-	1536	-3.154	-1
Gas6	ENSMUSG00000031451	chr8	13465374	13494535	-	2593	-3.155	-1
Ankrd54	ENSMUSG00000033055	chr15	78883524	78893323	-	4270	-3.155	-1
Zfp30	ENSMUSG00000047473	chr7	30569017	30579721	+	6078	-3.155	-1
Sssca1	ENSMUSG00000079478	chr19	5728088	5731732	-	3083	-3.158	-1
Sergef	ENSMUSG00000030839	chr7	53698538	53895177	-	2368	-3.16	-1
Fxc1	ENSMUSG00000089847	chr7	112788570	112804262	+	4087	-3.16	-1
Ppp1r16a	ENSMUSG00000033819	chr15	76502045	76525349	+	4119	-3.16	-1
Elmod3	ENSMUSG00000056698	chr6	72515916	72548407	-	3585	-3.161	-1

Gm3511	ENSMUSG00000069196	chr5	114217108	114217421	+	314	-3.161		-1
Narfl	ENSMUSG00000002280	chr17	25910721	25920277	+	3826	-3.161		-1
Cdc34-ps	ENSMUSG00000020870	chr11	94603096	94604347	+	1252	-3.164		-1
2310047M10Rik	ENSMUSG00000045176	chr11	68873277	68875078	+	1802	-3.164		-1
Sfxn2	ENSMUSG00000025036	chr19	46647855	46671388	+	3210	-3.165		-1
3300002A11Rik	ENSMUSG00000033731	chr12	100579364	100600037	+	726	-3.166		-1
Gm17530	ENSMUSG00000092099	chr9	86440289	86440402	-	114	-3.166		-1
Sepx1	ENSMUSG00000075705	chr17	24873587	24879723	+	1258	-3.167		-1
Pyroxd2	ENSMUSG00000060224	chr19	42800348	42827265	-	3119	-3.168		-1
Emg1	ENSMUSG00000004268	chr6	124654399	124662196	-	1404	-3.168		-1
Med11	ENSMUSG00000018923	chr11	70265421	70267229	+	1046	-3.168		-1
Pwwp2b	ENSMUSG00000060260	chr7	146434381	146453152	+	2640	-3.168		-1
Gm4950	ENSMUSG00000069379	chr18	52024918	52025535	-	618	-3.17		-1
Slc44a3	ENSMUSG00000039865	chr3	121162447	121235262	-	2559	-3.17		-1
Akr1b10	ENSMUSG00000061758	chr6	34334218	34346950	+	2635	-3.17		-1
Yars	ENSMUSG00000028811	chr4	128867004	128896851	+	5943	-3.173		-1
St5	ENSMUSG00000031024	chr7	116667425	116760661	-	4526	-3.174		-1
Phyh	ENSMUSG00000026664	chr2	4840065	4859776	+	1893	-3.174		-1
Dctn3	ENSMUSG00000028447	chr4	41661830	41670202	-	1042	-3.174		-1
Nfe2l3	ENSMUSG00000029832	chr6	51382669	51408767	+	2973	-3.176		-1
Ccl5	ENSMUSG00000035042	chr11	83339280	83344020	-	960	-3.176		-1
Dusp28	ENSMUSG00000047067	chr1	94803566	94805197	+	1491	-3.176		-1
Prr24	ENSMUSG00000091811	chr7	16857363	16859041	-	1679	-3.178		-1
C130074G19Rik	ENSMUSG00000039349	chr1	186695805	186707097	-	3035	-3.178		-1
Arfgap2	ENSMUSG00000027255	chr2	91105131	91117088	+	4129	-3.178		-1
Wdr6	ENSMUSG00000066357	chr9	108474644	108481070	-	4161	-3.178		-1
2900010M23Rik	ENSMUSG00000024208	chr17	27259608	27270861	-	837	-3.179		-1
5430437P03Rik	ENSMUSG00000031820	chr8	73920754	73928671	+	1592	-3.18		-1
Zbtb17	ENSMUSG00000006215	chr4	141000569	141023845	+	3877	-3.181		-1
Ifi27l1	ENSMUSG00000064215	chr12	104672421	104678449	+	1467	-3.183		-1
Mecr	ENSMUSG00000028910	chr4	131399385	131423701	+	3041	-3.183		-1
Fars2	ENSMUSG00000021420	chr13	36209280	36629461	+	2125	-3.184		-1

Arhgap28	ENSMUSG00000024043	chr17	68192053	68353460	-	5377	-3.186	-1
Klc4	ENSMUSG00000003546	chr17	46767574	46782104	-	2339	-3.186	-1
Ddx49	ENSMUSG00000057788	chr8	72816765	72826388	-	2928	-3.189	-1
Hdac5	ENSMUSG00000008855	chr11	102055746	102091480	-	8780	-3.189	-1
D2Wsu81e	ENSMUSG00000039660	chr2	30028967	30033979	-	3016	-3.189	-1
Slc38a10	ENSMUSG00000061306	chr11	119965274	120012660	-	6397	-3.191	-1
Clpb	ENSMUSG00000001829	chr7	108812147	108943974	+	9914	-3.191	-1
Ubl4	ENSMUSG00000015290	chrX	71611057	71614012	-	2799	-3.193	-1
Atp5d	ENSMUSG00000003072	chr10	79601377	79608563	+	1928	-3.194	-1
Ppan	ENSMUSG00000004100	chr9	20692619	20696622	+	2504	-3.196	-1
Dad1	ENSMUSG00000022174	chr14	54855154	54873779	-	3916	-3.197	-1
Gm684	ENSMUSG00000079559	chr9	51078656	51080522	-	465	-3.198	-1
A230051G13Rik	ENSMUSG00000049287	chr11	58968871	58977241	-	4885	-3.199	-1
Hras1	ENSMUSG00000025499	chr7	148375004	148379904	-	3733	-3.2	-1
Gm10146	ENSMUSG00000064317	chr10	77856059	77856497	+	439	-3.203	-1
Ormdl2	ENSMUSG00000025353	chr10	128254513	128258687	-	2055	-3.204	-1
Tbxa2r	ENSMUSG00000034881	chr10	80791477	80797916	+	1763	-3.204	-1
Decr2	ENSMUSG00000036775	chr17	26218156	26227274	-	2288	-3.208	-1
Ager	ENSMUSG00000015452	chr17	34734807	34737879	+	2139	-3.211	-1
Wdyhv1	ENSMUSG00000022359	chr15	57972963	57990207	+	1537	-3.211	-1
Fis1	ENSMUSG00000019054	chr5	137429145	137442104	+	1714	-3.212	-1
Pef1	ENSMUSG00000028779	chr4	129779802	129805379	+	1915	-3.213	-1
Rpl18	ENSMUSG00000059070	chr7	52970827	52976205	+	4042	-3.216	-1
2410006H16Rik	ENSMUSG00000086841	chr11	62416379	62418309	+	462	-3.219	-1
Rpl18a	ENSMUSG00000045128	chr8	73418621	73421342	-	1291	-3.22	-1
Psme1	ENSMUSG00000022216	chr14	56196960	56200366	+	2209	-3.22	-1
1700029I15Rik	ENSMUSG00000044916	chr2	92223075	92225865	+	644	-3.221	-1
Cenpv	ENSMUSG00000018509	chr11	62338448	62352763	-	1811	-3.222	-1
Zfp524	ENSMUSG00000051184	chr7	4967103	4970088	+	1110	-3.222	-1
Lypla2	ENSMUSG00000028670	chr4	135524139	135528541	-	2054	-3.223	-1
Mrpl48	ENSMUSG00000030706	chr7	107694265	107756811	-	6139	-3.224	-1
Igsf9	ENSMUSG00000037995	chr1	174411919	174429009	+	6612	-3.224	-1
2400001E08Rik	ENSMUSG00000030842	chr7	109054351	109060415	+	1184	-3.224	-1
Ntrk1	ENSMUSG00000028072	chr3	87582166	87599084	-	2588	-3.224	-1
Gm16740	ENSMUSG00000085137	chr3	95021379	95030580	+	956	-3.225	-1
5830415F09Rik	ENSMUSG00000028331	chr4	46389377	46402309	-	2271	-3.226	-1

Psmb4	ENSMUSG00000005779	chr3	94688015	94690883	-	1384	-3.227	-1
Wdr74	ENSMUSG00000042729	chr19	8810329	8815113	+	1203	-3.227	-1
Eif3k	ENSMUSG00000053565	chr7	29756392	29766883	-	1074	-3.227	-1
Elovl7	ENSMUSG00000021696	chr13	109004598	109077299	+	5435	-3.227	-1
Gm962	ENSMUSG00000049562	chr19	5568074	5571261	+	3092	-3.227	-1
Gm11547	ENSMUSG00000085604	chr11	100548731	100558834	+	1594	-3.23	-1
Gipc1	ENSMUSG00000019433	chr8	86176577	86188687	+	1615	-3.233	-1
Nudt8	ENSMUSG00000024869	chr19	4000580	4005816	+	4298	-3.234	-1
Sdf2	ENSMUSG00000002064	chr11	78059248	78068998	+	1515	-3.235	-1
Ergic3	ENSMUSG00000005881	chr2	155833781	155844015	+	2121	-3.236	-1
Pvrl2	ENSMUSG00000062300	chr7	20301993	20334922	-	3440	-3.236	-1
Fkbp8	ENSMUSG00000019428	chr8	73051623	73059227	+	4529	-3.236	-1
Lsm7	ENSMUSG00000035215	chr10	80315570	80317954	-	468	-3.237	-1
Glb1l2	ENSMUSG00000036395	chr9	26570629	26614053	-	5031	-3.24	-1
BC056474	ENSMUSG00000059355	chr8	87604639	87606238	+	1031	-3.241	-1
Polr2j	ENSMUSG00000039771	chr5	136592501	136598817	+	1106	-3.242	-1
Supt4h1	ENSMUSG00000020485	chr11	87551054	87557125	+	942	-3.242	-1
Gm11808	ENSMUSG00000068240	chr4	3900239	3900742	-	504	-3.242	-1
Nudt13	ENSMUSG00000021809	chr14	21113912	21136797	+	2523	-3.243	-1
Tubb2a	ENSMUSG00000058672	chr13	34166170	34169877	-	1584	-3.244	-1
Zfp707	ENSMUSG00000034429	chr15	75799553	75806298	+	3094	-3.245	-1
8430419L09Rik	ENSMUSG00000030207	chr6	135147995	135194973	+	5776	-3.246	-1
Gtpbp5	ENSMUSG00000039069	chr2	179805293	179820607	+	5949	-3.247	-1
Gsn	ENSMUSG00000026879	chr2	35111927	35163420	+	3136	-3.248	-1
Hexim2	ENSMUSG00000043372	chr11	102993743	103001190	+	2373	-3.248	-1
Spata2l	ENSMUSG00000033594	chr8	125754423	125760109	-	2845	-3.249	-1
Dedd2	ENSMUSG00000054499	chr7	25987859	26004878	-	1828	-3.249	-1
Bst2	ENSMUSG00000046718	chr8	74058163	74061336	-	764	-3.25	-1
Nat9	ENSMUSG00000015542	chr11	115044146	115049173	-	2066	-3.251	-1
Tbx2	ENSMUSG00000000093	chr11	85646053	85655450	+	3626	-3.252	-1
Timm50	ENSMUSG00000003438	chr7	29090535	29097091	-	2684	-3.252	-1
Mtx1	ENSMUSG00000064068	chr3	89013003	89031010	-	3584	-3.253	-1
Ndufs6	ENSMUSG00000021606	chr13	73457286	73465990	-	898	-3.253	-1
Nlrx1	ENSMUSG00000032109	chr9	44060796	44076682	-	3980	-3.253	-1
Mrps21	ENSMUSG00000054312	chr3	95666557	95675442	-	1282	-3.256	-1
Mypop	ENSMUSG00000048481	chr7	19576594	19587115	+	2175	-3.257	-1
Rab40c	ENSMUSG00000025730	chr17	26019059	26056672	-	3094	-3.257	-1
2610002I17Rik	ENSMUSG00000054836	chr9	110207696	110224596	+	2090	-3.257	-1
Recql5	ENSMUSG00000020752	chr11	115753909	115794791	-	6986	-3.258	-1
Rab1b	ENSMUSG00000024870	chr19	5099207	5107019	-	1858	-3.259	-1
Sepw1	ENSMUSG00000041571	chr7	16502557	16507751	-	736	-3.26	-1

Gpn2	ENSMUSG00000028848	chr4	133140277	133147650	+	1482	-3.26		-1
Id3	ENSMUSG00000007872	chr4	135699412	135701670	+	1791	-3.262		-1
Map3k10	ENSMUSG00000040390	chr7	28441394	28459617	-	4142	-3.262		-1
Uba7	ENSMUSG00000032596	chr9	107877836	107886391	+	6692	-3.263		-1
9330020H09Rik	ENSMUSG00000091050	chr15	98397755	98400014	+	2147	-3.264		-1
Wbscr16	ENSMUSG00000061979	chr5	134623924	134652644	-	2874	-3.264		-1
2310033P09Rik	ENSMUSG00000020441	chr11	59021823	59024240	+	1285	-3.267		-1
Ptgis	ENSMUSG00000017969	chr2	167017305	167066104	-	2567	-3.269		-1
Clstn1	ENSMUSG00000039953	chr4	148960577	149023008	+	4662	-3.271		-1
Solh	ENSMUSG00000037326	chr17	26095315	26122741	-	5111	-3.272		-1
Xab2	ENSMUSG00000019470	chr8	3608421	3621316	-	4685	-3.273		-1
Rps19-ps4	ENSMUSG00000072528	chr18	42412040	42412489	+	450	-3.274		-1
Ctu2	ENSMUSG00000049482	chr8	125000043	125008038	+	5164	-3.274		-1
Med29	ENSMUSG00000003444	chr7	29171165	29177727	-	1281	-3.274		-1
Atp5o	ENSMUSG00000022956	chr16	91684643	91931932	-	2259	-3.276		-1
Rnpepl1	ENSMUSG00000026269	chr1	94807982	94817415	+	2712	-3.276		-1
Maf1	ENSMUSG00000022553	chr15	76181724	76184810	+	2412	-3.277		-1
Fam203a	ENSMUSG00000022554	chr15	76199328	76201833	+	1959	-3.278		-1
2410017P09Rik	ENSMUSG00000073240	chr12	4234027	4236693	+	583	-3.281		-1
Ndufa2	ENSMUSG00000014294	chr18	36902020	36904202	-	529	-3.281		-1
Hax1	ENSMUSG00000027944	chr3	89799378	89802608	-	1118	-3.282		-1
D16H22S680E	ENSMUSG00000013539	chr16	18300918	18348196	-	7040	-3.285		-1
Akr1a1	ENSMUSG00000028692	chr4	116309115	116324285	-	2750	-3.285		-1
5430407P10Rik	ENSMUSG00000045319	chr2	6021071	6051257	-	2328	-3.285		-1
Ncaph2	ENSMUSG00000008690	chr15	89186150	89203257	+	4198	-3.286		-1
Fastk	ENSMUSG00000028959	chr5	23944258	23951105	-	4372	-3.289		-1
Gm16845	ENSMUSG00000087161	chr9	21875476	21890566	+	1415	-3.289		-1
Wnt10a	ENSMUSG00000026167	chr1	74838090	74850753	+	2468	-3.29		-1
Irf2bp1	ENSMUSG00000044030	chr7	19589414	19592110	+	2697	-3.295		-1
Hemk1	ENSMUSG00000032579	chr9	107230023	107240681	-	3059	-3.297		-1
Mea1	ENSMUSG00000002768	chr17	46818086	46820054	+	840	-3.299		-1
H2-Oa	ENSMUSG00000024334	chr17	34229285	34232179	+	1314	-3.299		-1
Srp14	ENSMUSG00000009549	chr2	118301586	118305447	-	1195	-3.299		-1
Zcchc17	ENSMUSG00000028772	chr4	129992627	130037190	-	2592	-3.3		-1

Pin1	ENSMUSG00000032171	chr9	20456574	20471028	+	3808	-3.3	-1
Hagh	ENSMUSG00000024158	chr17	24977088	25001395	+	1908	-3.304	-1
Pkn1	ENSMUSG00000057672	chr8	86193661	86223078	-	7391	-3.305	-1
Spr	ENSMUSG00000033735	chr6	85083672	85087760	-	1230	-3.306	-1
Itfg2	ENSMUSG00000001518	chr6	128359462	128374949	-	5069	-3.307	-1
2810008D09Rik	ENSMUSG00000086859	chr11	116938104	116940269	+	848	-3.312	-1
Cacnb1	ENSMUSG00000020882	chr11	97862822	97884348	-	4529	-3.313	-1
Sac3d1	ENSMUSG00000024790	chr19	6116004	6118586	-	1374	-3.317	-1
N4bp3	ENSMUSG00000001053	chr11	51456565	51464344	-	2728	-3.318	-1
Cuedc2	ENSMUSG00000036748	chr19	46404302	46413150	-	1941	-3.318	-1
Uck1	ENSMUSG00000002550	chr2	32110522	32115679	-	2751	-3.319	-1
Tlcd1	ENSMUSG00000019437	chr11	77990213	77995411	+	5009	-3.323	-1
Uqcrc1	ENSMUSG00000025651	chr9	108838661	108851685	+	1655	-3.324	-1
Tbc1d25	ENSMUSG00000039201	chrX	7731598	7753307	-	2794	-3.324	-1
Mrm1	ENSMUSG00000018405	chr11	84626563	84633017	-	2868	-3.325	-1
Fance	ENSMUSG00000007570	chr17	28450475	28463513	+	3183	-3.326	-1
Wdr18	ENSMUSG00000035754	chr10	79422897	79431991	+	2954	-3.327	-1
Pigv	ENSMUSG00000043257	chr4	133216302	133228562	-	4180	-3.328	-1
Slc4a3	ENSMUSG00000006576	chr1	75542841	75558747	+	8599	-3.329	-1
Dapk3	ENSMUSG00000034974	chr10	80646663	80655729	+	1483	-3.33	-1
Alg3	ENSMUSG00000033809	chr16	20605524	20610841	-	1451	-3.33	-1
Ndufv1	ENSMUSG00000037916	chr19	4007497	4012806	-	2521	-3.331	-1
Zmat5	ENSMUSG00000009076	chr11	4604681	4637672	+	1248	-3.331	-1
Pnkp	ENSMUSG00000002963	chr7	52112509	52118295	+	3133	-3.332	-1
Mfsd10	ENSMUSG00000001082	chr5	34976291	34979861	-	2752	-3.333	-1
Atp6v0d1	ENSMUSG00000013160	chr8	108048365	108089947	-	1793	-3.333	-1
Cecr5	ENSMUSG00000058979	chr6	120459512	120481337	-	3355	-3.333	-1
Wars	ENSMUSG00000021266	chr12	110098240	110132384	-	3447	-3.335	-1
Slc25a10	ENSMUSG00000025792	chr11	120353154	120360501	+	3455	-3.335	-1
Fam183b	ENSMUSG00000049154	chr11	58606299	58615462	-	567	-3.34	-1
Gm4799	ENSMUSG00000071151	chr10	82417089	82417745	+	657	-3.34	-1
Pygb	ENSMUSG00000033059	chr2	150612471	150657494	+	4809	-3.34	-1
Fibp	ENSMUSG00000024911	chr19	5460637	5465051	+	1209	-3.342	-1
Anxa13	ENSMUSG00000055114	chr15	58173429	58197682	-	951	-3.343	-1
Gdf10	ENSMUSG00000021943	chr14	34736773	34749700	+	3741	-3.343	-1
Ccdc85b	ENSMUSG00000042878	chr19	5453163	5457549	-	4387	-3.344	-1
Gaa	ENSMUSG00000025579	chr11	119129201	119146768	+	4783	-3.344	-1
Lrrc56	ENSMUSG00000038637	chr7	148380056	148395954	+	6068	-3.345	-1
Ndufb7	ENSMUSG00000033938	chr8	86090570	86095525	+	4522	-3.345	-1
Asns	ENSMUSG00000029752	chr6	7625169	7643254	-	2735	-3.346	-1

Qars	ENSMUSG00000032604	chr9	108410336	108418272	+	6200	-3.348	-1
Nhp2	ENSMUSG00000001056	chr11	51433237	51437216	+	1146	-3.348	-1
Cd59a	ENSMUSG00000032679	chr2	103935958	103955511	+	1811	-3.349	-1
Bphl	ENSMUSG00000038286	chr13	34129502	34165943	+	1237	-3.35	-1
Rps27	ENSMUSG00000090733	chr3	90016593	90017573	-	345	-3.35	-1
Tcf21	ENSMUSG00000045680	chr10	22537085	22539934	-	1223	-3.35	-1
1520402 A15Rik	ENSMUSG00000028701	chr4	115805011	115823943	-	5886	-3.351	-1
Commd 9	ENSMUSG00000027163	chr2	101726404	101741803	+	1602	-3.351	-1
Dpp4	ENSMUSG00000035000	chr2	62168130	62250288	-	6319	-3.351	-1
Atp5h	ENSMUSG00000034566	chr11	115277003	115281276	-	1465	-3.352	-1
Msra	ENSMUSG00000054733	chr14	64741466	65074740	-	1856	-3.354	-1
Atp6v1f	ENSMUSG00000004285	chr6	29417718	29420512	+	1063	-3.355	-1
Trim7	ENSMUSG00000040350	chr11	48639642	48665711	+	7157	-3.355	-1
Vegfb	ENSMUSG00000024962	chr19	7056963	7062141	-	1605	-3.357	-1
Ppif	ENSMUSG00000021868	chr14	26513640	26519954	+	3641	-3.357	-1
Taz	ENSMUSG00000009995	chrX	71527251	71535490	+	5364	-3.357	-1
Acot1	ENSMUSG00000072949	chr12	85350448	85358621	+	1564	-3.358	-1
Scrn2	ENSMUSG00000020877	chr11	96891252	96895272	+	2050	-3.36	-1
Asb6	ENSMUSG00000039483	chr2	30678617	30685853	-	5439	-3.363	-1
Grtp1	ENSMUSG00000038515	chr8	13175162	13200620	-	3091	-3.364	-1
1110049 F12Rik	ENSMUSG00000028669	chr4	135531375	135543179	-	2477	-3.366	-1
Krt17	ENSMUSG00000035557	chr11	100117531	100122343	-	1576	-3.366	-1
Rpl13a	ENSMUSG00000074129	chr7	52380928	52391787	-	3316	-3.367	-1
Aldh16a 1	ENSMUSG00000007833	chr7	52396054	52409954	-	5501	-3.368	-1
Ift27	ENSMUSG00000016637	chr15	77989895	78004538	-	1012	-3.368	-1
Gm1007 3	ENSMUSG00000060019	chr8	109096906	109097250	-	345	-3.368	-1
Imp3	ENSMUSG00000032288	chr9	56785307	56786204	+	898	-3.369	-1
Tmub1	ENSMUSG00000028958	chr5	23951276	23953672	-	1879	-3.369	-1
Cby1	ENSMUSG00000022428	chr15	79489657	79498090	+	1119	-3.369	-1
Coq6	ENSMUSG00000021235	chr12	85702607	85714746	+	3142	-3.37	-1
Alg1	ENSMUSG00000039427	chr16	5233714	5245005	+	2682	-3.371	-1
Dus1l	ENSMUSG00000025155	chr11	120650515	120657717	-	3758	-3.374	-1
Ngfrap1	ENSMUSG00000046432	chrX	132804800	132806517	+	965	-3.374	-1
Rbck1	ENSMUSG00000027466	chr2	152142070	152158389	-	4601	-3.375	-1
Sars2	ENSMUSG00000070699	chr7	29527010	29538871	+	1860	-3.376	-1
2700094 K13Rik	ENSMUSG00000076437	chr2	84509375	84510927	-	999	-3.377	-1
Man2c1	ENSMUSG00000032295	chr9	56978497	56990529	+	7052	-3.378	-1
Rpl37- ps1	ENSMUSG00000049247	chr19	16099941	16100210	+	270	-3.378	-1

Aspscr1	ENSMUSG00000025142	chr11	120534287	120570761	+	4770	-3.379	-1
Dguok	ENSMUSG00000014554	chr6	83430211	83456963	-	2191	-3.382	-1
Amdhd2	ENSMUSG00000036820	chr17	24292800	24300733	-	2728	-3.383	-1
1810013D10Rik	ENSMUSG00000061461	chr5	53658345	53674749	+	6494	-3.384	-1
Gm10250	ENSMUSG00000068706	chr15	5070653	5071135	-	483	-3.385	-1
Pih1d1	ENSMUSG00000003423	chr7	52409673	52415439	+	2024	-3.386	-1
0610009E02Rik	ENSMUSG00000086714	chr2	26301216	26314910	+	1803	-3.386	-1
Asna1	ENSMUSG00000052456	chr8	87541833	87549177	-	1250	-3.387	-1
Trmt1	ENSMUSG00000001909	chr8	87210206	87223707	+	6306	-3.388	-1
Slc9a7	ENSMUSG00000037341	chrX	19682881	19868933	-	3819	-3.389	-1
1810006K21Rik	ENSMUSG00000036372	chr19	10278504	10282314	+	1611	-3.389	-1
Arl2	ENSMUSG00000024944	chr19	6134374	6141406	-	2891	-3.389	-1
Rdbp	ENSMUSG00000024369	chr17	34987336	34993317	+	2076	-3.39	-1
Fam3a	ENSMUSG00000031399	chrX	71630058	71638611	-	2485	-3.391	-1
Uqcr10	ENSMUSG00000059534	chr11	4601976	4604345	-	584	-3.396	-1
Uxt	ENSMUSG00000001134	chrX	20518848	20539143	-	1639	-3.397	-1
Gm16380	ENSMUSG00000059830	chr9	53731889	53732368	+	459	-3.397	-1
Flad1	ENSMUSG00000042642	chr3	89204926	89215792	-	4087	-3.397	-1
Bckdhb	ENSMUSG00000032263	chr9	83818757	84017846	+	1487	-3.398	-1
Plekhj1	ENSMUSG00000035278	chr10	80258845	80261371	-	1259	-3.399	-1
Smpdl3b	ENSMUSG00000028885	chr4	132288881	132313167	-	2006	-3.4	-1
Spnb3	ENSMUSG00000067889	chr19	4711208	4752353	+	8251	-3.402	-1
Klhl17	ENSMUSG00000078484	chr4	155603153	155608966	-	3014	-3.402	-1
Rplp1	ENSMUSG00000007892	chr9	61761091	61762345	-	495	-3.403	-1
2210417K05Rik	ENSMUSG00000085347	chr10	61803004	61826115	+	1556	-3.403	-1
Ap1g2	ENSMUSG00000040701	chr14	55717415	55725430	-	4177	-3.405	-1
Adck2	ENSMUSG00000046947	chr6	39523872	39546594	+	4095	-3.406	-1
Gprc5c	ENSMUSG00000051043	chr11	114712466	114733931	+	5246	-3.407	-1
Ppp2r5b	ENSMUSG00000024777	chr19	6227765	6235872	-	2895	-3.407	-1
Ccdc62	ENSMUSG00000061882	chr5	124377427	124419904	+	4306	-3.407	-1
Ndufa1	ENSMUSG00000016427	chrX	34727583	34731158	-	419	-3.408	-1
H2-T22	ENSMUSG00000056116	chr17	36174073	36179692	-	3414	-3.408	-1
E430018J23Rik	ENSMUSG00000078580	chr7	134533187	134537143	-	3334	-3.409	-1
Cnpy2	ENSMUSG00000025381	chr10	127759515	127764243	+	1017	-3.41	-1
Cars2	ENSMUSG00000056228	chr8	11514021	11550771	-	2623	-3.414	-1

Gls2	ENSMUSG00000044005	chr10	127631513	127647060	+	3474	-3.416	-1
Tmem222	ENSMUSG00000028857	chr4	132821960	132833707	-	2003	-3.417	-1
Ap2s1	ENSMUSG00000008036	chr7	17323759	17334643	+	1333	-3.419	-1
Tsen54	ENSMUSG00000020781	chr11	115676038	115684408	+	2458	-3.42	-1
Gm15545	ENSMUSG00000087138	chr7	52242270	52249967	+	1976	-3.426	-1
Rpl32	ENSMUSG00000057841	chr6	115755523	115758765	-	517	-3.429	-1
Akt1s1	ENSMUSG00000011096	chr7	52104361	52110788	+	3198	-3.431	-1
Taf1c	ENSMUSG00000031832	chr8	122121875	122129122	-	3386	-3.435	-1
Klhl36	ENSMUSG00000031828	chr8	122386205	122400888	+	2141	-3.435	-1
Rps15	ENSMUSG00000063457	chr10	79755176	79756859	+	652	-3.437	-1
Dhdds	ENSMUSG00000012117	chr4	133524943	133556833	-	3784	-3.437	-1
Kcnma1	ENSMUSG00000063142	chr14	24117983	24622526	-	4756	-3.441	-1
Psme2b-ps	ENSMUSG00000078153	chr11	48758856	48759692	-	837	-3.442	-1
Atp5j2	ENSMUSG00000038690	chr5	145944567	145952931	-	1231	-3.442	-1
Gm17025	ENSMUSG00000091492	chr15	81245990	81249618	-	580	-3.443	-1
Ebpl	ENSMUSG00000021928	chr14	61958600	61979282	-	2077	-3.443	-1
Gm3699	ENSMUSG00000069648	chr10	43176122	43176321	-	200	-3.443	-1
Lipc	ENSMUSG00000032207	chr9	70645936	70800033	-	2102	-3.444	-1
Abcb9	ENSMUSG00000029408	chr5	124511539	124545807	-	5817	-3.445	-1
Gstk1	ENSMUSG00000029864	chr6	42195934	42200440	+	930	-3.445	-1
Slc16a10	ENSMUSG00000019838	chr10	39753341	39862062	-	6146	-3.447	-1
1500032L24Rik	ENSMUSG00000022452	chr15	82169389	82179521	+	1077	-3.448	-1
Thap7	ENSMUSG00000022760	chr16	17528075	17531229	-	1880	-3.448	-1
Srcrb4d	ENSMUSG00000029699	chr5	136436081	136450401	-	2976	-3.448	-1
Zfp213	ENSMUSG00000071256	chr17	23693736	23701193	-	2672	-3.45	-1
Tomm5	ENSMUSG00000078713	chr4	45118080	45120986	-	868	-3.45	-1
Fbxo6	ENSMUSG00000055401	chr4	147519825	147526249	-	2207	-3.451	-1
Arvcf	ENSMUSG00000000325	chr16	18348275	18407169	+	6517	-3.454	-1
Sdhb	ENSMUSG00000009863	chr4	140517118	140535108	+	2239	-3.455	-1
Selenbp1	ENSMUSG00000068874	chr3	94736978	94748680	+	2273	-3.456	-1
Tmem143	ENSMUSG00000002781	chr7	53152311	53172783	+	5636	-3.46	-1
Tecr	ENSMUSG00000031708	chr8	86095599	86118390	-	1334	-3.462	-1
Trnau1ap	ENSMUSG00000028898	chr4	131867678	131885453	-	2710	-3.463	-1
Pomgnt1	ENSMUSG00000028700	chr4	115796445	115832454	+	5463	-3.464	-1
Ubxn6	ENSMUSG00000019578	chr17	56206468	56214451	-	5685	-3.465	-1

5830454E08Rik	ENSMUSG00000052658	chr9	120486463	120487189	+	727	-3.466	-1
Tusc2	ENSMUSG00000010054	chr9	107465586	107468439	+	1661	-3.467	-1
Ptov1	ENSMUSG00000038502	chr7	52118438	52125158	-	4212	-3.47	-1
Psme2	ENSMUSG00000079197	chr14	56206278	56209950	-	1764	-3.47	-1
Ccdc159	ENSMUSG00000006241	chr9	21731915	21740316	+	1545	-3.471	-1
Plekhb1	ENSMUSG00000030701	chr7	107791406	107810928	-	3554	-3.471	-1
Rps2	ENSMUSG00000044533	chr17	24855061	24858874	+	1830	-3.471	-1
Creb3l4	ENSMUSG00000027938	chr3	90041422	90047434	-	1679	-3.472	-1
Rusc1	ENSMUSG00000041263	chr3	88887903	88897285	-	3840	-3.473	-1
Fbxo44	ENSMUSG00000029001	chr4	147526909	147534591	-	3024	-3.474	-1
Nudt1	ENSMUSG00000036639	chr5	140807814	140814091	+	1390	-3.474	-1
Gm6576	ENSMUSG00000090544	chr15	26955056	26956010	+	955	-3.475	-1
Cpm	ENSMUSG00000020183	chr10	117066556	117124408	+	5964	-3.476	-1
Egflam	ENSMUSG00000042961	chr15	7156120	7348395	-	6121	-3.476	-1
Hist2h4	ENSMUSG00000091405	chr3	96065627	96067234	-	1608	-3.477	-1
Fuk	ENSMUSG00000033703	chr8	113406363	113426359	-	3965	-3.477	-1
Cyp4f13	ENSMUSG00000024055	chr17	33061633	33084347	-	3330	-3.478	-1
Tmem191c	ENSMUSG00000055692	chr16	17276393	17283322	+	2441	-3.48	-1
Rce1	ENSMUSG00000024889	chr19	4622551	4625617	-	1446	-3.48	-1
1700112E06Rik	ENSMUSG00000063458	chr14	22838934	23875307	+	2717	-3.482	-1
Immp2l	ENSMUSG00000056899	chr12	41750677	43056575	+	1832	-3.482	-1
Galk1	ENSMUSG00000020766	chr11	115869771	115874033	-	1406	-3.483	-1
Tesk2	ENSMUSG00000033985	chr4	116393553	116478561	+	4272	-3.485	-1
1110014N23Rik	ENSMUSG00000024797	chr19	6067842	6080324	-	4748	-3.486	-1
Kazald1	ENSMUSG00000025213	chr19	45149731	45153779	+	1681	-3.487	-1
Snrpd2	ENSMUSG00000040824	chr7	19735071	19738084	+	613	-3.487	-1
Exosc4	ENSMUSG00000034259	chr15	76157827	76161107	+	1945	-3.487	-1
Acads	ENSMUSG00000029545	chr5	115560308	115569355	-	2269	-3.488	-1
Qtrt1	ENSMUSG00000002825	chr9	21216281	21224721	+	1319	-3.488	-1
G6pc3	ENSMUSG00000034793	chr11	102050934	102055395	+	2226	-3.493	-1
Gm4285	ENSMUSG00000085829	chr14	76243186	76244771	-	902	-3.494	-1
Mib2	ENSMUSG00000029060	chr4	155028786	155043307	-	4772	-3.495	-1
Zbtb48	ENSMUSG00000028952	chr4	151393883	151401780	-	3199	-3.495	-1
Psmg4	ENSMUSG00000071451	chr13	34254833	34270041	+	554	-3.497	-1
Dctpp1	ENSMUSG00000042462	chr7	134400473	134404223	-	676	-3.498	-1
Peli3	ENSMUSG00000024901	chr19	4930651	4943127	-	3786	-3.502	-1

Tbc1d17	ENSMUSG00000038520	chr7	52096271	52104449	-	3448	-3.502		-1
B3gat3	ENSMUSG00000071649	chr19	8994864	9001724	+	1601	-3.503		-1
Pmm1	ENSMUSG00000022474	chr15	81781538	81791360	-	2776	-3.508		-1
Hap1	ENSMUSG00000006930	chr11	100208641	100217455	-	3779	-3.509		-1
Ccs	ENSMUSG00000034108	chr19	4825366	4839322	-	1057	-3.51		-1
Haghl	ENSMUSG00000061046	chr17	25916788	25922618	-	2643	-3.51		-1
Lamtor2	ENSMUSG00000028062	chr3	88353741	88356996	-	1218	-3.51		-1
Ica1	ENSMUSG00000062995	chr6	8580527	8728488	-	3243	-3.513		-1
Tbl3	ENSMUSG00000040688	chr17	24834894	24844605	-	5686	-3.513		-1
Gss	ENSMUSG00000027610	chr2	155388917	155418546	-	5915	-3.517		-1
Wbscr27	ENSMUSG00000040557	chr5	135408238	135418507	+	3342	-3.517		-1
Mt2	ENSMUSG00000031762	chr8	96696518	96697467	+	556	-3.518		-1
Ring1	ENSMUSG00000024325	chr17	34157737	34161625	-	3243	-3.518		-1
Rab19	ENSMUSG00000029923	chr6	39331174	39340379	+	1575	-3.519		-1
Mettl11a	ENSMUSG00000026857	chr2	30663346	30678553	+	2798	-3.519		-1
Cd52	ENSMUSG00000000682	chr4	133638363	133650997	-	1187	-3.521		-1
Adam33	ENSMUSG00000027318	chr2	130876327	130889550	-	5394	-3.522		-1
H2-Ke6	ENSMUSG00000073422	chr17	34162978	34165005	-	1533	-3.522		-1
Rps6ka1	ENSMUSG00000003644	chr4	133403205	133443712	-	4258	-3.523		-1
Zfp94	ENSMUSG00000074282	chr7	25086723	25101685	-	3510	-3.523		-1
Grcc10	ENSMUSG00000072772	chr6	124689201	124691392	-	1988	-3.524		-1
2310004N24Rik	ENSMUSG00000020778	chr11	116060169	116076632	+	2728	-3.525		-1
Psen2	ENSMUSG00000010609	chr1	182157135	182193569	-	3832	-3.525		-1
Traf1	ENSMUSG00000026875	chr2	34797270	34817292	-	5083	-3.526		-1
Gm9843	ENSMUSG00000050299	chr16	76403454	76403957	-	504	-3.527		-1
Gm14966	ENSMUSG00000079467	chr19	6355522	6363759	-	802	-3.527		-1
Cyc1	ENSMUSG00000022551	chr15	76173953	76176364	+	1194	-3.53		-1
Gpr108	ENSMUSG00000005823	chr17	57374341	57387082	-	2109	-3.53		-1
Oit1	ENSMUSG00000021749	chr14	9181462	9211277	-	1251	-3.531		-1
Slc2a8	ENSMUSG00000026791	chr2	32828510	32837603	-	2336	-3.533		-1
Tmco6	ENSMUSG00000006850	chr18	36894722	36902045	+	1817	-3.534		-1
Ccdc107	ENSMUSG00000028461	chr4	43505772	43508793	+	1547	-3.535		-1
Romo1	ENSMUSG00000067847	chr2	155969775	155971533	+	747	-3.536		-1
3110001D03Rik	ENSMUSG00000028398	chr4	74923258	74924209	-	741	-3.537		-1
Fbxw9	ENSMUSG00000008167	chr8	87583954	87591023	+	3383	-3.537		-1
Fam195b	ENSMUSG00000061111	chr11	120404202	120411041	-	1282	-3.539		-1

1190005I06Rik	ENSMUSG00000043687	chr8	123132502	123158392	-	970	-3.539		-1
Gfer	ENSMUSG00000040888	chr17	24830136	24833101	-	1775	-3.541		-1
2610204G22Rik	ENSMUSG00000054514	chr4	155135287	155143373	+	1549	-3.541		-1
Tnxb	ENSMUSG00000033327	chr17	34807480	34856760	+	12533	-3.541		-1
Oaz1	ENSMUSG00000035242	chr10	80289401	80292035	+	1071	-3.542		-1
Slc25a1	ENSMUSG00000003528	chr16	17925304	17928312	-	2176	-3.543		-1
Hpn	ENSMUSG00000001249	chr7	31883744	31900309	-	3323	-3.543		-1
Aamp	ENSMUSG00000006299	chr1	74326421	74331254	-	1752	-3.544		-1
Surf1	ENSMUSG00000015790	chr2	26768903	26772050	-	1724	-3.545		-1
Mrpl2	ENSMUSG00000002767	chr17	46783178	46787088	+	1170	-3.546		-1
Taf6l	ENSMUSG00000003680	chr19	8848844	8860907	-	5147	-3.547		-1
Acy1	ENSMUSG00000023262	chr9	106335327	106340567	-	1431	-3.548		-1
Ift20	ENSMUSG00000001105	chr11	78349863	78355239	+	1525	-3.55		-1
Phf1	ENSMUSG00000024193	chr17	27070072	27074835	+	2429	-3.551		-1
Dact3	ENSMUSG00000078794	chr7	17460666	17472650	+	2828	-3.553		-1
Egln2	ENSMUSG00000058709	chr7	27943677	27951821	-	2800	-3.553		-1
Gm10120	ENSMUSG00000062678	chr10	127448832	127449188	+	357	-3.554		-1
Cnpy3	ENSMUSG00000023973	chr17	46872654	46889163	-	2659	-3.556		-1
Ovca2	ENSMUSG00000038268	chr11	74989444	74992337	-	2670	-3.556		-1
Abhd11	ENSMUSG00000040532	chr5	135485022	135488045	+	2172	-3.558		-1
AI413582	ENSMUSG00000062753	chr17	27700520	27702694	-	1398	-3.558		-1
Polr1c	ENSMUSG00000067148	chr17	46380869	46385003	-	2278	-3.562		-1
Gm20522	ENSMUSG00000092241	chr17	35259683	35263385	-	784	-3.562		-1
Ost4	ENSMUSG00000038803	chr5	31208258	31210161	-	1272	-3.562		-1
Dhcr7	ENSMUSG00000058454	chr7	151009050	151034315	+	3784	-3.563		-1
Gm16972	ENSMUSG00000086983	chr4	43058906	43066125	+	592	-3.567		-1
Dcps	ENSMUSG00000032040	chr9	34931993	34983646	-	1451	-3.567		-1
Tuba8	ENSMUSG00000030137	chr6	121160752	121176872	+	2225	-3.57		-1
Naa10	ENSMUSG00000031388	chrX	71162212	71167283	-	4132	-3.57		-1
Gm5292	ENSMUSG00000059565	chr5	44335141	44335722	-	582	-3.572		-1
Slc39a7	ENSMUSG00000024327	chr17	34165212	34168635	-	2594	-3.572		-1
Ppie	ENSMUSG00000028651	chr4	122804358	122817194	-	1719	-3.572		-1
Rpl36-ps3	ENSMUSG00000066629	chr12	12918792	12919175	+	384	-3.573		-1
Slc25a38	ENSMUSG00000032519	chr9	120019492	120033622	+	2747	-3.574		-1
Zbtb8a	ENSMUSG00000028807	chr4	129030872	129055360	-	2321	-3.579		-1
Mrpl17	ENSMUSG00000030879	chr7	112955172	112959613	-	4153	-3.58		-1
Smarcd2	ENSMUSG00000078619	chr11	106124493	106134286	-	3589	-3.582		-1

Ncdn	ENSMUSG00000028833	chr4	126420994	126430682	-	3710	-3.583	-1
Sars	ENSMUSG00000068739	chr3	108227783	108248127	-	3653	-3.584	-1
Fbxl15	ENSMUSG00000025226	chr19	46402674	46404936	+	1348	-3.596	-1
Dhodh	ENSMUSG00000031730	chr8	112115243	112132573	-	6326	-3.602	-1
Trappc2l	ENSMUSG00000015013	chr8	125135526	125139488	+	607	-3.604	-1
Ppt2	ENSMUSG00000015474	chr17	34753607	34765455	-	2614	-3.605	-1
Itm2c	ENSMUSG00000026223	chr1	87790856	87805250	+	2491	-3.606	-1
Manf	ENSMUSG00000032575	chr9	106740643	106794310	-	3524	-3.607	-1
Tm2d2	ENSMUSG00000031556	chr8	26127683	26133731	+	1280	-3.608	-1
Slc16a7	ENSMUSG00000020102	chr10	124663520	124766019	-	3890	-3.612	-1
Loh12cr1	ENSMUSG00000042992	chr6	134589645	134661202	+	1822	-3.614	-1
Acss1	ENSMUSG00000027452	chr2	150443841	150494236	-	4128	-3.615	-1
Comt	ENSMUSG00000000326	chr16	18406979	18426945	-	2532	-3.615	-1
Eif3g	ENSMUSG00000070319	chr9	20698796	20703044	-	1075	-3.615	-1
Sh3glb2	ENSMUSG00000026860	chr2	30200329	30214857	-	3990	-3.617	-1
Mrps6	ENSMUSG00000039680	chr16	92058515	92112472	+	1590	-3.619	-1
Ntf5	ENSMUSG00000074121	chr7	52669065	52672548	+	1962	-3.62	-1
Sphk2	ENSMUSG00000057342	chr7	52964837	52973372	-	4159	-3.627	-1
2010012P19Rik	ENSMUSG00000085890	chr11	69495640	69504643	+	1109	-3.627	-1
Gm10076	ENSMUSG00000060143	chr14	106081045	106081421	+	377	-3.627	-1
Efs	ENSMUSG00000022203	chr14	55535380	55545625	-	3571	-3.634	-1
Rps21	ENSMUSG00000039001	chr2	179992082	179993150	+	810	-3.634	-1
Gpr137	ENSMUSG00000024958	chr19	7012559	7016940	-	2768	-3.635	-1
Ndufa11	ENSMUSG00000002379	chr17	56857185	56863671	+	2661	-3.638	-1
Rplp2	ENSMUSG00000025508	chr7	148633269	148637484	+	2072	-3.638	-1
Acot8	ENSMUSG00000017307	chr2	164618268	164630382	-	1994	-3.643	-1
Gmpr	ENSMUSG00000000253	chr13	45602813	45641751	+	1871	-3.643	-1
1700022N22Rik	ENSMUSG00000090452	chr17	26122760	26125414	+	709	-3.644	-1
Gm10736	ENSMUSG00000074660	chr13	112383634	112383833	-	200	-3.644	-1
Entpd2	ENSMUSG00000015085	chr2	25251394	25256843	+	2130	-3.646	-1
Mvd	ENSMUSG00000006517	chr8	124957496	124967322	-	1761	-3.646	-1
Adprhl2	ENSMUSG00000042558	chr4	125993291	125998947	-	2475	-3.647	-1
Naga	ENSMUSG00000022453	chr15	82159962	82169256	-	1913	-3.648	-1
2700012I20Rik	ENSMUSG00000090823	chr9	56923023	56923820	-	684	-3.649	-1
Lgals2	ENSMUSG00000043501	chr15	78681292	78685959	-	590	-3.649	-1
Nkg7	ENSMUSG00000004612	chr7	50692443	50693616	+	878	-3.653	-1
Rpl32-ps	ENSMUSG00000068969	chr3	69520908	69521315	-	408	-3.653	-1

Atf6b	ENSMUSG00000015461	chr17	34784091	34792019	+	2757	-3.654	-1
B930041F14Rik	ENSMUSG00000074738	chr4	155068451	155070590	+	2140	-3.656	-1
Lsm4	ENSMUSG00000031848	chr8	73197147	73202651	+	1038	-3.658	-1
Pard6a	ENSMUSG00000005699	chr8	108225054	108227393	+	1428	-3.659	-1
Eif3i	ENSMUSG00000028798	chr4	129269204	129277892	-	3702	-3.663	-1
Zdhhc1	ENSMUSG00000039199	chr8	107996325	108008330	-	1873	-3.664	-1
Slc39a11	ENSMUSG00000041654	chr11	113106167	113511393	-	3359	-3.665	-1
Mthfsd	ENSMUSG00000031816	chr8	123618912	123632292	-	4894	-3.665	-1
Coasy	ENSMUSG00000001755	chr11	100943879	100947933	+	2867	-3.667	-1
Apeh	ENSMUSG00000032590	chr9	107987748	107996811	-	2388	-3.667	-1
Gm16517	ENSMUSG00000020308	chr10	79132155	79138871	+	1101	-3.667	-1
Uba52	ENSMUSG00000090137	chr8	73032162	73034700	-	1498	-3.669	-1
Hcfc1r1	ENSMUSG00000023904	chr17	23810575	23812491	+	1243	-3.671	-1
Ndufv3	ENSMUSG00000024038	chr17	31657060	31668266	+	2016	-3.673	-1
Ttc7	ENSMUSG00000036918	chr17	87682226	87781110	+	6316	-3.673	-1
Mrps26	ENSMUSG00000037740	chr2	130389478	130394431	+	4358	-3.677	-1
Ydjc	ENSMUSG00000041774	chr16	17144714	17160794	+	5556	-3.678	-1
Sema6c	ENSMUSG00000038777	chr3	94964379	94977946	+	5935	-3.681	-1
Lage3	ENSMUSG00000015289	chrX	71596615	71598957	-	2021	-3.682	-1
Cbx7	ENSMUSG00000053411	chr15	79746237	79801549	-	6487	-3.682	-1
Lmf1	ENSMUSG00000002279	chr17	25716119	25799771	+	14440	-3.683	-1
Gm7535	ENSMUSG00000090957	chr17	18048003	18048911	-	909	-3.684	-1
Zfp667	ENSMUSG00000054893	chr7	6238181	6259485	+	4755	-3.685	-1
Aldh2	ENSMUSG00000029455	chr5	122016036	122043833	-	4704	-3.687	-1
Cd79b	ENSMUSG00000040592	chr11	106172655	106176076	-	1431	-3.688	-1
Tmem203	ENSMUSG00000078201	chr2	25110959	25111872	+	914	-3.69	-1
Nipal2	ENSMUSG00000038879	chr15	34502554	34608461	-	3825	-3.694	-1
0610007P22Rik	ENSMUSG00000015126	chr17	25377115	25379743	+	1380	-3.695	-1
Pgap2	ENSMUSG00000030990	chr7	109358722	109387081	+	4531	-3.698	-1
C030006K11Rik	ENSMUSG00000079002	chr15	76551896	76554275	-	1758	-3.7	-1
BC003266	ENSMUSG00000042380	chr4	126921028	126925053	+	1022	-3.7	-1
1190007I07Rik	ENSMUSG00000063320	chr10	82082596	82085941	-	818	-3.702	-1
Kifc2	ENSMUSG00000004187	chr15	76490996	76498625	+	3217	-3.707	-1
Zap70	ENSMUSG00000026117	chr1	36818695	36839663	+	2330	-3.709	-1
Cbr3	ENSMUSG00000022947	chr16	93683460	93691235	+	1170	-3.71	-1
Lsmd1	ENSMUSG00000059278	chr11	69208989	69210182	+	890	-3.711	-1
Dact2	ENSMUSG00000048826	chr17	14332238	14340838	-	2808	-3.714	-1

Timm13	ENSMUSG00000020219	chr10	80362195	80363714	-	1225	-3.716	-1
Plekha6	ENSMUSG00000041757	chr1	135142674	135200012	+	7471	-3.717	-1
Mrpl12	ENSMUSG00000039640	chr11	120345927	120350379	+	1532	-3.718	-1
Fam46c	ENSMUSG00000044468	chr3	100255551	100293247	-	6097	-3.719	-1
Cbr1	ENSMUSG00000051483	chr16	93608082	93610750	+	1237	-3.719	-1
4930572 J05Rik	ENSMUSG00000056665	chr15	74551664	74554803	+	1488	-3.719	-1
Fam86	ENSMUSG00000022544	chr16	5244245	5256076	-	2649	-3.719	-1
Slc50a1	ENSMUSG00000027953	chr3	89072168	89074492	-	1466	-3.719	-1
Psmb5	ENSMUSG00000022193	chr14	55232644	55236851	-	922	-3.72	-1
Mrps16	ENSMUSG00000049960	chr14	21210453	21212777	-	566	-3.72	-1
Pcbd2	ENSMUSG00000021496	chr13	55828729	55878191	+	546	-3.722	-1
Uqcrh	ENSMUSG00000063882	chr4	115739570	115747676	-	1237	-3.722	-1
Proc	ENSMUSG00000024386	chr18	32282783	32299224	-	1643	-3.723	-1
Galnt10	ENSMUSG00000020520	chr11	57458944	57601016	+	5965	-3.726	-1
Zbtb42	ENSMUSG00000037638	chr12	113917039	113920958	+	3490	-3.73	-1
Abhd14 a	ENSMUSG00000042210	chr9	106342382	106350009	-	1493	-3.731	-1
Tead2	ENSMUSG00000030796	chr7	52471123	52489005	+	4668	-3.732	-1
Park7	ENSMUSG00000028964	chr4	150271242	150288546	-	2479	-3.733	-1
Usp20	ENSMUSG00000026854	chr2	30837799	30879106	+	7431	-3.734	-1
Abhd8	ENSMUSG00000007950	chr8	73980599	73987556	-	1985	-3.735	-1
Tor2a	ENSMUSG00000009563	chr2	32612754	32617764	+	3050	-3.736	-1
Lims2	ENSMUSG00000024395	chr18	32091161	32118273	+	1688	-3.738	-1
Chchd8	ENSMUSG00000044881	chr7	107685614	107688883	+	1425	-3.74	-1
A4galt	ENSMUSG00000047878	chr15	83057156	83082204	-	2171	-3.74	-1
Deaf1	ENSMUSG00000058886	chr7	148483075	148524660	-	2873	-3.745	-1
Pnkd	ENSMUSG00000026179	chr1	74331504	74400268	+	4362	-3.746	-1
0910001 L09Rik	ENSMUSG00000050552	chr5	138696836	138700626	+	1176	-3.747	-1
Rnaseh 2c	ENSMUSG00000024925	chr19	5601873	5603439	+	1455	-3.747	-1
Rpl13a- ps1	ENSMUSG00000062083	chr19	50104614	50105225	-	612	-3.748	-1
Gm1039 9	ENSMUSG00000072688	chr5	117768754	117769251	-	498	-3.748	-1
Thop1	ENSMUSG00000004929	chr10	80532780	80545304	+	5059	-3.748	-1
Chmp1a	ENSMUSG00000000743	chr8	125728164	125736663	-	3260	-3.748	-1
Cdk10	ENSMUSG00000033862	chr8	125748741	125756150	+	1643	-3.75	-1
Car11	ENSMUSG00000003273	chr7	52955213	52960052	+	1926	-3.75	-1
Bola2	ENSMUSG00000047721	chr7	133838915	133840572	+	1658	-3.75	-1
Ndufb8	ENSMUSG00000025204	chr19	44623062	44629930	-	3146	-3.753	-1
Thap3	ENSMUSG00000039759	chr4	151356748	151363106	-	1334	-3.753	-1
Hsd17b 10	ENSMUSG00000025260	chrX	148436388	148438985	+	1298	-3.754	-1

Creg2	ENSMUSG00000050967	chr1	39675251	39708027	-	5799	-3.754	-1
9130230N09Rik	ENSMUSG00000092626	chr11	22758931	22761264	+	663	-3.755	-1
Atp6v1g2	ENSMUSG00000024403	chr17	35370605	35375712	+	2510	-3.758	-1
Megf6	ENSMUSG00000057751	chr4	153544839	153649822	+	7746	-3.761	-1
Ndufs7	ENSMUSG00000020153	chr10	79711866	79719539	+	1718	-3.761	-1
Trpt1	ENSMUSG00000047656	chr19	7070621	7073534	+	1142	-3.765	-1
Osgep	ENSMUSG00000006289	chr14	51526153	51544568	-	4933	-3.765	-1
Gm14235	ENSMUSG00000085267	chr2	167973517	167979033	-	366	-3.766	-1
Slc25a39	ENSMUSG00000018677	chr11	102264299	102269260	-	2756	-3.767	-1
Ndufs8	ENSMUSG00000059734	chr19	3908870	3912717	-	963	-3.77	-1
Csad	ENSMUSG00000023044	chr15	102007429	102019474	-	2265	-3.772	-1
Smad9	ENSMUSG00000027796	chr3	54559504	54605179	+	5368	-3.773	-1
Zfand2b	ENSMUSG00000026197	chr1	75165220	75168203	+	1772	-3.773	-1
Cgn	ENSMUSG00000068876	chr3	94464507	94590414	-	6534	-3.774	-1
Mrpl11	ENSMUSG00000024902	chr19	4962315	4964943	+	821	-3.775	-1
Znhit2-ps	ENSMUSG00000075227	chr19	6061192	6062472	+	1281	-3.775	-1
Commd5	ENSMUSG00000055041	chr15	76730340	76731735	+	1060	-3.775	-1
Sirt3	ENSMUSG00000025486	chr7	148049569	148068208	-	1570	-3.778	-1
E230013L22Rik	ENSMUSG00000090605	chr8	11477929	11480241	+	1856	-3.778	-1
Aurkaip1	ENSMUSG00000065990	chr4	155205381	155207239	+	1766	-3.778	-1
Krt23	ENSMUSG00000006777	chr11	99339288	99354451	-	1565	-3.784	-1
Arf5	ENSMUSG00000020440	chr6	28373560	28376602	+	1116	-3.784	-1
Ndufa3	ENSMUSG00000035674	chr7	3568975	3571765	+	858	-3.784	-1
Orai3	ENSMUSG00000043964	chr7	134913329	134918664	+	2141	-3.787	-1
4930404N11Rik	ENSMUSG00000020234	chr10	80825812	80828561	-	1751	-3.787	-1
A430018G15Rik	ENSMUSG00000085315	chr2	52280532	52290405	+	1713	-3.79	-1
Slc25a33	ENSMUSG00000028982	chr4	149118145	149148386	-	1887	-3.79	-1
Arrdc1	ENSMUSG00000026972	chr2	24780872	24790772	-	2510	-3.791	-1
Ubxn11	ENSMUSG00000012126	chr4	133658485	133683508	+	4742	-3.792	-1
Bad	ENSMUSG00000024959	chr19	7016351	7026388	+	2007	-3.792	-1
Tsen34	ENSMUSG00000035585	chr7	3644977	3652625	+	2416	-3.793	-1
Ang	ENSMUSG00000072115	chr14	51710752	51721682	+	1273	-3.794	-1
Gm5299	ENSMUSG00000063324	chr5	30547454	30547729	-	276	-3.795	-1

1300018J18Rik	ENSMUSG00000035757	chr15	88919514	88930770	+	3744	-3.796		-1
0610031J06Rik	ENSMUSG00000001418	chr3	88128945	88135235	+	5862	-3.798		-1
Ccdc85c	ENSMUSG00000084883	chr12	109444555	109513627	-	1547	-3.798		-1
Ndufaf3	ENSMUSG00000070283	chr9	108468197	108469673	-	1066	-3.8		-1
4930412F15Rik	ENSMUSG00000043633	chr4	43672494	43682017	-	4413	-3.8		-1
Snx17	ENSMUSG00000029146	chr5	31495600	31501273	+	2791	-3.801		-1
BC048355	ENSMUSG00000040658	chr17	46633738	46636567	+	1106	-3.803		-1
Pink1	ENSMUSG00000028756	chr4	137869324	137882222	-	2673	-3.804		-1
Rpl41	ENSMUSG00000093674	chr10	127985170	127986361	-	1168	-3.807		-1
Gm5239	ENSMUSG00000062742	chr18	35696237	35696605	+	369	-3.81		-1
Trim29	ENSMUSG00000032013	chr9	43118846	43144208	+	2830	-3.81		-1
Coq9	ENSMUSG00000031782	chr8	97362221	97378795	+	3055	-3.811		-1
Wbp1	ENSMUSG00000030035	chr6	83069038	83071553	-	2079	-3.813		-1
Ndufb10	ENSMUSG00000040048	chr17	24859005	24861423	-	1217	-3.813		-1
Wbscr17	ENSMUSG00000034040	chr5	131347952	131783948	-	8989	-3.814		-1
2410015M20Rik	ENSMUSG00000049760	chr17	56746875	56749194	-	755	-3.816		-1
Tmem107	ENSMUSG00000020895	chr11	68884308	68886795	+	1369	-3.816		-1
Scly	ENSMUSG00000026307	chr1	93194915	93217652	+	4549	-3.817		-1
Ctns	ENSMUSG00000005949	chr11	72997098	73012544	-	3391	-3.819		-1
Tox3	ENSMUSG00000043668	chr8	92770939	92872242	-	3356	-3.821		-1
Fkbp2	ENSMUSG00000056629	chr19	7052231	7054930	-	642	-3.821		-1
Dus3l	ENSMUSG00000007603	chr17	56904174	56909515	+	2303	-3.823		-1
Pecr	ENSMUSG00000026189	chr1	72305741	72330888	-	1516	-3.826		-1
Sod1	ENSMUSG00000022982	chr16	90220987	90226567	+	646	-3.826		-1
Prom1	ENSMUSG00000029086	chr5	44384864	44493271	-	4517	-3.827		-1
Xk	ENSMUSG00000015342	chrX	8849882	8890376	+	5076	-3.828		-1
Tssc4	ENSMUSG00000045752	chr7	150255154	150256998	+	1760	-3.83		-1
Ppapdc3	ENSMUSG00000051373	chr2	31951048	31966340	+	2078	-3.83		-1
Tysnd1	ENSMUSG00000020087	chr10	61158262	61165521	+	2286	-3.831		-1
Gpank1	ENSMUSG00000092417	chr17	35258400	35261759	+	1879	-3.833		-1
Zfp593	ENSMUSG00000028840	chr4	133799204	133801507	-	2088	-3.835		-1
Fxyd1	ENSMUSG00000036570	chr7	31836700	31840675	-	709	-3.836		-1
Muc20	ENSMUSG00000035638	chr16	32777505	32797521	-	4027	-3.837		-1
Ascc1	ENSMUSG00000044475	chr10	59465587	59562734	+	1539	-3.838		-1
P2rx4	ENSMUSG00000029470	chr5	123157593	123179747	+	3309	-3.839		-1
Gng8	ENSMUSG00000063594	chr7	17477135	17480784	+	920	-3.85		-1
Creg1	ENSMUSG00000040713	chr1	167693877	167705439	+	2362	-3.852		-1

Gm9846	ENSMUSG00000050621	chr9	114891515	114891858	-	344	-3.854	-1
Slc22a17	ENSMUSG00000022199	chr14	55525564	55531969	-	2810	-3.86	-1
Coq4	ENSMUSG00000026798	chr2	29643013	29653455	+	3399	-3.861	-1
Fam46b	ENSMUSG00000046694	chr4	133036047	133043853	+	2287	-3.861	-1
Rpl36	ENSMUSG00000057863	chr17	56752818	56753666	+	404	-3.863	-1
Ndufa13	ENSMUSG00000036199	chr8	72418087	72426457	-	1243	-3.864	-1
Engase	ENSMUSG00000033857	chr11	118338143	118350523	+	5184	-3.864	-1
Wdr31	ENSMUSG00000028391	chr4	62109687	62131930	-	4738	-3.864	-1
Dohh	ENSMUSG00000078440	chr10	80847173	80854305	+	3202	-3.865	-1
Ict1	ENSMUSG00000018858	chr11	115265066	115273988	+	3332	-3.865	-1
Fut2	ENSMUSG00000055978	chr7	52903965	52921764	-	2951	-3.868	-1
Slain1	ENSMUSG00000055717	chr14	104049445	104104124	+	3284	-3.868	-1
Galntl4	ENSMUSG00000038296	chr7	118615175	118923491	-	2575	-3.872	-1
Amigo3	ENSMUSG00000032593	chr9	107955490	107958032	+	2543	-3.872	-1
Klhdc9	ENSMUSG00000045259	chr1	173288577	173290929	-	1558	-3.872	-1
Miip	ENSMUSG00000029022	chr4	147234887	147242925	-	3354	-3.872	-1
Dusp23	ENSMUSG00000026544	chr1	174560900	174563105	-	1417	-3.873	-1
Tpm2	ENSMUSG00000028464	chr4	43527583	43536637	-	2909	-3.873	-1
Tmem102	ENSMUSG00000089876	chr11	69617105	69619126	-	1933	-3.874	-1
Rps10	ENSMUSG00000052146	chr17	27767367	27773574	-	1937	-3.877	-1
Mrpl28	ENSMUSG00000024181	chr17	26260465	26263558	+	1274	-3.877	-1
Car2	ENSMUSG00000027562	chr3	14886426	14900769	+	1789	-3.877	-1
Psmb6	ENSMUSG00000018286	chr11	70338869	70341360	+	1872	-3.878	-1
Acbd4	ENSMUSG00000056938	chr11	102962996	102973514	+	2937	-3.882	-1
Bet1l	ENSMUSG00000025484	chr7	148039283	148042282	-	1552	-3.886	-1
D17Wsu104e	ENSMUSG00000019579	chr17	56315167	56323343	-	2222	-3.886	-1
Slc45a3	ENSMUSG00000026435	chr1	133867186	133879540	+	3339	-3.888	-1
Gmds	ENSMUSG00000038372	chr13	31911448	32430483	-	1587	-3.889	-1
Ptges2	ENSMUSG00000026820	chr2	32251416	32258259	+	2025	-3.891	-1
Hdac10	ENSMUSG00000062906	chr15	88953737	88959130	-	3379	-3.893	-1
Rasal1	ENSMUSG00000029602	chr5	121098821	121129606	+	3539	-3.895	-1
BC051019	ENSMUSG00000031022	chr7	116855695	116867364	-	2070	-3.898	-1
Stap2	ENSMUSG00000038781	chr17	56136504	56144993	-	1491	-3.9	-1
Gga2	ENSMUSG00000030872	chr7	129130236	129164736	-	9769	-3.9	-1
Mrpl55	ENSMUSG00000036860	chr11	59015988	59019636	+	1027	-3.9	-1
AW011738	ENSMUSG00000078349	chr4	155577413	155579260	+	1848	-3.901	-1
Yif1a	ENSMUSG00000024875	chr19	5088553	5092856	+	1455	-3.905	-1
Ankrd13d	ENSMUSG00000005986	chr19	4270180	4283137	-	2460	-3.906	-1
Vps28	ENSMUSG00000062381	chr15	76452518	76456457	-	983	-3.909	-1

Vkorc1	ENSMUSG00000030804	chr7	135029741	135039131	-	2403	-3.909		-1
Pde8b	ENSMUSG00000021684	chr13	95794409	96020291	-	5879	-3.912		-1
1810027 O10Rik	ENSMUSG00000070394	chr11	69652016	69653120	+	1105	-3.915		-1
2310044 H10Rik	ENSMUSG00000008140	chr7	51745307	51751899	-	2917	-3.916		-1
Carkd	ENSMUSG00000031505	chr8	11497602	11513284	+	1322	-3.918		-1
Psmb10	ENSMUSG00000031897	chr8	108459628	108462292	-	1270	-3.918		-1
1110021 J02Rik	ENSMUSG00000024018	chr17	29826323	29853957	-	4896	-3.918		-1
Ptpn18	ENSMUSG00000026126	chr1	34516591	34532499	+	1580	-3.919		-1
Lancl1	ENSMUSG00000026000	chr1	67047091	67085446	-	4461	-3.924		-1
Mcat	ENSMUSG00000048755	chr15	83377227	83386141	-	1893	-3.926		-1
Dalrd3	ENSMUSG00000019039	chr9	108472223	108475102	+	1737	-3.926		-1
Liph	ENSMUSG00000044626	chr16	21953899	21995615	-	3793	-3.927		-1
1600016 N20Rik	ENSMUSG00000025500	chr7	148395942	148399981	-	2079	-3.927		-1
Gpatch3	ENSMUSG00000028850	chr4	133130660	133140158	+	2086	-3.928		-1
Sdhaf1	ENSMUSG00000074211	chr7	31106430	31107394	-	965	-3.929		-1
Nucb1	ENSMUSG00000030824	chr7	52748244	52765776	-	5796	-3.929		-1
Yipf1	ENSMUSG00000057375	chr4	106986968	107032428	+	6783	-3.931		-1
Wdr24	ENSMUSG00000025737	chr17	25960572	25965675	+	3432	-3.937		-1
Rhov	ENSMUSG00000034226	chr2	119094937	119097008	-	1703	-3.937		-1
Cst3	ENSMUSG00000027447	chr2	148697458	148701428	-	1266	-3.938		-1
Urod	ENSMUSG00000028684	chr4	116662570	116667018	-	2591	-3.939		-1
2810453 I06Rik	ENSMUSG00000083012	chr5	144309576	144325399	+	2732	-3.939		-1
Rtn4rl1	ENSMUSG00000045287	chr11	75007285	75081271	+	3525	-3.945		-1
Dom3z	ENSMUSG00000040482	chr17	34973964	34976178	+	1961	-3.946		-1
Pddc1	ENSMUSG00000051007	chr7	148594034	148600035	-	2871	-3.946		-1
Osr2	ENSMUSG00000022330	chr15	35225853	35233059	+	1723	-3.947		-1
Cuta	ENSMUSG00000024194	chr17	27074918	27076423	-	812	-3.949		-1
Nr2f6	ENSMUSG00000002393	chr8	73898022	73905859	-	2983	-3.952		-1
Dhrs13	ENSMUSG00000020834	chr11	77845782	77851368	+	1967	-3.957		-1
Il17d	ENSMUSG00000050222	chr14	58143666	58162002	+	1235	-3.957		-1
Slc7a5	ENSMUSG00000040010	chr8	124405052	124431594	-	3515	-3.957		-1
Six5	ENSMUSG00000040841	chr7	19679943	19683898	+	3007	-3.962		-1
Ifi35	ENSMUSG00000010358	chr11	101309721	101320012	+	1727	-3.962		-1
Rprm	ENSMUSG00000075334	chr2	53936504	53937963	-	1460	-3.963		-1
Zfp444	ENSMUSG00000044876	chr7	6123828	6142951	+	3421	-3.964		-1
Snrpn	ENSMUSG00000000948	chr7	67127388	67285105	-	2197	-3.965		-1
Qpctl	ENSMUSG00000030407	chr7	19725566	19734545	-	2100	-3.967		-1
Cables1	ENSMUSG00000040957	chr18	11997729	12104136	+	3397	-3.968		-1

Rplp2-ps1	ENSMUSG00000091018	chr12	76731924	76732365	-	442	-3.968	-1
Mrps18a	ENSMUSG00000023967	chr17	46247935	46265859	+	3690	-3.97	-1
Nfkbil1	ENSMUSG00000042419	chr17	35357120	35372737	-	1746	-3.971	-1
Bcam	ENSMUSG00000002980	chr7	20341480	20356365	-	3199	-3.972	-1
Slc25a28	ENSMUSG00000040414	chr19	43738312	43749371	-	1514	-3.974	-1
Fgfr4	ENSMUSG00000005320	chr13	55254001	55270120	+	3554	-3.976	-1
Ebp	ENSMUSG00000031168	chrX	7762455	7770638	-	2310	-3.979	-1
Rassf7	ENSMUSG00000038618	chr7	148401759	148404557	+	1803	-3.979	-1
2210011C24Rik	ENSMUSG00000074217	chr8	86534129	86535341	-	627	-3.979	-1
Ankrd24	ENSMUSG00000054708	chr10	81091285	81110355	+	8142	-3.98	-1
Mylk	ENSMUSG00000022836	chr16	34745296	35002520	+	10630	-3.981	-1
Txnrd2	ENSMUSG00000075704	chr16	18426510	18479166	+	3339	-3.986	-1
Tmem208	ENSMUSG00000014856	chr8	107850262	107859119	+	1503	-3.99	-1
Ndufb9	ENSMUSG00000022354	chr15	58765365	58771044	+	651	-3.993	-1
Upk1a	ENSMUSG00000006313	chr7	31388111	31397753	-	1308	-3.996	-1
Spsb2	ENSMUSG00000038451	chr6	124758679	124760637	+	1658	-3.998	-1
Gpr30	ENSMUSG00000053647	chr5	139899105	139903754	+	2537	-3.999	-1
Tmem9	ENSMUSG00000026411	chr1	137904796	137931607	+	1945	-4	-1
Gnptg	ENSMUSG00000035521	chr17	25371262	25377061	-	1333	-4	-1
A930005H10Rik	ENSMUSG00000054426	chr3	115590740	115591048	-	309	-4.001	-1
Rab3a	ENSMUSG00000031840	chr8	73278578	73282576	+	2226	-4.003	-1
Mrpl54	ENSMUSG00000034932	chr10	80727458	80729679	-	825	-4.007	-1
Ndufb11	ENSMUSG00000031059	chrX	20192452	20194745	-	908	-4.008	-1
Phpt1	ENSMUSG00000036504	chr2	25428950	25430744	-	1074	-4.008	-1
H2afv	ENSMUSG00000041126	chr11	6327229	6344446	-	1639	-4.01	-1
2810468N07Rik	ENSMUSG00000091475	chr17	25707861	25711979	+	1235	-4.01	-1
Cd3e	ENSMUSG00000032093	chr9	44806826	44817673	-	1436	-4.011	-1
Noxo1	ENSMUSG00000019320	chr17	24833179	24837474	+	3013	-4.013	-1
Irf3	ENSMUSG00000003184	chr7	52253018	52258218	+	3187	-4.014	-1
Gm11423	ENSMUSG00000085175	chr11	82593103	82594544	-	885	-4.015	-1
Fgf1	ENSMUSG00000036585	chr18	38998327	39089058	-	4648	-4.016	-1
Parp10	ENSMUSG00000063268	chr15	76061604	76073871	-	4275	-4.016	-1
Zfp787	ENSMUSG00000046792	chr7	6083091	6107573	-	1899	-4.017	-1
Slc25a42	ENSMUSG00000002346	chr8	72708239	72736204	-	5999	-4.017	-1
Grhpr	ENSMUSG00000035637	chr4	44994267	45003606	+	1603	-4.023	-1
Arfip2	ENSMUSG00000030881	chr7	112782717	112788930	-	4902	-4.024	-1

2810432D09Rik	ENSMUSG00000036114	chr4	41659065	41660566	-	856	-4.024	-1
Mccc2	ENSMUSG00000021646	chr13	100718488	100785594	-	2203	-4.026	-1
Tm2d3	ENSMUSG00000078681	chr7	72836055	72846799	+	1489	-4.026	-1
AI646023	ENSMUSG00000046807	chr10	75012870	75023075	-	4601	-4.027	-1
2810428I15Rik	ENSMUSG00000058833	chr8	73028191	73030651	-	1822	-4.028	-1
Eif2b4	ENSMUSG00000029145	chr5	31489931	31495803	-	2594	-4.033	-1
Stxbp2	ENSMUSG00000004626	chr8	3630955	3643644	+	4159	-4.034	-1
C330013E15Rik	ENSMUSG00000090611	chr15	100444571	100445541	-	878	-4.035	-1
A830052D11Rik	ENSMUSG00000085670	chr18	32518711	32537938	-	1130	-4.036	-1
Gm6277	ENSMUSG00000087420	chr18	11979185	11997846	-	1745	-4.036	-1
Pmm2	ENSMUSG00000022711	chr16	8637767	8657624	+	4519	-4.037	-1
Slc35c2	ENSMUSG00000017664	chr2	165102054	165113369	-	3914	-4.037	-1
Metrn	ENSMUSG00000002274	chr17	25931516	25934081	-	1434	-4.04	-1
Srm	ENSMUSG00000006442	chr4	147965612	147969102	+	1599	-4.041	-1
Sdr42e1	ENSMUSG00000034308	chr8	120185299	120197589	-	2957	-4.042	-1
Ltb	ENSMUSG00000024399	chr17	35331384	35333265	+	1516	-4.043	-1
Rps14	ENSMUSG00000024608	chr18	60934164	60938200	+	1384	-4.044	-1
Sdsl	ENSMUSG00000029596	chr5	120908195	120922819	-	1497	-4.047	-1
Fam55c	ENSMUSG00000075033	chr16	55840066	55895392	-	6430	-4.047	-1
Mrp63	ENSMUSG00000021967	chr14	58445076	58447582	+	2321	-4.05	-1
Tmem221	ENSMUSG00000043664	chr8	74078202	74082770	-	1539	-4.06	-1
Aldh4a1	ENSMUSG00000028737	chr4	139178781	139205605	+	4907	-4.061	-1
Rangrf	ENSMUSG00000032892	chr11	68785986	68788687	-	1269	-4.062	-1
Acsf3	ENSMUSG00000015016	chr8	125299405	125341780	+	2206	-4.063	-1
Snrnp25	ENSMUSG00000040767	chr11	32105415	32108984	+	1041	-4.063	-1
Ndufb4	ENSMUSG00000022820	chr16	37647256	37654539	-	1170	-4.064	-1
Slc5a6	ENSMUSG00000006641	chr5	31338409	31351297	-	3991	-4.065	-1
Ankrd22	ENSMUSG00000024774	chr19	34197039	34240543	-	1972	-4.065	-1
Gstz1	ENSMUSG00000021033	chr12	88488668	88505673	+	1656	-4.066	-1
Npdc1	ENSMUSG00000015094	chr2	25254871	25265014	+	3906	-4.067	-1
Gmppa	ENSMUSG00000033021	chr1	75432505	75439754	+	2395	-4.067	-1
Ncrna00086	ENSMUSG00000054850	chrX	53627787	53630976	+	2369	-4.067	-1
Kcnn1	ENSMUSG00000002908	chr8	73365948	73380907	-	4049	-4.07	-1
Mtg1	ENSMUSG00000039018	chr7	147323463	147336685	+	4019	-4.072	-1

Mri1	ENSMUSG00000004996	chr8	86773805	86781225	-	3273	-4.075	-1
Uros	ENSMUSG00000030979	chr7	140877926	140901755	-	3576	-4.077	-1
Card14	ENSMUSG00000013483	chr11	119169082	119206689	+	4605	-4.079	-1
Gm4535	ENSMUSG00000091083	chr7	148592536	148593093	+	558	-4.085	-1
Bdh1	ENSMUSG00000046598	chr16	31422366	31458987	+	3800	-4.086	-1
Hist1h4c	ENSMUSG00000060678	chr13	23789929	23790323	-	395	-4.088	-1
Tmem159	ENSMUSG00000030917	chr7	127245890	127264506	+	1926	-4.091	-1
Pold2	ENSMUSG00000020471	chr11	5772183	5778295	-	1896	-4.099	-1
Pfdn5	ENSMUSG00000001289	chr15	102156547	102162304	+	1394	-4.104	-1
Asl	ENSMUSG00000025533	chr5	130487128	130501002	-	4563	-4.106	-1
N6amt2	ENSMUSG00000021951	chr14	58168435	58190406	-	836	-4.106	-1
Psmg3	ENSMUSG00000029551	chr5	140299548	140302797	-	839	-4.106	-1
Clic3	ENSMUSG00000015093	chr2	25312358	25314296	+	1012	-4.11	-1
Gm17178	ENSMUSG00000090758	chr11	120550460	120570761	-	357	-4.111	-1
Acy3	ENSMUSG00000024866	chr19	3986661	3990007	+	1505	-4.115	-1
1110034G24Rik	ENSMUSG00000044991	chr2	132512667	132576791	+	2117	-4.116	-1
Vamp8	ENSMUSG00000050732	chr6	72335217	72340697	-	2311	-4.117	-1
Gorasp1	ENSMUSG00000032513	chr9	119834791	119846676	-	3483	-4.118	-1
Atp13a2	ENSMUSG00000036622	chr4	140542788	140563245	+	6000	-4.118	-1
1810063B07Rik	ENSMUSG00000045107	chr14	20894868	20902394	-	2294	-4.127	-1
Rac3	ENSMUSG00000018012	chr11	120582784	120585283	+	1293	-4.128	-1
Mrps28	ENSMUSG00000040269	chr3	8802146	8923918	-	783	-4.13	-1
Slc12a8	ENSMUSG00000035506	chr16	33517414	33664221	+	4444	-4.135	-1
Ears2	ENSMUSG00000030871	chr7	129180727	129210777	-	5152	-4.136	-1
Olfm4	ENSMUSG00000022026	chr14	80400109	80422667	+	2570	-4.136	-1
3110070M22Rik	ENSMUSG00000074635	chr13	120276068	120277190	-	1123	-4.139	-1
1810020D17Rik	ENSMUSG00000035642	chr7	104698844	104728007	-	1612	-4.141	-1
Npr1	ENSMUSG00000027931	chr3	90254513	90269788	-	4783	-4.144	-1
AI462493	ENSMUSG00000071654	chr19	8954506	8955423	-	829	-4.144	-1
Ttc22	ENSMUSG00000034919	chr4	106295037	106312794	+	3318	-4.146	-1
Ppp1r35	ENSMUSG00000029725	chr5	138220077	138221338	+	1085	-4.146	-1
Gm15954	ENSMUSG00000089979	chr16	18069307	18070455	+	466	-4.148	-1

1700040I03Rik	ENSMUSG00000030008	chr6	85396804	85401964	-	1594	-4.149	-1
1700009P17Rik	ENSMUSG00000026649	chr1	173044049	173057098	+	1774	-4.152	-1
Ssr2	ENSMUSG00000041355	chr3	88383593	88392319	+	1167	-4.153	-1
Fdps	ENSMUSG00000059743	chr3	88897514	88905881	-	1233	-4.153	-1
Fxn	ENSMUSG00000059363	chr19	24335943	24355095	-	2053	-4.157	-1
Gjc2	ENSMUSG00000043448	chr11	58989068	58996715	-	2260	-4.158	-1
Gm5805	ENSMUSG00000061848	chr15	81802804	81803322	+	519	-4.161	-1
Gchfr	ENSMUSG00000046814	chr2	118993509	118998126	+	645	-4.164	-1
Mvk	ENSMUSG00000041939	chr5	114894278	114910600	+	2674	-4.165	-1
Atp9a	ENSMUSG00000027546	chr2	168459938	168567909	-	5911	-4.166	-1
Polr2i	ENSMUSG00000019738	chr7	31016967	31018409	+	1212	-4.169	-1
Atp5e	ENSMUSG00000016252	chr2	174286573	174289606	-	1756	-4.173	-1
1110054M08Rik	ENSMUSG00000091210	chr16	24391698	24393674	-	1603	-4.175	-1
Btbd2	ENSMUSG00000003344	chr10	80105362	80119816	-	3586	-4.182	-1
1600002K03Rik	ENSMUSG00000035595	chr10	79635689	79637891	+	678	-4.183	-1
Enho	ENSMUSG00000028445	chr4	41585177	41587357	-	1269	-4.183	-1
Prkcz	ENSMUSG00000029053	chr4	154634238	154735470	-	6318	-4.187	-1
1810014F10Rik	ENSMUSG00000025466	chr7	147282669	147288340	-	3272	-4.188	-1
Armcx6	ENSMUSG00000050394	chrX	131282994	131285956	-	2509	-4.192	-1
9130213A22Rik	ENSMUSG00000090920	chr11	68933556	68936091	-	1581	-4.195	-1
Srd5a2	ENSMUSG00000038541	chr17	74367046	74397256	-	854	-4.201	-1
Fam115c	ENSMUSG00000029851	chr6	42573018	42595253	-	4775	-4.202	-1
Trpm4	ENSMUSG00000038260	chr7	52558002	52589150	-	4768	-4.204	-1
Lck	ENSMUSG00000000409	chr4	129225588	129250885	-	2929	-4.204	-1
Nptx2	ENSMUSG00000059991	chr5	145306756	145318347	+	2551	-4.208	-1
Myeov2	ENSMUSG00000073616	chr1	94533722	94538562	-	1499	-4.209	-1
Rps15a-ps2	ENSMUSG00000067351	chr19	28816649	28817041	-	393	-4.21	-1
2410133F24Rik	ENSMUSG00000086728	chr9	56947757	56979097	-	2157	-4.21	-1
Cyb5rl	ENSMUSG00000028621	chr4	106739593	106760873	+	7430	-4.211	-1
Alkbh6	ENSMUSG00000042831	chr7	31093736	31099323	+	3123	-4.213	-1
Abtb1	ENSMUSG00000030083	chr6	88785909	88791929	-	1884	-4.22	-1
Pgp	ENSMUSG00000043445	chr17	24607418	24608536	+	1027	-4.224	-1
Get4	ENSMUSG00000025858	chr5	139728278	139746005	+	2431	-4.225	-1
Ufsp1	ENSMUSG00000051502	chr5	137735897	137736892	+	996	-4.226	-1

Krtcap2	ENSMUSG00000042747	chr3	89049888	89053644	+	1260	-4.23	-1
Il15	ENSMUSG00000031712	chr8	84855536	84927121	-	1908	-4.231	-1
Pcp4	ENSMUSG00000090223	chr16	96689213	96747400	+	641	-4.232	-1
Ddx56	ENSMUSG00000004393	chr11	6158922	6167775	-	2533	-4.233	-1
Gm11767	ENSMUSG00000085207	chr11	119797117	119804012	-	493	-4.235	-1
Spon2	ENSMUSG00000037379	chr5	33556167	33560887	-	2237	-4.236	-1
Pgls	ENSMUSG00000031807	chr8	74116075	74124991	+	3770	-4.236	-1
Eci1	ENSMUSG00000024132	chr17	24563628	24576261	+	1091	-4.237	-1
Osbpl10	ENSMUSG00000040875	chr9	114976397	115141341	+	1872	-4.241	-1
Ovol1	ENSMUSG00000024922	chr19	5549137	5560575	-	2900	-4.242	-1
Morn2	ENSMUSG00000045257	chr17	80689552	80696813	+	644	-4.245	-1
Fkbp4	ENSMUSG00000030357	chr6	128379753	128388695	-	2795	-4.247	-1
1700007L15Rik	ENSMUSG00000090918	chr16	33379941	33380813	-	737	-4.248	-1
Pam16	ENSMUSG00000014301	chr16	4616464	4624988	-	857	-4.248	-1
Stub1	ENSMUSG00000039615	chr17	25967581	25970306	-	1672	-4.249	-1
Gm10029	ENSMUSG00000068324	chr13	6661765	6662187	-	423	-4.251	-1
Gm12750	ENSMUSG00000085873	chr4	109076399	109078760	-	858	-4.251	-1
Nr2c2ap	ENSMUSG00000071078	chr8	72655232	72657638	+	1175	-4.251	-1
Pde9a	ENSMUSG00000041119	chr17	31523179	31613255	+	2834	-4.254	-1
Tmem147	ENSMUSG00000006315	chr7	31512720	31514553	-	868	-4.258	-1
Clpp	ENSMUSG00000002660	chr17	57129687	57135794	+	1207	-4.258	-1
4632428C04Rik	ENSMUSG00000086897	chr16	30008753	30021516	+	2430	-4.269	-1
Stra13	ENSMUSG00000025144	chr11	120572256	120575052	-	1077	-4.271	-1
9930023K05Rik	ENSMUSG00000035818	chr19	56536127	56561219	+	2385	-4.271	-1
Rspo1	ENSMUSG00000028871	chr4	124663674	124686343	+	1938	-4.278	-1
Prrt1	ENSMUSG00000015476	chr17	34766478	34770071	+	2306	-4.28	-1
Dbp	ENSMUSG00000059824	chr7	52960458	52965525	+	2945	-4.283	-1
Mpi	ENSMUSG00000032306	chr9	57392063	57400570	-	3142	-4.283	-1
Cope	ENSMUSG00000055681	chr8	72826417	72836892	+	3353	-4.284	-1
Esrp1	ENSMUSG00000040728	chr4	11259080	11313930	-	6305	-4.285	-1
Pygm	ENSMUSG00000032648	chr19	6384399	6398459	+	3218	-4.289	-1
Cog8	ENSMUSG00000031916	chr8	109570189	109580589	-	4637	-4.291	-1
Pex10	ENSMUSG00000029047	chr4	154441125	154446542	+	3298	-4.293	-1
Tmem218	ENSMUSG00000032121	chr9	37015918	37030795	+	1019	-4.293	-1
H2afj	ENSMUSG00000060032	chr6	136756769	136758595	+	1827	-4.296	-1
Epha1	ENSMUSG00000029859	chr6	42308486	42323267	-	3273	-4.297	-1
Clec2d	ENSMUSG00000030157	chr6	129130633	129136552	+	1213	-4.298	-1

Cib3	ENSMUSG00000074240	chr8	74728234	74736736	-	646	-4.3	-1
Ighv1-66	ENSMUSG00000076728	chr12	116831321	116831754	-	351	-4.302	-1
Dnajc12	ENSMUSG00000036764	chr10	62845191	62871588	+	2740	-4.302	-1
6030429G01Rik	ENSMUSG00000055809	chr7	4474535	4484055	-	2224	-4.303	-1
Tmem219	ENSMUSG00000060538	chr7	134029685	134066431	-	2292	-4.304	-1
Slc9a3r2	ENSMUSG00000002504	chr17	24776227	24787250	-	2257	-4.308	-1
Nudt16l1	ENSMUSG00000022516	chr16	4939111	4941025	+	1274	-4.308	-1
0610040B10Rik	ENSMUSG00000089889	chr5	144090926	144094383	+	683	-4.31	-1
Prss36	ENSMUSG00000070371	chr7	135076152	135090239	-	3221	-4.312	-1
Naglu	ENSMUSG00000001751	chr11	100931326	100938986	+	2658	-4.325	-1
2810405K02Rik	ENSMUSG00000029059	chr4	154269613	154273244	-	1027	-4.325	-1
Dcaf4	ENSMUSG00000021222	chr12	84861416	84882938	+	2081	-4.331	-1
Fbp2	ENSMUSG00000021456	chr13	62938244	62959760	-	1325	-4.331	-1
Reep6	ENSMUSG00000035504	chr10	79792698	79799186	+	2506	-4.332	-1
Gm17507	ENSMUSG00000091428	chr18	35024439	35024763	-	36	-4.339	-1
Use1	ENSMUSG00000002395	chr8	73890747	73893631	+	2203	-4.343	-1
Papln	ENSMUSG00000021223	chr12	85104584	85133332	+	5635	-4.344	-1
Prg2	ENSMUSG00000027073	chr2	84820618	84823789	+	838	-4.347	-1
Fam26f	ENSMUSG00000046031	chr10	33845875	33847778	-	1080	-4.347	-1
Tspan15	ENSMUSG00000037031	chr10	61648144	61693974	-	3613	-4.351	-1
Cln6	ENSMUSG00000032245	chr9	62686592	62699813	+	2434	-4.352	-1
Copz2	ENSMUSG00000018672	chr11	96711184	96722517	+	2208	-4.354	-1
Zfr2	ENSMUSG00000034949	chr10	80695900	80714868	+	7492	-4.354	-1
Cdk5rap3	ENSMUSG00000018669	chr11	96768740	96777810	-	3812	-4.363	-1
Ppp1r37	ENSMUSG00000051403	chr7	20116316	20147747	-	2971	-4.363	-1
Chad	ENSMUSG00000039084	chr11	94426361	94430441	+	1680	-4.37	-1
Uqcrq	ENSMUSG00000044894	chr11	53241424	53244333	-	1831	-4.37	-1
Pla2g16	ENSMUSG00000060675	chr19	7631949	7663035	+	6396	-4.372	-1
1110031I02Rik	ENSMUSG00000025169	chr11	121038905	121066025	-	1308	-4.377	-1
Batf3	ENSMUSG00000026630	chr1	192922293	192932822	+	712	-4.389	-1
Gm15492	ENSMUSG00000085012	chr6	113258660	113276741	-	606	-4.389	-1
Fam173a	ENSMUSG00000057411	chr17	25927453	25929332	-	857	-4.394	-1

Lhpp	ENSMUSG00000030946	chr7	139802321	139898103	+	2085	-4.395	-1
Mfsd3	ENSMUSG00000019080	chr15	76531972	76534669	+	2169	-4.398	-1
Rorc	ENSMUSG00000028150	chr3	94176716	94202198	+	3566	-4.409	-1
Mrps24	ENSMUSG00000020477	chr11	5603986	5615683	-	1466	-4.41	-1
Gadd45gip1	ENSMUSG00000033751	chr8	87355421	87359381	+	2494	-4.41	-1
1810012P15Rik	ENSMUSG00000072640	chr11	78640086	78660372	+	8146	-4.411	-1
Tomm40l	ENSMUSG00000005674	chr1	173147933	173152645	-	3344	-4.413	-1
Kiss1r	ENSMUSG00000035773	chr10	79379716	79385018	+	3163	-4.419	-1
Galnt9	ENSMUSG00000033316	chr5	110973364	111050401	+	2997	-4.421	-1
Rell2	ENSMUSG00000044024	chr18	38114733	38118833	+	3340	-4.421	-1
Tppp3	ENSMUSG00000014846	chr8	107991393	107995426	-	1848	-4.422	-1
Tha1	ENSMUSG00000017713	chr11	117729266	117734795	-	2910	-4.431	-1
Gm4968	ENSMUSG00000062365	chr6	127183774	127184637	+	864	-4.437	-1
Gm5908	ENSMUSG00000065950	chr8	33587355	33587801	-	447	-4.437	-1
Ripk4	ENSMUSG00000005251	chr16	97963540	97985344	-	3953	-4.444	-1
Morn1	ENSMUSG00000029049	chr4	154460686	154519614	+	4320	-4.445	-1
D10Jhu81e	ENSMUSG00000053329	chr10	77624812	77632513	-	1363	-4.45	-1
Trabd	ENSMUSG00000015363	chr15	88905554	88917507	+	5591	-4.454	-1
1810021B22Rik	ENSMUSG00000087331	chr15	88901626	88906282	-	896	-4.455	-1
Apoa1bp	ENSMUSG00000028070	chr3	87860445	87862417	-	905	-4.459	-1
Mettl1	ENSMUSG00000006732	chr10	126478470	126483421	+	2907	-4.461	-1
Fpgs	ENSMUSG00000009566	chr2	32538129	32559657	-	3794	-4.466	-1
2010001M09Rik	ENSMUSG00000024353	chr18	35806920	35809021	-	905	-4.468	-1
Hist4h4	ENSMUSG00000068851	chr6	136752589	136752900	-	312	-4.471	-1
Ccdc64b	ENSMUSG00000043782	chr17	23797490	23805577	+	1913	-4.472	-1
Hmgcl	ENSMUSG00000028672	chr4	135502363	135518532	+	2434	-4.472	-1
Yif1b	ENSMUSG00000030588	chr7	30023348	30032612	+	1378	-4.473	-1
Tstd1	ENSMUSG00000091166	chr1	173349164	173350483	+	551	-4.474	-1
Entpd6	ENSMUSG00000033068	chr2	150574778	150597411	+	4775	-4.475	-1
Dcaf11	ENSMUSG00000022214	chr14	56178843	56188902	+	8070	-4.475	-1
Vmac	ENSMUSG00000054723	chr17	56853355	56857122	-	2276	-4.487	-1
Pccb	ENSMUSG00000032527	chr9	100882451	100935317	-	2771	-4.488	-1
Rhbdd3	ENSMUSG00000034175	chr11	4998929	5006096	+	2790	-4.493	-1
Ccbl2	ENSMUSG00000040213	chr3	142364015	142407874	+	2619	-4.495	-1
Mrpl14	ENSMUSG00000023939	chr17	45823271	45835444	+	949	-4.498	-1

1810043H04Rik	ENSMUSG00000078572	chr11	119960242	119962018	+	780	-4.499	-1
Ndufa7	ENSMUSG00000041881	chr17	33961536	33975261	+	1701	-4.499	-1
Fam158a	ENSMUSG00000022217	chr14	56200353	56204139	-	2465	-4.501	-1
Herpud1	ENSMUSG00000031770	chr8	96910338	96919277	+	2333	-4.501	-1
Cldn4	ENSMUSG00000047501	chr5	135420996	135422804	-	1809	-4.505	-1
Pla2g12a	ENSMUSG00000027999	chr3	129581524	129598739	+	1670	-4.507	-1
Mrps34	ENSMUSG00000038880	chr17	25032061	25034447	+	2064	-4.511	-1
Jph2	ENSMUSG00000017817	chr2	163161978	163223729	-	4255	-4.515	-1
Ttc39a	ENSMUSG00000028555	chr4	109079228	109117350	+	4904	-4.516	-1
Gm11538	ENSMUSG00000085983	chr11	96064768	96066794	+	1945	-4.517	-1
Poll	ENSMUSG00000025218	chr19	45626765	45635021	-	2324	-4.517	-1
Alox12	ENSMUSG00000000320	chr11	70054959	70068855	-	3985	-4.517	-1
5133401N09Rik	ENSMUSG00000050002	chr13	58259015	58266052	+	1504	-4.519	-1
Gm12999	ENSMUSG00000087352	chr4	132088602	132089516	+	485	-4.522	-1
Gm13205	ENSMUSG00000086606	chr4	148321601	148323023	-	562	-4.522	-1
Gm7589	ENSMUSG00000066592	chr9	58993481	58994017	-	537	-4.522	-1
Icam4	ENSMUSG00000001014	chr9	20833817	20834975	+	907	-4.522	-1
Pex6	ENSMUSG00000002763	chr17	46848412	46862490	+	3796	-4.527	-1
Slc38a5	ENSMUSG00000031170	chrX	7848259	7857305	+	2241	-4.528	-1
Fbxl22	ENSMUSG00000050503	chr9	66356266	66362400	-	3935	-4.533	-1
Abhd14b	ENSMUSG00000042073	chr9	106350971	106355246	+	1558	-4.534	-1
Alad	ENSMUSG00000028393	chr4	62170203	62180952	-	1871	-4.534	-1
Bola1	ENSMUSG00000015943	chr3	96000511	96023632	-	1082	-4.536	-1
Gcdh	ENSMUSG00000003809	chr8	87410292	87417820	-	2754	-4.537	-1
Sec61b	ENSMUSG00000053317	chr4	47487530	47496114	+	1271	-4.542	-1
Ddt	ENSMUSG00000001666	chr10	75233975	75236159	-	773	-4.543	-1
D630003M21Rik	ENSMUSG00000037813	chr2	158008269	158054958	-	5104	-4.544	-1
1190007F08Rik	ENSMUSG00000078350	chr4	153394697	153400261	-	5070	-4.547	-1
Gm14154	ENSMUSG00000087004	chr2	151773009	151776377	-	579	-4.549	-1
Gm5614	ENSMUSG00000066771	chr9	35089103	35089420	-	318	-4.549	-1
Rps4y2	ENSMUSG00000063171	chr6	148303178	148304120	+	943	-4.55	-1

4930412O13Rik	ENSMUSG00000025783	chr2	9802879	9805548	+	1381	-4.552	-1
Isoc2a	ENSMUSG00000086784	chr7	4828655	4847319	+	1088	-4.554	-1
Neu1	ENSMUSG00000007038	chr17	35068198	35072898	+	3733	-4.558	-1
Rdm1	ENSMUSG00000010362	chr11	101488509	101497414	+	1575	-4.559	-1
Casz1	ENSMUSG00000028977	chr4	148178538	148328998	+	12240	-4.559	-1
Slc52a3	ENSMUSG00000027463	chr2	151822247	151834994	+	2767	-4.568	-1
1700021C14Rik	ENSMUSG00000010538	chr3	88084043	88100921	-	1144	-4.571	-1
Ttc38	ENSMUSG00000035944	chr15	85662734	85689252	+	3844	-4.572	-1
Rasef	ENSMUSG00000043003	chr4	73360483	73436613	-	5461	-4.575	-1
Hspb6	ENSMUSG00000036854	chr7	31337197	31340462	+	2299	-4.578	-1
Aldh7a1	ENSMUSG00000053644	chr18	56669341	56732605	-	5181	-4.581	-1
Mmp17	ENSMUSG00000029436	chr5	130090089	130114086	+	3489	-4.582	-1
Pgap3	ENSMUSG00000038208	chr11	98249991	98261804	-	3248	-4.583	-1
Trappc6a	ENSMUSG00000002043	chr7	20094019	20101494	+	1317	-4.584	-1
Aldh1l1	ENSMUSG00000030088	chr6	90436421	90550197	+	4512	-4.584	-1
Ctsf	ENSMUSG00000083282	chr19	4855129	4860912	+	1975	-4.586	-1
Ddost	ENSMUSG00000028757	chr4	137860645	137868543	+	2120	-4.587	-1
Slco1a5	ENSMUSG00000063975	chr6	142182748	142271501	-	3729	-4.591	-1
Cnksr1	ENSMUSG00000028841	chr4	133783956	133794314	-	2804	-4.593	-1
Timm17b	ENSMUSG00000031158	chrX	7476483	7485477	+	2557	-4.593	-1
Csrp1	ENSMUSG00000026421	chr1	137616638	137648809	+	1956	-4.595	-1
Ggnbp1	ENSMUSG00000048731	chr17	27110162	27173323	+	6943	-4.598	-1
Cldn3	ENSMUSG00000070473	chr5	135462084	135463342	+	1259	-4.607	-1
Gm4890	ENSMUSG00000085259	chr8	81819022	81831584	+	2508	-4.61	-1
5830444B04Rik	ENSMUSG00000084803	chr4	154772872	154795813	-	5410	-4.615	-1
Prph	ENSMUSG00000023484	chr15	98885605	98889409	+	1865	-4.623	-1
Sel1l3	ENSMUSG00000029189	chr5	53498323	53604691	-	4511	-4.629	-1
Gm20492	ENSMUSG00000092593	chr10	126725570	126726737	+	286	-4.63	-1
Abhd16a	ENSMUSG00000007036	chr17	35226208	35239932	+	3222	-4.63	-1
Klf15	ENSMUSG00000030087	chr6	90412570	90425232	+	2527	-4.631	-1
Cma1	ENSMUSG00000022225	chr14	56560290	56563498	-	1074	-4.64	-1
Pycr1	ENSMUSG00000025140	chr11	120497026	120505083	-	3935	-4.646	-1
Etfb	ENSMUSG00000004610	chr7	50699478	50713170	+	1139	-4.647	-1
Rasd2	ENSMUSG00000034472	chr8	77737843	77748012	+	3176	-4.648	-1
B9d1	ENSMUSG00000001039	chr11	61318646	61326433	+	1593	-4.649	-1
Kptn	ENSMUSG00000006021	chr7	16705244	16712865	+	3341	-4.652	-1

Atg4d	ENSMUSG00000002820	chr9	21069743	21078812	+	2541	-4.655	-1
Ramp1	ENSMUSG00000034353	chr1	93076399	93121773	+	3022	-4.658	-1
Acta2	ENSMUSG00000035783	chr19	34315580	34329826	-	1781	-4.658	-1
Jmjd8	ENSMUSG00000025736	chr17	25965812	25968787	+	2758	-4.66	-1
Wnt5b	ENSMUSG00000030170	chr6	119382549	119494336	-	2625	-4.662	-1
BC029214	ENSMUSG00000047617	chr2	25310661	25316614	-	2066	-4.663	-1
Mosc2	ENSMUSG00000073481	chr1	186636947	186670330	-	6629	-4.668	-1
C130040N14Rik	ENSMUSG00000073429	chr17	27702057	27703165	+	1109	-4.672	-1
Tagln	ENSMUSG00000032085	chr9	45737711	45744141	-	1586	-4.678	-1
B3galt4	ENSMUSG00000067370	chr17	34086859	34088433	-	1575	-4.684	-1
Cox4i1	ENSMUSG00000031818	chr8	123192190	123198109	+	679	-4.69	-1
Nkd1	ENSMUSG00000031661	chr8	91045243	91118786	+	4459	-4.691	-1
Fam125a	ENSMUSG00000031813	chr8	74066829	74071923	+	1060	-4.693	-1
Clec4b1	ENSMUSG00000030147	chr6	122999980	123021573	+	784	-4.693	-1
Sardh	ENSMUSG00000009614	chr2	27043913	27103857	-	5432	-4.698	-1
Phf19	ENSMUSG00000026873	chr2	34749277	34769546	-	5036	-4.703	-1
Izumo4	ENSMUSG00000055862	chr10	80165438	80168127	+	938	-4.704	-1
1110065P20Rik	ENSMUSG00000078570	chr4	124526729	124527974	-	929	-4.705	-1
Slc1a5	ENSMUSG00000001918	chr7	17366695	17383623	+	4156	-4.707	-1
Kctd14	ENSMUSG00000051727	chr7	104599844	104608063	+	2553	-4.707	-1
Slc35b2	ENSMUSG00000037089	chr17	45700909	45704619	+	2722	-4.708	-1
Smpd2	ENSMUSG00000019822	chr10	41205448	41210175	-	4468	-4.716	-1
Ndufb2	ENSMUSG00000002416	chr6	39542573	39553381	+	5628	-4.722	-1
Mpv17l2	ENSMUSG00000035559	chr8	73282548	73284820	-	955	-4.725	-1
Pycr2	ENSMUSG00000026520	chr1	182834405	182838218	+	1598	-4.726	-1
Igfbp6	ENSMUSG00000023046	chr15	101974793	101979942	+	1112	-4.728	-1
Tsta3	ENSMUSG00000022570	chr15	75755114	75760160	-	1311	-4.731	-1
Uqcr11	ENSMUSG00000020163	chr10	79865742	79869575	-	641	-4.734	-1
Pex16	ENSMUSG00000027222	chr2	92214833	92221374	+	1775	-4.744	-1
Tmc4	ENSMUSG00000019734	chr7	3617392	3629126	-	3859	-4.748	-1
Eci3	ENSMUSG00000021416	chr13	35038483	35055679	-	1418	-4.749	-1
Pomc	ENSMUSG00000020660	chr12	3954951	3960618	+	1007	-4.749	-1
Ppp1r14a	ENSMUSG00000037166	chr7	30074339	30078409	+	531	-4.751	-1
2010001A14Rik	ENSMUSG00000087165	chr11	57609001	57614919	-	714	-4.751	-1
Gxylt2	ENSMUSG00000030074	chr6	100654728	100755075	+	2678	-4.755	-1
Cml1	ENSMUSG00000057103	chr6	85860148	85865681	-	1345	-4.757	-1
Gm8973	ENSMUSG00000064081	chr15	98836487	98836801	+	315	-4.759	-1

Retsat	ENSMUSG00000056666	chr6	72548469	72558419	+	3537	-4.76	-1
Pnpo	ENSMUSG00000018659	chr11	96799139	96805300	-	2925	-4.761	-1
Gale	ENSMUSG00000028671	chr4	135519642	135524093	+	2370	-4.763	-1
Dexi	ENSMUSG00000038055	chr16	10530300	10543147	-	1389	-4.763	-1
2310001 H17Rik	ENSMUSG00000085930	chr6	129158430	129188000	-	5694	-4.764	-1
Fuca1	ENSMUSG00000028673	chr4	135476650	135496226	+	3276	-4.767	-1
Acss2	ENSMUSG00000027605	chr2	155343684	155411460	+	10163	-4.767	-1
2700046 G09Rik	ENSMUSG00000024893	chr19	32463706	32465674	+	1641	-4.767	-1
Pkp3	ENSMUSG00000054065	chr7	148264117	148276409	+	3616	-4.771	-1
1700113 A16Rik	ENSMUSG00000086082	chr3	87975482	87981707	-	983	-4.772	-1
Tcta	ENSMUSG00000039461	chr9	108205290	108208282	-	1628	-4.778	-1
Scand1	ENSMUSG00000046229	chr2	156137583	156138440	-	759	-4.779	-1
Pdk2	ENSMUSG00000038967	chr11	94887572	94902668	-	3163	-4.781	-1
Gabrd	ENSMUSG00000029054	chr4	154759089	154772221	-	2875	-4.79	-1
0610039 K10Rik	ENSMUSG00000058812	chr2	163470586	163471536	+	951	-4.793	-1
Pigyl	ENSMUSG00000010607	chr9	21961290	21962802	+	777	-4.793	-1
Gpt2	ENSMUSG00000031700	chr8	88016475	88051459	+	5643	-4.795	-1
Slc6a4	ENSMUSG00000020838	chr11	76812105	76845842	+	4529	-4.795	-1
Ccdc92	ENSMUSG00000037979	chr5	125314788	125342794	-	2731	-4.799	-1
Ipo13	ENSMUSG00000033365	chr4	117567091	117587604	-	4279	-4.803	-1
Phgdh	ENSMUSG00000053398	chr3	98117093	98143913	-	2486	-4.806	-1
Mpst	ENSMUSG00000071711	chr15	78237142	78244432	+	1515	-4.806	-1
2310039 H08Rik	ENSMUSG00000062619	chr17	46909584	46910356	+	773	-4.81	-1
BC0497 62	ENSMUSG00000045942	chr11	51067153	51076548	-	935	-4.816	-1
Cd3d	ENSMUSG00000032094	chr9	44789876	44795135	+	1323	-4.818	-1
Mt1	ENSMUSG00000031765	chr8	96702989	96704225	+	753	-4.823	-1
Nhlrc1	ENSMUSG00000044231	chr13	47107927	47110219	-	2293	-4.823	-1
1010001 B22Rik	ENSMUSG00000091434	chr5	110424530	110425417	-	589	-4.823	-1
Dand5	ENSMUSG00000053226	chr8	87339304	87356140	-	1656	-4.825	-1
1700037 C18Rik	ENSMUSG00000005983	chr16	3895179	3908689	-	3227	-4.826	-1
Adck3	ENSMUSG00000026489	chr1	182095369	182129733	-	5293	-4.827	-1
Tmem1 25	ENSMUSG00000050854	chr4	118213546	118216649	-	2339	-4.828	-1
Fam71e 1	ENSMUSG00000051113	chr7	51751958	51756856	+	1147	-4.832	-1

Mcpt4	ENSMUSG00000061068	chr14	56678581	56681147	-	990	-4.837		-1
Chchd6	ENSMUSG00000030086	chr6	89333140	89545646	-	2527	-4.843		-1
Fabp3	ENSMUSG00000028773	chr4	129985839	129992707	+	823	-4.851		-1
Krtcap3	ENSMUSG00000029149	chr5	31554064	31555575	+	1338	-4.852		-1
Rnf43	ENSMUSG00000034177	chr11	87476224	87549041	+	6558	-4.863		-1
Wfdc1	ENSMUSG00000023336	chr8	122190423	122212122	+	1213	-4.863		-1
Prrg2	ENSMUSG00000007837	chr7	52308983	52318047	-	2091	-4.863		-1
Glt1d1	ENSMUSG00000049971	chr5	128112632	128189124	+	3758	-4.863		-1
Ccdc56	ENSMUSG00000017188	chr11	101139282	101140428	-	886	-4.863		-1
Tmem2 23	ENSMUSG00000075043	chr19	8845486	8846965	+	748	-4.87		-1
Lmod1	ENSMUSG00000048096	chr1	137221384	137264642	+	3948	-4.872		-1
Il17rc	ENSMUSG00000030281	chr6	113421421	113433134	+	2671	-4.873		-1
Llgl2	ENSMUSG00000020782	chr11	115685363	115717094	+	4772	-4.877		-1
Gm6723	ENSMUSG00000068226	chr15	78384832	78385278	-	447	-4.883		-1
Gm1294 2	ENSMUSG00000070737	chr4	126800935	126806888	+	1910	-4.893		-1
2410022 L05Rik	ENSMUSG00000031458	chr8	13884788	13890271	-	802	-4.895		-1
D63003 9A03Rik	ENSMUSG00000052117	chr4	57921355	57929169	-	2830	-4.898		-1
Gm1586 0	ENSMUSG00000087336	chr5	123784341	123794446	-	815	-4.899		-1
n- R5s156	ENSMUSG00000065311	chr7	106646228	106646332	+	105	-4.9		-1
2900053 A13Rik	ENSMUSG00000087687	chr8	3621575	3624227	+	1161	-4.902		-1
Pycrl	ENSMUSG00000022571	chr15	75746893	75751990	-	1353	-4.906		-1
Map1lc3 a	ENSMUSG00000027602	chr2	155102033	155103809	+	1181	-4.911		-1
Ldhb	ENSMUSG00000030246	chr6	142438769	142456477	-	1570	-4.916		-1
Pmel	ENSMUSG00000025359	chr10	128143314	128157294	+	2133	-4.921		-1
Popdc2	ENSMUSG00000022803	chr16	38362295	38378302	+	2360	-4.921		-1
Lamc3	ENSMUSG00000026840	chr2	31742811	31802059	+	6091	-4.921		-1
C85492	ENSMUSG00000066235	chr9	121890724	121905144	-	2369	-4.925		-1
K23001 0J24Rik	ENSMUSG00000075594	chr15	75839499	75865669	+	1123	-4.926		-1
Dpm3	ENSMUSG00000042737	chr3	89063280	89071001	+	919	-4.926		-1
6430573 F11Rik	ENSMUSG00000039620	chr8	37520602	37577374	+	4154	-4.926		-1
Lrrc29	ENSMUSG00000041679	chr8	107836257	107850178	-	1908	-4.934		-1
Gm1590 3	ENSMUSG00000086916	chr5	130232921	130234260	+	310	-4.943		-1
Gm7866	ENSMUSG00000061390	chr9	72022392	72022787	-	396	-4.943		-1

Mpnd	ENSMUSG00000003199	chr17	56145134	56156213	+	2305	-4.949	-1
Amigo1	ENSMUSG00000050947	chr3	107989253	107995204	+	5540	-4.949	-1
Alkbh7	ENSMUSG00000002661	chr17	57136762	57138745	+	1029	-4.949	-1
Krt14	ENSMUSG00000045545	chr11	100064476	100068862	-	1758	-4.957	-1
1810046J19Rik	ENSMUSG00000002580	chr11	98299022	98300305	-	747	-4.971	-1
Hist1h4d	ENSMUSG00000061482	chr13	23673467	23673859	+	393	-4.976	-1
Tmem42	ENSMUSG00000066233	chr9	122930444	122932608	+	1044	-4.98	-1
Gpt	ENSMUSG00000022546	chr15	76527163	76530104	+	1780	-4.981	-1
2210411K11Rik	ENSMUSG00000030431	chr7	4736197	4741258	-	1337	-4.987	-1
Ormdl3	ENSMUSG00000038150	chr11	98442570	98448682	-	2184	-4.988	-1
Chn2	ENSMUSG00000004633	chr6	53989548	54251804	+	5745	-4.994	-1
Arhgef10l	ENSMUSG00000040964	chr4	140070400	140221927	-	5693	-4.998	-1
1700001G17Rik	ENSMUSG00000044744	chr1	33726688	33727557	+	870	-4.999	-1
0610009L18Rik	ENSMUSG00000043644	chr11	120209992	120212504	+	619	-5.004	-1
Casq2	ENSMUSG00000027861	chr3	101890338	101950437	+	2736	-5.009	-1
Itpk1	ENSMUSG00000057963	chr12	103806793	103943079	-	2856	-5.01	-1
Sectm1b	ENSMUSG00000039364	chr11	120914772	120924883	-	2780	-5.011	-1
Mrps12	ENSMUSG00000045948	chr7	29524660	29526839	-	1040	-5.011	-1
Angel1	ENSMUSG00000021257	chr12	88041452	88067410	-	4463	-5.019	-1
Lrfn1	ENSMUSG00000030600	chr7	29237257	29252567	+	2729	-5.019	-1
Krt1	ENSMUSG00000046834	chr15	101675859	101681217	-	2445	-5.022	-1
Tmem51	ENSMUSG00000040616	chr4	141586907	141640219	-	1875	-5.026	-1
Naprt1	ENSMUSG00000022574	chr15	75721394	75724911	-	1890	-5.04	-1
Gm10320	ENSMUSG00000092116	chr13	99259303	99261956	-	527	-5.042	-1
Ctf1	ENSMUSG00000042340	chr7	134856250	134861699	+	1345	-5.045	-1
Prss53	ENSMUSG00000044139	chr7	135029355	135034484	-	2134	-5.048	-1
Akr7a5	ENSMUSG00000028743	chr4	138866659	138874341	+	1793	-5.057	-1
Gm5617	ENSMUSG00000042293	chr9	48303450	48304069	+	620	-5.06	-1
Pop5	ENSMUSG00000060152	chr5	115685845	115695360	+	2296	-5.063	-1
Mfsd7c	ENSMUSG00000034258	chr12	87087489	87154535	+	3435	-5.066	-1
Elf5	ENSMUSG00000027186	chr2	103251845	103291146	+	5877	-5.07	-1
Efcab4a	ENSMUSG00000048200	chr7	148646993	148652512	+	2764	-5.076	-1
Chchd10	ENSMUSG00000049422	chr10	75398318	75400470	+	939	-5.079	-1
A3galt2	ENSMUSG00000028794	chr4	128432608	128446542	+	3588	-5.079	-1

Ogfod2	ENSMUSG00000023707	chr5	124562306	124565492	+	2260	-5.082	-1
Abcb6	ENSMUSG00000026198	chr1	75168291	75176966	-	5315	-5.083	-1
Trex2	ENSMUSG00000031372	chrX	70679044	70680683	-	1017	-5.086	-1
Arhgap8	ENSMUSG00000078954	chr15	84550482	84602637	+	3535	-5.09	-1
1810059H22Rik	ENSMUSG00000085455	chr5	149765324	149781698	-	2897	-5.096	-1
Upk3b	ENSMUSG00000042985	chr5	136514366	136521173	+	2346	-5.099	-1
Ly6k	ENSMUSG00000044678	chr15	74627304	74630416	-	829	-5.106	-1
Shpk	ENSMUSG00000005951	chr11	73012962	73038013	+	2985	-5.107	-1
Tbx1	ENSMUSG00000009097	chr16	18581806	18588113	-	1811	-5.112	-1
Gstt3	ENSMUSG00000001665	chr10	75236860	75244159	-	2627	-5.121	-1
Gm9726	ENSMUSG00000051031	chr12	95166150	95166830	-	681	-5.123	-1
Gucy2g	ENSMUSG00000055523	chr19	55272787	55315726	-	3690	-5.129	-1
1700001O22Rik	ENSMUSG00000044320	chr2	30650289	30659181	-	2788	-5.13	-1
Rtn2	ENSMUSG00000030401	chr7	19868011	19881513	+	2034	-5.132	-1
Epb4.1l4b	ENSMUSG00000028434	chr4	57004844	57156309	-	8858	-5.134	-1
Gm166	ENSMUSG00000057176	chr7	134726602	134732109	-	1831	-5.137	-1
Gstt1	ENSMUSG00000001663	chr10	75246558	75261329	-	1528	-5.14	-1
Asb2	ENSMUSG00000021200	chr12	104559352	104594211	-	3542	-5.148	-1
Krt15	ENSMUSG00000054146	chr11	99993072	99997263	-	1788	-5.153	-1
Acad12	ENSMUSG00000042647	chr5	122048293	122068947	-	5007	-5.156	-1
P4htm	ENSMUSG00000006675	chr9	108481157	108499931	-	1810	-5.165	-1
2210021J22Rik	ENSMUSG00000064284	chr15	85637402	85642127	-	1215	-5.168	-1
C630043F03Rik	ENSMUSG00000084910	chr4	71862382	71876386	+	2581	-5.169	-1
Hddc3	ENSMUSG00000030532	chr7	87488023	87490983	+	860	-5.173	-1
Amhr2	ENSMUSG00000023047	chr15	102275798	102285061	+	2002	-5.181	-1
Coch	ENSMUSG00000020953	chr12	52694361	52706760	+	2601	-5.181	-1
Cited4	ENSMUSG00000070803	chr4	120339168	120340425	+	1258	-5.185	-1
Bik	ENSMUSG00000016758	chr15	83357292	83375065	+	943	-5.185	-1
Nudt14	ENSMUSG00000002804	chr12	114172944	114180329	-	793	-5.192	-1
Entpd3	ENSMUSG00000041608	chr9	120448936	120477435	+	3746	-5.193	-1
Gzma	ENSMUSG00000023132	chr13	113884033	113891187	-	939	-5.194	-1
Hdhd3	ENSMUSG00000038422	chr4	62160042	62164501	-	1366	-5.196	-1
Thnsl2	ENSMUSG00000054474	chr6	71078160	71094340	-	2071	-5.196	-1
Mlycd	ENSMUSG00000074064	chr8	121918778	121935002	+	2983	-5.2	-1
Pcx	ENSMUSG00000024892	chr19	4510472	4621752	+	4272	-5.202	-1
Gm16984	ENSMUSG00000085962	chr13	35055851	35082321	+	1018	-5.205	-1
Mcee	ENSMUSG00000033429	chr7	71537531	71557007	+	824	-5.205	-1
Spint2	ENSMUSG00000074227	chr7	30041350	30066996	-	1514	-5.207	-1

Paqr5	ENSMUSG00000032278	chr9	61801288	61874663	-	3954	-5.208	-1
Rabac1	ENSMUSG00000003380	chr7	25754769	25757747	-	875	-5.211	-1
Gm12843	ENSMUSG00000085749	chr4	116968422	116970330	+	891	-5.213	-1
Gm17619	ENSMUSG00000091760	chr2	170017949	170019553	-	1449	-5.213	-1
Gm16124	ENSMUSG00000086914	chr9	53475351	53489370	+	516	-5.213	-1
1110001J03Rik	ENSMUSG00000019689	chr6	38483502	38489449	+	689	-5.214	-1
Sil1	ENSMUSG00000024357	chr18	35426054	35658579	-	1695	-5.221	-1
Acot5	ENSMUSG00000042540	chr12	85410275	85416970	+	1519	-5.224	-1
1500011B03Rik	ENSMUSG00000092279	chr5	115262802	115263954	-	579	-5.236	-1
Tmprss13	ENSMUSG00000037129	chr9	45127183	45155664	+	3245	-5.238	-1
Limch1	ENSMUSG00000037736	chr5	67137074	67448398	+	7411	-5.246	-1
Ephx1	ENSMUSG00000038776	chr1	182906286	182951035	-	2005	-5.247	-1
Gm16894	ENSMUSG00000086705	chr1	40133073	40142698	-	844	-5.249	-1
Echdc2	ENSMUSG00000028601	chr4	107838042	107851913	+	2287	-5.249	-1
Rpl27a-ps1	ENSMUSG00000061488	chr10	60772569	60773065	+	497	-5.252	-1
Gm16136	ENSMUSG00000089804	chr15	37161676	37163064	-	1166	-5.255	-1
C630004H02Rik	ENSMUSG00000034586	chr11	115209021	115229070	-	4595	-5.256	-1
Gm5474	ENSMUSG00000067657	chr15	100127542	100128062	+	521	-5.263	-1
Exosc5	ENSMUSG00000061286	chr7	26444172	26453050	+	1030	-5.264	-1
Fam119b	ENSMUSG00000080115	chr10	126468238	126478569	-	2541	-5.267	-1
A830039N20Rik	ENSMUSG00000071567	chr14	24111513	24112865	-	1353	-5.272	-1
Khk	ENSMUSG00000029162	chr5	31223929	31233619	+	1670	-5.276	-1
Myl9	ENSMUSG00000067818	chr2	156601156	156607394	+	1162	-5.28	-1
Des	ENSMUSG00000026208	chr1	75356904	75365154	+	3754	-5.283	-1
Clec3b	ENSMUSG00000025784	chr9	123060064	123066548	+	990	-5.292	-1
Sidt1	ENSMUSG00000022696	chr16	44240293	44333309	-	5623	-5.294	-1
Tnk1	ENSMUSG00000001583	chr11	69664065	69672232	-	3771	-5.296	-1
n-R5s46	ENSMUSG00000065894	chr14	37669115	37669230	-	116	-5.303	-1
Ikzf3	ENSMUSG00000018168	chr11	98326216	98407345	-	8754	-5.312	-1
Creld2	ENSMUSG00000023272	chr15	88650076	88657111	+	1364	-5.315	-1
3010003L21Rik	ENSMUSG00000085355	chr6	148893389	148895651	+	1747	-5.317	-1
Vipr1	ENSMUSG00000032528	chr9	121551834	121582072	+	5564	-5.318	-1

Satb1	ENSMUSG00000023927	chr17	51875512	51972615	-	12603	-5.32	-1
Guca1a	ENSMUSG00000023982	chr17	47531509	47537533	-	871	-5.321	-1
C920021L13Rik	ENSMUSG00000080727	chr3	95675445	95693016	+	2553	-5.322	-1
2900008C10Rik	ENSMUSG00000087271	chrX	11711585	11737472	-	1355	-5.322	-1
4930555I21Rik	ENSMUSG00000028801	chr4	135050705	135093720	+	3056	-5.327	-1
Tox	ENSMUSG00000041272	chr4	6613500	6918704	-	7609	-5.333	-1
Bckdk	ENSMUSG00000030802	chr7	135047596	135053178	+	3063	-5.336	-1
Fam195a	ENSMUSG00000025732	chr17	26000643	26005683	-	816	-5.339	-1
Zfp771	ENSMUSG00000054716	chr7	134388040	134398317	+	1371	-5.34	-1
Dupd1	ENSMUSG00000063821	chr14	22495804	22533798	-	1093	-5.342	-1
Gm17520	ENSMUSG00000092117	chr1	173353316	173367449	-	954	-5.342	-1
Thap8	ENSMUSG00000013928	chr7	31065113	31075256	+	1184	-5.342	-1
Sult5a1	ENSMUSG00000000739	chr8	125666747	125682215	-	1764	-5.344	-1
Rogdi	ENSMUSG00000022540	chr16	5008823	5013620	-	1431	-5.353	-1
Mme	ENSMUSG00000027820	chr3	63099794	63186153	+	4379	-5.355	-1
Abhd12	ENSMUSG00000032046	chr2	150658229	150730477	-	2823	-5.365	-1
Krt5	ENSMUSG00000061527	chr15	101537501	101543322	-	2190	-5.371	-1
Elmo3	ENSMUSG00000014791	chr8	107829501	107835300	+	3109	-5.374	-1
C030034L19Rik	ENSMUSG00000085362	chr3	9403064	9437233	+	5786	-5.374	-1
Prss16	ENSMUSG00000006179	chr13	22094045	22101611	-	2444	-5.385	-1
D130020L05Rik	ENSMUSG00000042922	chr12	102320691	102327137	+	2431	-5.393	-1
Gpr162	ENSMUSG00000038390	chr6	124808462	124813935	-	3772	-5.401	-1
4921517L17Rik	ENSMUSG00000038085	chr2	156138035	156201374	+	3879	-5.402	-1
Il11ra1	ENSMUSG00000073889	chr4	41647021	41716347	+	4471	-5.405	-1
Slc5a5	ENSMUSG00000000792	chr8	73406788	73416656	-	2928	-5.408	-1
0610011F06Rik	ENSMUSG00000025731	chr17	26012409	26022952	+	1109	-5.417	-1
Gstm4	ENSMUSG00000027890	chr3	107843326	107847812	-	1707	-5.423	-1
Tspan13	ENSMUSG00000020577	chr12	36741144	36769087	-	2474	-5.427	-1
Abcg4	ENSMUSG00000032131	chr9	44081271	44096698	-	5211	-5.427	-1
Endog	ENSMUSG00000015337	chr2	30027013	30029589	+	1063	-5.436	-1
Marveld3	ENSMUSG00000001672	chr8	112471814	112486103	-	3284	-5.443	-1
Sh2d4a	ENSMUSG00000053886	chr8	70800466	70871593	+	2718	-5.443	-1
Ckb	ENSMUSG00000001270	chr12	112907566	112910549	-	1478	-5.446	-1

Hint2	ENSMUSG00000028470	chr4	43667099	43669338	-	1540	-5.451	-1
Gm2301	ENSMUSG00000087516	chr5	120061272	120121374	-	1353	-5.458	-1
Tmem38a	ENSMUSG00000031791	chr8	75096002	75111181	+	2094	-5.463	-1
Rbfox3	ENSMUSG00000025576	chr11	118351074	118772911	-	5820	-5.467	-1
Ehf	ENSMUSG00000012350	chr2	103103590	103143435	-	5576	-5.467	-1
Kcnk3	ENSMUSG00000049265	chr5	30890543	30927643	+	3810	-5.471	-1
Atp6v0e2	ENSMUSG00000039347	chr6	48487568	48491797	+	1832	-5.472	-1
Cox7a1	ENSMUSG00000074218	chr7	30969190	30971047	+	364	-5.476	-1
Plch2	ENSMUSG00000029055	chr4	154357224	154430893	-	8110	-5.482	-1
P2rx6	ENSMUSG00000022758	chr16	17561978	17577893	+	3995	-5.485	-1
Tmem97	ENSMUSG00000037278	chr11	78355319	78364279	-	1375	-5.502	-1
Mmp24	ENSMUSG00000027612	chr2	155601078	155644102	+	4683	-5.509	-1
Chac1	ENSMUSG00000027313	chr2	119176965	119180117	+	1637	-5.509	-1
Lin7b	ENSMUSG00000003872	chr7	52623261	52625953	-	1463	-5.519	-1
Mmp15	ENSMUSG00000031790	chr8	97876168	97898193	+	4348	-5.528	-1
Shroom1	ENSMUSG00000018387	chr11	53270707	53281268	+	4600	-5.53	-1
Gmppb	ENSMUSG00000070284	chr9	107951600	107955132	+	2758	-5.531	-1
Acad10	ENSMUSG00000029456	chr5	122071035	122110523	-	6068	-5.539	-1
Angptl6	ENSMUSG00000038742	chr9	20678253	20684171	-	1581	-5.552	-1
Tmem160	ENSMUSG00000019158	chr7	17038128	17040838	+	814	-5.555	-1
Slc25a23	ENSMUSG00000046329	chr17	57183134	57199286	-	5294	-5.559	-1
Gstm1	ENSMUSG00000058135	chr3	107815173	107820891	-	1394	-5.56	-1
Bcas3	ENSMUSG00000059439	chr11	85166669	85639560	+	6085	-5.565	-1
Ism2	ENSMUSG00000050671	chr12	88619588	88640655	-	2600	-5.57	-1
D730003I15Rik	ENSMUSG00000073478	chr1	193048353	193049565	+	547	-5.578	-1
Ngb	ENSMUSG00000021032	chr12	88438481	88443489	-	1723	-5.579	-1
Gm6665	ENSMUSG00000091561	chr18	31979516	31980067	-	255	-5.579	-1
Nudt22	ENSMUSG00000037349	chr19	7067509	7070527	-	999	-5.58	-1
Prr15	ENSMUSG00000045725	chr6	54277006	54280194	+	1381	-5.59	-1
Mmp7	ENSMUSG00000018623	chr9	7692086	7699585	+	1072	-5.592	-1
Scube2	ENSMUSG00000007279	chr7	116942190	117009193	-	4153	-5.597	-1
Wipf3	ENSMUSG00000086040	chr6	54379597	54453762	+	7114	-5.6	-1
Gstt2	ENSMUSG00000033318	chr10	75294590	75297626	-	1370	-5.607	-1
Ssr4	ENSMUSG00000002014	chrX	71032367	71036169	+	3096	-5.611	-1
Tm7sf2	ENSMUSG00000024799	chr19	6062821	6068373	-	3484	-5.611	-1
n-R5s193	ENSMUSG00000064798	chr4	149191841	149191949	+	109	-5.617	-1
Gm15199	ENSMUSG00000086428	chr10	42460345	42478466	-	542	-5.619	-1

1810049H13Rik	ENSMUSG00000039670	chr11	120317920	120319382	-	813	-5.626	-1
Gstm2-ps1	ENSMUSG00000091578	chr5	117082917	117083471	-	258	-5.633	-1
Tjp3	ENSMUSG00000034917	chr10	80735952	80754012	-	3030	-5.636	-1
Dgcr6	ENSMUSG00000003531	chr16	18052953	18071725	+	2985	-5.637	-1
Gm15884	ENSMUSG00000087327	chr6	124665591	124668849	-	643	-5.638	-1
Cryl1	ENSMUSG00000021947	chr14	57893871	58017333	-	1998	-5.641	-1
Faah	ENSMUSG00000034171	chr4	115639750	115690531	-	7834	-5.643	-1
Guk1	ENSMUSG00000020444	chr11	58997357	59005714	-	1855	-5.652	-1
Adm2	ENSMUSG00000054136	chr15	89153403	89155159	+	1043	-5.666	-1
Bcat2	ENSMUSG00000030826	chr7	52825523	52845080	+	3899	-5.669	-1
Adh1	ENSMUSG00000074207	chr3	137923955	137953662	+	2233	-5.671	-1
Acaa1b	ENSMUSG00000010651	chr9	119057141	119066211	-	1962	-5.687	-1
Nrip3	ENSMUSG00000034825	chr7	116901569	116925539	-	4012	-5.689	-1
Mon1a	ENSMUSG00000032583	chr9	107790460	107805456	+	2081	-5.693	-1
1810019D21Rik	ENSMUSG00000086390	chr8	108659300	108662425	+	1551	-5.695	-1
Cldn7	ENSMUSG00000018569	chr11	69778281	69781387	+	1948	-5.697	-1
Slc39a4	ENSMUSG00000063354	chr15	76442815	76447322	-	2264	-5.704	-1
Fam187b	ENSMUSG00000046826	chr7	31758823	31774739	+	2264	-5.713	-1
Pex11c	ENSMUSG00000069633	chr8	3457105	3467680	-	3397	-5.713	-1
Edem2	ENSMUSG00000038312	chr2	155527413	155555211	-	3463	-5.725	-1
C030014I23Rik	ENSMUSG00000060380	chr9	44286007	44290263	+	2446	-5.728	-1
Syt3	ENSMUSG00000030731	chr7	51639472	51655557	+	3118	-5.74	-1
Gm88	ENSMUSG00000071073	chr17	46391114	46394265	+	1384	-5.741	-1
Fahd2a	ENSMUSG00000027371	chr2	127261951	127270301	-	1463	-5.743	-1
Blnk	ENSMUSG00000061132	chr19	41003417	41069025	-	5991	-5.746	-1
Cd7	ENSMUSG00000025163	chr11	120898061	120900732	-	1133	-5.756	-1
Inmt	ENSMUSG00000003477	chr6	55120620	55125037	-	1600	-5.773	-1
Slc2a4	ENSMUSG00000018566	chr11	69756045	69761690	-	2833	-5.797	-1
Fuz	ENSMUSG00000011658	chr7	52151449	52158001	+	5003	-5.81	-1
Dtx1	ENSMUSG00000029603	chr5	121130211	121161936	-	4663	-5.813	-1
Fxyd2	ENSMUSG00000059412	chr9	45207957	45218361	+	1010	-5.82	-1
Gm16737	ENSMUSG00000086678	chr7	25416164	25422935	-	1184	-5.82	-1
Rab3d	ENSMUSG00000019066	chr9	21711935	21722636	-	4196	-5.824	-1
Wnt6	ENSMUSG00000033227	chr1	74818466	74831893	+	2068	-5.834	-1
Lrrc4	ENSMUSG00000049939	chr6	28611831	28781747	-	4001	-5.849	-1
Nxnl2	ENSMUSG00000021396	chr13	51266394	51270556	+	1163	-5.857	-1
Adck5	ENSMUSG00000022550	chr15	76406788	76426246	+	3452	-5.87	-1
Gm11031	ENSMUSG00000079004	chr11	4999387	4999881	-	60	-5.875	-1

Gm17624	ENSMUSG00000091107	chr11	12548080	12548279	+	200	-5.875		-1
n-R5s47	ENSMUSG00000084465	chr14	63488551	63488669	+	119	-5.875		-1
Actg2	ENSMUSG00000059430	chr6	83462899	83486259	-	1562	-5.876		-1
Fgl1	ENSMUSG00000031594	chr8	42276788	42300510	-	4434	-5.877		-1
1110017F19Rik	ENSMUSG00000075420	chr11	115773331	115775234	+	978	-5.882		-1
2810432L12Rik	ENSMUSG00000039611	chr4	49597378	49610748	-	3325	-5.883		-1
Gcat	ENSMUSG00000006378	chr15	78861304	78873988	+	3851	-5.886		-1
Rspo3	ENSMUSG00000019880	chr10	29172915	29255673	-	2394	-5.897		-1
Snph	ENSMUSG00000027457	chr2	151416285	151458329	-	7861	-5.9		-1
Ppp1r36	ENSMUSG00000052221	chr12	77518586	77540460	+	1435	-5.903		-1
Mpz	ENSMUSG00000056569	chr1	173080842	173091261	+	2111	-5.904		-1
Gm9949	ENSMUSG00000054589	chr18	62339780	62344059	+	962	-5.911		-1
Tmem79	ENSMUSG00000001420	chr3	88132577	88138426	-	2666	-5.924		-1
4631405K08Rik	ENSMUSG00000091593	chr1	195333432	195335997	-	1741	-5.94		-1
Mapk8ip1	ENSMUSG00000027223	chr2	92223833	92241420	-	4644	-5.944		-1
Cd320	ENSMUSG00000002308	chr17	33980036	33986719	+	2592	-5.957		-1
Plekha4	ENSMUSG00000040428	chr7	52781700	52809599	+	4187	-5.973		-1
2610528J11Rik	ENSMUSG00000028536	chr4	118199604	118202833	+	1202	-5.974		-1
Grb7	ENSMUSG00000019312	chr11	98307708	98316687	+	3365	-5.98		-1
B3gnt7	ENSMUSG00000079445	chr1	88199796	88203878	+	2593	-5.988		-1
Tmem121	ENSMUSG00000049036	chr12	114424114	114427731	+	1598	-5.993		-1
Sema4a	ENSMUSG00000028064	chr3	88239881	88265104	-	5406	-5.997		-1
Extl1	ENSMUSG00000028838	chr4	133912287	133939765	-	4333	-6.004		-1
Tmem205	ENSMUSG00000040883	chr9	21725453	21731906	-	785	-6.006		-1
Gstm2	ENSMUSG00000040562	chr3	107784620	107789371	-	1679	-6.01		-1
Slc25a35	ENSMUSG00000018740	chr11	68781633	68787867	+	3677	-6.012		-1
BC051226	ENSMUSG00000092564	chr17	34045127	34046262	-	979	-6.014		-1
Tfap2c	ENSMUSG00000028640	chr2	172375093	172384122	+	3679	-6.022		-1
C1rl	ENSMUSG00000038527	chr6	124443131	124460661	+	3010	-6.024		-1
Smagp	ENSMUSG00000053559	chr15	100451773	100467296	-	1142	-6.026		-1
Fam189a2	ENSMUSG00000071604	chr19	24047241	24105509	-	2547	-6.033		-1

Pnck	ENSMUSG00000002012	chrX	70901333	70905456	-	1971	-6.039	-1
Myh11	ENSMUSG00000018830	chr16	14194620	14291501	-	6632	-6.065	-1
0610040J01Rik	ENSMUSG00000060512	chr5	64203734	64290858	+	1870	-6.07	-1
Kcnk1	ENSMUSG00000033998	chr8	128519002	128554583	+	2301	-6.084	-1
Gm16065	ENSMUSG00000086847	chr5	120135104	120141227	-	1345	-6.087	-1
Aass	ENSMUSG00000029695	chr6	23022173	23082986	-	3817	-6.104	-1
Sema4g	ENSMUSG00000025207	chr19	45063591	45077887	+	4870	-6.111	-1
Tnfsf13	ENSMUSG00000089669	chr11	69496348	69499286	-	1637	-6.112	-1
Lrrc51	ENSMUSG00000064307	chr7	109061498	109082409	-	1210	-6.117	-1
4930524O07Rik	ENSMUSG00000090207	chr9	106794502	106797513	+	1121	-6.132	-1
Gm12216	ENSMUSG00000081769	chr11	53596920	53672758	-	2647	-6.141	-1
Sp5	ENSMUSG00000075304	chr2	70312980	70315786	+	2100	-6.163	-1
Chid1	ENSMUSG00000025512	chr7	148679035	148725756	-	10799	-6.187	-1
St14	ENSMUSG00000031995	chr9	30896987	30939438	-	4577	-6.187	-1
Rab26	ENSMUSG00000079657	chr17	24664186	24671155	-	2852	-6.194	-1
Fkbp11	ENSMUSG00000003355	chr15	98554804	98558629	-	731	-6.195	-1
Gm11730	ENSMUSG00000087057	chr11	116956262	116958381	-	829	-6.198	-1
Nrtn	ENSMUSG00000039481	chr17	56890748	56896953	-	1023	-6.221	-1
Dnase1l2	ENSMUSG00000024136	chr17	24577026	24580050	-	2328	-6.236	-1
Gm13822	ENSMUSG00000087477	chr5	115367327	115373286	-	346	-6.25	-1
Nkx2-6	ENSMUSG00000044186	chr14	69789633	69793596	+	1385	-6.25	-1
Slc25a29	ENSMUSG00000021265	chr12	110064083	110074093	-	2606	-6.269	-1
Stat5a	ENSMUSG00000004043	chr11	100720665	100746483	+	7133	-6.271	-1
Cblc	ENSMUSG00000040525	chr7	20364230	20382158	-	2541	-6.294	-1
Fam174b	ENSMUSG00000078670	chr7	80885193	80921805	+	2678	-6.32	-1
Arhgef16	ENSMUSG00000029032	chr4	153652595	153675785	-	5481	-6.33	-1
Wscd2	ENSMUSG00000063430	chr5	113919467	114039679	+	4490	-6.332	-1
Pdlim4	ENSMUSG00000020388	chr11	53868430	53882516	-	2560	-6.349	-1
Bspry	ENSMUSG00000028392	chr4	62141085	62158332	+	2354	-6.35	-1
Il17re	ENSMUSG00000043088	chr6	113408478	113420745	+	3031	-6.357	-1
Bai1	ENSMUSG00000034730	chr15	74346626	74419873	+	6391	-6.38	-1
Pgm5	ENSMUSG00000041731	chr19	24757506	24936345	-	3611	-6.383	-1
Fbp1	ENSMUSG00000069805	chr13	62966113	62989642	-	1702	-6.387	-1
Slc44a4	ENSMUSG00000007034	chr17	35051411	35067381	+	3113	-6.392	-1
Yipf2	ENSMUSG00000032182	chr9	21393153	21397223	-	2098	-6.397	-1
Upk2	ENSMUSG00000041523	chr9	44260799	44262850	-	873	-6.404	-1
Amt	ENSMUSG00000032607	chr9	108199184	108203902	+	1706	-6.411	-1

Crym	ENSMUSG00000030905	chr7	127329894	127345625	-	3391	-6.416	-1
Gm11724	ENSMUSG00000087669	chr11	117651211	117652627	-	472	-6.425	-1
Gstm6	ENSMUSG00000068762	chr3	107741765	107746667	-	1989	-6.434	-1
Fut9	ENSMUSG00000055373	chr4	25536479	25727391	-	13824	-6.434	-1
Pck2	ENSMUSG00000040618	chr14	56159103	56168854	+	3400	-6.443	-1
Ppm1e	ENSMUSG00000046442	chr11	87040408	87172525	-	6702	-6.444	-1
Adig	ENSMUSG00000044405	chr2	158328348	158333934	+	810	-6.445	-1
Cytl1	ENSMUSG00000062329	chr5	38126758	38131059	+	974	-6.445	-1
Mcoln2	ENSMUSG00000011008	chr3	145812797	145858477	+	3288	-6.445	-1
Ighv9-4	ENSMUSG00000076673	chr12	115538172	115538465	-	294	-6.452	-1
n-R5s151	ENSMUSG00000065893	chr7	17057162	17057280	+	119	-6.452	-1
n-R5s56	ENSMUSG00000064455	chr13	104186282	104186386	+	105	-6.452	-1
Prlh	ENSMUSG00000090550	chr1	92849685	92850589	+	261	-6.452	-1
Upk1b	ENSMUSG00000049436	chr16	38773297	38800316	-	1769	-6.458	-1
Aldh3b2	ENSMUSG00000075296	chr19	3972328	3981645	+	2141	-6.465	-1
Arid3c	ENSMUSG00000066224	chr4	41670868	41678174	-	1772	-6.485	-1
Prss41	ENSMUSG00000024114	chr17	23973752	23981139	-	2586	-6.485	-1
Gm13594	ENSMUSG00000086881	chr2	65076813	65077732	+	476	-6.502	-1
Pdlim3	ENSMUSG00000031636	chr8	46970839	47004900	+	1578	-6.517	-1
Gm10614	ENSMUSG00000074055	chr8	123113431	123117701	+	2538	-6.518	-1
Bcl2l14	ENSMUSG00000030200	chr6	134346336	134388754	+	4093	-6.521	-1
Bckdha	ENSMUSG00000060376	chr7	26414871	26443780	-	1773	-6.532	-1
Hsd17b14	ENSMUSG00000030825	chr7	52810263	52823122	+	2240	-6.543	-1
C1qtnf4	ENSMUSG00000040794	chr2	90725943	90730683	+	1365	-6.613	-1
Ccbl1	ENSMUSG00000039648	chr2	30040644	30061367	-	2923	-6.618	-1
Gm10644	ENSMUSG00000074219	chr8	86456228	86479104	-	2798	-6.631	-1
Pdzk1ip1	ENSMUSG00000028716	chr4	114761313	114766504	+	1778	-6.654	-1
Tmed3	ENSMUSG00000032353	chr9	89594041	89599881	-	1324	-6.656	-1
D730039F16Rik	ENSMUSG00000026870	chr2	34729916	34747652	+	2441	-6.687	-1
Bnipl	ENSMUSG00000028115	chr3	95045193	95055115	-	2297	-6.691	-1
Panx2	ENSMUSG00000058441	chr15	88890156	88903997	+	3609	-6.693	-1
Cbs	ENSMUSG00000024039	chr17	31749568	31774144	-	3343	-6.715	-1
1810019J16Rik	ENSMUSG00000037600	chr4	133074878	133086705	+	2379	-6.715	-1
Sectm1a	ENSMUSG00000025165	chr11	120928721	120942534	-	1692	-6.722	-1
Park2	ENSMUSG00000073465	chr17	11033258	11133829	+	309	-6.739	-1
Kcng1	ENSMUSG00000074575	chr2	168085617	168107236	-	4508	-6.744	-1
Akr1b3	ENSMUSG00000001642	chr6	34253934	34267497	-	3933	-6.744	-1

4930579G22Rik	ENSMUSG00000025535	chr5	130384410	130387150	+	620	-6.754	-1
Slc5a1	ENSMUSG00000011034	chr5	33446868	33505521	+	4118	-6.756	-1
Rab39b	ENSMUSG00000031202	chrX	72817385	72823570	-	3401	-6.762	-1
Prdx6b	ENSMUSG00000050114	chr2	80132629	80135513	+	2885	-6.769	-1
Prdx6	ENSMUSG00000026701	chr1	163170243	163181350	-	3671	-6.786	-1
Gdf15	ENSMUSG00000038508	chr8	73153292	73156355	-	1852	-6.798	-1
Sytl3	ENSMUSG00000041831	chr17	6877808	6942393	+	4665	-6.822	-1
Epn3	ENSMUSG00000010080	chr11	94350913	94361288	-	4067	-6.847	-1
2010107G23Rik	ENSMUSG00000020083	chr10	61570403	61606668	-	2981	-6.861	-1
Igkv3-7	ENSMUSG00000076598	chr6	70557732	70558030	+	299	-6.863	-1
n-R5s40	ENSMUSG00000064409	chr15	79522932	79523055	+	124	-6.863	-1
Gm13031	ENSMUSG00000087698	chr4	140503581	140513817	-	924	-6.865	-1
Rab17	ENSMUSG00000026304	chr1	92854710	92866238	-	1992	-6.873	-1
Nkpd1	ENSMUSG00000060621	chr7	20104080	20110399	+	2753	-6.897	-1
Prr15l	ENSMUSG00000047040	chr11	96789418	96799144	+	3923	-6.902	-1
Ces2e	ENSMUSG00000031886	chr8	107450160	107458572	+	2699	-6.903	-1
Gstm7	ENSMUSG00000004035	chr3	107729252	107734735	-	1601	-6.905	-1
P2rx2	ENSMUSG00000029503	chr5	110768831	110772168	-	1982	-6.907	-1
Acrbp	ENSMUSG00000072770	chr6	124999707	125013285	+	3277	-6.91	-1
Apof	ENSMUSG00000047631	chr10	127705053	127707207	+	1930	-6.919	-1
Gm14097	ENSMUSG00000084839	chr2	132520168	132524259	-	470	-6.924	-1
4930481A15Rik	ENSMUSG00000086938	chr19	5406740	5422847	+	2240	-6.933	-1
Otud7a	ENSMUSG00000033510	chr7	70589637	70903914	+	3619	-6.939	-1
Spnb1	ENSMUSG00000021061	chr12	77681475	77811534	-	12147	-6.95	-1
Rhpn1	ENSMUSG00000022580	chr15	75534710	75544840	+	2813	-6.959	-1
Trbv14	ENSMUSG00000076471	chr6	41085166	41085608	+	339	-6.977	-1
2310042D19Rik	ENSMUSG00000078486	chr4	155590036	155595416	+	3842	-6.981	-1
Cnn1	ENSMUSG00000001349	chr9	21903725	21914074	+	2368	-6.995	-1
Fbxo2	ENSMUSG00000041556	chr4	147534730	147540533	+	1288	-7.009	-1
Nkain4	ENSMUSG00000027574	chr2	180669477	180689404	-	1239	-7.011	-1
Hdac11	ENSMUSG00000034245	chr6	91106659	91124686	+	3849	-7.019	-1
Zpbp2	ENSMUSG00000017195	chr11	98412411	98419979	+	1683	-7.022	-1
Tmem184a	ENSMUSG00000036687	chr5	140278439	140295871	-	5277	-7.05	-1
Hoxb13	ENSMUSG00000049604	chr11	96055630	96058761	+	2206	-7.057	-1
Tpsb2	ENSMUSG00000033825	chr17	25503486	25505804	+	1117	-7.068	-1
2900060B14Rik	ENSMUSG00000073596	chr1	120355247	120355324	-	78	-7.084	-1

Ighv2-9	ENSMUSG00000076651	chr12	115117307	115117740	-	347	-7.084	-1
Stard10	ENSMUSG00000030688	chr7	108465600	108495140	+	2383	-7.094	-1
Igfbp2	ENSMUSG00000039323	chr1	72871077	72899048	+	1847	-7.114	-1
Ckm	ENSMUSG00000030399	chr7	19996443	20006932	+	1415	-7.141	-1
Crb3	ENSMUSG00000044279	chr17	57198528	57205341	+	1564	-7.149	-1
Grin2d	ENSMUSG00000002771	chr7	53087253	53126298	-	5862	-7.183	-1
Ggt1	ENSMUSG00000006345	chr10	75024349	75048945	+	3411	-7.195	-1
Tnnc2	ENSMUSG00000017300	chr2	164602661	164605467	-	800	-7.199	-1
Col4a6	ENSMUSG00000031273	chrX	137599946	137908619	-	8002	-7.209	-1
Upb1	ENSMUSG00000033427	chr10	74869656	74904424	+	6599	-7.219	-1
Kcng4	ENSMUSG00000045246	chr8	122147754	122159580	-	3806	-7.228	-1
Gm13790	ENSMUSG00000086302	chr5	115001298	115004025	+	473	-7.275	-1
Ly6g6d	ENSMUSG00000073413	chr17	35208388	35215010	-	1172	-7.288	-1
3100003L05Rik	ENSMUSG00000086254	chr7	131769184	131852449	-	664	-7.328	-1
Dcxr	ENSMUSG00000039450	chr11	120586713	120588595	-	1144	-7.359	-1
Gm17197	ENSMUSG00000090990	chr19	56438752	56440731	+	452	-7.362	-1
Gpr110	ENSMUSG00000041293	chr17	43407278	43461686	+	3853	-7.371	-1
Tmem132e	ENSMUSG00000020701	chr11	82202402	82259834	+	4544	-7.371	-1
C8g	ENSMUSG00000015083	chr2	25354171	25357239	-	1493	-7.381	-1
Slc18a1	ENSMUSG00000036330	chr8	71561610	71613134	-	5401	-7.438	-1
Gm15775	ENSMUSG00000089820	chr8	126848678	126855695	-	538	-7.444	-1
Gm106	ENSMUSG00000032719	chr1	15843943	15882803	-	2748	-7.498	-1
Fhit	ENSMUSG00000060579	chr14	10382606	11994549	-	1491	-7.516	-1
1700066J03Rik	ENSMUSG00000092091	chr13	58250138	58251053	+	444	-7.521	-1
9130409J20Rik	ENSMUSG00000085683	chr11	66770155	66778518	+	1503	-7.529	-1
Asb16	ENSMUSG00000034768	chr11	102130057	102140776	+	2843	-7.551	-1
Sdf2l1	ENSMUSG00000022769	chr16	17130231	17132476	-	1108	-7.554	-1
Rsph9	ENSMUSG00000023966	chr17	46258984	46281165	-	1602	-7.563	-1
Tesc	ENSMUSG00000029359	chr5	118477752	118511887	+	1591	-7.568	-1
Ttll10	ENSMUSG00000029074	chr4	155408949	155433453	-	3651	-7.57	-1
Banf2	ENSMUSG00000037307	chr2	143858795	143899715	+	571	-7.595	-1
Npw	ENSMUSG00000071230	chr17	24794275	24795370	-	606	-7.595	-1
Tmprss2	ENSMUSG00000000385	chr16	97786291	97832802	-	3159	-7.631	-1
Mycl1	ENSMUSG00000028654	chr4	122672895	122679728	+	4038	-7.677	-1
Ldoc1	ENSMUSG00000057615	chrX	58962789	58964124	+	1336	-7.686	-1
Mtfp1	ENSMUSG00000004748	chr11	3991483	3995448	-	1738	-7.691	-1
BC007180	ENSMUSG00000078161	chr3	154425121	154430751	+	5631	-7.695	-1
Prodh	ENSMUSG00000003526	chr16	18060450	18090296	-	3461	-7.696	-1

Gm16174	ENSMUSG00000087593	chr7	20401902	20403974	+	402	-7.697	-1
Prss8	ENSMUSG00000030800	chr7	135069239	135073627	-	1852	-7.711	-1
Sord	ENSMUSG00000027227	chr2	122060485	122091076	+	2352	-7.722	-1
BC030870	ENSMUSG00000074300	chr8	67610121	67653222	-	658	-7.732	-1
Ap1m2	ENSMUSG00000003309	chr9	21099901	21116777	-	1738	-7.757	-1
Bex4	ENSMUSG00000047844	chrX	132673535	132674982	+	837	-7.796	-1
Gm16283	ENSMUSG00000087020	chr18	49915448	49916843	+	522	-7.796	-1
Gm17592	ENSMUSG00000091640	chr17	13030801	13031000	-	200	-7.796	-1
Xcl1	ENSMUSG00000026573	chr1	166861775	166865658	-	523	-7.796	-1
BC057022	ENSMUSG00000041930	chr5	115018025	115063229	+	3141	-7.826	-1
Cd164l2	ENSMUSG00000028865	chr4	132768046	132780470	+	1659	-7.84	-1
Folr1	ENSMUSG00000001827	chr7	109006845	109019302	-	2297	-7.853	-1
Gm15420	ENSMUSG00000087387	chr17	27772288	27773807	+	382	-7.857	-1
Gm15533	ENSMUSG00000087530	chr7	135146363	135148704	-	465	-7.857	-1
Gm16739	ENSMUSG00000085131	chr5	110351668	110352500	-	411	-7.857	-1
AV064505	ENSMUSG00000086381	chr9	7669429	7695631	-	522	-7.915	-1
Cldn8	ENSMUSG00000050520	chr16	88561073	88563428	-	2356	-7.956	-1
Egf	ENSMUSG00000028017	chr3	129380493	129458240	-	4785	-7.961	-1
Macrod1	ENSMUSG00000036278	chr19	7131258	7272551	+	1274	-7.963	-1
n-R5s115	ENSMUSG00000075862	chr8	126088186	126088304	-	119	-7.972	-1
Lysmd1	ENSMUSG00000053769	chr3	94938010	94943440	+	2394	-7.999	-1
Prlr	ENSMUSG00000005268	chr15	10106993	10303504	+	15494	-8.009	-1
Gm11365	ENSMUSG00000086893	chr13	30032152	30034130	+	774	-8.026	-1
Gm16137	ENSMUSG00000089949	chr15	37172158	37172917	+	664	-8.026	-1
Isoc2b	ENSMUSG00000052605	chr7	4796561	4817795	-	1297	-8.04	-1
Sh3bgr	ENSMUSG00000040666	chr16	96422077	96450540	+	1542	-8.042	-1
Slc7a4	ENSMUSG00000022756	chr16	17572111	17577091	-	3978	-8.096	-1
Gyltl1b	ENSMUSG00000040434	chr2	92205203	92215026	-	5837	-8.136	-1
5330417C22Rik	ENSMUSG00000040412	chr3	108258612	108339444	-	6294	-8.16	-1
Esrrg	ENSMUSG00000026610	chr1	189432670	190038764	+	6208	-8.183	-1
Camk2b	ENSMUSG00000057897	chr11	5869647	5966365	-	9126	-8.199	-1
Camsap3	ENSMUSG00000044433	chr8	3587450	3609074	+	4298	-8.221	-1

AY761184	ENSMUSG00000058618	chr8	22812991	22814117	-		503	-8.225	-1
Gm13066	ENSMUSG00000086949	chr4	148939729	148941899	-		884	-8.225	-1
Pcp4l1	ENSMUSG00000038370	chr1	173103393	173126399	-		1577	-8.23	-1
2610027K06Rik	ENSMUSG00000087013	chr11	85605163	85645725	-		2574	-8.249	-1
2210020M01Rik	ENSMUSG00000048442	chr11	115761280	115767583	+		1649	-8.276	-1
Tmem54	ENSMUSG00000028786	chr4	128782792	128788870	+		1222	-8.284	-1
AI428936	ENSMUSG00000019737	chr7	31099826	31104065	+		2946	-8.328	-1
Gm6434	ENSMUSG00000091233	chr7	26667031	26667438	-		408	-8.399	-1
Myoc	ENSMUSG00000026697	chr1	164569281	164579824	+		2079	-8.4	-1
Gm11535	ENSMUSG00000086337	chr11	96038865	96040114	+		778	-8.402	-1
Pou2f3	ENSMUSG00000032015	chr9	42930711	43018452	-		3955	-8.419	-1
2700046A07Rik	ENSMUSG00000041789	chr18	62911329	62916001	-		2466	-8.438	-1
0610009B14Rik	ENSMUSG00000044062	chr12	81787357	81793453	-		944	-8.44	-1
E030007J07Rik	ENSMUSG00000087183	chr19	40942876	40948561	+		1266	-8.44	-1
Gm9783	ENSMUSG00000043488	chr19	5848508	5851372	+		468	-8.44	-1
Tspan1	ENSMUSG00000028699	chr4	115834474	115840206	-		2329	-8.45	-1
Azgp1	ENSMUSG00000037053	chr5	138422749	138431461	+		1315	-8.458	-1
Gm12	ENSMUSG00000086545	chr11	98459605	98460900	+		902	-8.479	-1
Gm11755	ENSMUSG00000086547	chr11	118984325	118999735	-		891	-8.517	-1
Gjb1	ENSMUSG00000047797	chrX	98571717	98580968	+		1707	-8.567	-1
Sytl1	ENSMUSG00000028860	chr4	132809005	132819028	-		2518	-8.568	-1
Defb42	ENSMUSG00000054763	chr14	63665828	63676986	+		2415	-8.579	-1
9330162012Rik	ENSMUSG00000086448	chr13	25029270	25033286	+		3836	-8.582	-1
Gm6736	ENSMUSG00000071414	chr12	13461465	13462815	-		1351	-8.591	-1
Nxf7	ENSMUSG00000031410	chrX	132114094	132133316	-		2710	-8.669	-1
9130230L23Rik	ENSMUSG00000054598	chr5	66370597	66395524	-		5755	-8.699	-1
Cux2	ENSMUSG00000042589	chr5	122307972	122500111	-		8456	-8.721	-1
Pllp	ENSMUSG00000031775	chr8	97198795	97220142	-		1889	-8.723	-1
Gm13824	ENSMUSG00000086054	chr5	115418793	115448055	+		1374	-8.761	-1

Tmem45b	ENSMUSG00000041737	chr9	31233784	31271823	-		1572	-8.832	-1
Gm11611	ENSMUSG00000087042	chr11	97380926	97383209	-		1326	-8.854	-1
Rab25	ENSMUSG00000008601	chr3	88345951	88352222	-		1673	-8.957	-1
Ppp1r14d	ENSMUSG00000027317	chr2	119043855	119055642	-		852	-8.969	-1
Gm15889	ENSMUSG00000089712	chr8	96901821	96903522	+		388	-8.996	-1
Etnk2	ENSMUSG00000070644	chr1	135260149	135276913	+		4566	-9.001	-1
1810019N24Rik	ENSMUSG00000091727	chr7	13533941	13547117	-		1474	-9.023	-1
Gsdma3	ENSMUSG00000064224	chr11	98487674	98499540	+		1614	-9.023	-1
Magix	ENSMUSG00000031147	chrX	7250290	7258377	-		2910	-9.057	-1
Atp12a	ENSMUSG00000022229	chr14	56983905	57007388	+		3951	-9.191	-1
Clec18a	ENSMUSG00000033633	chr8	113593398	113605988	-		1997	-9.222	-1
Gnmt	ENSMUSG00000002769	chr17	46862613	46866117	-		1206	-9.262	-1
BC107364	ENSMUSG00000046317	chr3	96237711	96256229	-		1219	-9.268	-1
Defb1	ENSMUSG00000044748	chr8	22887071	22905657	+		872	-9.283	-1
Gm9008	ENSMUSG00000072476	chr6	76445472	76447778	-		2307	-9.312	-1
Gpld1	ENSMUSG00000021340	chr13	25035021	25082622	+		3584	-9.35	-1
Car14	ENSMUSG00000038526	chr3	95701691	95708614	-		2543	-9.438	-1
Bglap-rs1	ENSMUSG00000074489	chr3	88172538	88176665	-		2629	-9.549	-1
Opn1sw	ENSMUSG00000058831	chr6	29326671	29338468	-		2557	-9.571	-1
4930526L06Rik	ENSMUSG00000085490	chr19	11271307	11374376	+		1022	-9.589	-1
Gm1110	ENSMUSG00000079644	chr9	26687152	26730666	-		2394	-9.727	-1
Tst	ENSMUSG00000044986	chr15	78229695	78236289	-		1108	-9.744	-1
Hs3st6	ENSMUSG00000039628	chr17	24889933	24895628	+		1357	-9.775	-1
Acta1	ENSMUSG00000031972	chr8	126415669	126418651	-		1446	-9.87	-1
Atp6v1c2	ENSMUSG00000020566	chr12	17291527	17336165	-		2070	-9.888	-1
Hapln4	ENSMUSG00000007594	chr8	72607428	72614761	+		3576	-9.89	-1
Gm436	ENSMUSG00000078505	chr4	144259840	144276271	-		1224	-9.939	-1
Rnase1	ENSMUSG00000035896	chr14	51764677	51766442	-		970	-9.989	-1
Svs1	ENSMUSG00000039215	chr6	48936860	48941724	+		2778	-10.02	-1
Spdef	ENSMUSG00000024215	chr17	27851297	27865896	-		2188	-10.05	-1
Lrrc38	ENSMUSG00000028584	chr4	142939660	142960935	+		2324	-10.1	-1
Cpa2	ENSMUSG00000071553	chr6	30491582	30514476	+		3212	-10.12	-1
Gdf7	ENSMUSG00000037660	chr12	8304740	8308760	-		1407	-10.12	-1
Ovol2	ENSMUSG00000037279	chr2	144130911	144157882	-		2113	-10.15	-1

Prss28	ENSMUSG00000024171	chr17	25445591	25448821	+	1190	-10.16	-1
Fbxo16	ENSMUSG00000034532	chr14	65885538	65942808	+	3734	-10.26	-1
Slc30a2	ENSMUSG00000028836	chr4	133899096	133910399	+	3564	-10.33	-1
Adra2c	ENSMUSG00000045318	chr5	35621215	35624410	+	3196	-10.39	-1
Mt3	ENSMUSG00000031760	chr8	96676507	96678048	+	538	-10.51	-1
Sost	ENSMUSG00000001494	chr11	101823772	101828329	-	2066	-10.58	-1
Fabp6	ENSMUSG00000020405	chr11	43409551	43415042	-	479	-10.62	-1
Nkx3-1	ENSMUSG00000022061	chr14	69808695	69812719	+	3195	-10.63	-1
Fam115e	ENSMUSG00000018656	chr6	42534865	42547691	-	3692	-10.65	-1
4930415O20Rik	ENSMUSG00000022993	chr15	98401452	98420010	+	540	-10.79	-1
Gm12474	ENSMUSG00000053957	chr3	100293587	100327843	+	1410	-10.83	-1
Gm13874	ENSMUSG00000087032	chr2	103017944	103026838	+	1678	-11.01	-1
Tulp2	ENSMUSG00000023467	chr7	52741632	52779002	+	2939	-11.06	-1
Rec8	ENSMUSG00000002324	chr14	56236874	56244232	+	2748	-11.12	-1
Tmod4	ENSMUSG00000005628	chr3	94928398	94933131	+	1717	-11.18	-1
Degs2	ENSMUSG00000021263	chr12	109925002	109940516	-	3574	-11.22	-1
Glb1l3	ENSMUSG00000031966	chr9	26625538	26668408	-	2468	-11.27	-1
Bhlha15	ENSMUSG00000052271	chr5	144951155	144955310	+	3494	-11.28	-1
Pnliprp2	ENSMUSG00000025091	chr19	58834213	58852023	+	1567	-11.29	-1
Agr2	ENSMUSG00000020581	chr12	36719494	36730674	+	1291	-11.31	-1
Slc26a3	ENSMUSG00000001225	chr12	32075736	32158782	+	6369	-11.33	-1
Derl3	ENSMUSG00000009092	chr10	75356143	75358686	+	1336	-11.37	-1
Ly6g6e	ENSMUSG00000013766	chr17	35213847	35215749	+	1273	-11.39	-1
Slc22a1	ENSMUSG00000023829	chr17	12841740	12868704	-	1994	-11.49	-1
2210407C18Rik	ENSMUSG00000037145	chr11	58421706	58429577	-	1399	-11.49	-1
Olfr750	ENSMUSG00000035932	chr14	51689986	51691117	-	1132	-11.51	-1
Golt1a	ENSMUSG00000041717	chr1	135206400	135219603	+	1842	-11.56	-1
Gm5077	ENSMUSG00000079343	chr6	124574584	124587055	-	3061	-11.7	-1
Fcgbp	ENSMUSG00000047730	chr7	28856255	28905881	+	10497	-11.71	-1
Mrap2	ENSMUSG00000042761	chr9	87039140	87078880	+	2258	-12.03	-1
Defb50	ENSMUSG00000058568	chr8	22934011	22941768	+	373	-12.15	-1
Gm13869	ENSMUSG00000086957	chr2	103046366	103050514	-	1054	-12.56	-1
Itln1	ENSMUSG00000038209	chr1	173448253	173465425	-	1141	-12.7	-1
9530002B09Rik	ENSMUSG00000023263	chr4	122366518	122382372	+	803	-12.79	-1

Gsdma	ENSMUSG00000017204	chr11	98525665	98539022	+	2726	-12.99	-1
Aifm3	ENSMUSG00000022763	chr16	17489704	17507578	+	4688	-13.43	-1
Gm10935	ENSMUSG00000078384	chr7	28884679	28887506	-	2828	-13.86	-1
C1rb	ENSMUSG00000092005	chr6	124462790	124531062	+	2166	-13.93	-1
Ms4a5	ENSMUSG00000054523	chr19	11348356	11358303	-	945	-14.12	-1
Gpha2	ENSMUSG00000024784	chr19	6226380	6227768	+	770	-14.2	-1
Kiss1	ENSMUSG00000091251	chr1	135218452	135226478	+	779	-14.22	-1
Pbsn	ENSMUSG00000000003	chrX	75083240	75098962	-	902	-14.58	-1
Chrm1	ENSMUSG00000032773	chr19	8738279	8758077	+	6487	-14.96	-1
Ren1	ENSMUSG00000070645	chr1	135247087	135256902	+	1657	-15.14	-1
9530053A07Rik	ENSMUSG00000078776	chr7	28914485	28949830	+	11843	-15.2	-1
Gpr37l1	ENSMUSG00000026424	chr1	137056811	137064258	-	2269	-15.39	-1
Tgm4	ENSMUSG00000025787	chr9	122943857	122976679	+	3936	-15.53	-1
Gm16063	ENSMUSG00000085708	chr5	120075951	120084473	+	1701	-15.55	-1
Pnliprp1	ENSMUSG00000042179	chr19	58803377	58818659	+	1619	-15.57	-1
9530003J23Rik	ENSMUSG00000020177	chr10	116669293	116675759	-	1687	-16.61	-1
Gm5615	ENSMUSG00000074448	chr9	36339988	36349548	-	2342	-17.99	-1
