# Supplementary material for: Deletion of the p16INK4a tumor suppressor and expression of the androgen receptor induce sarcomatoid carcinomas with signet ring cells in the mouse prostate
Source: PLoS One. 2019 Jan 24;14(1):e0211153. doi: 10.1371/journal.pone.0211153 (PMC6345450; doi:10.1371/journal.pone.0211153)
Supplement: S2 Table — Seven clinical prostatectomy specimens with signet ring prostatic carcinoma component were mounted on one TMA (tissue microarray) and analyzed for AR, p16, CK8, CK5 and SPP1. “+” indicates pathologist determined classification of presence of staining, while “–”indicates pathologist determined absence of staining. (PDF) [file pone.0211153.s005.pdf]

Cellular Properties of Human Prostatic Signet Ring Cell Carcinomas

**Patients/Cases #**	1	2	3	4	5	6	7
Cellular Markers							
AR	+	+	+	+	+	+	+
p16	−	−	+	−	−	−	+
CK8	+	+	+	+	+	+	+
CK5	−	−	−	−	−	−	−
SPP1	+	+	+	−	+	+	+

Supplemental Table 2
